# To Fold or Not to Fold: Diastereomeric Optimization of an α-Helical Antimicrobial Peptide

**DOI:** 10.1021/acs.jmedchem.3c00460

**Published:** 2023-05-25

**Authors:** Hippolyte Personne, Thierry Paschoud, Sofia Fulgencio, Stéphane Baeriswyl, Thilo Köhler, Christian van Delden, Achim Stocker, Sacha Javor, Jean-Louis Reymond

**Affiliations:** † Department of Chemistry, Biochemistry and Pharmaceutical Sciences, University of Bern, Freiestrasse 3, CH-3012 Bern, Switzerland; ‡ Department of Microbiology and Molecular Medicine, University of Geneva, CH-1211 Geneva, Switzerland; § Service of Infectious Diseases, University Hospital of Geneva, CH-1205 Geneva, Switzerland

## Abstract

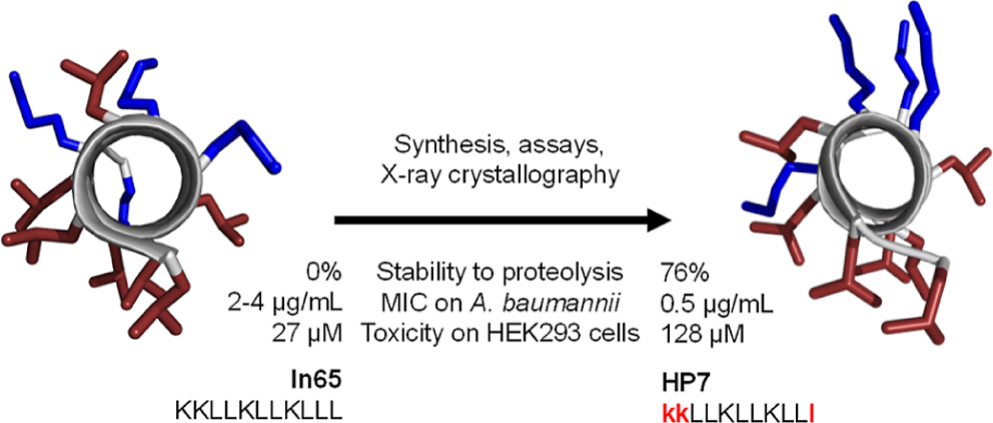

Membrane disruptive α-helical antimicrobial peptides (AMPs) offer an opportunity to address multidrug resistance; however, most AMPs are toxic and unstable in serum. These limitations can be partly overcome by introducing D-residues, which often confers protease resistance and reduces toxicity without affecting antibacterial activity, presumably due to lowered α-helicity. Here, we investigated 31 diastereomers of the α-helical AMP KKLLKLLKLLL. Three diastereomers containing two, three, and four D-residues showed increased antibacterial effects, comparable hemolysis, reduced toxicity against HEK293 cells, and excellent serum stability, while another diastereomer with four D-residues additionally displayed lower hemolysis. X-ray crystallography confirmed that high or low α-helicity as measured by circular dichroism indicated α-helical or disordered structures independently of the number of chirality switched residues. In contrast to previous reports, α-helicity across diastereomers correlated with both antibacterial activity and hemolysis and revealed a complex relationship between stereochemistry, activity, and toxicity, highlighting the potential of diastereomers for property optimization.

## Introduction

Membrane disruptive antimicrobial peptides (AMPs), which occur naturally as part of the innate immune system, offer an opportunity to address multidrug-resistant (MDR) bacteria because of their unspecific mechanism of action, against which resistance does not occur easily.^1−3^ Such AMPs are however unstable in serum and most often toxic owing to their membrane disruptive amphiphilic and usually α-helical structure triggering their antibacterial effect. Their properties can be improved by sequence optimization,^4−7^ whereby the most versatile approach consists in introducing non-natural structural elements^8^ such as d-amino acids,^9−13^ non-natural residues,^14^ β- or γ-amino acids,^15,16^ isopeptide bonds,^17^ or entirely non-peptidic elements such as spermine^18^ or fatty acids.^19,20^ A complete redesign of AMPs is also possible in the form of dimers,^21^ cyclic or bicyclic staples,^22−24^ small molecules,^25^ peptoids,^26,27^ foldamers,^28^ or dendrimers.^29,30^

For α-helical AMPs and analogues, the toxicity reduction effect observed upon introducing D-residues or similar perturbations, often measured as lower lysis of red blood cells, is generally attributed to a reduced α-helical folding, which would block pore formation on the membrane surface as a trigger for hemolysis. On the other hand, coating and destabilization of the bacterial membrane, and therefore the antibacterial effect, would still be possible with the modified peptide in the absence of folding.^12,31−33^ However, very little structural evidence or systematic studies support the hypothesis that reduced α-helical folding should generally preserve antibacterial activity while reducing toxicity.

In our own search for new antibacterial compounds, we have discovered several AMP dendrimers (AMPDs) with very low hemolysis and strong activity against Gram-negative bacteria including MDR clinical isolates.^34−37^ By investigating stereorandomized sequences, which are obtained by solid-phase synthesis using racemic building blocks and consist of a mixture of all possible diastereomers, we found that stereorandomized (*sr-*) AMPDs also exhibit strong antibacterial effects and very low hemolysis, suggesting an intrinsically disordered bioactive conformation.^38,39^ The same effect was observed with the intrinsically disordered AMP indolicidin^40^ but not with α-helical linear AMPs such as DJK-5,^41^ which lost their activity when stereorandomized.^38^

**Figure 1:**
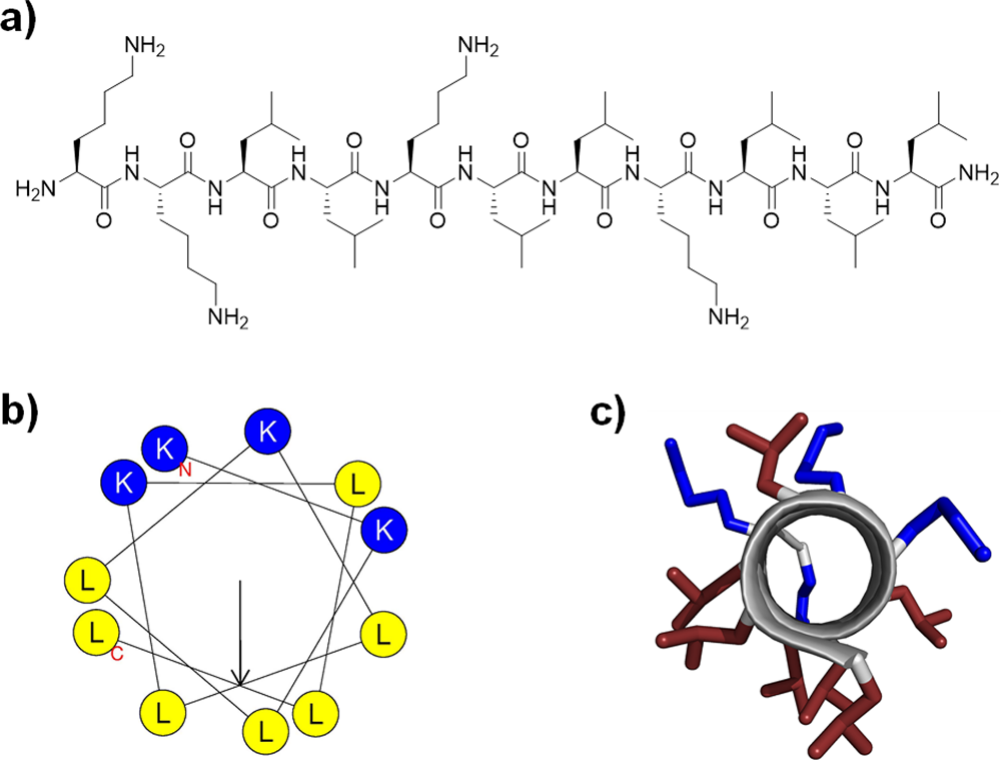
(a) Chemical structure of **ln65**. (b) Helix wheel of **ln65** sequence predicted by HeliQuest.^51^ Blue and yellow indicate, respectively, cationic and hydrophobic residues. The arrow inside the helix wheel indicates the magnitude and direction of the hydrophobic moment. (c) Structure of **ln65** (PDB 7NEF, chain I) obtained by X-ray crystallography of a fucosylated analogue in complex with the bacterial lectin LecB. Cationic side chains are colored in blue, and hydrophobic side chains are colored in red.

In a separate series of experiments with antimicrobial bicyclic peptides,^22,42^ we discovered a short membrane disruptive antibacterial but somewhat hemolytic linear undecapeptide, KKLLKLLKLLL (**ln65**), which did not appear, even as partial sequence, in databases of AMPs,^43,44^ proteins,^45^ or ChEMBL (Figure 1).^46^ The activity of this AMP was preserved upon inverting its four lysine residues to D-enantiomers to form kkLLkLLkLLL (**ln69**), while its hemolysis was strongly reduced.^47^ Strikingly, both the all-L sequence **ln65** and its diastereomer **ln69** were strongly α-helical, as established by circular dichroism (CD) and X-ray crystallography, showing that in this case lowered hemolysis was not related to a reduced α-helical folding. Intrigued by this observation, we set out to prepare and test the stereorandomized version *sr*-**ln65** as well as multiple diastereomers of **ln65** in search for analogues with possibly improved activity and/or reduced toxicity. Systematic studies of multiple diastereomers have shown significant activity modulations in the case of short, non-helical arginine–tryptophan containing AMPs.^48−50^

## Results and Discussion

### Enantiomeric and Stereorandomized Sequences

**Figure 2:**
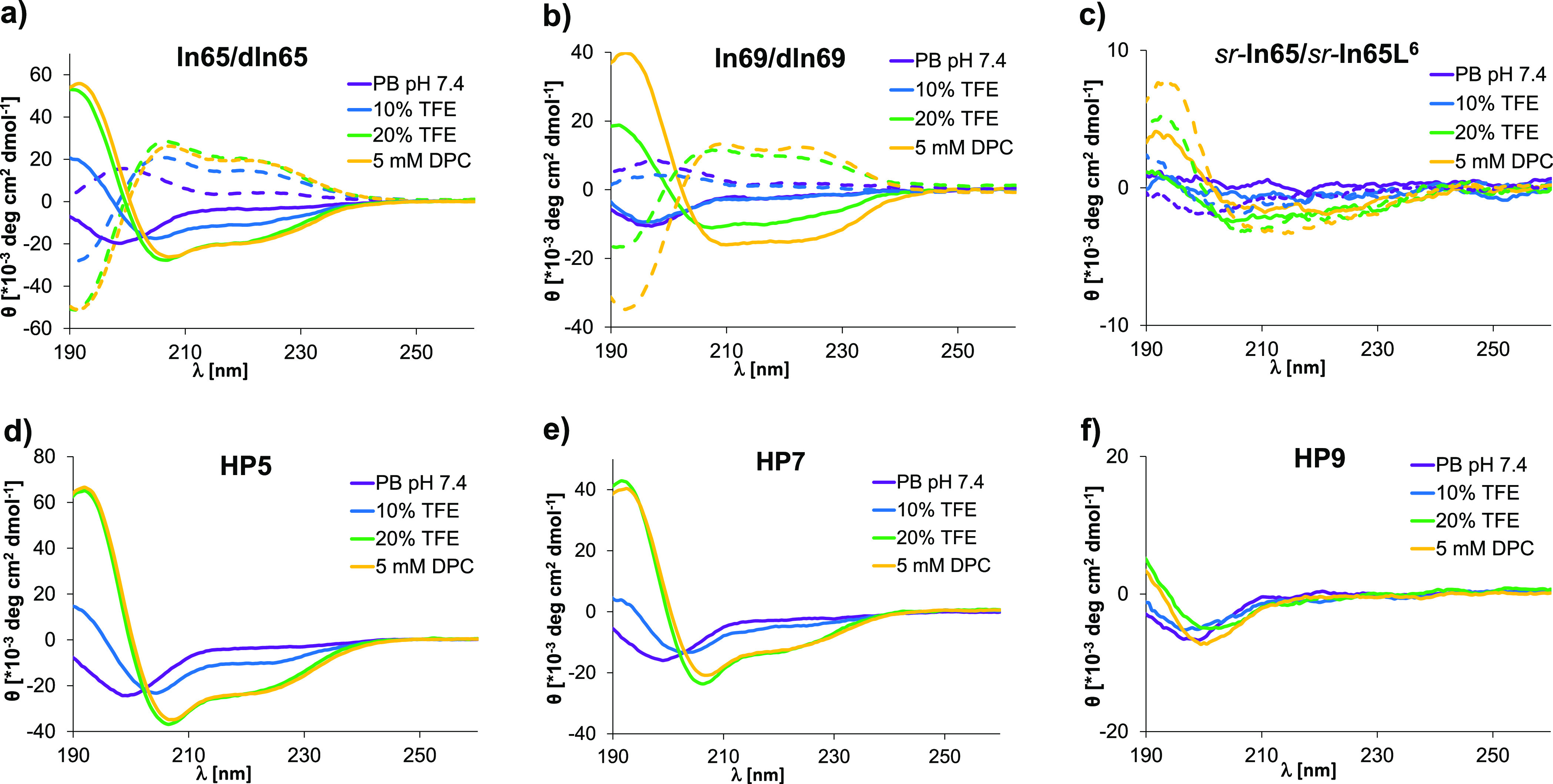
CD-spectra of **ln65**, **dln65**, **ln69**, **dln69**, sr-**ln65**, sr-**ln65L^6^**, **HP5**, **HP7**, and **HP9**, measured with 0.1 mg/mL peptide in phosphate buffer at pH 7.4 with 10 and 20% v/v 2,2,2-TFE and with 5 mM DPC of **(a) ln65** (full lines) and **dln65** (dashed lines), **(b) ln69** (full lines) and **dln69** (dashed lines), (c) sr**-ln65** (full lines) and sr**-ln65L^6^** (dashed lines), **(d) HP5**, **(e) HP7**, and **(f) HP9**.

We first investigated **dln65** and **dln69**, the enantiomers of **ln65** and its diastereomer **ln69**, to check that they displayed similar activities as expected for enantiomeric membrane disruptive AMPs (Table S1). CD spectra of **dln65** and **dln69** in aqueous phosphate buffer in the presence of either 5 mM dodecylphosphocholine (DPC), which forms micelles mimicking a membrane environment,^52^ or 20% trifluoroethanol (TFE) as a folding inducer,^53,54^ were mirror images from those of the L-enantiomers and confirmed their α-helical folding (Figure 2a,b). The enantiomeric pair **ln65**/**dln65** gave essentially the same minimal inhibitory concentration (MIC) values against the five bacterial species used in this study (*Pseudomonas aeruginosa*, *Klebsiella pneumoniae*, *Acinetobacter baumannii*, *Escherichia coli*, and methicillin-resistant *Staphylococcus aureus*), as well as the same minimum hemolytic concentration (MHC) on human red blood cells (hRBCs) indicating significant hemolysis (125 μg/mL, Table 1, Figure S1 and Table S2). In line with these activities, the membrane disruptive effects of both enantiomers on fluorescein-loaded vesicles^55^ made of the anionic egg yolk phosphatidyl glycerol (EYPG) mimicking bacterial membranes as well as on vesicles made of zwitterionic egg yolk phosphatidyl choline (EYPC) mimicking eukaryotic membranes were comparably strong (Table 1, columns 10 and 11 and Figure S2). A similar behavior of **ln65** and its enantiomer **dln65** was consistent with membrane disruption as the primary mechanism of action for these α-helical AMPs. On the other hand, despite the mirror image CD-spectra and comparable vesicle leakage activities of **ln69** and **dln69**, **dln69** was four-fold more antibacterial and hemolytic than **ln69**, which might reflect an additional activity of **dln69** unrelated to its membrane activity.

**Table 1:** Activity of Linear AMPs

			MIC (μg/mL)^c^							
Cpd.	sequence^a^	α-helix content (%)^b^	*E. coli* W3110	*P. aeruginosa* PAO1	*A. baumannii* ATCC19606	*K. pneumoniae* NCTC418	*S. aureus* COL MRSA	MHC^d^ (μg/mL)	EYPG vesicle leakage (%)^e^	EYPC vesicle leakage (%)^e^
**ln65**	KKLLKLLKLLL	73	4	2–4	2–4	4	4	125	79	85
**dln65**	**kkllkllklll**	67	2–4	2–4	4	4	2–4	125	82	90
**ln69**	**kk**LL**k**LL**k**LLL	61	4	8	2–4	8	16	1000	98	26
**dln69**	KK**ll**K**ll**K**lll**	59	0.5–1	2–4	2	4	2	250	94	24
*sr***-ln65**	KKLLKLLKLLL	10	4	4	4	16–32	8	1000	90	22
*sr***-ln65L^6^**	KKLLKLLKLLL	17	4	8	8	16–32	16	2000	91	12
Diastereomers of **ln65**										
**HP1**	K**k**LLKLLKLLL	73	2	4	4	4–8	2	<15.6	73	87
**HP2**	**kk**LLKLLKLLL	69	4	4	4	2–4	4	<15.6	70	87
**HP3**	K**k**LL**k**LLKLLL	69	2	4	2	8	2–4	<15.6	90	55
**HP4**	K**k**L**l**KLLKLLL	46	2–4	2–4	2–4	2	2–4	<15.6	74	83
**HP5**	**k**KLLKLLKLL**l**	90	0.5	2	0.5	2	2	62.5	64	39
**HP6**	KKLLK**ll**KLLL	29	2	8	4	64	16	500	90	9
**HP7**	**kk**LLKLLKLL**l**	60	0.5	2	0.5	4	2	125	90	28
**HP8**	K**kll**KLLKLLL	37	2	4	2–4	4	4	<15.6	91	63
**HP9**	KKLL**kll**KLLL	10	8	64	>64	>64	>64	31.3	59	5
**HP10**	**kk**LL**k**LLKLLL	90	2	4	2	4	2	<15.6	82	54
**HP11**	K**kll**KL**l**KLLL	52	2	2–4	2	4–8	2	62.5	94	43
**HP12**	K**kll**K**l**LKLLL	17	2–4	4–8	8	8–16	4	250	97	34
**HP13**	KKLL**kllk**LLL	9	4–8	16–32	16–32	>64	>64	1000	42	8
**HP14**	KK**ll**K**ll**KLLL	13	2	8	4–8	>64	16–32	1000	95	10
**HP15**	KK**l**L**k**L**l**K**l**LL	6	8	16	32–64	>64	>64	>2000	34	3
**HP16**	KKL**lk**LL**kl**LL	8	4	32	32–64	8–16	>64	125	50	2
**HP17**	K**kl**LKLLK**ll**L	16	8	16	32	32–64	32–64	1000	20	12
**HP18**	**kk**LLKLLKL**ll**	63	4–8	8	8	>64	16–32	1000	61	11
**HP19**	**kk**LL**k**LLKLL**l**	55	2–4	8	4	16	8	1000	95	9
**HP20**	KK**ll**KLL**kl**LL	15	8	16	32–64	>64	>64	1000	58	12
**HP21**	K**kl**LK**l**LK**l**LL	11	4	8	32	16–32	16–32	>2000	44	5
**HP22**	KK**ll**KL**l**KL**l**L	23	2	8	8	>64	16	>2000	76	5
**HP23**	KKL**lk**LL**k**LL**l**	10	2	4–8	>64	>64	32	>2000	51	10
**HP24**	K**kll**K**ll**KLLL	7	4–8	16	8	>64	32	250	81	13
**HP25**	KK**ll**K**ll**K**l**LL	12	8–16	8–16	32	>64	>64	>2000	70	4
**HP26**	**kk**LL**k**LLKL**ll**	41	4	4	16	>64	32	>2000	68	6
**HP27**	**kk**LL**k**LL**k**LL**l**	23	4	8	32	>64	32	>2000	31	5
**HP28**	**k**KLL**kll**KLL**l**	10	2–4	4	64	>64	32	1000	29	3
**HP29**	KKL**lkllk**LLL	7	2	4	8	64	32	250	53	6
**HP30**	K**k**L**l**K**l**L**k**L**l**L	7	8	8–16	32	>64	64	>2000	11	10
**HP31**	**k**K**l**L**k**L**l**K**l**L**l**	5	8	8	32	>64	32	>2000	22	9
Lys → Arg and Leu → Ile Analogues of **ln65**/**ln69** and Dimers										
**HP32**	RRLLRLLRLLL	62	4–8	8–16	4	4–8	4–8	15.6	30	99
**HP33**	**rr**LL**r**LL**r**LLL	63	4–8	4–8	4	4–8	2–4	125	85	56
**HP34**	KKIIKIIKIII	68	32	>64	8–16	>64	>64	62.5	95	10
**HP35**	**kk**II**k**II**k**III	22	4	16	8	>64	>64	125	98	13
**HP36**	RRIIRIIRIII	60	16	64	8–16	16	16–32	62.5	99	39
**HP37**	**rr**II**r**II**r**III	50	8	4–8	8–16	32–64	8–16	250	98	8
**2ln65**	(KKLLKLLKLLL)_2_	91	>64	>64	>64	>64	>64	<15.6	73	40
**2ln69**	(**kk**LL**k**LL**k**LLL)_2_	82	>64	>64	>64	>64	>64	<15.6	71	74

a One letter for amino acids. d-amino acids are shown in lower case and bold, and stereorandomized residues (ratio 1:1 of L and D) are underlined.

b Values are corresponding to data recorded by CD for the condition 5 mM DPC in 7 mM PB buffer (pH 7.4). Percentage of the α-helix content were extracted from using Dichroweb.^58^ (Contin LL method, set 4^59^).

c MICs were determined after incubation in Mueller–Hinton (MH) broth (pH 7.4) for 16–20 h at 37 °C. Values represent two independent duplicates of MIC determinations.

d MHC measured on human red blood cells in PBS (pH 7.4) after 4 h incubation at room temperature.

e Lipid vesicles made of EYPG or EYPC were suspended in buffer (10 mM TRIS, 107 mM NaCl, pH 7.4). After 45 s, the indicated compound was added at the desired concentration and after 240 s, 30 μL of Triton X-100 1.2% was added for full fluorescein release. The percentage leakage observed with 10 μg/mL of compound is given. See the Supporting Information for full curves.

To further probe if α-helical folding was required for activity, we prepared the fully stereorandomized sequence *sr*-**ln65**, a racemic mixture of the 1024 possible diastereomers, as well as *sr*-**ln65L^6^** with pure l-leucine at position 6 of the sequence, containing all 1024 diastereomers with single chirality at position 6 such as to make a possible folding detectable by CD. Remarkably, both *sr*-**ln65** and *sr*-**ln65L^6^** were as antibacterial as **ln65** but much less hemolytic, an effect comparable to our previous observation with AMPDs and *sr*-AMDPs, suggesting that the antibacterial bioactive conformation of **ln65** might be disordered while the hemolytic bioactive conformation would be α-helical.^38,39^ However, while CD spectra of *sr*-**ln65** were nearly flat as expected because the stereorandomized sequence is racemic, those of *sr*-**ln65L^6^** showed approximately 17% α-helix content in 5 mM DPC or with TFE, suggesting that a significant fraction of the 1024 possible diastereomers of **ln65** might be α-helical (Figure 2c). Therefore, the activity of *sr*-**ln65** might also be explained by the presence of some highly active and α-helical diastereomers, such as **ln69**, mixed with inactive and possibly disordered diastereomers.

### Diastereomers and Mutants of ln65

In view of these preliminary experiments, we set out to test a series of diastereomers of **ln65** for their α-helicity and antibacterial and hemolytic effects. From the 1024 possible diastereomers, 11 (0.1%) sequences are possible with a single inverted chirality residue, 55 (5.4%) with two, 165 (16.1%) with three, 330 (32.2%, including **ln69**) with four, and 462 (45.1%) with five inverted chirality residues. Balancing our interest to investigate diastereomers with multiple D-residues related to **ln69** with the expectation that α-helical folding was more likely to be preserved with only a few inverted chirality residues,^56,57^ we selected 31 diastereomers **HP1**–**HP31**, one (3%) with a single D-residue, five (16%) with two D-residues, four (13%) with three D-residues, 13 (42%) with four D-resides, and eight (26%) with five D-residues, distributing D-residues in groups or scattered, at N- or C-termini, or in the middle of the sequence (Table 1).

Many of these diastereomers showed substantial α-helical folding in their CD spectra recorded in 5 mM DPC (Table 1, Figures 2d–f, S1 and Table S2). The average α-helicity decreased with increasing D-residues from 73% for **ln65** and **HP1** (zero and one D-residues), to 61 ± 24% for **HP2**–**HP6** (two D-residues), 49 ± 34% for **HP7**–**HP10** (three D-residues), 23 ± 20% for **HP11**–**HP23** (four D-residues), and 14 ± 12% for **HP24**–**HP31** (five D-residues). Assuming that these average α-helicity values were representative of the average across all **ln65** diastereomers with the corresponding number of D-residues giving a predicted weighted average α-helicity of 26% for *sr*-**ln65L^6^**, slightly above the measured 17%.

Diastereomers with one, two, or three D-residues (**HP1**–**HP10**) generally showed activities comparable to the full L peptide **ln65** against the five bacterial strains (MIC = 0.5–8 μg/mL) but were slightly more hemolytic (MHC = 15.6–62.5 μg/mL) than **ln65**. Notable exceptions were **HP6**, which was less active than **ln65** against *K. pneumoniae* (MIC = 64 μg/mL) and MRSA (MIC = 16 μg/mL) and less hemolytic (MHC = 500 μg/mL), and **HP9**, which had much weaker antibacterial effects than **ln65** (MIC = 8–>64 μg/mL) but was quite hemolytic (MHC = 31.3 μg/mL). **HP6** and **HP9** both had a relatively low α-helicity (29% and 10%). On the other hand, **HP5** (2 D-residues) and **HP7** (3 D-residues) stood out in this series as particularly antibacterial (MIC = 0.5–4 μg/mL) although somewhat hemolytic diastereomers (MHC = 62.5–125 μg/mL). Both peptides completely killed bacteria within 1 h in the time-kill assay as expected for membrane disruptive compounds (Figure S3). Furthermore, EYPG vesicle leakage activities of **HP5** and **HP7** were strong in line with antibacterial effects. Except for the non-helical but hemolytic **HP9**, EYPC vesicle leakage activities varied in line with hemolysis, consistent with a membrane disruptive activity.

Diastereomers with four and five D-residues (**HP11**–**HP31**) were generally less active against bacteria, especially against *A. baumannii*, *K. pneumoniae*, and MRSA, although they all kept significant EYPG vesicle leakage activities, reflecting the fact that vesicle leakage activity is often not sufficient for antibacterial effects to occur due to the much more complex nature of bacteria compared to lipid vesicles. Furthermore, these diastereomers mostly lost their hemolytic activity in proportion to their low EYPC vesicle leakage activities, except for **HP16**, **HP24**, and **HP29**, which, like **HP9**, showed significant hemolysis despite being non-helical and inactive on EYPC vesicles. The least active peptides were **HP13** with four D-residues and **HP25** with five D-residues. Both peptides retained some activity against *E. coli*, *P. aeruginosa*, and *A. baumannii* (MIC = 4–32 μg/mL) but were inactive against *K. pneumoniae* and MRSA, were non-hemolytic, and were not α-helical (7 and 11% in 5 mM DPC). Gratifyingly, one peptide with four D-residues, **HP19**, was as strongly antibacterial and low hemolytic as the previously identified **ln69** with four D-residues. Another peptide with four D-residues, **HP11** (MIC = 2–8 μg/mL), was even slightly more antibacterial than **ln69**, although slightly more hemolytic (MHC = 62.5 μg/mL). **HP11** and **HP19** were among the most α-helical in this set (52–55% in 5 mM DPC) although not as much as **ln69** (61%).

To compare the effects of diastereomeric changes with more classical sequence variations, we performed conservative mutations in **ln65** and **ln69** by mutating all lysines to arginines, all leucines to isoleucines, or both, preserving their chirality pattern. In this series, the Lys → Arg exchanges (**ln65** → **HP32** and **ln69** → **HP33**) preserved α-helicity, antibacterial activity, and EYPG vesicle leakage, but increased hemolysis and EYPC vesicle leakage, which might be related to the better cell-penetrating properties of poly-arginines versus poly-lysines attributed to stronger binding to phospholipids.^60^ On the other hand, Leu → Ile exchanges (**ln65** → **HP34**, **ln69** → **HP35**, **HP32** → **HP36**, **HP33** → **HP37**) led to reduced antibacterial effects and in part lower hemolysis, accompanied by slightly lower α-helicity as expected since Leu stabilizes and Ile destabilizes α-helices.^61^ Surprisingly, dimerization of **ln65** to **2ln65** and **ln69** to **2ln69** gave peptides that were strongly α-helical and hemolytic but entirely inactive against bacteria. Vesicle leakage activities were generally high for EYPG vesicles and partially followed hemolysis trends for EYPC.

**Figure 3:**
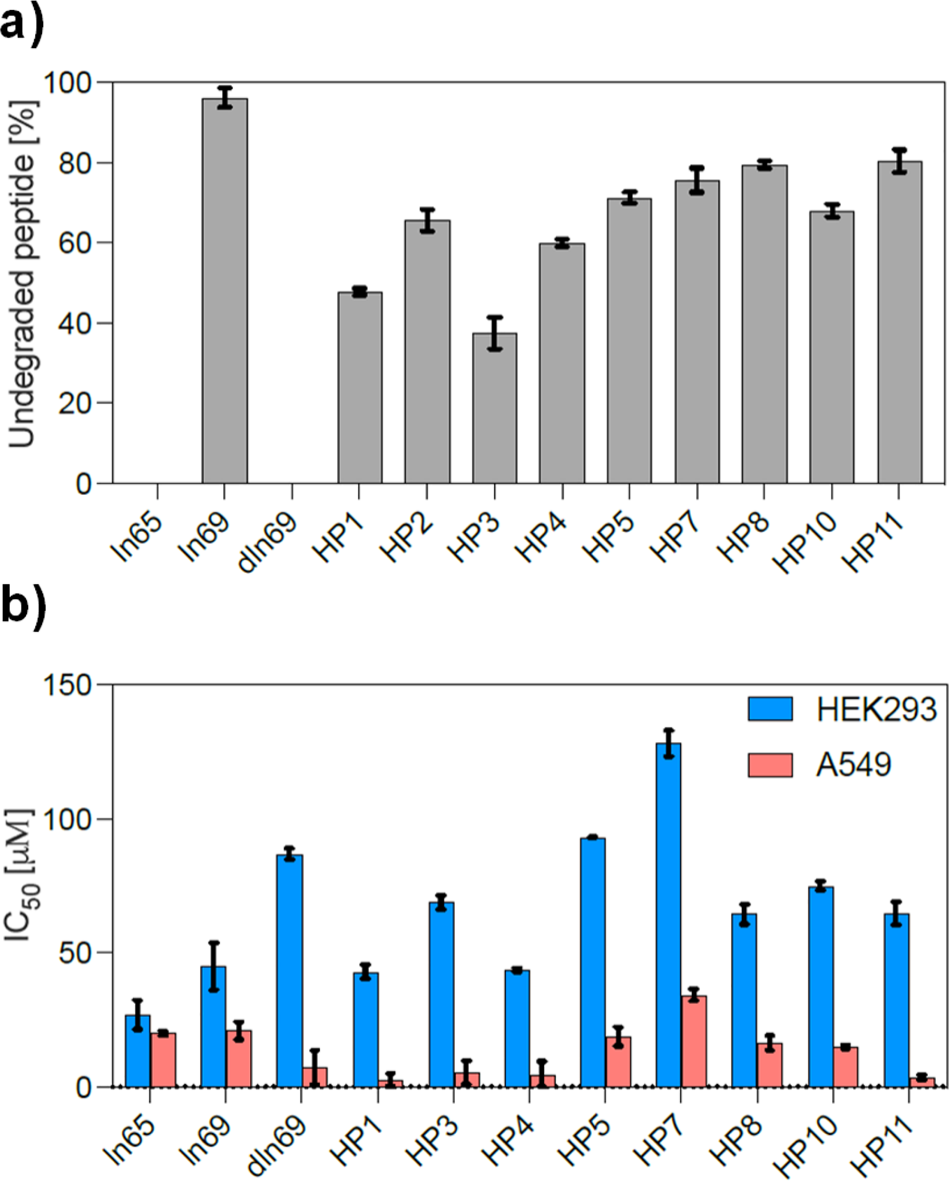
(a) Percentage of undegraded peptide after 24 h incubation in 12.5% human serum in TRIS buffer (pH 7.4) at 37 °C. Data are presented in mean ± SD, n = 3. See the Supporting Information for full curves. (b) Toxicity on HEK293 and A549 cells represented as the IC_50_ measured by Alamar blue assay after 24 h treatments with concentrations of peptide ranging from 0 to 200 μM. Data are presented as the mean ± SD, *n* = 3. See the Supporting Information for all data and procedure.

Taken together, these experiments showed that diastereomers of **ln65** featured new analogues with interesting activity profiles, while other simple modifications such as Lys → Arg, Leu → Ile mutations or dimerization were not as profitable. For further evaluation, we selected the most strongly antibacterial diastereomeric AMPs irrespective of their hemolytic properties, namely, **ln65**, **ln69**, **dln69**, and all diastereomers **HP1**–**HP11** except **HP6** and **HP9**. These AMPs showed good activities (MIC = 2–8 μg/mL) against additional Gram-negative and Gram-positive bacteria including several drug-resistant *P. aeruginosa* variants,^62^ although none of them were active against *Burkholderia cenocepacia*, a Gram-negative bacterium which is naturally resistant to AMPs like colicin (Table 2).^63^ Furthermore, most diastereomers were much more stable against serum degradation than the full L-sequence **ln65** (Figure 3a). Interestingly, inverting the chirality of only the *N*- and *C*-termini (**ln65** → **HP5**) was sufficient to entirely stabilize the peptide in line with the non-recognition of d-amino acids by proteases preventing the proteolysis from peptide extremities. On the other hand, **dln69** with 7 d-leucine residues was entirely degraded due to proteolytic scission at the N-terminal l-lysine residue presumably from trypsin-like proteases (Figure S4).

**Table 2:** Antimicrobial Activity of Diastereomeric AMPs^e^

Cpd.	PA14^b^	PA14 4.13 (*phoQ*)^a^,^b^	PA14 4.18 (*pmrB*)^a^,^b^	PA14 2P4 (*pmrB*)^a^,^b^	ZEM-1A^a^^,^^c^	ZEM9A^a^^,^^c^	*K. pneumoniae* Oxa-48^a^^,^^c^	*Enterobacter cloacae*^a^^,^^c^	*Stenotrophomonas maltophilia*^a^^,^^c^	*Burkolderia cenocepacia*^a^^,^^c^	*Staphylococcus epidermis*^c^^,^^d^	*S. aureus* Newman^d^
**ln65**	2–4	4	32	32	4	4	2	2	2	>64	2	2
**ln69**	2	4	16	32	2	8	4	4	2	>64	4	8
**dln69**	1	2	8	16	4	8	8	4	2	>64	4	1
**HP1**	2	4	32	64	2	4	8	2	4	>64	2	2
**HP2**	2	4	32	64	2	4	2	2	2	>64	2	2
**HP3**	4	8	32	32	2	8	4	4	4	>64	4	2
**HP4**	2	4	32	64	2	4	2	2	2	>64	2	2
**HP5**	2	4	16	32	2	4	2	2	2	>64	2	2
**HP7**	2	4	16	16	1	4	4	2	2	>64	2	4
**HP8**	2	4	16	32	2	8	4	4	2	>64	4	4
**HP10**	2	4	16	32	2	4	2	2	2	>64	2	2
**HP11**	1	2	8	16	2	8	4	4	4	>64	4	2
**Pol B**	<0.125	0.25	1	1	<0.125	2	2	1	0.5	>16		
**Vancomycin**											0.5	0.5

a Gram-negative strains.

b Strains carrying spontaneous mutations in the indicated genes, all leading to polymyxin B resistance.

c MDR strains.

d Gram-positive strains.

e MIC were determined after incubation in MH broth pH 7.4 for 16–20 h at 37 °C. Values represent two independent duplicate MIC determinations.

While most of these strongly antibacterial diastereomers were equally or more hemolytic than the full L-sequence **ln65**, they showed reduced cytotoxicity against human embryonic kidney HEK293 cells (Figure 3b). Diastereomer **HP7**, which was the most active AMP against bacteria, showed the lowest toxicity in the series (IC_50_ = 128 ± 5 μM). Furthermore, diastereomers were generally toxic against A549 lung cancer cells, with **HP11** showing the strongest toxicity (IC_50_ = 3.6 ± 0.1 μM), in line with the fact that many AMPs are often active against cancer cells (Figures S5 and S6).^64,65^ The observed differences between diastereomers in hemolysis, toxicity against HEK293 cells or A549 lung cancer cells, are probably caused by diastereomeric interactions with the different membrane components of the different cell types and possibly proteins in the cell culture medium.^66^

### X-ray Crystallography

To establish whether the CD signal observed with diastereomeric AMPs was indeed caused by α-helical folding, we prepared derivatives with their *N*-termini acylated with an α-C-fucosylacetyl group for crystallization as complexes with lectin LecB,^67^ an approach which we have successfully used for oligonucleotides,^68^ cyclic,^69^ bicyclic,^47^ and linear peptides,^70^ as well as for peptide dendrimers.^71,72^ We considered the nine most potent diastereomers detailed above, the Lys → Arg mutants **HP32** and **HP33**, and the inactive, alternating chirality diastereomers **HP30** and **HP31**. Crystallization screening provided good diffracting LecB crystals with well-resolved ligand electron density for complexes with the fucosylated analogues **FHP5**, **FHP8**, **FHP30**, and **FHP31** (Table 3).

**Table 3:** X-ray Crystallography of Mixed-Chirality AMPs

Cpd.	sequence^a^	conditions	composition	PDB ID
**FHP5**	(*)**k**KLLKLLKLL**l**	crystal screen G7	0.1 M HEPES pH 7.5, 20% v/v Jeffamine M-600	8AN9
**FHP8**	(*)K**kll**KLLKLLL	index screen D8	0.1 M HEPES pH 7.5, 25% w/v polyethylene glycol 3350	8ANO
**FHP30**	(*)K**k**L**l**K**l**L**k**L**l**L	index screen H4	0.2 M ammonium citrate tribasic pH 7.0, 20% w/v polyethylene glycol 3350	8ANR
**FHP31**	(*)**k**K**l**L**k**L**l**K**l**L**l**	index screen G8	0.2 M ammonium acetate, 0.1 M HEPES pH 7.5, 25% w/v polyethylene glycol 3350	8AOO

a One letter code for amino acids. * = α-l-fucosyl-acetyl. Many high-quality LecB crystals were also obtained in complex with fucosylated **ln65R** and **ln69r**; however, in these two cases, electron density only revealed the l-fucose and the adjacent two arginine residues.

**Figure 4:**
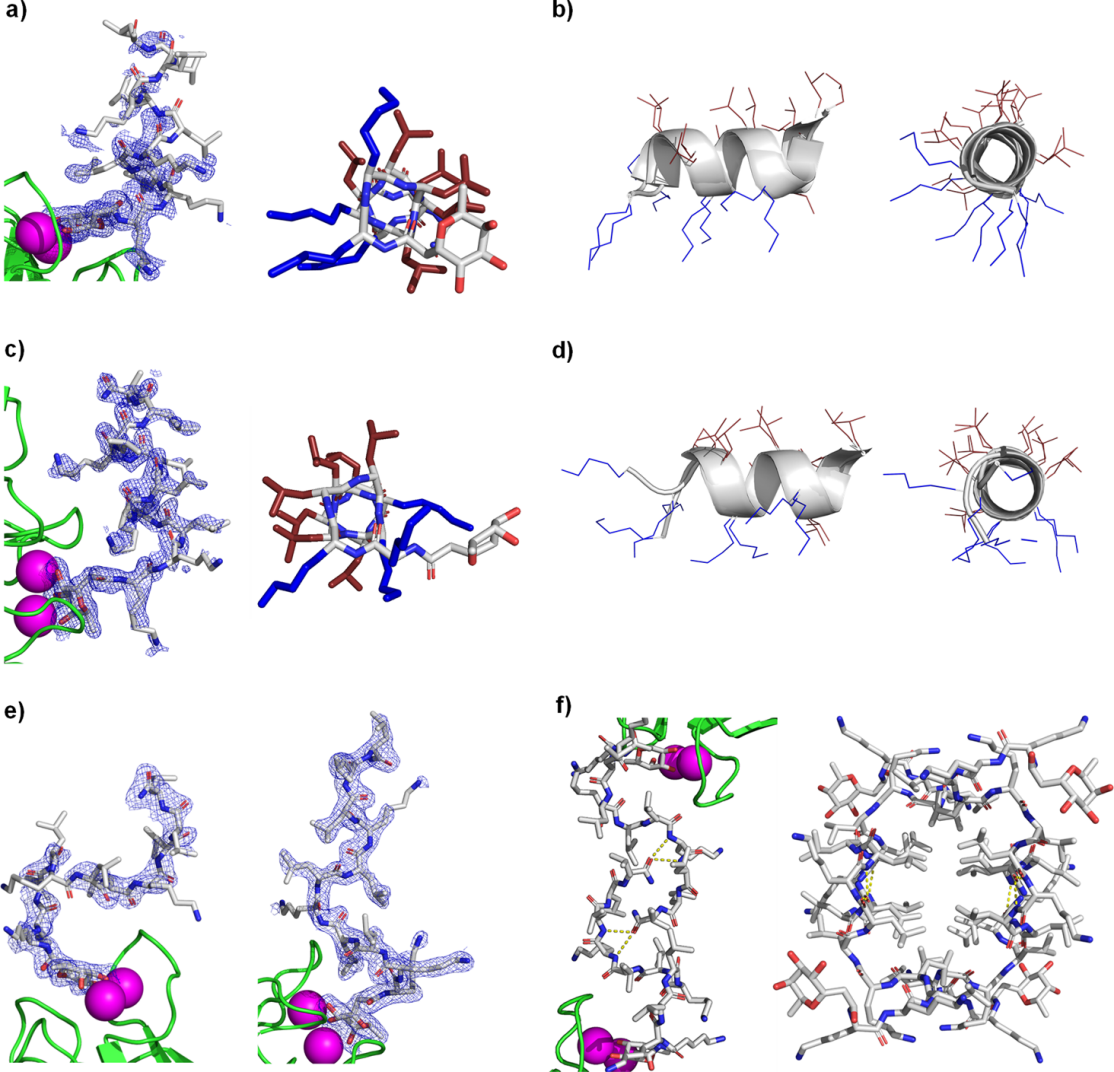
X-ray crystallography of mixed-chirality AMPs. (a) X-ray crystal structure of the **FHP5·**LecB complex (PDB 8AN9, chain E). Left panel: peptide is represented in stick, Ca^2+^ atoms in magenta spheres, and LecB in green cartoon. Blue mesh represents electron density (0.5σ level). Right panel: Stick model of the **FHP5** crystal structure, lysine side chains shown in blue, and leucine side chains shown in red. (b) Superposition of the two complete non-equivalent peptides in the unit cell of PDB 8AN9. Fucose is omitted for more clarity. (c) Same as (a) for X-ray structure of the **FHP8·** LecB complex (PDB 8ANO, chain H). Electron density is shown for a 0.7σ level. (d) Same as (b) for the two complete non-equivalent peptides in the unit cell of PDB 8ANO. (e) X-ray structures of the two different asymmetric peptides in the **FHP30**·LecB complex (PDB 8ANR). Same color code as shown in (a). Electron densities are shown for a 1.0σ level. (f) Left panel: H-bonds between two symmetrical **FHP30** chains. Right panel: full bundle of four symmetrical **FHP30** chains. Lectin monomers and calcium atoms were omitted for clarity in the right panel. Same color code as shown in (a).

In the X-ray crystal structure of **FHP5** in complex with LecB, the undecapeptide was visible in full α-helical conformation in two of the four different fucose-binding sites present in the asymmetric unit, while the other two fucose-binding sites only showed electron density for the fucosyl group, probably due to a disordered conformation (PDB 8AN9, 1.3 Å resolution, Figure 4a, Table S3 and Figure S7). The two α-helices in the well-resolved binding sites are superimposable and interact through intermolecular hydrophobic interactions between leucine side chains (Figure 4b). We observed a similar situation for the structure of **FHP8** in complex with LecB (PDB 8ANO, 1.3 Å resolution, Figure 4c,d, Table S4 and Figure S8). Both structures were very similar to the previously reported structure of fucosylated **dln69** with LecB.^47^

Although the inactive undecapeptides **HP30** and **HP31** with alternating L- and D-residues in their sequences had almost the same number of L- and D-residues, their flat CD spectra most likely indicated a disordered conformation considering that an excess of just one chiral residue was sufficient to indicate folding with *sr*-**ln65L^6^**. Indeed, the structure containing two asymmetric units of the LecB complex with **FHP30** showed two different undefined structures, one forming a four members bundle maintained by H-bonds and hydrophobic interactions between the four symmetric peptides and the other one forming H-bonds with LecB (PDB 8ANR, 1.6 Å resolution, Figure 4e,f, Table S5 and Figure S9). A similar situation was observed in the LecB complex with fucosylated **HP31** containing four different asymmetric units. In this case, only two of them were completely resolved and showed unordered conformations interacting with LecB via H-bonds but also with symmetrical peptides (PDB 8AOO, 1.2 Å resolution, Table S6 and Figure S10).

### Molecular Dynamics

**Figure 5:**
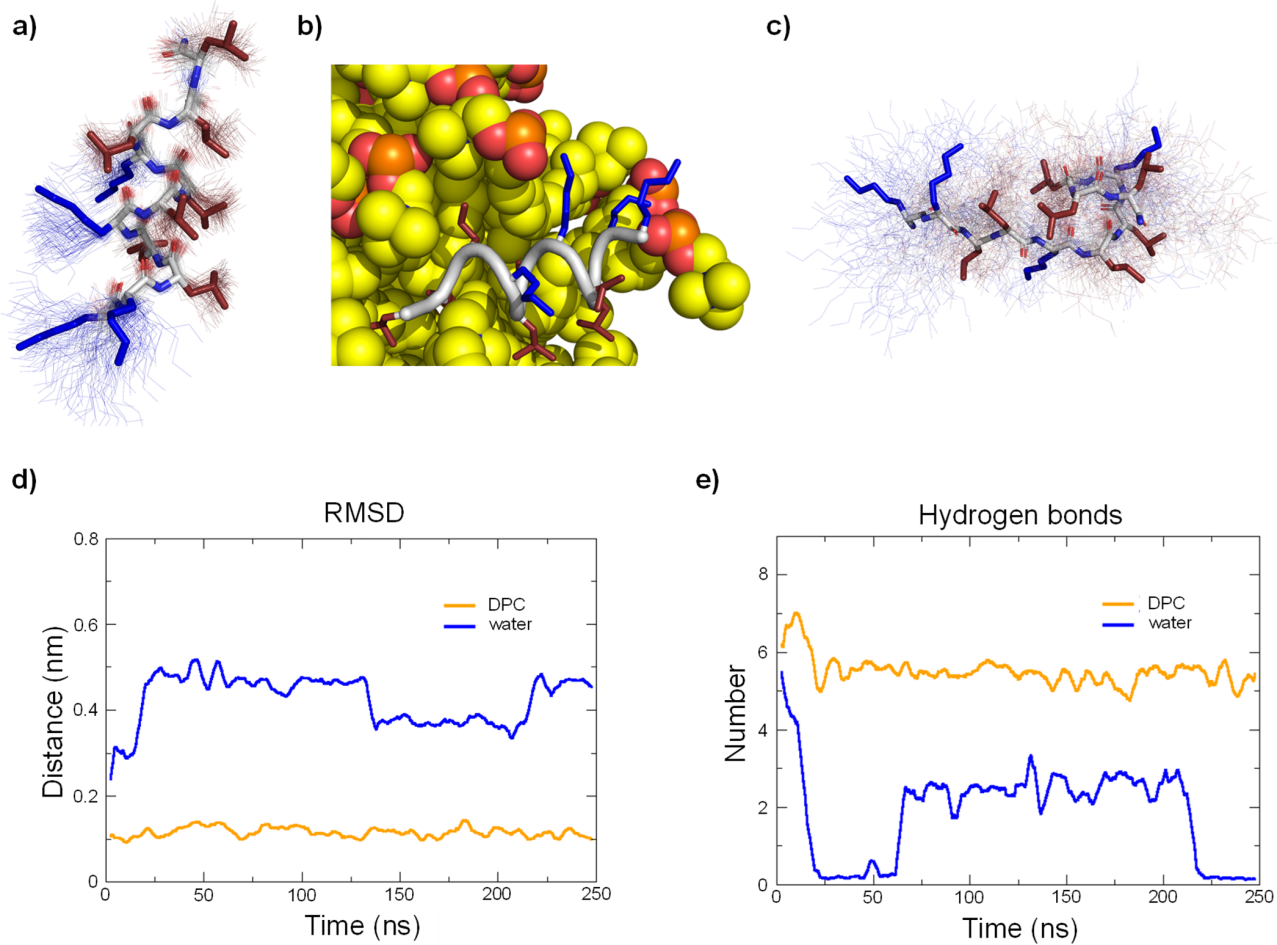
MD simulations of **HP5** with and without DPC micelle. (a) Average structure (stick model) in the presence of DPC micelle over 100 structures sampled during the last 100 ns (thin lines). Hydrophobic side chains are colored in red, and cationic side chains are colored in blue. DPC micelle was omitted for clarity. (b) Last frame of the 250 ns run with DPC micelle. Peptide backbone is in gray cartoon, cationic side chains are colored in blue, hydrophobic side chains are colored in red, and DPC molecules are represented in spheres. (c) Same as (a) for run in water. (d) Comparison of root-mean-square deviation of the peptide backbone relative to the starting coordinates of the α-helix built in PyMol between run with DPC and run in water. (e) Comparison of the number of intramolecular backbone hydrogen bonds between run with DPC and run in water.

To further investigate the α-helical folding of our diastereomers, we performed molecular dynamics (MD) simulations over 250 ns using GROMACS^73^ starting from a pre-folded α-helical structure in water with or without a DPC micelle. For active diastereomers such as **HP5** in the presence of DPC micelles, the peptide first entered in contact with the micelle surface by salt bridges between lysine side chain ε-ammonium groups and phosphate groups of DPC and later remained in an α-helical conformation at the micelle surface (Figure 5a,b). The peptide did not deviate significantly from the starting α-helical conformation (Figure 5d) and retained the full set of backbone H-bonds (Figure 5e).

In water by contrast, the α-helix of **HP5** completely and irreversibly unfolded to an unordered conformation (Figure 5c). This unordered conformation strongly differed from the starting α-helix (Figure 5a) with complete loss of backbone H-bonds (Figure 5b). Similar results were obtained for the other active compounds (**HP1**, **HP2**, **HP3**, **HP4**, **HP7**, **HP8**, **HP10**, **and HP11**, Figures S11–S19). For the inactive, non-helical diastereomers **HP16** and **HP29** by contrast, the starting α-helical conformation rapidly unfolded to an unordered conformation with complete loss of backbone H-bonds even in the presence of the DPC micelle (Figures S20 and S21).

### Statistical Analysis

**Figure 6:**
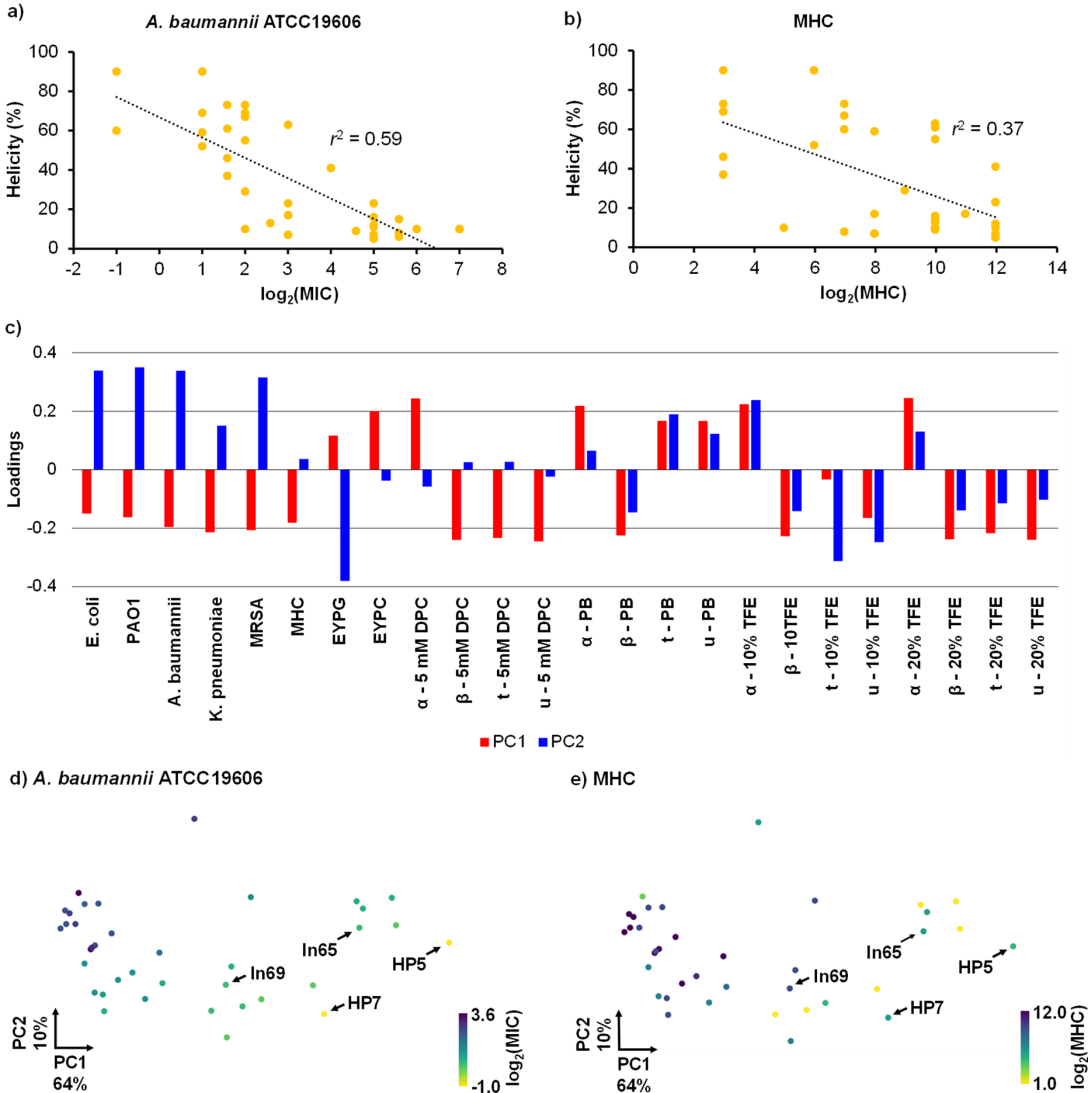
Statistical analysis of data set measured on **ln65** derivatives. (a) Scatter plot of % helicity in 5 mM DPC against log_2_(MIC) for *A. baumannii* ATCC19606. (b) Same as (a) for log2(MHC). (c) Loading analysis of principal components 1 and 2. α = α-helix, β = β-sheet, t = turn, and u = unordered. Visualization of the (PC1 and PC2) plane. Each point represents one compound and is color coded depending on (d) activity on *A. baumannii* and (e) hemolytic activity.

In view of the structural studies above showing in several cases that the degree of α-helicity of **ln65** diastereomers as measured by CD corresponded to an observable α-helix or structural disorder, we assumed that the CD signal could be used as indication of folding across the entire series. Strikingly, increasing α-helicity (% in 5 mM DPC) was linearly correlated with increasing antibacterial activity measured as log_2_(MIC) against *K. pneumoniae* (*r*^2^ = 0.57), *A. baumannii* (*r*^2^ = 0.59), and MRSA (*r*^2^ = 0.62), but to a lesser extent with activity against *P. aeruginosa* (*r*^2^ = 0.41) and with hemolysis (*r*^2^ = 0.37), and only quite poorly with activity against *E. coli* (*r*^2^ = 0.29) against which most diastereomers were active (Figures 6a,b and S22).

To gain an overview of the series, we performed principal component analysis of the complete data set of antimicrobial activity, hemolysis, and folding under the different conditions measured. The first principal component PC1 covered 64% of data variance and reflected the variation of antimicrobial activities, hemolysis, and vesicle leakage activities with α-helicity measured in any of the four conditions (Figure 6c). The second principal component PC2 covered another 10% of data variance and reflected a modulation of antimicrobial activities with α-helicity and EYPG vesicle leakage independent of hemolysis and EYPC vesicle leakage. The distribution of the diastereomers on the (PC1 and PC2) plane separated active from inactive compounds from right to left and separated the two most active diastereomers identified, **HP5** and **HP7**, from the majority of tested diastereomers (Figures 6d,e and S23). Both optimized AMPs stood out by their increased antimicrobial activity, which was particularly strong against *A. baumannii*, while keeping a moderate level of hemolysis, and reduced toxicity against HEK293 cells.

## Conclusions

The above experiments with diastereomers of undecapeptide **ln65** supported by X-ray crystallography show that this α-helical AMP preserves folding and activity across many of its diastereomers. In contrast to previous studies of diastereomers focused on cases with reduction in toxicity and preservation of antibacterial effects, our study across a broad set of diastereomers shows that introducing D-residues in an α-helical AMP can affect antibacterial effects at least as much as toxicity as measured by hemolysis. Although these activities were correlated, sufficient variability was available to identify two diastereomers with improved properties, **HP5** and **HP7**, as two AMPs with increased antibacterial effects compared to the full L-AMP **ln65**, and moderate hemolysis and reduced toxicity against HEK293 cells.

In the present study, the preservation of folding and activity across many diastereomers of **ln65** was anticipated by characterizing its stereorandomized version *sr*-**ln65L^6^**, which showed only modest reduction in antibacterial effects and significant α-helicity. Identifying active diastereomers might be more difficult for other α-helical AMPs if they lose their activity in the stereorandomized form as reported for DJK-5 (vqwrairvrvir) and SB1 (KYKKALKKLAKLL).^38^ Testing the stereorandomized sequence might therefore be the first step to address diastereomeric optimization of other AMPs.

## Experimental Section

### Peptide Synthesis

#### Materials and Reagents

*N*,*N*-dimethylformamide (DMF) was purchased from Thommen-Furler AG. Ethyl cyanohydroxyiminoacetate (Oxyma Pure) was purchased from SENN AG. *N*,*N*′-diisopropyl carbodiimide (DIC) was purchased from Iris BIOTECH GMBH. Piperazine, butanol, and 1,8-diazabicyclo[5.4.0]undec-7-ene (DBU) were purchased from Alfa Aesar. Triisopropylsilane and trifluoroacetic acid (TFA) were purchased from Fluorochem Ltd. d-amino acids were purchased from GL Biochem Shanghai Ltd, and l-amino acids were purchased from Shanghai Space Peptides Pharmaceuticals Co., Ltd. Chemicals were used as supplied, and solvents were of technical grade. Amino acids were used as the following derivatives: Fmoc-Leu-OH, Fmoc-(D)-Leu-OH, Fmoc-Lys(Boc)-OH, Fmoc-(D)-Lys(Boc)-OH, Fmoc-Ile-OH, Fmoc-Arg(Pbf)-OH, and Fmoc-D-Arg(Pbf)-OH. Tentagel S RAM resin was purchased from RAPP Polymer. Analytical RP-HPLC–MS was performed with an Ultimate 3000 Rapid Separation LC–MS System (DAD-3000RS diode array detector) using an Acclaim RSLC 120 C18 column (2.2 μm, 120 Å, 3 × 50 mm, flow 1.2 mL/min) from Dionex. The HPLC is directly linked to a Thermo Scientific LCQ-Fleet Ion-trap MS. Data recording and processing were carried out with Dionex Chromeleon Management System Version 6.80 (analytical RP-HPLC) and FreeStyle software. All RP-HPLC were using HPLC-grade acetonitrile and Milli-Q deionized water. The elution solutions were A: MilliQ deionized water containing 0.05% TFA and D: MilliQ deionized water/acetonitrile (10:90, v/v) containing 0.05% TFA. Preparative RP-HPLC was performed with a Waters automatic Prep LC Controller System containing the four following modules: Waters2489 UV/vis detector, Waters2545 pump, Waters Fraction Collector III, and Waters 2707 Autosampler. A Dr. Maisch GmbH Reprospher column (C18-DE, 100 × 30 mm, particle size 5 μm, pore size 100 Å, and flow rate 40 mL/min) was used. Compounds were detected by UV absorption at 214 nm using a Waters 248 Tunable Absorbance Detector. Data recording and processing were performed with Waters ChromScope version 1.40 from Waters Corporation. MS spectra, recorded on a Thermo Scientific LTQ OrbitrapXL, were provided by the MS analytical service of the Department of Chemistry, Biochemistry and Pharmaceutical Sciences at the University of Bern (group of PD Dr. Stefan Schürch).

#### Solid-Phase Peptide Synthesis

Peptides were synthesized manually using Tentagel S RAM resin (0.22–0.25 mmol/g) and standard Fmoc solid phase peptide synthesis at 60 °C under nitrogen bubbling. The resin was swollen in DMF during 10 min. Double deprotections of the Fmoc group were performed using a solution of 5% w/v piperazine/2% DBU with 10% of butanol in DMF for 1 and 4 min. The resin was washed five times (5 × 8 mL DMF) after deprotection. Double couplings (2 × 8 min) were performed with 3 mL of amino acid (0.2 M), 2 mL of DIC (0.8 M), and 1.5 mL of Oxyma (0.8 M) in DMF. Resin was washed twice (2 × 8 mL DMF) between couplings and three times (3 × 8 mL DMF) after second coupling. The reaction mixture was removed by filtration, and the resin was washed with DMF and MeOH before cleavage.

#### On-Beads Sugar Coupling and Deprotection for Fucosylated Compounds

Peracetylated α-l-fucosyl-acetic acid (3 equiv), Oxyma (3 equiv), and DIC (3 equiv) were dissolved in 6 mL of DMF. Double coupling (2 × 1 h) was performed at 50 °C under nitrogen bubbling on resin. Deacetylation of sugar was performed directly on-bead using a mixture of MeOH/H_2_O/NH_3_ (8:1:1, v/v/v). Reaction was stirred overnight at room temperature. The reaction mixture was removed by filtration, and the resin was washed with DMF and MeOH before cleavage.

#### Cleavage from the Resin

Cleavage was carried out by treating the resins with 7 mL of a TFA/TIS/H_2_O (94:5:1, v/v/v) solution for 3 h at room temperature. The peptide solutions were precipitated with 25 mL of cold TBME, centrifuged for 10 min at 3500 rpm, evaporated, and dried with argon.

#### Purification and Characterization

The dried crude was dissolved in a water/ACN mixture, filtered (pore size 0.22 μm), and purified by preparative RP-HPLC with gradients of 60 min. Fractions were analyzed by analytical LCMS. Peptides were obtained as white foamy solids after lyophilization and analyzed by both LCMS and HRMS. Yields were calculated for the TFA salts. All compounds are >95% pure by HPLC.

### CD Spectroscopy

CD experiments were measured on a Jasco J-715 spectropolarimeter. All the experiments were performed using Hellma Suprasil 110-QS 0.1 cm cuvettes. For each peptide, the measurements were performed in phosphate buffer (PB, pH = 7.4, 7 mM), 10% TFE, 20% TFE, 5 mM DPC. The buffer was degassed for 10 min under high vacuum before each set of experiments. The concentration of the peptides was 0.100 mg/mL, and each sample was measured in one accumulation. The scan rate was 20 nm/min, pitch was 0.5 nm, response was 16 s, and bandwidth was 1.0 nm. The nitrogen flow was kept >8 L/min. After each measurement, the cuvettes were washed successively with milli-Q H_2_O and PB (pH 7.4). The baseline was recorded under the same conditions and subtracted manually. Primary CD spectra were analyzed using DichroWeb^58^ and Contin-LL method (set 4).^74^

### Antimicrobial Activity

Antimicrobial activity was determined for all peptides on *E. coli* W3110, *P. aeruginosa* PAO1, *A. baumannii* ATCC19606, *K. pneumoniae* NCTC418, and methicillin-resistant *S. aureus* COL and for selected peptides on *P. aeruginosa* PA14 and the polymyxin B-resistant derivatives PA14 4.13, PA14 4.18, and PA14 2P4 as well as the clinical isolates ZEM-1A and ZEM9A, *K. pneumoniae* OXA-48, *Enterobacter cloacae*, *Stenotrophomonas maltophilia*, *B. cenocepacia*, and *Staphylococus epidermis*. To determine the MIC, the broth microdilution method was used.^75^ A single bacterial colony was grown in LB medium overnight at 37 °C and 180 rpm shaking. The compounds were prepared as stock solutions of 2 mg/mL in sterilized milliQ deionized water, added to the first well of 96-well sterile, polypropylene round-bottom microtiter plates (TPP, untreated, Corning Incorporated, Kennebunk, USA), and diluted serially by 1/2. The concentration range tested was 0.5–64 μg/mL. The bacterial inoculum was prepared by measuring the absorbance of the overnight culture, which was diluted to concentration of the bacteria was quantified by measuring absorbance at 600 nm and diluted to an OD_600_ of 0.022 in MH medium pH 7.4 (Sigma-Aldrich, Buchs, Switzerland). The sample solutions (150 μL) were mixed with 4 μL of diluted bacterial suspension with a final inoculation of about 5 × 10^5^ CFU. For each test, two columns of the plate were kept for sterility control (MH medium only) and growth control (MH medium with bacterial inoculum, no compound). Positive control was carried out using either polymyxin B for Gram-negative or vancomycin for Gram-positive strains (starting with a concentration of 16 μg/mL) in MH medium. The plates were incubated at 37 °C for 16–20 h under static conditions. 15 μL of 3-(4,5-dimethylthiazol-2-yl)-2,5-diphenyltetrazolium bromide (MTT)^76^ (1 mg/mL in sterilized milliQ deionized water) was added to each well, and the plates were incubated at room temperature until MTT staining was completed. The MIC was defined as the lowest concentration of the dendrimer that inhibits the visible growth of the tested bacteria (yellow) with the unaided eye.

### Hemolysis Assay

To determine the MHC stock solutions, 8 mg/mL peptide in PBS (pH 7.4) was prepared, and 50 μL was diluted serially by l/2 in 50 μL of PBS (pH 7.4) in a 96-well plate (Costar or Nunc, polystyrene, untreated). The concentration range tested was 15.6–2000 μg/mL. hRBCs were obtained by centrifugation of 1.5 mL of whole blood, from the blood bank of Bern, at 3000 rpm for 15 min at 4 °C. Plasma was discarded, and the pellet was re-suspended in a 15 mL Falcon tube in 5 mL of PBS. The washing was repeated three times, and the remaining pellet was re-suspended in 10 mL of PBS. The hRBC suspension (50 μL) was added to each well, and the plate was incubated at room temperature for 4 h. MHC end points were determined by visual determination of the wells after the incubation period. Controls on each plate included a blank medium control (50 μL PBS + 50 μL of hRBCs suspension) and a hemolytic activity control (mQ-deionized water 50 μL + 50 μL hRBC suspension).

### Vesicle Leakage Assay

5(6)-carboxyfluorescein (CF) was purchased from Sigma. EYPC, EYPG, and a Mini-Extruder were purchased from Avanti Polar Lipids. Egg PC or Egg PG thin lipid layers were prepared by evaporating a solution of 100 mg of EYPC or EYPG in 4 mL of MeOH/CHCl_3_ (1:1) on a rotary evaporator at room temperature and then dried in vacuo overnight. The resulting film was then hydrated with 2 mL of CF buffer (50 mM CF, 10 mM TRIS, 10 mM NaCl, pH 7.4) for 30 min at room temperature under stirring and then subjected to freeze–thaw cycles (7×) and extrusion (15×) through a polycarbonate membrane (pore size 100 nm). Extravesicular components were removed by gel filtration (Sephadex G-50) with 10 mM TRIS, 107 mM NaCl, pH 7.4 buffer. Final conditions: ∼2.5 mM PC or PG; inside: 50 mM CF, 10 mM TRIS, 10 mM NaCl, pH 7.4 buffer; outside: 10 mM TRIS, 107 mM NaCl, pH 7.4. PC or PG stock solutions (37.5 μL) were diluted to 3000 μL with a buffer (10 mM TRIS, 107 mM NaCl, pH 7.4) in a thermostated fluorescence cuvette (25 °C) and gently stirred (final lipid concentration ∼31 μM). The CF efflux was monitored at λ_em_ 517 nm (λ_ex_ 492 nm) as a function of time after addition of the desired volume of peptide from 2 mg/mL stock in mQ water at *t* = 45 s, 10 and 50 μg/mL were monitored for both EYPC and EYPG. Finally, 30 μL of 1.2% Triton X-100 was added to the cuvette (0.012% final concentration) at *t* = 240 s to reach the maximum intensity. Fluorescence intensities were then normalized to the maximal emission intensity using *I*(*t*) = (*I*_*t*_ – *I*_0_)/(*I*_∞_ – *I*_0_) where *I*_0_ = *I*_*t*_ at peptide addition, *I*_∞_ = *I*_t_ at saturation of lysis.

### Time-Killing Kinetic Assay

A single colony of *P. aeruginosa* PAO1 was picked and grown overnight with shaking (180 rpm) in LB (Sigma-Aldrich, Buchs, Switzerland) medium 5 mL overnight at 37 °C. The overnight bacterial culture was diluted to OD_600_ 0.002 (2 × 10^6^ CFU/mL) in fresh MH medium. Stock solutions of AMPs in sterilized milliQ water were prepared in 1 mg/mL and were diluted to two times more than required concentration in fresh MH (Sigma-Aldrich, Buchs, Switzerland) medium at pH 7.4. 100 μL of prepared bacteria solution in MH and 100 μL of samples in MH were mixed in a 96-well microtiter plate (TPP, untreated, Corning Incorporated, Kennebunk, USA). Untreated bacteria at 1 × 10^6^ CFU/mL were used as a growth control. 96-well microtiter plates were incubated in 37 °C with shaking (180 rpm). Surviving bacteria were quantified at 0, 0.5, 1, 2, 3, 4, 5, and 6 h by plating 10-fold dilutions of the sample in sterilized normal saline on LB agar plates. LB agar plates were incubated at 37 °C for 10 h, and the number of individual colonies was counted at each timepoint. The assay was performed in triplicate in the biosafety level 2 lab.

### Serum Stability Assay

Human serum was diluted in 0.1 M filtered TRIS buffer (pH 7.4) (25%, 1:3, v/v). Selected peptides were diluted in 0.1 M filtered TRIS buffer (pH 7.4) to a concentration of 400 μM, and 0.1 mg/mL 4- hydroxybenzoic acid was added as an internal standard. Aliquots of peptide solution (50 μL) were added to aliquots of serum (50 μL) in sterile Eppendorf tubes, to reach a peptide concentration of 200 μM during the assay. Samples were incubated at 37 °C under gentle stirring (350 rpm). Different samples (triplicates) were quenched at different time points (0/1/6/12/24 h) by precipitating serum proteins through the addition of ZnSO_4_·7 H_2_O/ACN (1:1) (0.1 M, 100 μL) and cooling in ice bath for 10 min. Protein precipitates were pelleted under centrifugation, and supernatants were then sampled and analyzed by LC–MS. Experiment controls included two references, one known to be degraded and one known to be undegraded. Peaks corresponding to the internal standard and the undegraded peptides were integrated, with the ratio peptide/standard at *t* = 0 h as 100%.

### Cytotoxicity Assay

#### Cell Culture

The A549 human lung adenocarcinoma cells are derived from a patient and were kindly given to us by Dr. Georgia Konstatinidou (Pharmacology Institute, Bern University). HEK293T cells were obtained from ATCC (CRL-11268). A549 and HEK293 were cultured in an incubator at 37 °C with 5% CO_2_ in RPMI-1640 (Gibco) and DMEM (Gibco) containing 10% fetal bovine serum (Thermo Fisher), 100 I.U./mL penicillin, and 100 μg/mL streptomycin (Gibco).

#### Cell-Viability Assay

The viability of the cells was assessed with an AlamarBlue assay (ThermoFisher). Cells were seeded into 96-well plates, 4000 cells/well (HEK293) and 8000 cells/well (A549), the day before the experiment. Cells were then treated with the increasing concentration of the compound and incubated for 24 h at 37 °C in the presence of 5% CO_2_. The next day, the medium was removed and replaced by a 10% AlamarBlue solution in full growth medium (DMEM or RPMI-1640). The cells were incubated for 3–5 h at 37 °C with 5% CO_2_ in a humidified atmosphere. The fluorescence was then measured on a Tecan Infinite M1000 Pro plate reader at λ_ex_ 560 nm and λ_em_ 590 nm. The value was normalized according to the untreated cells.

### Crystallography Experiment and Data Acquisition

Suitable diffracting crystals were obtained via co-crystallization of the C-fucosylated derivatives with the bacterial lectin LecB. The sitting drop vapor diffusion method was used, screening 192 different conditions per compound. The lyophilized protein was dissolved in milli-Q water (5 mg/mL) in the presence of salts (6 mM CaCl_2_ and MgCl_2_). The peptides were added to the protein at a 5:1 molar excess related to the LecB lectin monomer. Crystals were obtained within 1–3 months after mixing 1.5 μL of the LecB ligand complex with 1.5 μL of reservoir solution and incubation at 18 °C. All crystallization conditions were found in Index screens I/II (96 conditions) and Crystal Screen I/II (96 conditions) (Hampton Research, Laguna Niguel, CA, USA). Diffraction data were collected at the Paul Scherrer Institute (Villigen, Switzerland) on beamline X06DA PX-III using a DECTRIS PILATUS 2M-F detector and a multi-axis PRIGo goniometer. The structures were solved and visualized with the help of Phenix,^77^ ccp4,^78^ PyMol,^79^ coot,^80^ and XDS.^81^

### MD Simulation

MD simulations were performed using GROMACS^73^ software version 2018.1 and the GROMOS53a6 force field.^82^ The starting topologies were built from PyMOL. A dodecahedral box was created around the peptide 1.0 nm from the edge of the peptide and filled with extended simple point charge water molecules. Sodium and chloride ions were added to produce an electroneutral solution at a final concentration of 0.15 M NaCl. The energy was minimized using a steepest gradient method to remove any close contacts before the system was subjected to a two-phase position-restrained MD equilibration procedure. The system was first allowed to evolve for 100 ps in a canonical NVT (*N* is the number of particles, *V* the system volume, and *T* the temperature) ensemble at 300 K before pressure coupling was switched on, and the system was equilibrated for an additional 100 ps in the NPT (P is the system pressure) ensemble at 1.0 bar.

#### MD in the Presence of DPC Micelle

MD simulations in the presence of a DPC (*n*-DPC) micelle were performed as follows. Parameters and references for the DPC molecule for the GROMOS53a6 forcefield are given in the Supporting Information. Peptides were manually placed at a distance from the pre-equilibrated micelle (of 65 DPC molecules) equal to the diameter of said peptide. Box, solvation, and NVT equilibration procedures were performed as explained previously. For each peptide/micelle system, 10 runs of 50 ns were generated to show the possibility for the peptide to either interact or diffuse away from the micelle. Then, runs of interest were extended up to 250 ns.

#### Clustering of Stable Structures

To obtain a representative conformer for each run, the last 100 ns (10001 frames) were clustered using an RMSD cutoff adapted to get a good balance between the number of clusters and the size of the main cluster. Many clusters combined with a very large percentage of structures in the top cluster is an indication of the stability of the one main conformer in each case. The PyMol Molecular Graphics System, version 1.8 (Schrödinger, LLC), was used to create structural models.

## Supporting Information Available

Peptides yields, MS and analytical data, CD spectra and analysis, vesicle leakage fluorescence curves, time-killing kinetics curves, full serum stability curves, full cytotoxicity data, data collection, refinement statistics, X-ray structures, topology of DPC molecules for MD simulations, MD simulation analyses, additional statistical analysis of **ln65** diastereomers, structures, and HPLC–MS chromatograms and HRMS spectra for all compounds (PDF)
SMILES and activity of all tested peptides (CSV)

## Accession Codes

Coordinates and structure factors of refined LecB complexed with fucosylated peptides are available from the Protein data Bank with accession codes 8AN9 (FHP5), 8ANO (FHP8), 8ANR (FHP30), and 8AOO (FHP31).

## Notes

Raw data of LC–MS analyses for characterization and serum stability, HRMS, CD measurements, vesicle leakage assay, X-ray crystallography, MD simulations, cytotoxicity assay, and pictures of 96-well plates for microbiological and hemolysis assays are accessible at: 10.5281/zenodo.7824946

## Abbreviations

ACN: acetonitrile
AMP: antimicrobial peptides
AMPD: antimicrobial peptide dendrimer
ATCC: American-type culture collection
CD: circular dichroism
CF: 5(6)-carboxyfluorescein
CFU: colony-forming units
ChEMBL: Chemical European Molecular Biology Laboratory Database
DBU: 1,8-diazabicyclo[5.4.0]undec-7-ene
DIC: *N*,*N*-diisoprpylcarbodiimide
DMEM: Dulbecco’s modified Eagle’s medium
DMF: dimethylformamide
DPC: dodecylphosphocholine
EYPC: egg yolk phosphatidyl choline
EYPG: egg yolk phosphatidyl glycerol
HEK cells: human embryonic kidney cells
HEPES: 2-[4-(2-hydroxyethyl)piperazin-1-yl]ethanesulfonic acid
HPLC: high-performance liquid chromatography
hRBC: human red blood cell
HRMS: high resolution mass spectrometry
LB: Luria–Bertani
LCMS: liquid chromatography-mass spectrometry
MD: molecular dynamics
MDR: multidrug-resistant
MIC: minimal inhibitory concentration
MH: Mueller–Hinton
MHC: minimum hemolytic concentration
MRSA: methicillin-resistant *Staphylococcus aureus*
MTT: 3-(4,5-dimethylthiazol-2-yl)-2,5-diphenyltetrazolium bromide
NCTC: national collection of type cultures
Oxyma: ethyl cyanohydroxyiminoacetate
PB: phosphate buffer
PBS: phosphate buffer saline
PCA: principal components analysis
PDB: protein data bank
RMSD: root-mean-square deviation
RP-HPLC: reverse-phase high-performance liquid chromatography
RPMI: Roswell Park memorial institute medium
SDS: sodium dodecyl sulfate
SPPS: solid-phase peptide synthesis
TBME: terbutylmethyl ether
TFA: trifluoroacetic acid
TFE: trifluoroethanol
TIS: triisopropylsilane
TRIS: 2-amino-2-hydroxymethyl-propane-1,3-diol
