# Supplementary material for: To Fold or Not to Fold: Diastereomeric Optimization of an α-Helical Antimicrobial Peptide
Source: J Med Chem. 2023 May 25;66(11):7570–83. doi: 10.1021/acs.jmedchem.3c00460 (PMC10258794; doi:10.1021/acs.jmedchem.3c00460)
Supplement: Supplementary file 1 — jm3c00460_si_001.pdf [file jm3c00460_si_001.pdf]

Supporting information for:

To fold or not to fold: diastereomeric optimization of an α-helical antimicrobial peptide

Hippolyte Personne,[1] Thierry Paschoud,[1] Sofia Fulgencio,[1] Stéphane Baeriswyl,[1] Thilo Köhler,[2] Christian van Delden,[2,3] Achim Stocker,[1] Sacha Javor,[1] and Jean-Louis Reymond*[1]

[1]Department of Chemistry, Biochemistry and Pharmaceutical Sciences, University of Bern, Freiestrasse 3, CH-3012 Bern, Switzerland, e-mail: jean-louis.reymond@unibe.ch

[2]Department of Microbiology and Molecular Medicine, University of Geneva, CH-1211 Geneva, Switzerland

[3]Service of Infectious Diseases, University Hospital of Geneva, CH-1205 Geneva, Switzerland

Table of Contents

1. Peptide synthesis 3
Table S1 3
2. Circular dichroism spectroscopy 5
Figure S1 11
Table S2 12
3. Vesicle leakage assay 14
Figure S2 25
4. Time kill kinetics assay 26
Figure S3 26
5. Serum stability assay 27
Figure S4 27
6. Cytotoxicity assay 28
Figure S5 28
Figure S6 29
7. Crystallography 30
Table S3 30
Figure S7 31
Table S4 32
Figure S8 33
Table S5 34
Figure S9 35
Table S6 36

Figure S10 37
8. Molecular dynamics 38
Figure S11 40
Figure S12 41
Figure S13 42
Figure S14 43
Figure S15 44
Figure S16 45
Figure S17 46
Figure S18 47
Figure S19 48
Figure S20 49
Figure S21 50
9. Statistical Analysis 51
Figure S22 51
Figure S23 52
10. HPLC-MS and HRMS data 53
11. References 169

1. Peptide synthesis

Table S1 (pages S3 and S4): Synthesis of linear AMPs.

Cpd.	Sequence[a)]	SPPS yield[b)] mg (%)	MS analysis[c)] calc./obs.	Analytical HPLC[d)] t_R (min)
**ln65**	KKLLKLLKLLL	84.0 (58.0)	1320.99/1320.99	1.66
**dln65**	kkllkllklll	34.9 (24.1)	1320.99/1322.00	1.62
**ln69**	kkLLkLLkLLL	102.6 (70.9)	1320.99/1320.99	1.55
**dln69**	KKllKllKlll	69.7 (44.6)	1320.99/1322.00	1.52
***sr*-ln65**	<u>KKLLKLLKLLL</u>	64.6 (41.3)	1320.99/1321.00	1.58
***sr*-ln65L^{6}**	<u>KKLLK</u>L<u>LKLLL</u>	77.3 (49.4)	1320.99/1322.00	1.56
**HP1**	KkLLKLLKLLL	53.0 (47.7)	1320.99/1321.00	1.67
**HP2**	kkLLKLLKLLL	56.7 (51.0)	1320.99/1320.99	1.66
**HP3**	KkLLkLLKLLL	50.1 (45.1)	1320.99/1322.00	1.61
**HP4**	KkLlKLLKLLL	42.6 (38.3)	1320.99/1322.00	1.74
**HP5**	kKLLKLLKLLl	49.1 (44.2)	1320.99/1322.00	1.65
**HP6**	KKLLKllKLLL	49.3 (44.4)	1320.99/1322.00	1.53
**HP7**	kkLLKLLKLLl	60.6 (54.5)	1320.99/1322.00	1.62
**HP8**	KkllKLLKLLL	46.9 (42.2)	1320.99/1322.00	1.63
**HP9**	KKLLkllKLLL	63.9 (57.5)	1320.99/1322.00	1.52
**HP10**	kkLLkLLKLLL	50.0 (45.0)	1320.99/1322.00	1.60
**HP11**	KkllKLlKLLL	31.5 (35.4)	1320.99/1322.00	1.60
**HP12**	KkllKlLKLLL	26.8 (30.1)	1320.99/1322.00	1.60
**HP13**	KKLLkllkLLL	37.4 (42.1)	1320.99/1322.00	1.53
**HP14**	KKllKllKLLL	33.7 (37.9)	1320.99/1322.00	1.53
**HP15**	KKlLkLlKlLL	34.5 (38.8)	1320.99/1322.00	1.60
**HP16**	KKLlkLLklLL	16.0 (18.0)	1320.99/1322.00	1.59
**HP17**	KklLKLLKllL	29.0 (26.1)	1320.99/1322.00	1.61
**HP18**	kkLLKLLKLll	36.3 (32.7)	1320.99/1320.99	1.58
**HP19**	kkLLkLLKLLl	53.0 (47.7)	1320.99/1322.00	1.56
**HP20**	KKllKLLklLL	51.4 (46.3)	1320.99/1322.00	1.52
**HP21**	KklLKlLKlLL	64.2 (65.7)	1320.99/1322.00	1.60
**HP22**	KKllKLlKLlL	45.9 (46.9)	1320.99/1322.00	1.55
**HP23**	KKLlkLLkLLl	64.7 (66.2)	1320.99/1322.00	1.56
**HP24**	KkllKllKLLL	54.7 (49.2)	1320.99/1322.00	1.56
**HP25**	KKllKllKlLL	38.8 (34.9)	1320.99/1322.00	1.55
**HP26**	kkLLkLLKLll	52.6 (47.3)	1320.99/1322.00	1.57
**HP27**	kkLLkLLkLLl	53.7 (48.3)	1320.99/1322.00	1.54
**HP28**	kKLLkllKLLl	45.6 (41.0)	1320.99/1322.00	1.55
**HP29**	KKLlkllkLLL	59.3 (53.4)	1320.99/1322.00	1.58
**HP30**	KkLlKlLkLlL	35.7 (32.1)	1320.99/1322.00	1.57
**HP31**	kKlLkLlKlLl	45.3 (40.8)	1320.99/1322.00	1.57
**HP32**	RRLLRLLRLLL	32.5 (27.5)	1433.02/1434.03	1.64
**HP33**	rrLLrLLrLLL	44.3 (37.5)	1433.02/1434.03	1.52
**HP34**	KKIIKIIKIII	35.1 (31.6)	1320.99/1322.00	1.44
**HP35**	kkIIkIIkIII	29.3 (26.4)	1320.99/1322.00	1.36
**HP36**	RRIIRIIRIII	7.7 (6.5)	1433.02/1434.03	1.47
**HP37**	rrIIrIIrIII	38.7 (32.8)	1433.02/1434.03	1.68
**2ln65**	$(KKLLKLLKLLL)_2$	119.3 (38.3)	2624.96/2625.67	2.38
**2ln69**	$(kkLLkLLkLLL)_2$	85.8 (27.6)	2624.96/2625.97	1.85
**FHP1**	(*)KkLLKLLKLLL	2.9 (3.9)	1509.06/1511.07	1.76
**FHP2**	(*)kkLLKLLKLLL	0.6 (0.8)	1509.06/1510.07	1.73
**FHP3**	(*)KkLLkLLKLLL	4.5 (6.1)	1509.06/1510.07	1.67
**FHP4**	(*)KkLlKLLKLLL	4.5 (6.1)	1509.06/1510.07	1.74
**FHP5**	(*)kKLLKLLKLLl	4.3 (5.8)	1509.06/1510.07	1.66
**FHP7**	(*)kkLLKLLKLLl	3.4 (4.6)	1509.06/1510.07	1.63
**FHP8**	(*)KkllKLLKLLL	5.7 (7.7)	1509.06/1510.06	1.63
**FHP10**	(*)kkLLkLLKLLL	4.0 (5.4)	1509.06/1510.07	1.67
**FHP11**	(*)KkllKLlKLLL	16.9 (17.2)	1509.06/1510.07	1.63

**FHP30**	(*)KkLlKlLkLlL	9.0 (12.2)	1509.06/1510.06	1.62
**FHP31**	(*)kKlLkLlKlLl	5.7 (7.7)	1509.06/1510.07	1.62
**FHP32**	(*)RRLLRLLRLLL	5.7 (7.3)	1621.09/1621.09	1.79
**FHP33**	(*)rrLLrLLrLLL	2.5 (3.2)	1621.09/1621.09	1.62

a) One letter code for amino acid. D- amino acids in lower case. (*) = α-L-fucosyl acetyl moiety. b) Yields given for RP-HPLC purified products. c) High-resolution electrospray ionization mass spectrometry (positive mode), the calculated monoisotopic mass and the observed mass are given. d) retention time in analytical RP-HPLC (A/D = 100/0 to 0/100 in 3.5 min, λ = 214 nm).

2. Circular dichroism spectroscopy

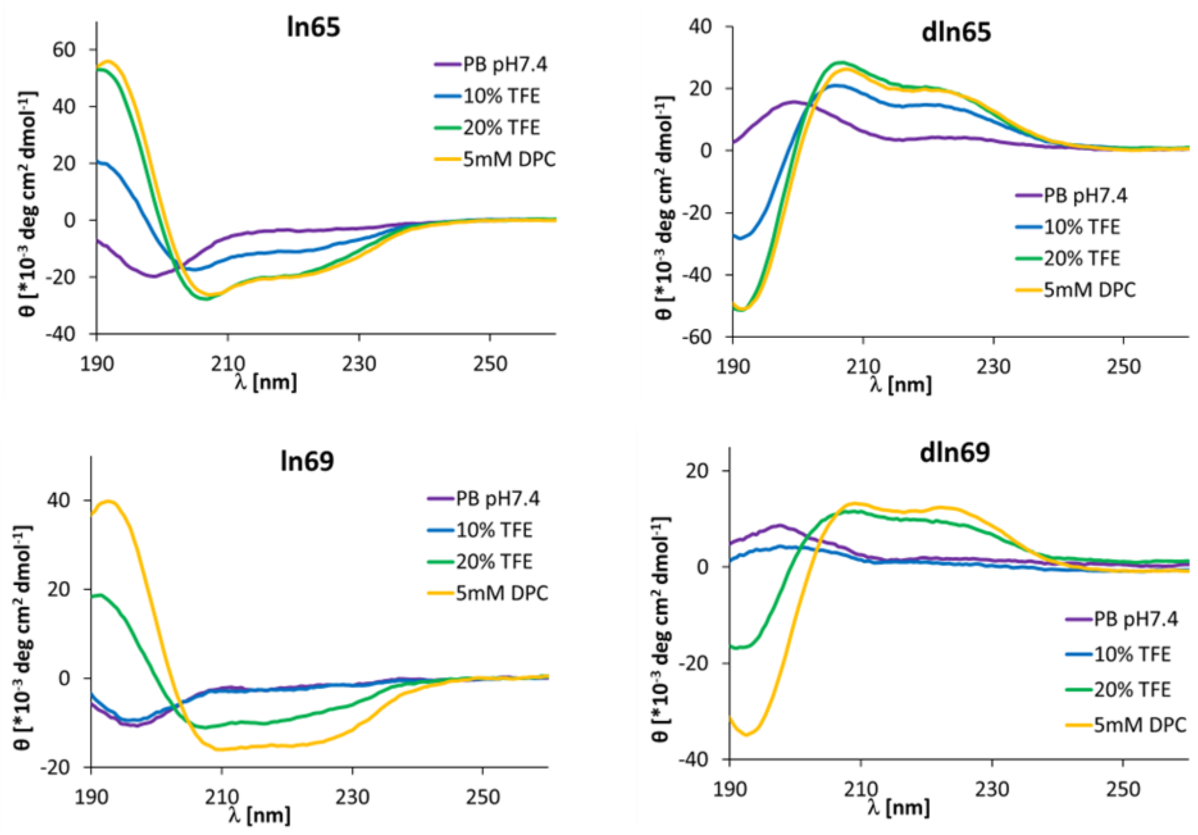

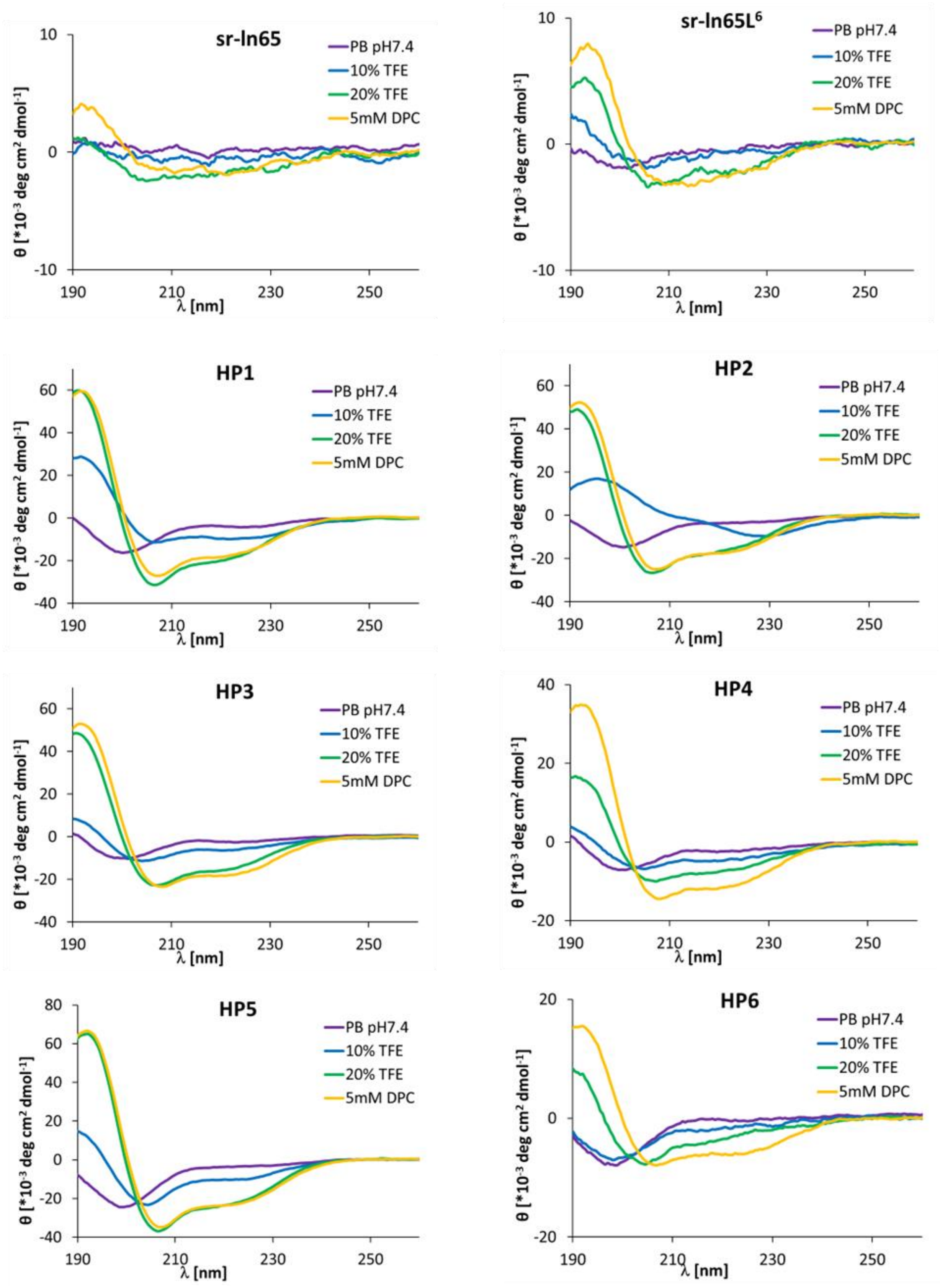
sr-ln65
sr-ln65L6
HP1
HP2
HP3
HP4
HP5
HP6
PB pH7.4
10% TFE
20% TFE
5mM DPC
θ [*10-3 deg cm2 dmol-1]
λ [nm]
190
210
230
250

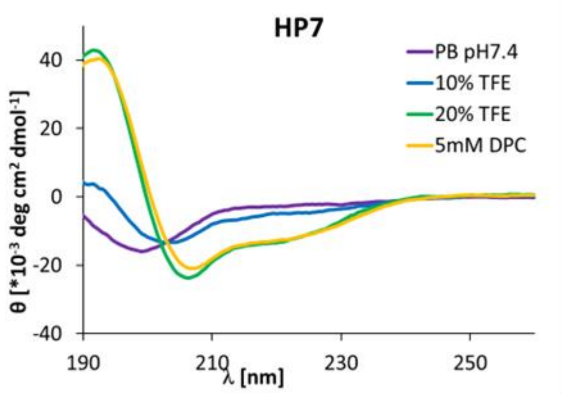
HP7
PB pH7.4
10% TFE
20% TFE
5mM DPC
θ [*10⁻³ deg cm² dmol⁻¹]
λ [nm]
40
20
0
-20
-40
190
210
230
250

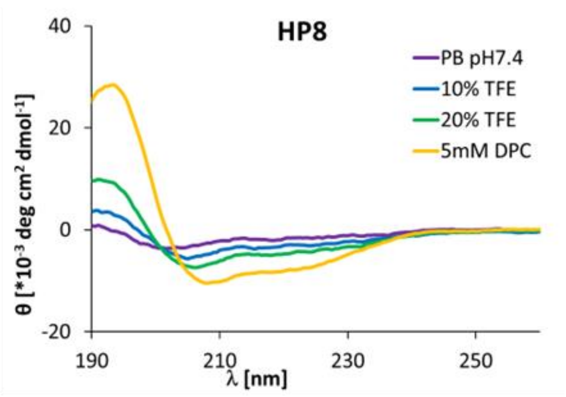
HP8
PB pH7.4
10% TFE
20% TFE
5mM DPC
θ [*10⁻³ deg cm² dmol⁻¹]
λ [nm]
40
20
0
-20
190
210
230
250

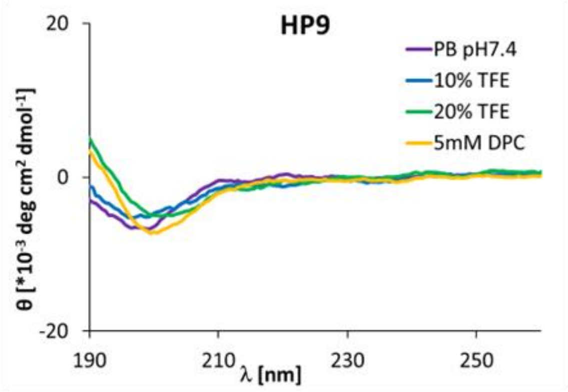
HP9
PB pH7.4
10% TFE
20% TFE
5mM DPC
θ [*10⁻³ deg cm² dmol⁻¹]
λ [nm]
20
0
-20
190
210
230
250

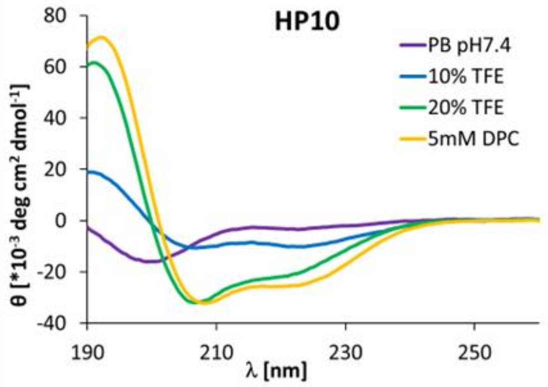
HP10
PB pH7.4
10% TFE
20% TFE
5mM DPC
θ [*10⁻³ deg cm² dmol⁻¹]
λ [nm]
80
60
40
20
0
-20
-40
190
210
230
250

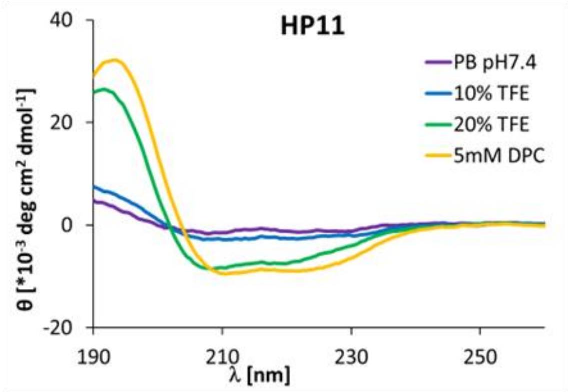
HP11
PB pH7.4
10% TFE
20% TFE
5mM DPC
θ [*10⁻³ deg cm² dmol⁻¹]
λ [nm]
40
20
0
-20
190
210
230
250

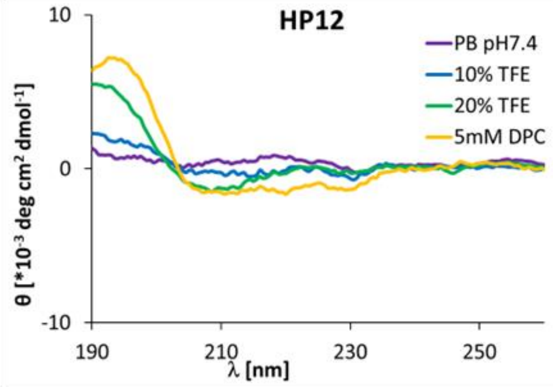
HP12
PB pH7.4
10% TFE
20% TFE
5mM DPC
θ [*10⁻³ deg cm² dmol⁻¹]
λ [nm]
10
0
-10
190
210
230
250

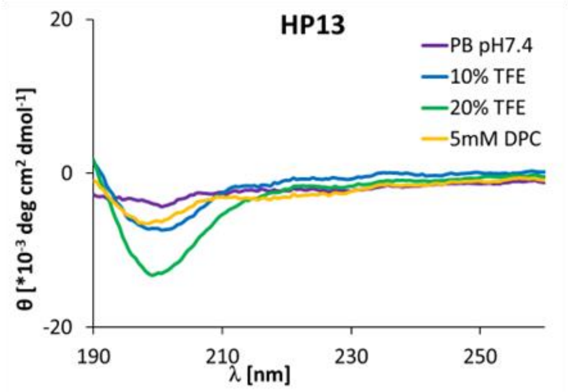
HP13
PB pH7.4
10% TFE
20% TFE
5mM DPC
θ [*10⁻³ deg cm² dmol⁻¹]
λ [nm]
20
0
-20
190
210
230
250

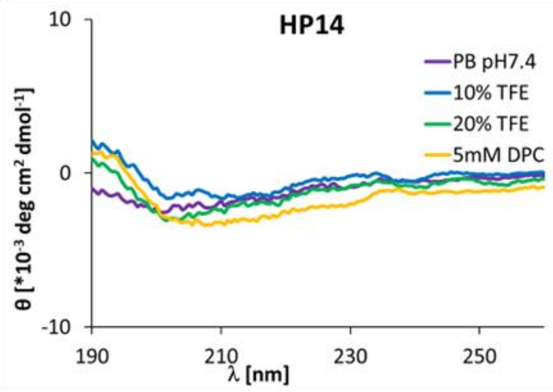
HP14
PB pH7.4
10% TFE
20% TFE
5mM DPC
θ [*10⁻³ deg cm² dmol⁻¹]
λ [nm]
10
0
-10
190
210
230
250

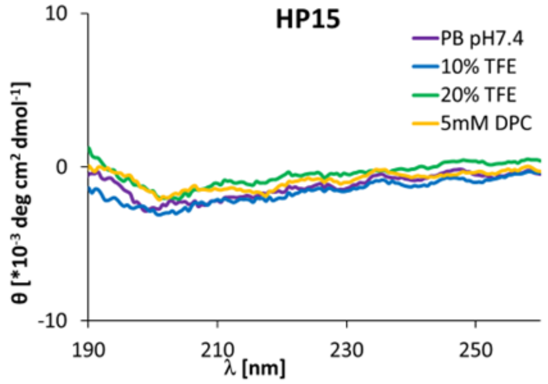
HP15
PB pH7.4
10% TFE
20% TFE
5mM DPC
θ [*10⁻³ deg cm² dmol⁻¹]
λ [nm]
10
0
-10
190
210
230
250

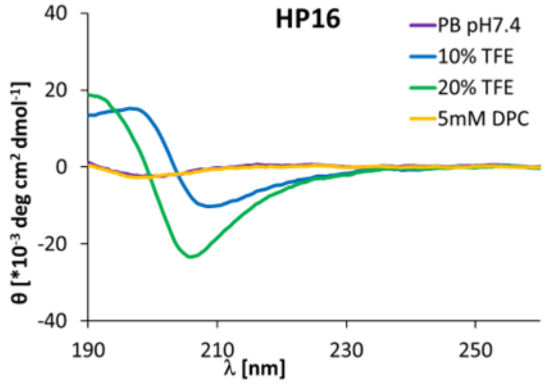
HP16
PB pH7.4
10% TFE
20% TFE
5mM DPC
θ [*10⁻³ deg cm² dmol⁻¹]
λ [nm]
40
20
0
-20
-40
190
210
230
250

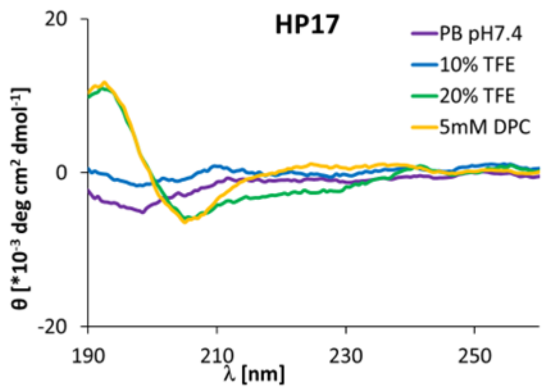
HP17
PB pH7.4
10% TFE
20% TFE
5mM DPC
θ [*10⁻³ deg cm² dmol⁻¹]
λ [nm]
20
0
-20
190
210
230
250

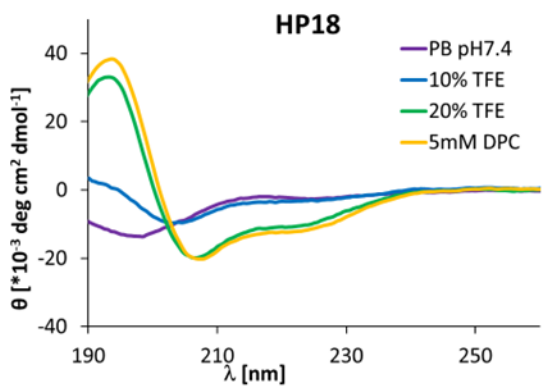
HP18
PB pH7.4
10% TFE
20% TFE
5mM DPC
θ [*10⁻³ deg cm² dmol⁻¹]
λ [nm]
40
20
0
-20
-40
190
210
230
250

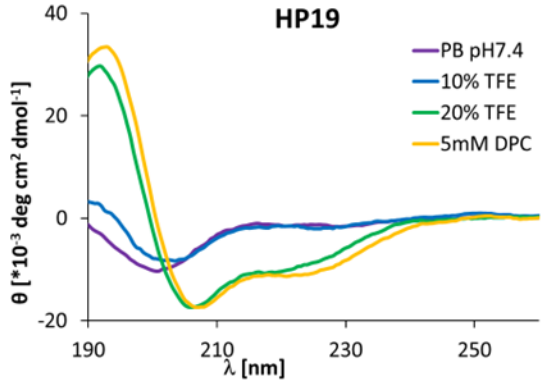
HP19
PB pH7.4
10% TFE
20% TFE
5mM DPC
θ [*10⁻³ deg cm² dmol⁻¹]
λ [nm]
40
20
0
-20
190
210
230
250

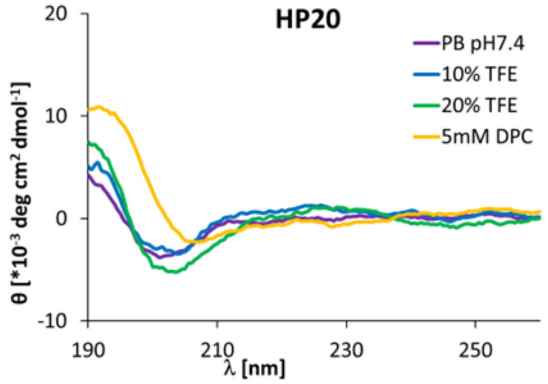
HP20
PB pH7.4
10% TFE
20% TFE
5mM DPC
θ [*10⁻³ deg cm² dmol⁻¹]
λ [nm]
20
10
0
-10
190
210
230
250

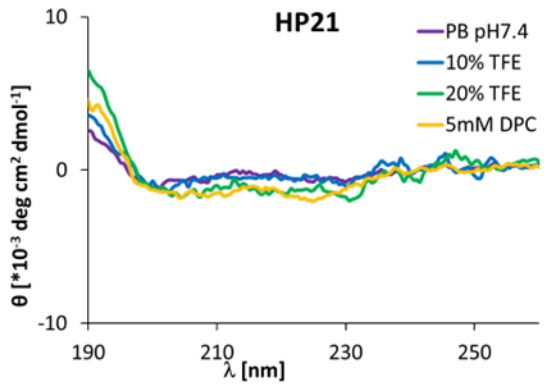
HP21
PB pH7.4
10% TFE
20% TFE
5mM DPC
θ [*10⁻³ deg cm² dmol⁻¹]
λ [nm]
10
0
-10
190
210
230
250

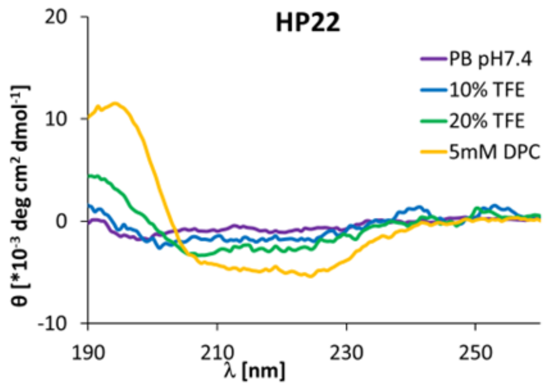
HP22
PB pH7.4
10% TFE
20% TFE
5mM DPC
θ [*10⁻³ deg cm² dmol⁻¹]
λ [nm]
20
10
0
-10
190
210
230
250

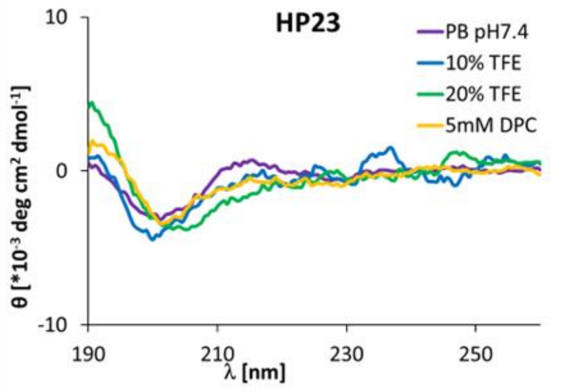
HP23
PB pH7.4
10% TFE
20% TFE
5mM DPC
θ [*10^-3 deg cm^2 dmol^-1]
10
0
-10
190
210
230
250
λ [nm]

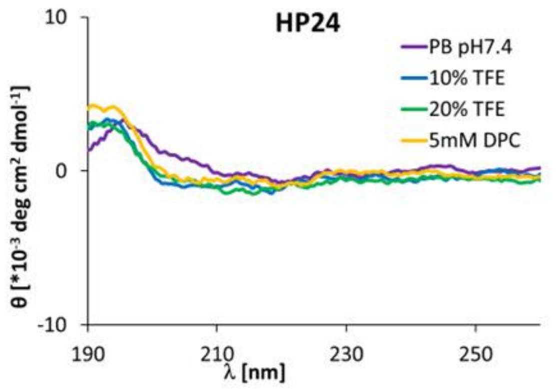
HP24
PB pH7.4
10% TFE
20% TFE
5mM DPC
θ [*10^-3 deg cm^2 dmol^-1]
10
0
-10
190
210
230
250
λ [nm]

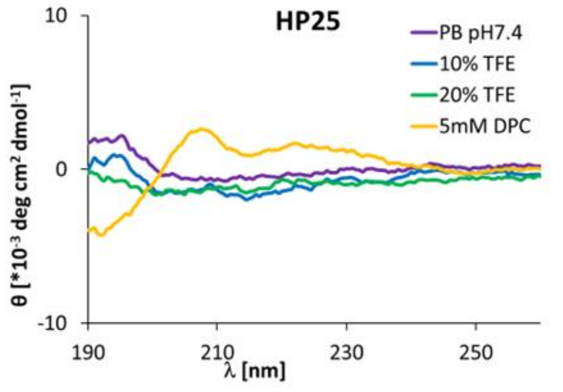
HP25
PB pH7.4
10% TFE
20% TFE
5mM DPC
θ [*10^-3 deg cm^2 dmol^-1]
10
0
-10
190
210
230
250
λ [nm]

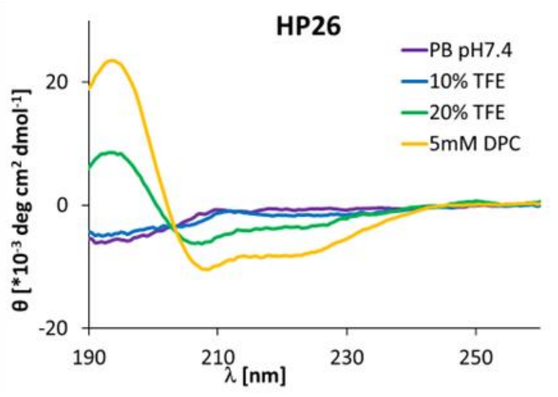
HP26
PB pH7.4
10% TFE
20% TFE
5mM DPC
θ [*10^-3 deg cm^2 dmol^-1]
20
0
-20
190
210
230
250
λ [nm]

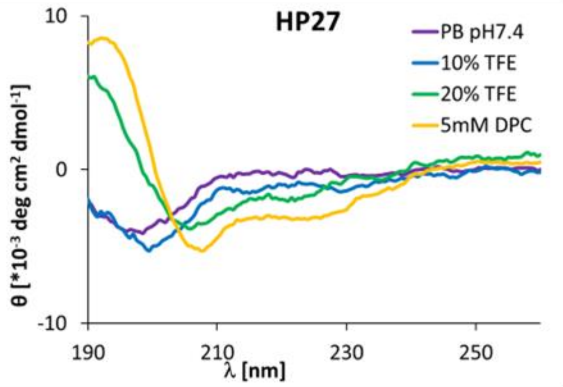
HP27
PB pH7.4
10% TFE
20% TFE
5mM DPC
θ [*10^-3 deg cm^2 dmol^-1]
10
0
-10
190
210
230
250
λ [nm]

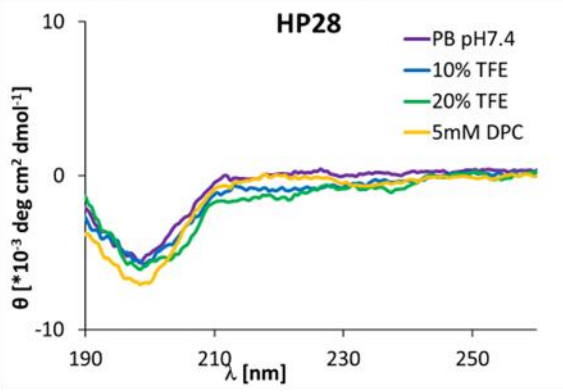
HP28
PB pH7.4
10% TFE
20% TFE
5mM DPC
θ [*10^-3 deg cm^2 dmol^-1]
10
0
-10
190
210
230
250
λ [nm]

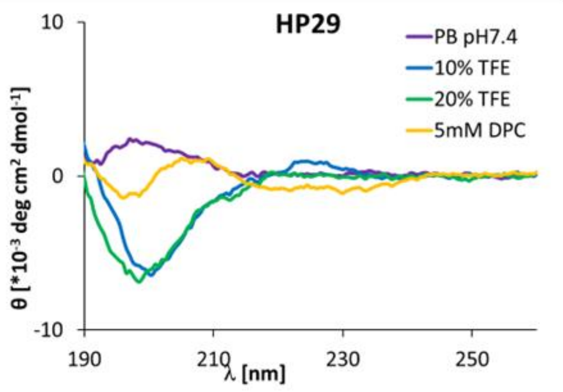
HP29
PB pH7.4
10% TFE
20% TFE
5mM DPC
θ [*10^-3 deg cm^2 dmol^-1]
10
0
-10
190
210
230
250
λ [nm]

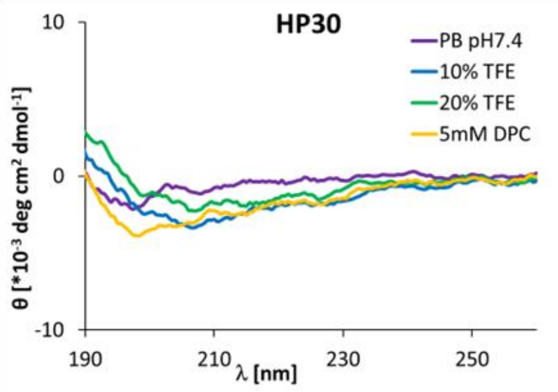
HP30
PB pH7.4
10% TFE
20% TFE
5mM DPC
θ [*10^-3 deg cm^2 dmol^-1]
10
0
-10
190
210
230
250
λ [nm]

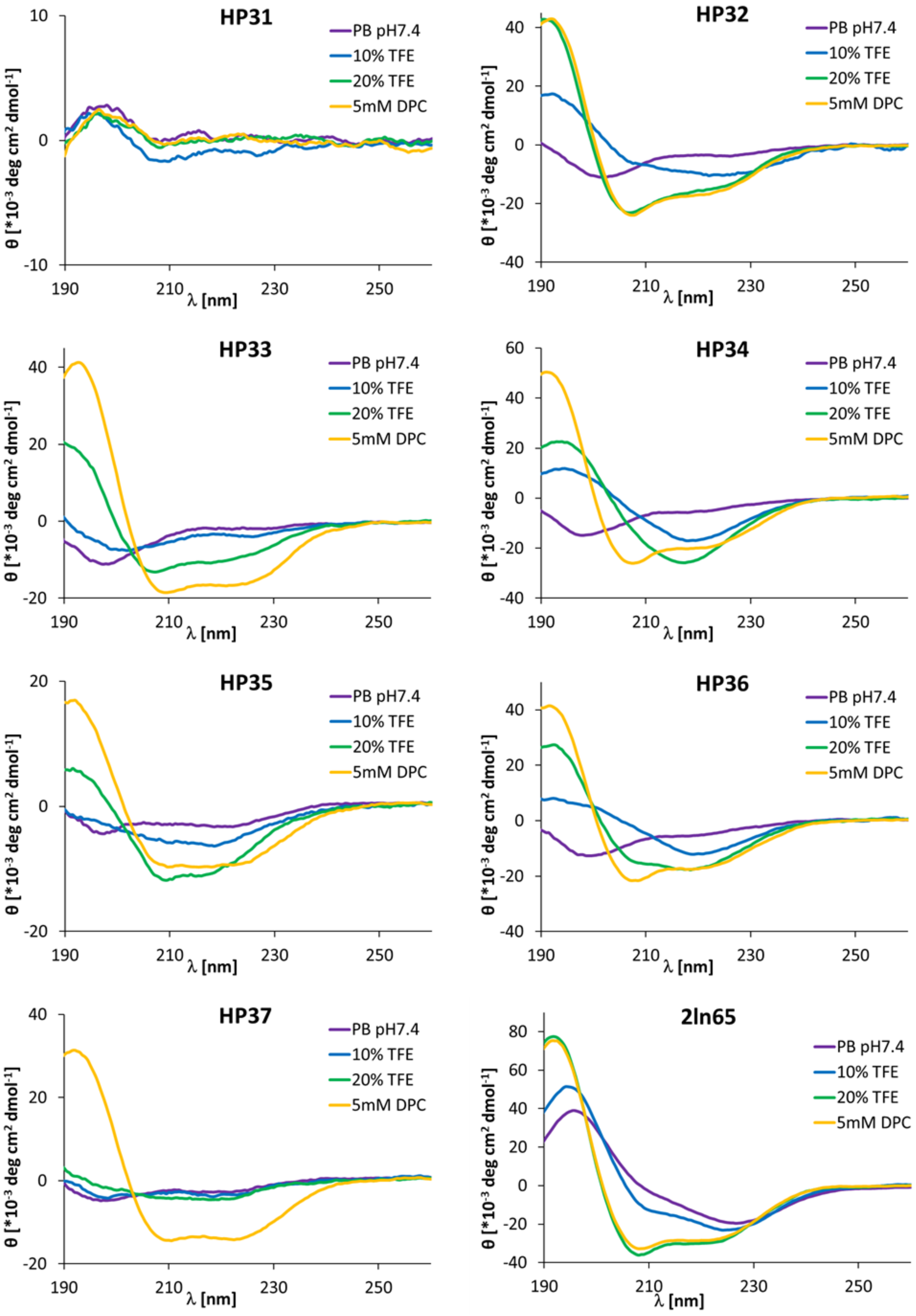
HP31
HP32
HP33
HP34
HP35
HP36
HP37
2ln65
PB pH7.4
10% TFE
20% TFE
5mM DPC
θ [*10⁻³ deg cm² dmol⁻¹]
λ [nm]
190
210
230
250

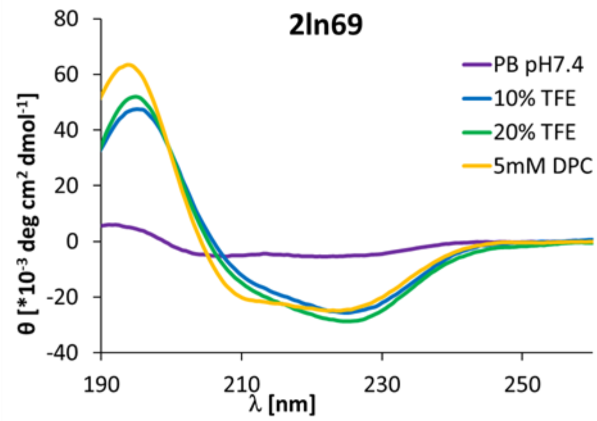

Figure S1: (from page S5 to S11) Circular dichroism spectra of linear peptides at 0.100 mg/mL in 7 mM phosphate buffer pH 7.4 with different amount of TFE and 5 mM DPC.

Table S1: (from page S12 to page S13) Dichroweb analysis of linear peptides.

Cpd.	Sequence [a)]	CD α/β/t/u (%) [b)]			
		PB Buffer	10% TFE	20% TFE	5 mM DPC
sr-**ln65**	KKLLKLLKLLL	5/42/21/32	7/39/22/32	10/37/22/31	10/36/23/31
sr-**ln65L**[6]	KKLLKLLKLLL	9/35/24/32	11/36/23/31	17/30/22/31	17/32/22/29
**ln65**	KKLLKLLKLLL	14/24/27/35	37/12/24/27	64/3/17/16	73/2/15/10
**dln65**	kkllkllklll	11/18/24/37	45/9/20/26	61/3/16/20	67/2/14/17
**ln69**	kkLLkLLkLLL	11/30/24/35	10/30/25/35	34/20/20/26	61/6/16/17
**dln69**	KKllKllKlll	8/34/23/35	7/36/22/35	28/23/20/29	59/13/15/23
**HP1**	KkLLKLLKLLL	18/22/24/36	43/18/20/19	69/3/16/12	73/2/14/11
**HP2**	kkLLKLLKLLL	16/23/27/34	35/24/21/20	60/3/17/20	69/2/15/14
**HP3**	KkLLkLLKLLL	13/30/23/34	24/22/25/29	56/8/16/20	69/3/15/13
**HP4**	KkLkLLKLLL	11/33/23/33	15/31/24/30	27/25/21/27	46/17/16/21
**HP5**	kKLLKLLKLLl	16/19/28/37	40/7/23/30	84/0/12/4	90/0/10/0
**HP6**	KKLLKllKLLL	9/32/24/35	9/22/24/34	16/30/24/30	29/23/22/26
**HP7**	kkLLKLLKLLl	14/25/27/34	23/21/25/31	59/2/22/17	60/6/17/17
**HP8**	KkllKLLKLLL	9/36/23/32	16/33/23/28	22/28/23/27	37/22/19/22
**HP9**	KKLLkllKLLL	8/34/24/34	9/34/24/33	9/35/22/34	10/33/25/32
**HP10**	kkLLkLLKLLL	12/26/26/36	35/19/21/25	72/2/13/13	90/1/9/0
**HP11**	KkllKLlKLLL	14/32/22/32	18/31/21/30	38/22/18/22	52/15/14/18
**HP12**	KkllKlLKLLL	8/37/23/32	10/37/22/31	12/36/22/30	17/34/21/28
**HP13**	KKLLkllkLLL	11/31/26/32	6/37/22/35	11/30/24/35	9/34/24/33
**HP14**	KKllKllKLLL	7/37/23/33	5/40/23/32	8/37/24/31	13/33/23/31
**HP15**	KKlLkLlKlLL	9/35/23/33	8/36/25/31	6/41/21/32	6/39/23/32
**HP16**	KKLlkLLklLL	8/36/24/32	39/23/16/22	56/5/20/19	8/31/19/22
**HP17**	KklLKLLKllL	8/35/24/33	7/38/22/33	17/31/22/30	16/33/22/29
**HP18**	kkLLKLLKLll	13/24/27/36	18/26/23/33	56/4/21/19	63/3/17/17
**HP19**	kkLLkLLKLLl	13/29/25/33	16/28/24/32	44/13/21/22	55/6/20/19
**HP20**	KKllKLLklLL	7/38/23/32	8/38/23/31	9/35/25/31	15/37/20/28

**HP21**	KklLKlLKlLL	8/37/23/32	8/38/22/32	11/37/22/30	11/35/22/32
**HP22**	KKllKLlKLlL	8/37/23/32	8/36/22/34	13/34/22/31	23/30/19/28
**HP23**	KKLlkLLkLLl	10/35/23/32	8/36/23/33	11/36/23/30	10/35/24/31
**HP24**	KkllKllKLLL	7/39/22/32	7/39/23/31	8/38/23/31	7/39/22/32
**HP25**	KKllKllKlLL	6/39/22/33	5/40/21/34	5/40/21/34	12/34/22/32
**HP26**	kkLLkLLKLll	9/33/24/34	12/33/24/31	24/26/22/28	41/18/19/22
**HP27**	kkLLkLLkLLl	9/34/24/33	10/33/24/33	14/32/22/32	23/27/21/29
**HP28**	kKLLkllKLLl	8/35/23/34	10/32/24/34	10/33/25/32	10/33/24/33
**HP29**	KKLlkllkLLL	6/39/22/33	7/36/24/33	7/35/23/35	7/38/22/33
**HP30**	KkLlKlLkLlL	6/38/22/34	10/35/23/32	7/38/23/32	7/37/23/33
**HP31**	kKlLkLlKlLl	5/38/22/35	4/40/21/35	6/39/21/34	5/39/20/36
**HP32**	RRLLRLLRLLL	16/27/24/33	32/25/20/23	56/5/17/22	62/3/16/19
**HP33**	rrLLrLLrLLL	10/30/25/35	14/29/23/34	32/21/19/28	63/5/16/16
**HP34**	KKIIKIIKIII	12/26/27/35	16/32/20/32	21/29/20/30	68/3/15/14
**HP35**	kkIIkIIkIII	10/32/23/35	12/30/24/34	22/23/23/32	22/22/19/27
**HP36**	RRIIRIIRIII	12/27/26/35	14/33/20/33	39/16/18/27	60/4/18/18
**HP37**	rrIIrIIrIII	7/34/23/36	10/33/23/34	11/32/23/34	50/11/16/23
**2ln65**	$(KKLLKLLKLLL)_2$	58/14/18/10	68/3/9/10	94/1/5/0	91/1/7/1
**2ln69**	$(kkLLkLLkLLL)_2$	20/28/21/31	73/5/11/11	80/5/13/2	82/3/9/6

[a] One letter code for amino acids. D- amino acids are in lower case and stereorandomized residues are underlined.
[b] CD spectra were recorded at 0.100 mg/mL in aqueous 7 mM phosphate buffer pH 7.4 with addition of 0, 10 and 20% TFE or 5 mM DPC. The primary CD spectra were analyzed using Dichroweb and the percentages of α-helical (α), β-sheet (β), turns (t) and unordered (u) signals were extracted. The Contin-LL method and reference set 4 were used.[1]

3. Vesicle leakage assay

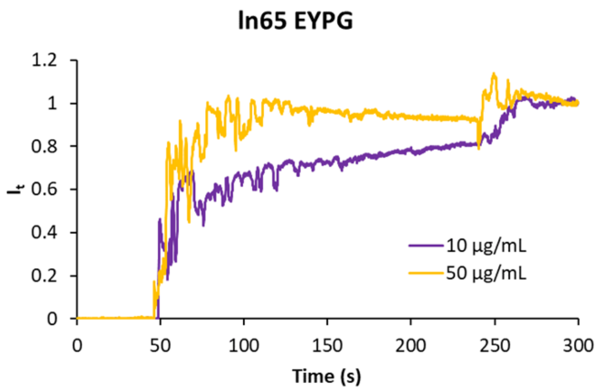

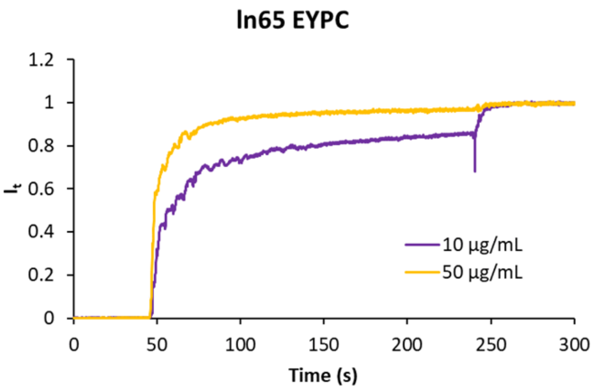

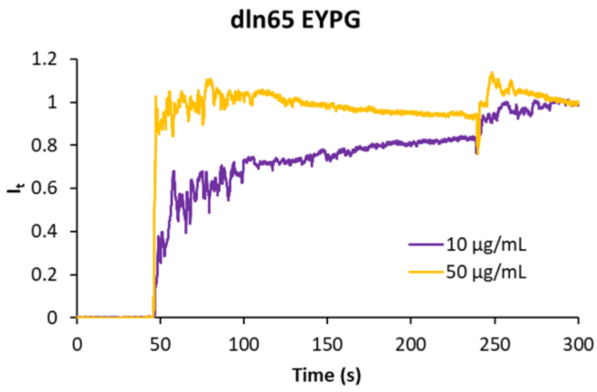

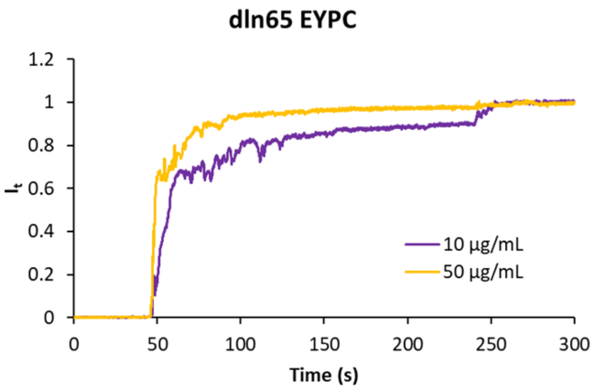

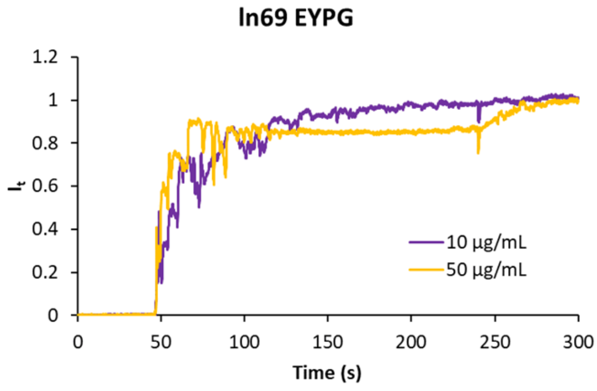

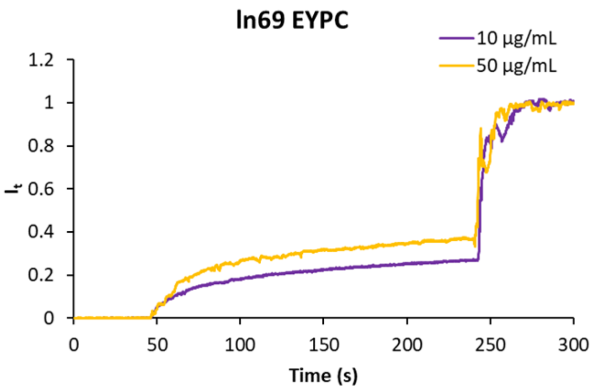

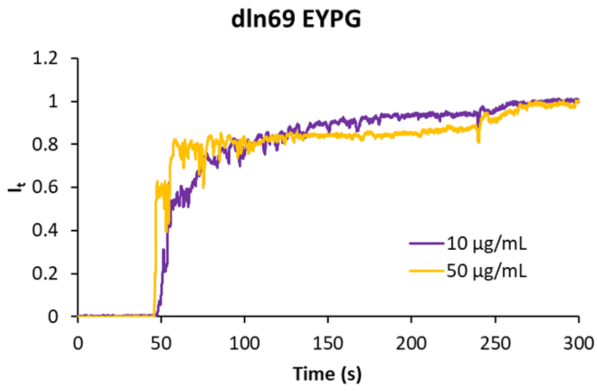

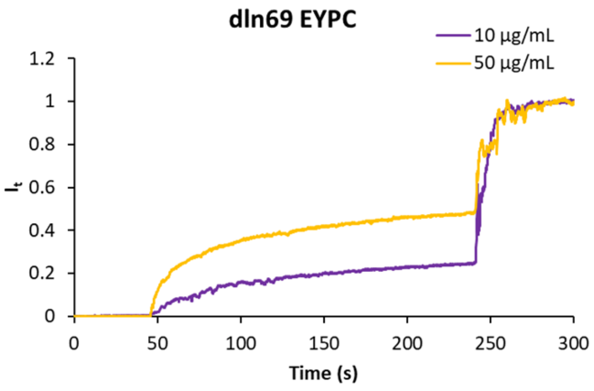

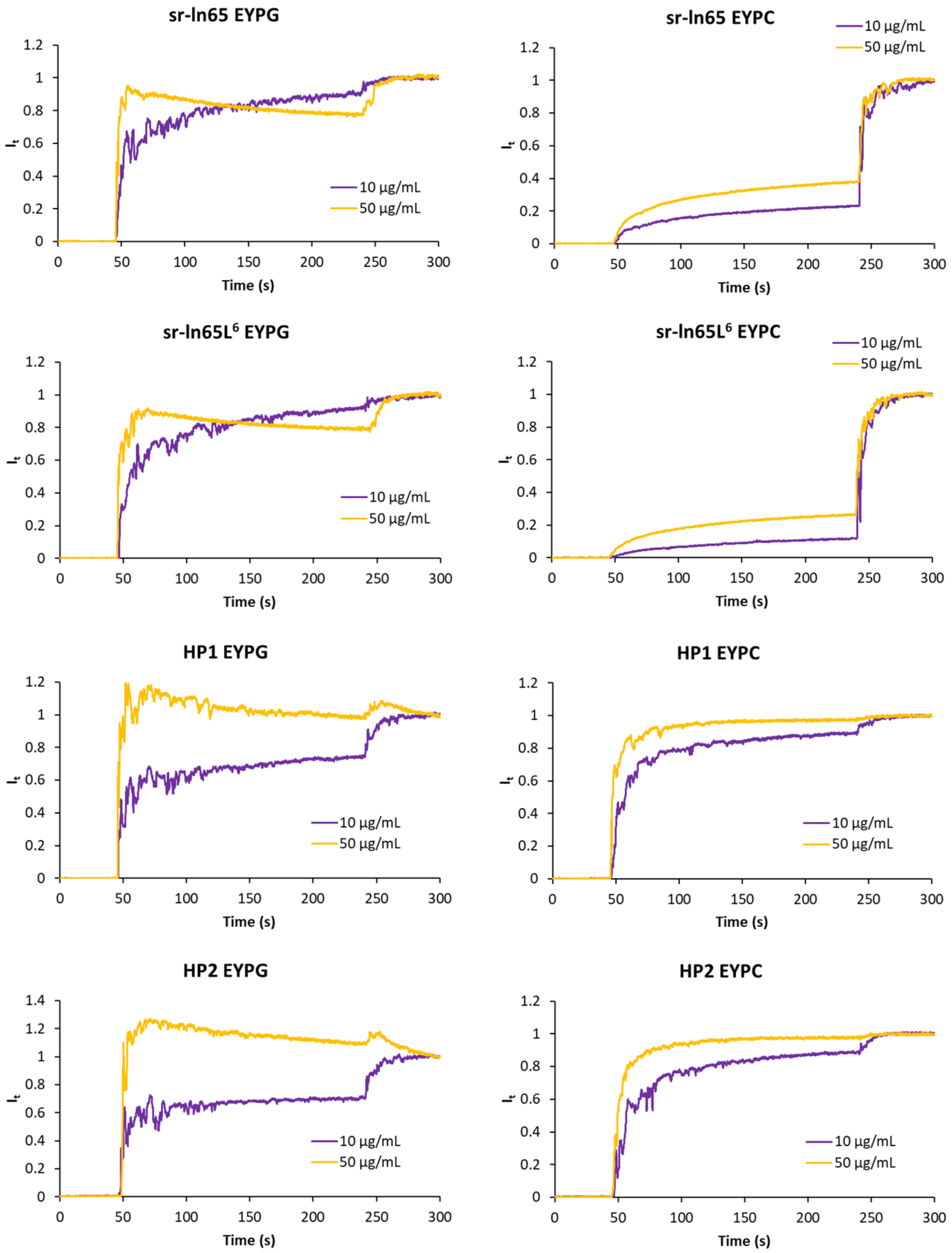
sr-In65 EYPG
sr-In65 EYPC
sr-In65L6 EYPG
sr-In65L6 EYPC
HP1 EYPG
HP1 EYPC
HP2 EYPG
HP2 EYPC
10 µg/mL
50 µg/mL
It
Time (s)

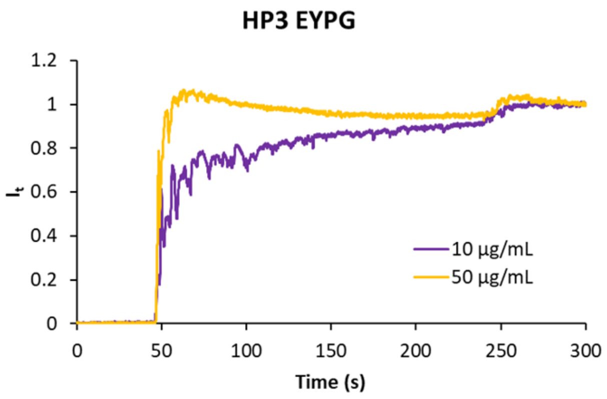
HP3 EYPG
1.2
1
0.8
0.6
0.4
0.2
0
It
0
50
100
150
200
250
300
Time (s)
10 μg/mL
50 μg/mL

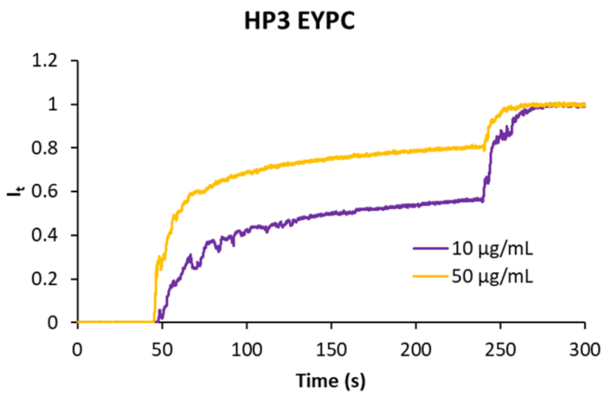
HP3 EYPC
1.2
1
0.8
0.6
0.4
0.2
0
It
0
50
100
150
200
250
300
Time (s)
10 μg/mL
50 μg/mL

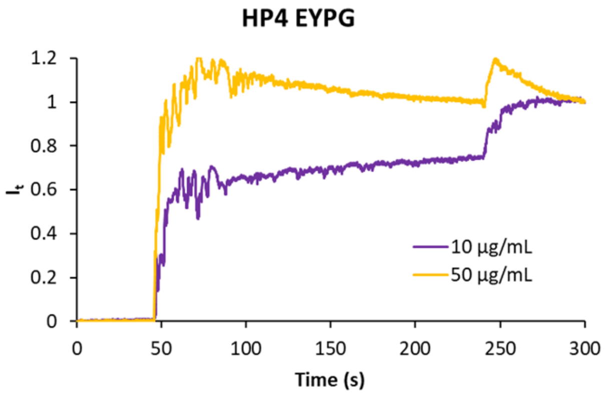
HP4 EYPG
1.2
1
0.8
0.6
0.4
0.2
0
It
0
50
100
150
200
250
300
Time (s)
10 μg/mL
50 μg/mL

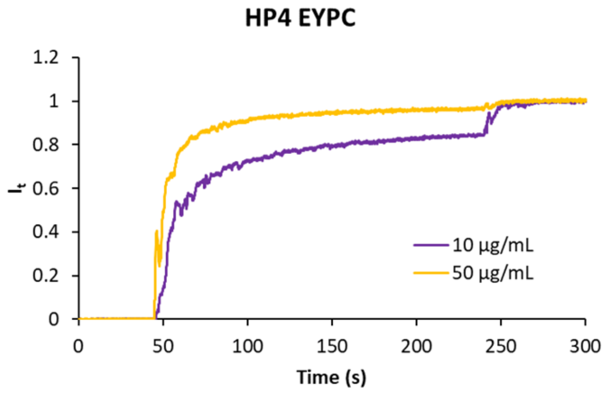
HP4 EYPC
1.2
1
0.8
0.6
0.4
0.2
0
It
0
50
100
150
200
250
300
Time (s)
10 μg/mL
50 μg/mL

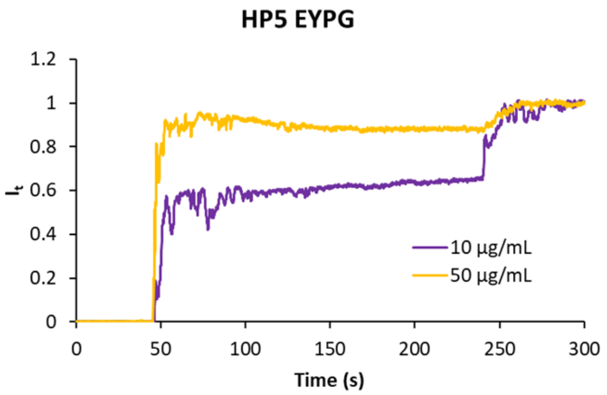
HP5 EYPG
1.2
1
0.8
0.6
0.4
0.2
0
It
0
50
100
150
200
250
300
Time (s)
10 μg/mL
50 μg/mL

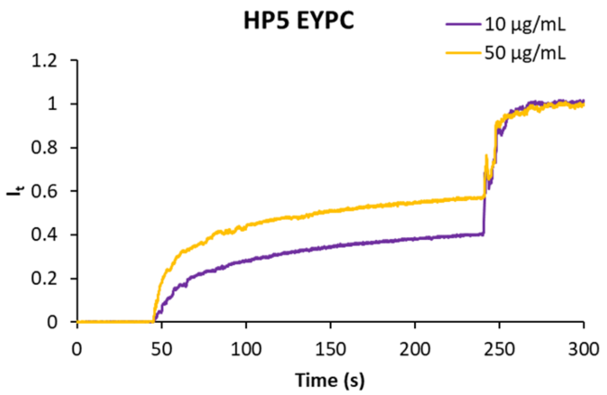
HP5 EYPC
10 μg/mL
50 μg/mL
1.2
1
0.8
0.6
0.4
0.2
0
It
0
50
100
150
200
250
300
Time (s)

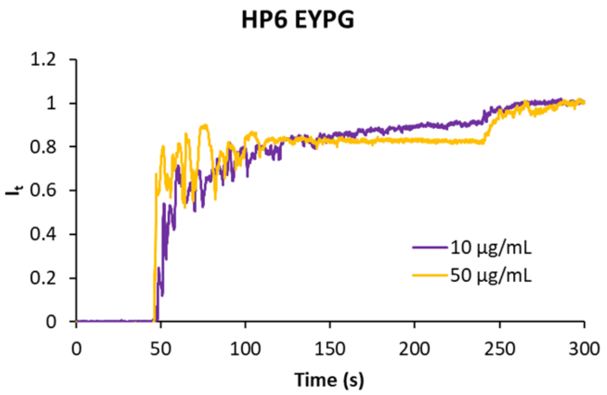
HP6 EYPG
1.2
1
0.8
0.6
0.4
0.2
0
It
0
50
100
150
200
250
300
Time (s)
10 μg/mL
50 μg/mL

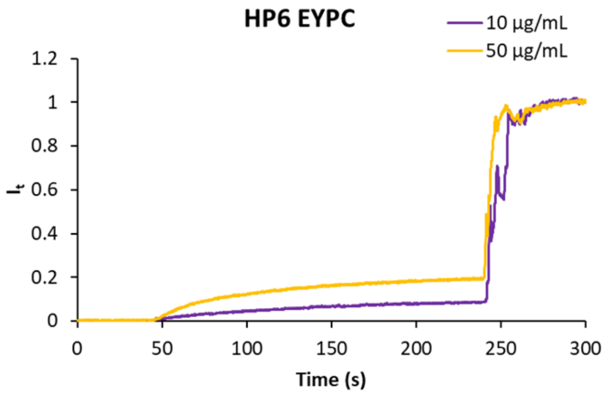
HP6 EYPC
10 μg/mL
50 μg/mL
1.2
1
0.8
0.6
0.4
0.2
0
It
0
50
100
150
200
250
300
Time (s)

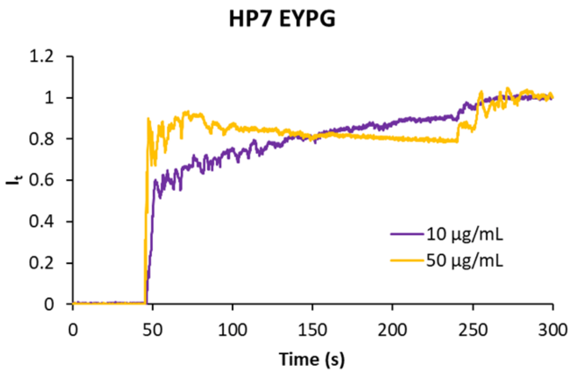
HP7 EYPG
I_t
Time (s)
10 µg/mL
50 µg/mL

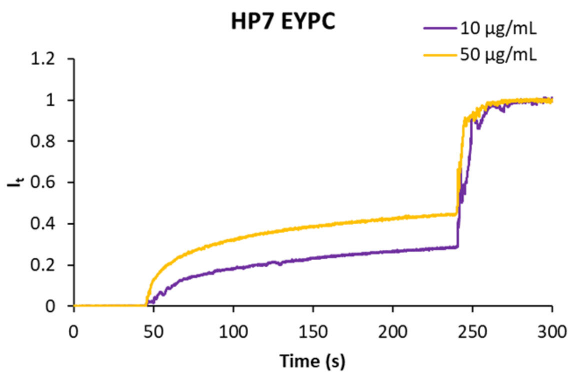
HP7 EYPC
I_t
Time (s)
10 µg/mL
50 µg/mL

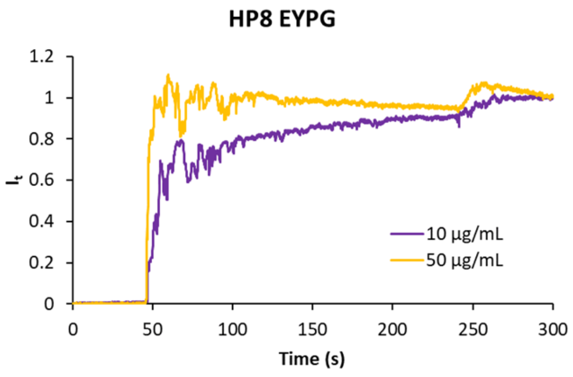
HP8 EYPG
I_t
Time (s)
10 µg/mL
50 µg/mL

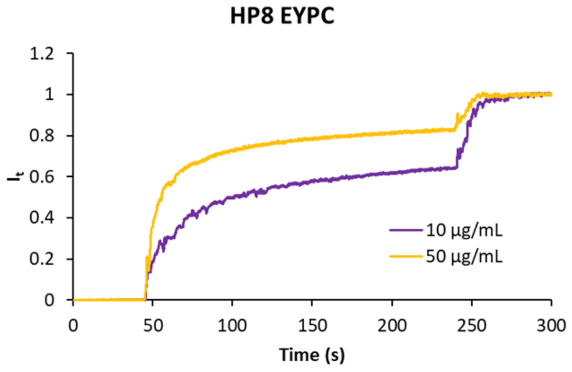
HP8 EYPC
I_t
Time (s)
10 µg/mL
50 µg/mL

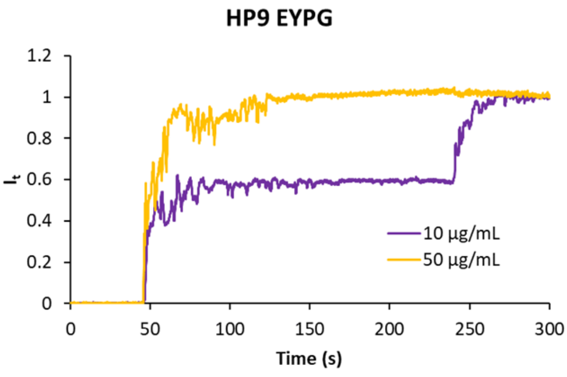
HP9 EYPG
I_t
Time (s)
10 µg/mL
50 µg/mL

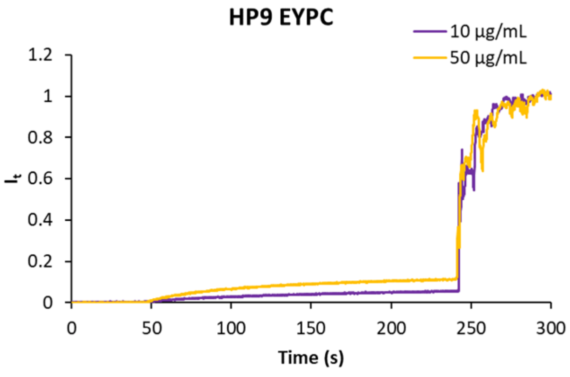
HP9 EYPC
I_t
Time (s)
10 µg/mL
50 µg/mL

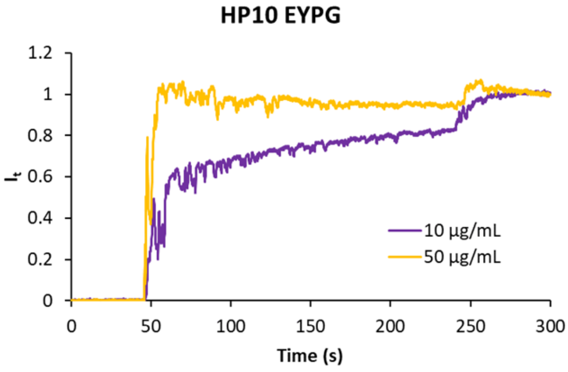
HP10 EYPG
I_t
Time (s)
10 µg/mL
50 µg/mL

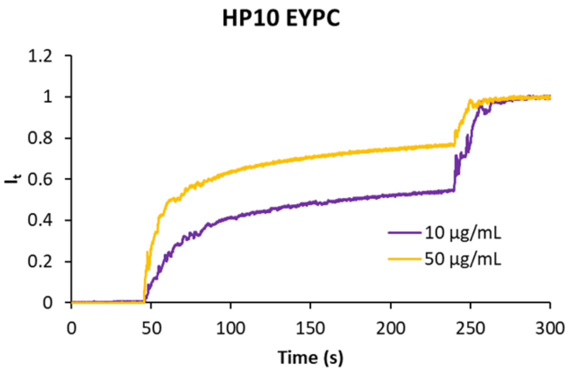
HP10 EYPC
I_t
Time (s)
10 µg/mL
50 µg/mL

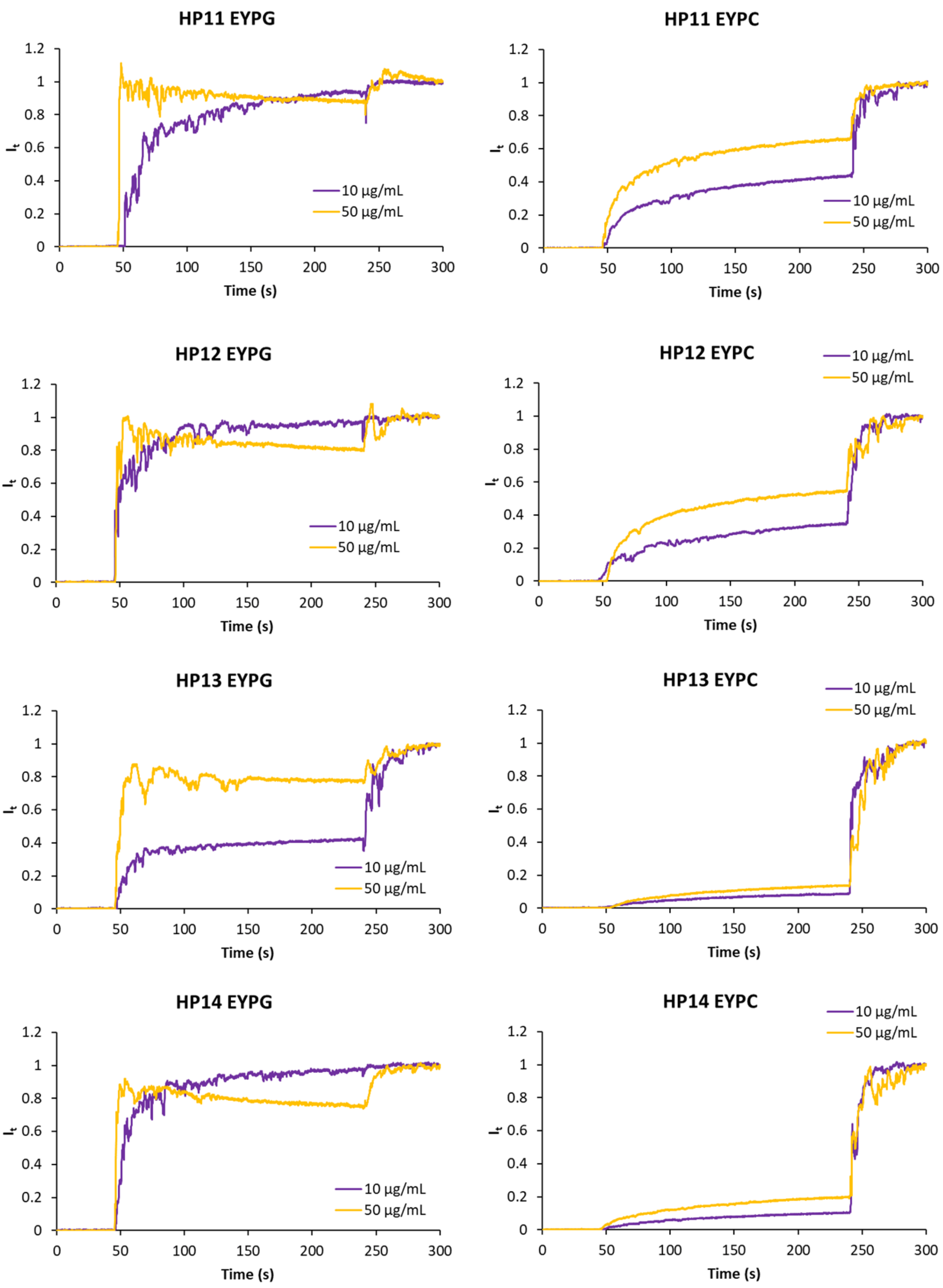
HP11 EYPG
HP11 EYPC
HP12 EYPG
HP12 EYPC
HP13 EYPG
HP13 EYPC
HP14 EYPG
HP14 EYPC
10 µg/mL
50 µg/mL
It
Time (s)
0
0.2
0.4
0.6
0.8
1
1.2
0
50
100
150
200
250
300

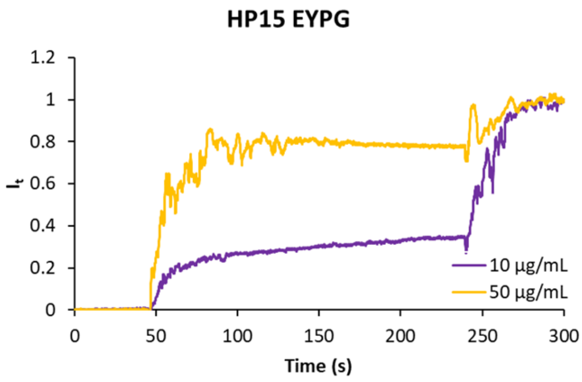
HP15 EYPG
I_t
Time (s)
10 µg/mL
50 µg/mL

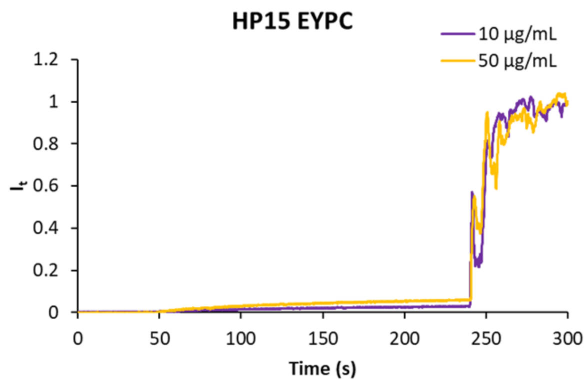
HP15 EYPC
I_t
Time (s)
10 µg/mL
50 µg/mL

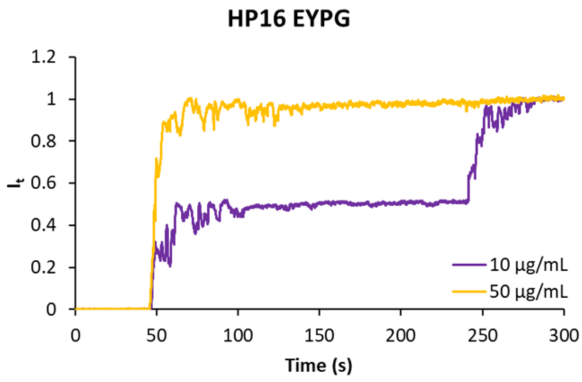
HP16 EYPG
I_t
Time (s)
10 µg/mL
50 µg/mL

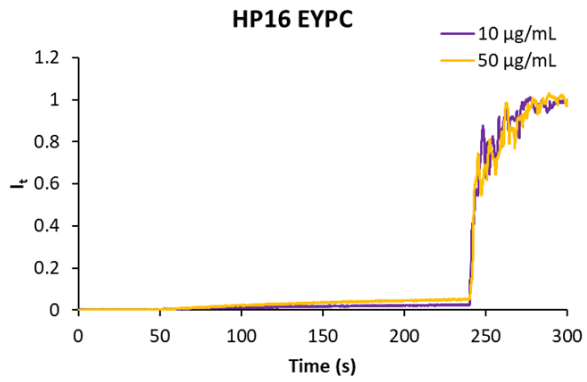
HP16 EYPC
I_t
Time (s)
10 µg/mL
50 µg/mL

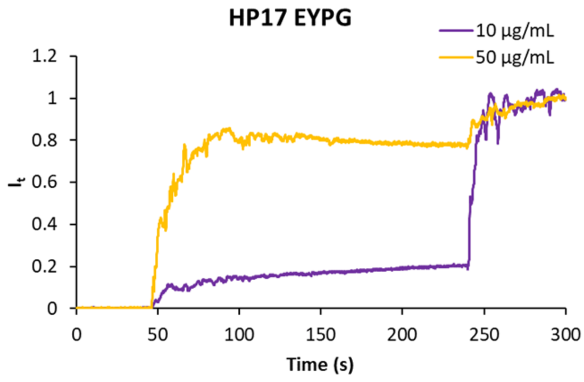
HP17 EYPG
I_t
Time (s)
10 µg/mL
50 µg/mL

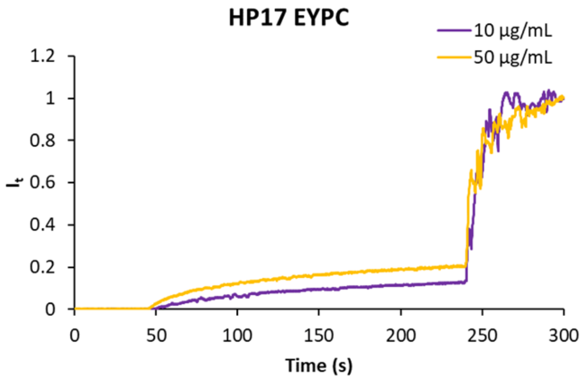
HP17 EYPC
I_t
Time (s)
10 µg/mL
50 µg/mL

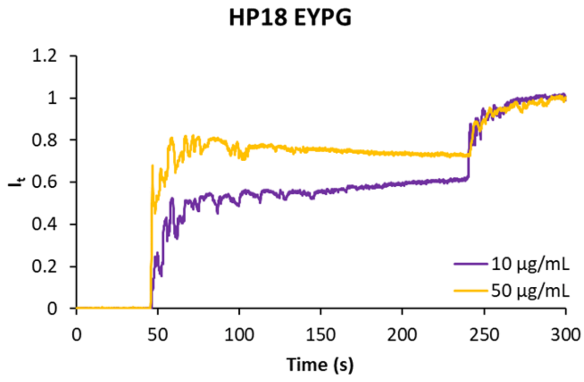
HP18 EYPG
I_t
Time (s)
10 µg/mL
50 µg/mL

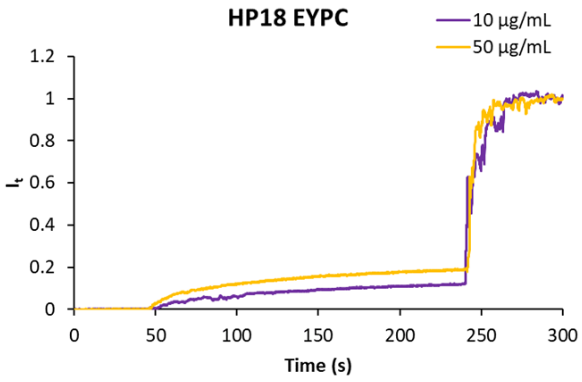
HP18 EYPC
I_t
Time (s)
10 µg/mL
50 µg/mL

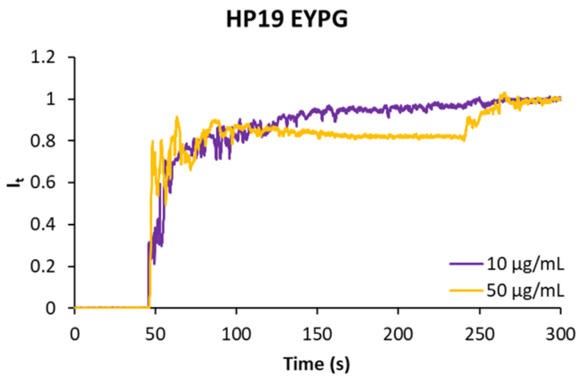
HP19 EYPG
It
Time (s)
10 µg/mL
50 µg/mL

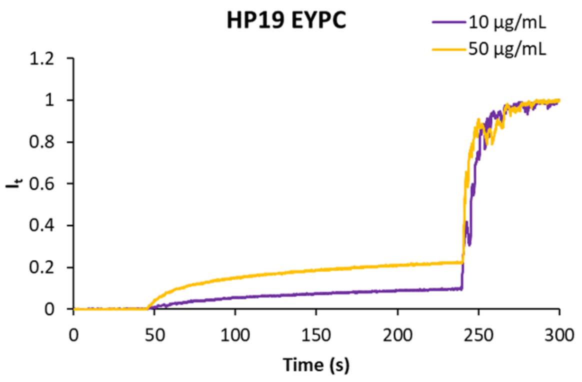
HP19 EYPC
It
Time (s)
10 µg/mL
50 µg/mL

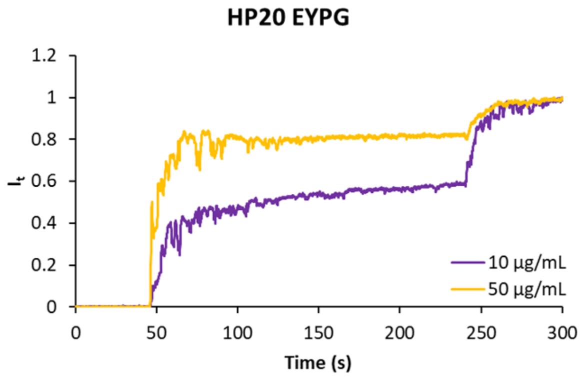
HP20 EYPG
It
Time (s)
10 µg/mL
50 µg/mL

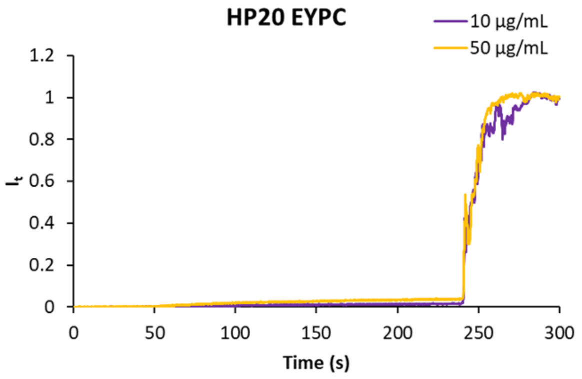
HP20 EYPC
It
Time (s)
10 µg/mL
50 µg/mL

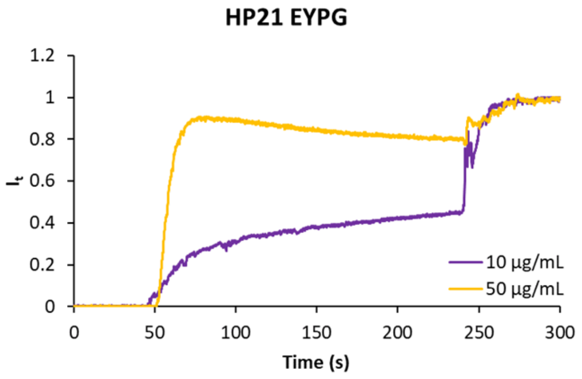
HP21 EYPG
It
Time (s)
10 µg/mL
50 µg/mL

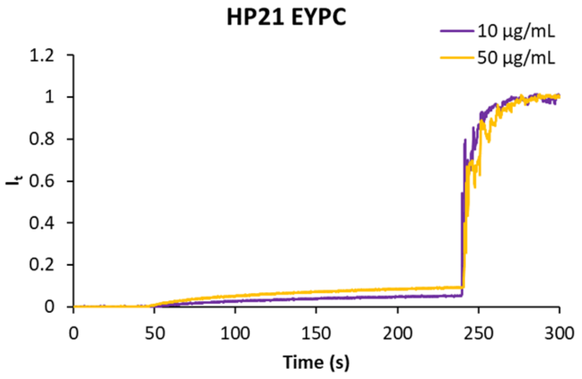
HP21 EYPC
It
Time (s)
10 µg/mL
50 µg/mL

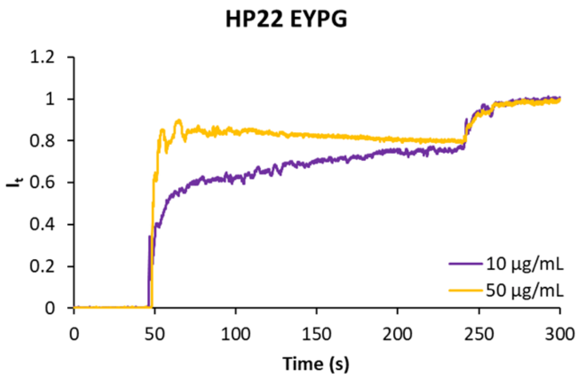
HP22 EYPG
It
Time (s)
10 µg/mL
50 µg/mL

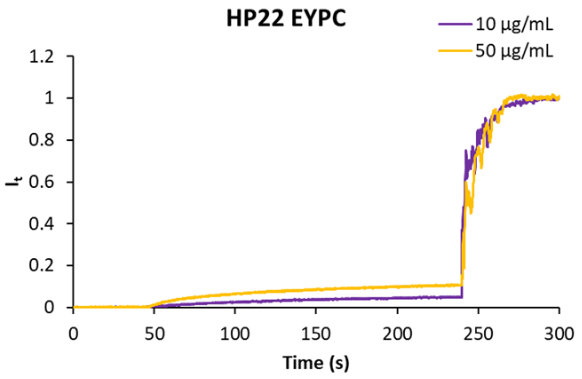
HP22 EYPC
It
Time (s)
10 µg/mL
50 µg/mL

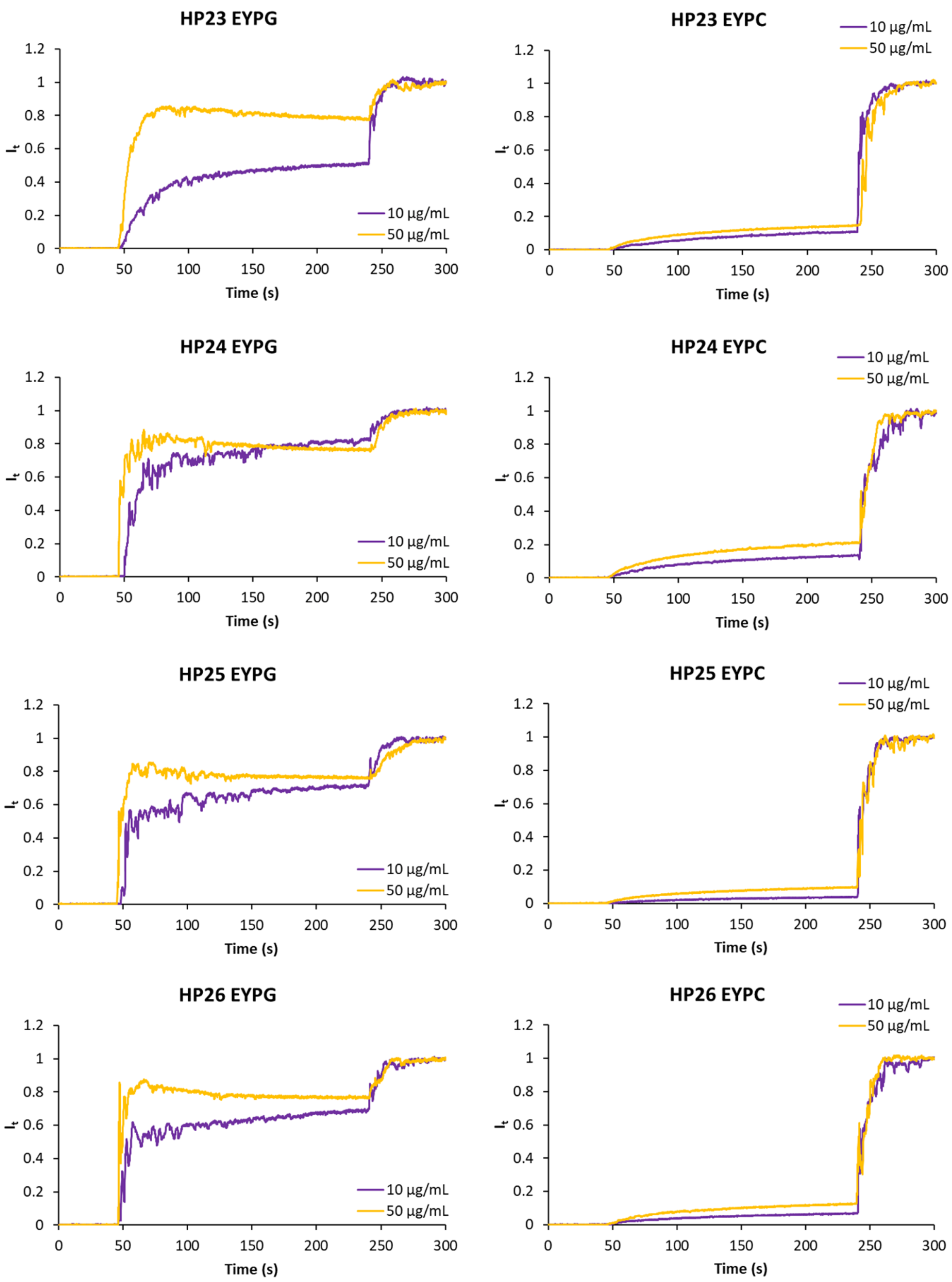
HP23 EYPG
HP23 EYPC
HP24 EYPG
HP24 EYPC
HP25 EYPG
HP25 EYPC
HP26 EYPG
HP26 EYPC
10 µg/mL
50 µg/mL
I_t
Time (s)
0
0.2
0.4
0.6
0.8
1
1.2
0
50
100
150
200
250
300

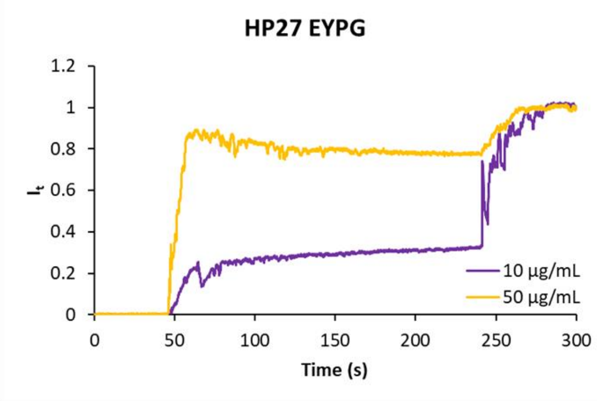
HP27 EYPG
10 µg/mL
50 µg/mL
It
Time (s)

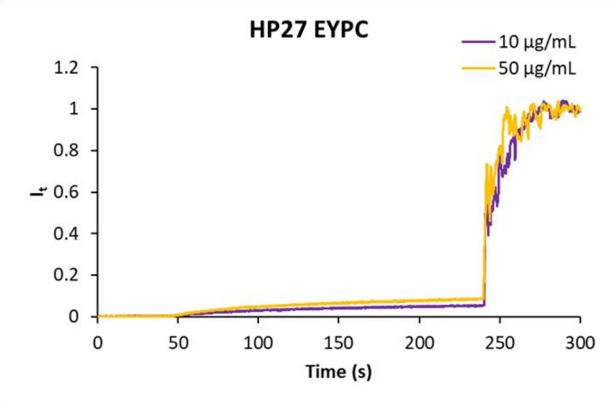
HP27 EYPC
10 µg/mL
50 µg/mL
It
Time (s)

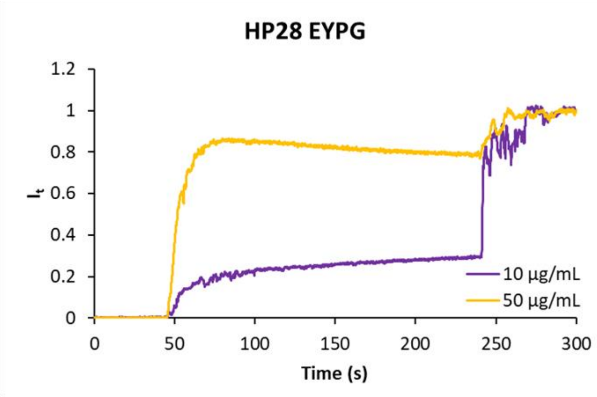
HP28 EYPG
10 µg/mL
50 µg/mL
It
Time (s)

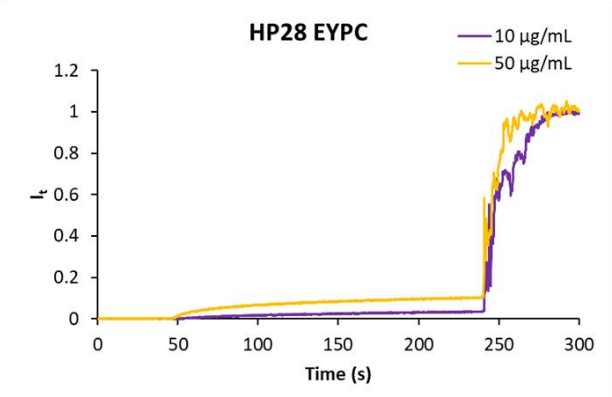
HP28 EYPC
10 µg/mL
50 µg/mL
It
Time (s)

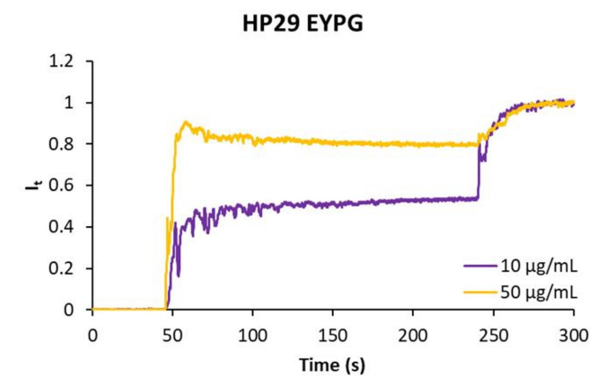
HP29 EYPG
10 µg/mL
50 µg/mL
It
Time (s)

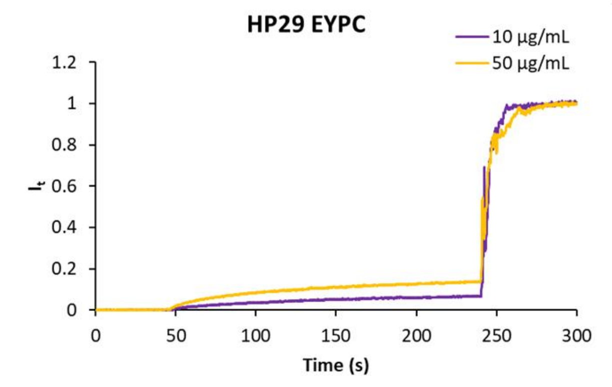
HP29 EYPC
10 µg/mL
50 µg/mL
It
Time (s)

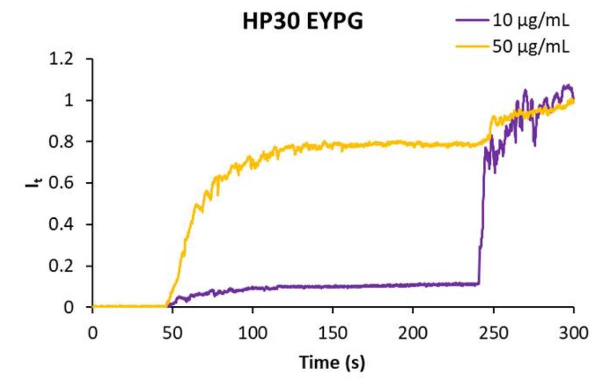
HP30 EYPG
10 µg/mL
50 µg/mL
It
Time (s)

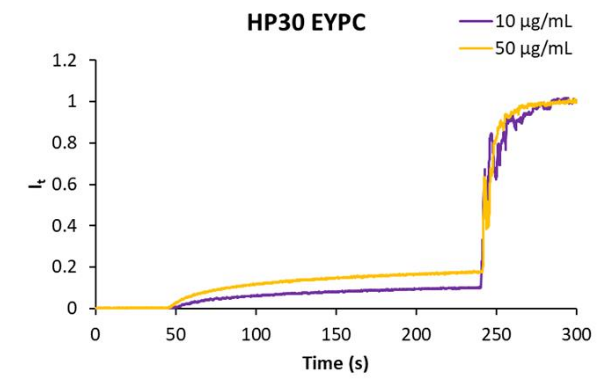
HP30 EYPC
10 µg/mL
50 µg/mL
It
Time (s)

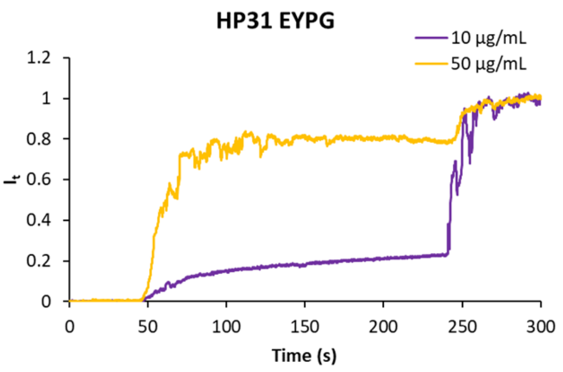
HP31 EYPG
10 µg/mL
50 µg/mL
It
1.2
1
0.8
0.6
0.4
0.2
0
0
50
100
150
200
250
300
Time (s)

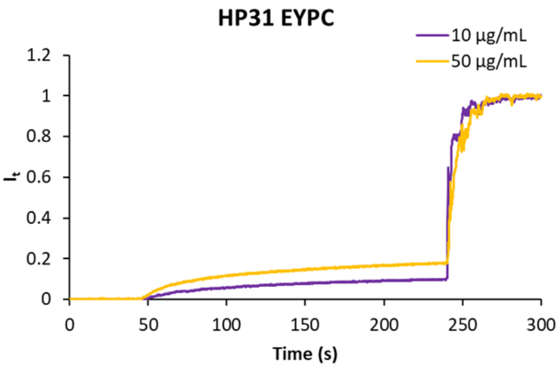
HP31 EYPC
10 µg/mL
50 µg/mL
It
1.2
1
0.8
0.6
0.4
0.2
0
0
50
100
150
200
250
300
Time (s)

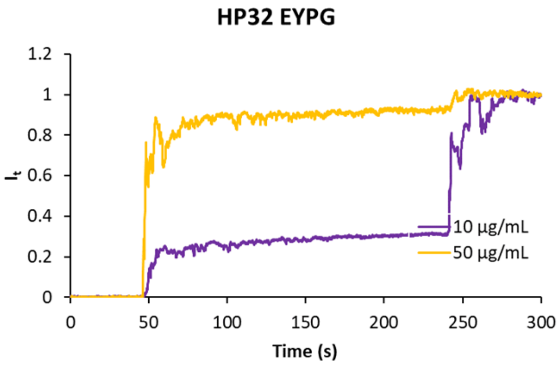
HP32 EYPG
10 µg/mL
50 µg/mL
It
1.2
1
0.8
0.6
0.4
0.2
0
0
50
100
150
200
250
300
Time (s)

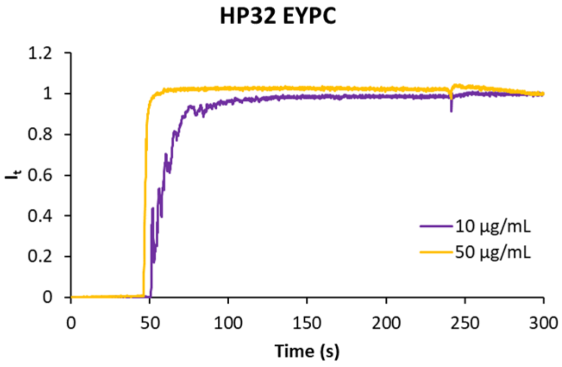
HP32 EYPC
10 µg/mL
50 µg/mL
It
1.2
1
0.8
0.6
0.4
0.2
0
0
50
100
150
200
250
300
Time (s)

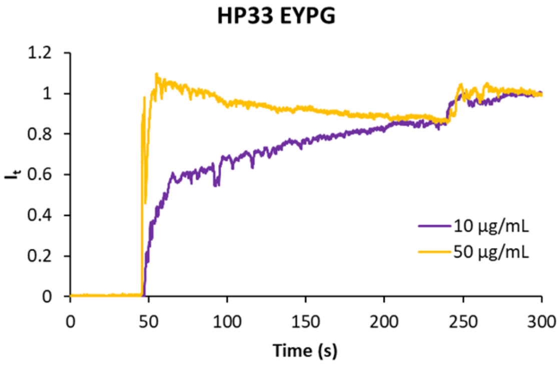
HP33 EYPG
10 µg/mL
50 µg/mL
It
1.2
1
0.8
0.6
0.4
0.2
0
0
50
100
150
200
250
300
Time (s)

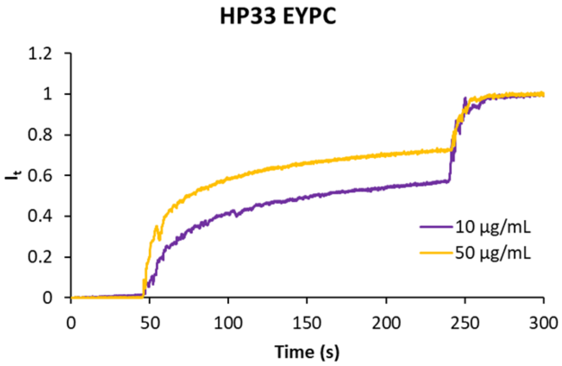
HP33 EYPC
10 µg/mL
50 µg/mL
It
1.2
1
0.8
0.6
0.4
0.2
0
0
50
100
150
200
250
300
Time (s)

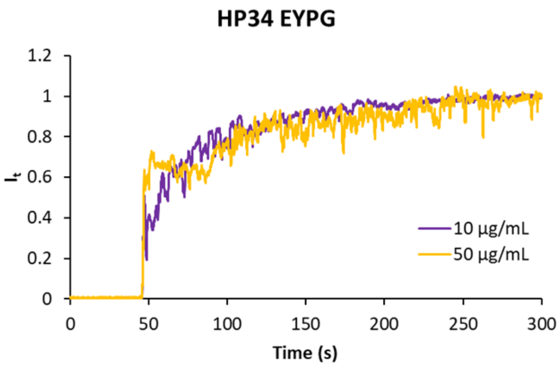
HP34 EYPG
10 µg/mL
50 µg/mL
It
1.2
1
0.8
0.6
0.4
0.2
0
0
50
100
150
200
250
300
Time (s)

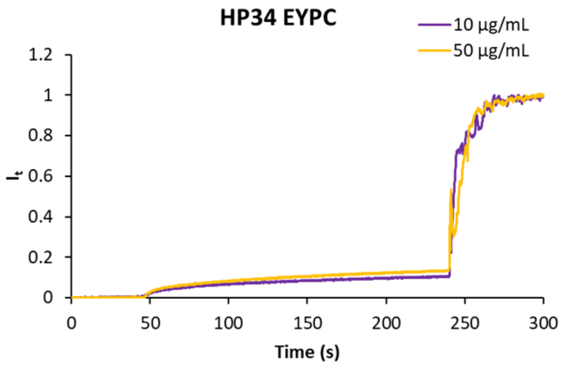
HP34 EYPC
10 µg/mL
50 µg/mL
It
1.2
1
0.8
0.6
0.4
0.2
0
0
50
100
150
200
250
300
Time (s)

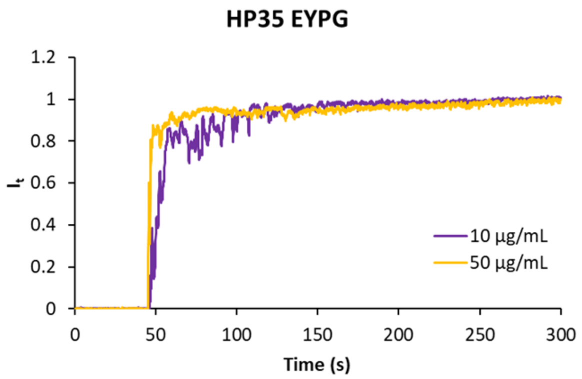
HP35 EYPG
It
Time (s)
10 µg/mL
50 µg/mL

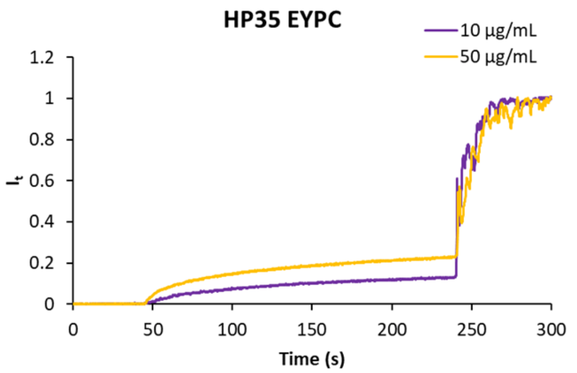
HP35 EYPC
It
Time (s)
10 µg/mL
50 µg/mL

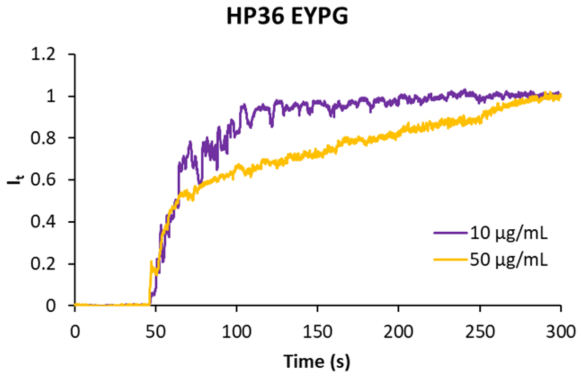
HP36 EYPG
It
Time (s)
10 µg/mL
50 µg/mL

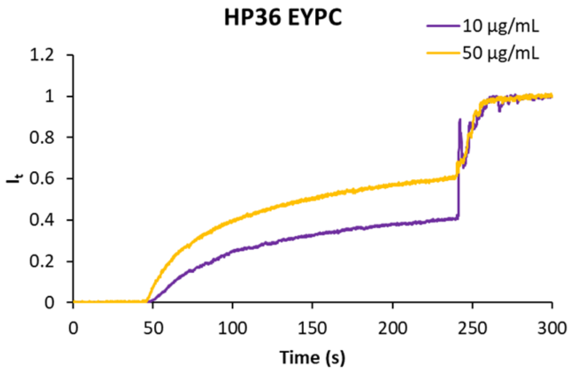
HP36 EYPC
It
Time (s)
10 µg/mL
50 µg/mL

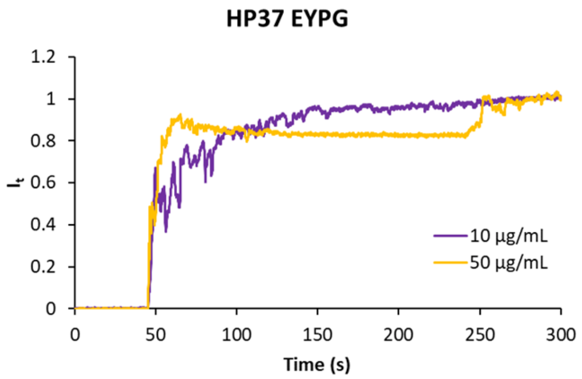
HP37 EYPG
It
Time (s)
10 µg/mL
50 µg/mL

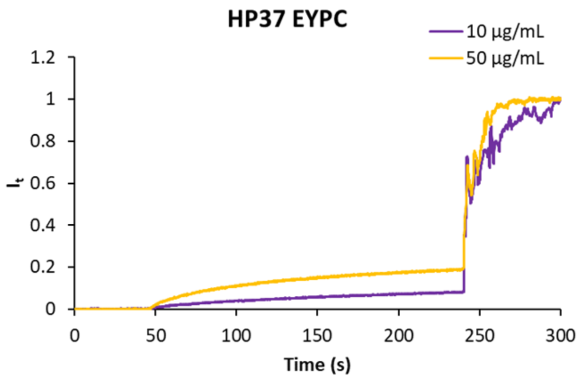
HP37 EYPC
It
Time (s)
10 µg/mL
50 µg/mL

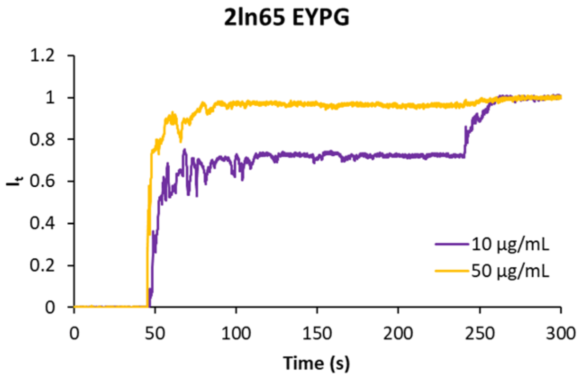
2In65 EYPG
It
Time (s)
10 µg/mL
50 µg/mL

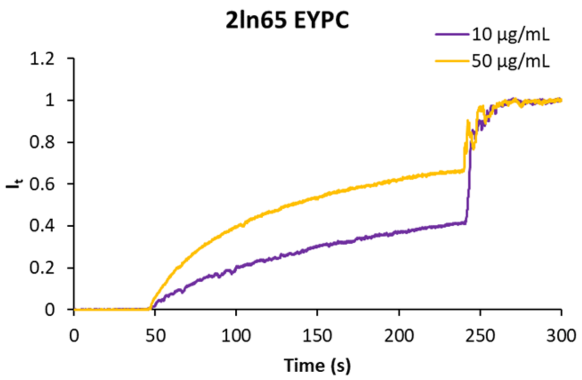
2In65 EYPC
It
Time (s)
10 µg/mL
50 µg/mL

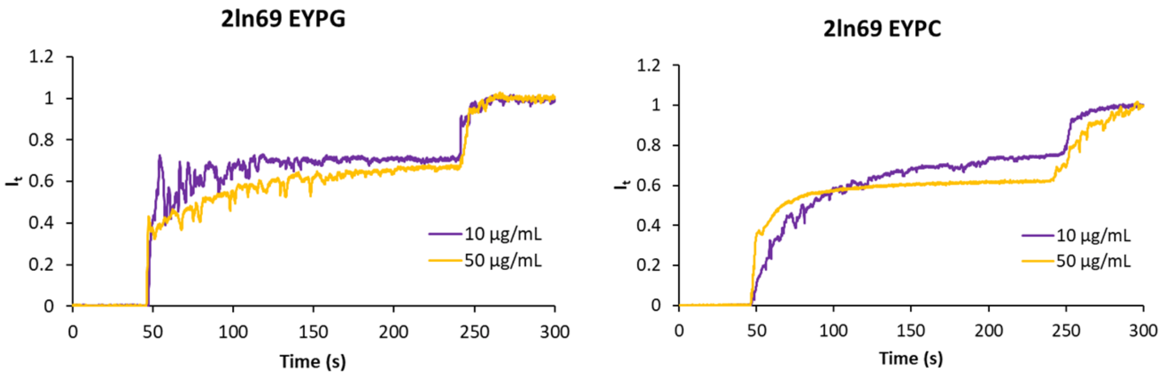

Figure S2: (from page S14 to 25) Vesicle leakage experiments using 5(6)-carboxyfluorescein induced by peptides. EYPG and EYPC vesicles were suspended in buffer (10 mM Tris, 107 mM NaCl, pH 7.4) and the indicated concentration of the compound was added after 45 seconds. After 240 seconds 30 µL of Triton X-100 1.2% was added for full release of the fluorescein.

4. Time kill kinetics assay

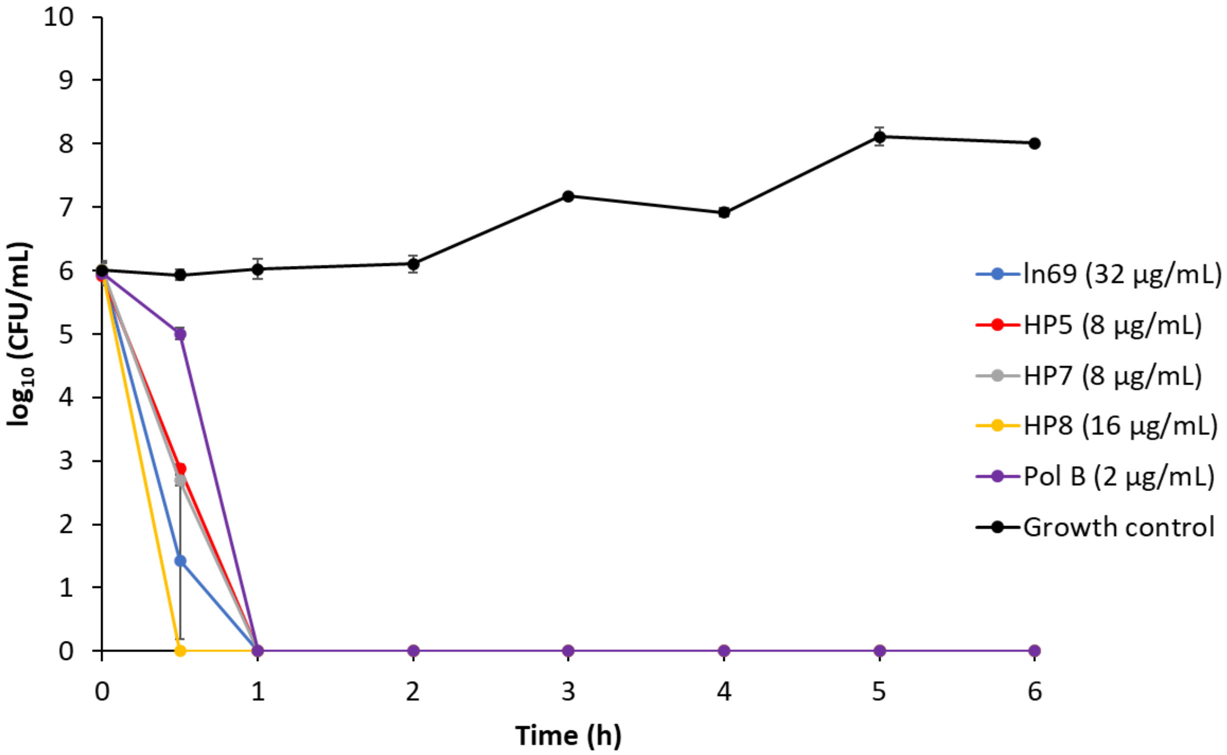

Figure S3: Bacteria killing assay at pH 7.4 against *P. aeruginosa* PAO1 at a concentration of 4 × MIC. Data are given as the mean ± SD, n = 3. A value of $\log_{10}$ of 0 means no colony was observed.

5. Serum stability assay

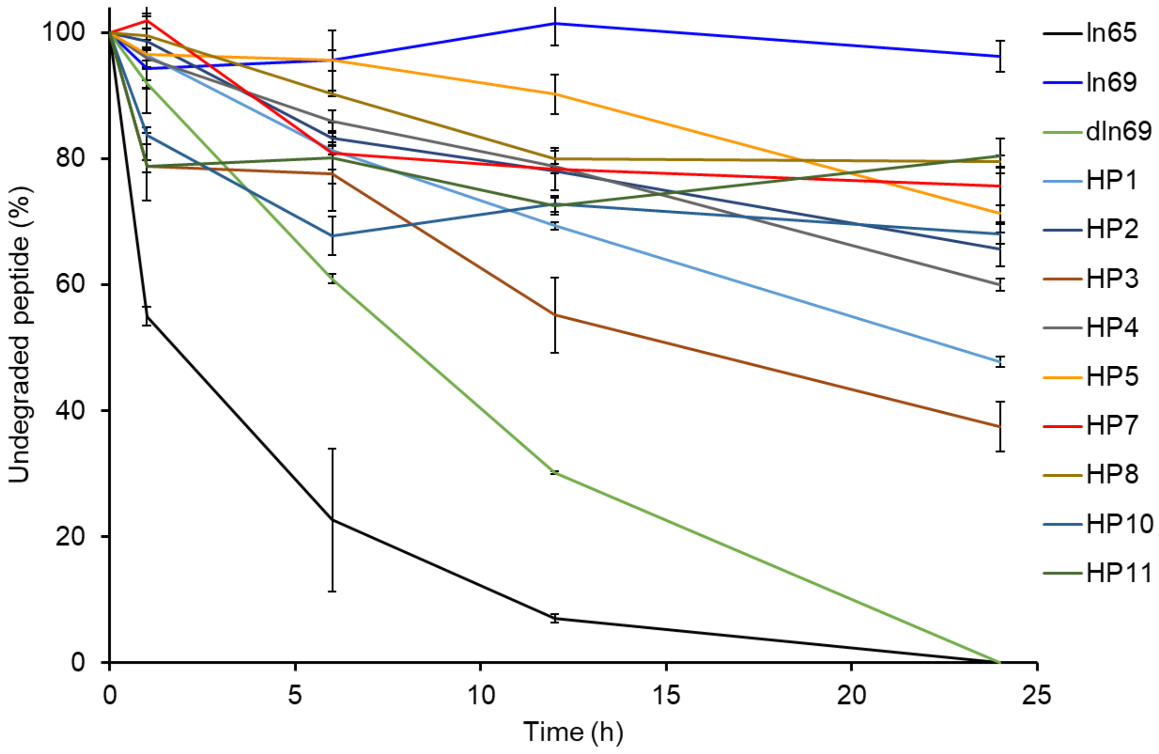

Figure S4: Serum stability of mixed-chirality peptides in 12.5% human serum in 0.1 M filtered TRIS buffer pH 7.4. Normalized undegraded peptides values were determined by RP-HPLC analysis using hydroxybenzoic acid as internal standard. Data from triplicate experiments.

6. Cytotoxicity assay

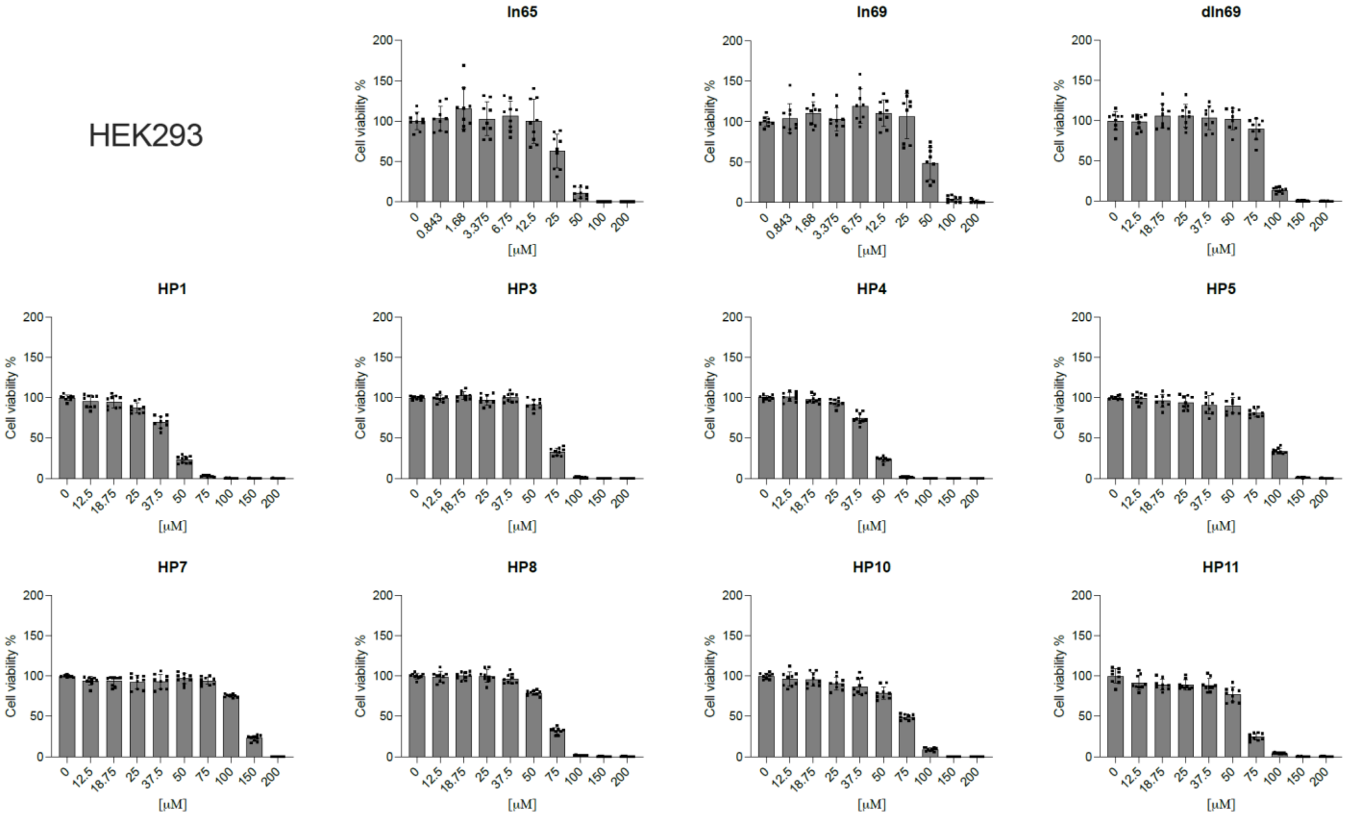

Figure S5: Cytotoxicity **ln65**, **ln69**, **dln69**, **HP1**, **HP3**, **HP4**, **HP5**, **HP7**, **HP8**, **HP10** and **HP11** on HEK293 cells. The data of three experiments with three replicates per sample were pooled and represented as barplots. The cells were treated with the desired concentration of compound for 24 h and their viability was measured with an Alamar Blue assay.

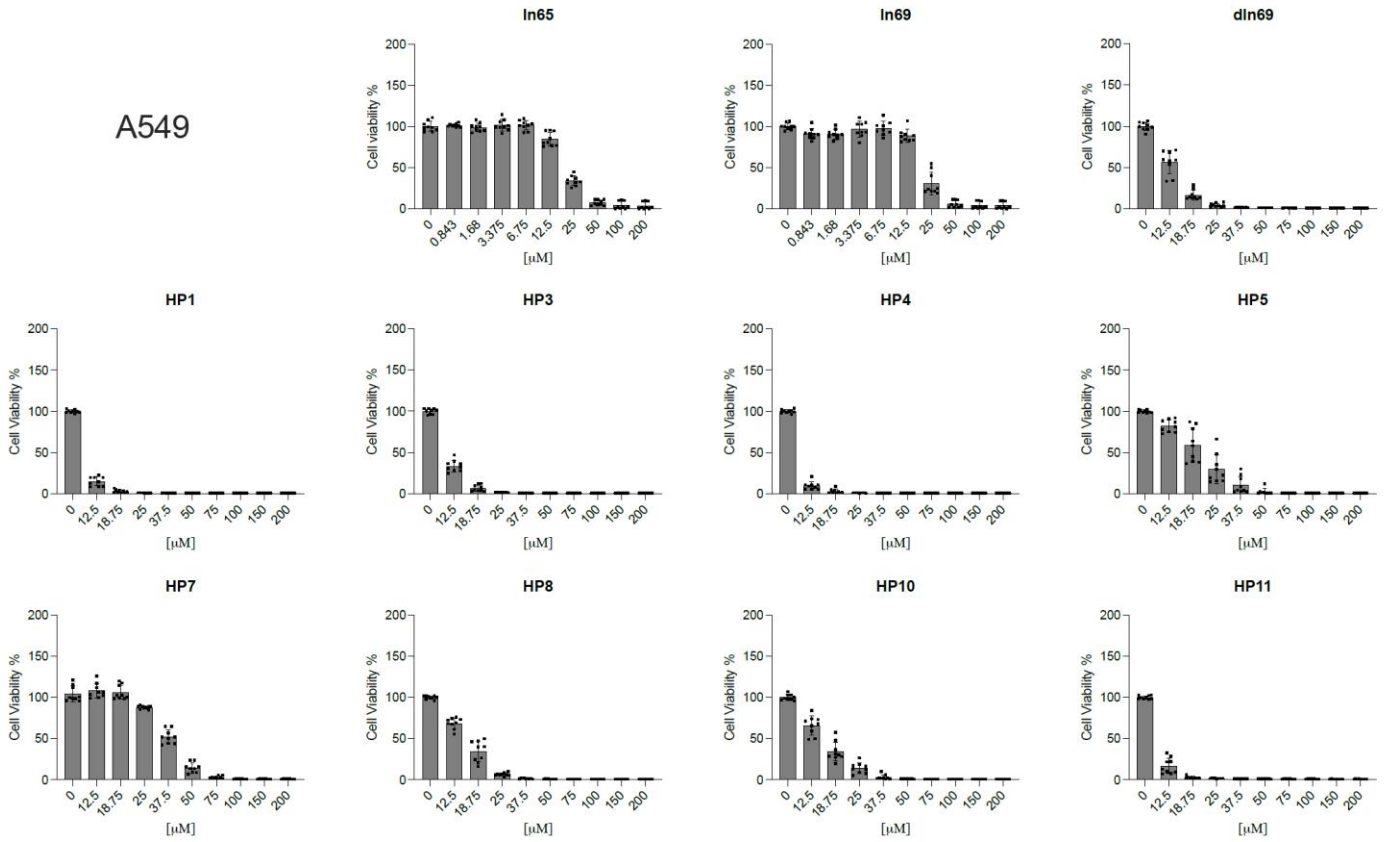

Figure S6: Cytotoxicity **ln65**, **ln69**, **dln69**, **HP1**, **HP3**, **HP4**, **HP5**, **HP7**, **HP8**, **HP10** and **HP11** on A549 cells. The data of three experiments with three replicates per sample were pooled and represented as barplots. The cells were treated with the desired concentration of compound for 24 h and their viability was measured with an Alamar Blue assay.

7. Crystallography

Table S3: Data collection and refinement statistics for the X-ray structure of the **FHP5**·LecB complex.

Structural data	FHP5·LecB
Beam line	PSI PXIII
Wavelength (Å)	1.000
Resolution (Å)	50.0-1.3 (1.35-1.27)[a)]
**Cell dimension**	
Space group	4, P 1 2_1 1
Unit cell (Å)	*a* = 52.8, *b* = 62.8, *c* = 64.2<br>α = 90.0°, β = 92.8°, γ = 90.0°
Measured reflection / unique	660242/211407
Average multiplicity	3.1 (2.4)
Completeness (%)	98.9 (92.4)
Average $I / \sigma(I)$	9.16 (1.08)
Correlation $CC_{1/2}$ (%)	99.8 (71.7)
R_{meas} (%)	6.9 (84.1)
Wilson β-factor ($Å^2$)	13.0
**Refinement**	
Resolution range (Å)	44.90-1.27
R_{work} (%)	16.27
R_{free} (%)	19.19
Average Biso ($Å^2$)	99.3
RMSD from ideal angles (°)	1.005
Bonds (Å)	0.007
Water molecules	492
Ligand molecules	4
Protein data Bank deposition code	8AN9

[a)] Values in brackets correspond to the outer shell.

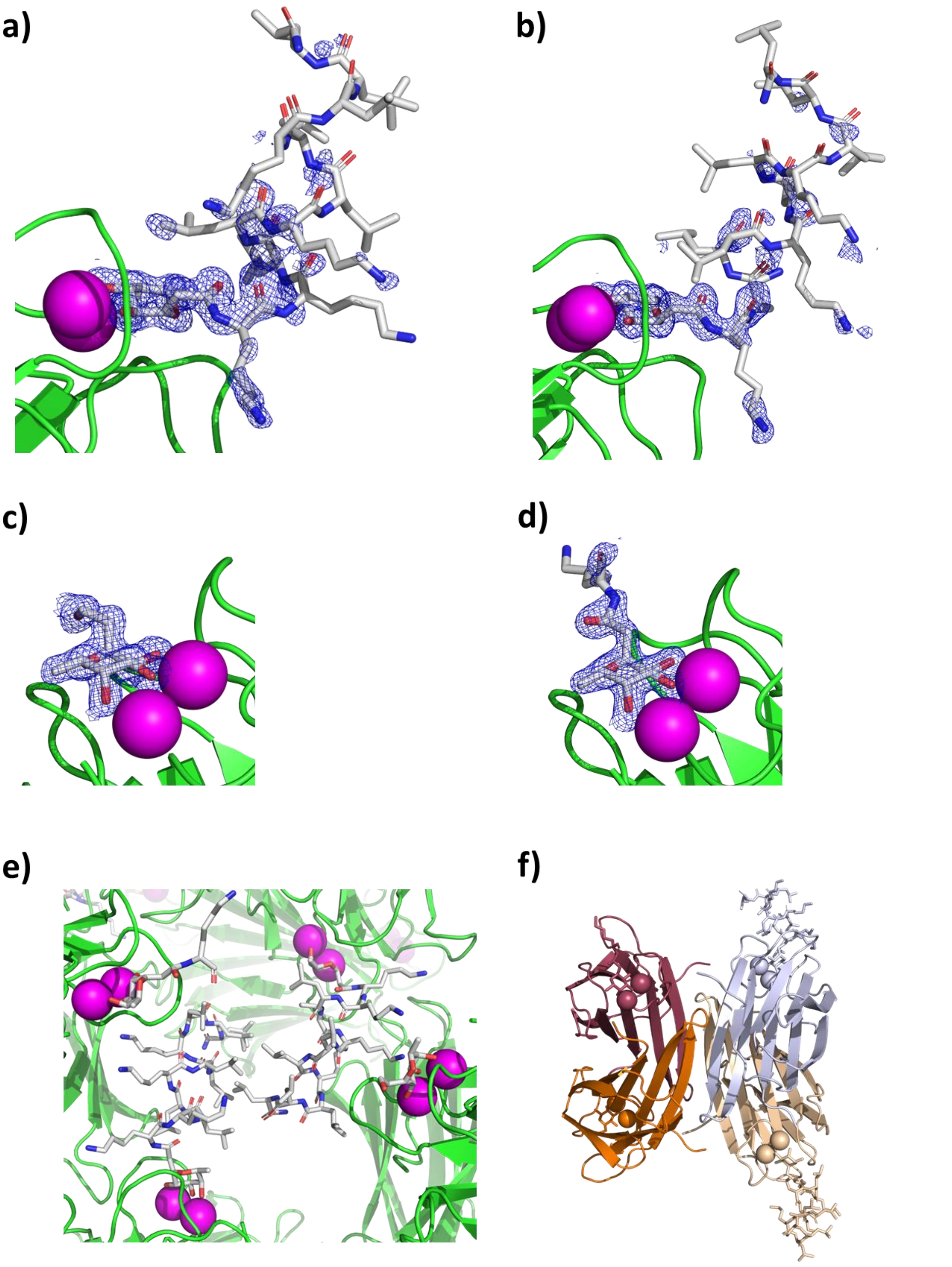

Figure S7: Details of the X-ray structure of the **FHP5**·LecB complex in P 1 2_1 1. **(a-d)** Asymmetric peptide entities with corresponding electron density map as blue mesh, Ca^{2+} atoms as magenta spheres and the bound LecB monomer as green cartoon. Electron density is shown for a 1.0σ level. **(e)** Incomplete bundles of four different asymmetric complete and incomplete peptides Same color code as in (a). **(f)** View of the unit cell including LecB subunits, Ca^{2+} atoms and the bound peptides. Peptides are shown as sticks, Ca^{2+} atoms as spheres and lectin monomers are displayed as cartoon of the same color.

Table S4: Data collection and refinement statistics for the X-ray structure of the **FHP8**·LecB complex.

**Structural data**	**FHP8·LecB**
Beam line	PSI PXIII
Wavelength (Å)	1.000
Resolution (Å)	50.0-1.3 (1.36-1.29)[a)]
**Cell dimension**	
Space group	4, P 1 2_1 1
Unit cell (Å)	*a* = 52.8, *b* = 62.9, *c* = 64.6<br>α = 90.0°, β = 92.9°, γ = 90.0°
Measured reflection / unique	660731/207353
Average multiplicity	3.2 (2.6)
Completeness (%)	99.0 (92.7)
Average $I / \sigma(I)$	8.08 (0.81)
Correlation $CC_{1/2}$ (%)	99.7 (81.0)
R_{meas} (%)	9.9 (143.3)
Wilson β-factor (Å^2)	10.7
**Refinement**	
Resolution range (Å)	45.00-1.29
R_{work} (%)	16.66
R_{free} (%)	20.17
Average Biso (Å^2)	83.8
RMSD from ideal angles (°)	1.225
Bonds (Å)	0.012
Water molecules	570
Ligand molecules	4
Protein data Bank deposition code	8ANO

[a)] Values in brackets correspond to the outer shell.

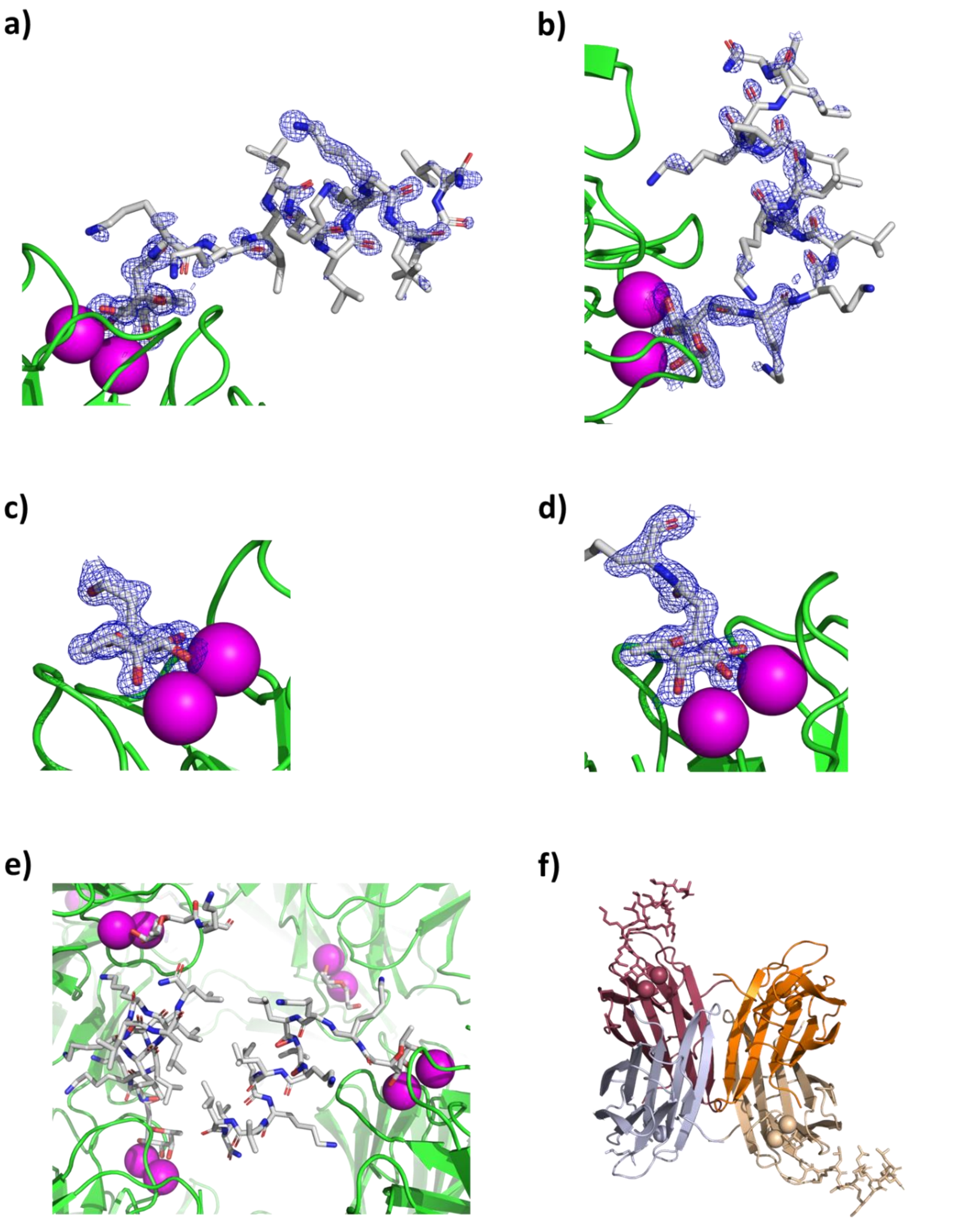

Figure S8: Details of the X-ray structure of the **FHP8**·LecB complex in P 1 2_1 1. **(a-d)** Asymmetric peptide entities with corresponding electron density map as blue mesh, Ca^{2+} atoms as magenta spheres and the bound LecB monomer as green cartoon. Electron density is shown for a 1.0σ level. **(e)** Incomplete bundles of four different asymmetric complete and incomplete peptides Same color code as in (a). **(f)** View of the unit cell including LecB subunits, Ca^{2+} atoms and the bound peptides. Peptides are shown as sticks, Ca^{2+} atoms as spheres and lectin monomers are displayed as cartoon of the same colour.

Table S5: Data collection and refinement statistics for the X-ray structure of the **FHP30**·LecB complex.

**Structural data**	**FHP30·LecB**
Beam line	PSI PXIII
Wavelength (Å)	1.000
Resolution (Å)	50.0-1.6 (1.72-1.62)[a]
**Cell dimension**	
Space group	94, P 4_2 2_1 2
Unit cell (Å)	a = 70.7, b = 70.7, c = 103.4<br>α = 90.0°, β = 90.0°, γ = 90.0°
Measured reflection / unique	885904/63954
Average multiplicity	13.9 (13.4)
Completeness (%)	100.0 (99.9)
Average I / $\sigma(I)$	15.46 (0.98)
Correlation $CC_{1/2}$ (%)	99.9 (37.8)
R_{meas} (%)	15.2 (284.0)
Wilson β-factor ($Å^2$)	18.1
**Refinement**	
Resolution range (Å)	45.00-1.62
R_{work} (%)	14.82
R_{free} (%)	18.34
Average Biso ($Å^2$)	83.1
RMSD from ideal angles (°)	0.802
Bonds (Å)	0.005
Water molecules	266
Ligand molecules	2
Protein data Bank deposition code	8ANR

[a] Values in brackets correspond to the outer shell.

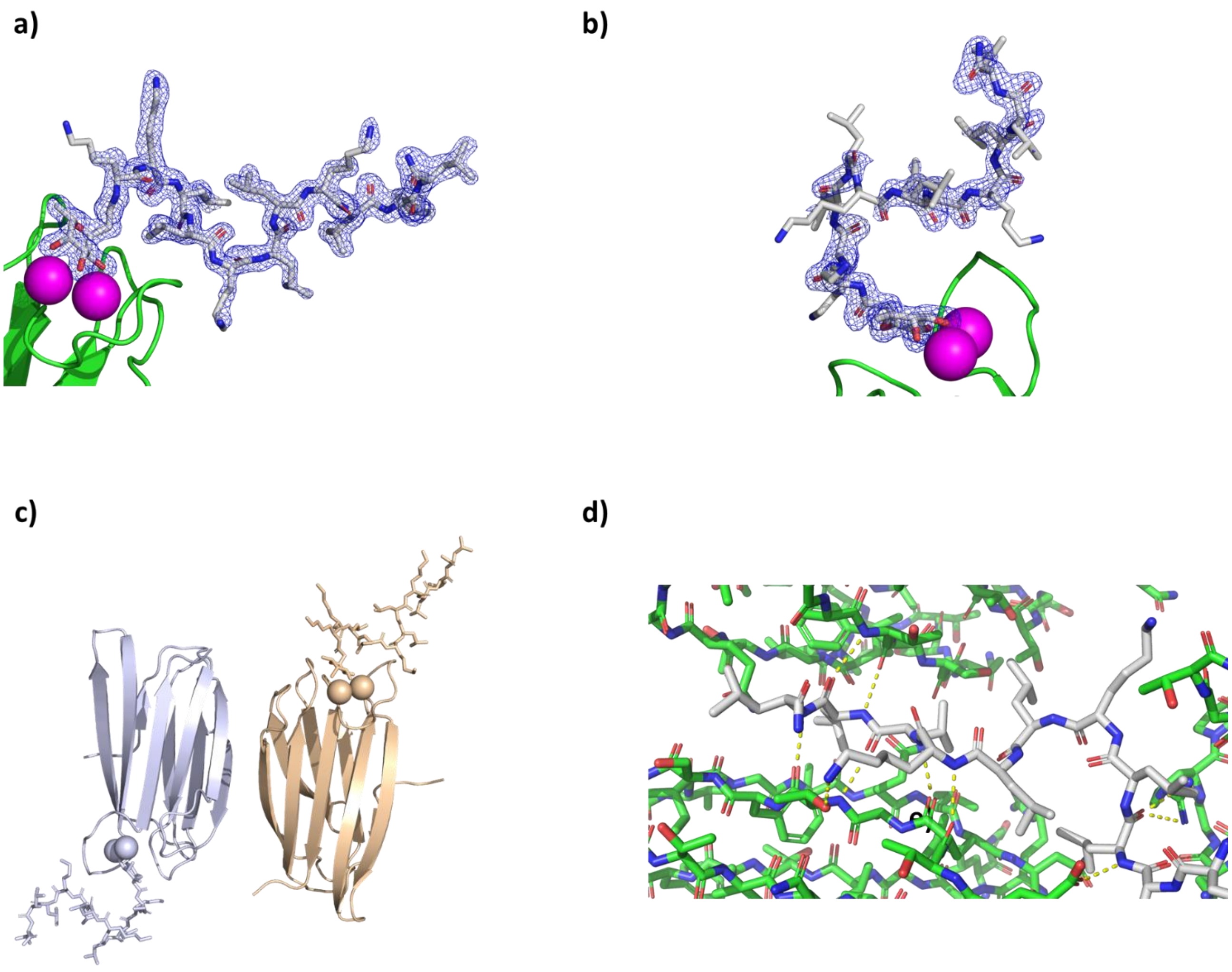

Figure S9: Details of the X-ray structure of the **FHP30**·LecB complex in P 4_2 2_1 2. **(a-b)** Asymmetric peptide entities with corresponding electron density map as blue mesh, Ca^{2+} atoms as magenta spheres and the bound LecB monomer as green cartoon. Electron density is shown for a 1.0σ level. **(c)** View of the unit cell including LecB subunits, Ca^{2+} atoms and the bound peptides. Peptides are shown as sticks, Ca^{2+} atoms as spheres and lectin monomers are displayed as cartoon of the same color. **(d)** Hydrogen bonds (yellow dashed lines) between lectin B monomers and **FHP30** (chain C).

Table S6: Data collection and refinement statistics for the X-ray structure of the **FHP31**·LecB complex.

**Structural data**	**FHP31·LecB**
Beam line	PSI PXIII
Wavelength (Å)	1.000
Resolution (Å)	50.0-1.2 (1.25-1.18)[a)]
**Cell dimension**	
Space group	4, P 1 2_1 1
Unit cell (Å)	*a* = 52.8, *b* = 62.7, *c* = 64.4<br>α = 90.0°, β = 93.2°, γ = 90.0°
Measured reflection / unique	714411/235325
Average multiplicity	3.0 (1.3)
Completeness (%)	90.1 (35.0)
Average $I / \sigma(I)$	20.70 (2.50)
Correlation $CC_{1/2}$ (%)	99.9 (81.2)
R_{meas} (%)	4.4 (46.5)
Wilson β-factor ($Å^2$)	9.4
**Refinement**	
Resolution range (Å)	44.88-1.18
R_{work} (%)	12.68
R_{free} (%)	15.14
Average Biso ($Å^2$)	60.1
RMSD from ideal angles (°)	1.196
Bonds (Å)	0.009
Water molecules	656
Ligand molecules	4
Protein data Bank deposition code	8AOO

[a)] Values in brackets correspond to the outer shell.

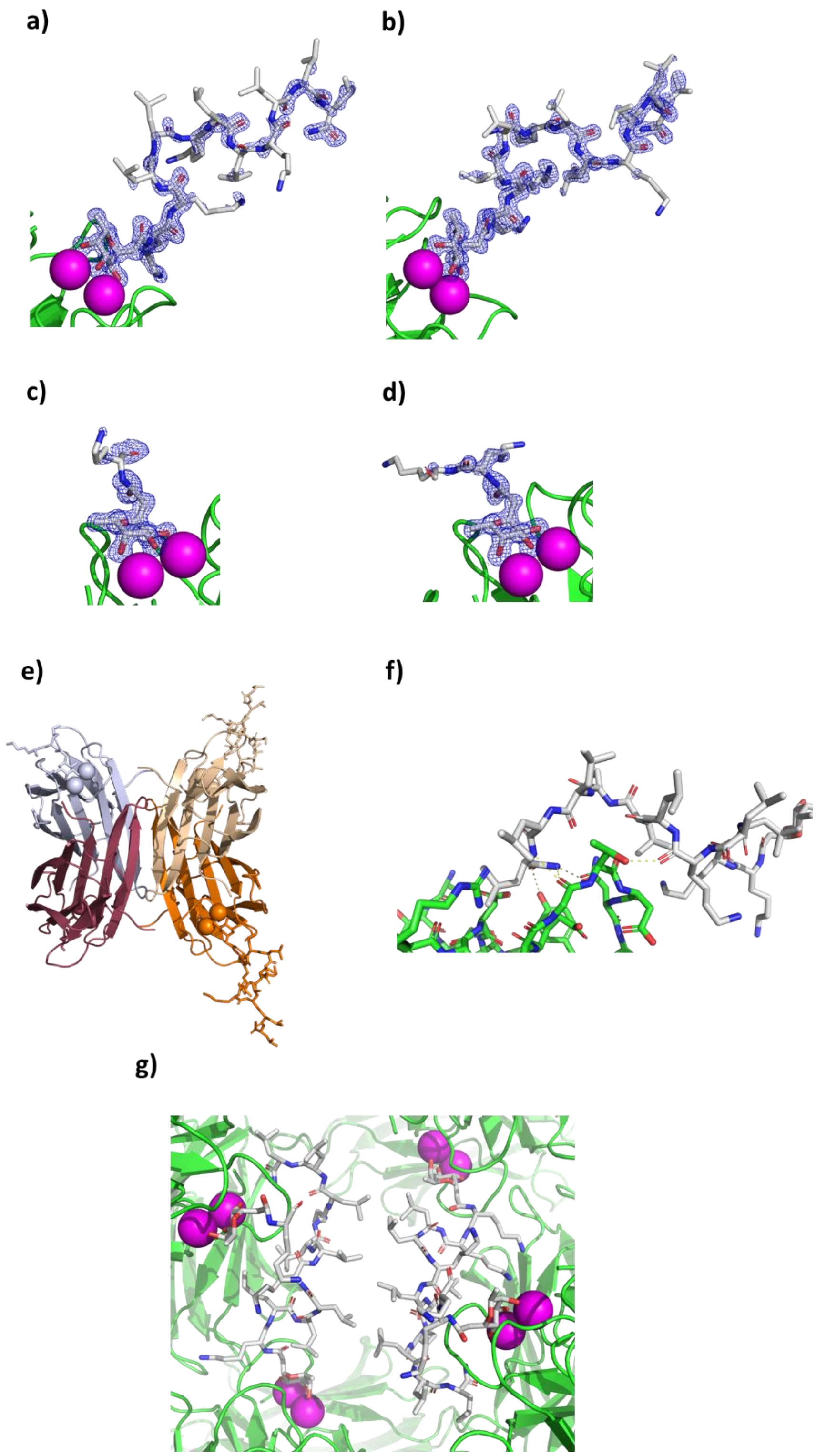

Figure S10: Details of the X-ray structure of the **FHP31**·LecB complex in P 1 2_1 1. **(a-d)** Asymmetric peptide entities with corresponding electron density map as blue mesh, Ca^{2+} atoms as magenta spheres and the bound LecB monomer as green cartoon. Electron density is shown for a 1.0σ level. **(e)** View of the unit cell including LecB subunits, Ca^{2+} atoms and the bound peptides. Peptides are shown as sticks, Ca^{2+} atoms as spheres and lectin monomers are displayed as cartoon of the same color. **(f)** H-bonds interaction (yellow dashed lines) between FHP31 (grey stick) and lectin monomer (green stick). (g) Incomplete bundle of four different complete and incomplete peptides. Same color code as in (a).

8. Molecular dynamics

Topology of DPC molecules[2]

```
; Charge from Chiu et al.
; Chiu, S. W.; Clark, M.; Balaji, V.; Subramaniam, S.; Scott, H. L.; Jakobsson, E.
Incorporation of surface tension into molecular dynamics simulation of an interface: a fluid
phase lipid bilayer membrane. Biophys. J. 1995, 69, 1230-1245.
; Atom types from GROMOS53A6
; Oostenbrink, C.; Soares, T. A.; van der Vegt, N. F. A.; van Gunsteren, W. F. Validation of
the 53A6 GROMOS force field. Eur. Biophys. J. 2005, 34, 273-284.

[ moleculetype ]
; Name   nrexcl
DPC      3

[ atoms ]
;   nr    type   resnr  residu    atom    cgnr        charge          mass
     1     CH3       1    DPC      C1       1         0.40  15.035  ; qtot: 0.25
     2     CH3       1    DPC      C2       2         0.40  15.035  ; qtot: 0.50
     3     CH3       1    DPC      C3       3         0.40  15.035 ; qtot: 0.75
     4     NL        1    DPC      N4       4        -0.5  14.0067 ; qtot: 0.75
     5     CH2       1    DPC      C5       5         0.30  14.027 ; qtot:  1.0
     6     CH2       1    DPC      C6       6         0.40  14.027 ; qtot: 1.0
     7      OA       1    DPC      O7       7        -0.80  15.999 ; qtot: 0.64
     8       P       1    DPC      P8       8         1.7  30.973 ; qtot : 1.63
     9      OM       1    DPC      O9       9        -0.8 15.999 ; qtot: 0.995
    10      OM       1    DPC     O10      10        -0.8 15.999 ; qtot: 0.36
    11      OA       1    DPC     O11      11        -0.7  15.999 ; qtot: 0.0
    12     CH2       1    DPC     C12      12         0.0   14.027  ; qtot: 0
    13     CH2       1    DPC     C13      13         0.0   14.027  ; qtot: 0
    14     CH2       1    DPC     C14      14         0.0   14.027  ; qtot: 0
    15     CH2       1    DPC     C15      15         0.0   14.027  ; qtot: 0
    16     CH2       1    DPC     C16      16         0.0   14.027  ; qtot: 0
    17     CH2       1    DPC     C17      17         0.0   14.027  ; qtot: 0
    18     CH2       1    DPC     C18      18         0.0   14.027  ; qtot: 0
    19     CH2       1    DPC     C19      19         0.0   14.027  ; qtot: 0
    20     CH2       1    DPC     C20      20         0.0   14.027  ; qtot: 0
    21     CH2       1    DPC     C21      21         0.0   14.027  ; qtot: 0
    22     CH2       1    DPC     C22      22         0.0   14.027  ; qtot: 0
    23     CH3       1    DPC     C23      23         0.0   15.035  ; qtot: 0


[ bonds ]
;  ai    aj  funct            c0            c1            c2            c3

    1     4     2    gb_21
    2     4     2    gb_21
    3     4     2    gb_21
    4     5     2    gb_21
    5     6     2    gb_27
    6     7     2    gb_18
    7     8     2    gb_28
    8     9     2    gb_24
    8    10     2    gb_24
    8    11     2    gb_28
   11    12     2    gb_18
   12    13     2    gb_27
   13    14     2    gb_27
   14    15     2    gb_27
   15    16     2    gb_27
   16    17     2    gb_27
   17    18     2    gb_27
   18    19     2    gb_27
   19    20     2    gb_27
   20    21     2    gb_27
   21    22     2    gb_27
   22    23     2    gb_27

[ pairs ]
;  ai    aj  funct
    1     6     1
    2     6     1
    3     6     1
    4     7     1
```

```
     5     8     1
     6     9     1
     6    10     1
     6    11     1
     7    12     1
     8    13     1
     9    12     1
    10    12     1
    11    14     1
;   12    15     1
;   13    16     1
;   14    17     1
;   15    18     1
;   16    19     1
;   17    20     1
;   18    21     1
;   19    22     1
;   20    23     1

[ angles ]
;  ai    aj    ak funct
     1     4     2     2    ga_13
     1     4     3     2    ga_13
     1     4     5     2    ga_13
     2     4     3     2    ga_13
     2     4     5     2    ga_13
     3     4     5     2    ga_13
     4     5     6     2    ga_15
     5     6     7     2    ga_15
     6     7     8     2    ga_26
     7     8     9     2    ga_14
     7     8    10     2    ga_14
     7     8    11     2    ga_5
     9     8    10     2    ga_29
    10     8    11     1    ga_14
     8    11    12     1    ga_26
    11    12    13     1    ga_15
    12    13    14     1    ga_15
    13    14    15     1    ga_15
    14    15    16     1    ga_15
    15    16    17     1    ga_15
    16    17    18     1    ga_15
    17    18    19     1    ga_15
    18    19    20     1    ga_15
    19    20    21     1    ga_15
    20    21    22     1    ga_15
    21    22    23     1    ga_15

[ dihedrals ]
;  ai    aj    ak    al funct
     1     4     5     6     1 gd_29
     4     5     6     7     1 gd_4
     4     5     6     7     1 gd_36
     5     6     7     8     1 gd_29
;
; define gd_20     0.000       5.09          2
; O-P-O- (dna, lipids)  1.2
     6     7     8     9     1 gd_20
     7     8    11    12     1 gd_27
     8    11    12    13     1 gd_29
    11    12    13    14     1 gd_1
    12    13    14    15     1 gd_34
    13    14    15    16     1 gd_34
    14    15    16    17     1 gd_34
    15    16    17    18     1 gd_34
    16    17    18    19     1 gd_34
    17    18    19    20     1 gd_34
    18    19    20    21     1 gd_34
    19    20    21    22     1 gd_34
    20    21    22    23     1 gd_34
```

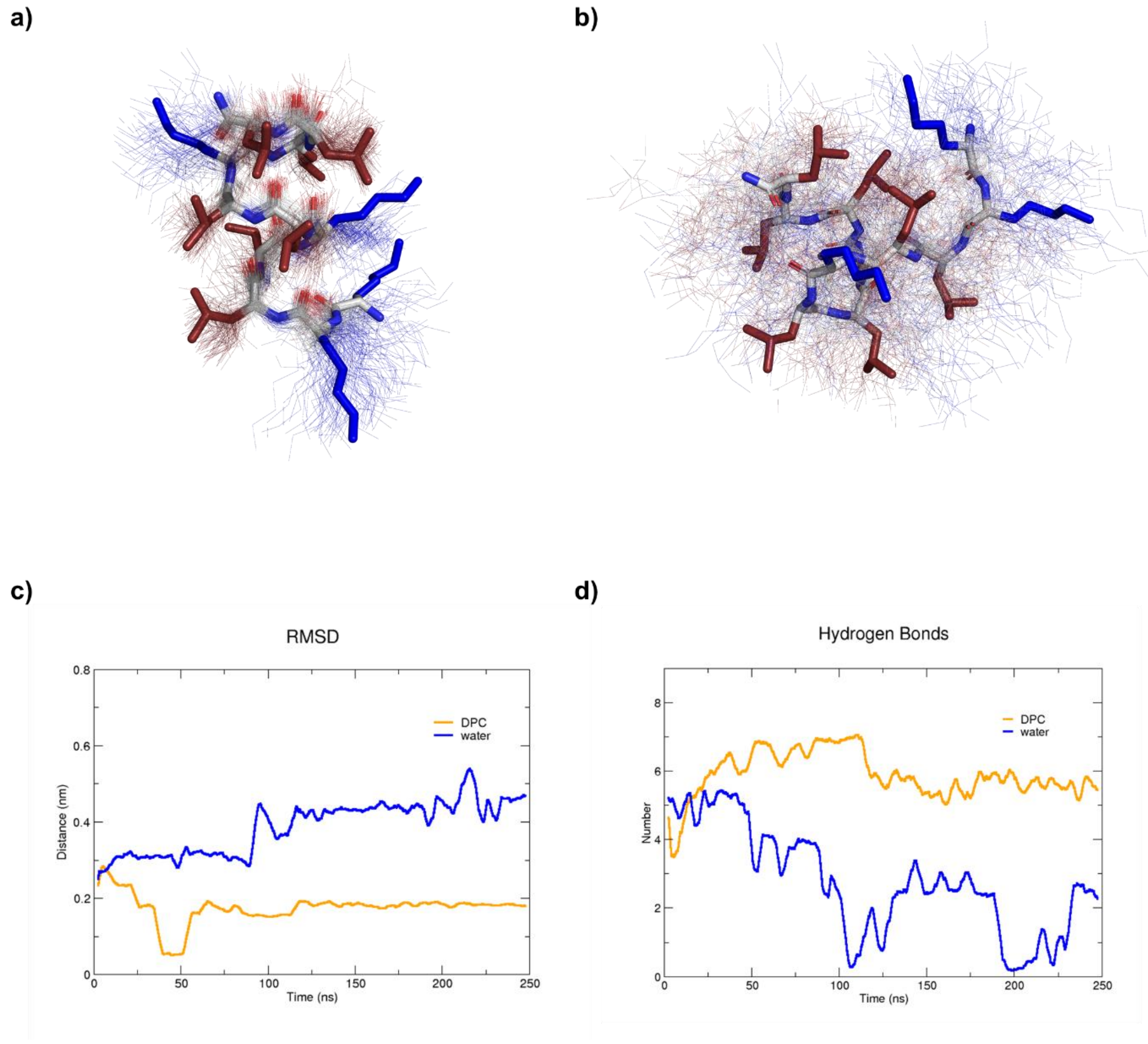

Figure S11: MD simulations of **dln69** with and without DPC micelle. **(a)** Average structure (stick model) in presence of DPC micelle over 100 structures sampled during the last 100 ns (thin lines). Hydrophobic side chains are colored in red and cationic side chains are colored in blue. **(b)** Same as (a) for run in water. **(c)** Comparison of root-mean square deviation of the peptide backbone relative to starting coordinates of the α-helix built in PyMol between run with DPC and run in water. **(d)** Comparison of the number of intramolecular backbone hydrogen bonds between run with DPC and run in water.

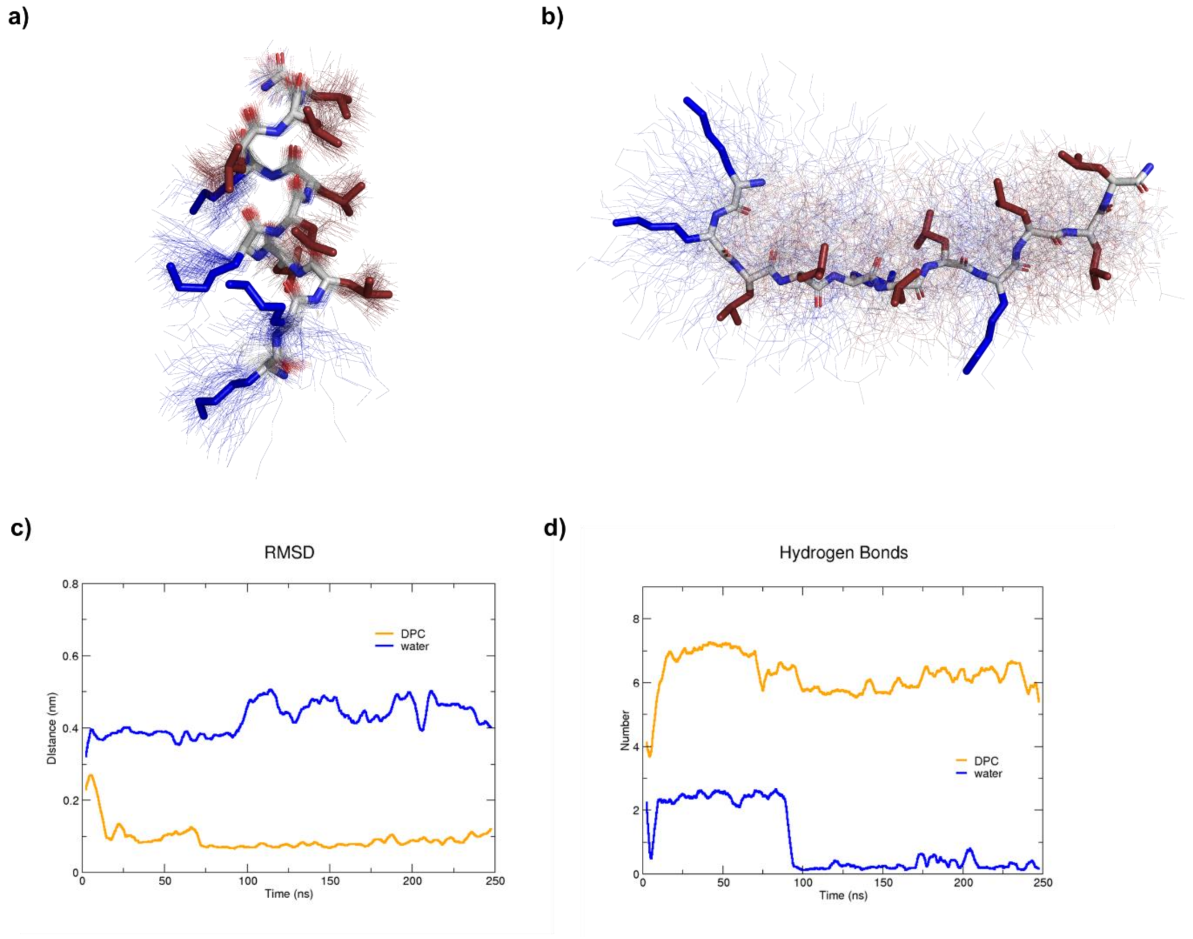

Figure S12: MD simulations of **HP1** with and without DPC micelle. **(a)** Average structure (stick model) in presence of DPC micelle over 100 structures sampled during the last 100 ns (thin lines). Hydrophobic side chains are colored in red and cationic side chains are colored in blue. **(b)** Same as (a) for run in water. **(c)** Comparison of root-mean square deviation of the peptide backbone relative to starting coordinates of the α-helix built in PyMol between run with DPC and run in water. **(d)** Comparison of the number of intramolecular backbone hydrogen bonds between run with DPC and run in water.

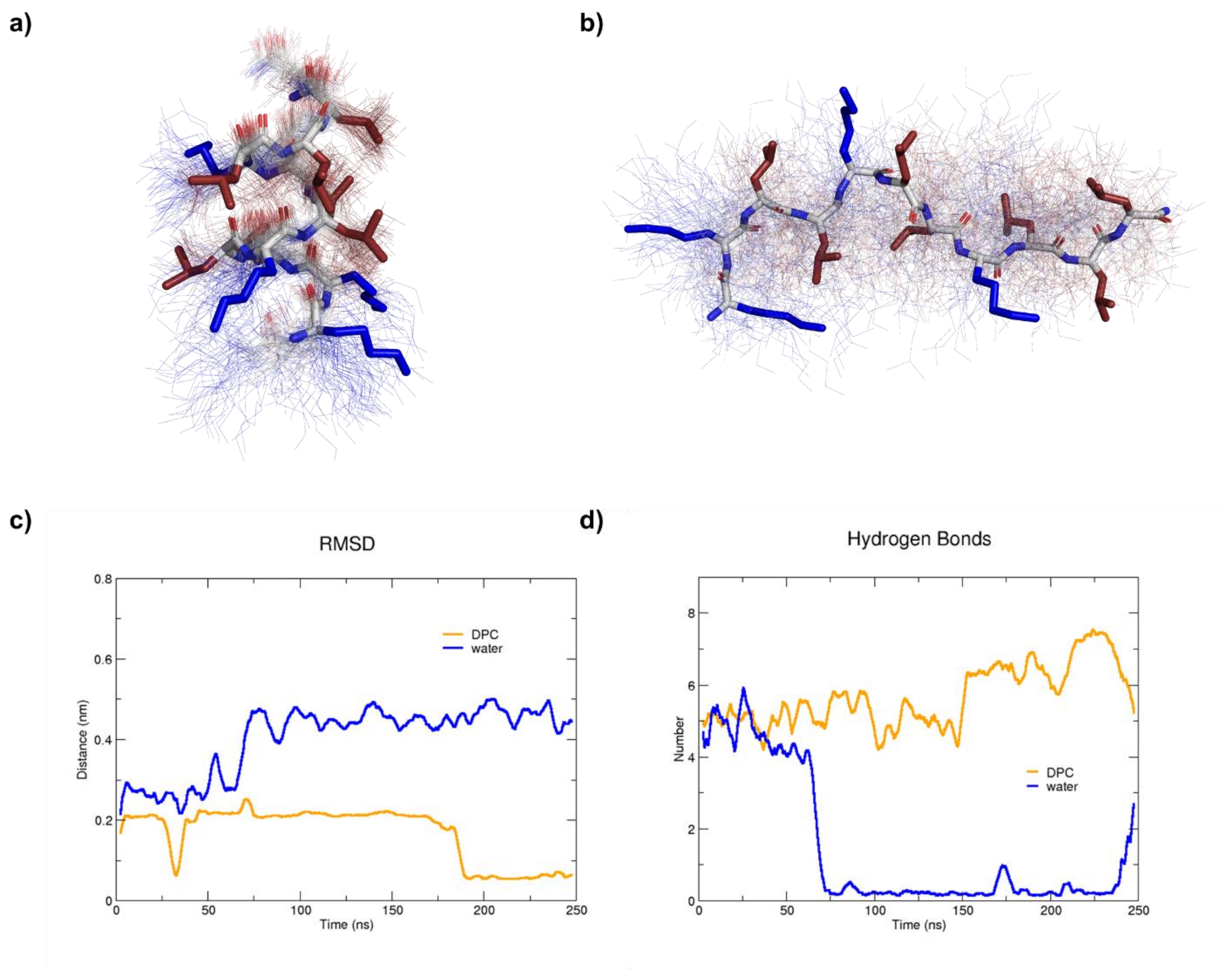

Figure S13: MD simulations of **HP2** with and without DPC micelle. **(a)** Average structure (stick model) in presence of DPC micelle over 100 structures sampled during the last 100 ns (thin lines). Hydrophobic side chains are colored in red and cationic side chains are colored in blue. **(b)** Same as (a) for run in water. **(c)** Comparison of root-mean square deviation of the peptide backbone relative to starting coordinates of the α-helix built in PyMol between run with DPC and run in water. **(d)** Comparison of the number of intramolecular backbone hydrogen bonds between run with DPC and run in water.

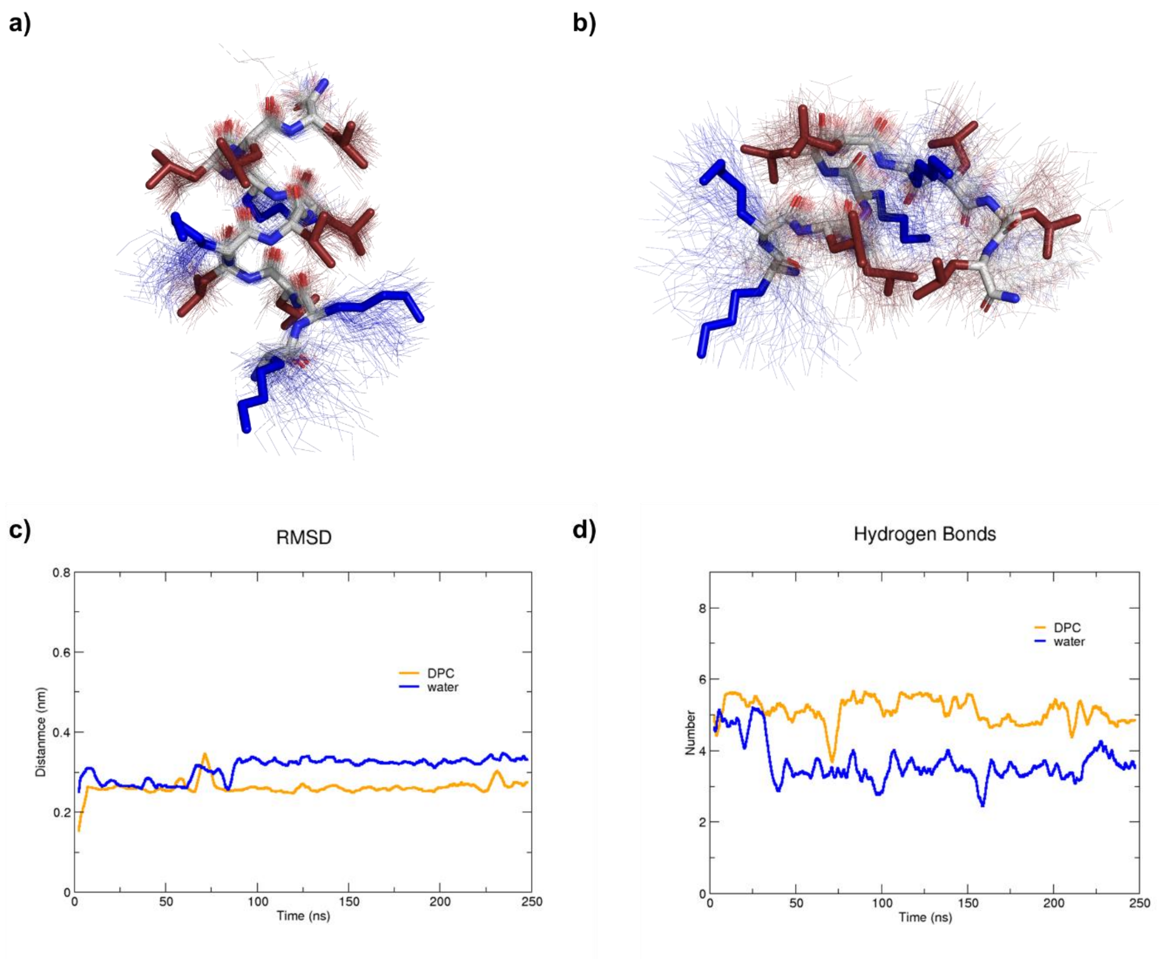

Figure S14: MD simulations of **HP3** with and without DPC micelle. **(a)** Average structure (stick model) in presence of DPC micelle over 100 structures sampled during the last 100 ns (thin lines). Hydrophobic side chains are colored in red and cationic side chains are colored in blue. **(b)** Same as (a) for run in water. **(c)** Comparison of root-mean square deviation of the peptide backbone relative to starting coordinates of the α-helix built in PyMol between run with DPC and run in water. **(d)** Comparison of the number of intramolecular backbone hydrogen bonds between run with DPC and run in water.

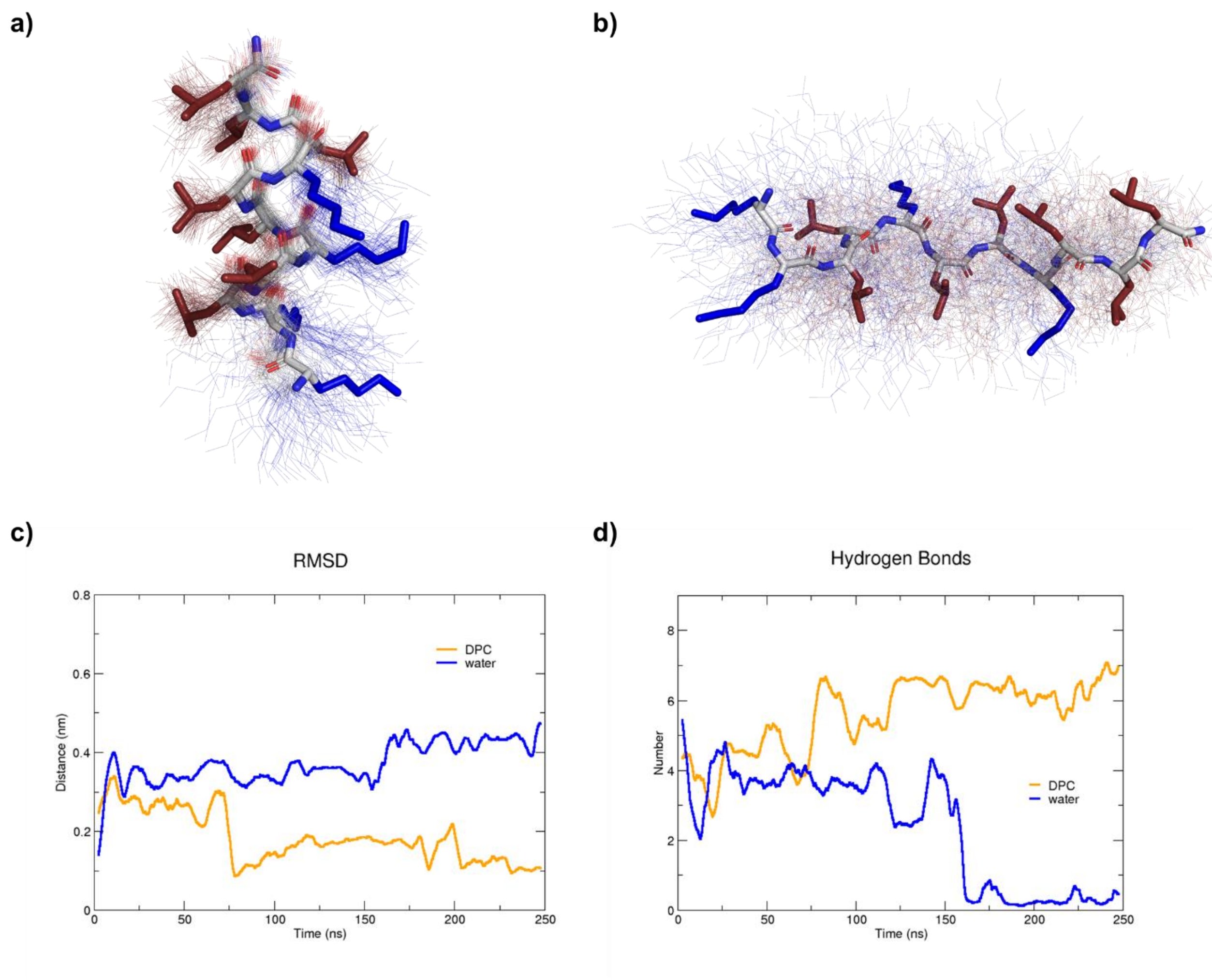

Figure S15: MD simulations of **HP4** with and without DPC micelle. **(a)** Average structure (stick model) in presence of DPC micelle over 100 structures sampled during the last 100 ns (thin lines). Hydrophobic side chains are colored in red and cationic side chains are colored in blue. **(b)** Same as (a) for run in water. **(c)** Comparison of root-mean square deviation of the peptide backbone relative to starting coordinates of the α-helix built in PyMol between run with DPC and run in water. **(d)** Comparison of the number of intramolecular backbone hydrogen bonds between run with DPC and run in water.

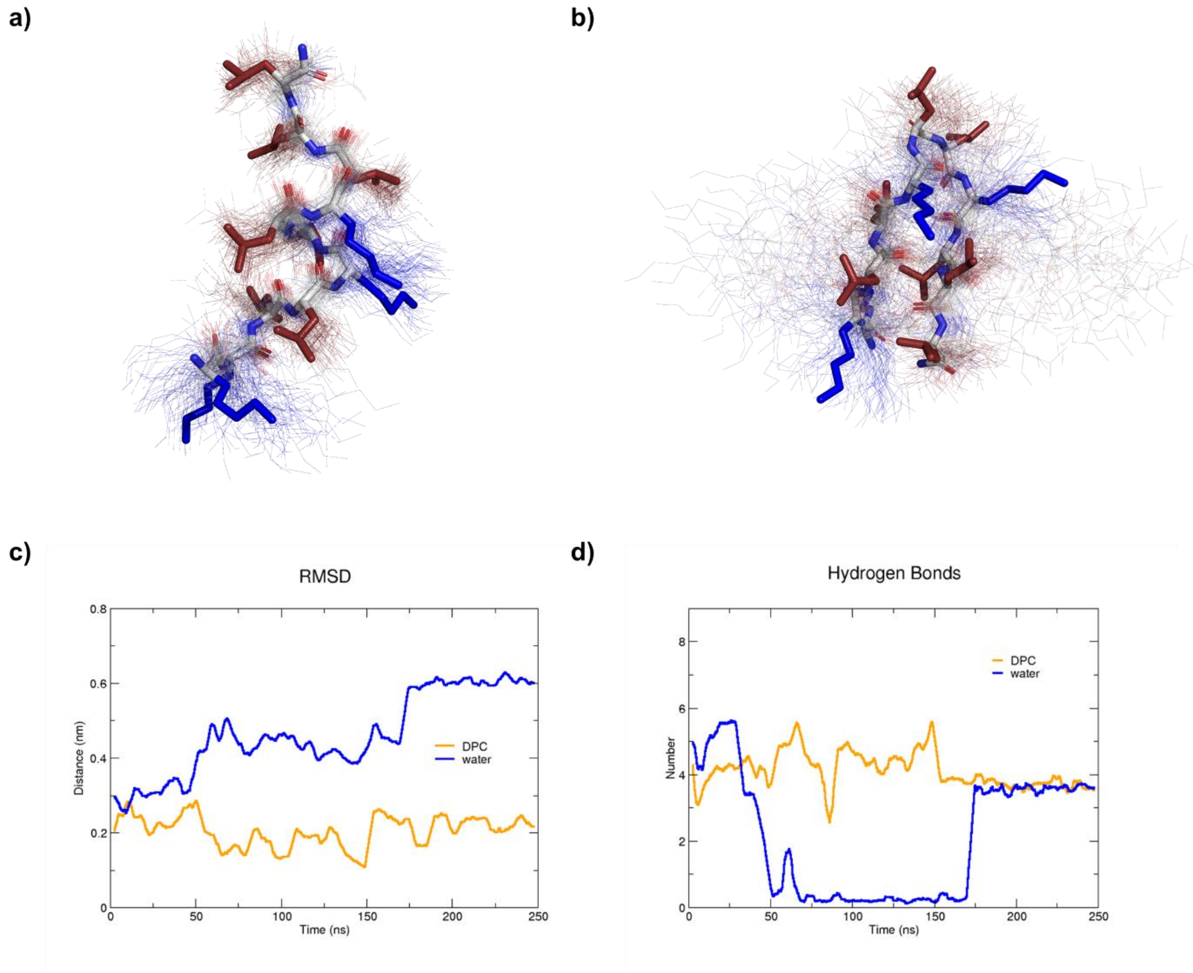

Figure S16: MD simulations of **HP7** with and without DPC micelle. **(a)** Average structure (stick model) in presence of DPC micelle over 100 structures sampled during the last 100 ns (thin lines). Hydrophobic side chains are colored in red and cationic side chains are colored in blue. **(b)** Same as (a) for run in water. **(c)** Comparison of root-mean square deviation of the peptide backbone relative to starting coordinates of the α-helix built in PyMol between run with DPC and run in water. **(d)** Comparison of the number of intramolecular backbone hydrogen bonds between run with DPC and run in water.

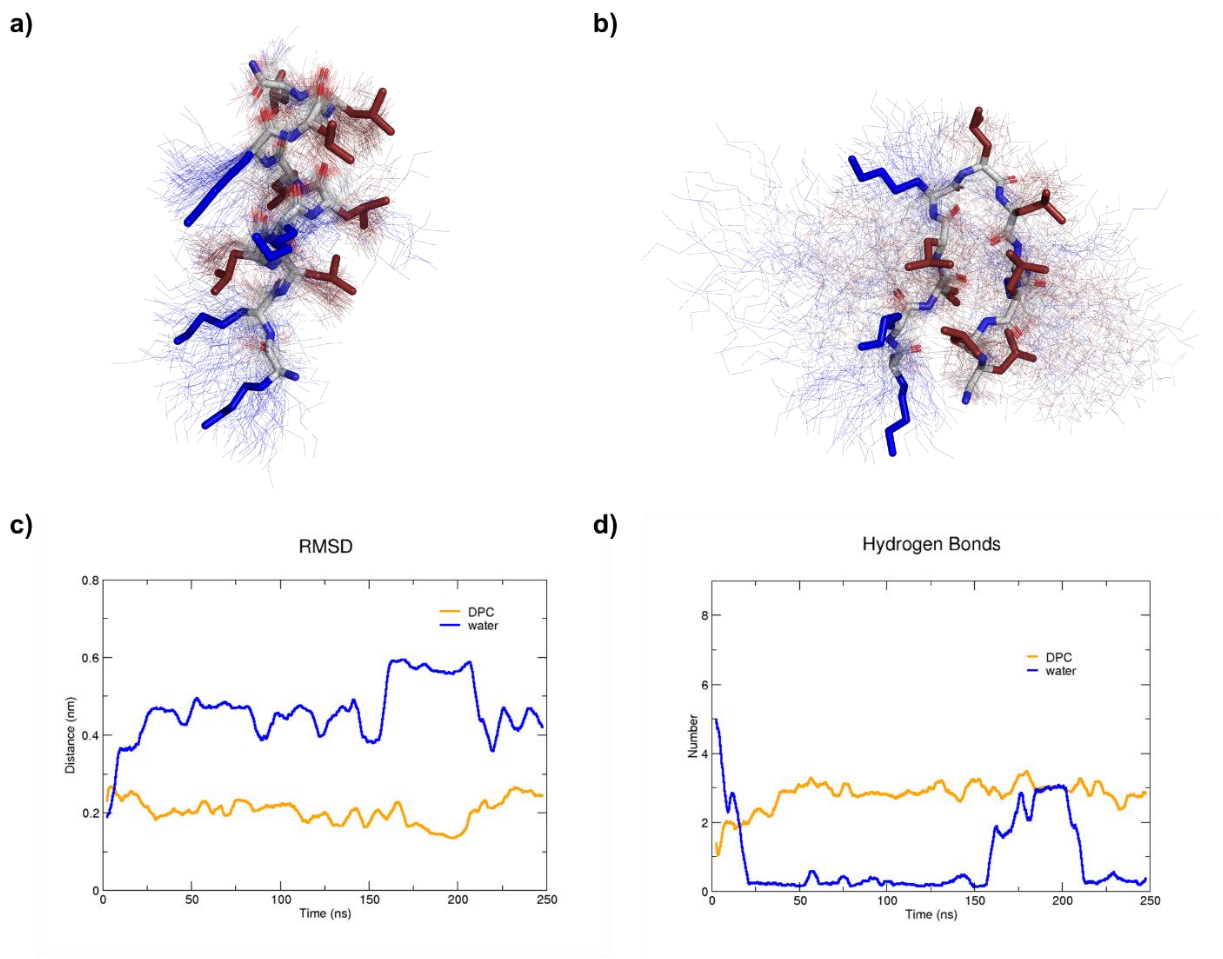

Figure S17: MD simulations of **HP8** with and without DPC micelle. **(a)** Average structure (stick model) in presence of DPC micelle over 100 structures sampled during the last 100 ns (thin lines). Hydrophobic side chains are colored in red and cationic side chains are colored in blue. **(b)** Same as (a) for run in water. **(c)** Comparison of root-mean square deviation of the peptide backbone relative to starting coordinates of the α-helix built in PyMol between run with DPC and run in water. **(d)** Comparison of the number of intramolecular backbone hydrogen bonds between run with DPC and run in water.

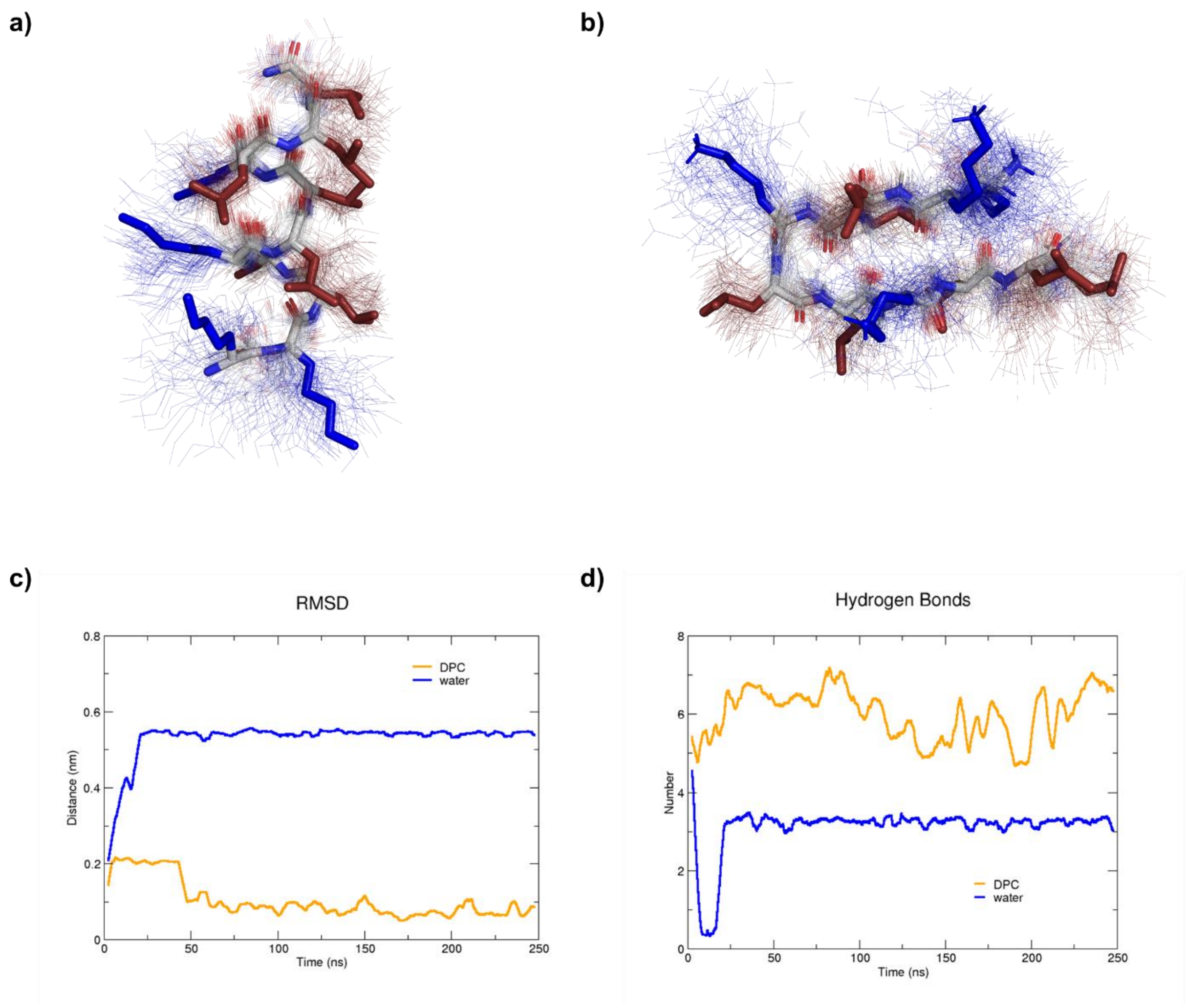

Figure S18: MD simulations of **HP10** with and without DPC micelle. **(a)** Average structure (stick model) in presence of DPC micelle over 100 structures sampled during the last 100 ns (thin lines). Hydrophobic side chains are colored in red and cationic side chains are colored in blue. **(b)** Same as (a) for run in water. **(c)** Comparison of root-mean square deviation of the peptide backbone relative to starting coordinates of the α-helix built in PyMol between run with DPC and run in water. **(d)** Comparison of the number of intramolecular backbone hydrogen bonds between run with DPC and run in water.

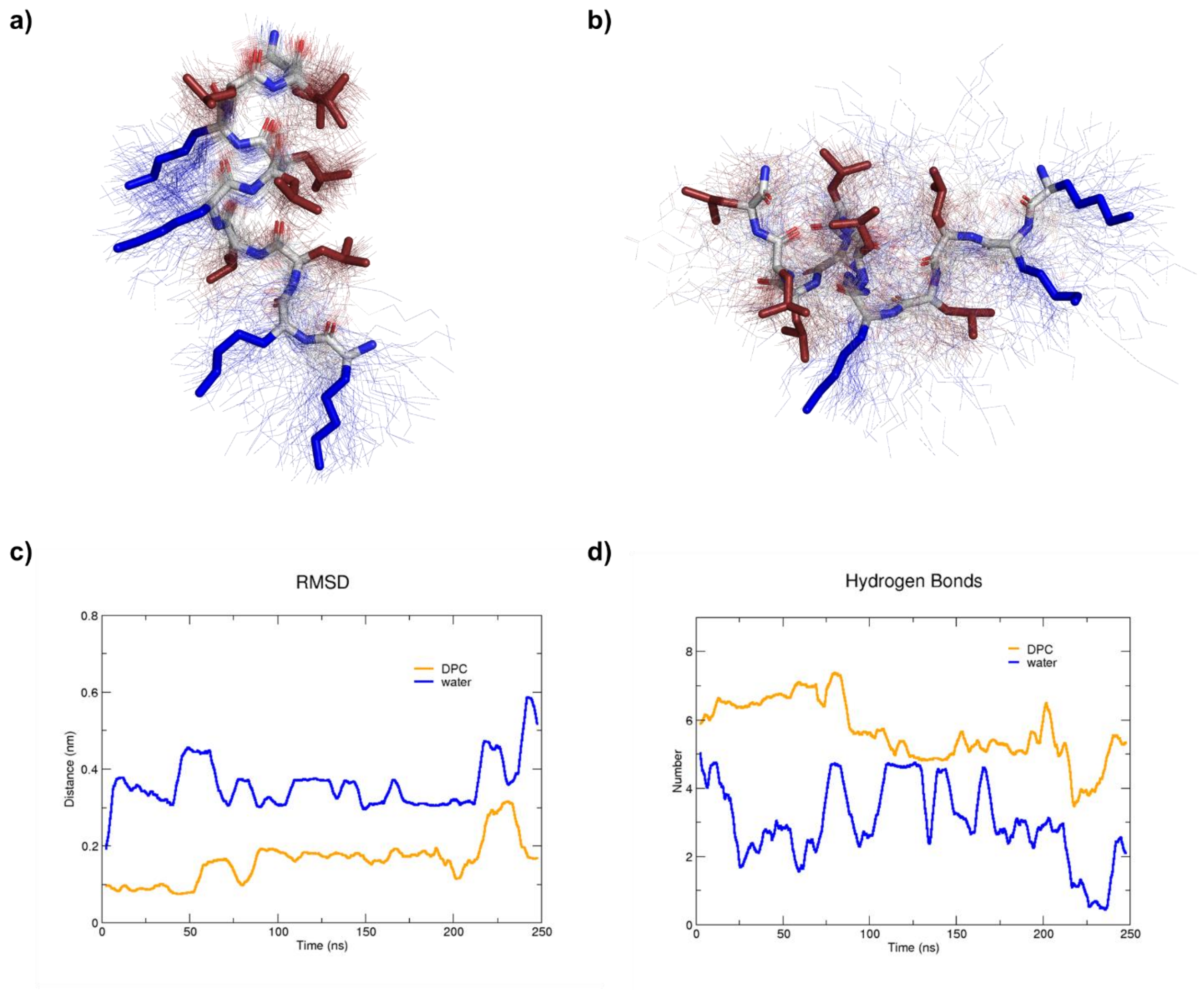

Figure S19: MD simulations of **HP11** with and without DPC micelle. **(a)** Average structure (stick model) in presence of DPC micelle over 100 structures sampled during the last 100 ns (thin lines). Hydrophobic side chains are colored in red and cationic side chains are colored in blue. **(b)** Same as (a) for run in water. **(c)** Comparison of root-mean square deviation of the peptide backbone relative to starting coordinates of the α-helix built in PyMol between run with DPC and run in water. **(d)** Comparison of the number of intramolecular backbone hydrogen bonds between run with DPC and run in water.

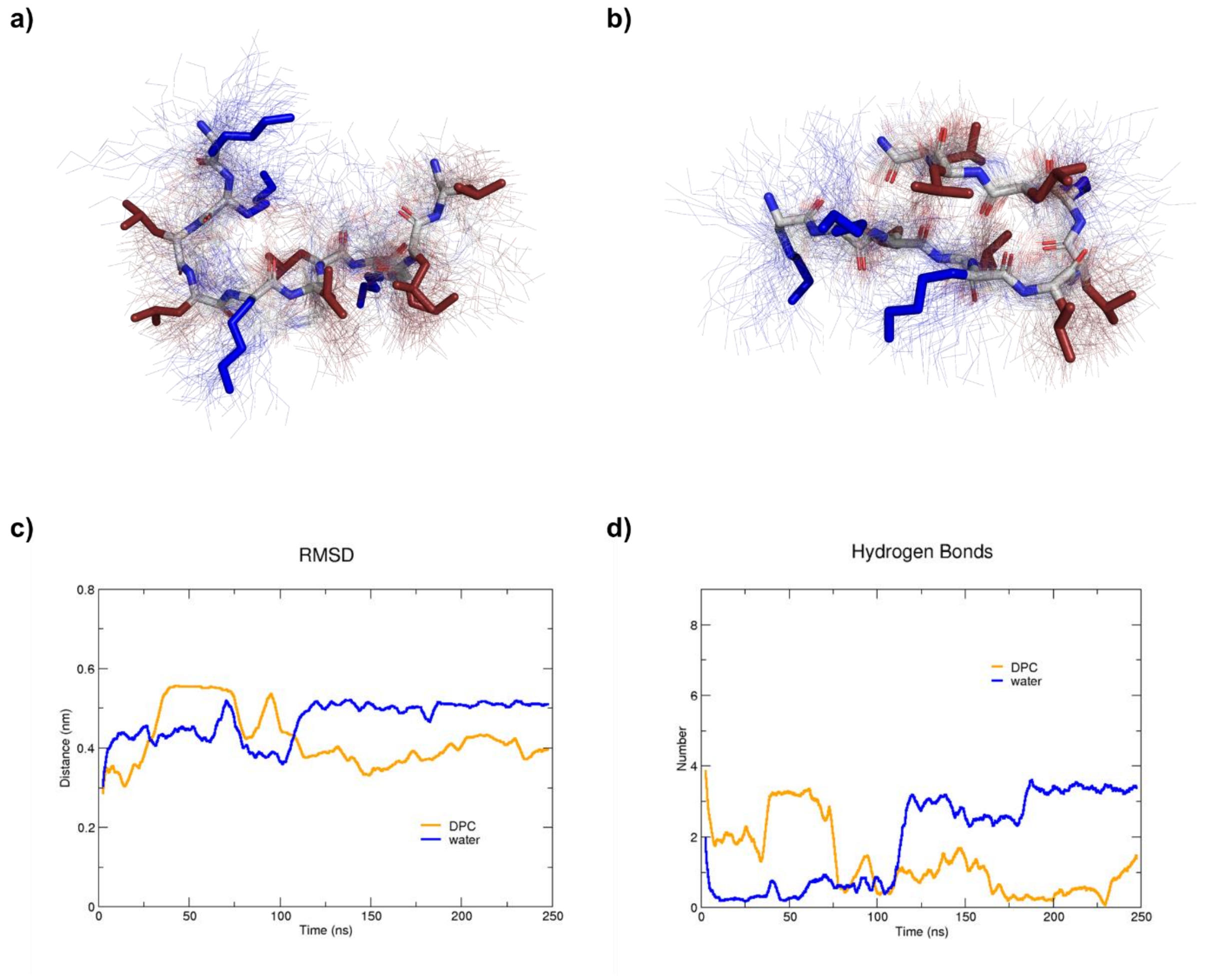

Figure S20: MD simulations of **HP16** with and without DPC micelle. **(a)** Average structure (stick model) in presence of DPC micelle over 100 structures sampled during the last 100 ns (thin lines). Hydrophobic side chains are colored in red and cationic side chains are colored in blue. **(b)** Same as (a) for run in water. **(c)** Comparison of root-mean square deviation of the peptide backbone relative to starting coordinates of the α-helix built in PyMol between run with DPC and run in water. **(d)** Comparison of the number of intramolecular backbone hydrogen bonds between run with DPC and run in water.

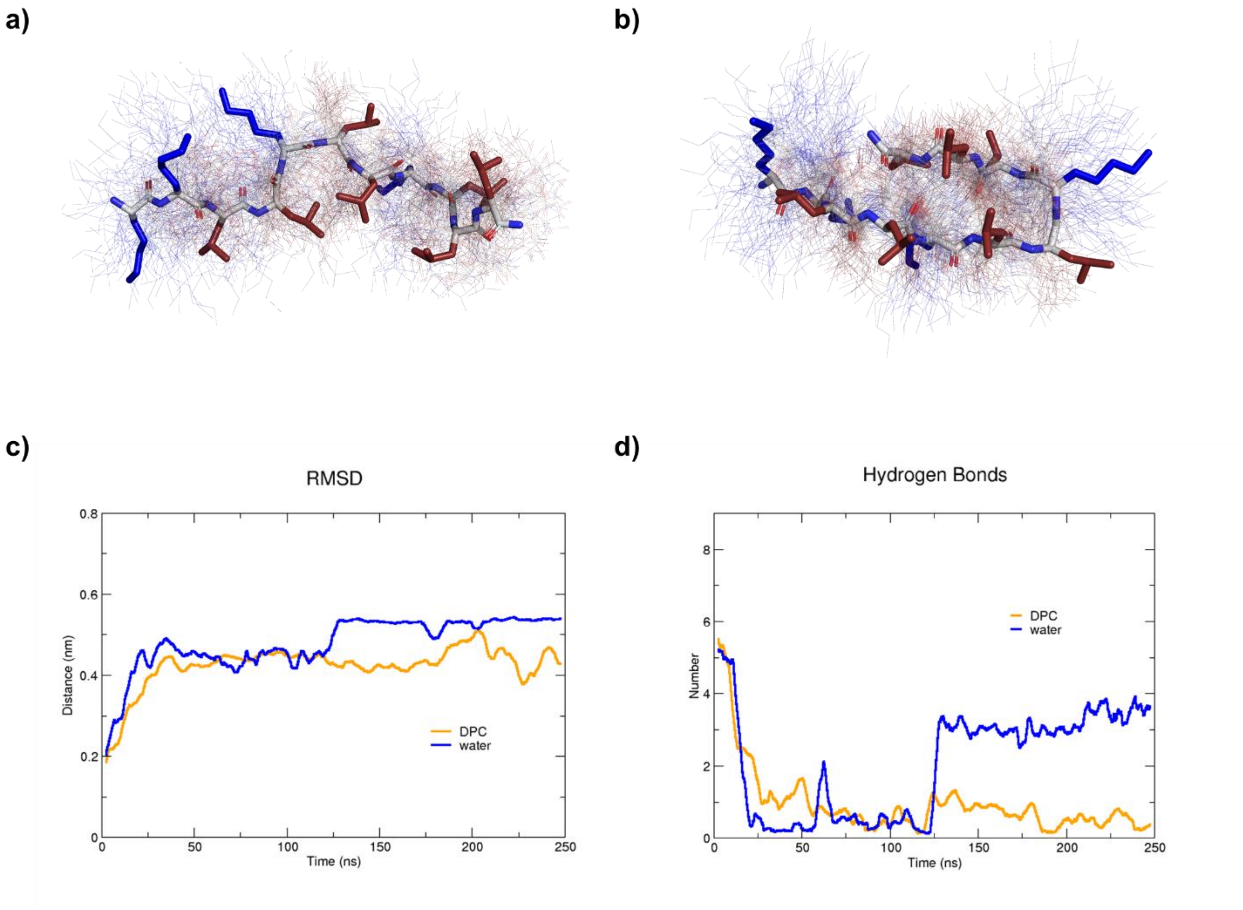

Figure S21: MD simulations of **HP29** with and without DPC micelle. **(a)** Average structure (stick model) in presence of DPC micelle over 100 structures sampled during the last 100 ns (thin lines). Hydrophobic side chains are colored in red and cationic side chains are colored in blue. **(b)** Same as (a) for run in water. **(c)** Comparison of root-mean square deviation of the peptide backbone relative to starting coordinates of the α-helix built in PyMol between run with DPC and run in water. **(d)** Comparison of the number of intramolecular backbone hydrogen bonds between run with DPC and run in water.

9. Statistical Analysis

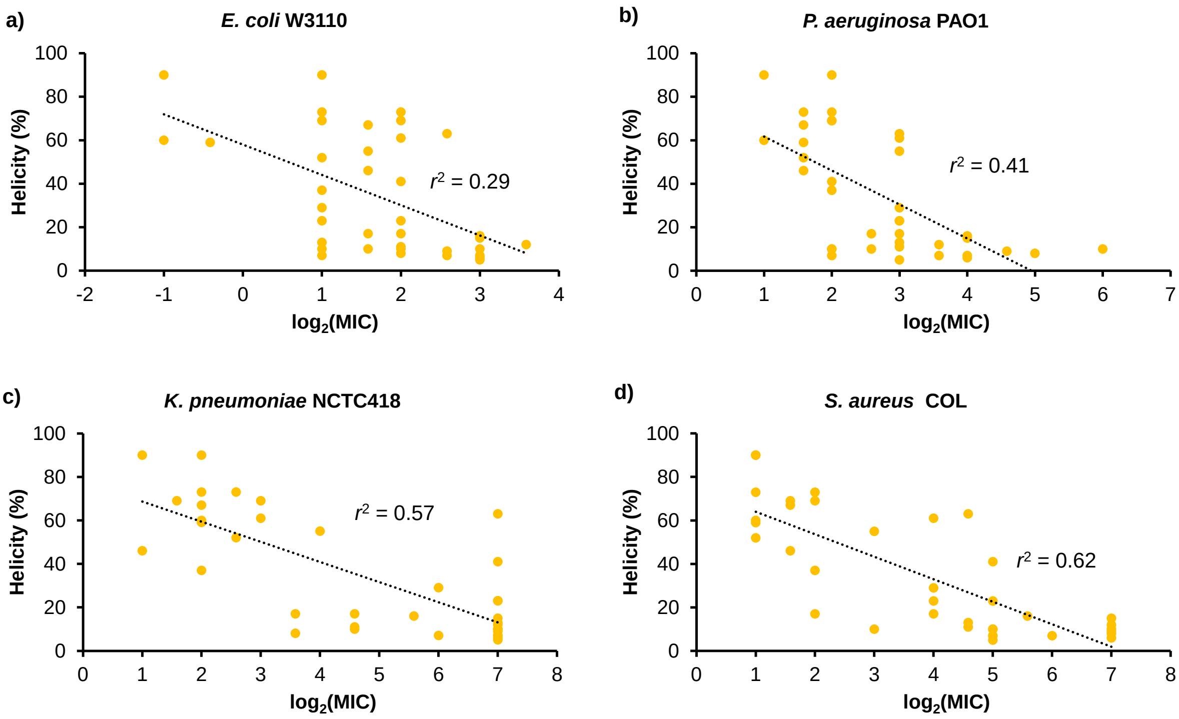

Figure S22: Scatter plot of % helicity in 5 mM DPC against $\log_2$(MIC) for (a) *E. coli*, (b) PAO1, (c) *K. pneumoniae* and (d) MRSA.

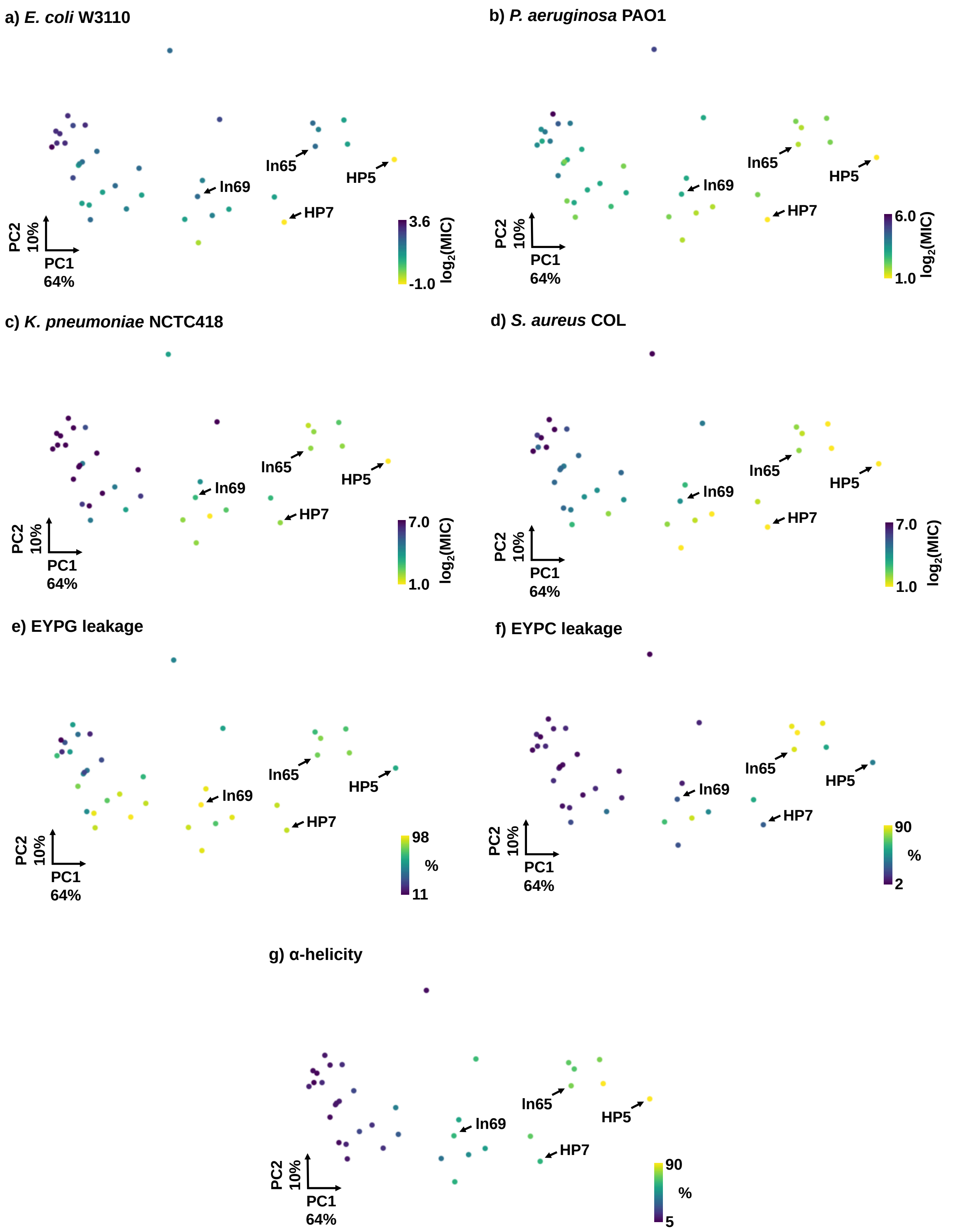

Figure S23: Principal Components Analysis visualization of dataset measured on **ln65** derivatives using Faerun.[3] Each point represents one compound and is colour coded depending on **(a)** activity on *E. coli* W3110, **(b)** activity on *P. aeruginosa* PAO1, **(c)** activity on *K. pneumoniae* NCTC418 **(d)** activity on *S. aureus* COL, **(e)** percentage of EYPG vesicles leakage, **(f)** percentage of EYPC vesicles leakage and **(g)** α-helicity in 5 mM DPC.

10. HPLC-MS and HRMS data

KKLLKLLKLLL (ln65) was obtained as white solid after preparative RP-HPLC (84.0 mg, 58.0%). Analytical RP-HPLC: t_R = 1.66 min (A/D 100:0 to 0:100 in 3.5 min, λ = 214 nm). MS (ESI+): $C_{66}H_{128}N_{16}O_{11}$ calc./obs. 1320.99/1320.99 Da [M].

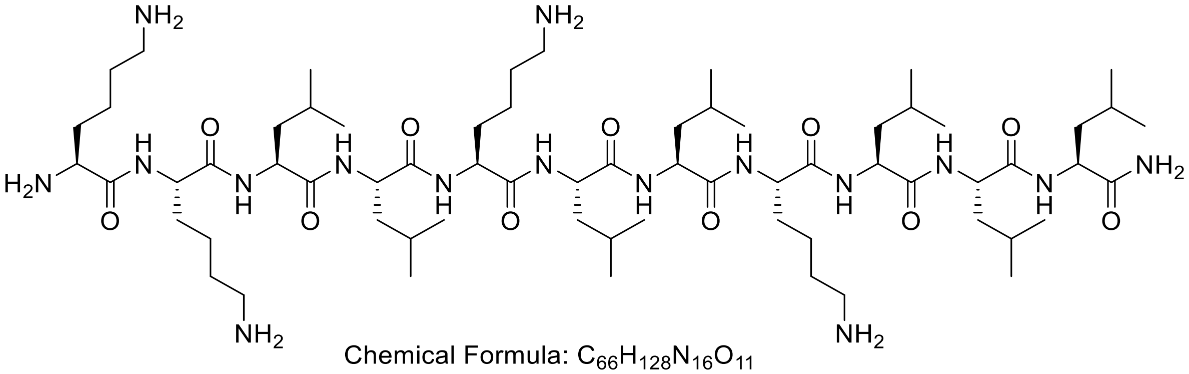

Chemical Formula: $C_{66}H_{128}N_{16}O_{11}$
Exact Mass: 1320.99
Molecular Weight: 1321.85

Analytical HPLC-MS data:

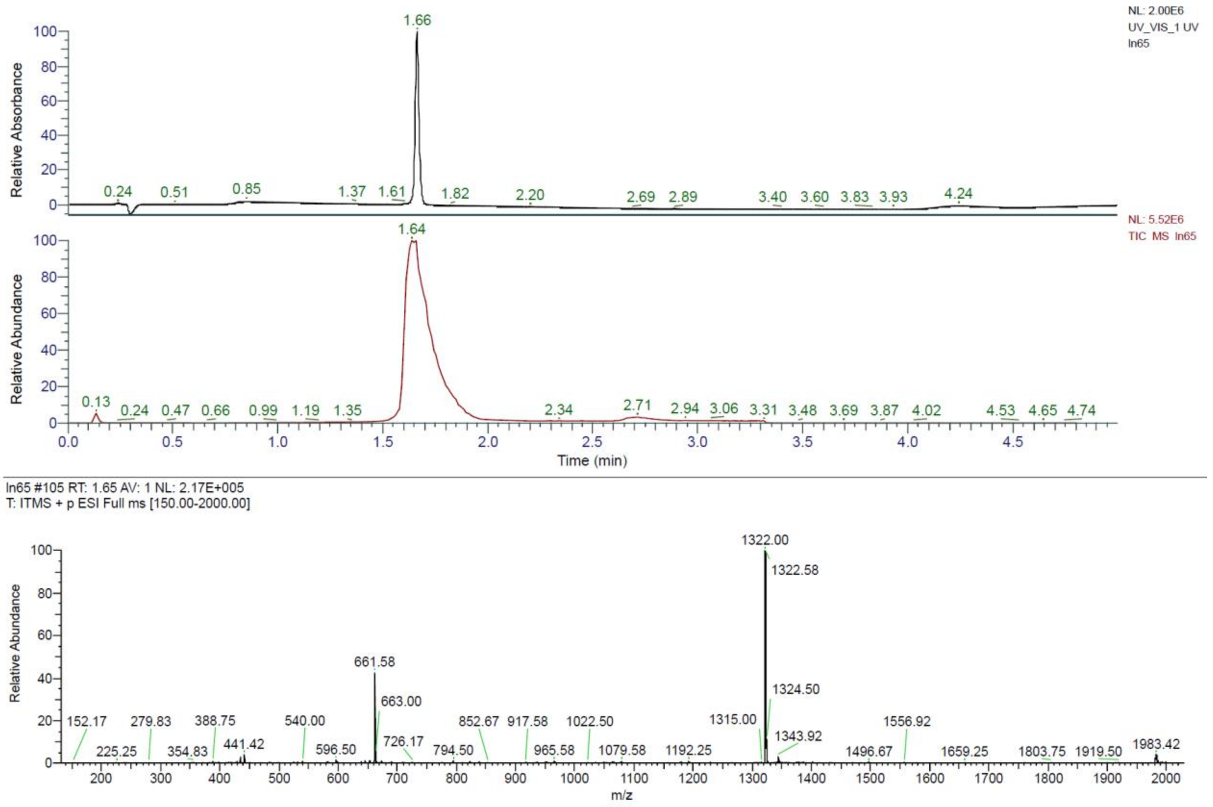

HRMS spectra:

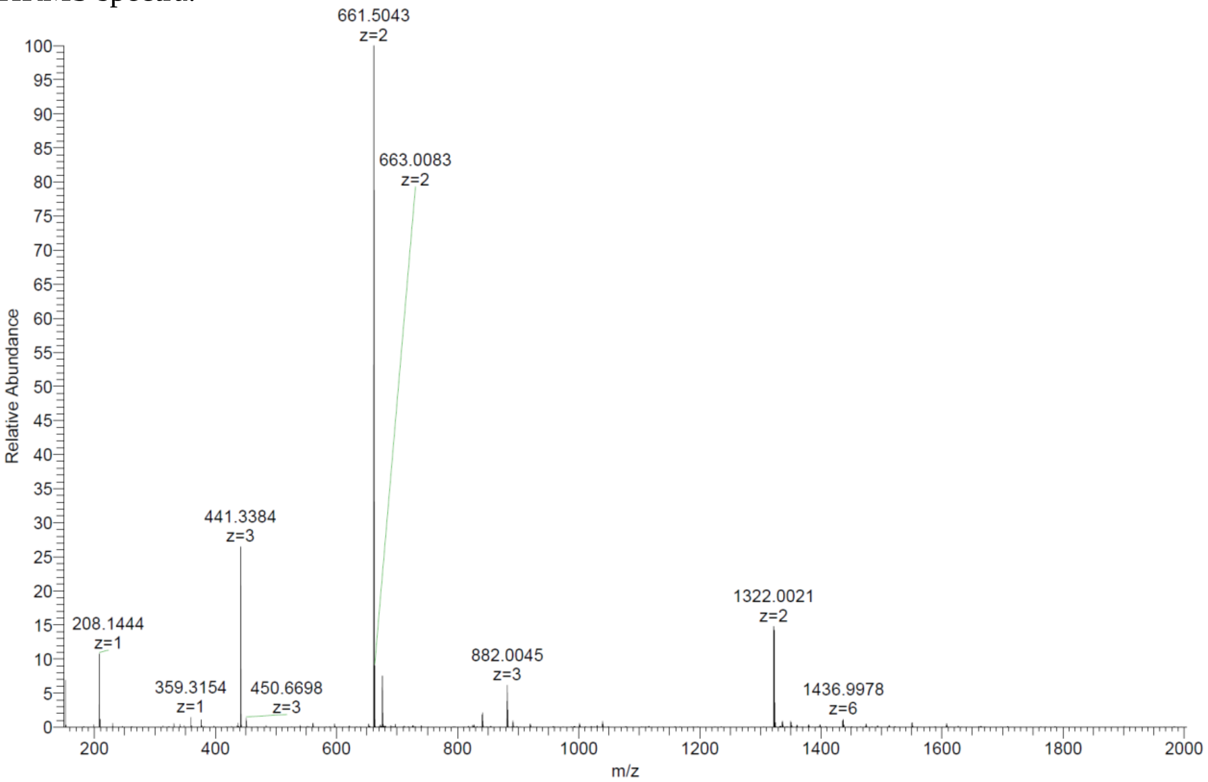

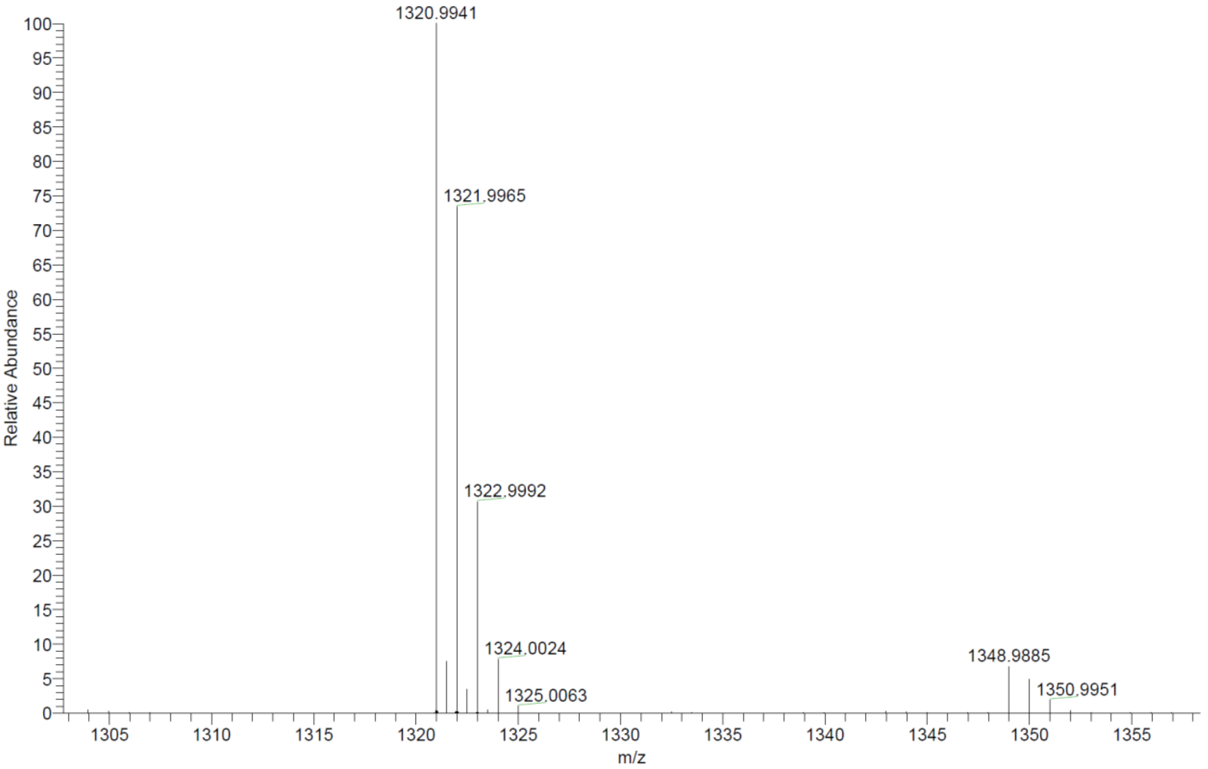

kkllkllklll (dln65) was obtained as white solid after preparative RP-HPLC (34.9 mg, 24.1%). Analytical RP-HPLC: t_R = 1.62 min (A/D 100:0 to 0:100 in 3.5 min, λ = 214 nm). MS (ESI+): $C_{66}H_{128}N_{16}O_{11}$ calc./obs. 1321.99/1322.00 Da $[M+H]^+$.

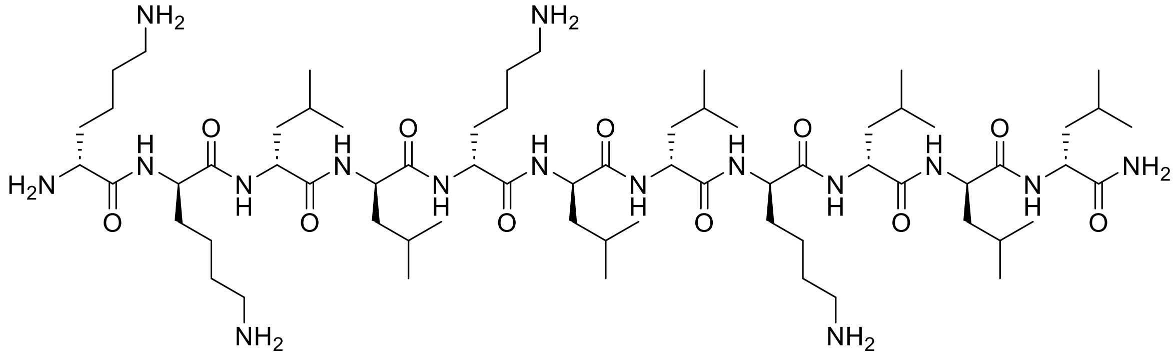

Analytical HPLC-MS data:

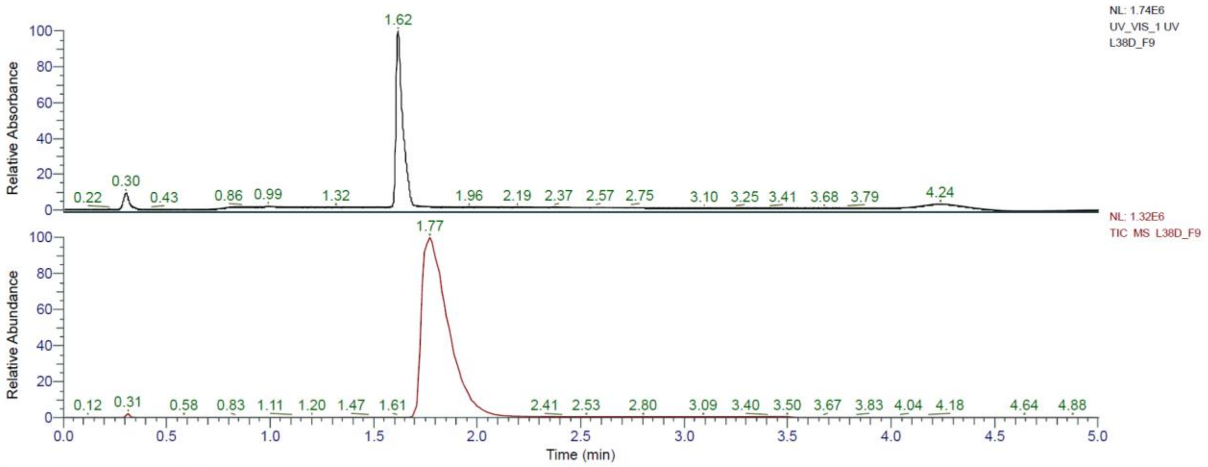

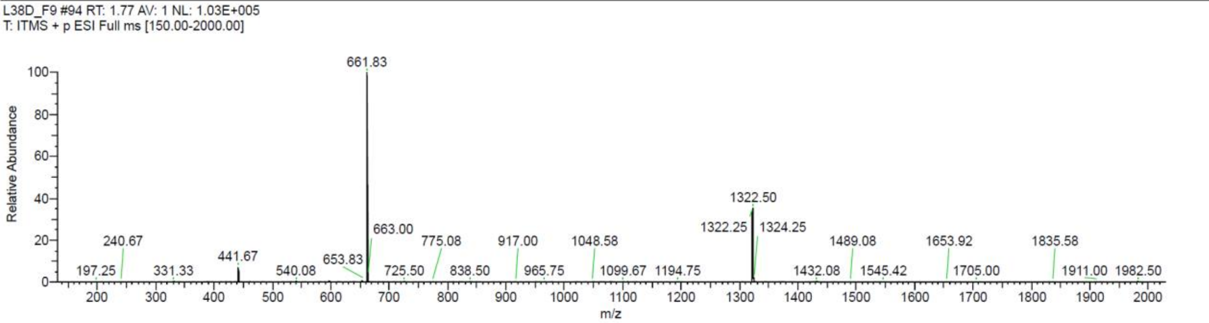

HRMS spectra:

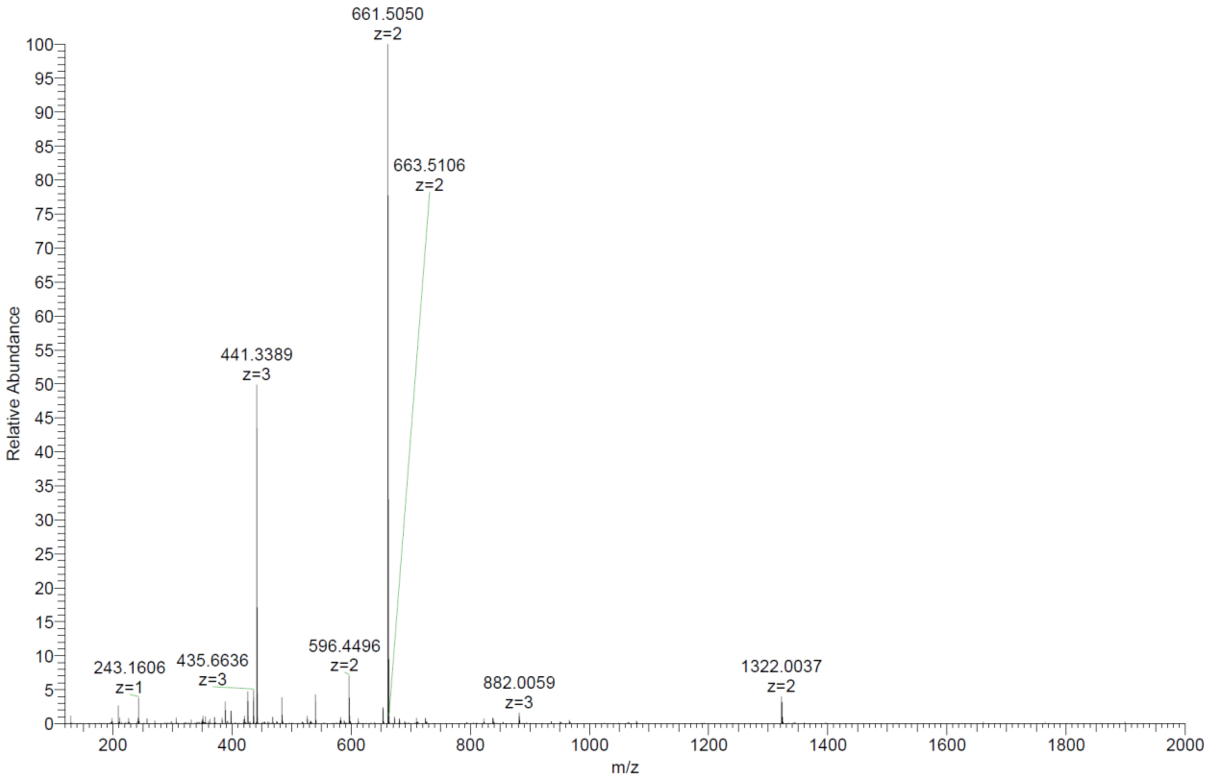

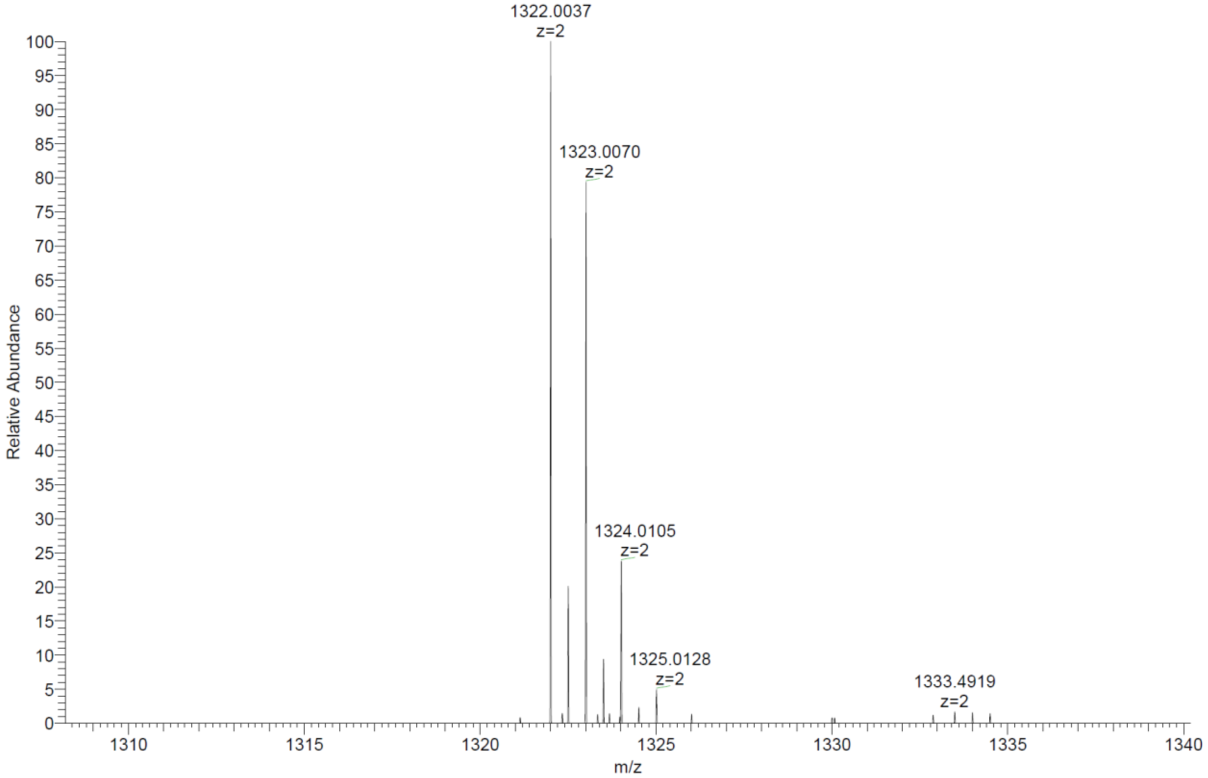

kkLLkLLkLLL (ln69) was obtained as white solid after preparative RP-HPLC (102.6 mg, 70.9%). Analytical RP-HPLC: t_R = 1.55 min (A/D 100:0 to 0:100 in 3.5 min, λ = 214 nm). MS (ESI+): $C_{66}H_{128}N_{16}O_{11}$ calc./obs. 1320.99/1320.99 Da [M].

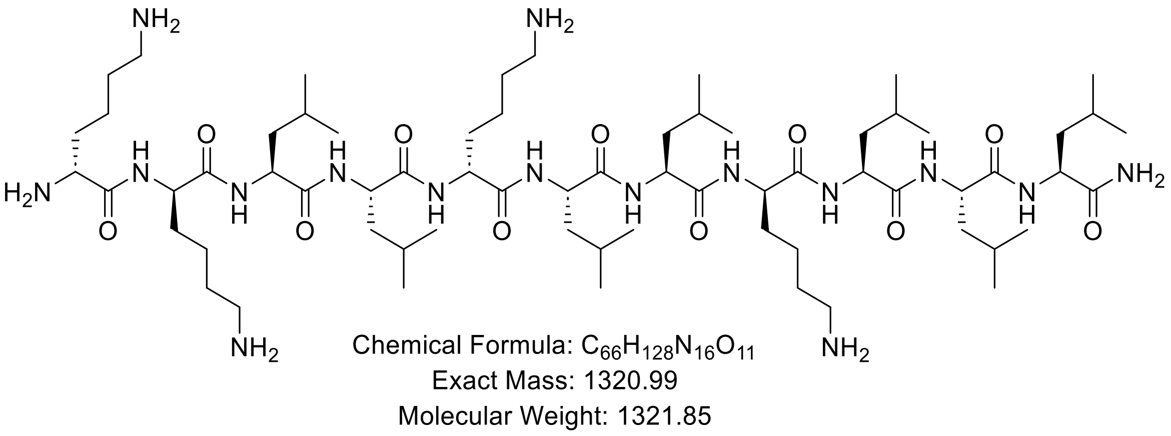

Analytical HPLC-MS data:

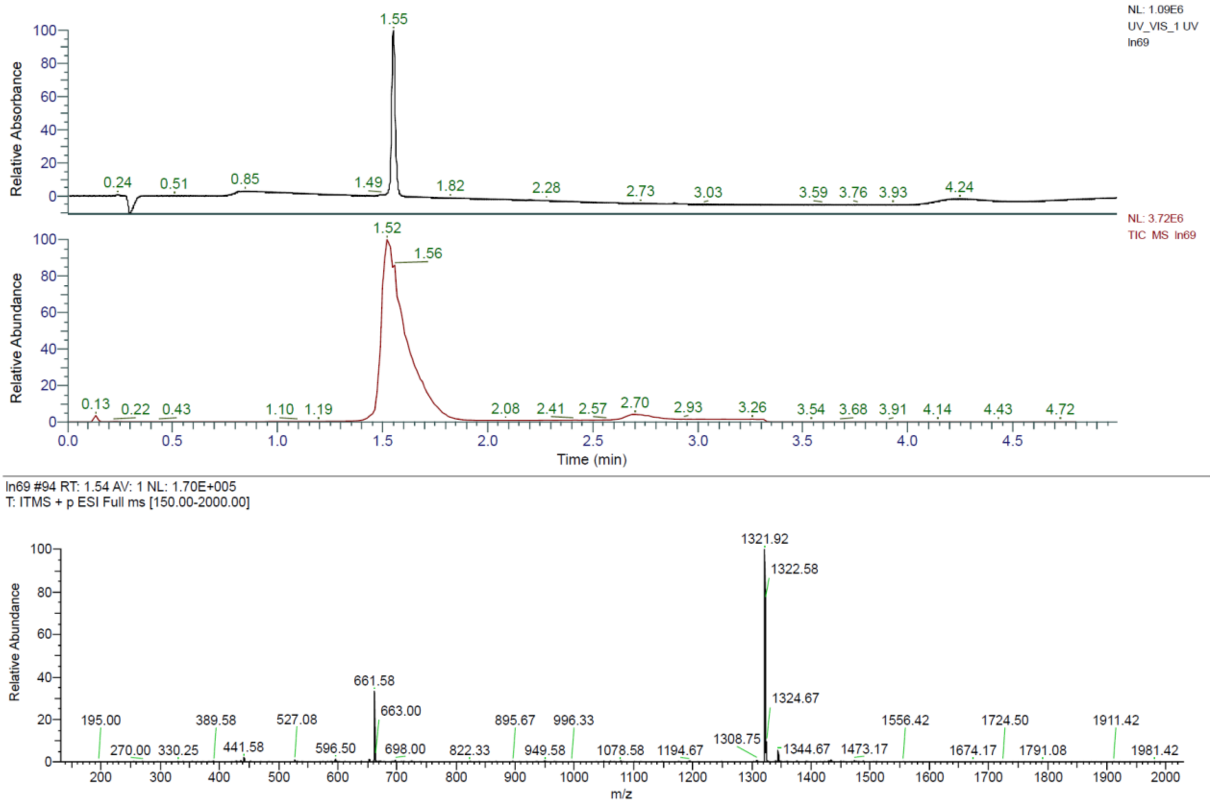

HRMS spectra:

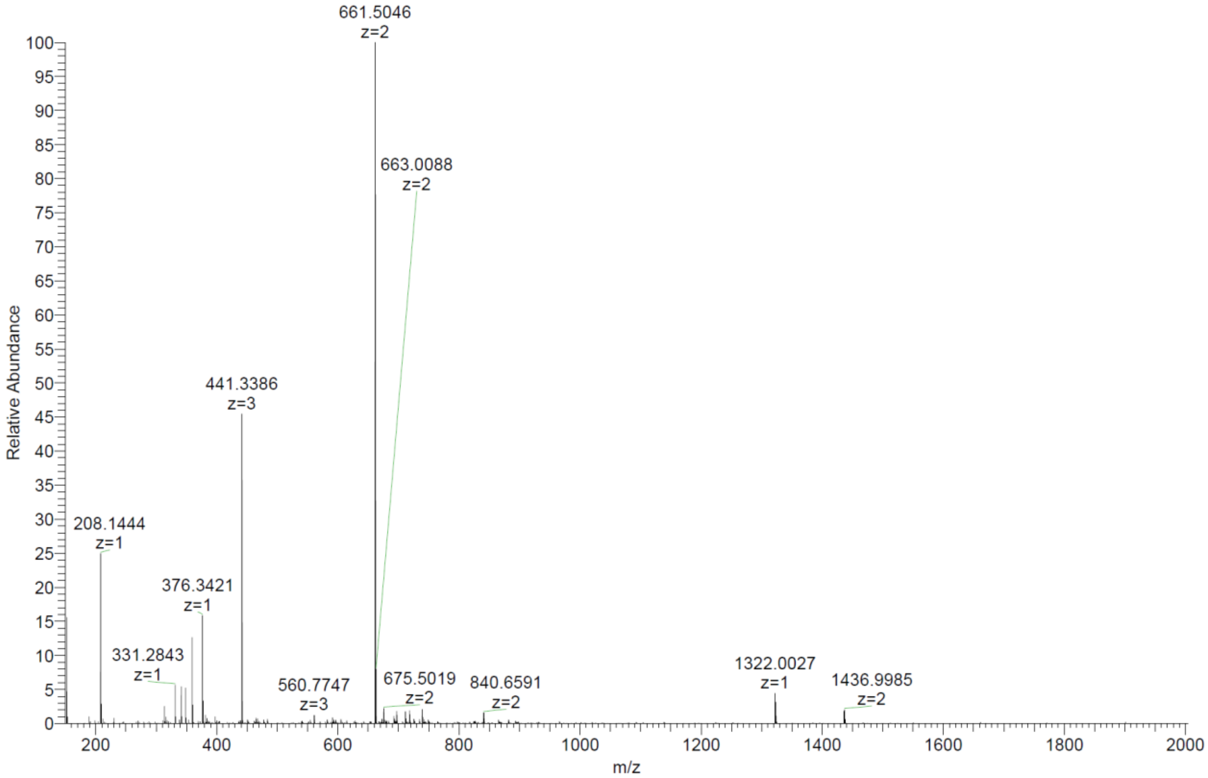

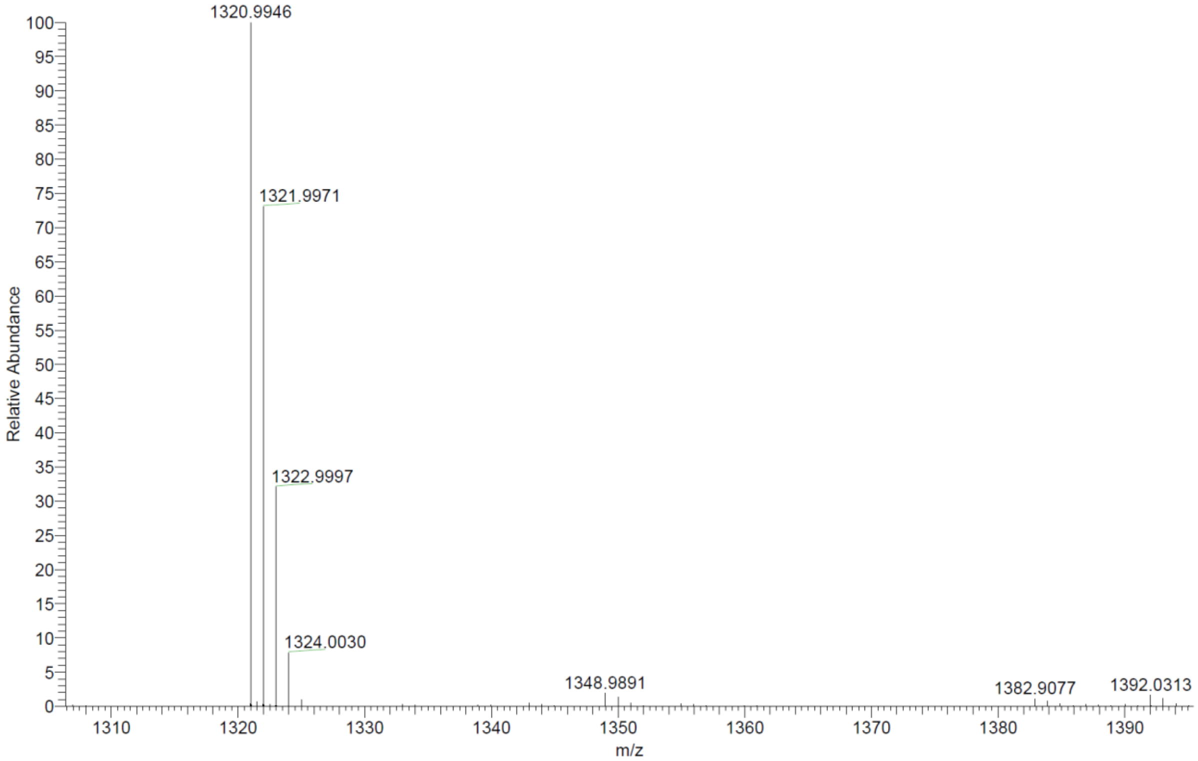

KKllKllKlll (dln69) was obtained as white solid after preparative RP-HPLC (69.7 mg, 44.6%). Analytical RP-HPLC: t_R = 1.52 min (A/D 100:0 to 0:100 in 3.5 min, λ = 214 nm). MS (ESI+): $C_{66}H_{128}N_{16}O_{11}$ calc./obs. 1321.99/1322.00 Da $[M+H]^+$.

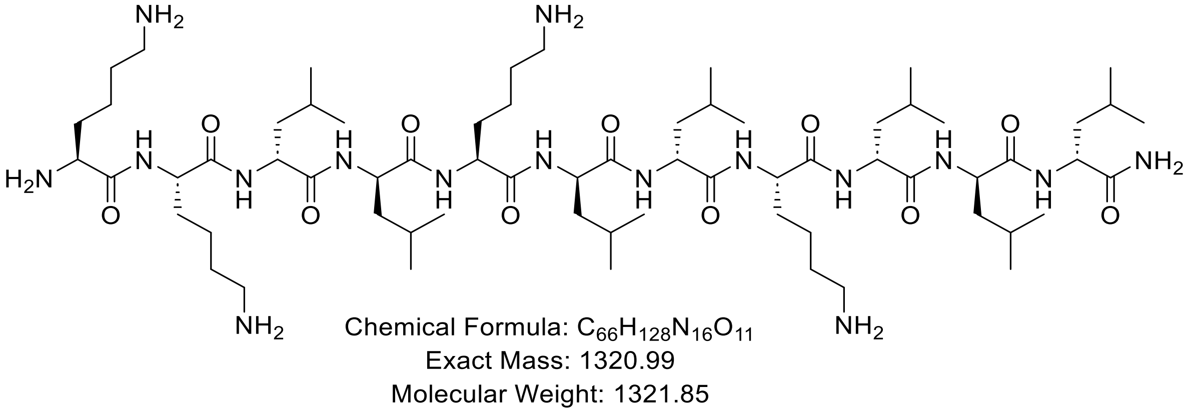

Analytical HPLC-MS data:

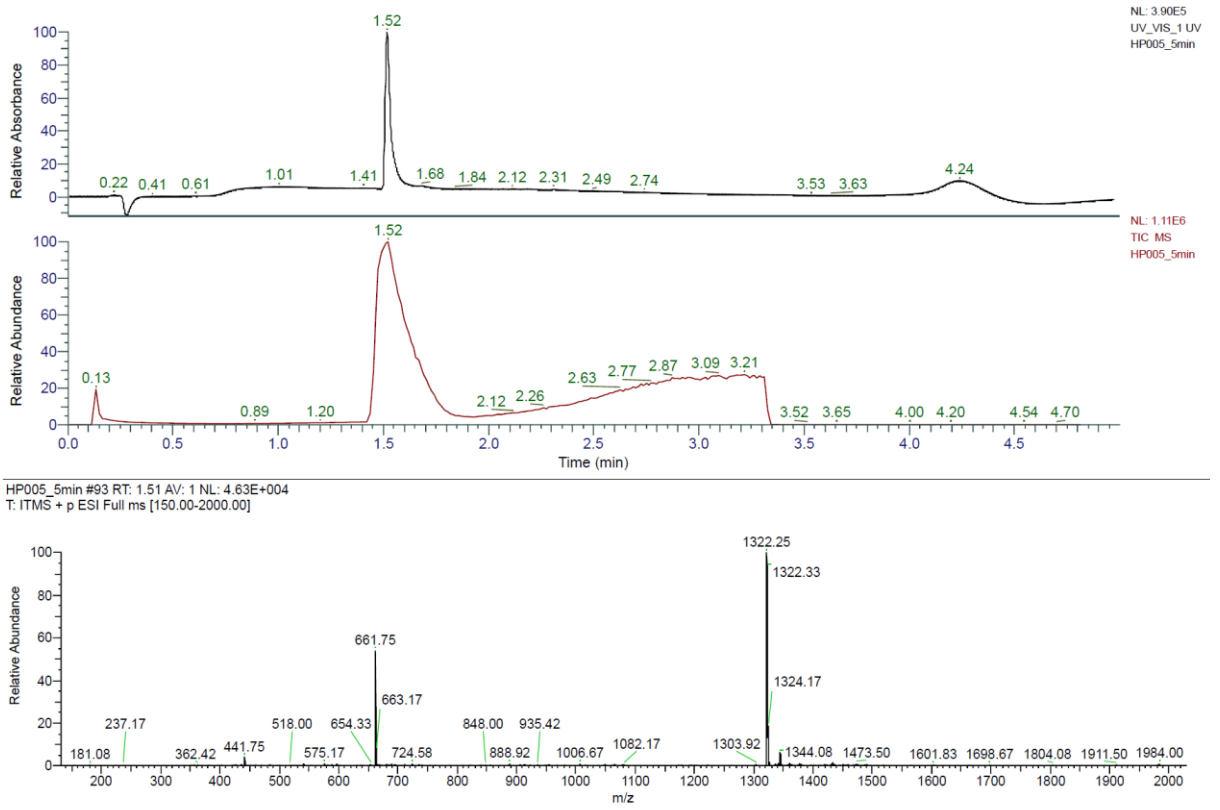

HRMS spectra:

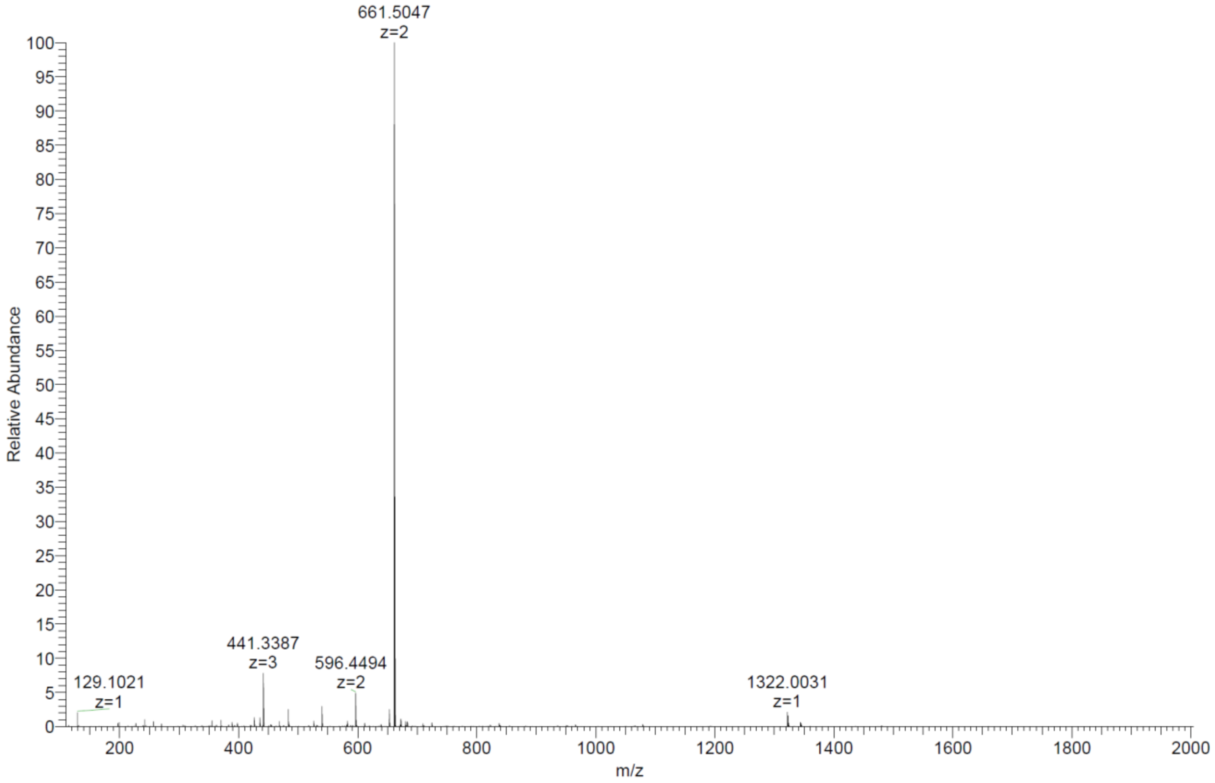
661.5047
z=2
441.3387
z=3
596.4494
z=2
129.1021
z=1
1322.0031
z=1
Relative Abundance
m/z

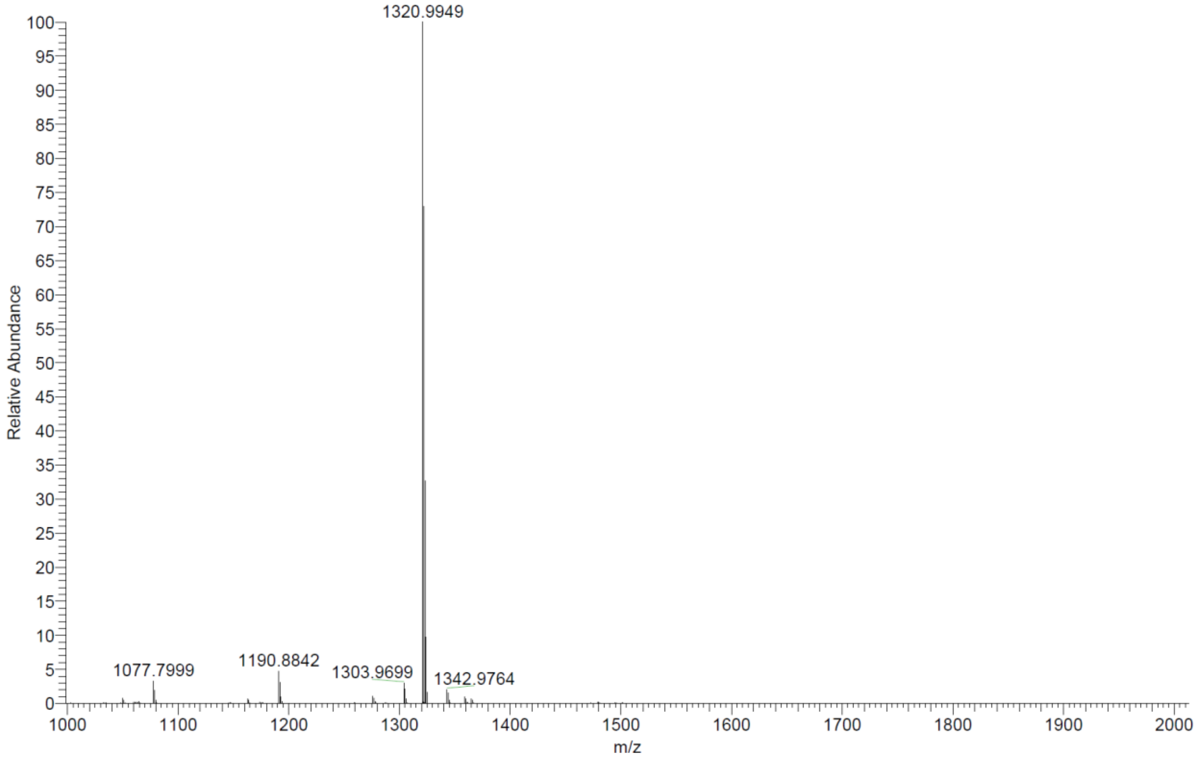
1320.9949
1077.7999
1190.8842
1303.9699
1342.9764
Relative Abundance
m/z

<u>KKLLKLLKLLL</u> (*sr*-ln65) was obtained as white solid after preparative RP-HPLC (64.6 mg, 41.3%). Analytical RP-HPLC: t_R = 1.58 min (A/D 100:0 to 0:100 in 3.5 min, λ = 214 nm). MS (ESI+): $C_{66}H_{128}N_{16}O_{11}$ calc./obs. 1320.99/1321.00 Da [M].

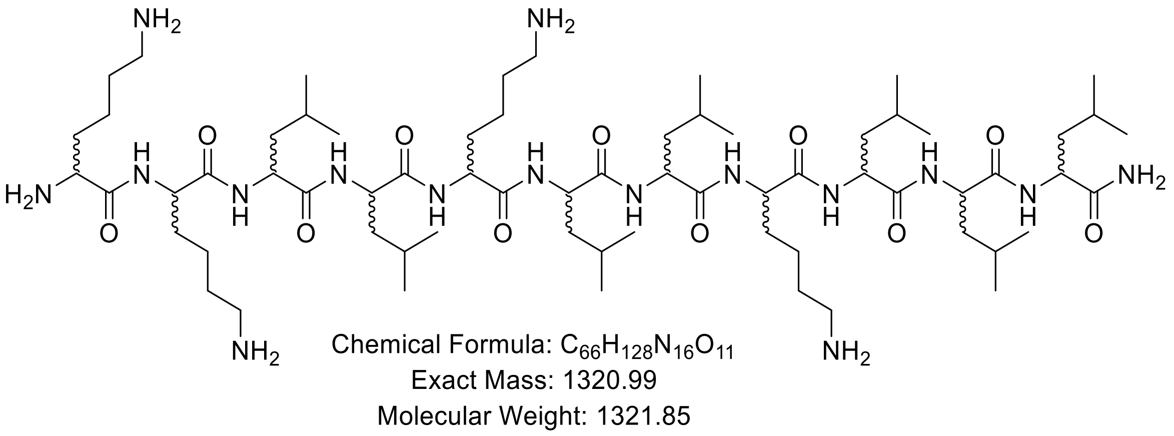

Analytical HPLC-MS data:

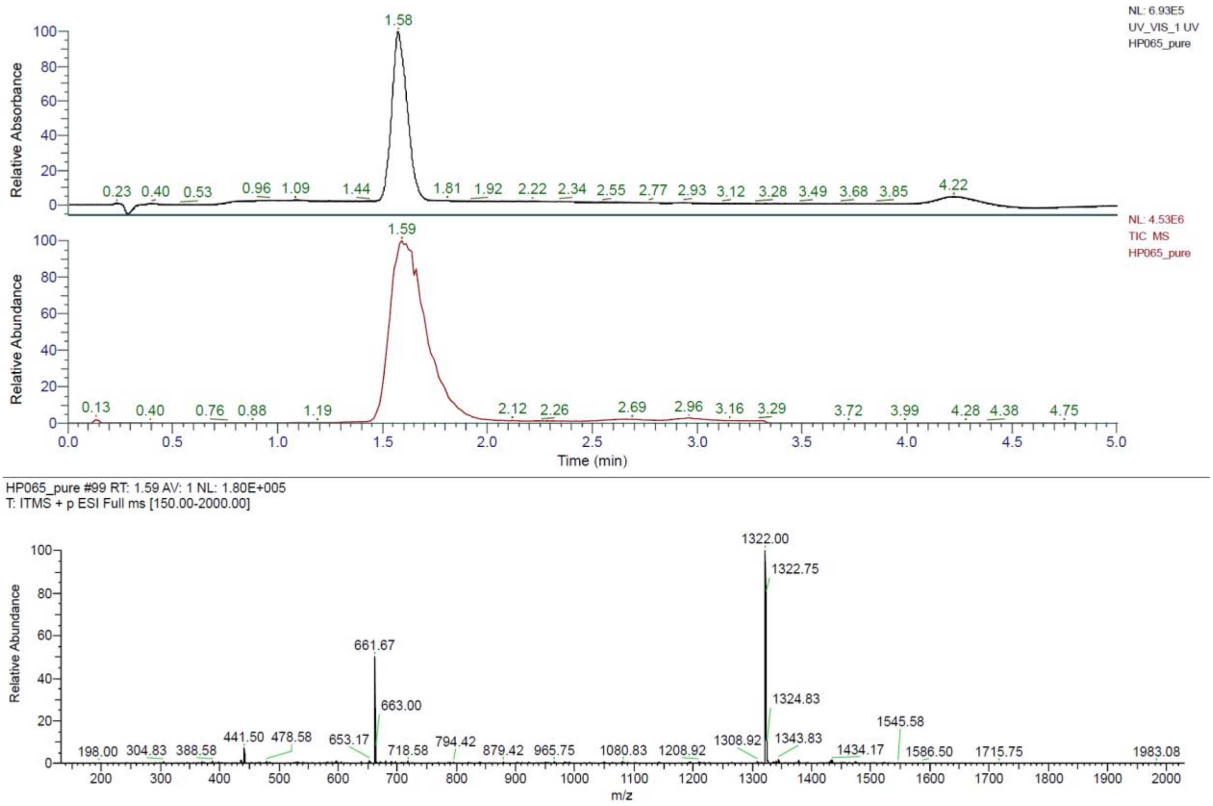

HRMS spectra:

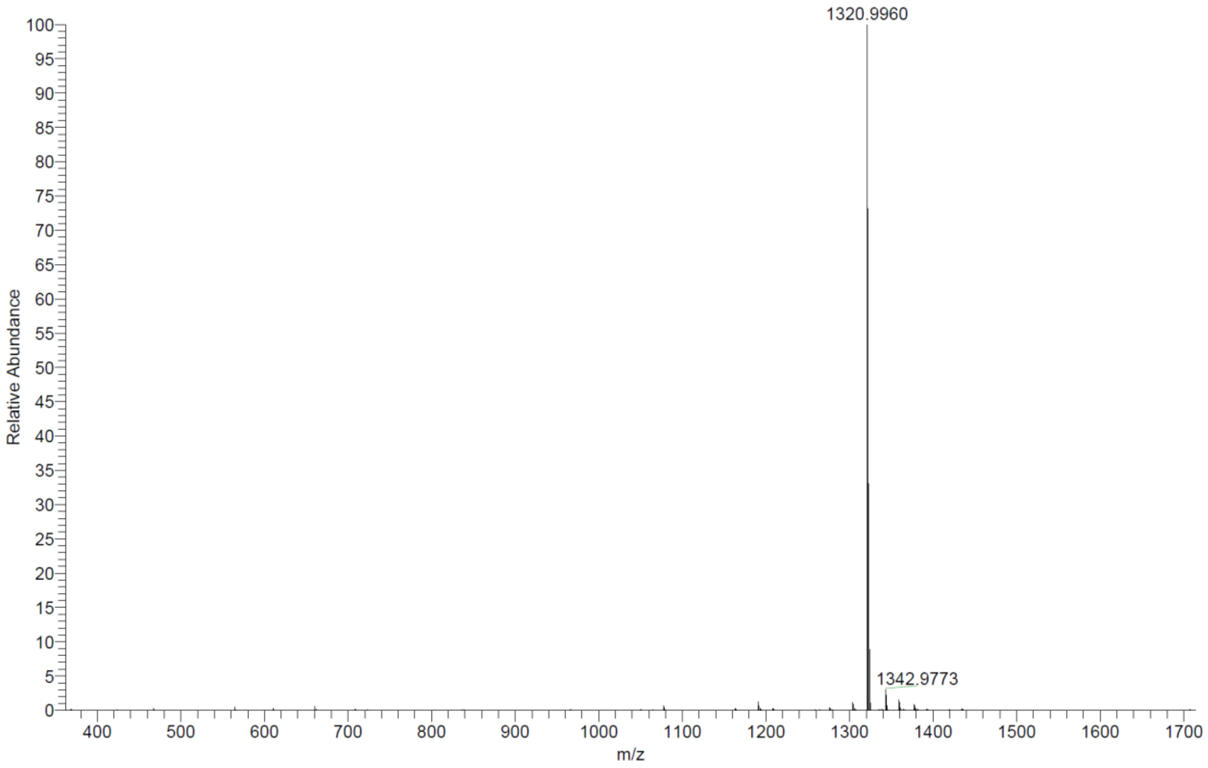
1320.9960
1342.9773
Relative Abundance
m/z

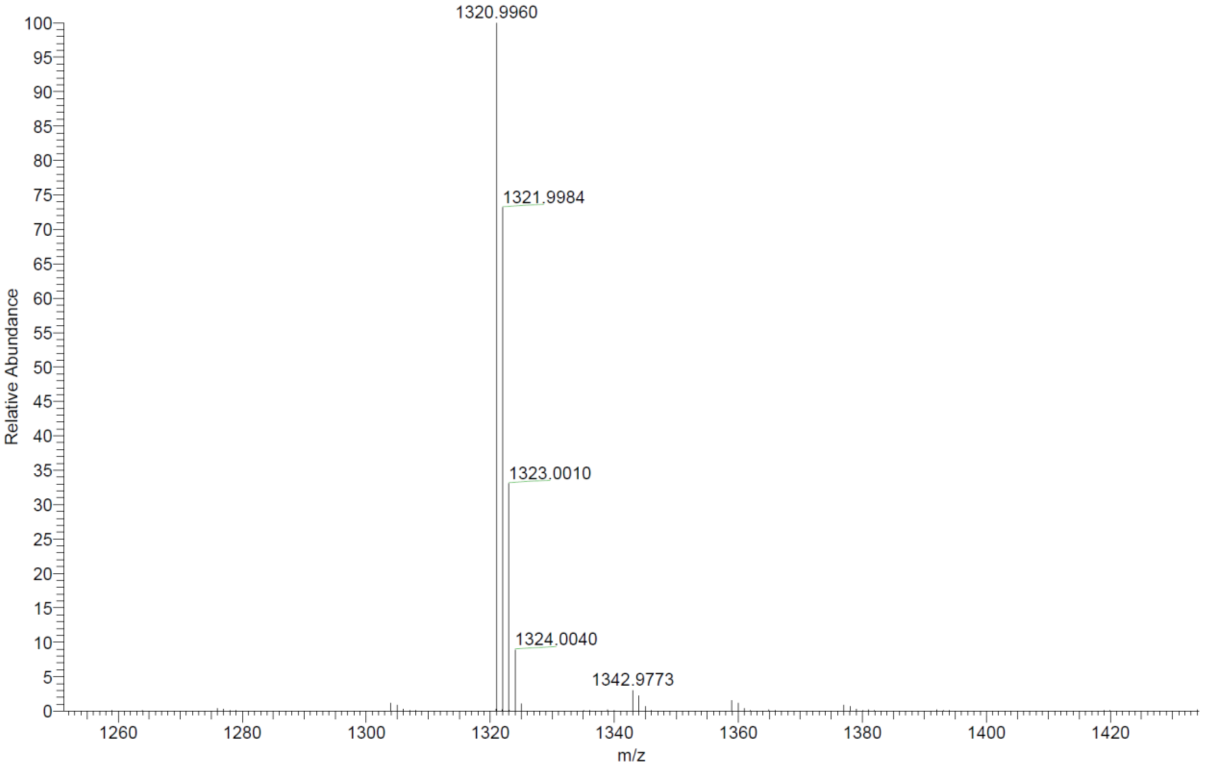
1320.9960
1321.9984
1323.0010
1324.0040
1342.9773
Relative Abundance
m/z

<u>KKLLK</u>L<u>LKLLL</u> (*sr*-ln65L[6]) was obtained as white solid after preparative RP-HPLC (77.3 mg, 49.4%). Analytical RP-HPLC: t_R = 1.56 min (A/D 100:0 to 0:100 in 3.5 min, λ = 214 nm). MS (ESI+): $C_{66}H_{128}N_{16}O_{11}$ calc./obs. 1321.99/1322.00 Da $[M+H]^+$.

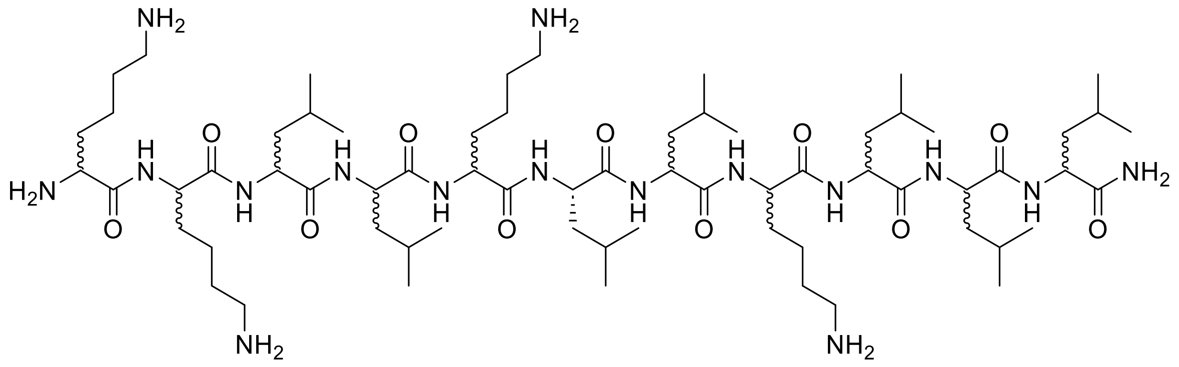

Chemical Formula: $C_{66}H_{128}N_{16}O_{11}$
Exact Mass: 1320.99
Molecular Weight: 1321.85

Analytical HPLC-MS data:

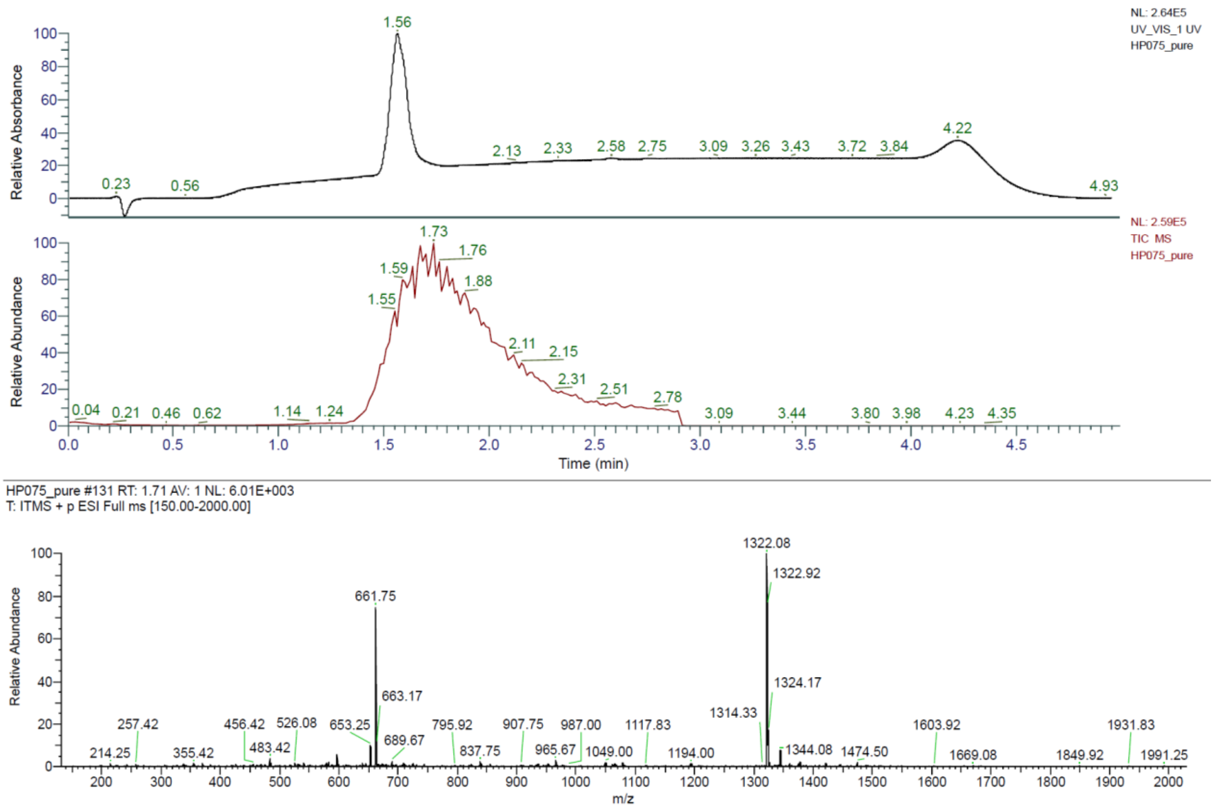

HRMS spectra:

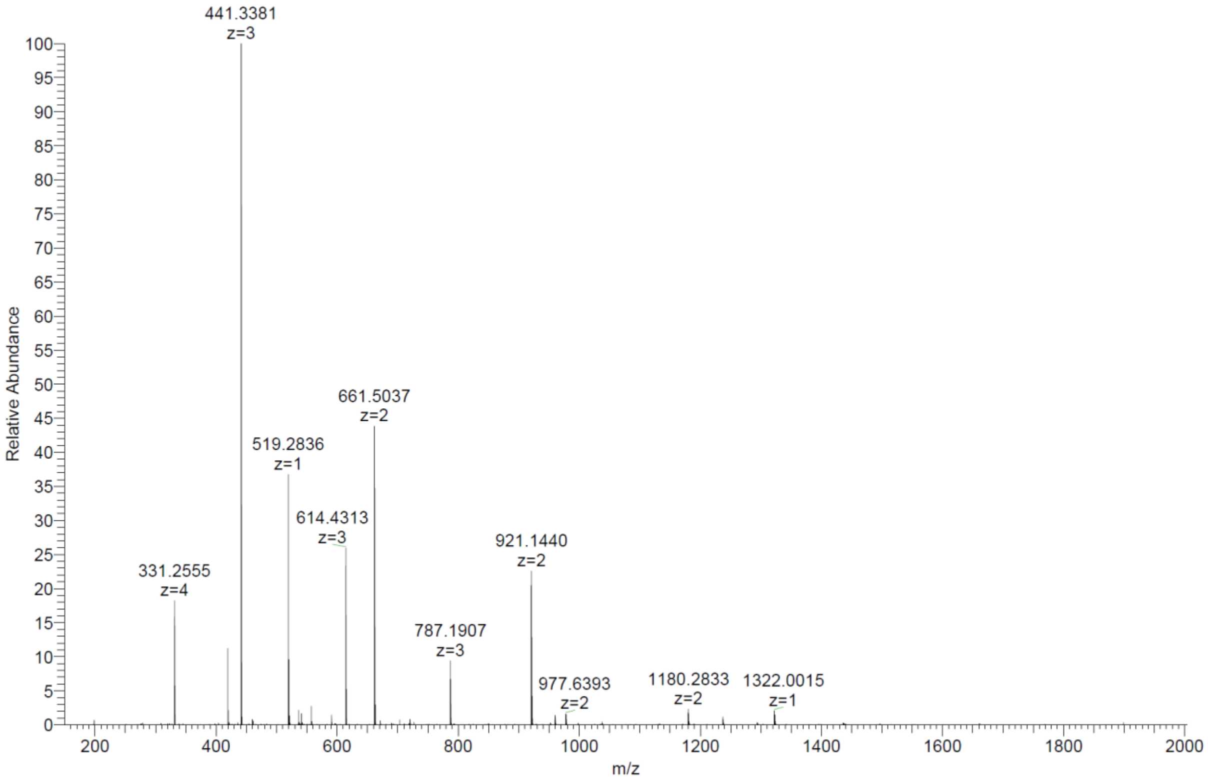

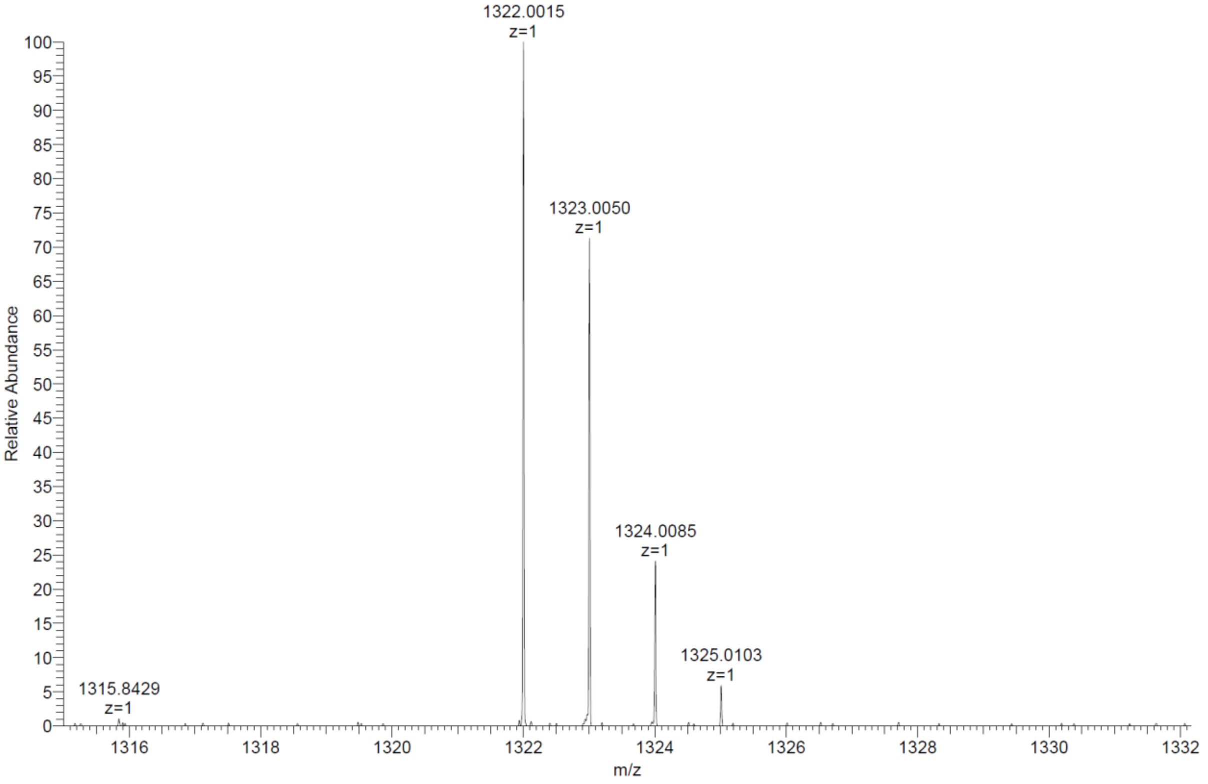

KkLLKLLKLLL (HP1) was obtained as white solid after preparative RP-HPLC (53.0 mg, 47.7%). Analytical RP-HPLC: t_R = 1.67 min (A/D 100:0 to 0:100 in 3.5 min, λ = 214 nm). MS (ESI+): $C_{66}H_{128}N_{16}O_{11}$ calc./obs. 1320.99/1321.00 Da [M].

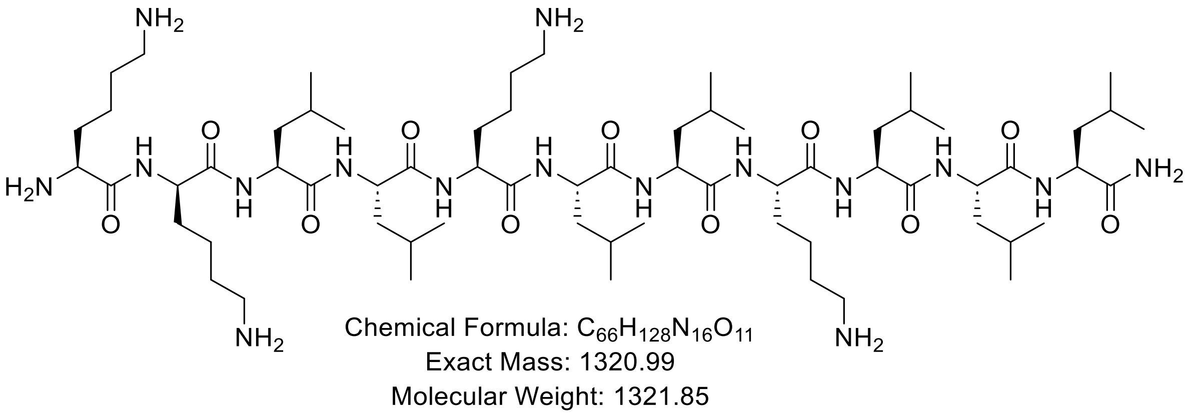

Analytical HPLC-MS data:

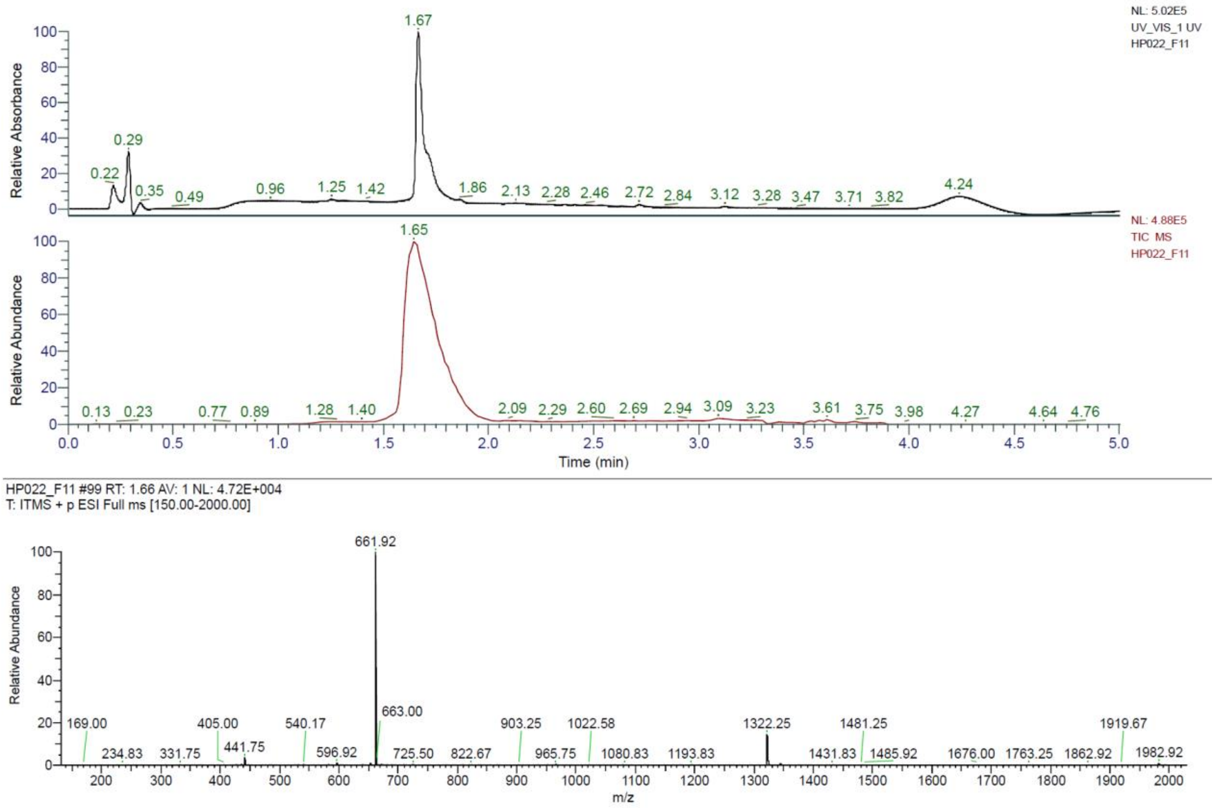

HRMS spectra:

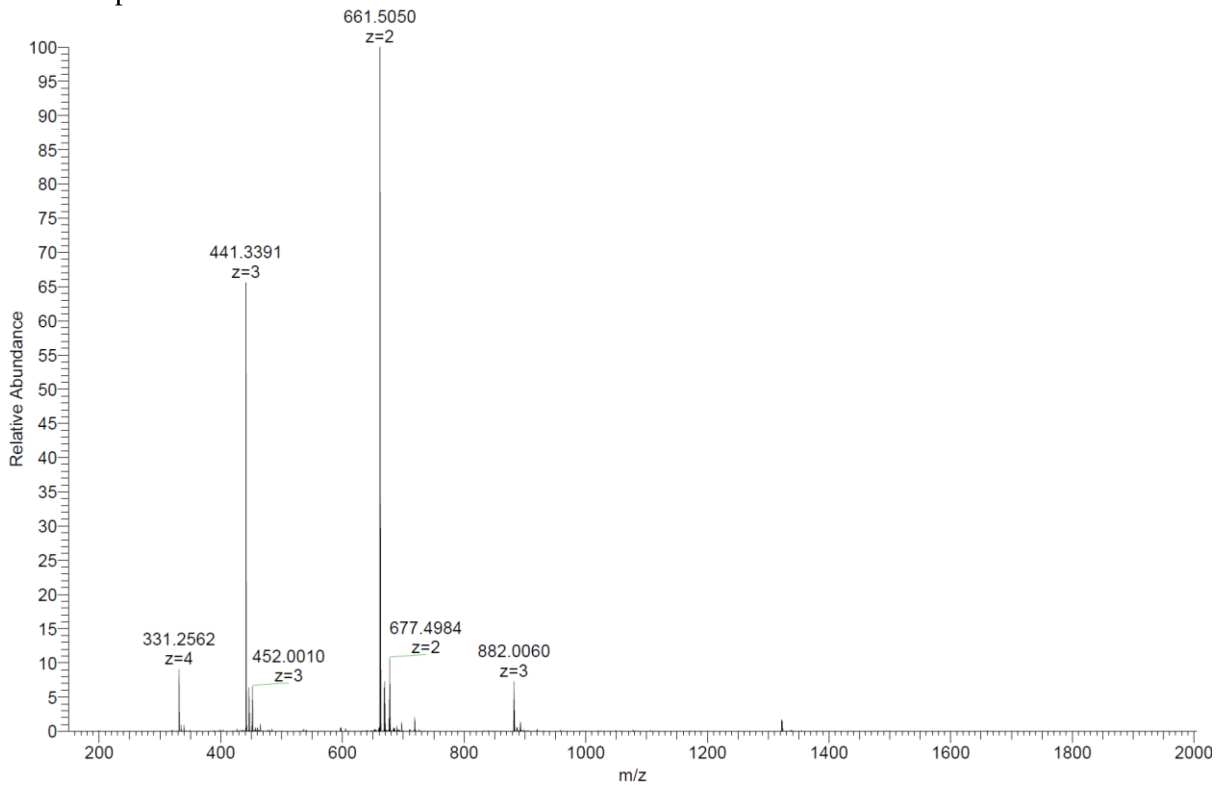

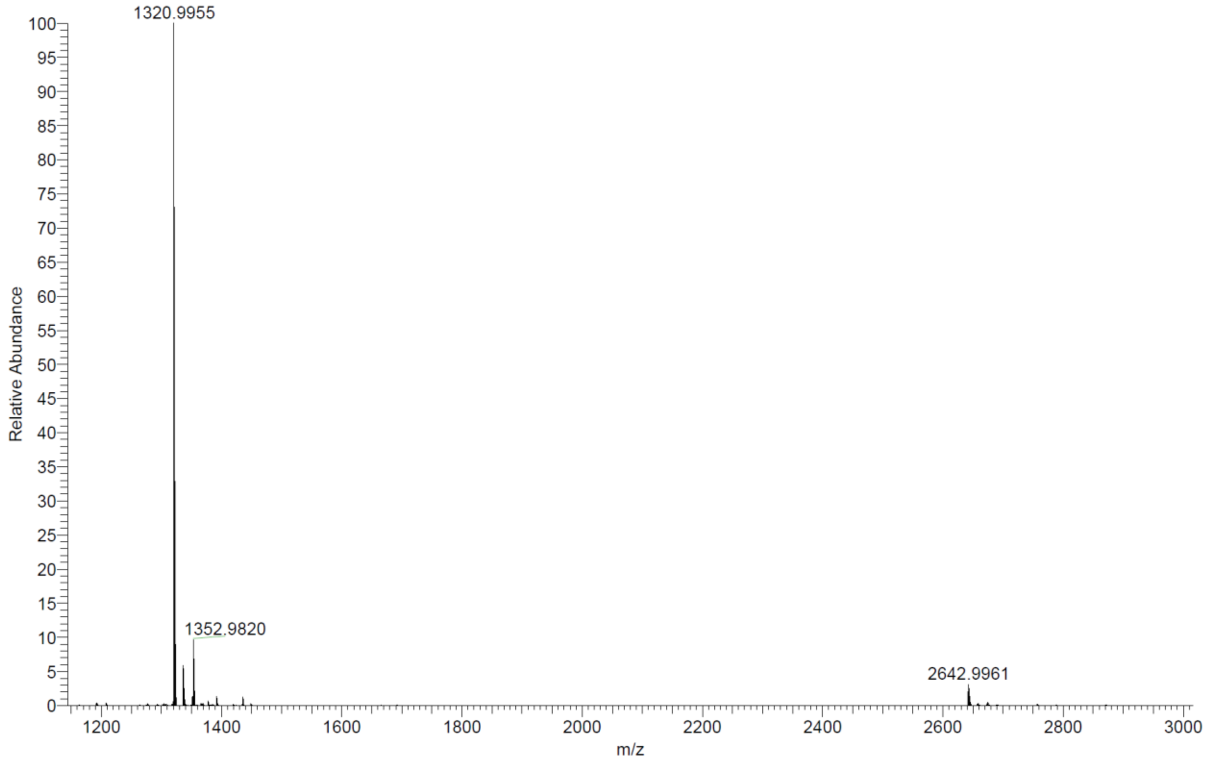

kkLLKLLKLLL (HP2) was obtained as white solid after preparative RP-HPLC (56.7 mg, 51.0%). Analytical RP-HPLC: t_R = 1.66 min (A/D 100:0 to 0:100 in 3.5 min, λ = 214 nm). MS (ESI+): $C_{66}H_{128}N_{16}O_{11}$ calc./obs. 1320.99/1320.99 Da [M].

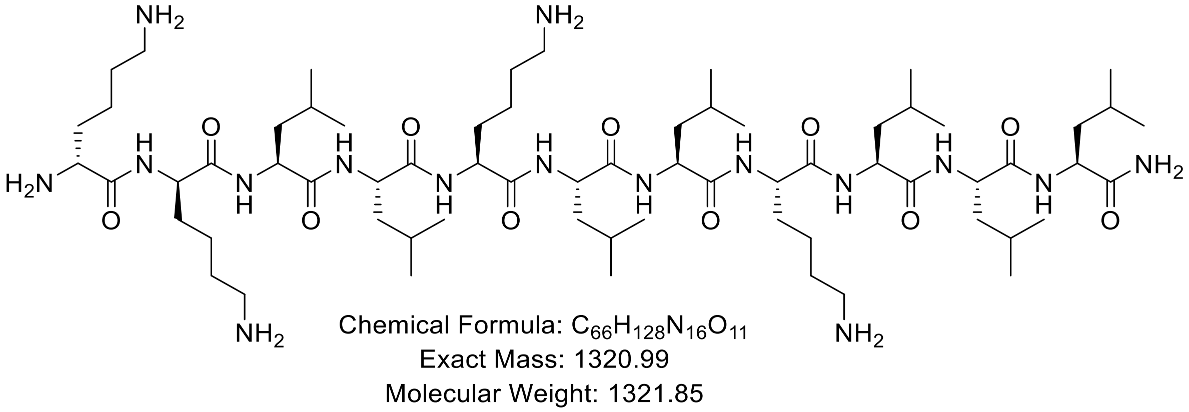

Analytical HPLC-MS data:

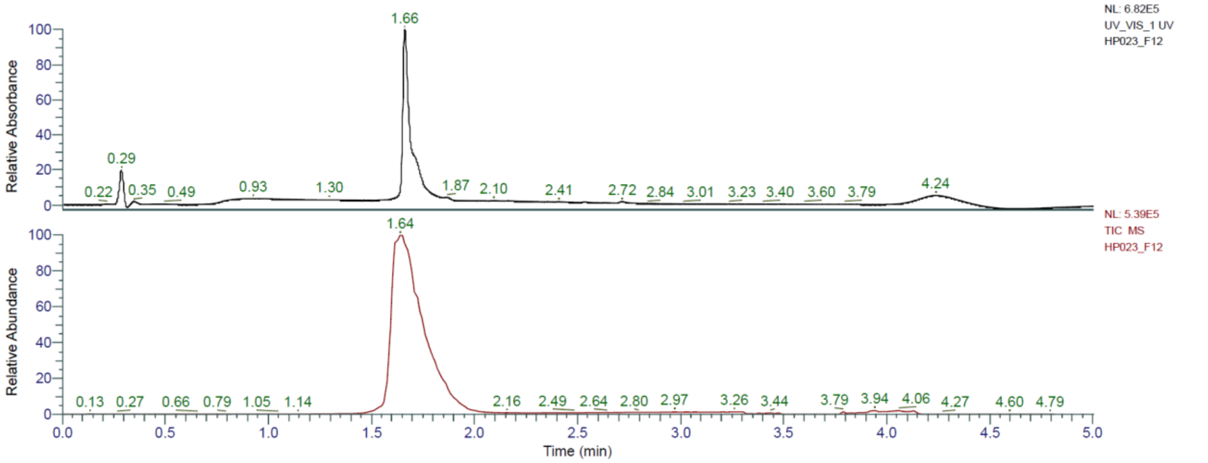

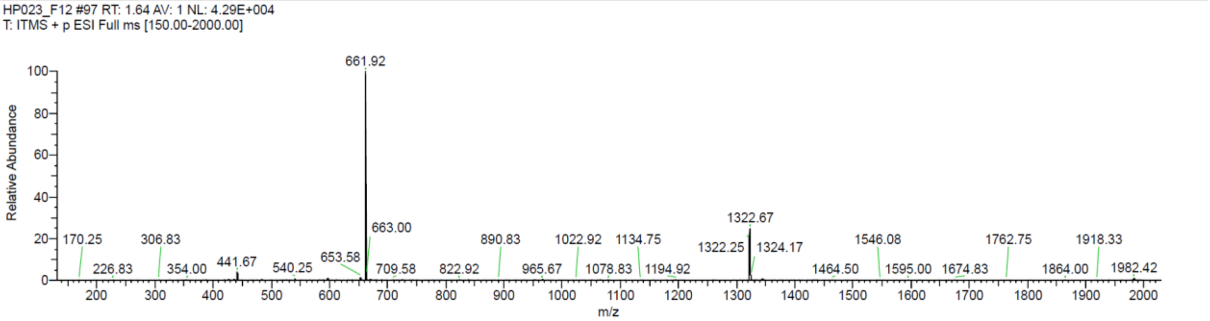

HRMS spectra:

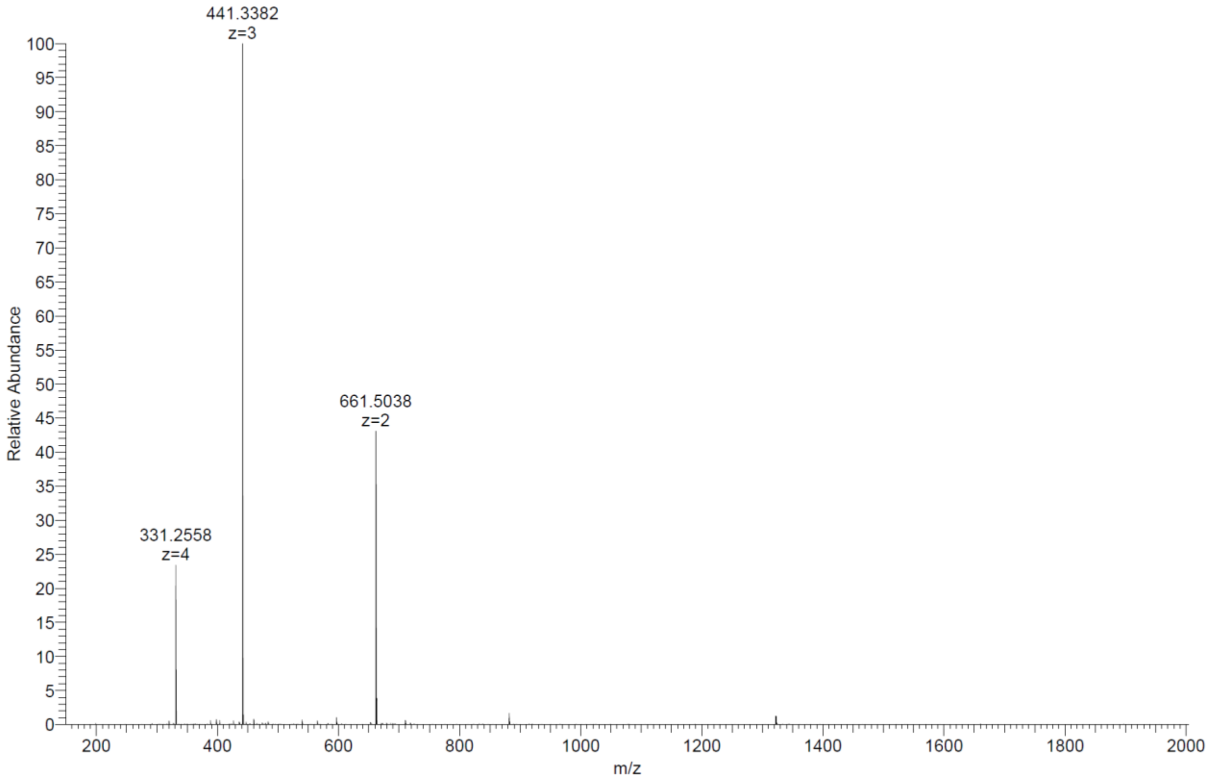

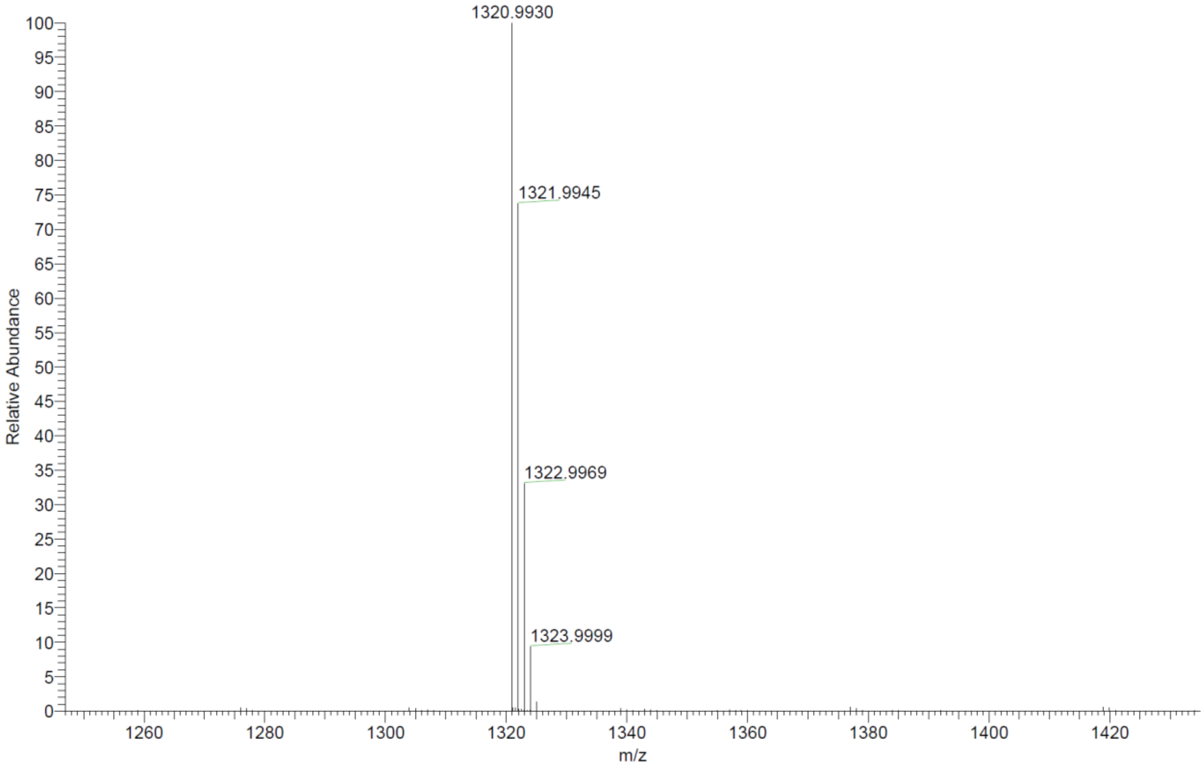

KkLLkLLKLLL (HP3) was obtained as white solid after preparative RP-HPLC (50.1 mg, 45.1%). Analytical RP-HPLC: t_R = 1.61 min (A/D 100:0 to 0:100 in 3.5 min, λ = 214 nm). MS (ESI+): $C_{66}H_{128}N_{16}O_{11}$ calc./obs. 1321.99/1322.00 Da $[M+H]^+$.

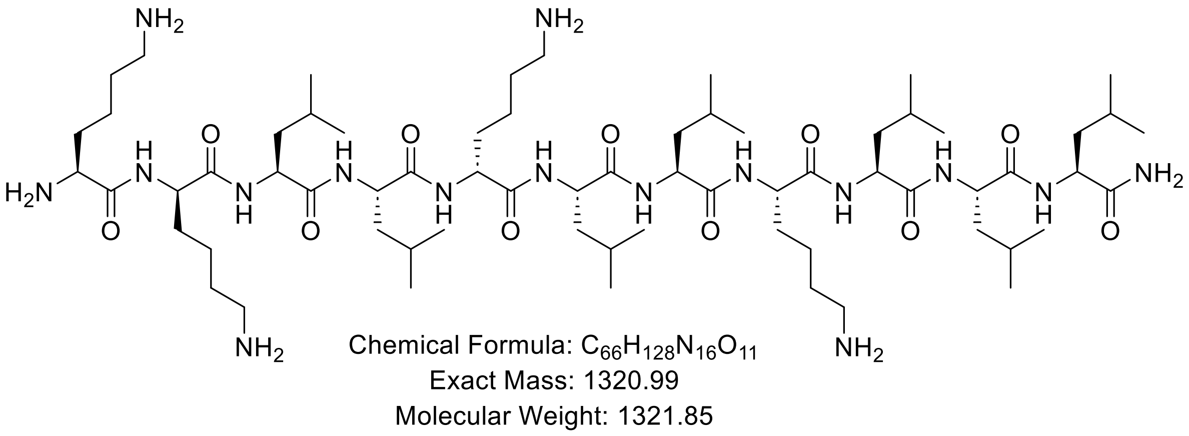

Analytical HPLC-MS data:

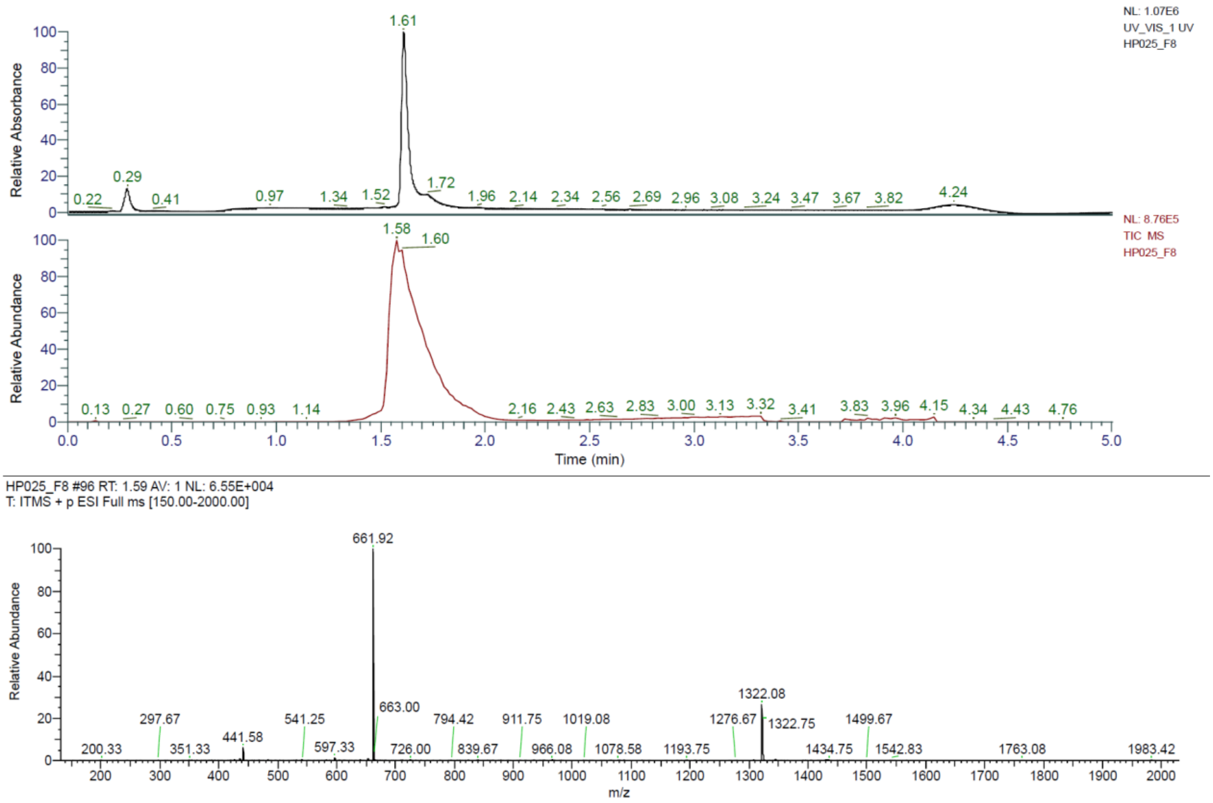

HRMS spectra:

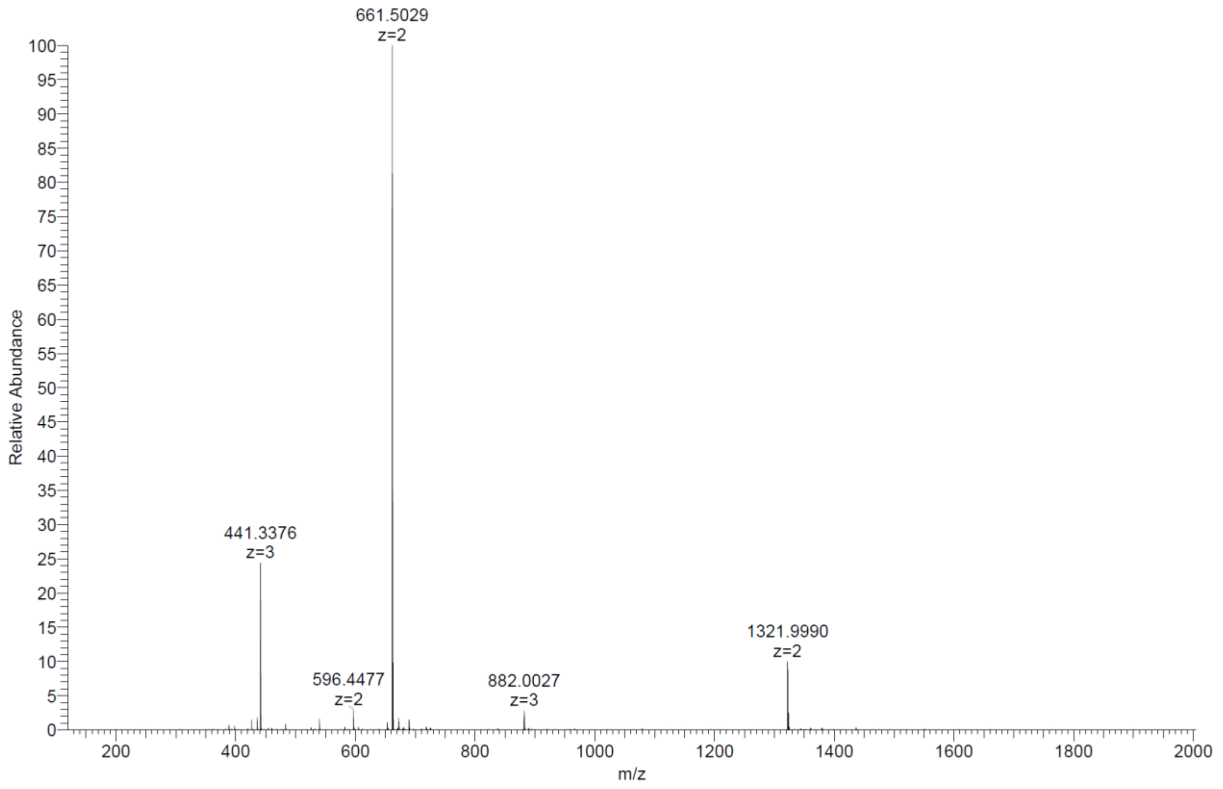

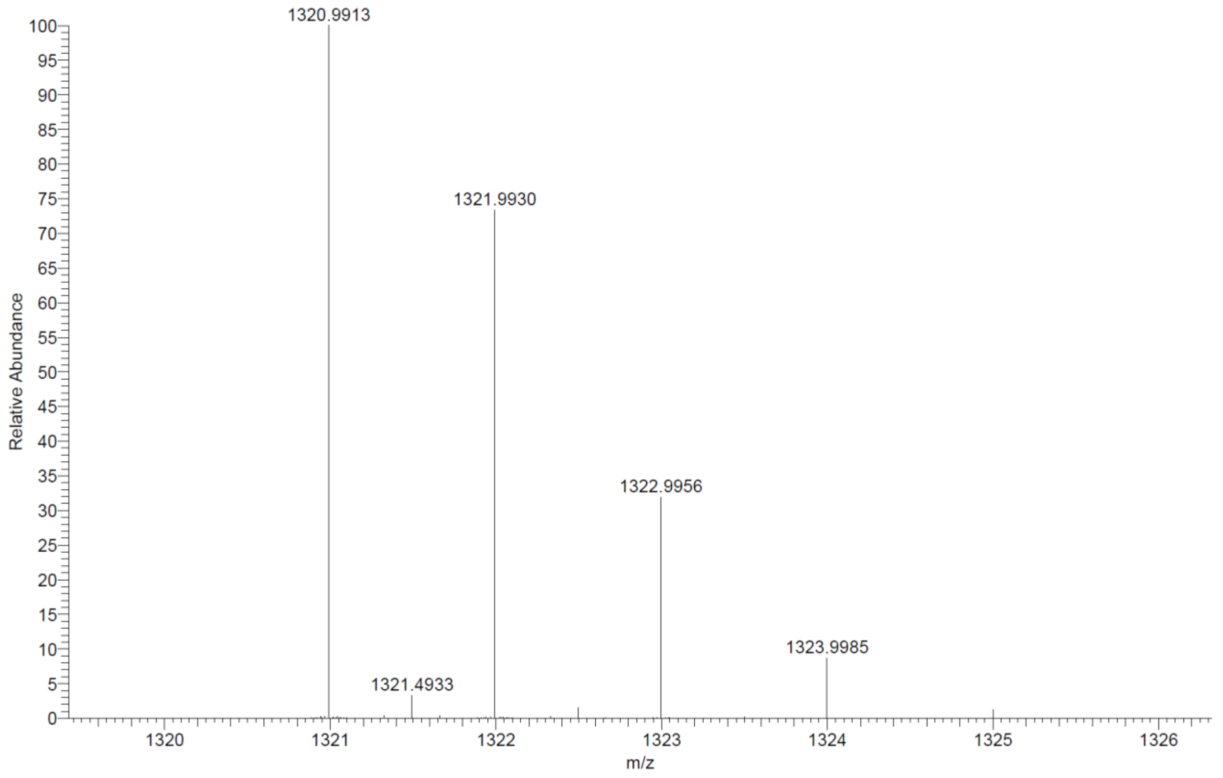

KkLlKLLKLLL (HP4) was obtained as white solid after preparative RP-HPLC (42.6 mg, 38.3%). Analytical RP-HPLC: t_R = 1.74 min (A/D 100:0 to 0:100 in 3.5 min, λ = 214 nm). MS (ESI+): $C_{66}H_{128}N_{16}O_{11}$ calc./obs. 1321.99/1322.00 Da $[M+H]^+$.

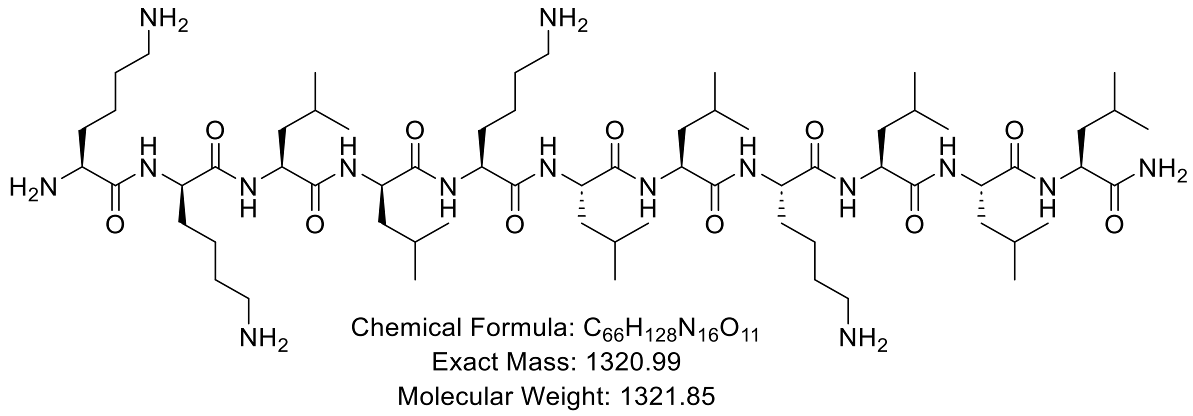

Analytical HPLC-MS data:

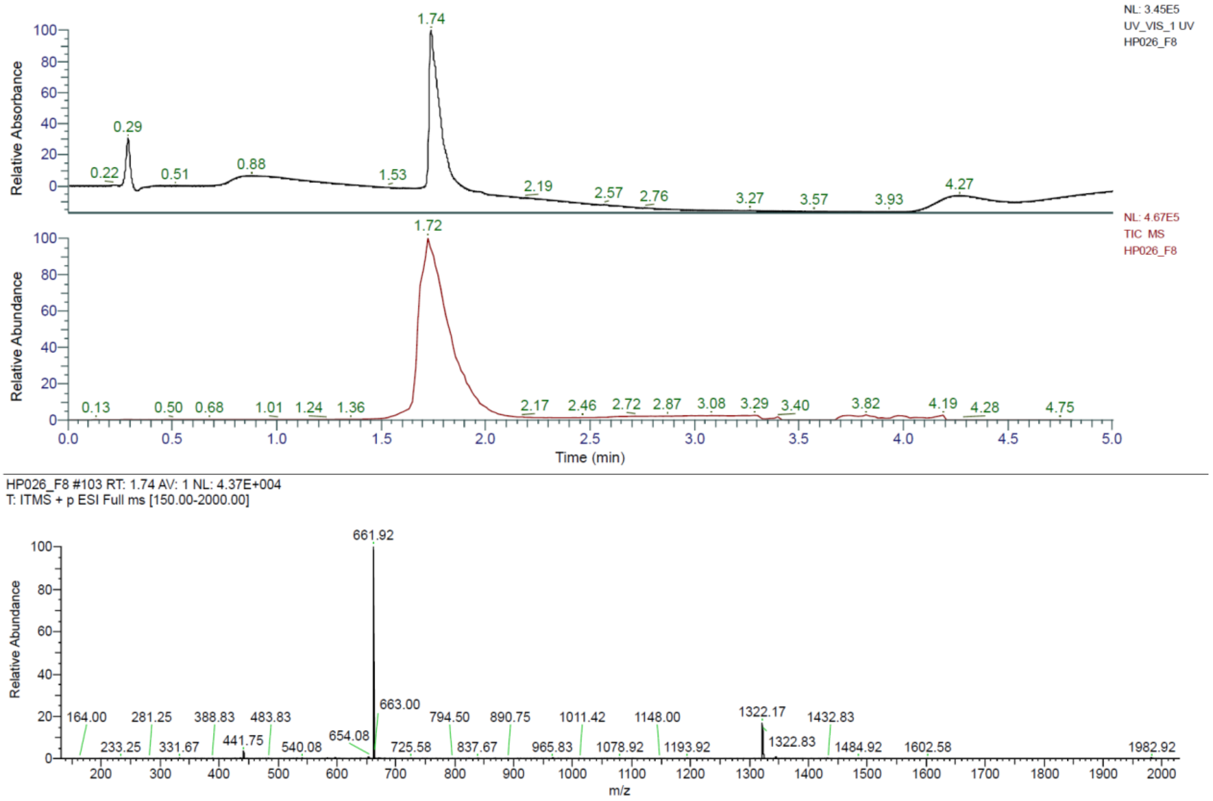

HRMS spectra:

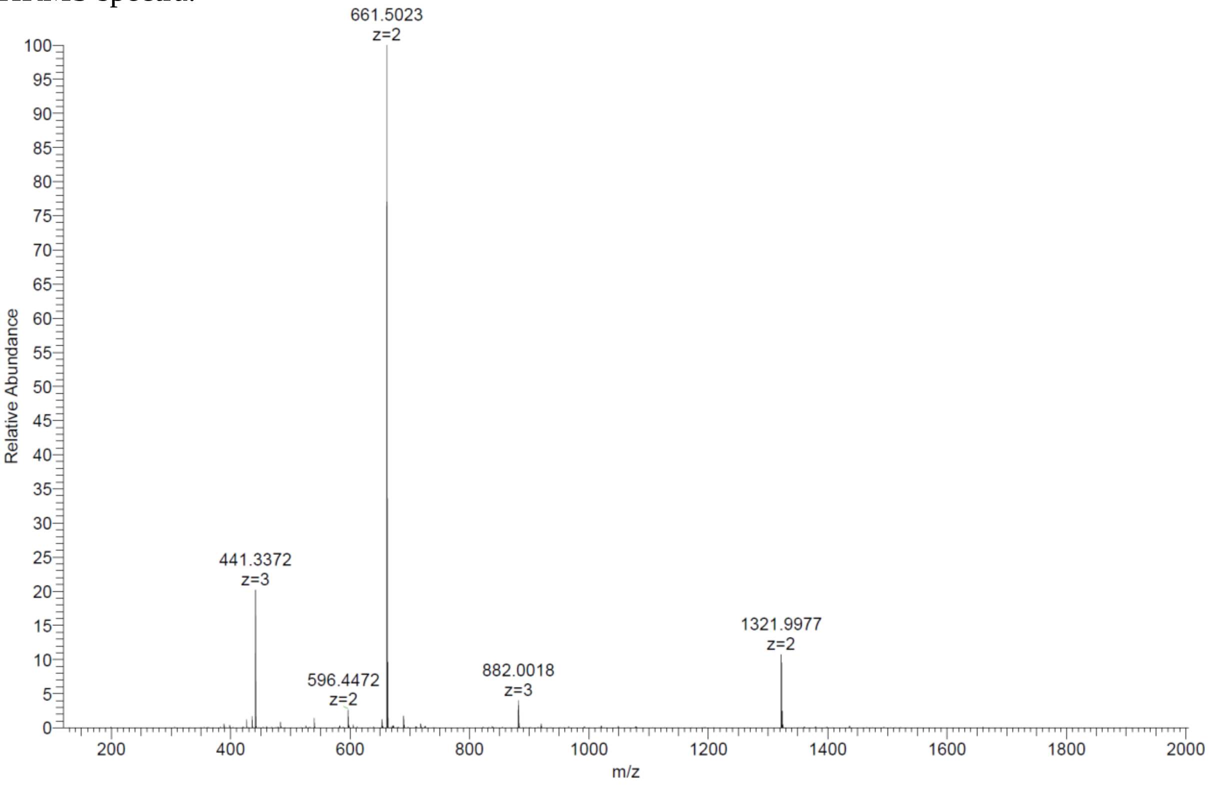

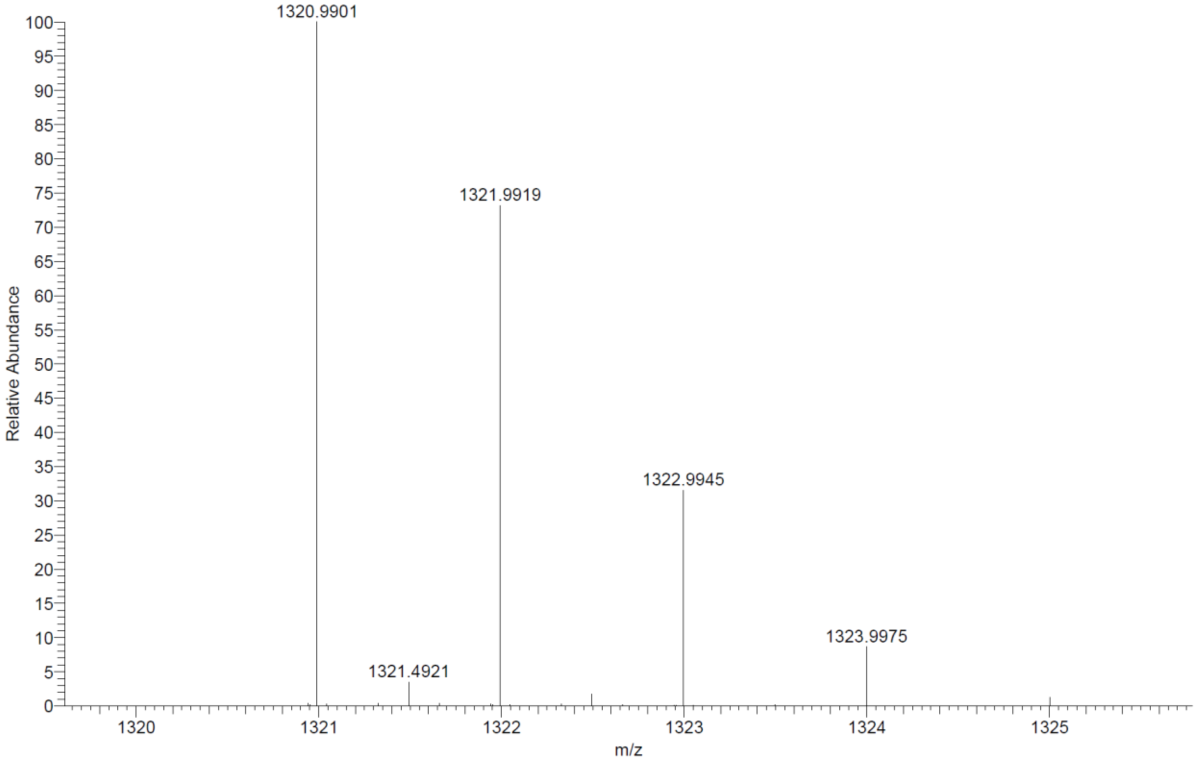

kKLLKLLKLLl (HP5) was obtained as white solid after preparative RP-HPLC (49.1 mg, 44.2%). Analytical RP-HPLC: t_R = 1.65 min (A/D 100:0 to 0:100 in 3.5 min, λ = 214 nm). MS (ESI+): $C_{66}H_{128}N_{16}O_{11}$ calc./obs. 1321.99/1322.00 Da $[M+H]^+$.

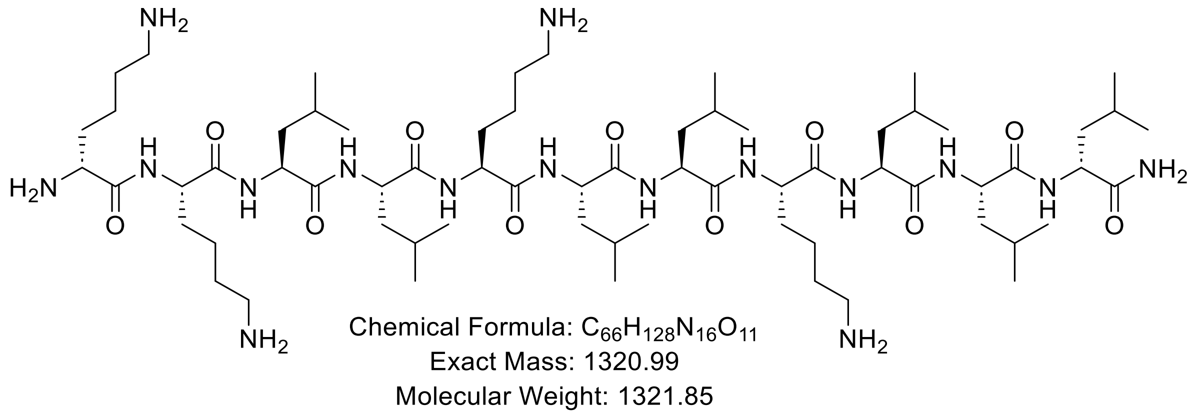

Analytical HPLC-MS data:

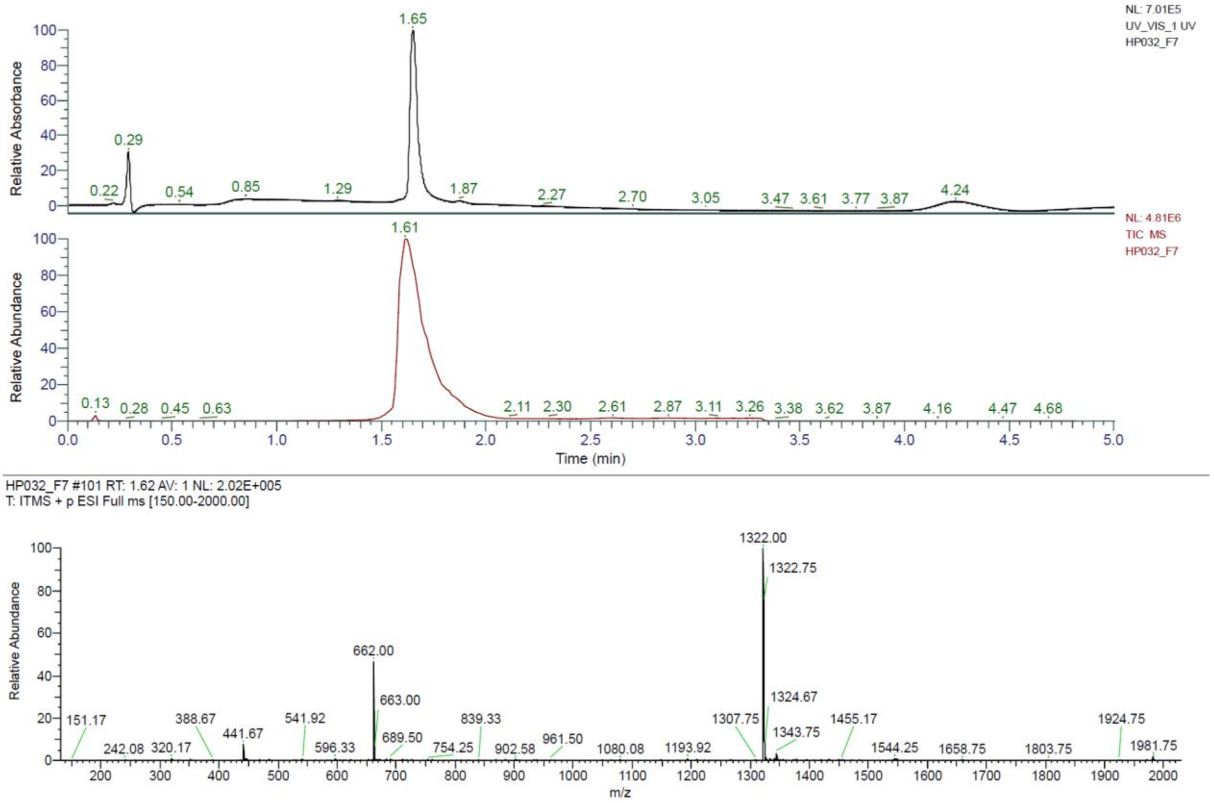

HRMS spectra:

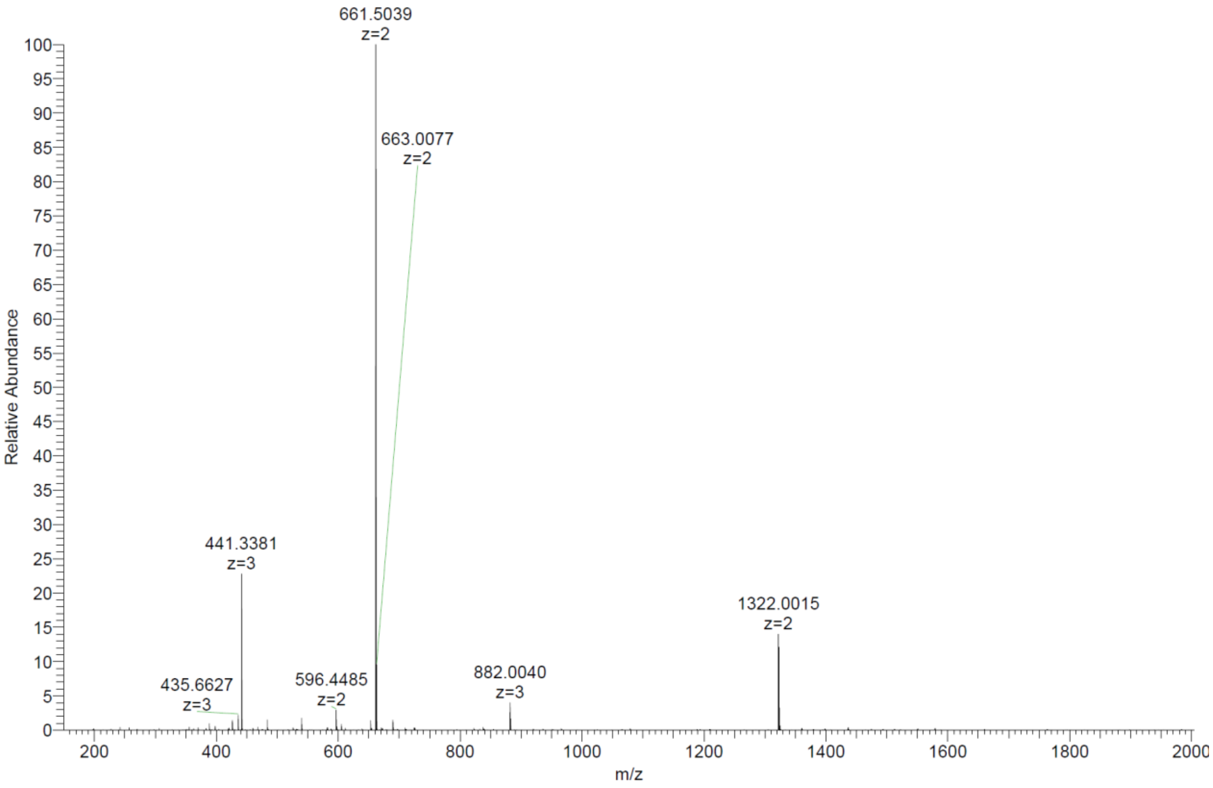

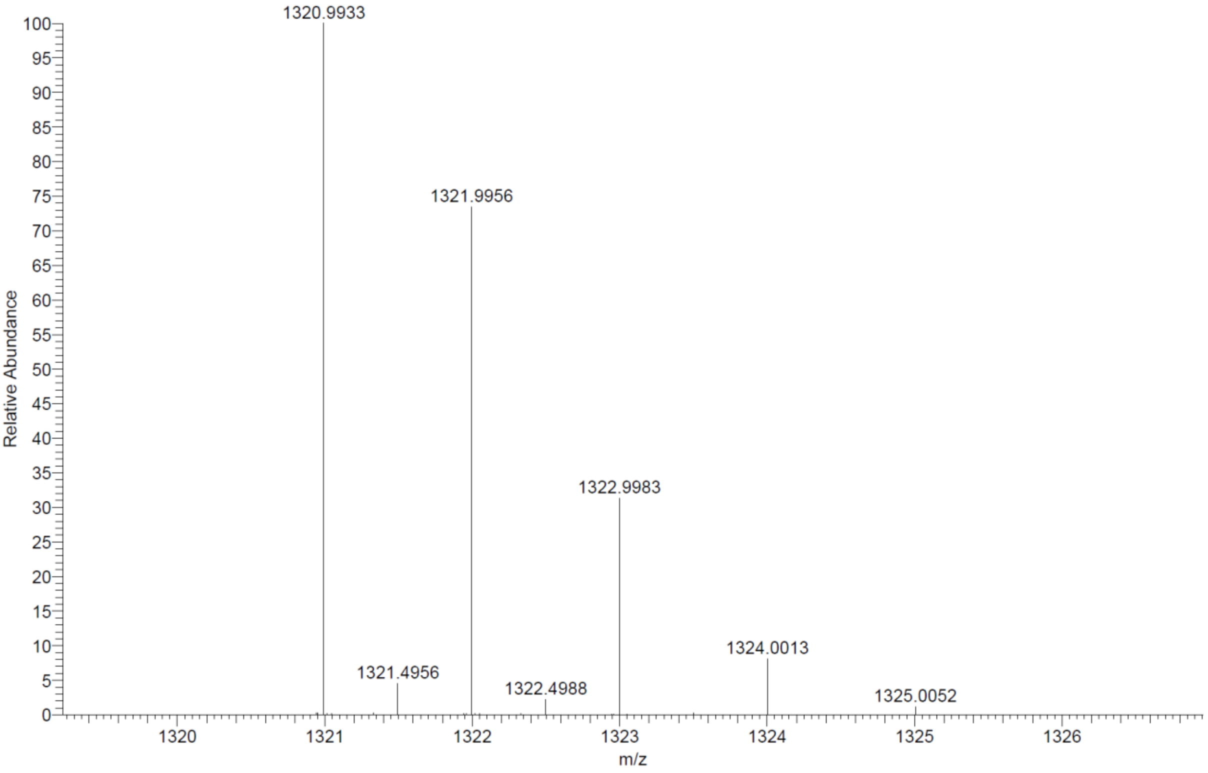

KKLLKllKLLL (HP6) was obtained as white solid after preparative RP-HPLC (49.3 mg, 44.4%). Analytical RP-HPLC: t_R = 1.53 min (A/D 100:0 to 0:100 in 3.5 min, λ = 214 nm). MS (ESI+): $C_{66}H_{128}N_{16}O_{11}$ calc./obs. 1321.99/1322.00 Da $[M+H]^+$.

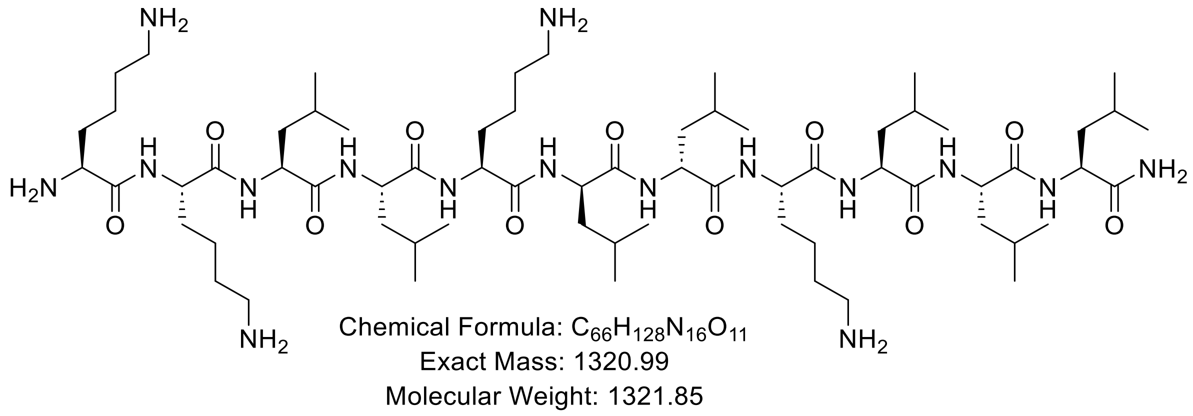

Analytical HPLC-MS data:

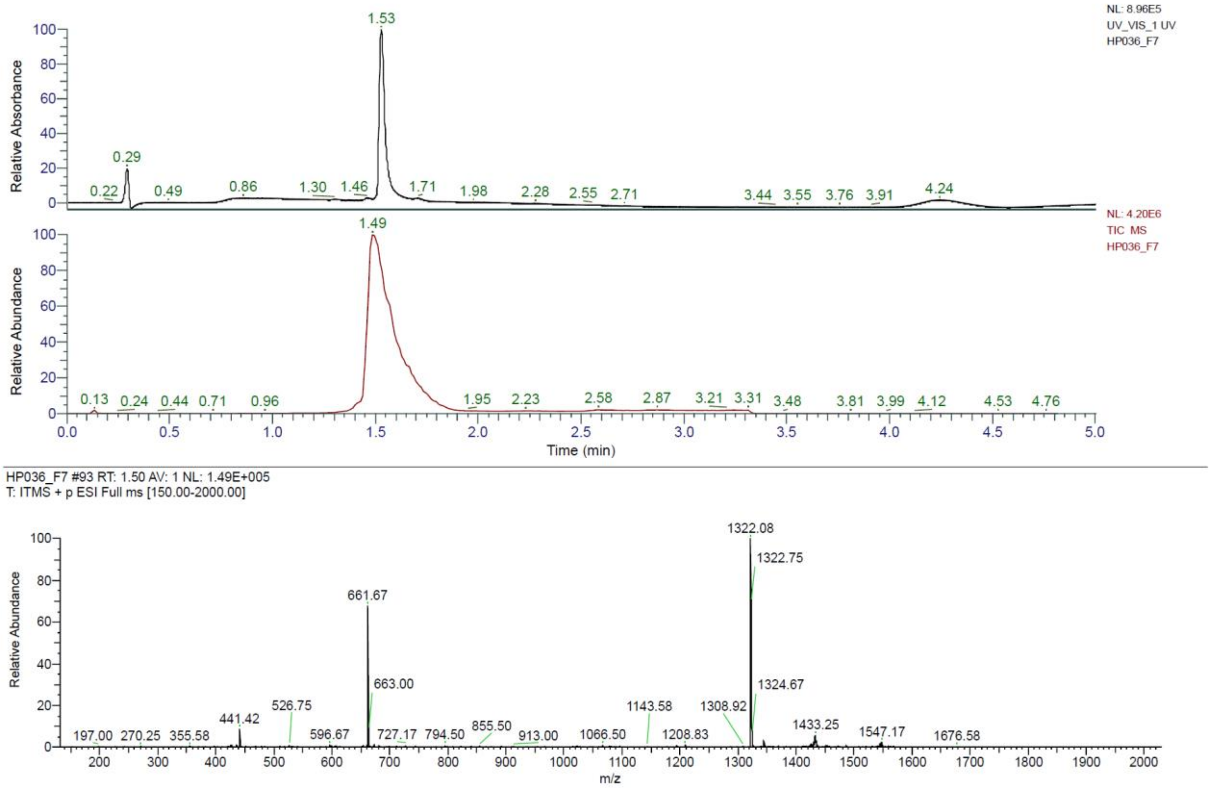

HRMS spectra:

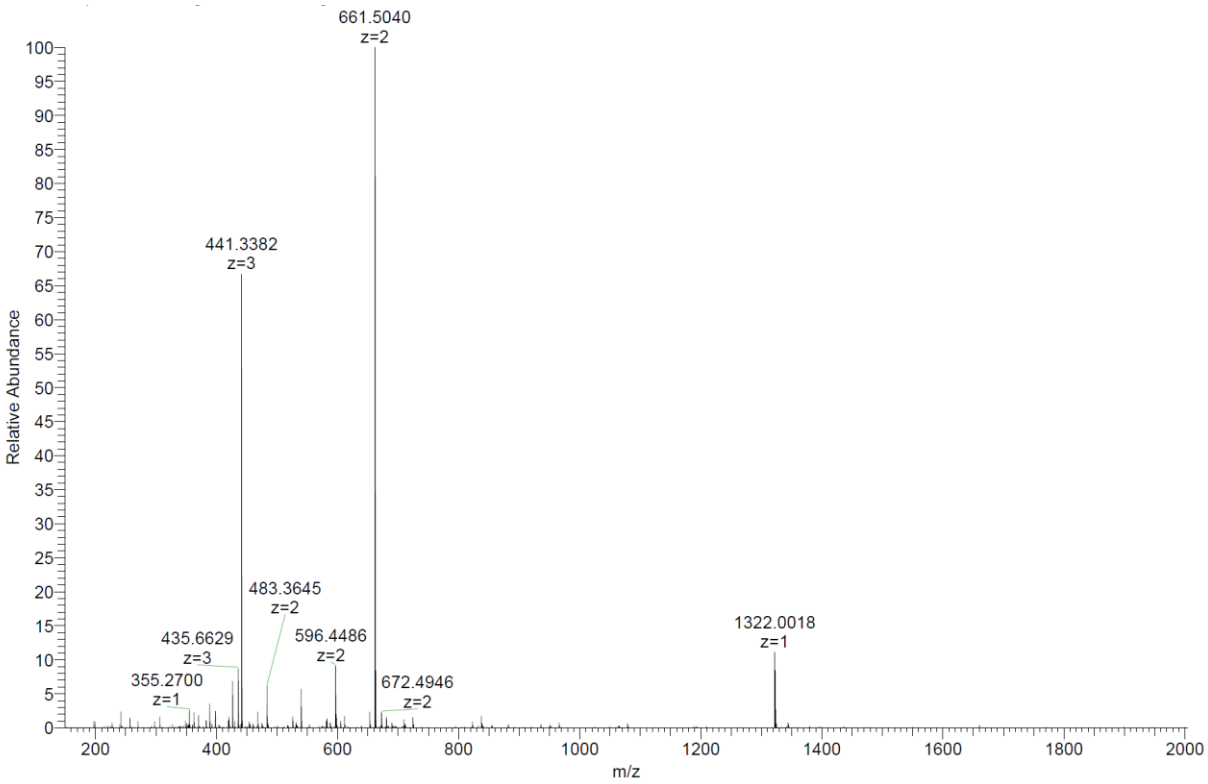

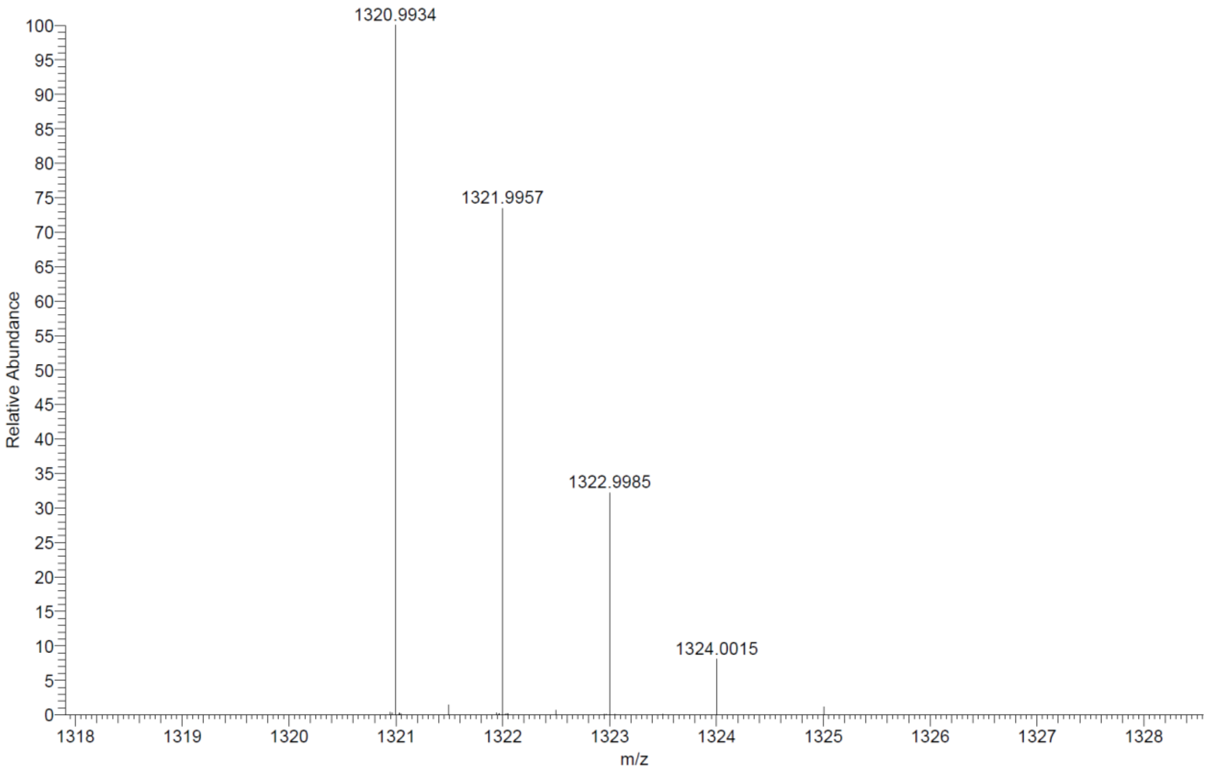

kkLLKLLKLLl (HP7) was obtained as white solid after preparative RP-HPLC (60.6 mg, 54.5%). Analytical RP-HPLC: t_R = 1.62 min (A/D 100:0 to 0:100 in 3.5 min, λ = 214 nm). MS (ESI+): $C_{66}H_{128}N_{16}O_{11}$ calc./obs. 1321.99/1322.00 Da $[M+H]^+$.

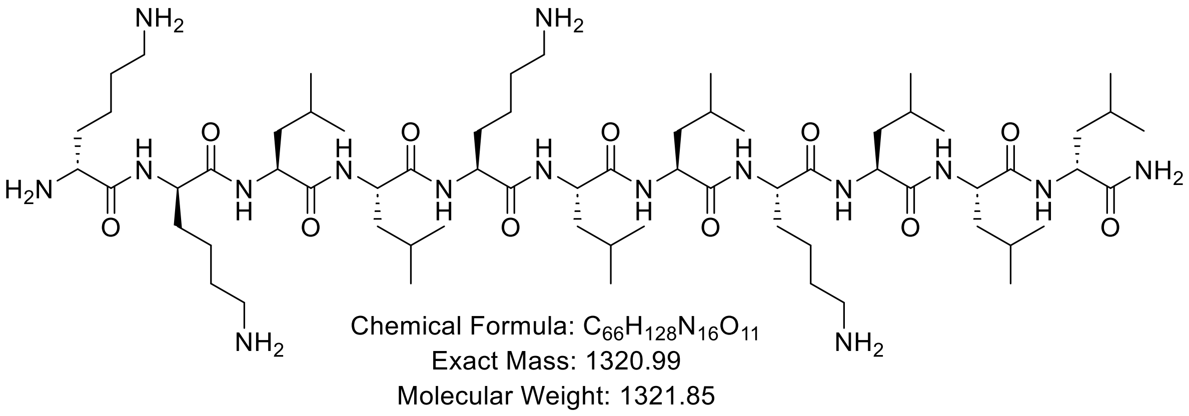

Analytical HPLC-MS data:

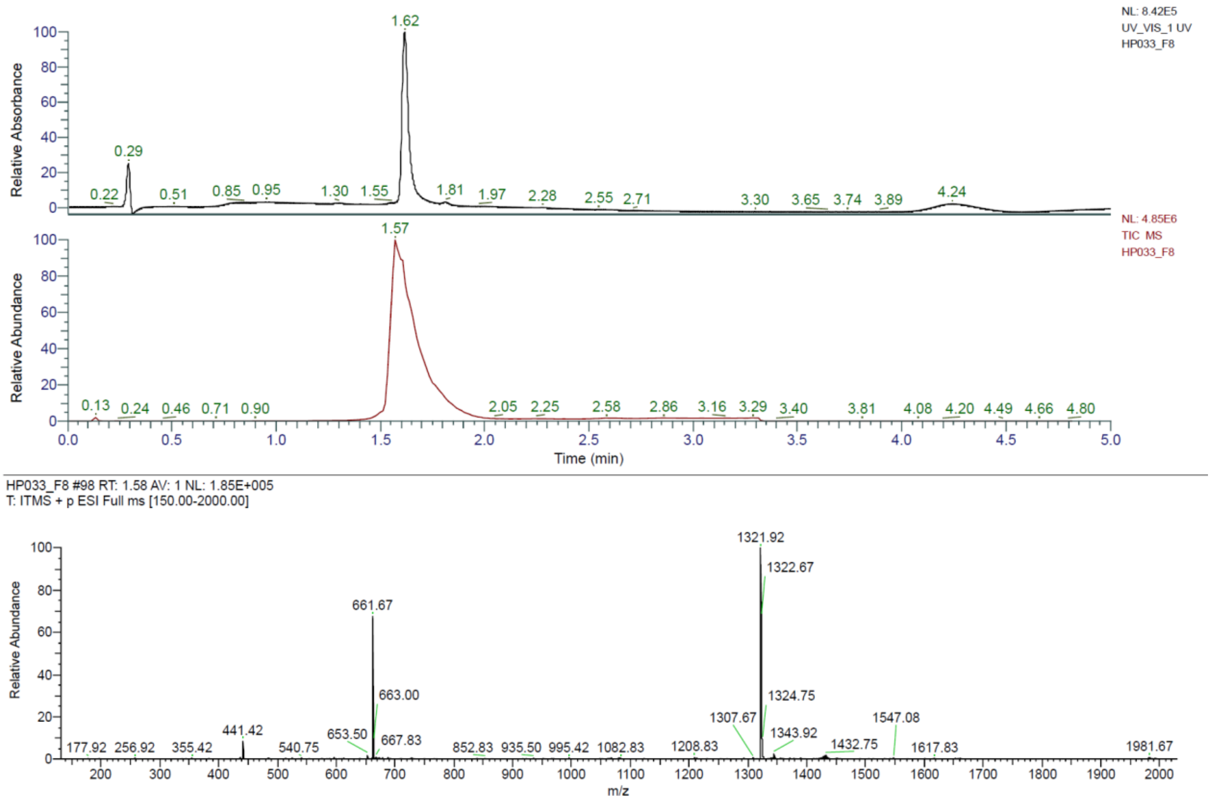

HRMS spectra:

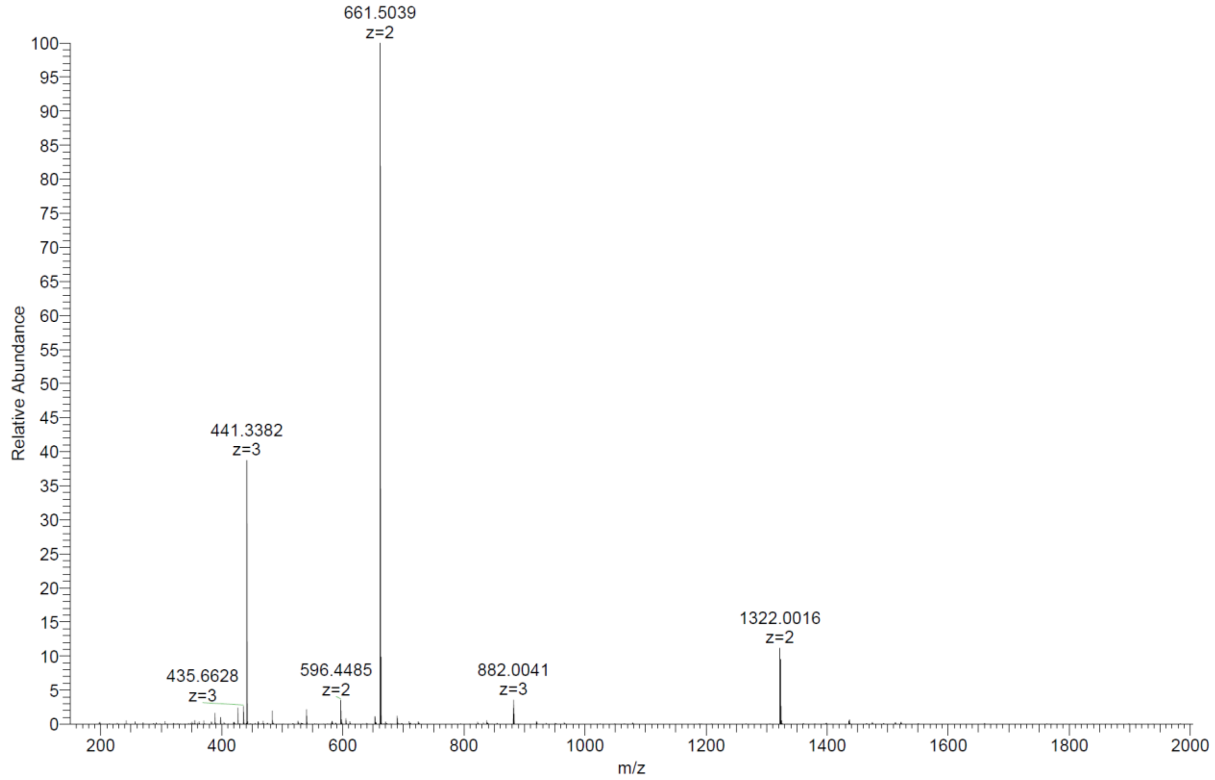

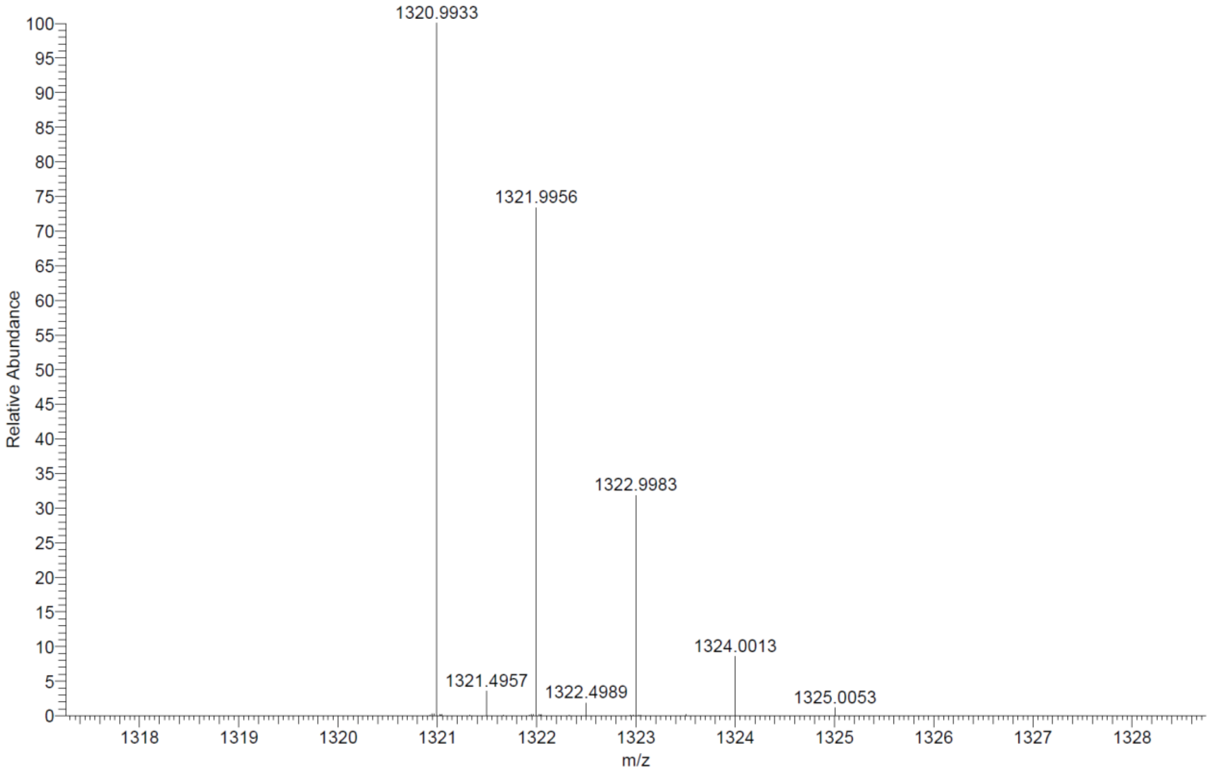

KllIKLLKLLL (HP8) was obtained as white solid after preparative RP-HPLC (46.9 mg, 42.2%). Analytical RP-HPLC: t_R = 1.63 min (A/D 100:0 to 0:100 in 3.5 min, λ = 214 nm). MS (ESI+): $C_{66}H_{128}N_{16}O_{11}$ calc./obs. 1321.99/1322.00 Da $[M+H]^+$.

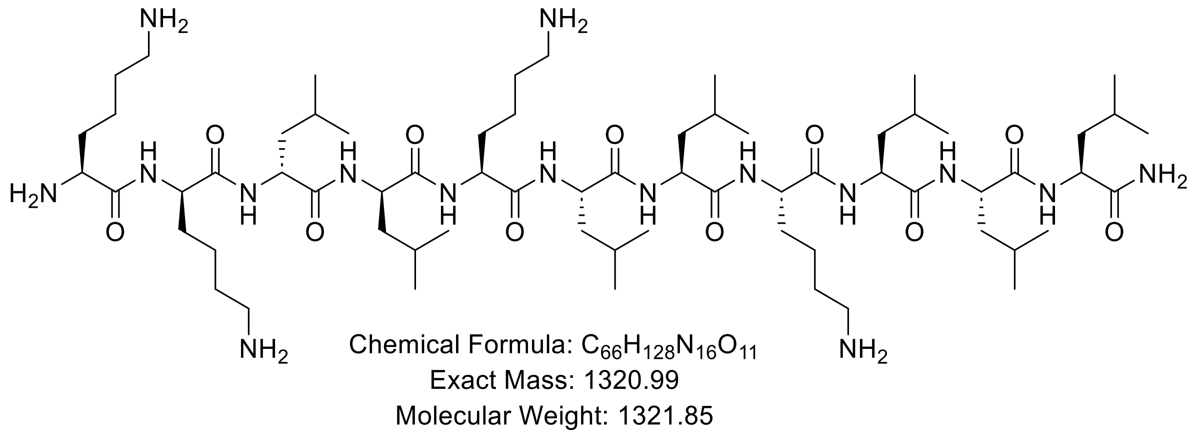

Analytical HPLC-MS data:

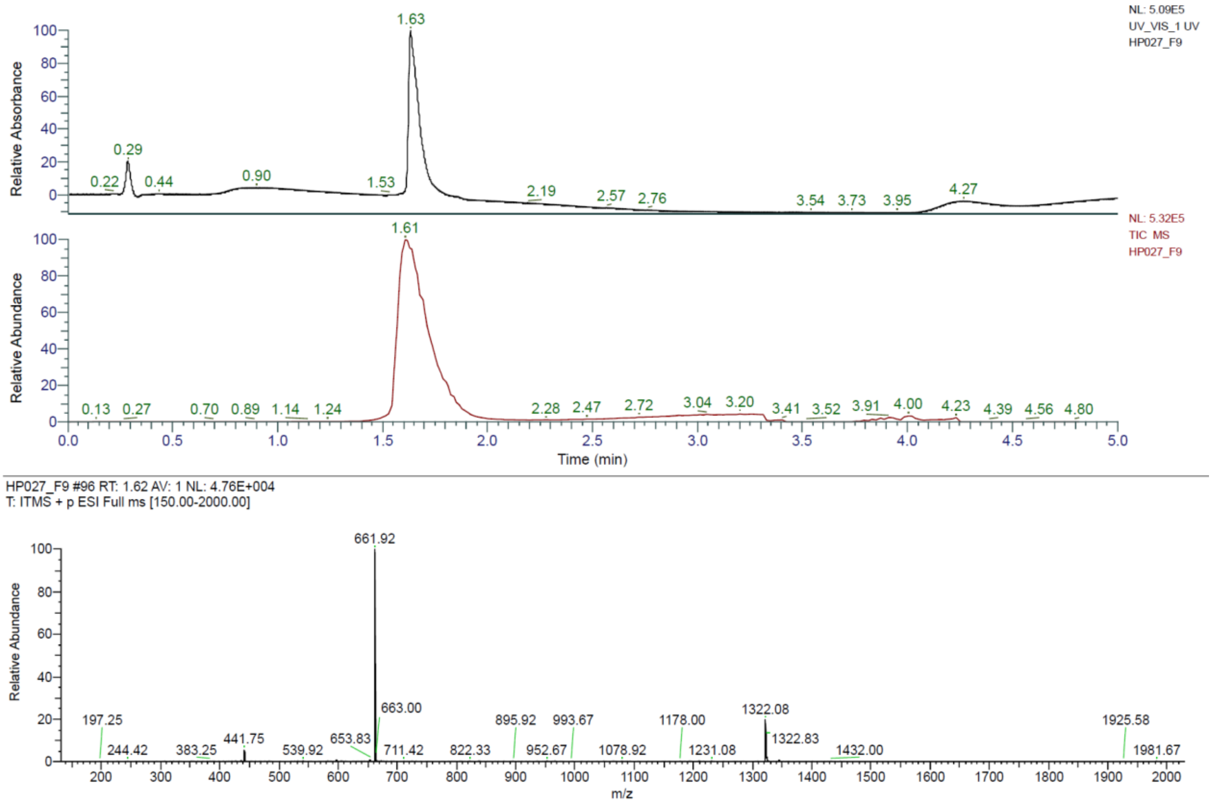

HRMS spectra:

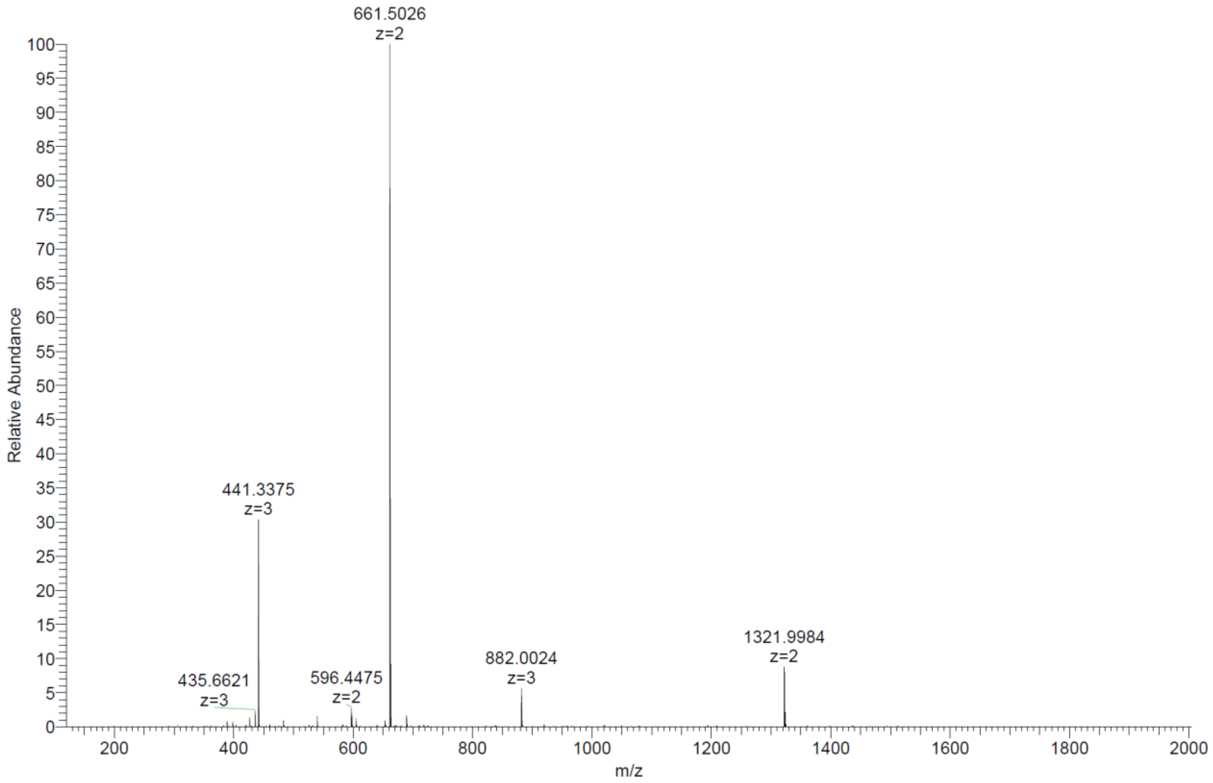

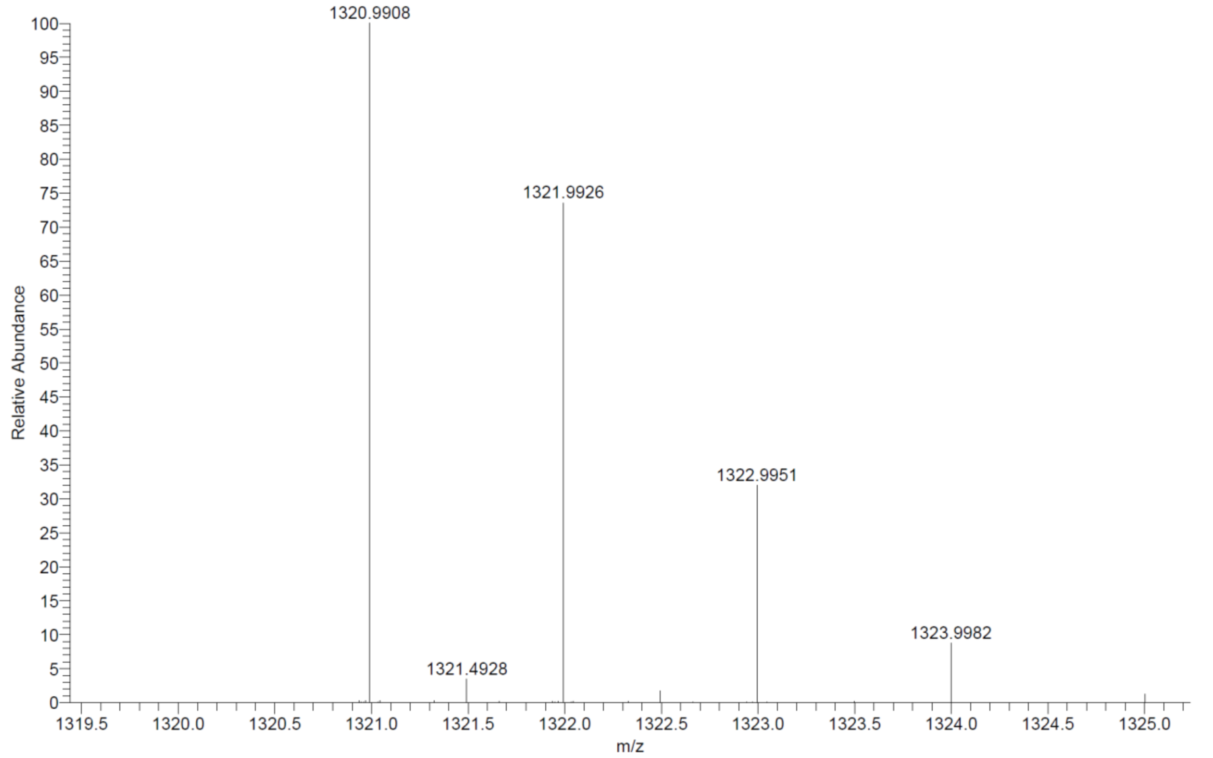

KKLLklIKLLL (HP9) was obtained as white solid after preparative RP-HPLC (63.9 mg, 57.5%). Analytical RP-HPLC: t_R = 1.52 min (A/D 100:0 to 0:100 in 3.5 min, λ = 214 nm). MS (ESI+): $C_{66}H_{128}N_{16}O_{11}$ calc./obs. 1321.99/1322.00 Da $[M+H]^+$.

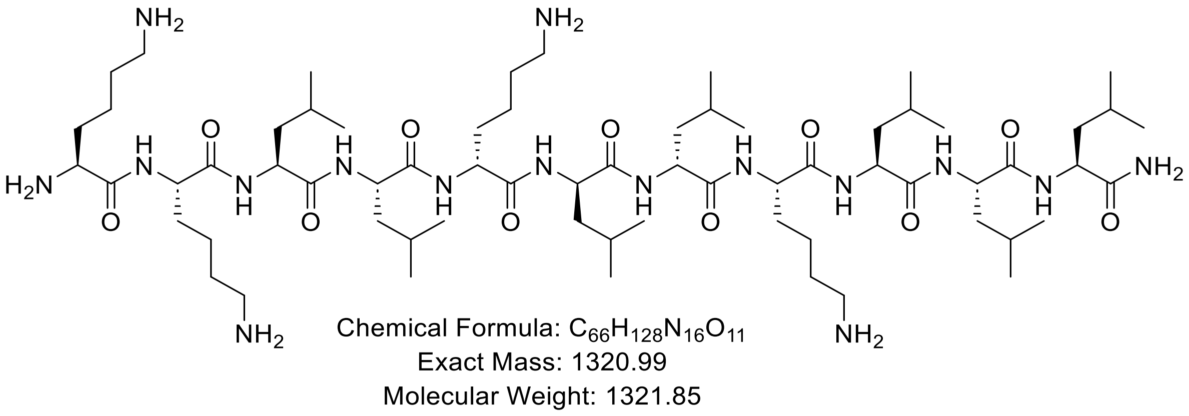

Analytical HPLC-MS data:

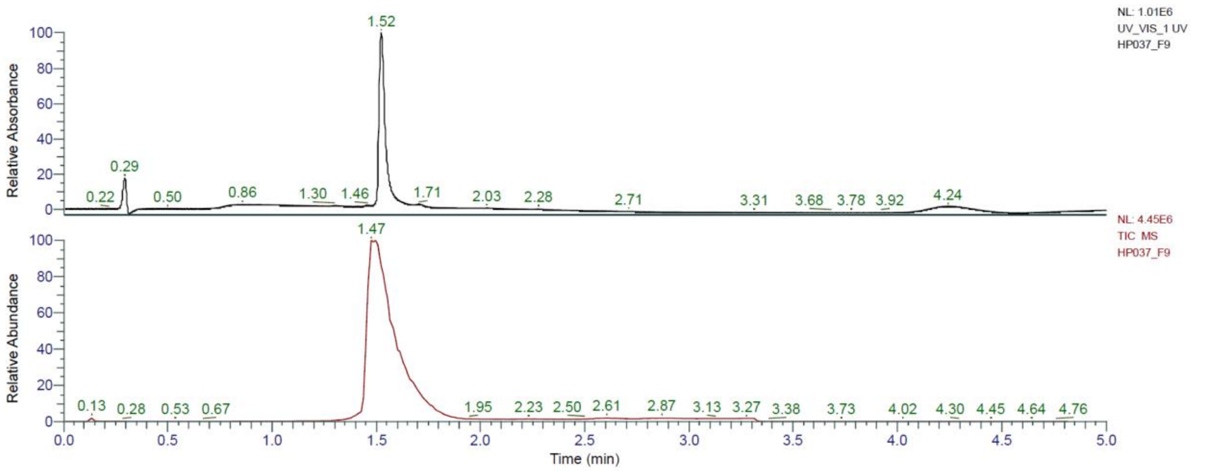

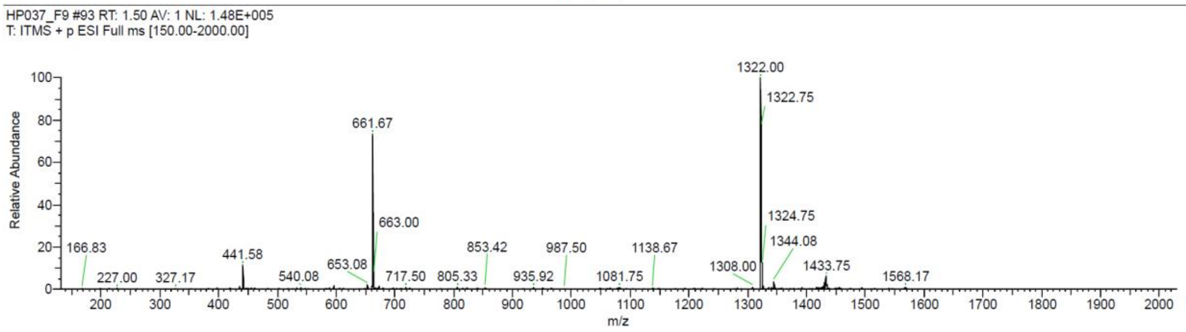

HRMS spectra:

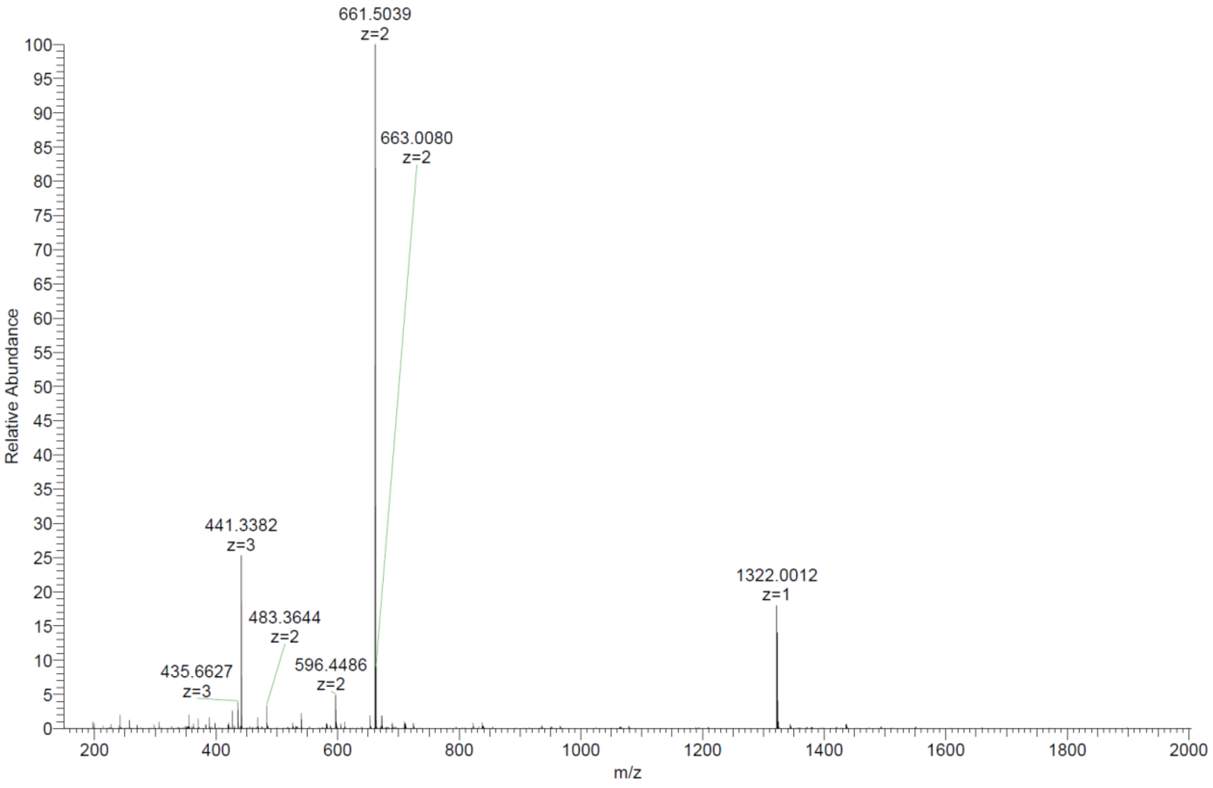

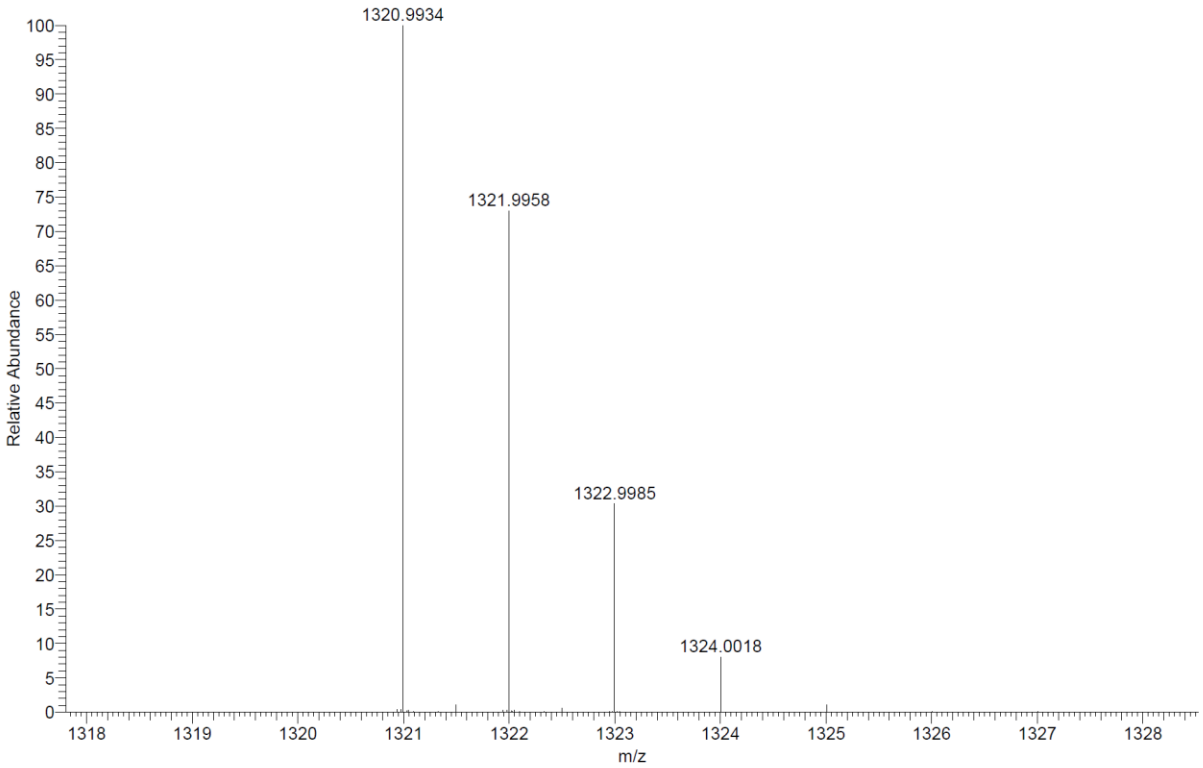

kkLLkLLKLLL (HP10) was obtained as white solid after preparative RP-HPLC (50.0 mg, 45.0%). Analytical RP-HPLC: t_R = 1.60 min (A/D 100:0 to 0:100 in 3.5 min, λ = 214 nm). MS (ESI+): $C_{66}H_{128}N_{16}O_{11}$ calc./obs. 1321.99/1322.00 Da $[M+H]^+$.

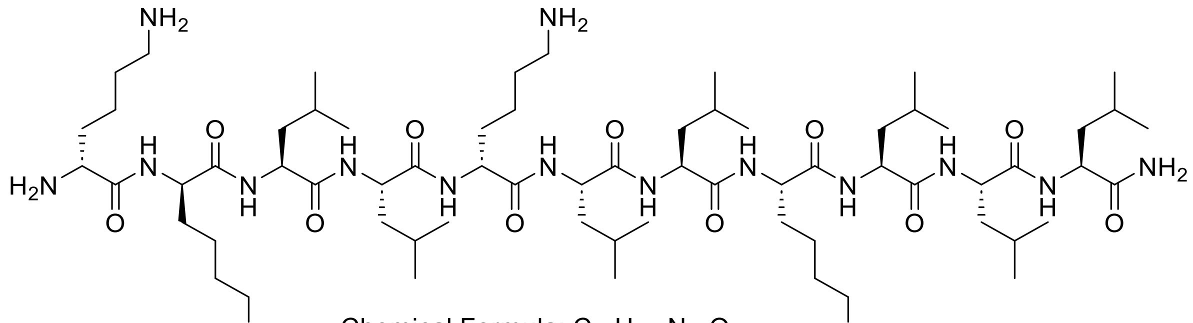

Analytical HPLC-MS data:

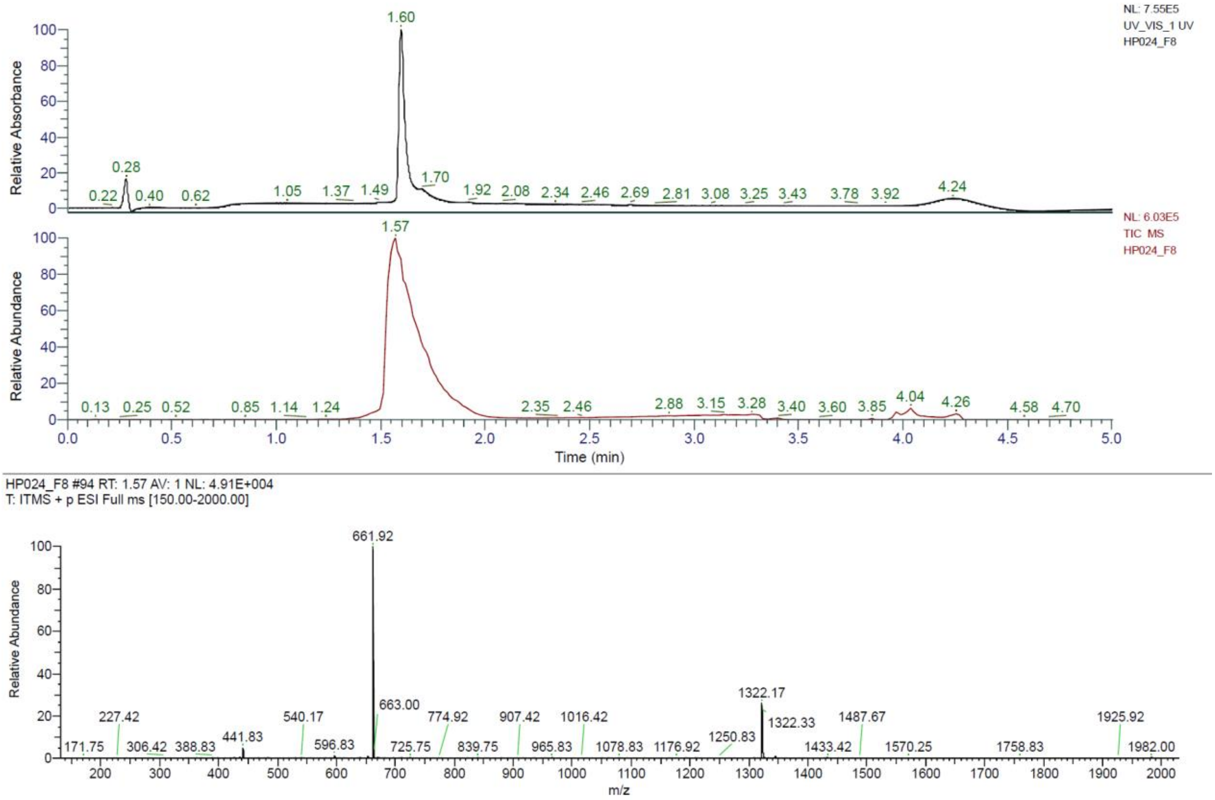

HRMS spectra:

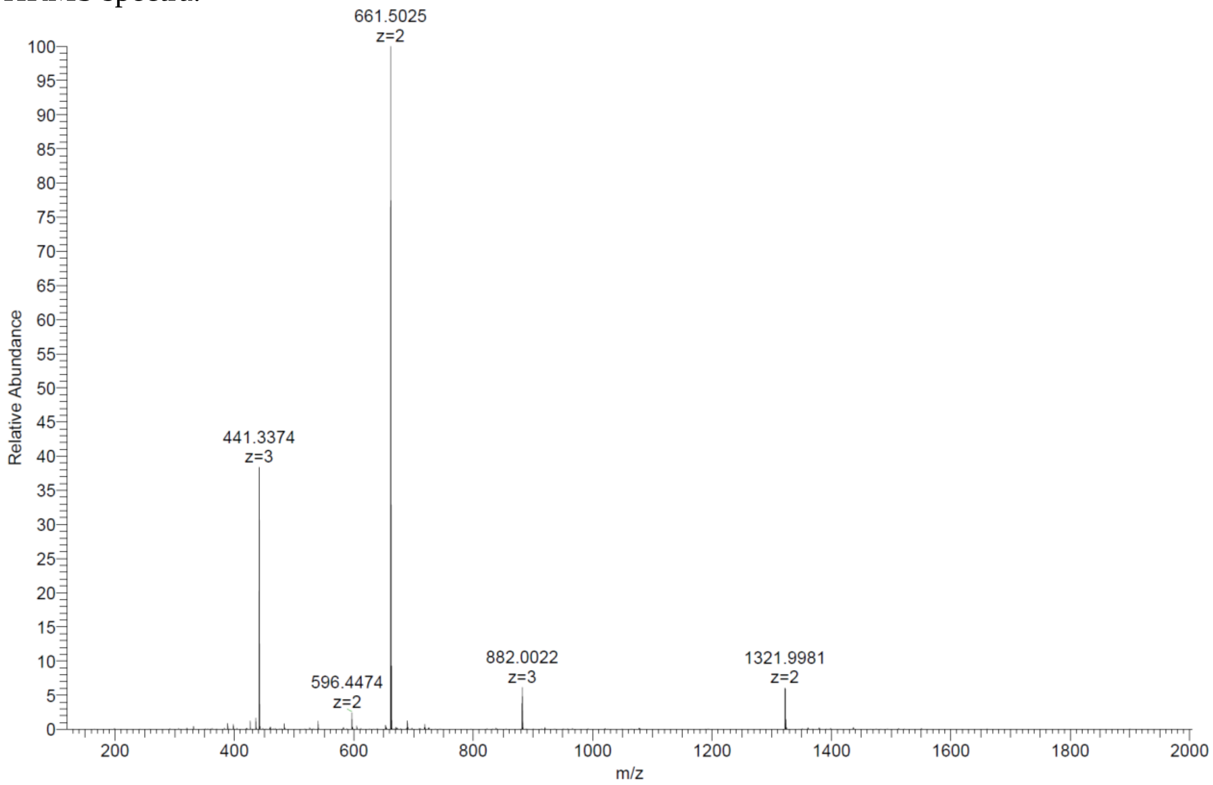

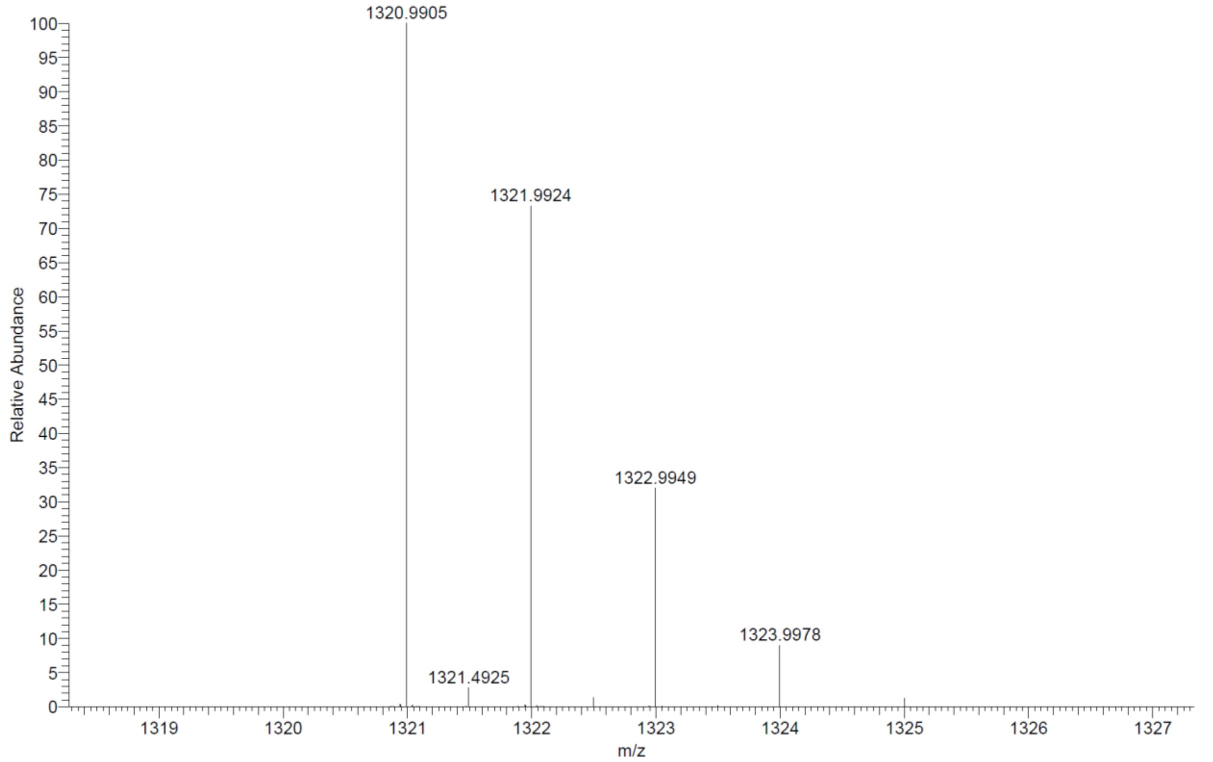

KkllKLlKLLL (HP11) was obtained as white solid after preparative RP-HPLC (31.5 mg, 35.4%). Analytical RP-HPLC: t_R = 1.60 min (A/D 100:0 to 0:100 in 3.5 min, λ = 214 nm). MS (ESI+): $C_{66}H_{128}N_{16}O_{11}$ calc./obs. 1321.99/1322.00 Da $[M+H]^+$.

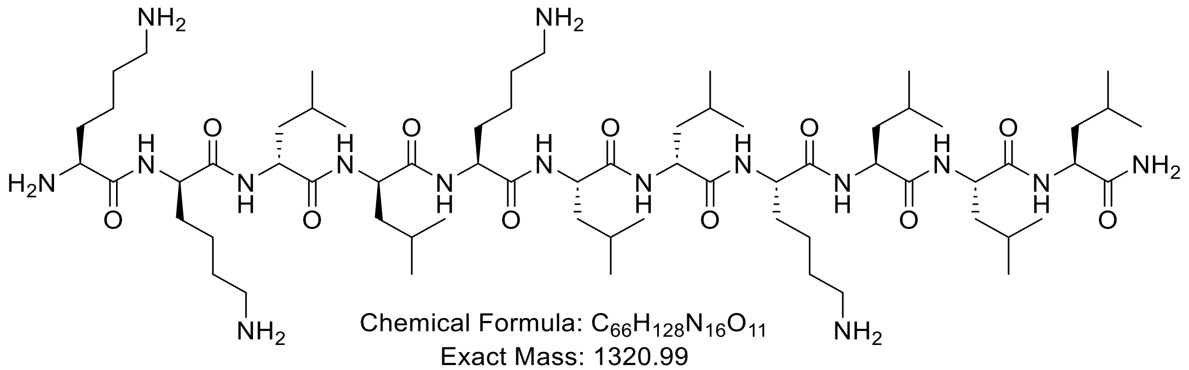

Chemical Formula: $C_{66}H_{128}N_{16}O_{11}$
Exact Mass: 1320.99
Molecular Weight: 1321.85

Analytical HPLC-MS data:

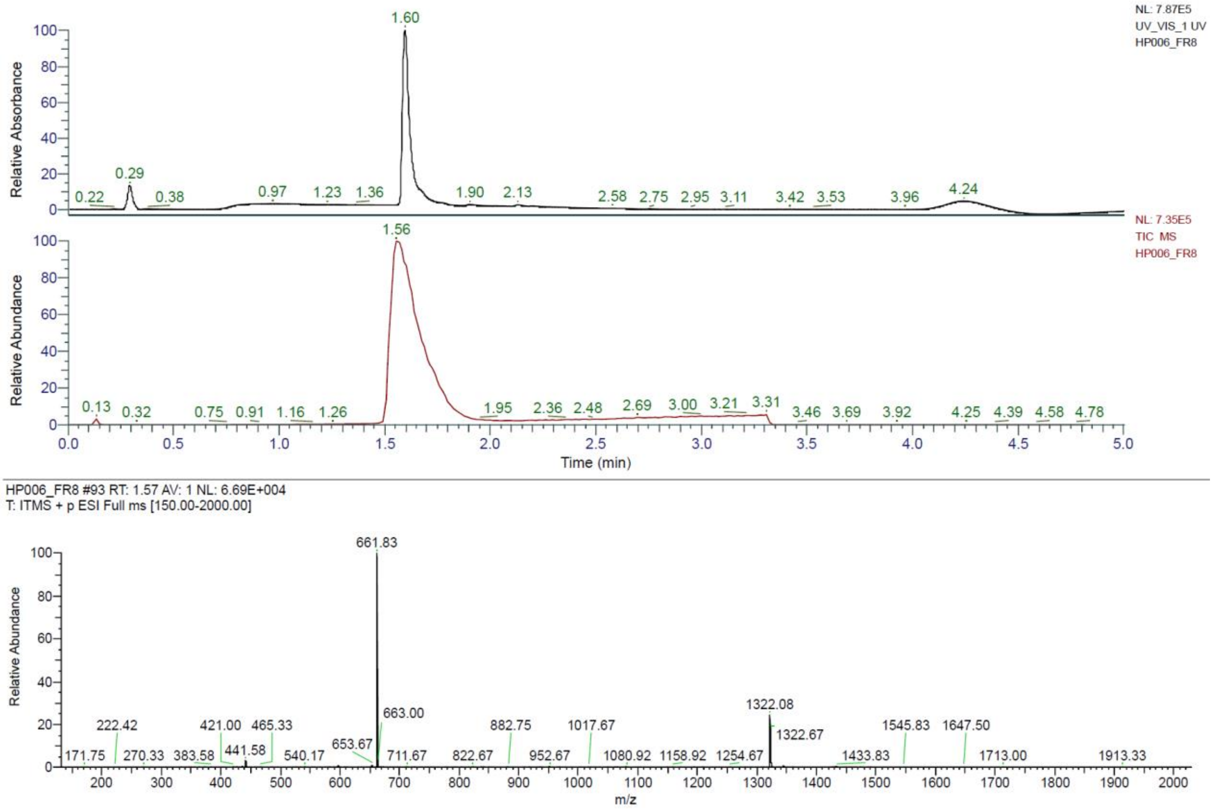

HRMS spectra:

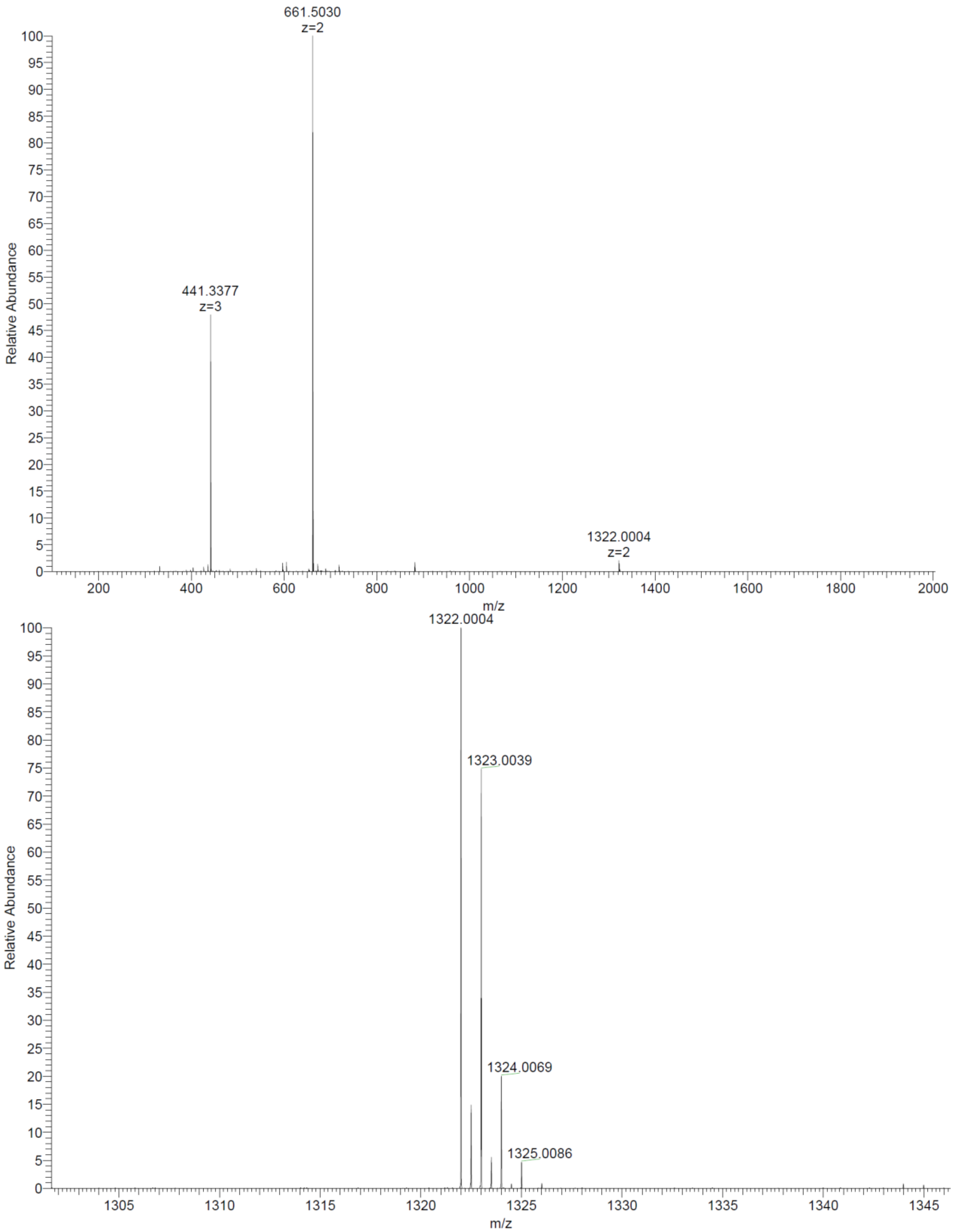
661.5030
z=2
441.3377
z=3
1322.0004
z=2
Relative Abundance
m/z
1322.0004
1323.0039
1324.0069
1325.0086
Relative Abundance
m/z

KkllKlLKLLL (HP12) was obtained as white solid after preparative RP-HPLC (26.8 mg, 30.1%). Analytical RP-HPLC: t_R = 1.60 min (A/D 100:0 to 0:100 in 3.5 min, λ = 214 nm). MS (ESI+): $C_{66}H_{128}N_{16}O_{11}$ calc./obs. 1321.99/1322.00 Da $[M+H]^+$.

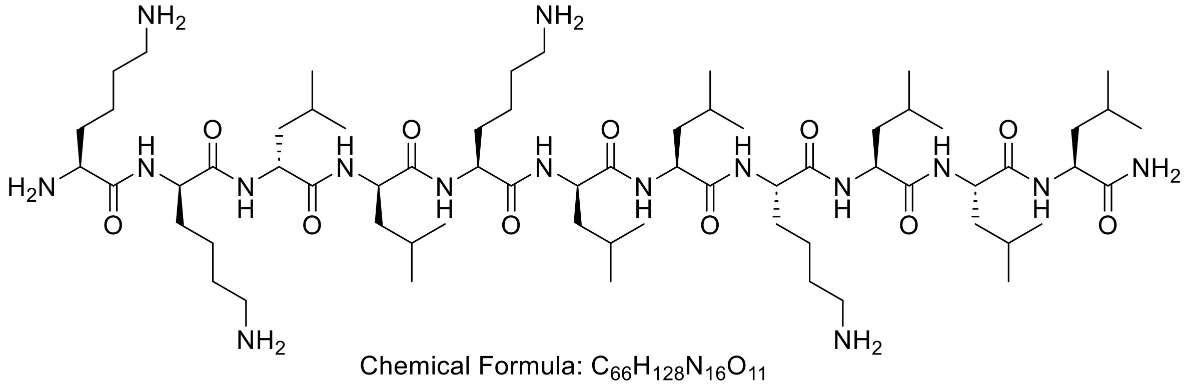

Chemical Formula: $C_{66}H_{128}N_{16}O_{11}$
Exact Mass: 1320.99
Molecular Weight: 1321.85

Analytical HPLC-MS data:

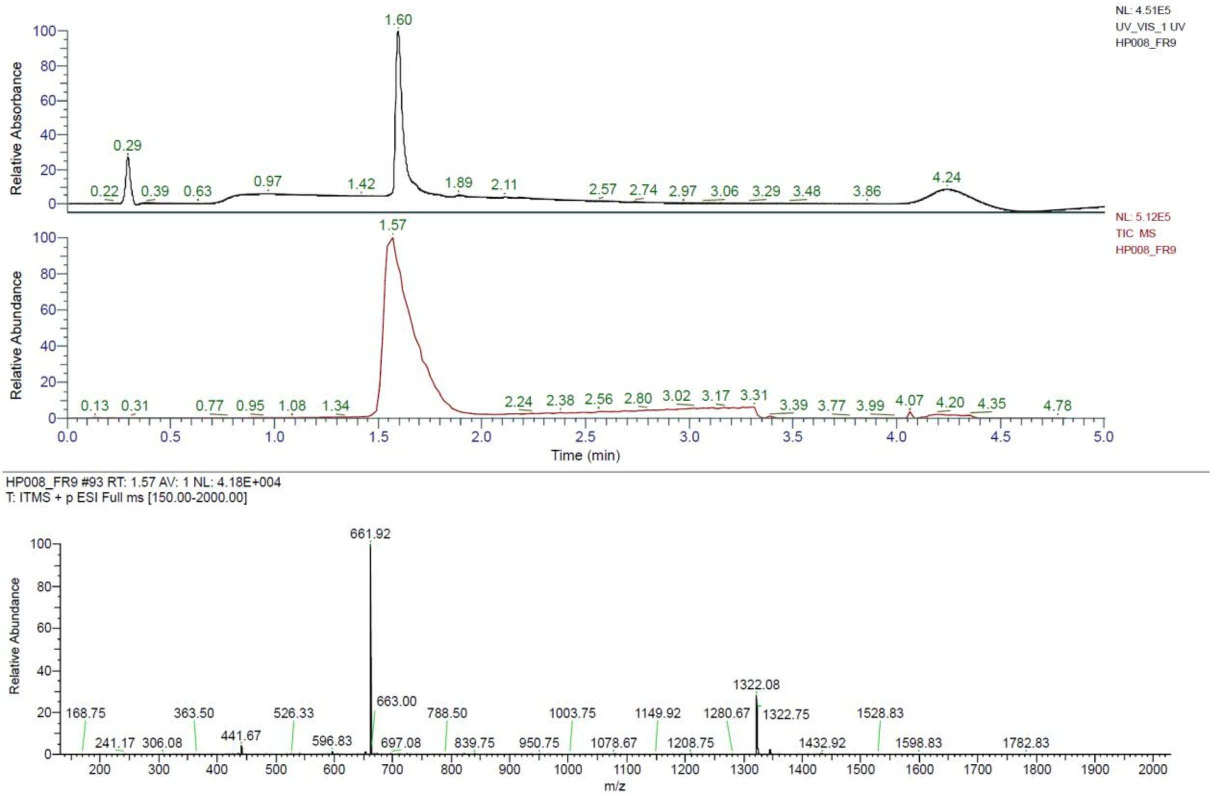

HRMS spectra:

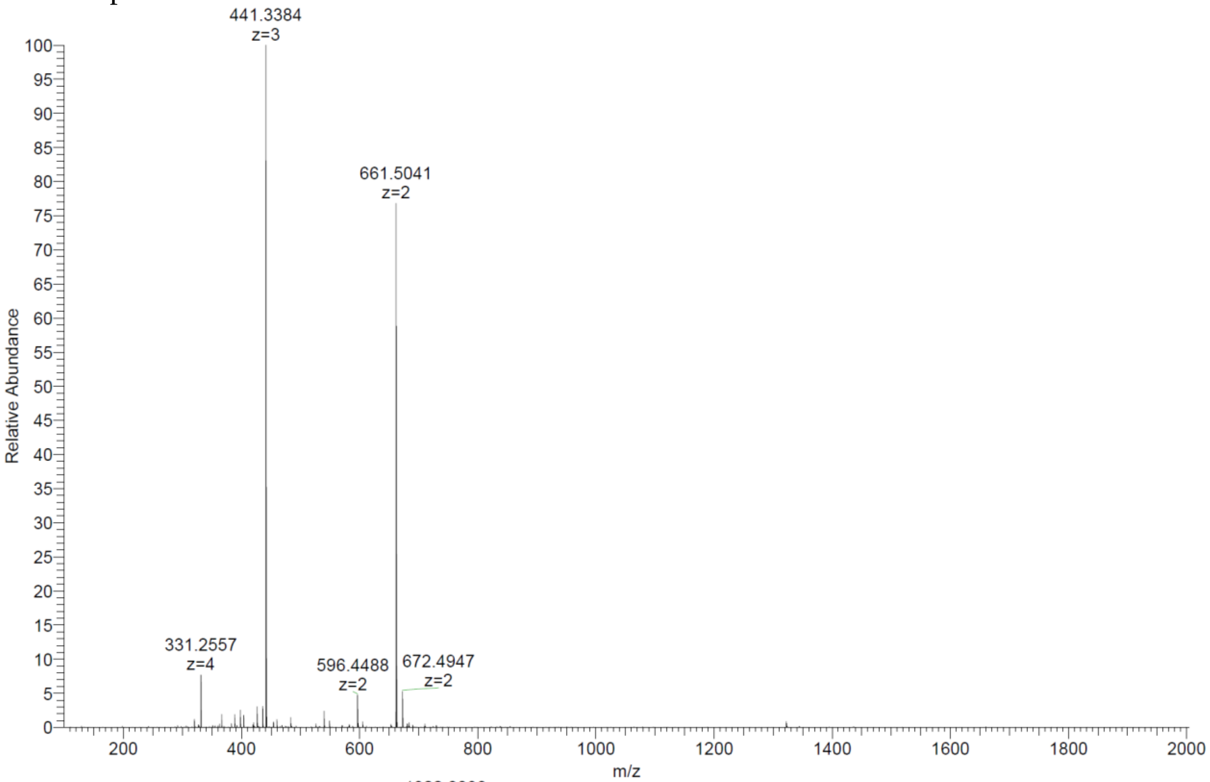

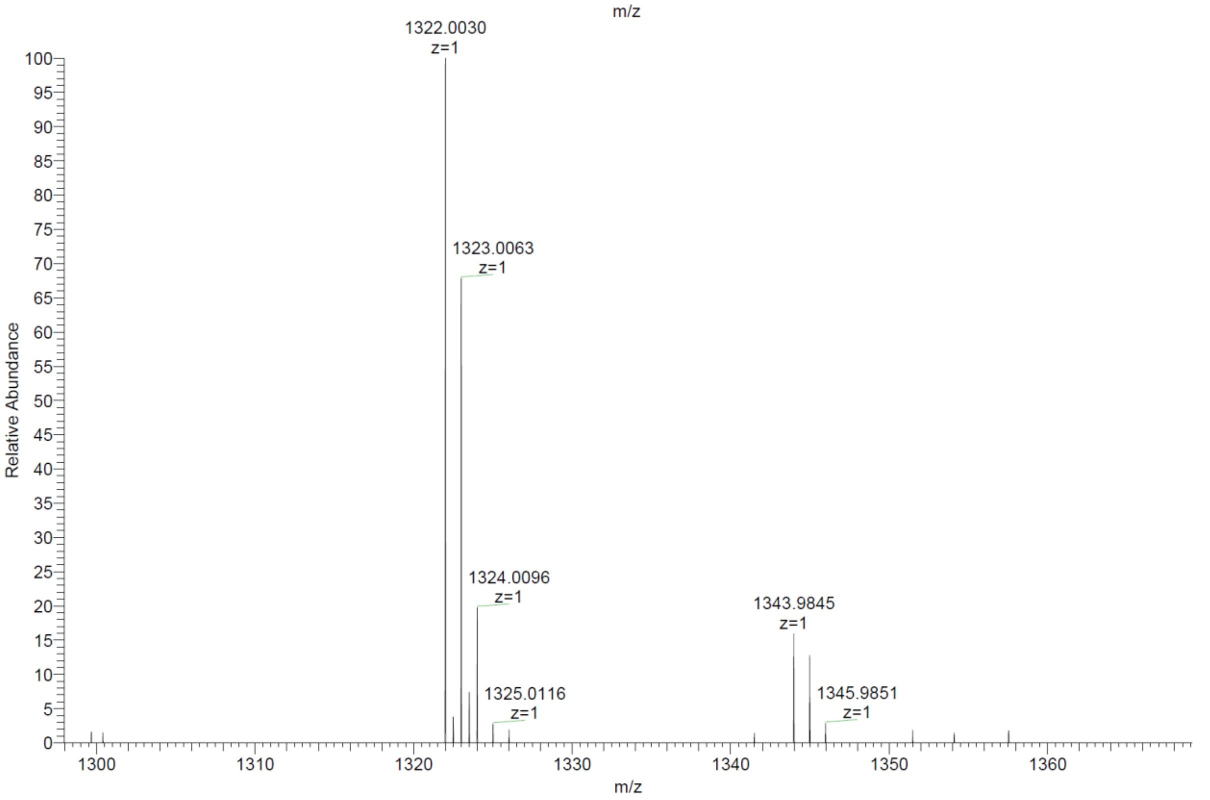

KKLLkllkLLL (HP13) was obtained as white solid after preparative RP-HPLC (37.4 mg, 42.1%). Analytical RP-HPLC: t_R = 1.53 min (A/D 100:0 to 0:100 in 3.5 min, λ = 214 nm). MS (ESI+): $C_{66}H_{128}N_{16}O_{11}$ calc./obs. 1321.99/1322.00 Da $[M+H]^+$.

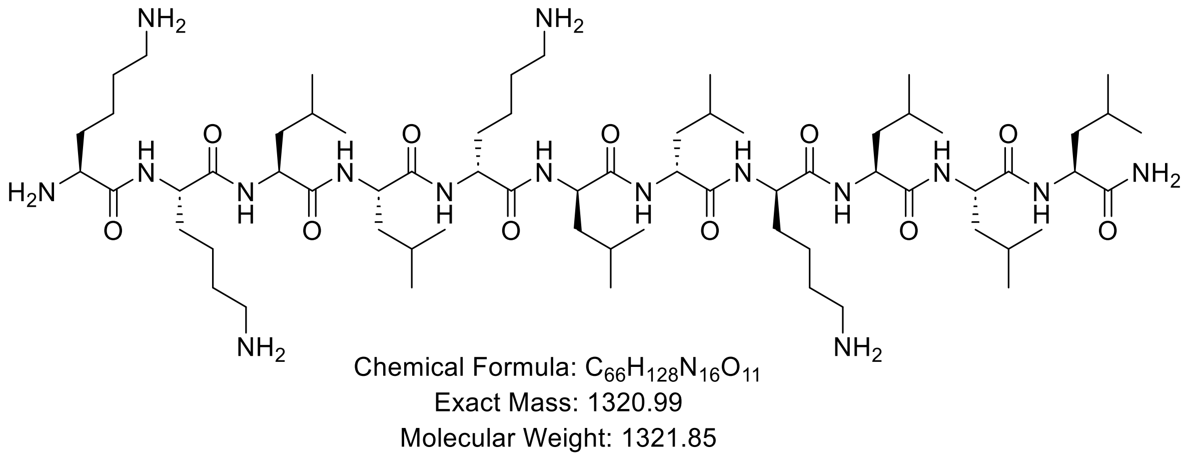

Analytical HPLC-MS data:

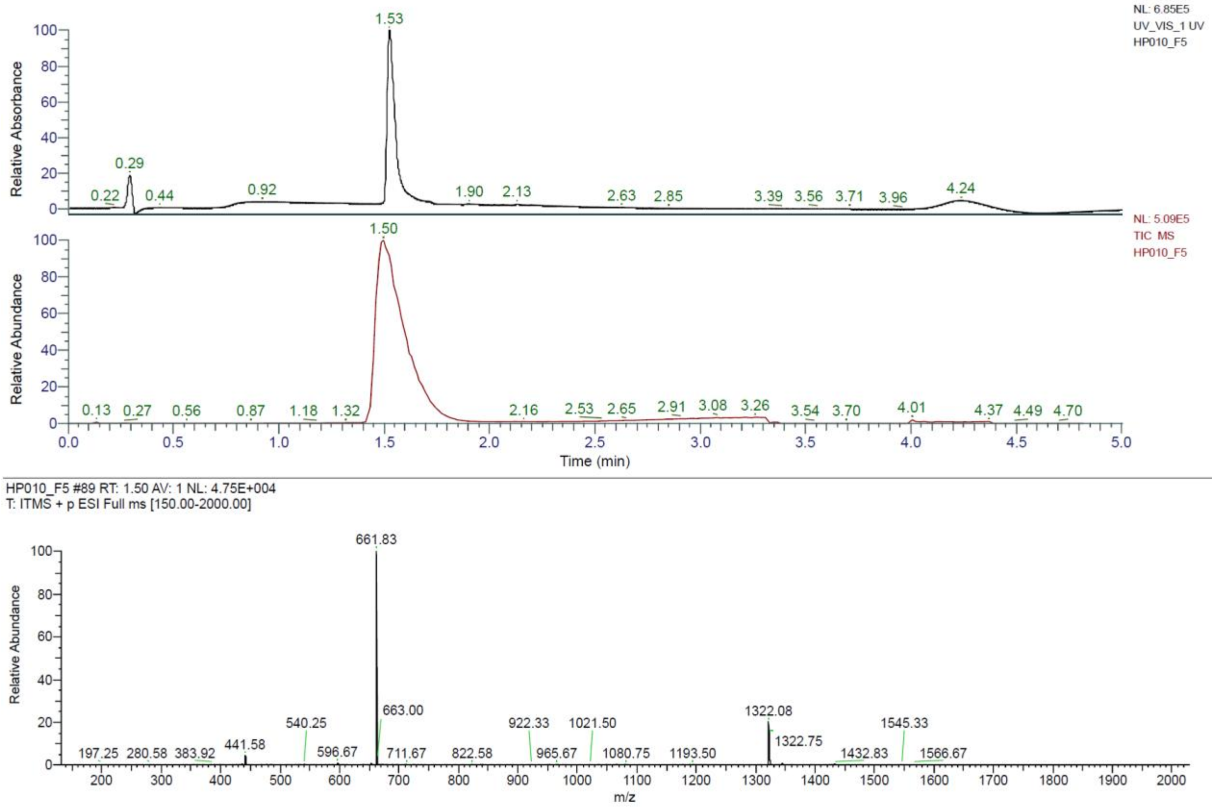

HRMS spectra:

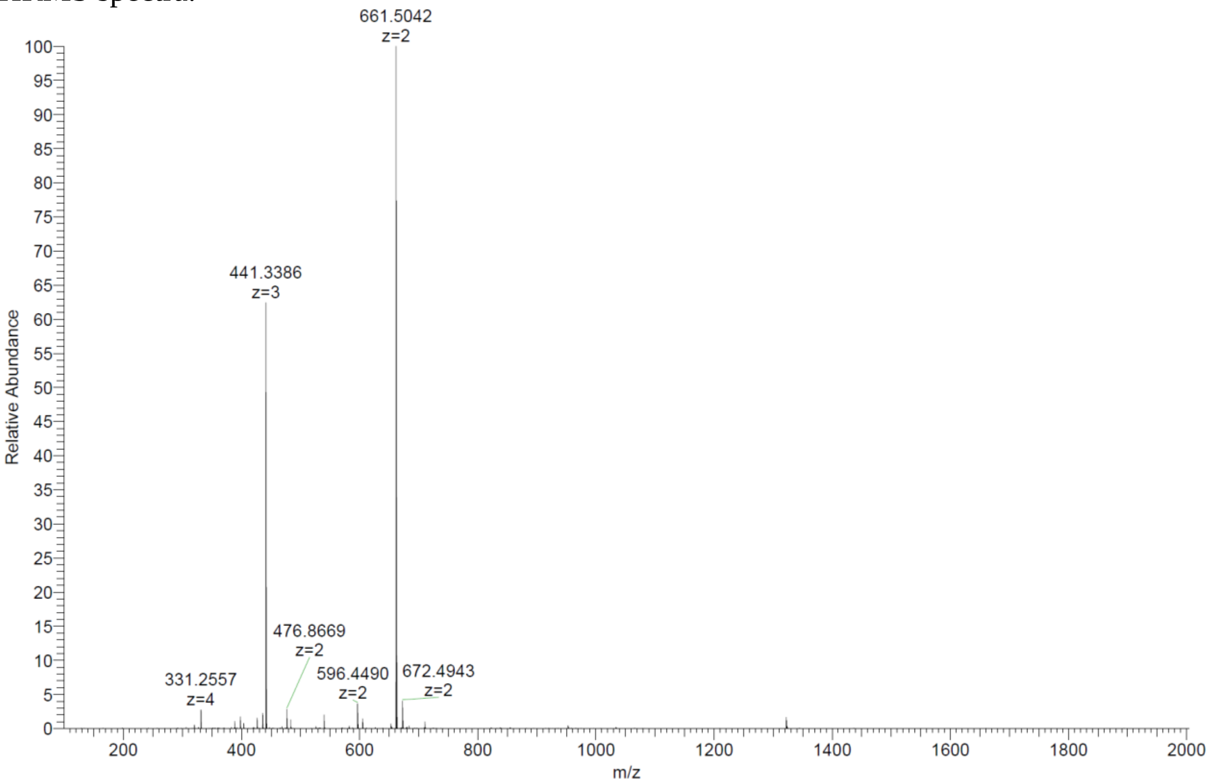

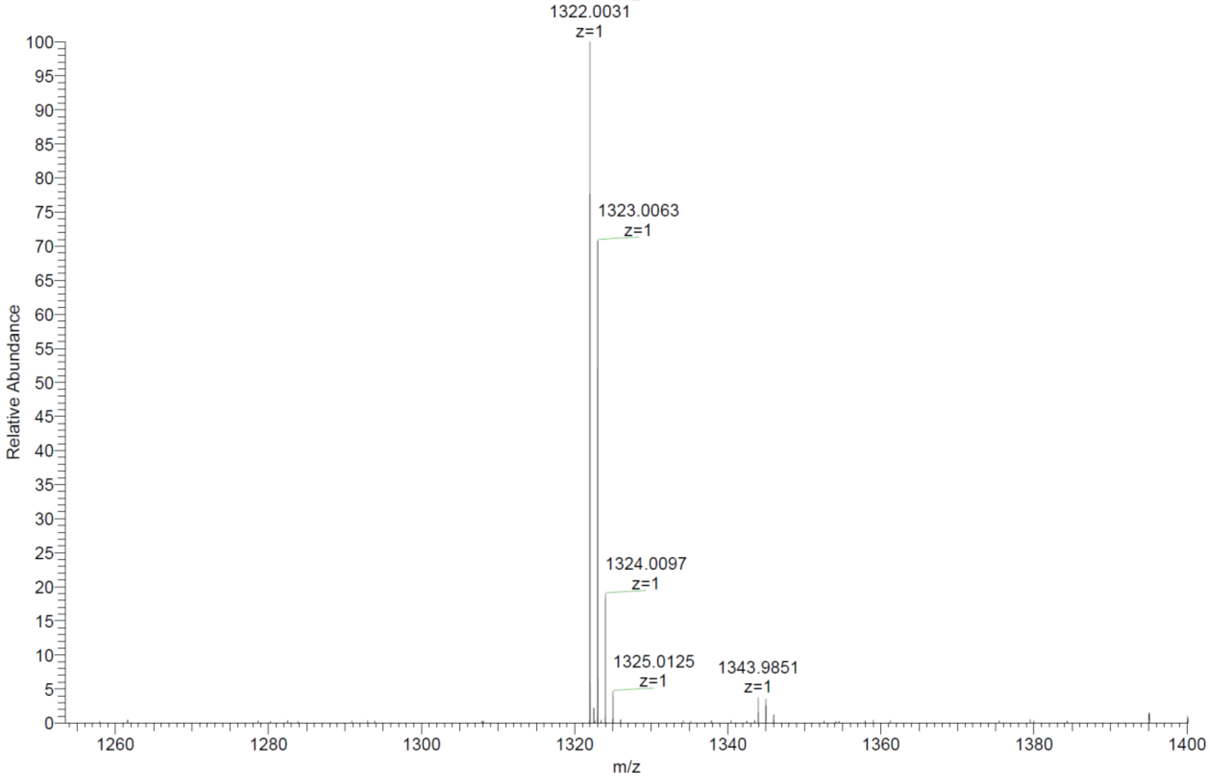

KKllKllKLLL (HP14) was obtained as white solid after preparative RP-HPLC (33.7 mg, 37.9%). Analytical RP-HPLC: t_R = 1.53 min (A/D 100:0 to 0:100 in 3.5 min, λ = 214 nm). MS (ESI+): $C_{66}H_{128}N_{16}O_{11}$ calc./obs. 1321.99/1322.00 Da $[M+H]^+$.

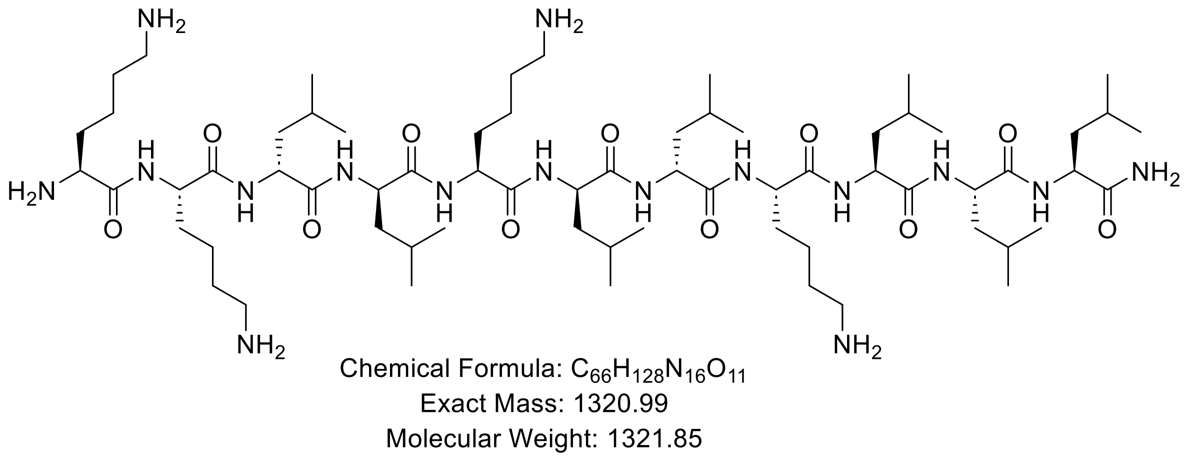

Chemical Formula: $C_{66}H_{128}N_{16}O_{11}$

Exact Mass: 1320.99

Molecular Weight: 1321.85

Analytical HPLC-MS data:

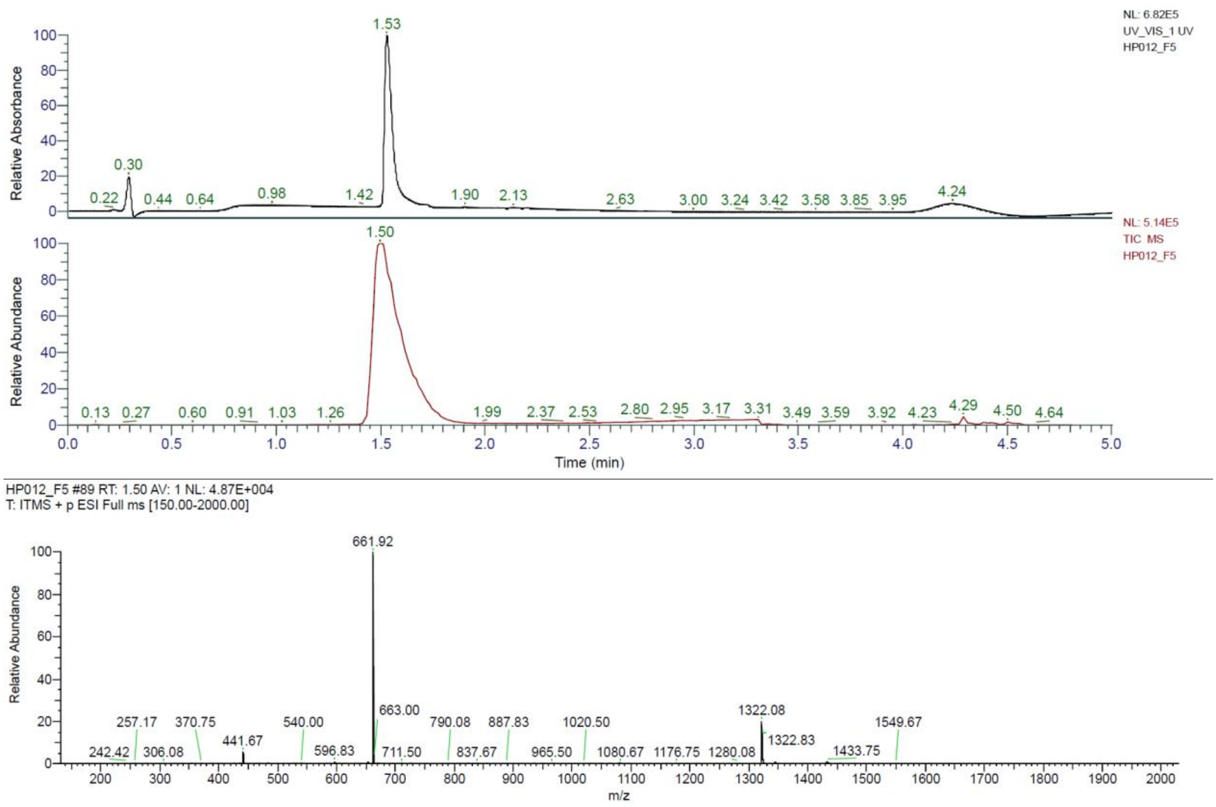

HRMS spectra:

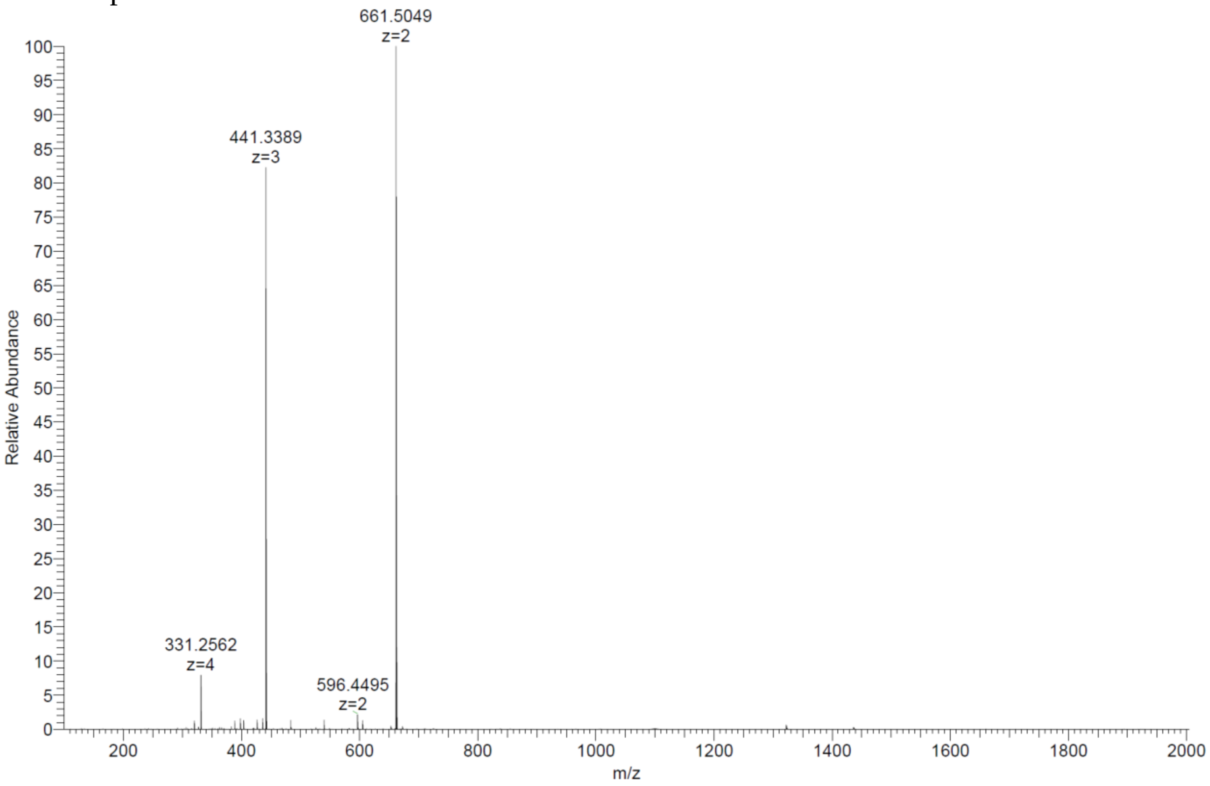

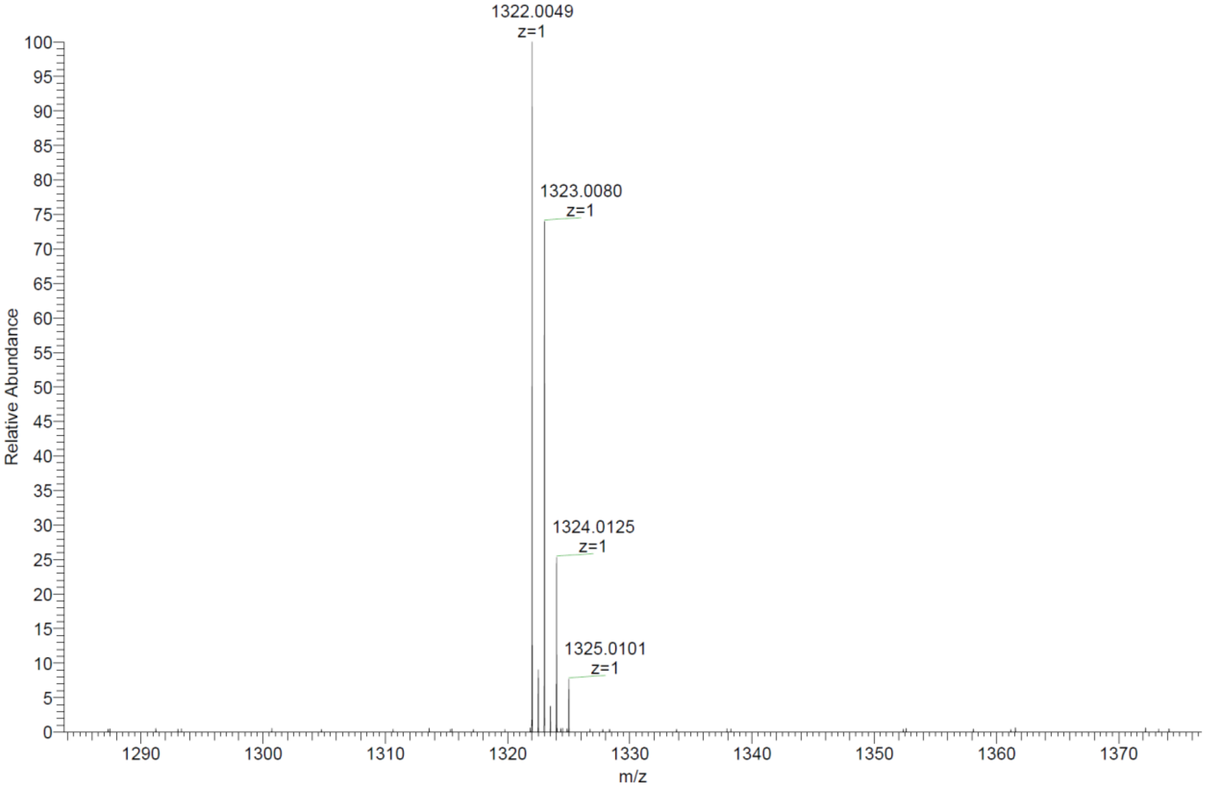

KKlKlLkLlLL (HP15) was obtained as white solid after preparative RP-HPLC (34.5 mg, 38.8%). Analytical RP-HPLC: t_R = 1.60 min (A/D 100:0 to 0:100 in 3.5 min, λ = 214 nm). MS (ESI+): $C_{66}H_{128}N_{16}O_{11}$ calc./obs. 1321.99/1322.00 Da $[M+H]^+$.

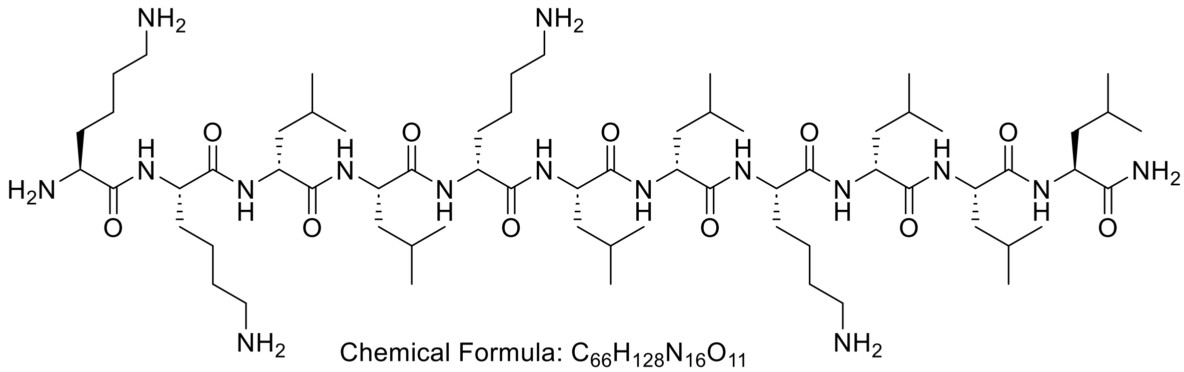

Chemical Formula: $C_{66}H_{128}N_{16}O_{11}$
Exact Mass: 1320.99
Molecular Weight: 1321.85

Analytical HPLC-MS data:

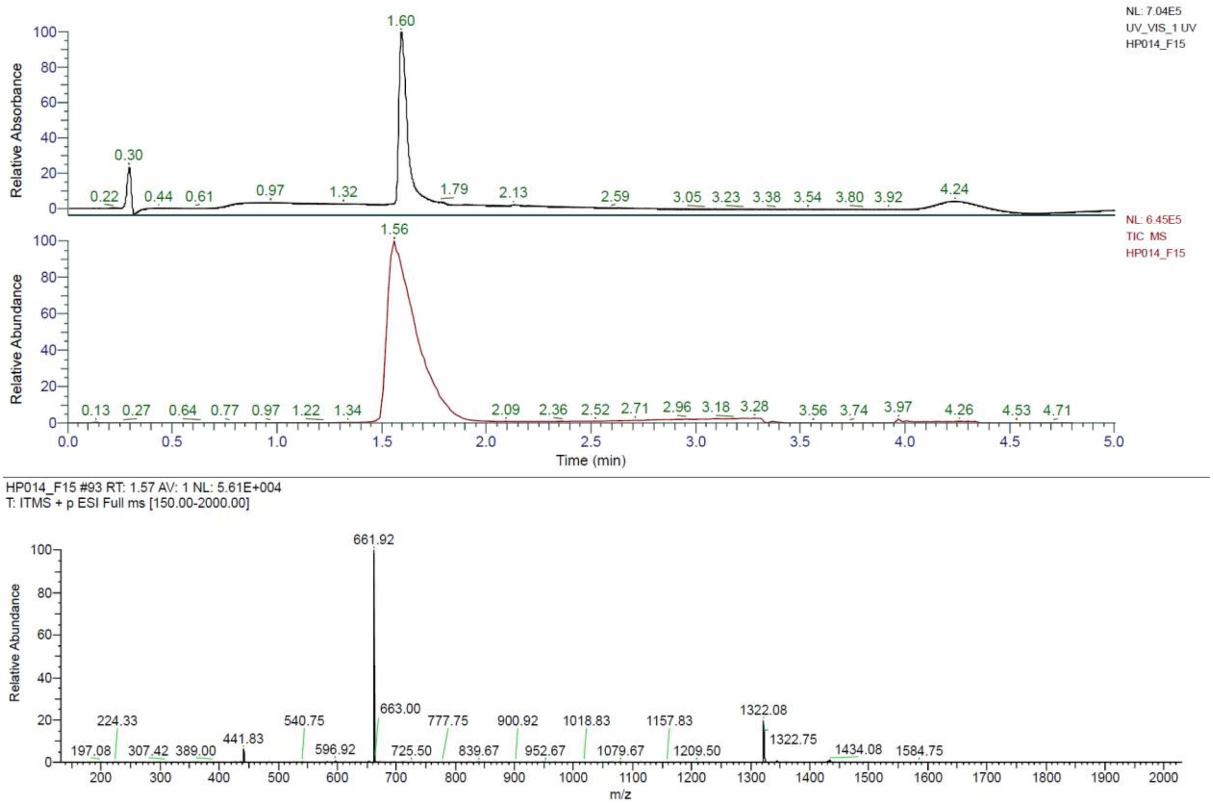

HRMS spectra:

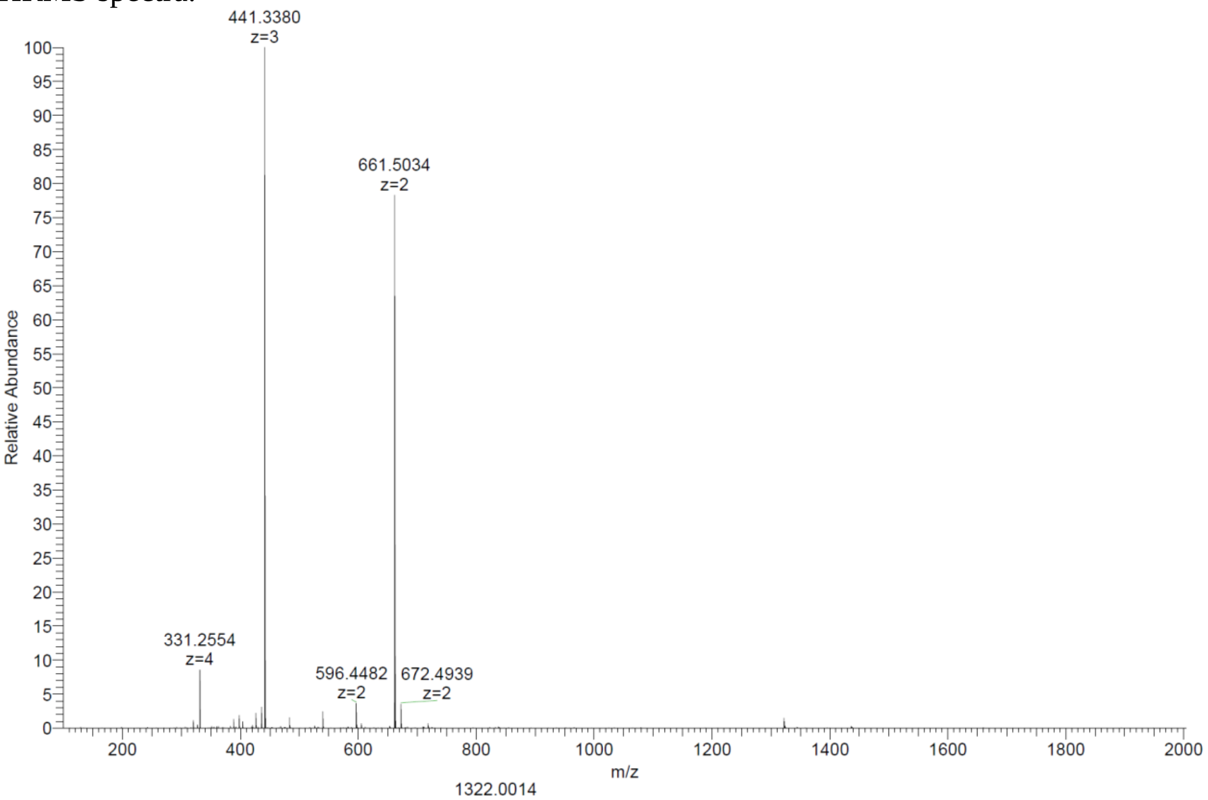

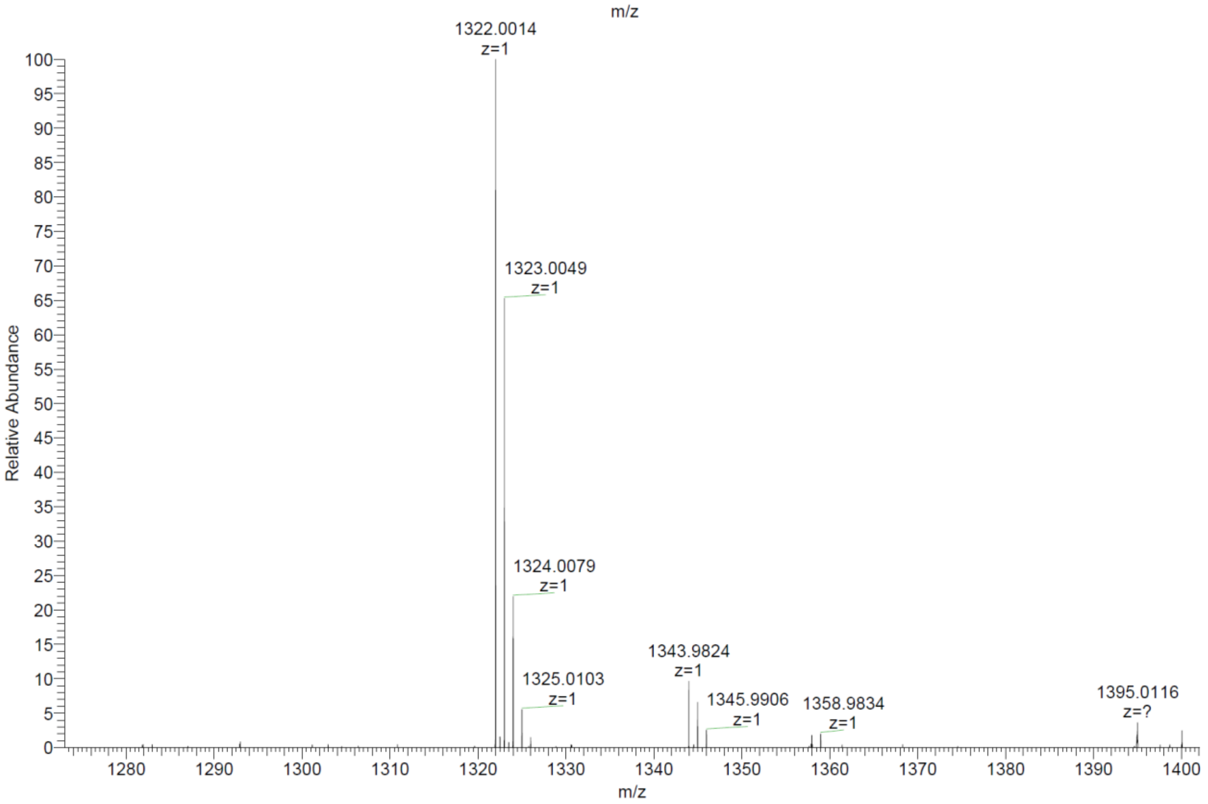

KKLlkLLklLL (HP16) was obtained as white solid after preparative RP-HPLC (16.0 mg, 18.0%). Analytical RP-HPLC: t_R = 1.59 min (A/D 100:0 to 0:100 in 3.5 min, λ = 214 nm). MS (ESI+): $C_{66}H_{128}N_{16}O_{11}$ calc./obs. 1321.99/1322.00 Da $[M+H]^+$.

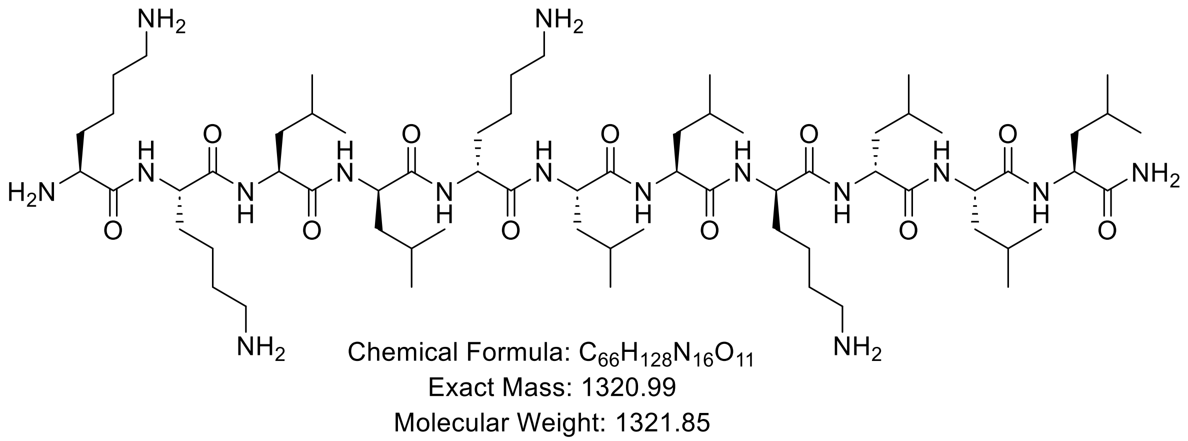

Chemical Formula: $C_{66}H_{128}N_{16}O_{11}$
Exact Mass: 1320.99
Molecular Weight: 1321.85

Analytical HPLC-MS data:

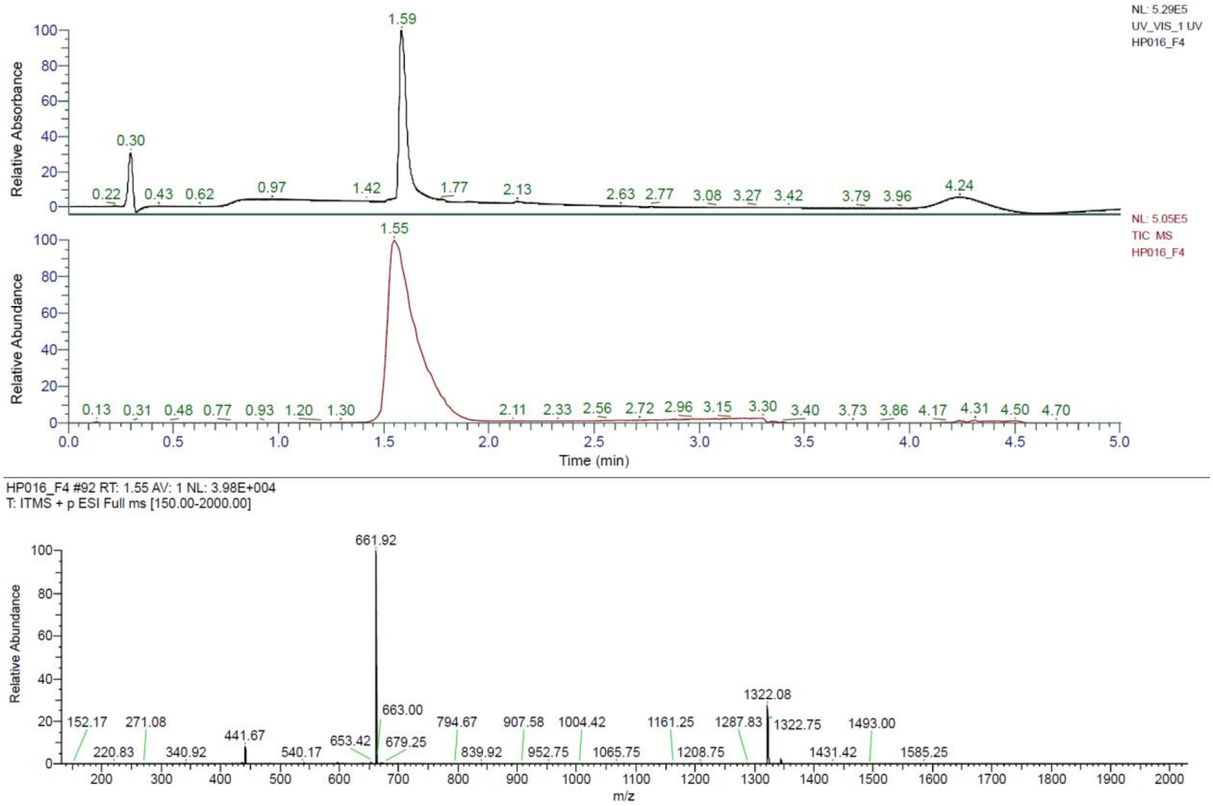

HRMS spectra:

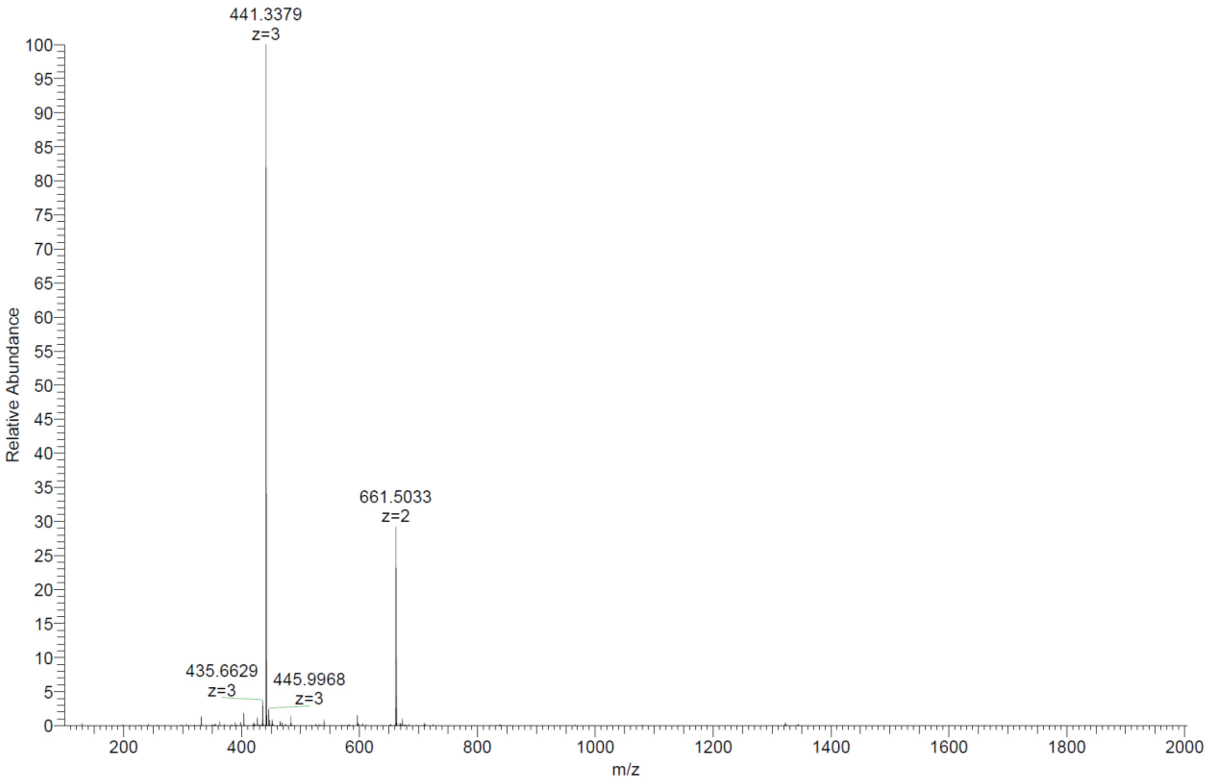

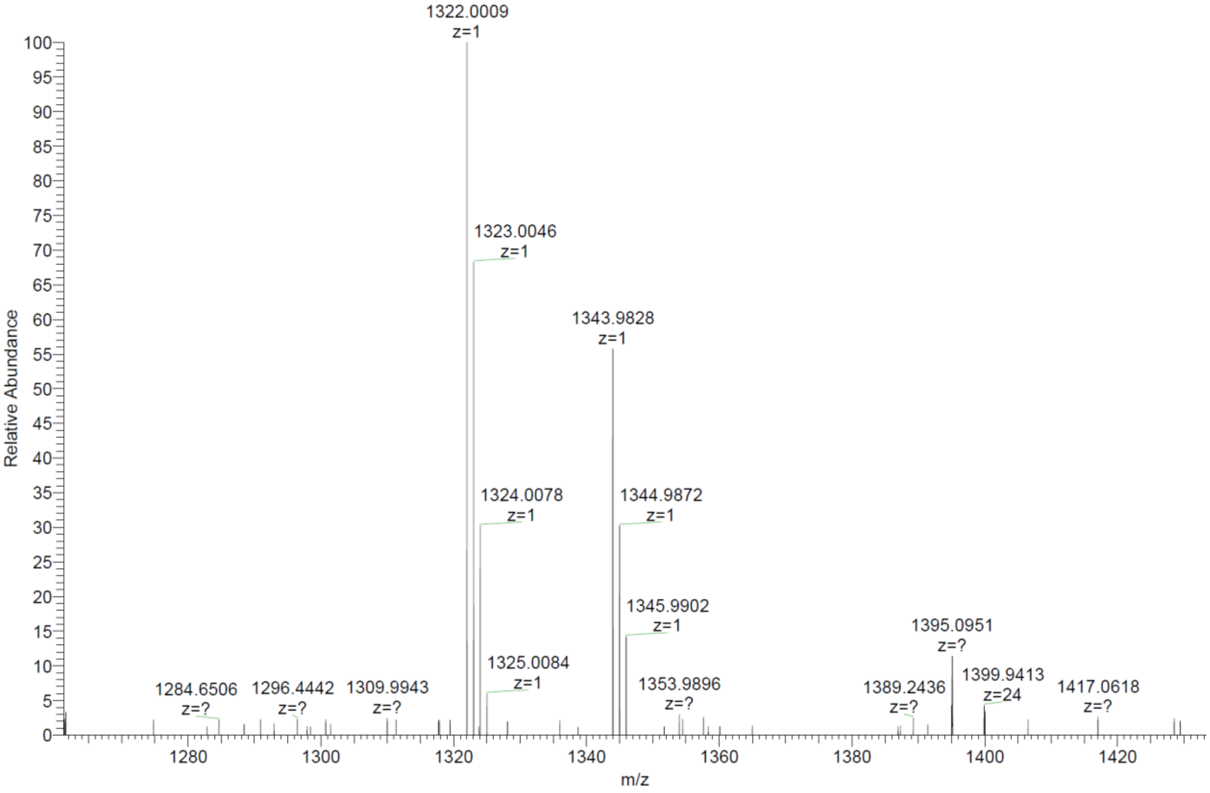

KklLKLLKllL (HP17) was obtained as white solid after preparative RP-HPLC (29.0 mg, 26.1%). Analytical RP-HPLC: t_R = 1.61 min (A/D 100:0 to 0:100 in 3.5 min, λ = 214 nm). MS (ESI+): $C_{66}H_{128}N_{16}O_{11}$ calc./obs. 1321.99/1322.00 Da $[M+H]^+$.

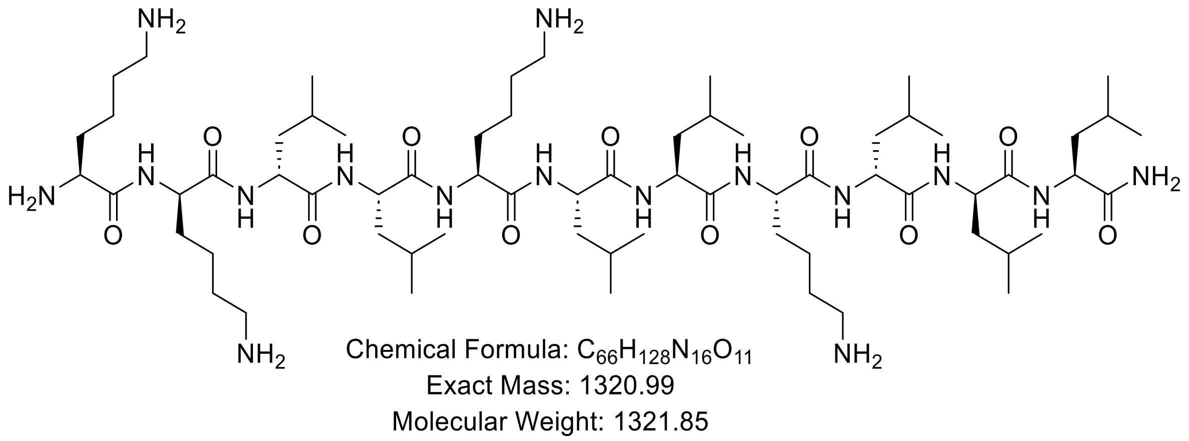

Analytical HPLC-MS data:

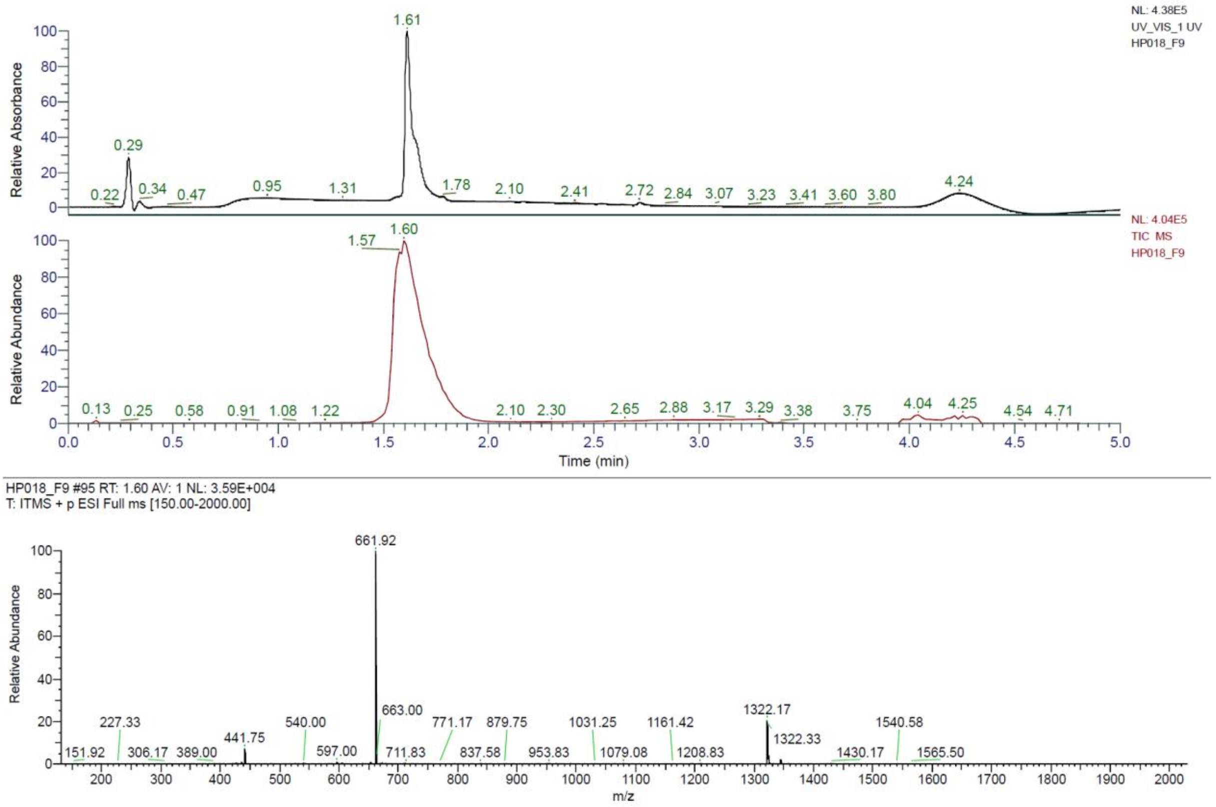

HRMS spectra:

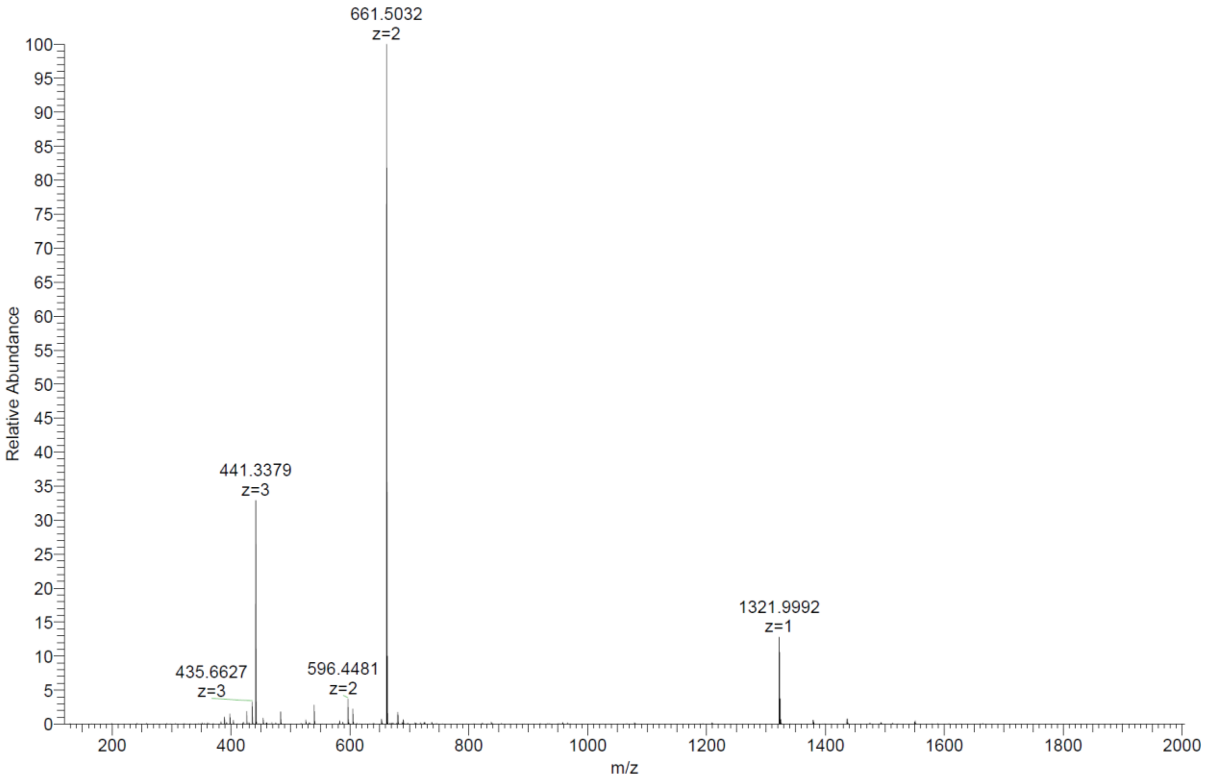

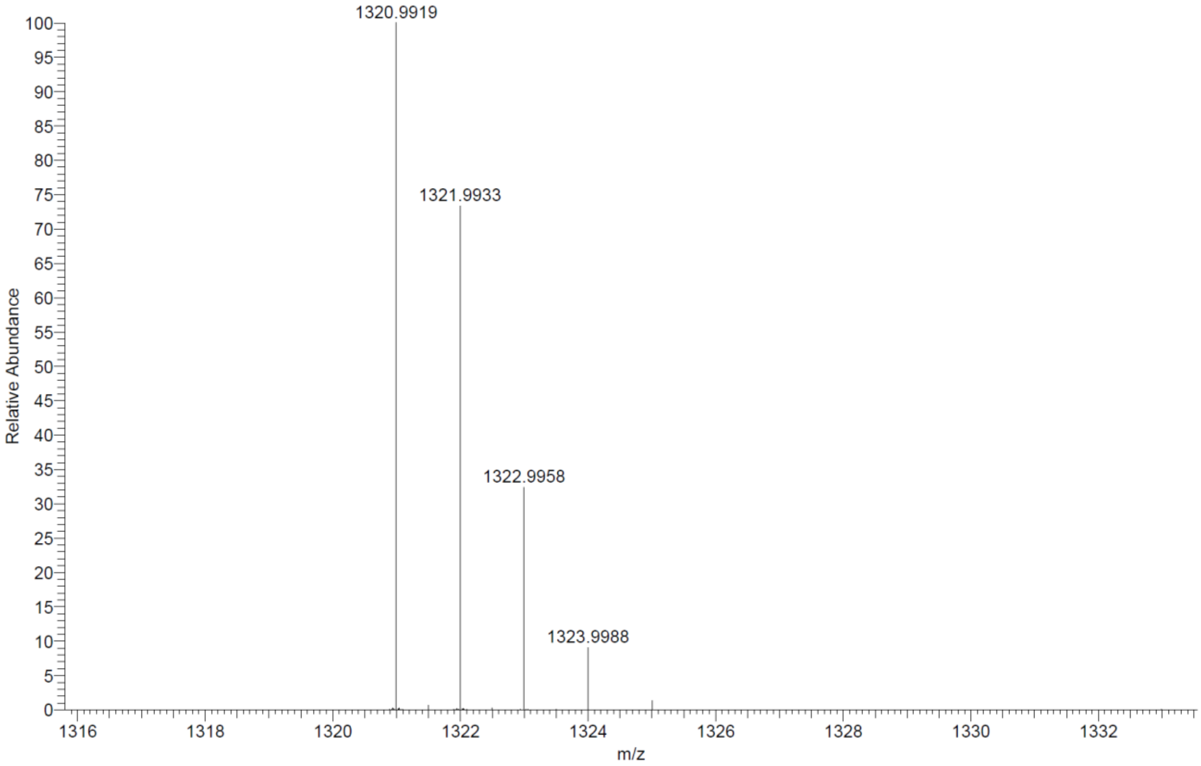

kkLLKLLKLll (HP18) was obtained as white solid after preparative RP-HPLC (36.3 mg, 32.7%). Analytical RP-HPLC: t_R = 1.58 min (A/D 100:0 to 0:100 in 3.5 min, λ = 214 nm). MS (ESI+): $C_{66}H_{128}N_{16}O_{11}$ calc./obs. 1320.99/1320.99 Da [M].

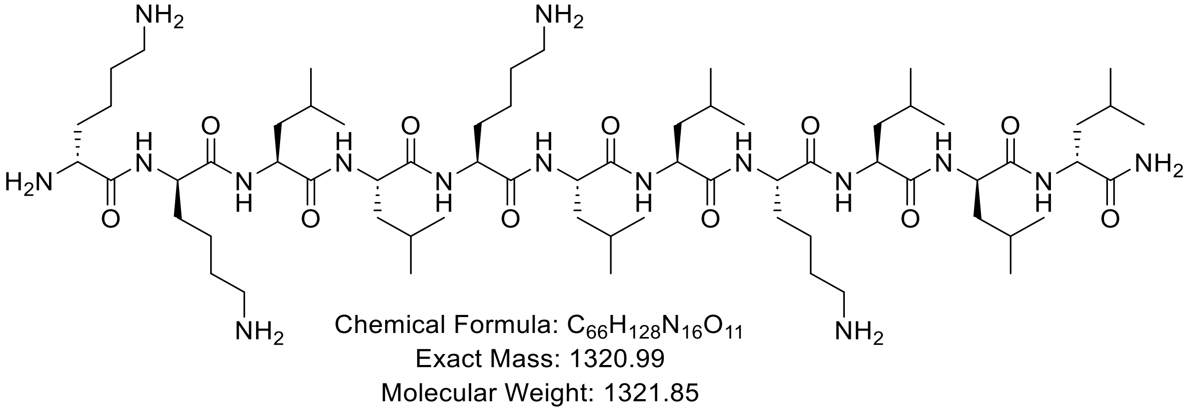

Analytical HPLC-MS data:

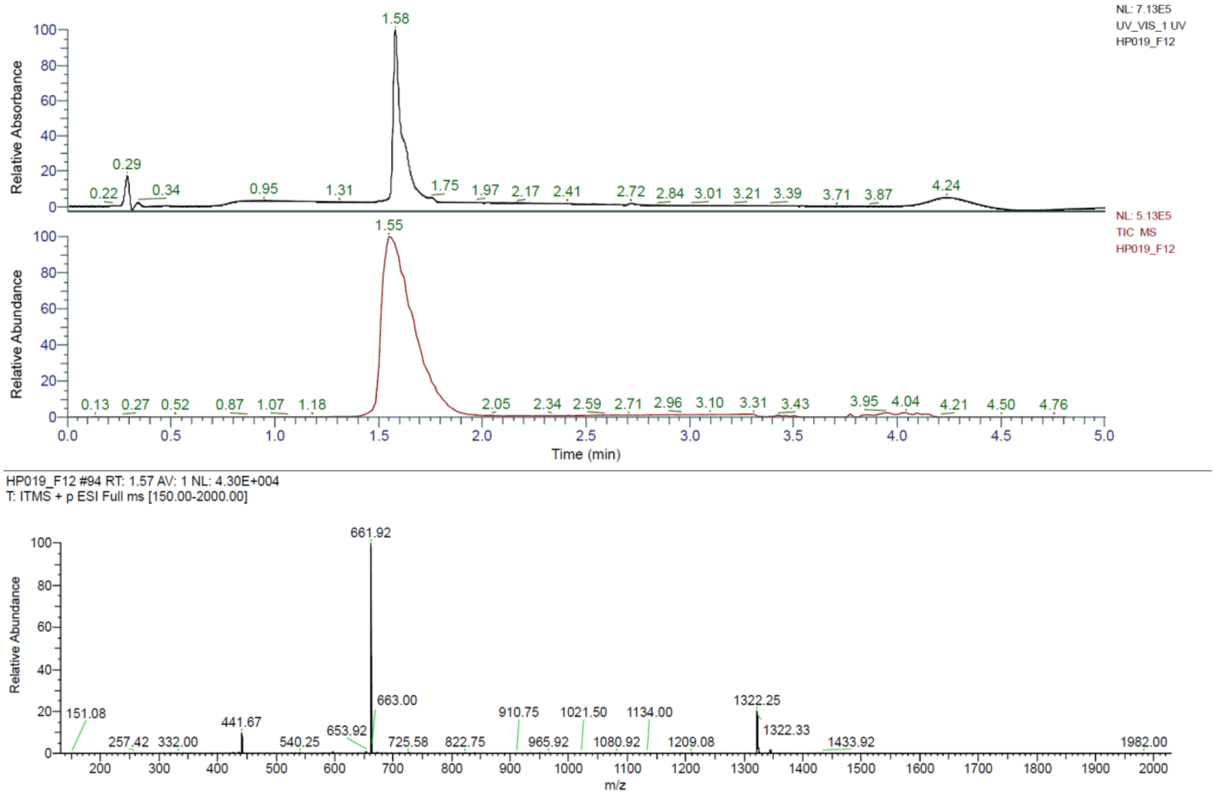

HRMS spectra:

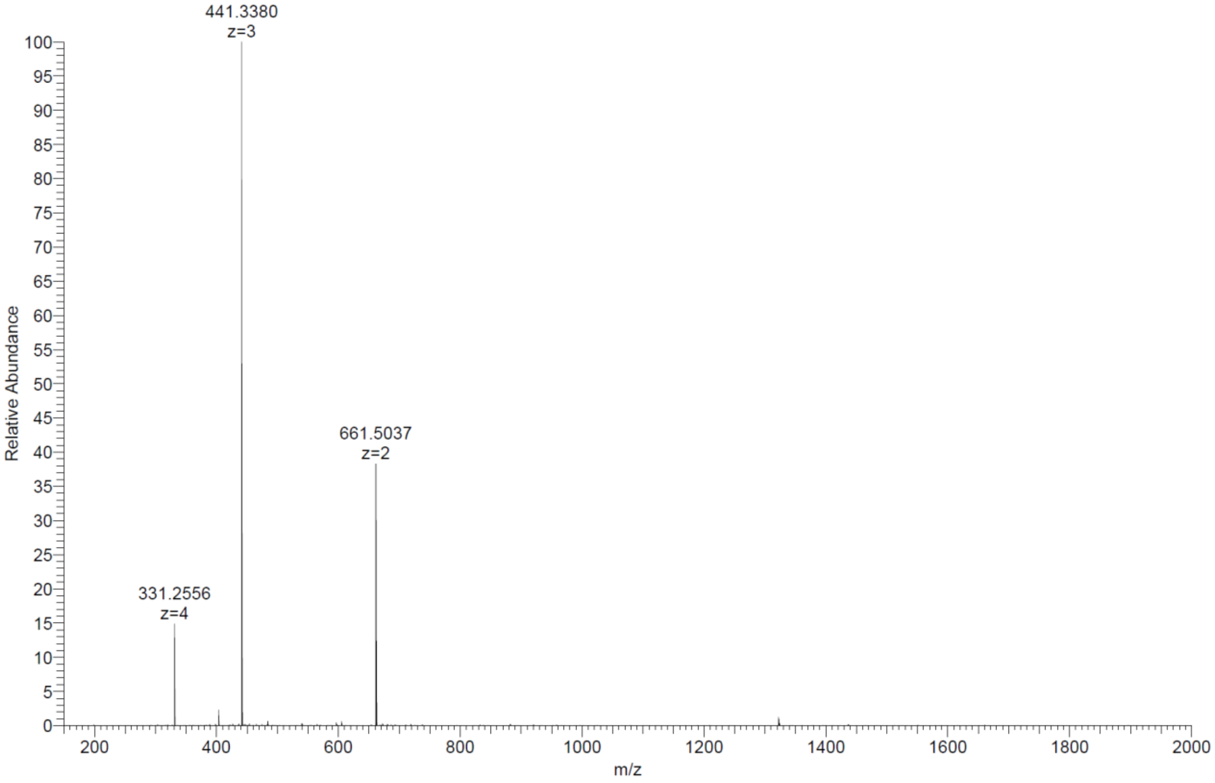

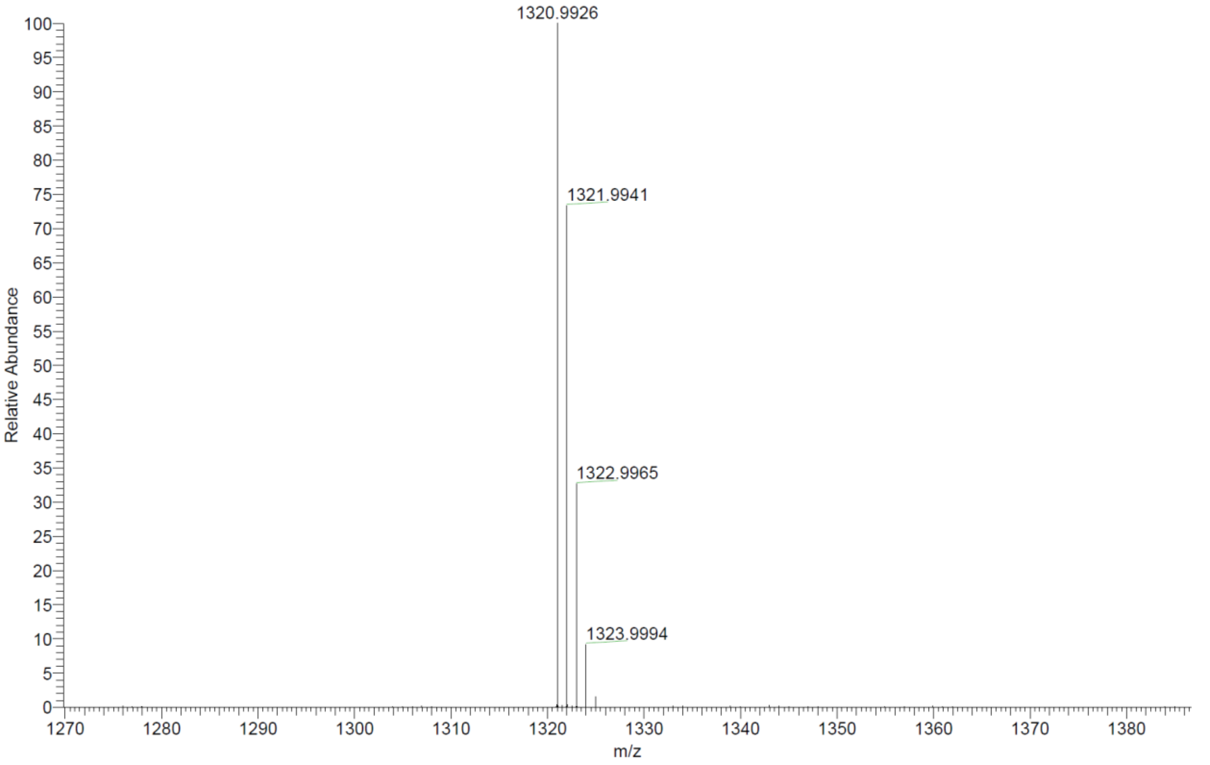

kkLLkLLKLLl (HP19) was obtained as white solid after preparative RP-HPLC (53.0 mg, 47.7%). Analytical RP-HPLC: t_R = 1.56 min (A/D 100:0 to 0:100 in 3.5 min, λ = 214 nm). MS (ESI+): $C_{66}H_{128}N_{16}O_{11}$ calc./obs. 1321.99/1322.00 Da $[M+H]^+$.

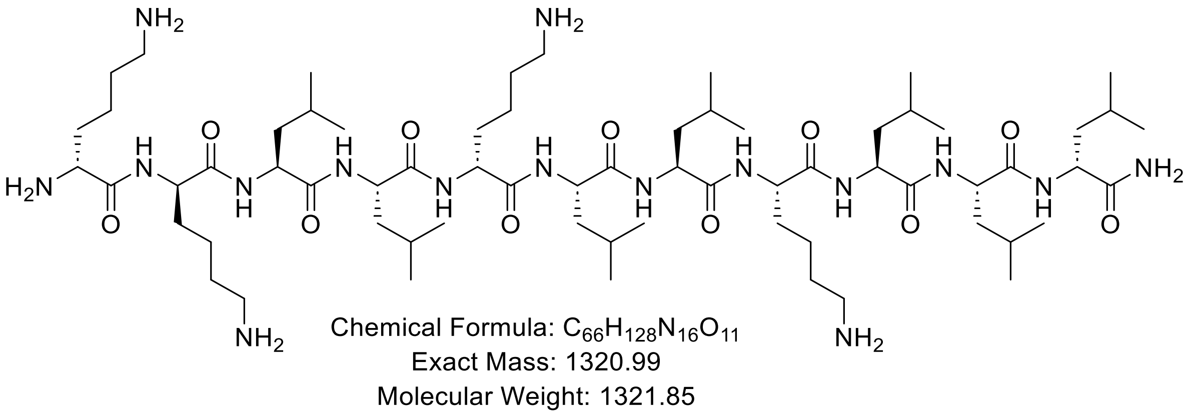

Analytical HPLC-MS data:

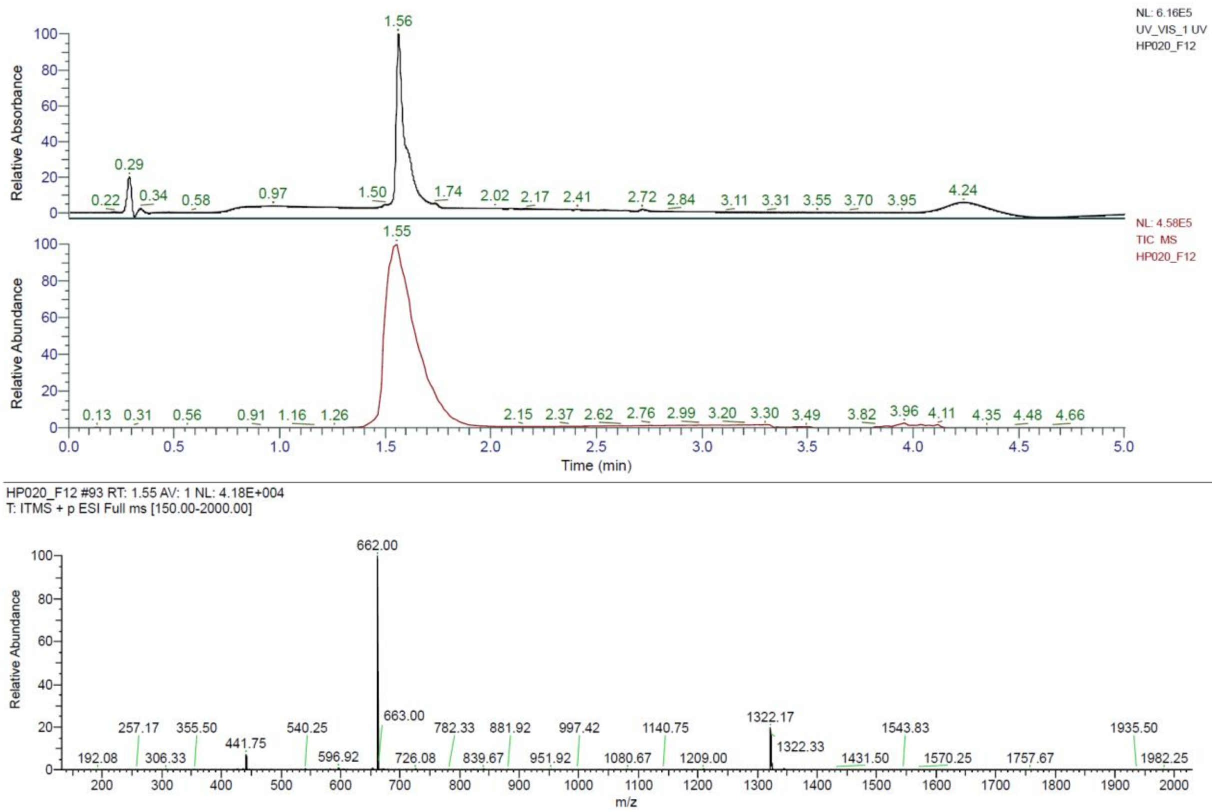

HRMS spectra:

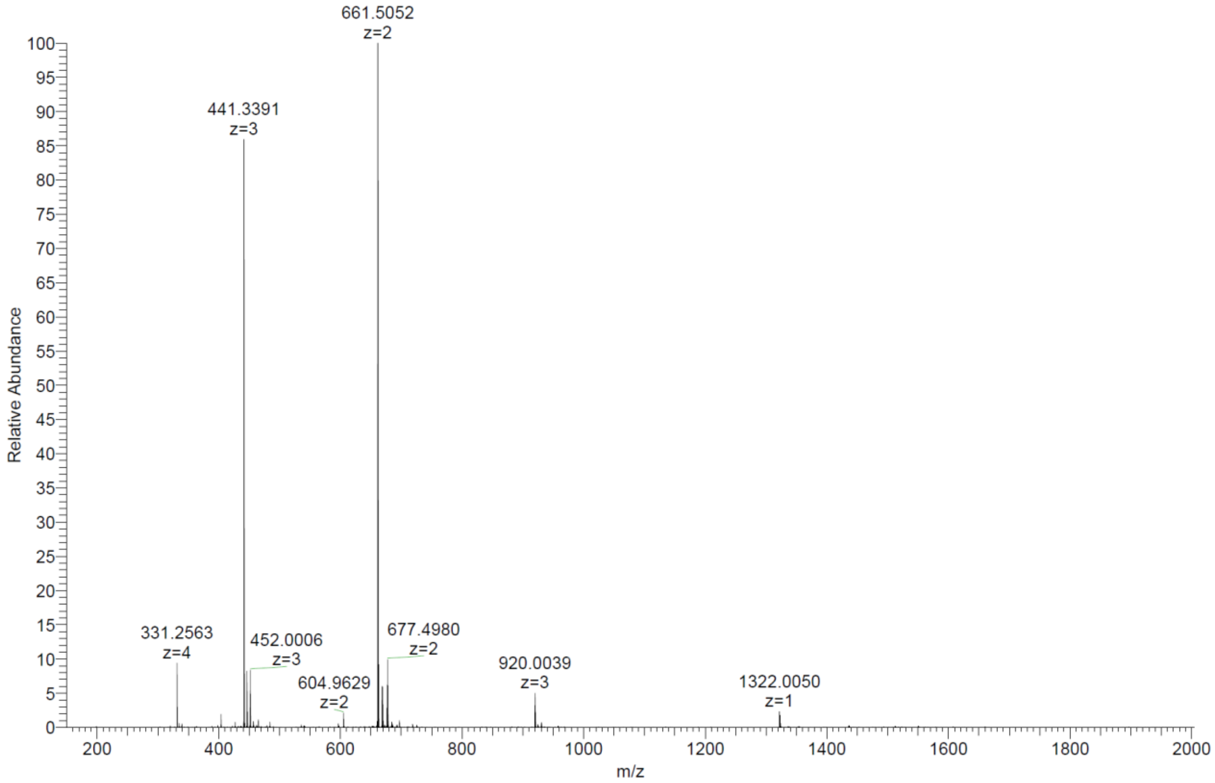

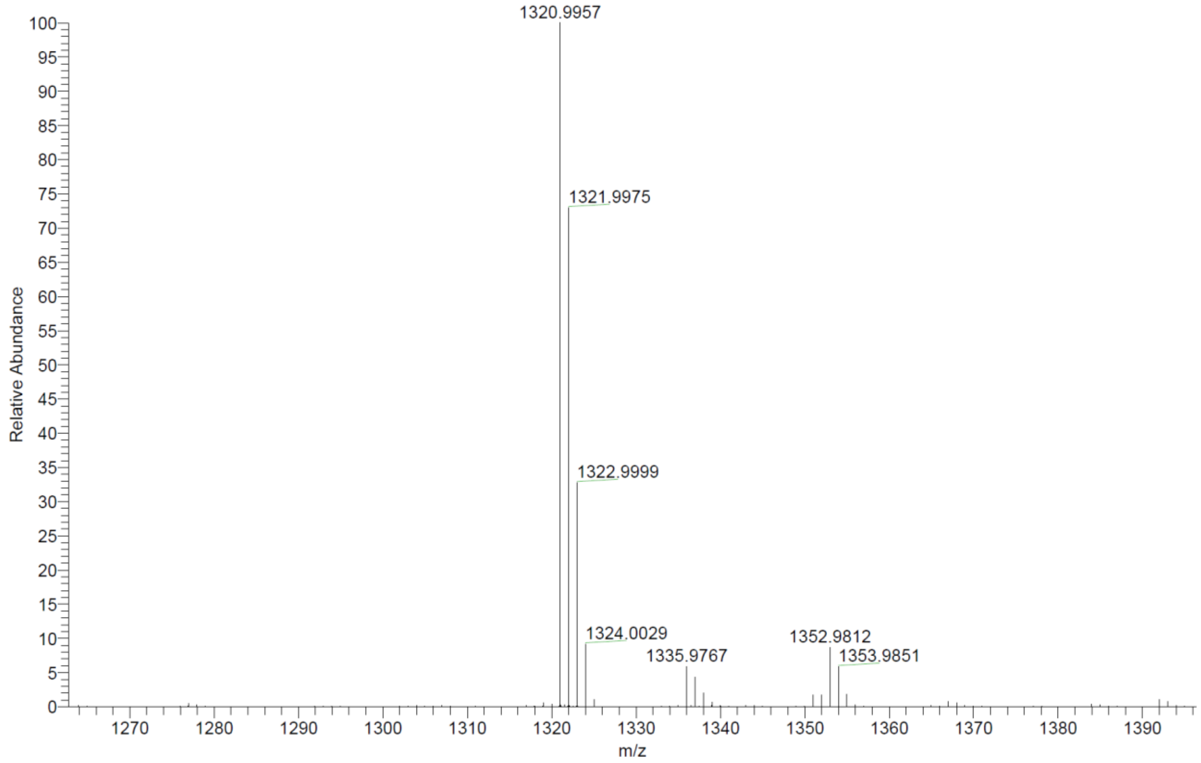

KKllKLLklLL (HP20) was obtained as white solid after preparative RP-HPLC (51.4 mg, 46.3%). Analytical RP-HPLC: t_R = 1.52 min (A/D 100:0 to 0:100 in 3.5 min, λ = 214 nm). MS (ESI+): $C_{66}H_{128}N_{16}O_{11}$ calc./obs. 1321.99/1322.00 Da $[M+H]^+$.

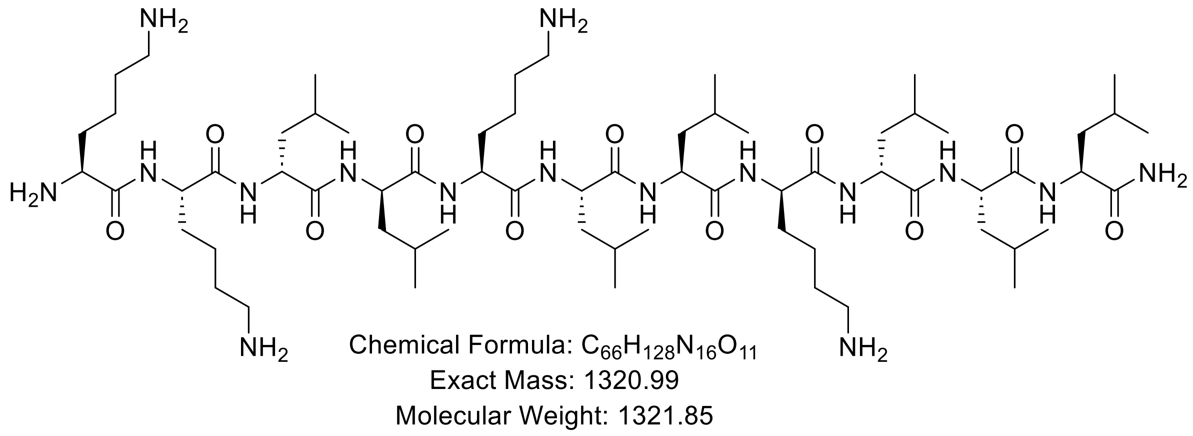

Analytical HPLC-MS data:

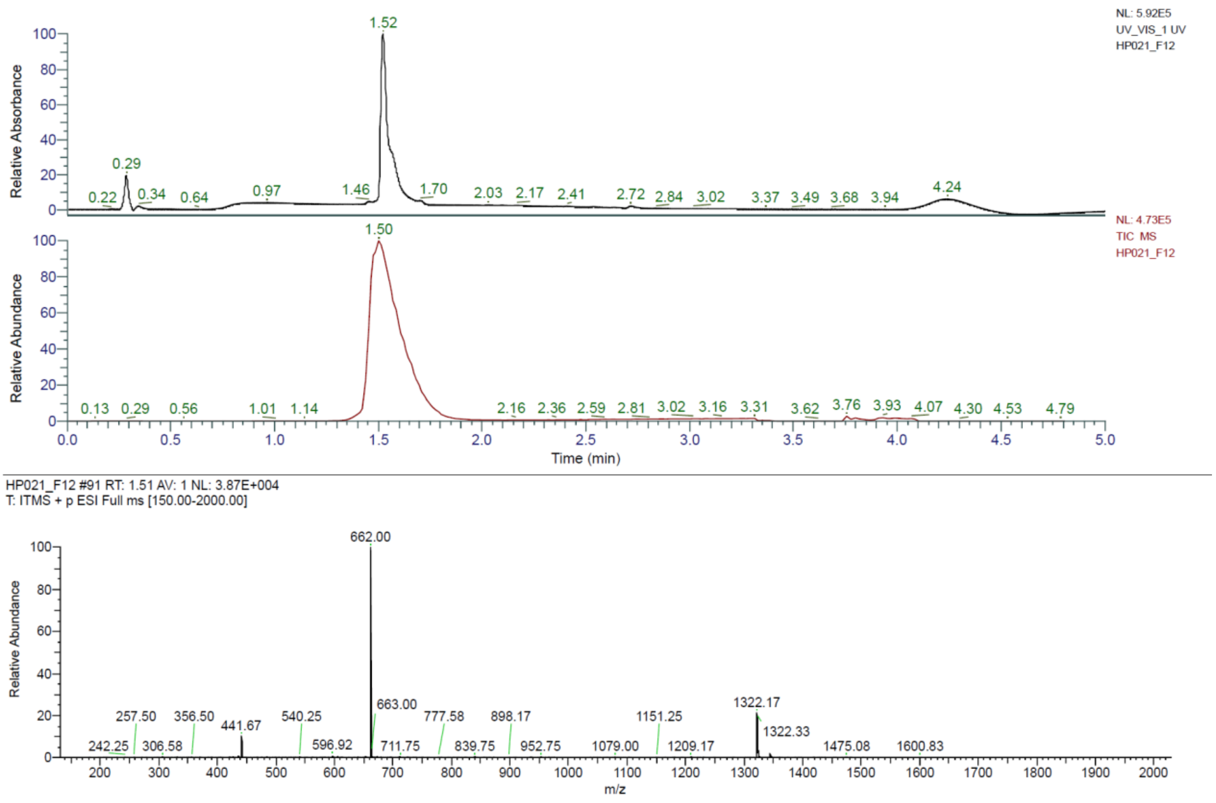

HRMS spectra:

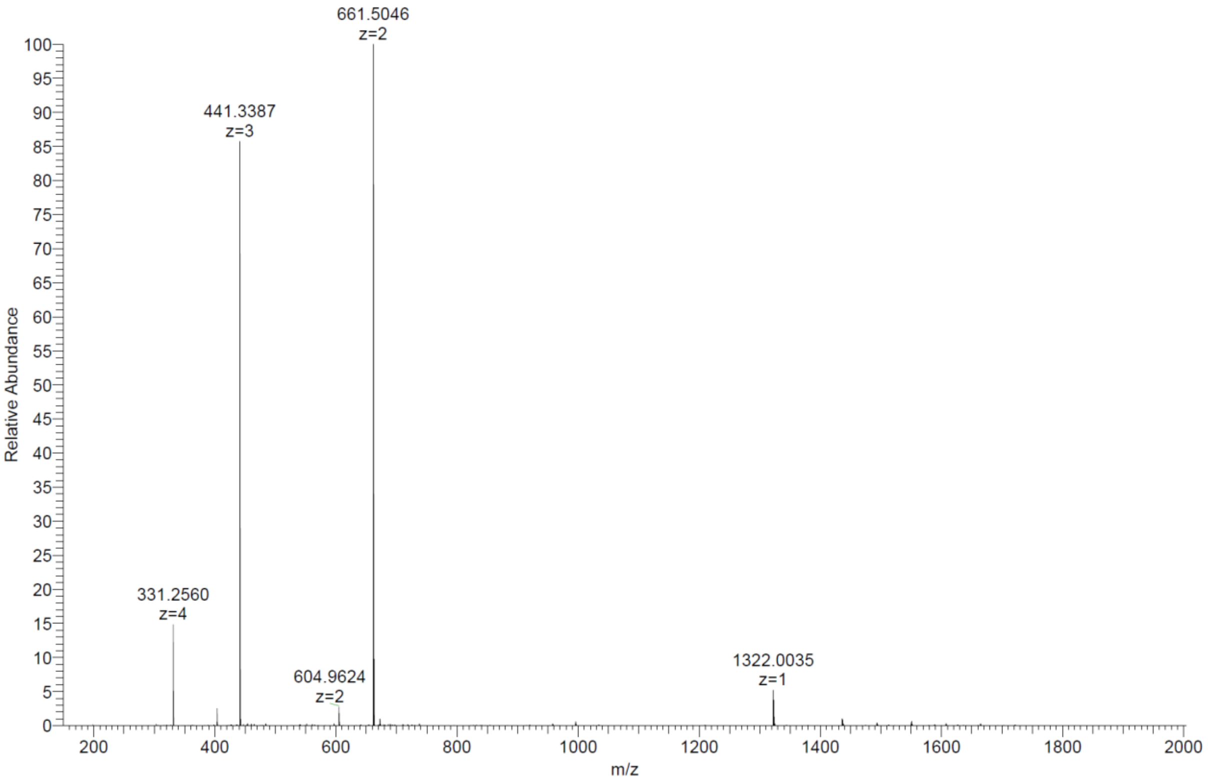

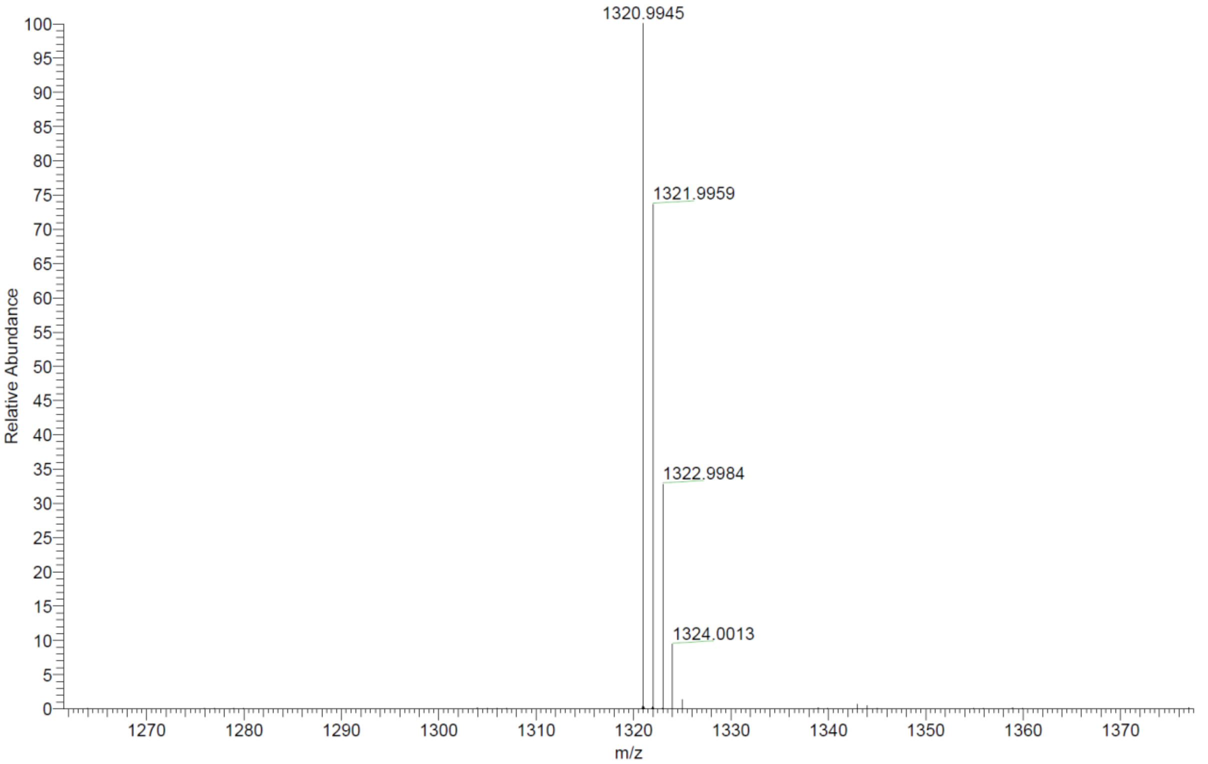

KklLKlLKlLL (HP21) was obtained as white solid after preparative RP-HPLC (64.2 mg, 65.7%). Analytical RP-HPLC: t_R = 1.60 min (A/D 100:0 to 0:100 in 3.5 min, λ = 214 nm). MS (ESI+): $C_{66}H_{128}N_{16}O_{11}$ calc./obs. 1321.99/1322.00 Da $[M+H]^+$.

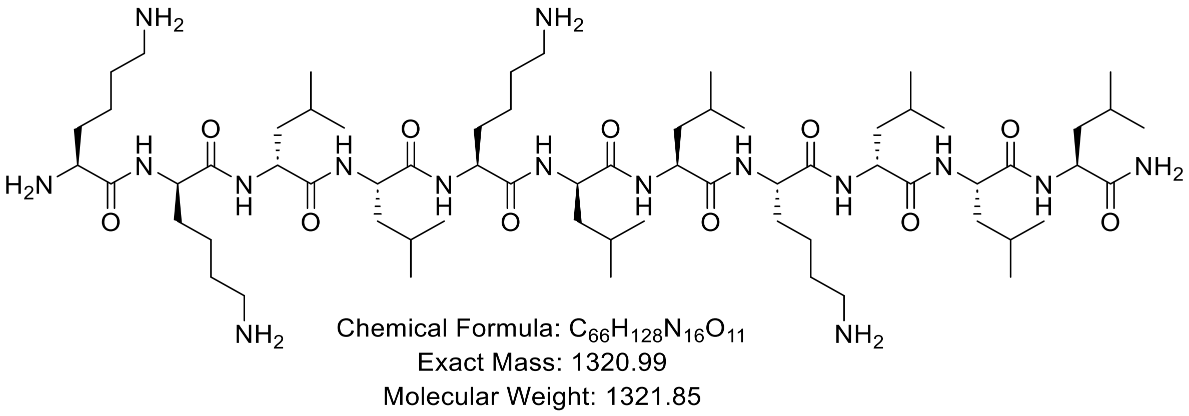

Analytical HPLC-MS data:

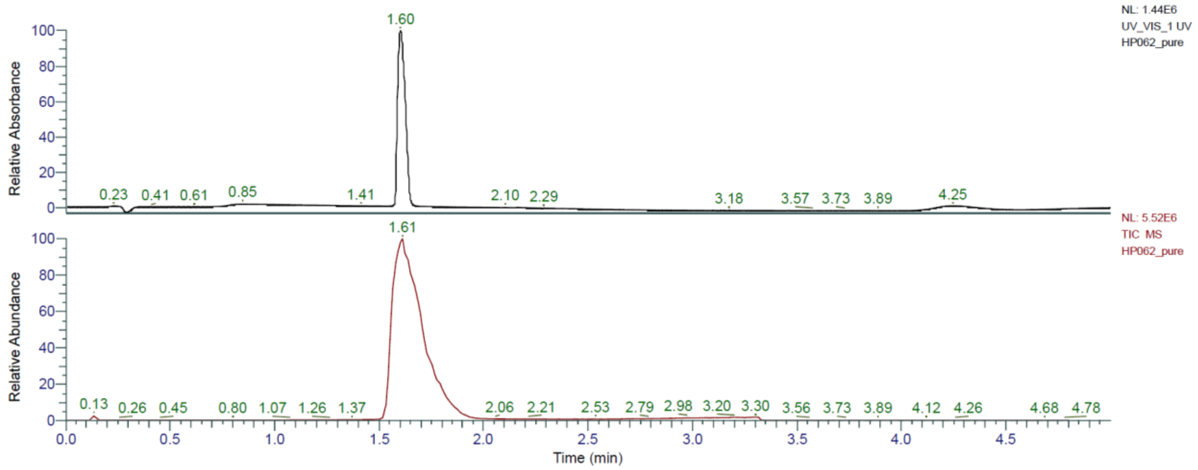

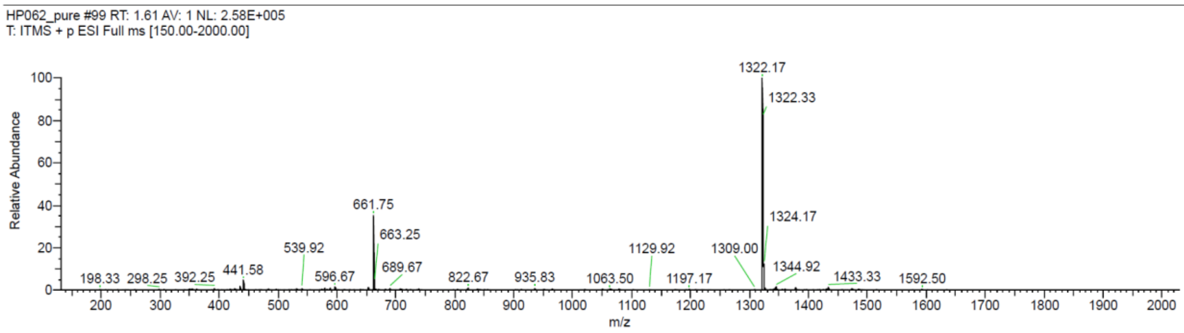

HRMS spectra:

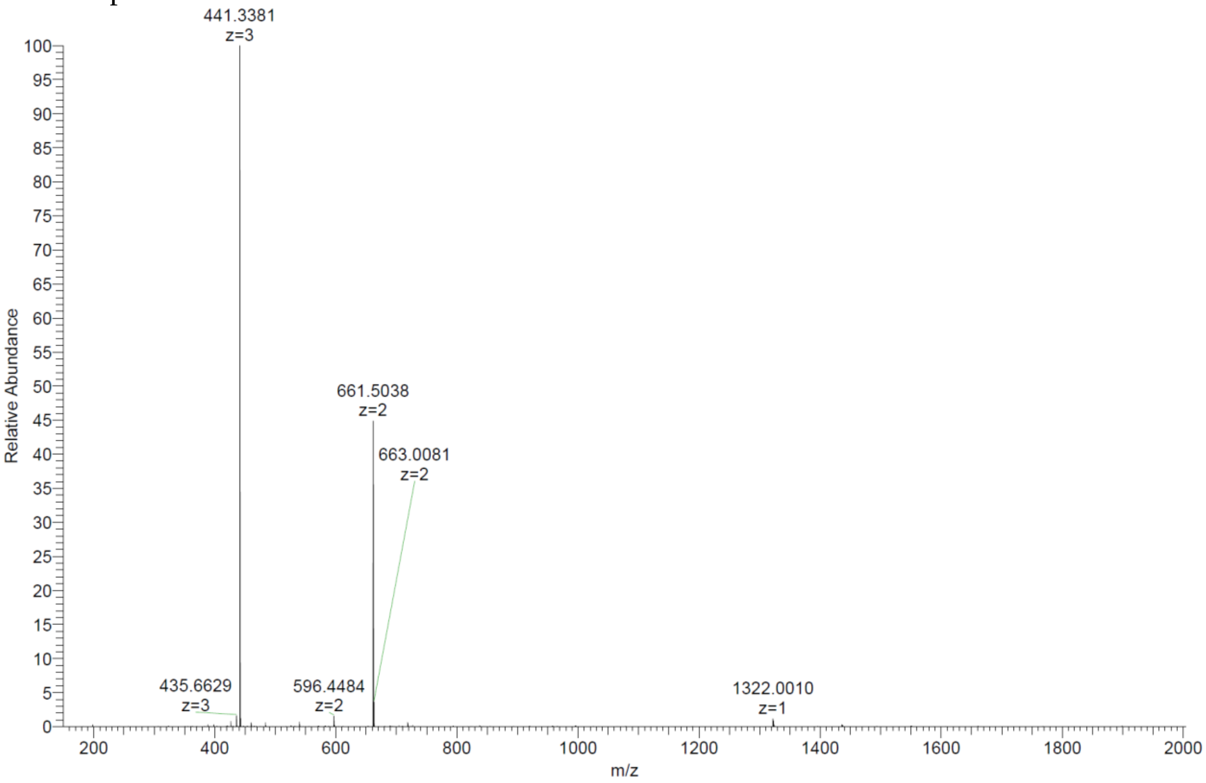

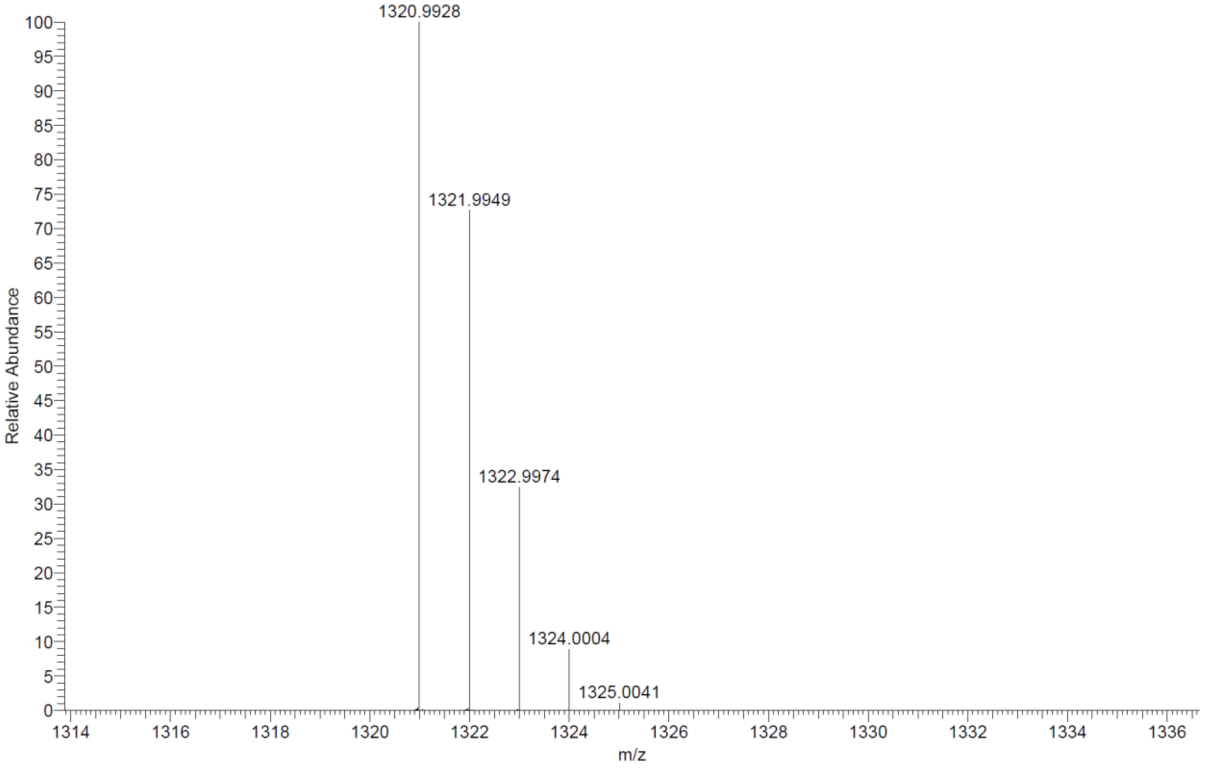

KKlIKLlKLlL (HP22) was obtained as white solid after preparative RP-HPLC (45.9 mg, 46.9%). Analytical RP-HPLC: t_R = 1.55 min (A/D 100:0 to 0:100 in 3.5 min, λ = 214 nm). MS (ESI+): $C_{66}H_{128}N_{16}O_{11}$ calc./obs. 1321.99/1322.00 Da $[M+H]^+$.

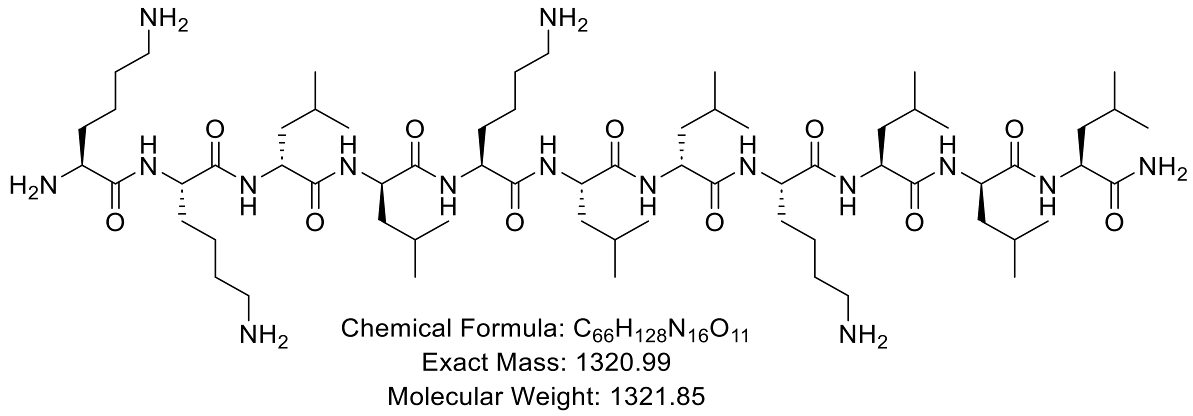

Analytical HPLC-MS data:

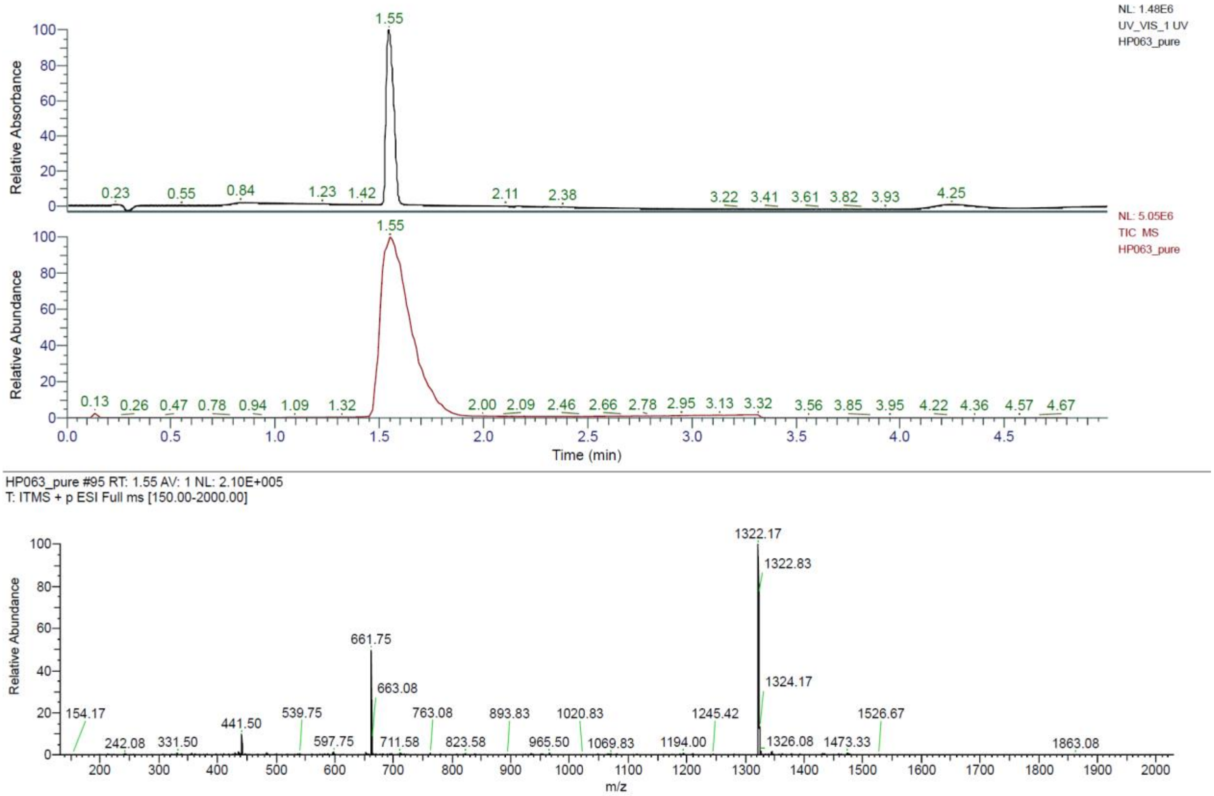

HRMS spectra:

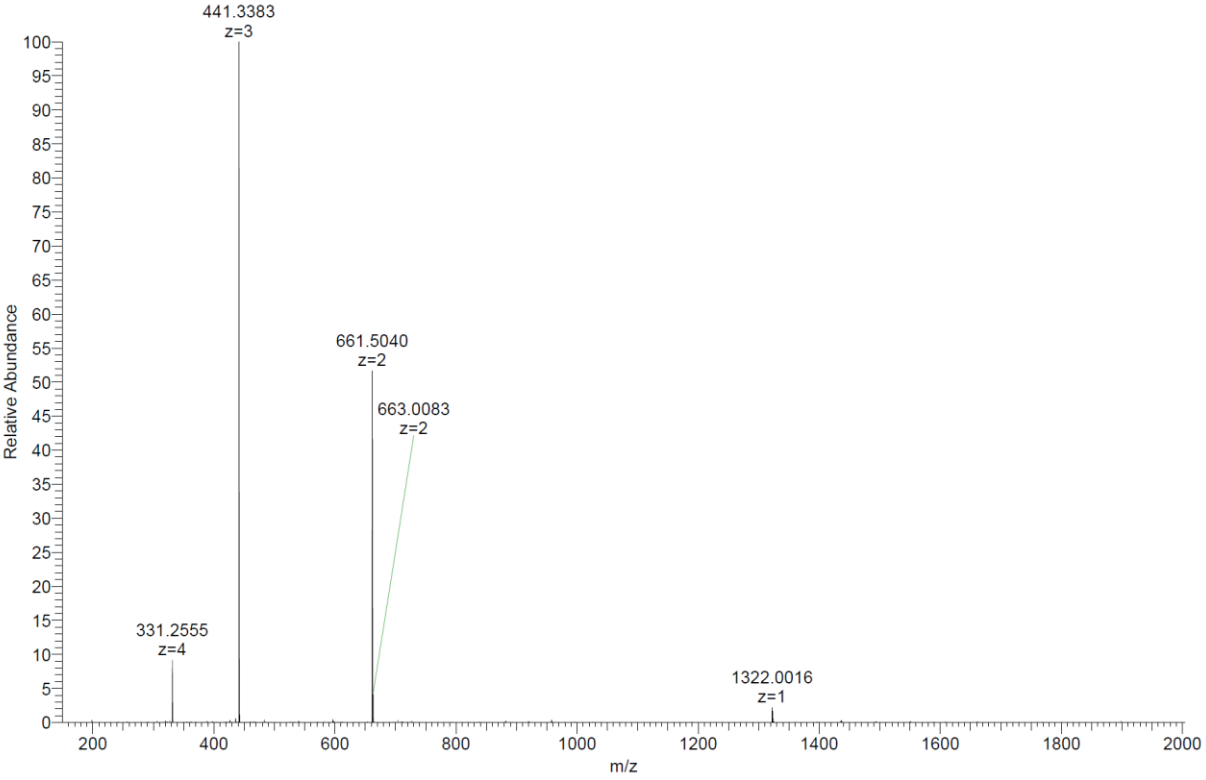

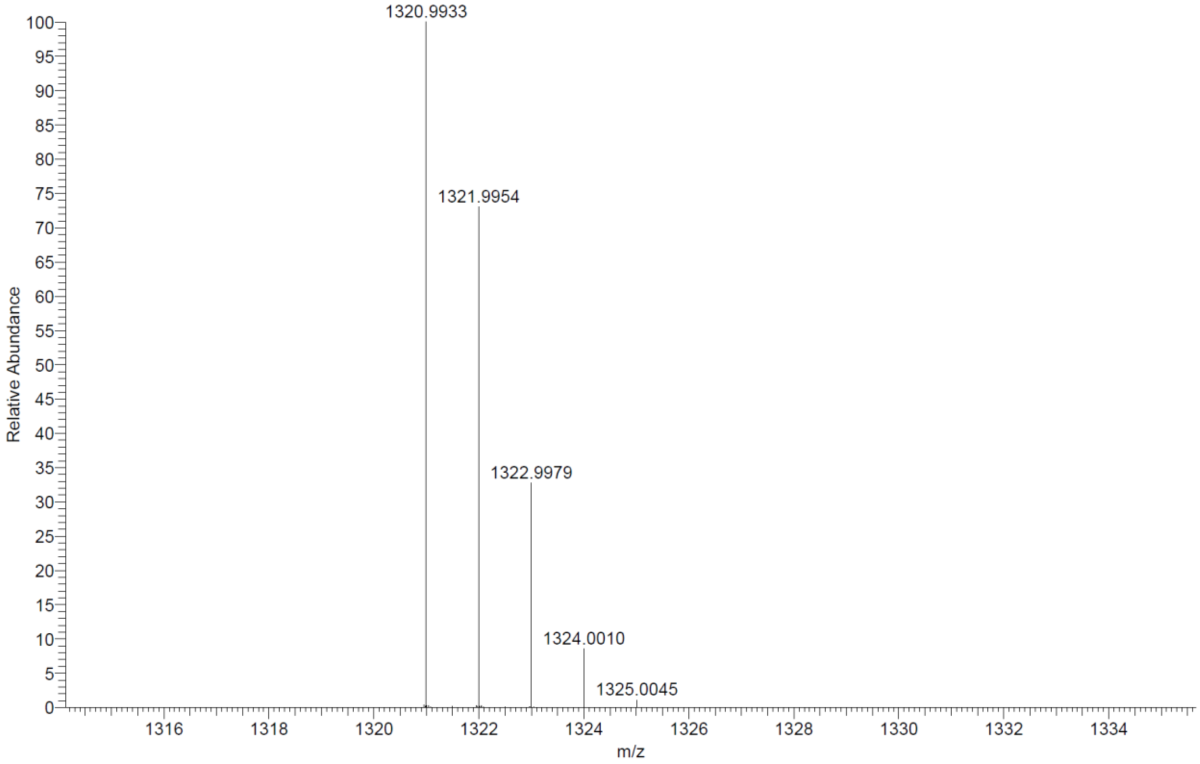

KKLlkLLkLLl (HP23) was obtained as white solid after preparative RP-HPLC (64.7 mg, 66.2%). Analytical RP-HPLC: t_R = 1.56 min (A/D 100:0 to 0:100 in 3.5 min, λ = 214 nm). MS (ESI+): $C_{66}H_{128}N_{16}O_{11}$ calc./obs. 1321.99/1322.00 Da $[M+H]^+$.

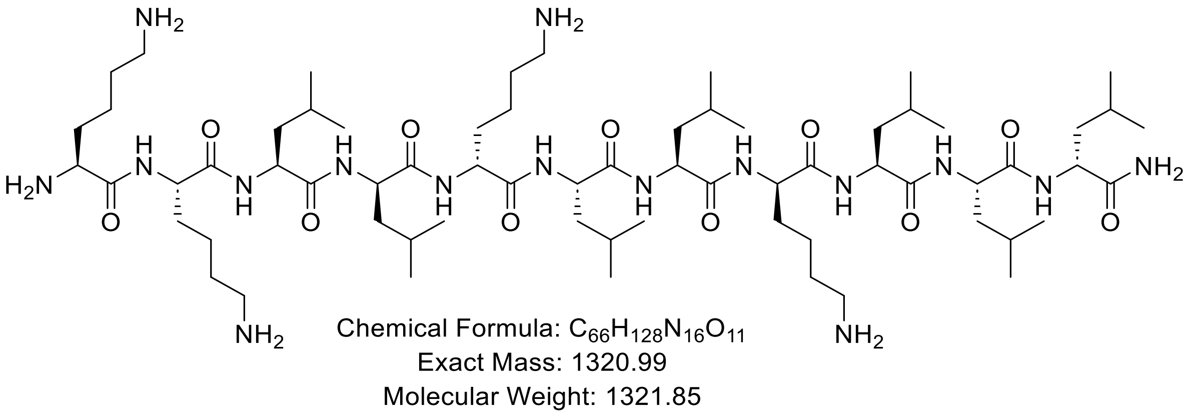

Analytical HPLC-MS data:

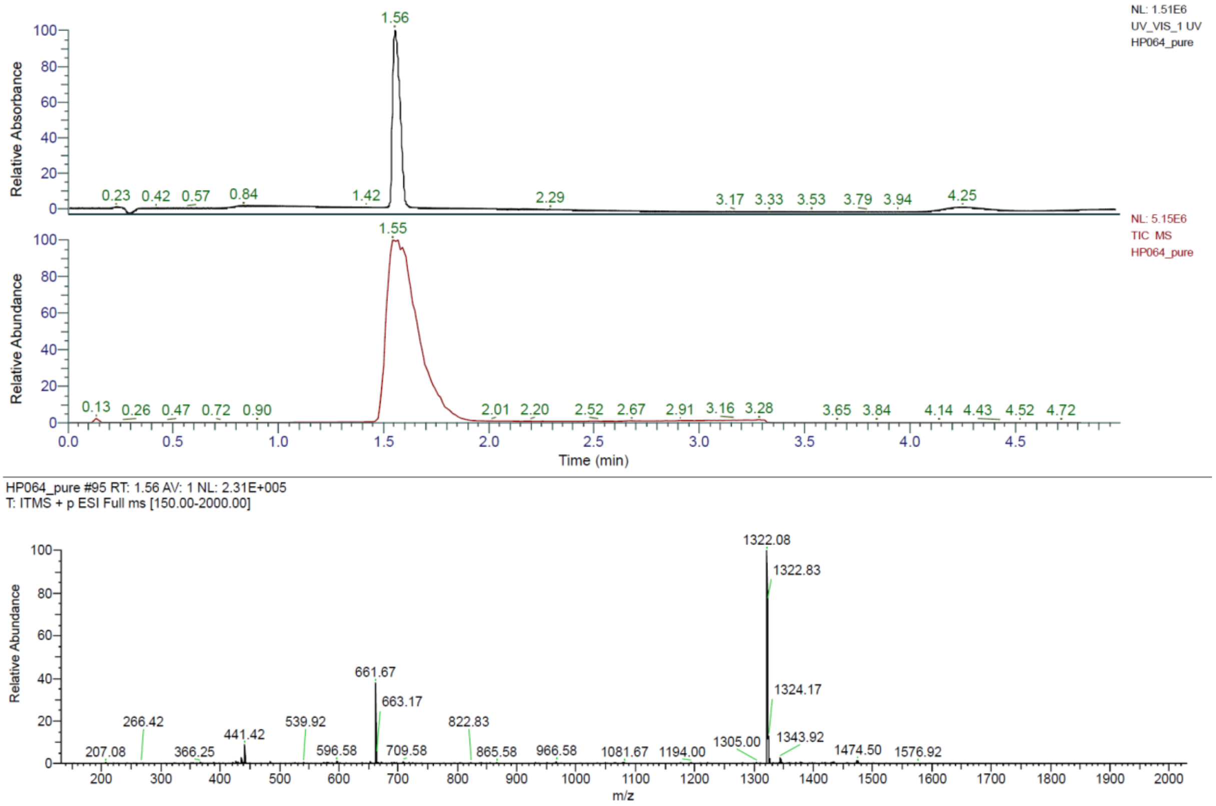

HRMS spectra:

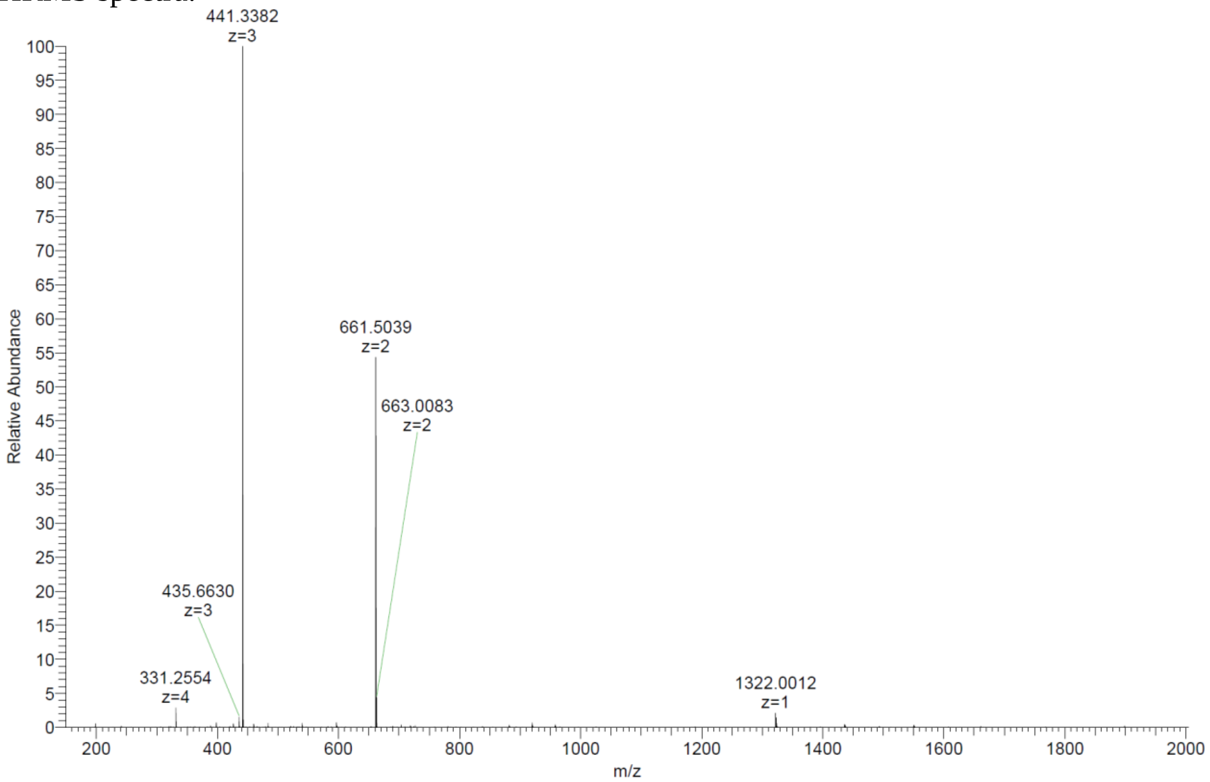

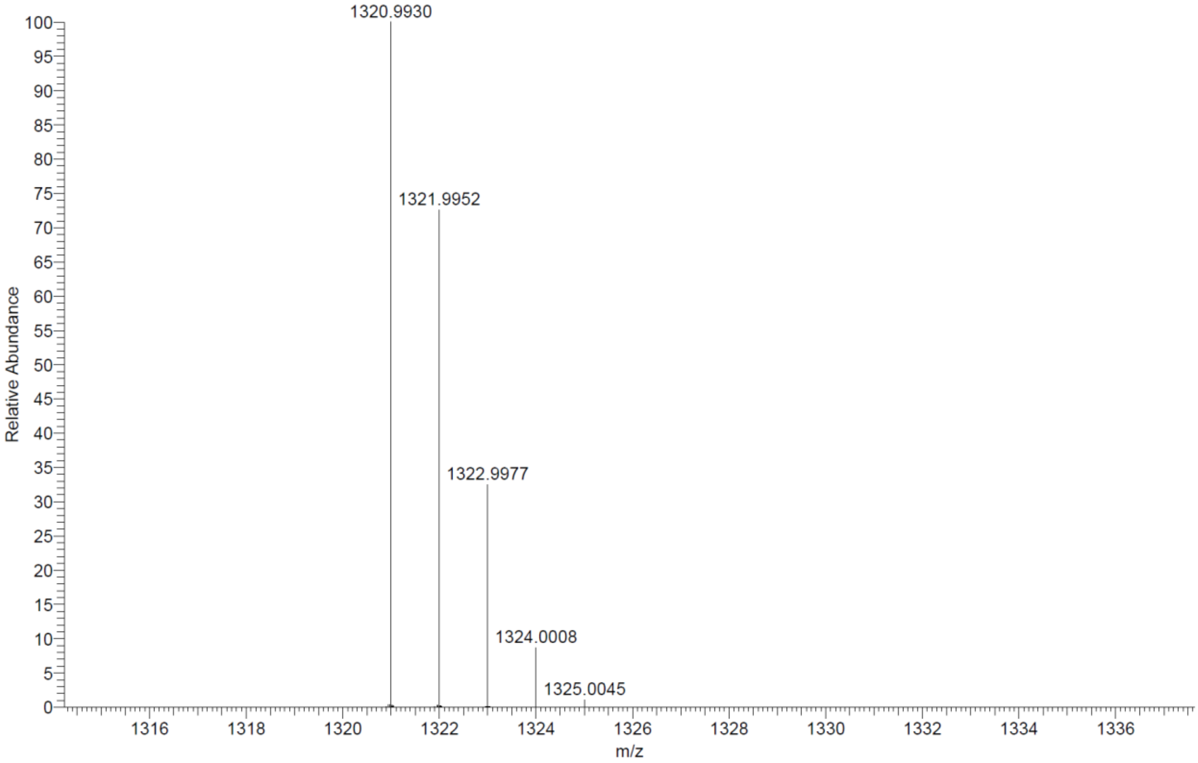

KkLlKllKLLL (HP24) was obtained as white solid after preparative RP-HPLC (54.7 mg, 49.2%). Analytical RP-HPLC: t_R = 1.56 min (A/D 100:0 to 0:100 in 3.5 min, λ = 214 nm). MS (ESI+): $C_{66}H_{128}N_{16}O_{11}$ calc./obs. 1321.99/1322.00 Da $[M+H]^+$.

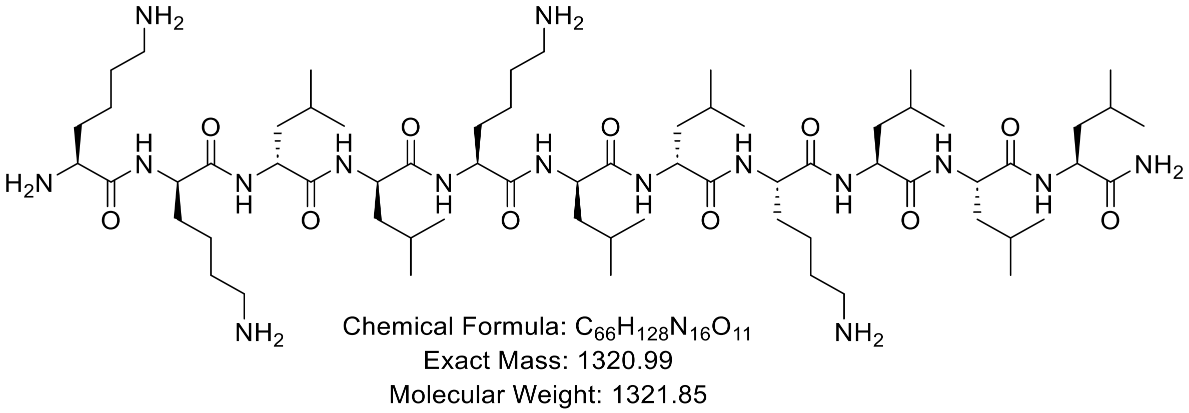

Analytical HPLC-MS data:

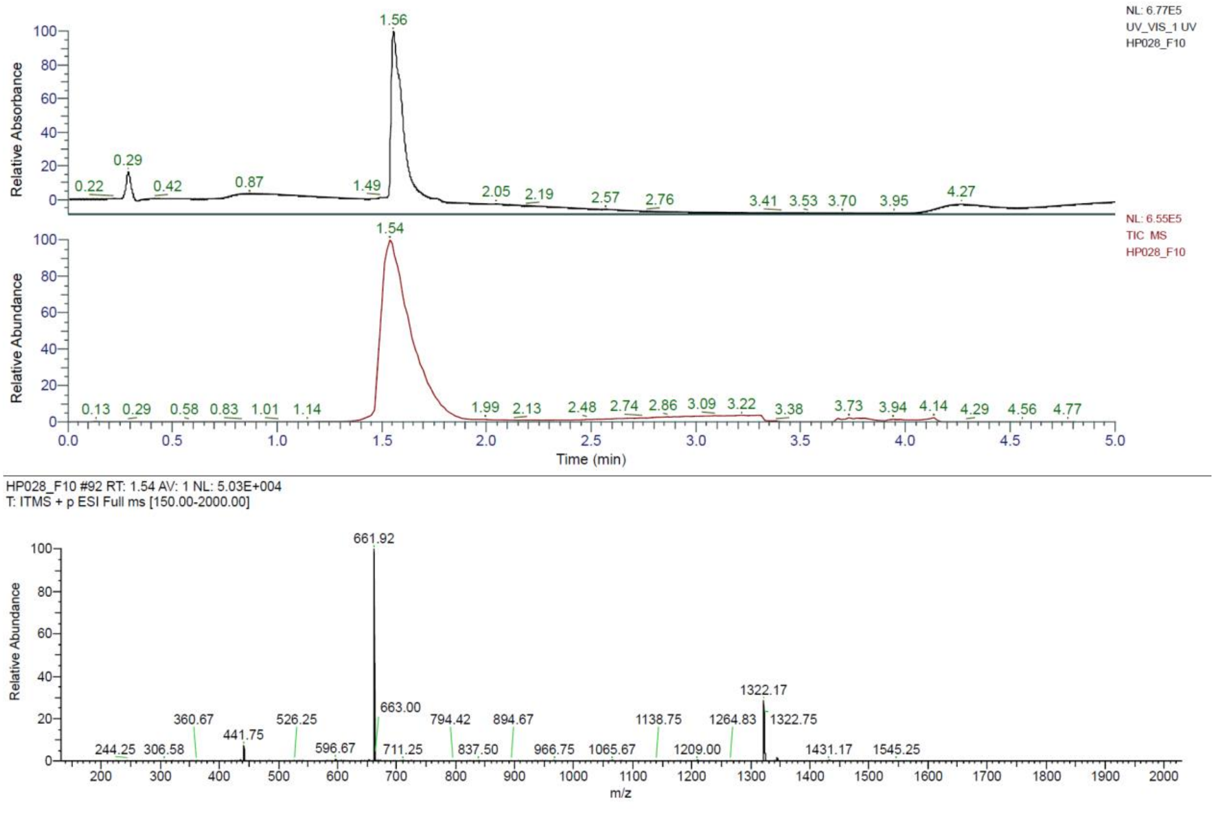

HRMS spectra:

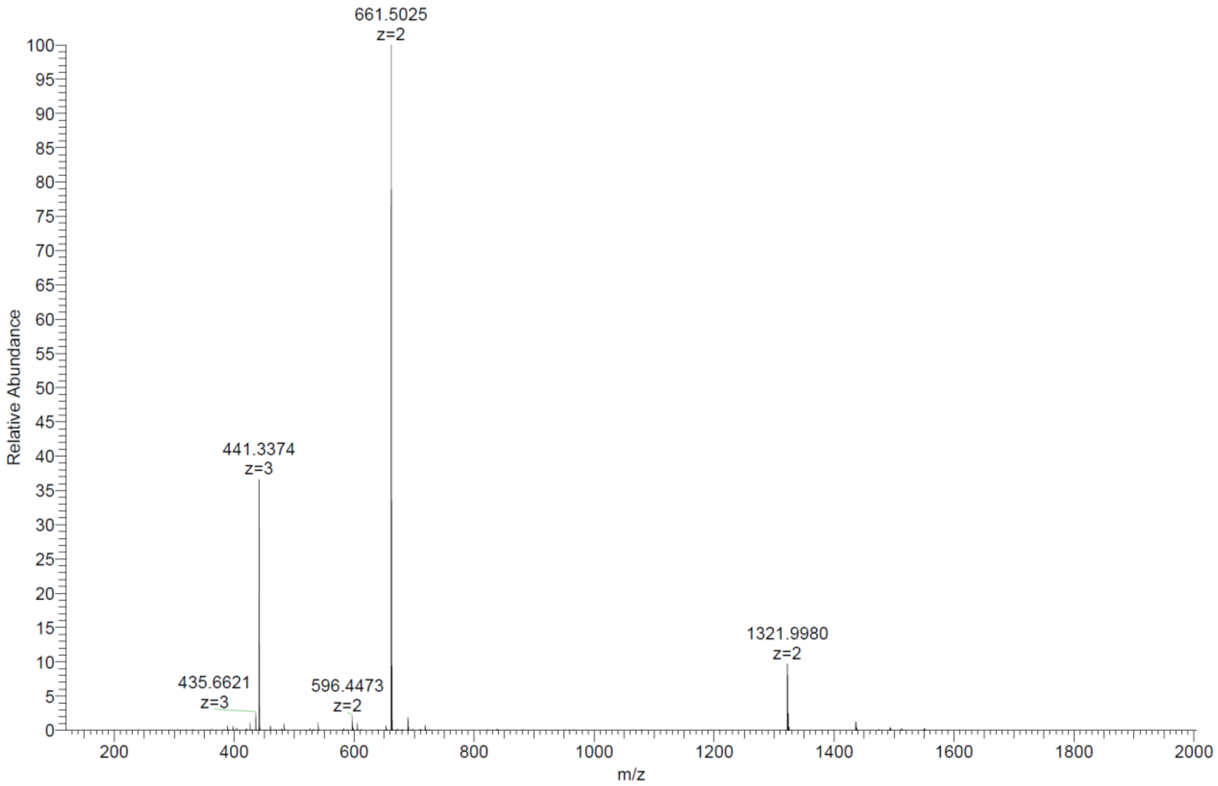

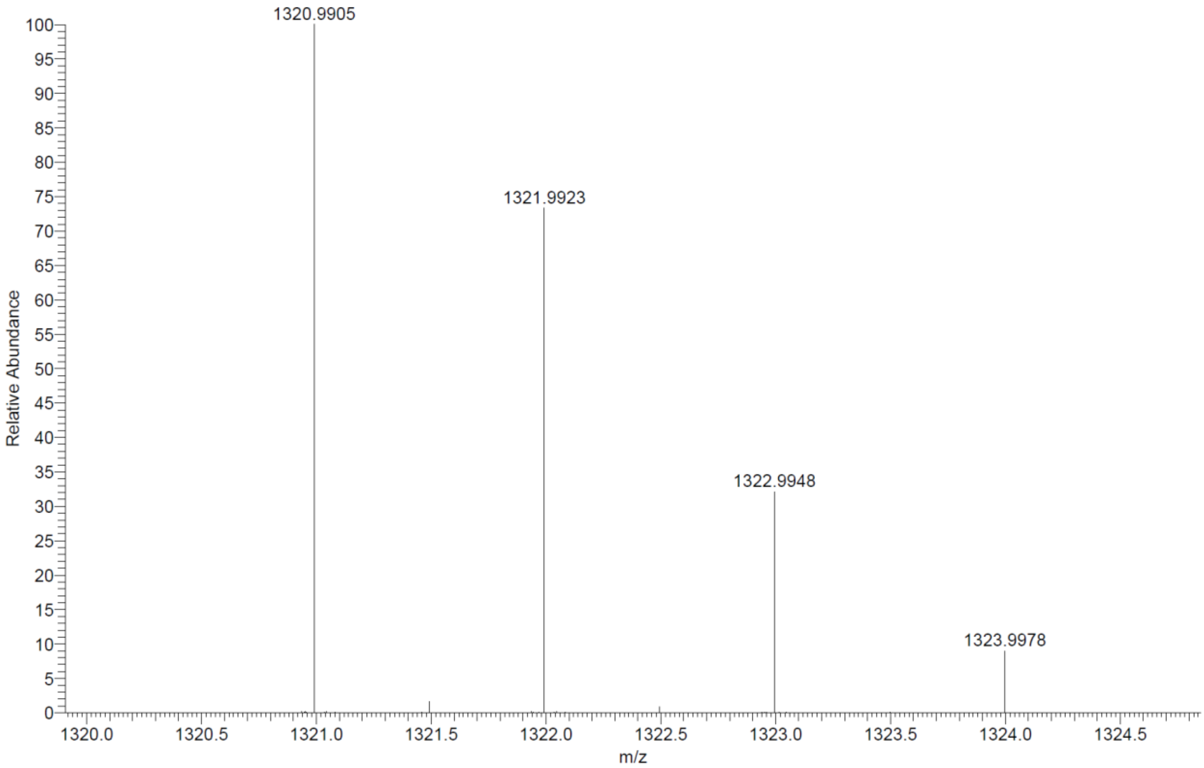

KKllKllKlLL (HP25) was obtained as white solid after preparative RP-HPLC (38.8 mg, 34.9%). Analytical RP-HPLC: t_R = 1.55 min (A/D 100:0 to 0:100 in 3.5 min, λ = 214 nm). MS (ESI+): $C_{66}H_{128}N_{16}O_{11}$ calc./obs. 1321.99/1322.00 Da $[M+H]^+$.

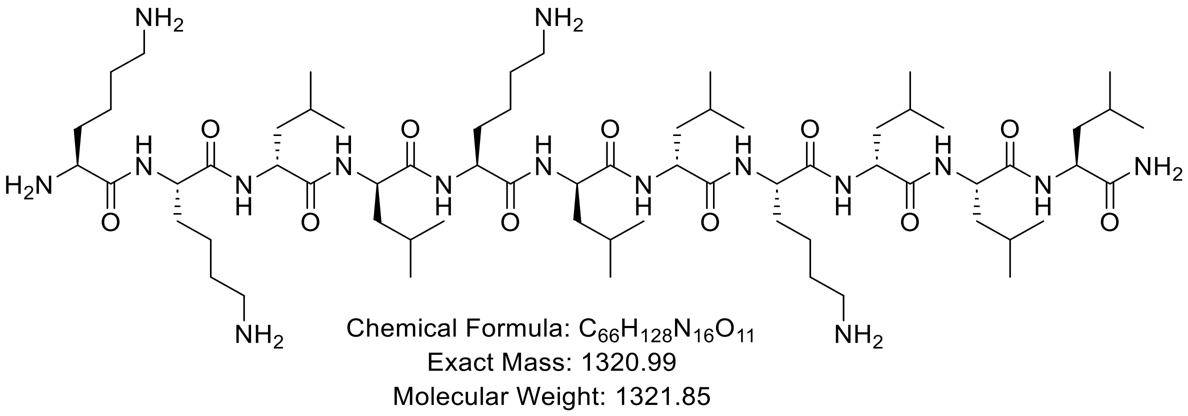

Analytical HPLC-MS data:

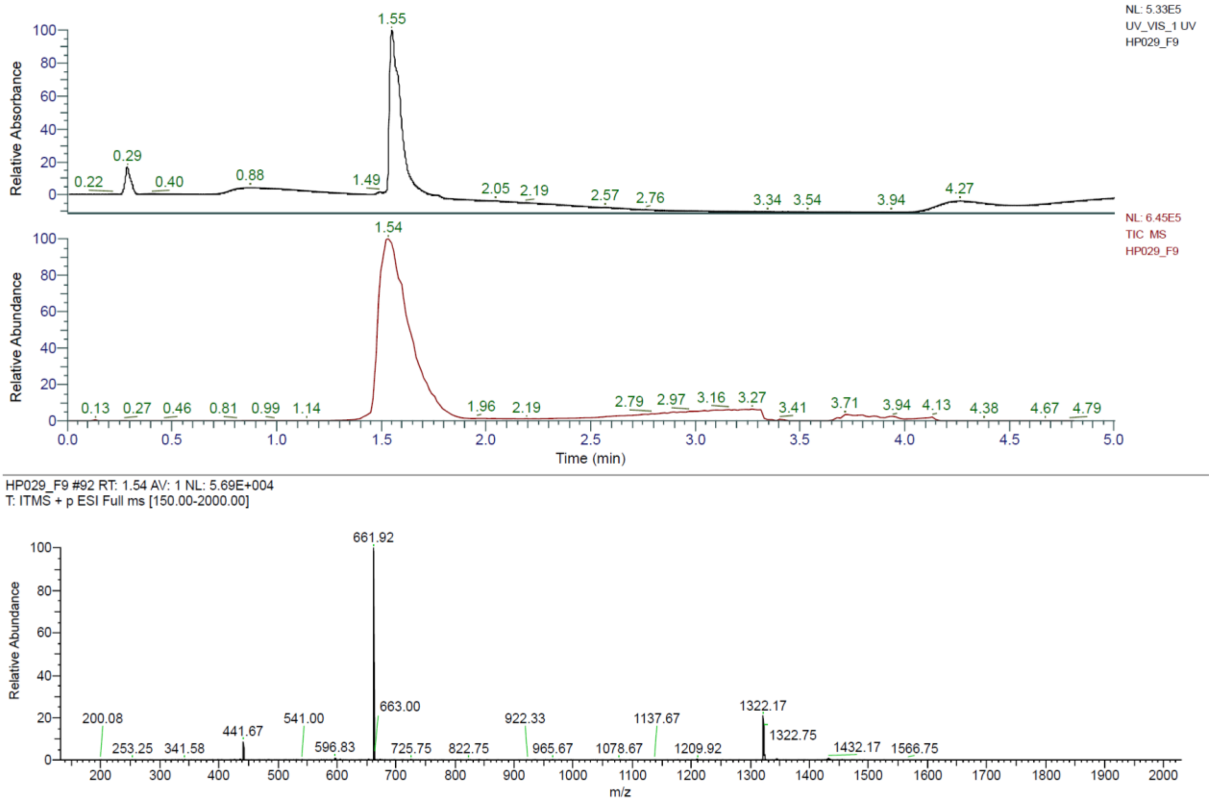

HRMS spectra:

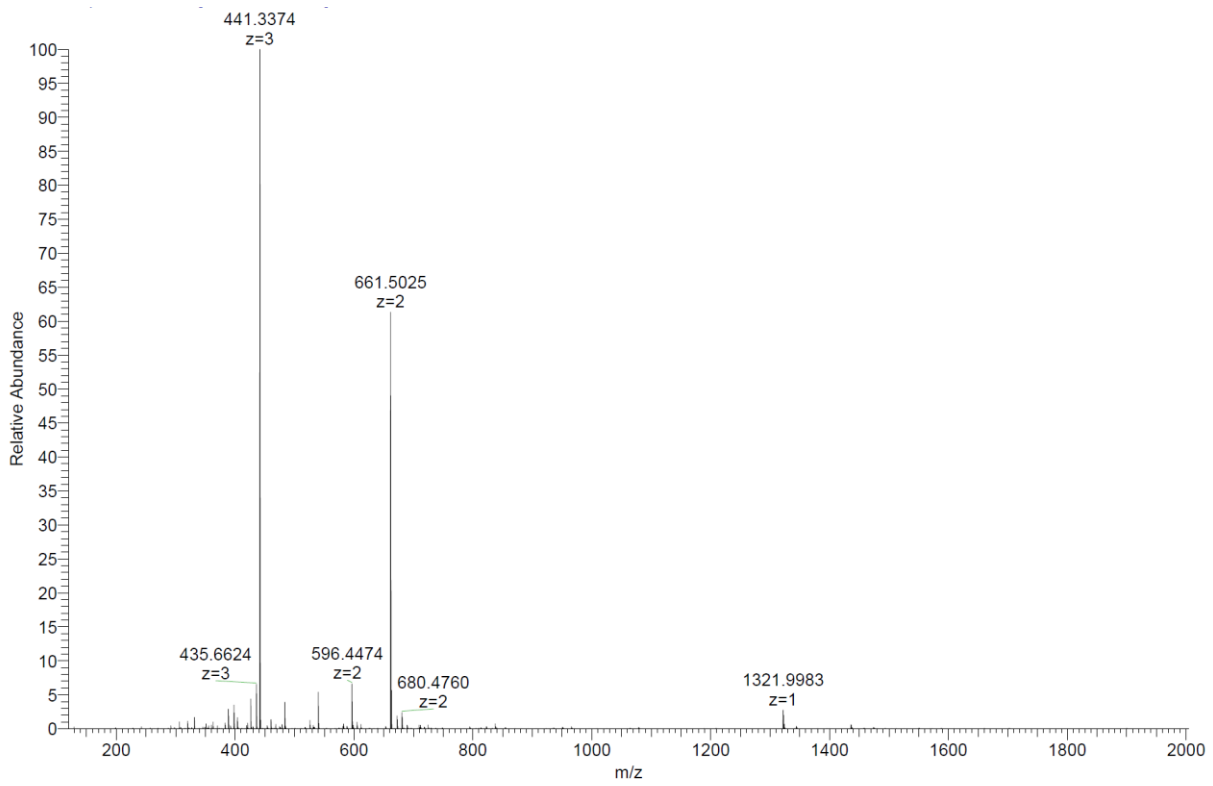

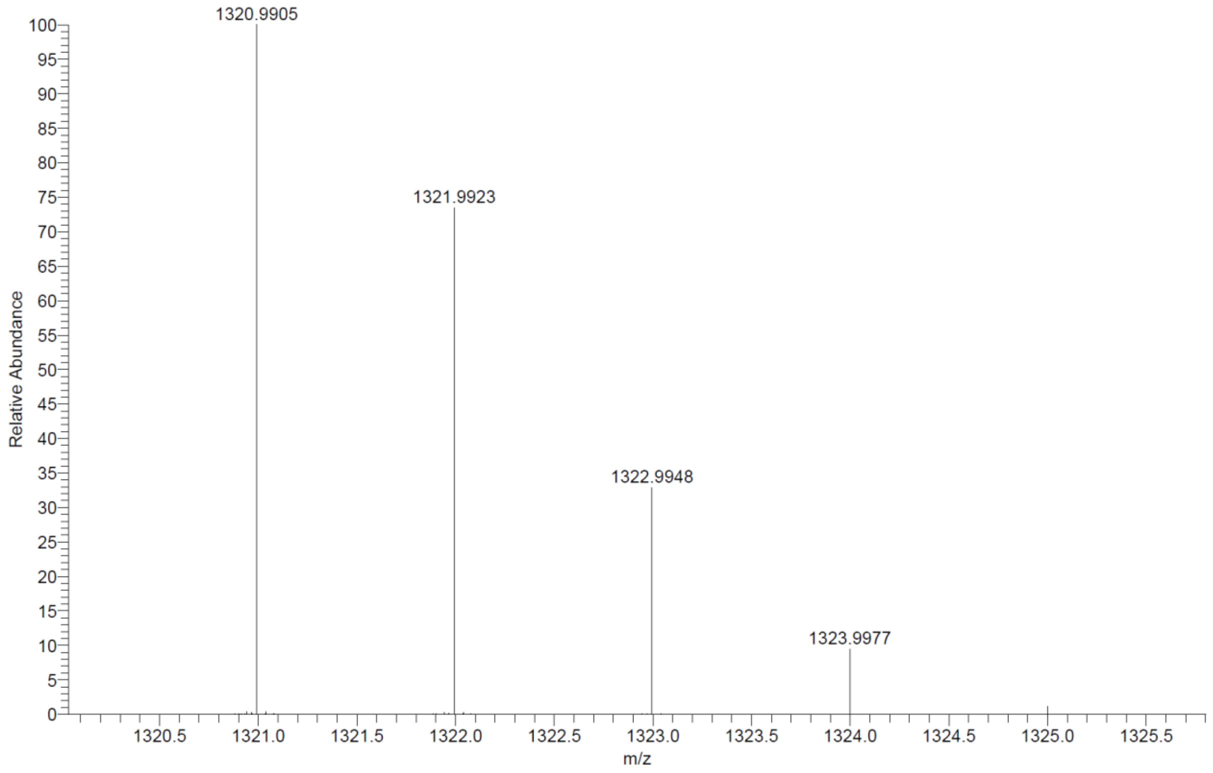

kkLLkLLKLll (HP26) was obtained as white solid after preparative RP-HPLC (52.6 mg, 47.3%). Analytical RP-HPLC: t_R = 1.57 min (A/D 100:0 to 0:100 in 3.5 min, λ = 214 nm). MS (ESI+): $C_{66}H_{128}N_{16}O_{11}$ calc./obs. 1321.99/1322.00 Da $[M+H]^+$.

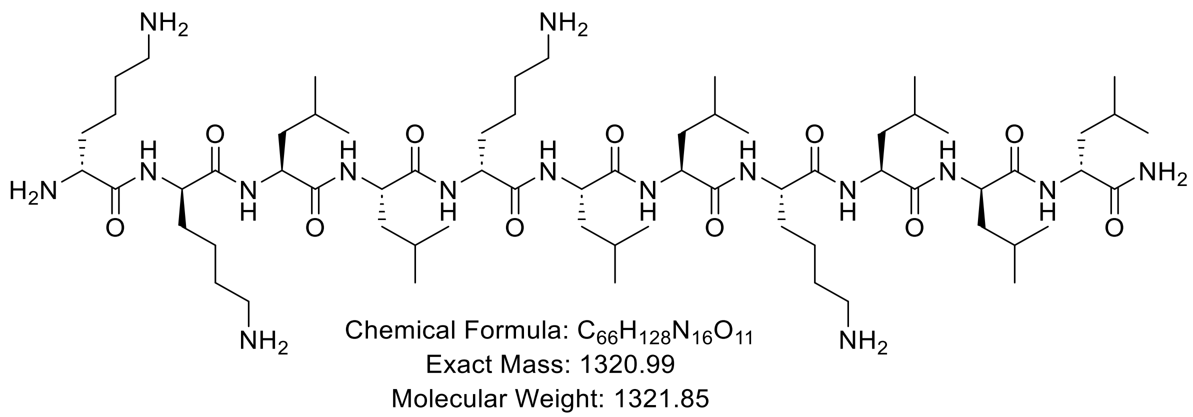

Analytical HPLC-MS data:

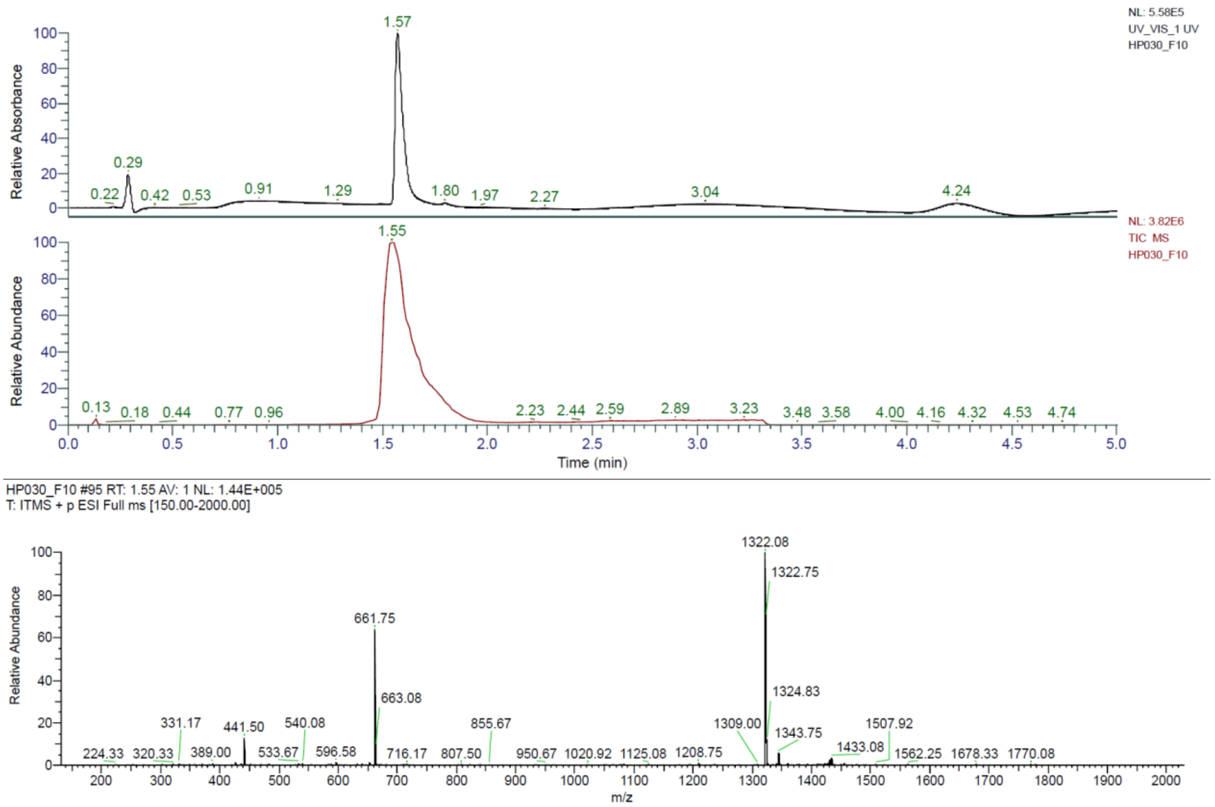

HRMS spectra:

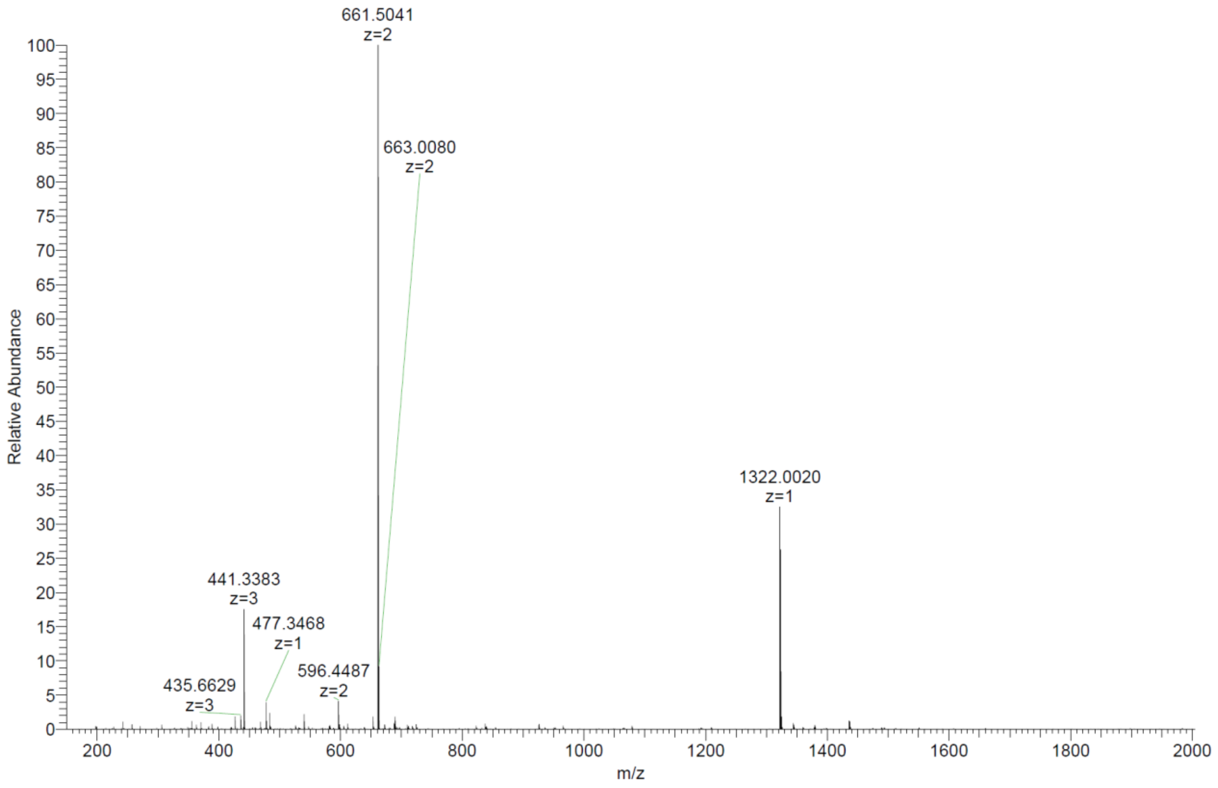

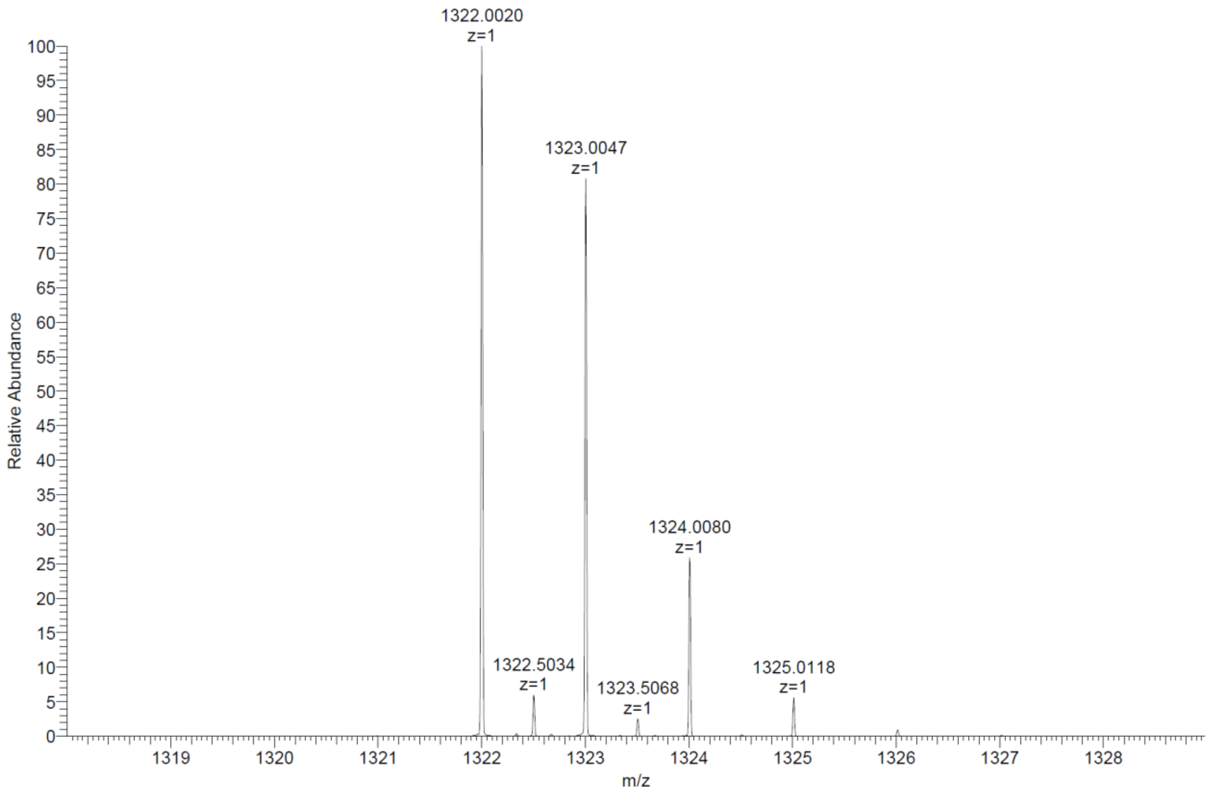

kkLLkLLkLLl (HP27) was obtained as white solid after preparative RP-HPLC (53.7 mg, 48.3%). Analytical RP-HPLC: t_R = 1.54 min (A/D 100:0 to 0:100 in 3.5 min, λ = 214 nm). MS (ESI+): $C_{66}H_{128}N_{16}O_{11}$ calc./obs. 1321.99/1322.00 Da $[M+H]^+$.

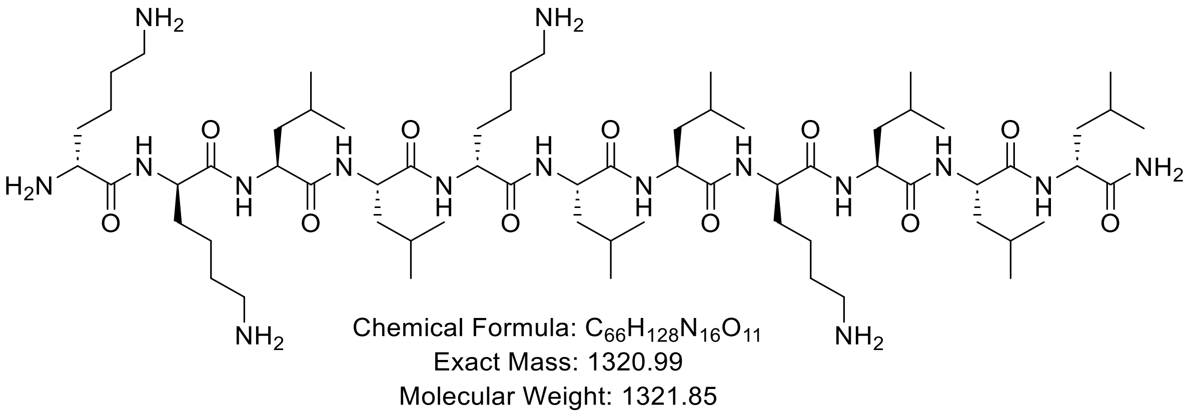

Analytical HPLC-MS data:

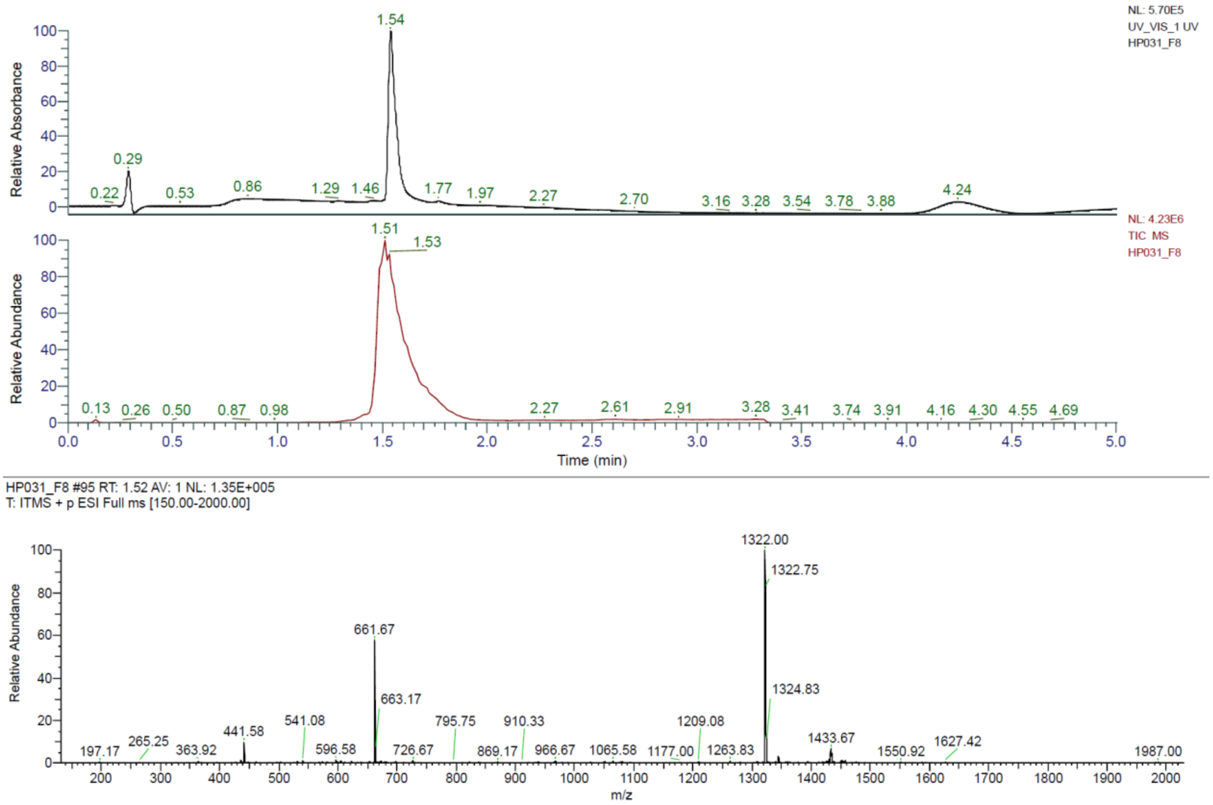

HRMS spectra:

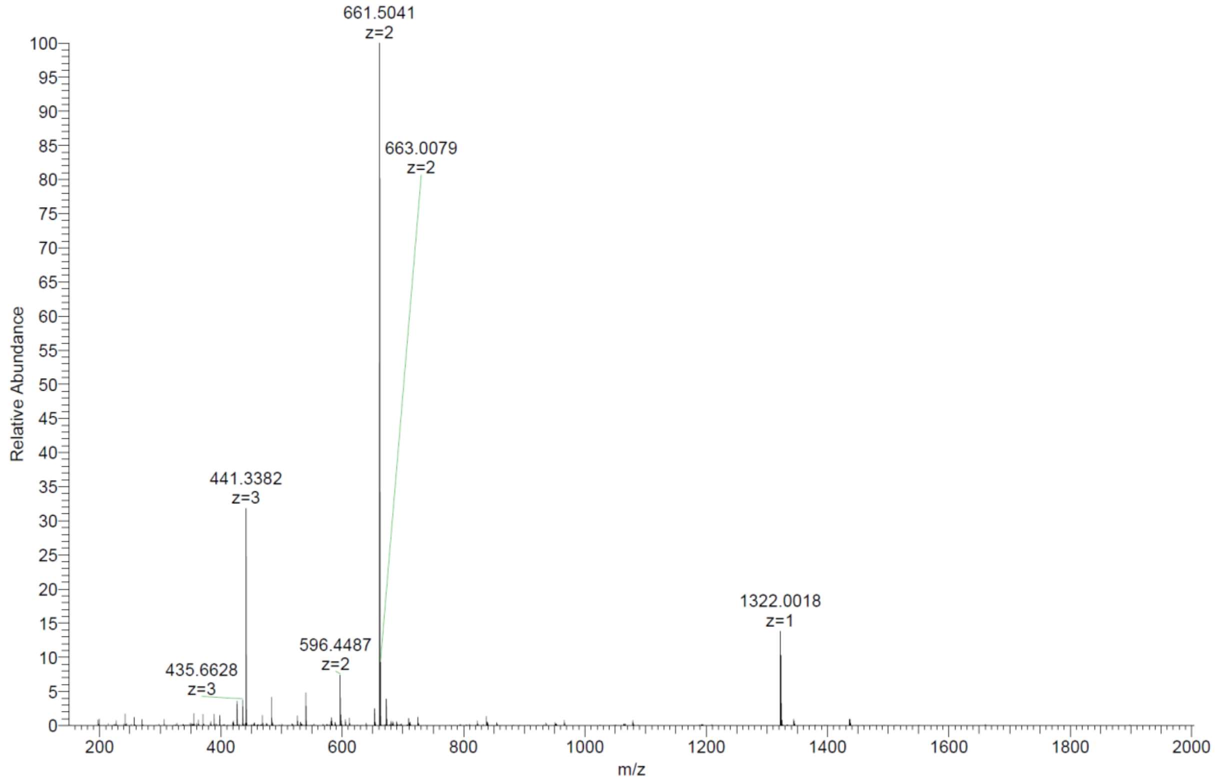

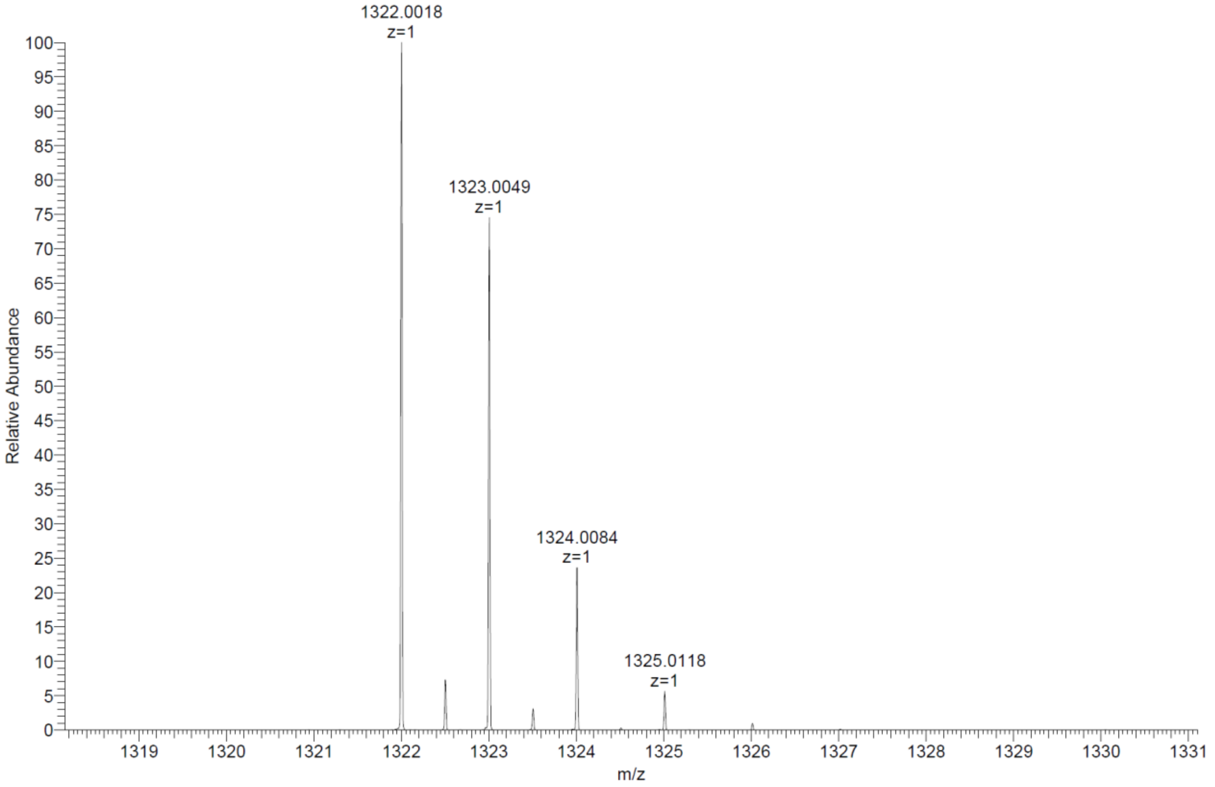

kKLLkllKLLl (HP28) was obtained as white solid after preparative RP-HPLC (45.6 mg, 41.0%). Analytical RP-HPLC: t_R = 1.55 min (A/D 100:0 to 0:100 in 3.5 min, λ = 214 nm). MS (ESI+): $C_{66}H_{128}N_{16}O_{11}$ calc./obs. 1321.99/1322.00 Da $[M+H]^+$.

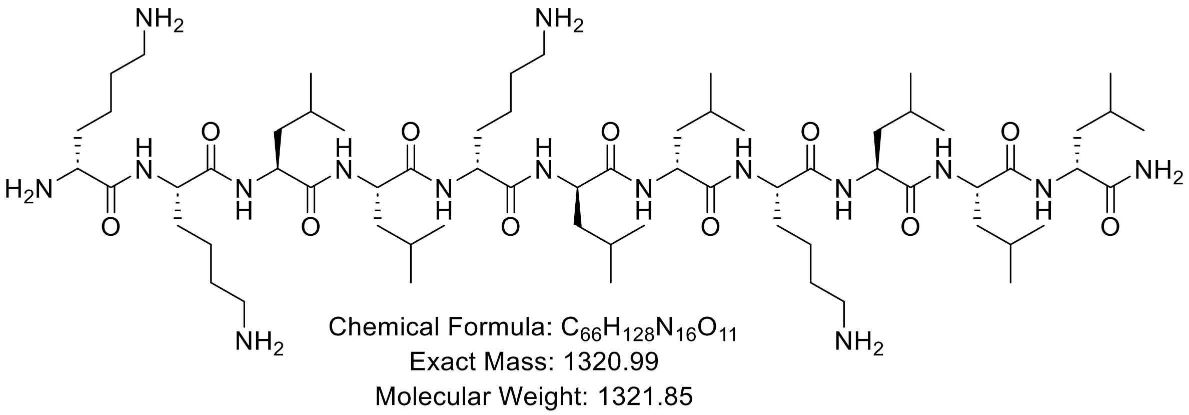

Analytical HPLC-MS data:

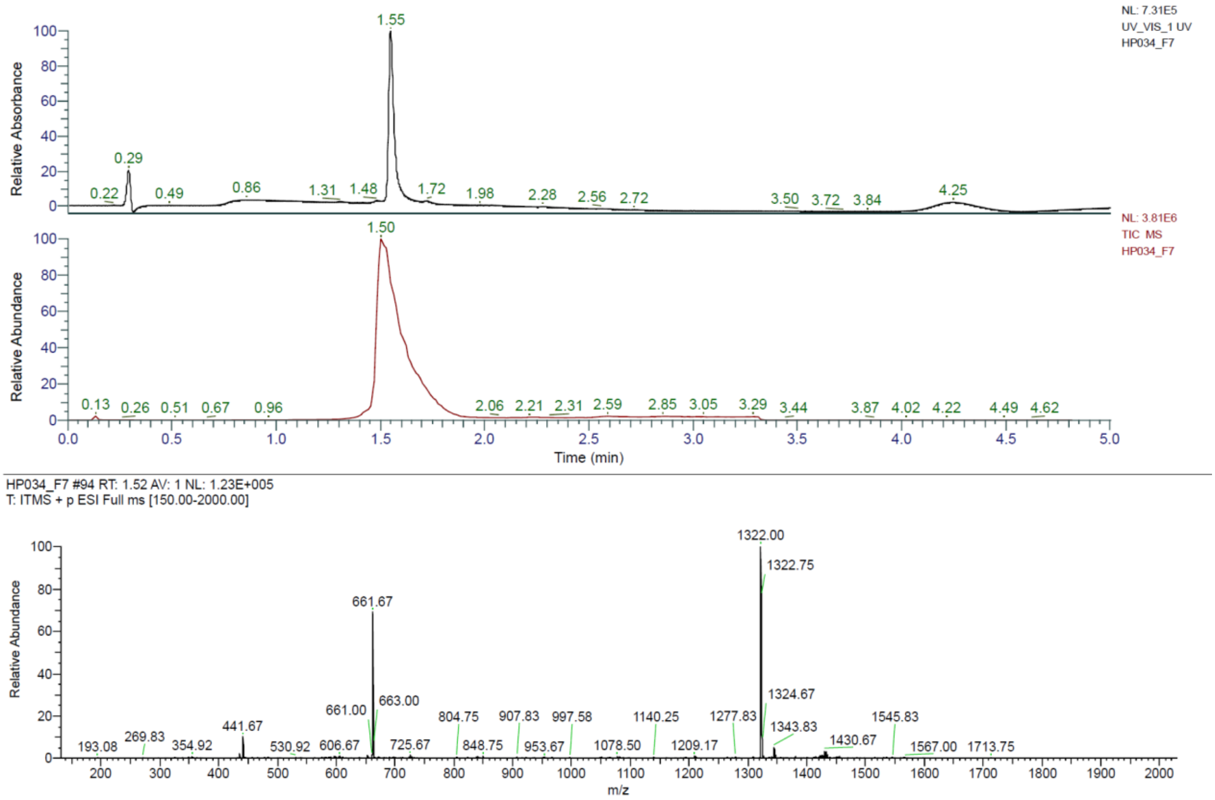

HRMS spectra:

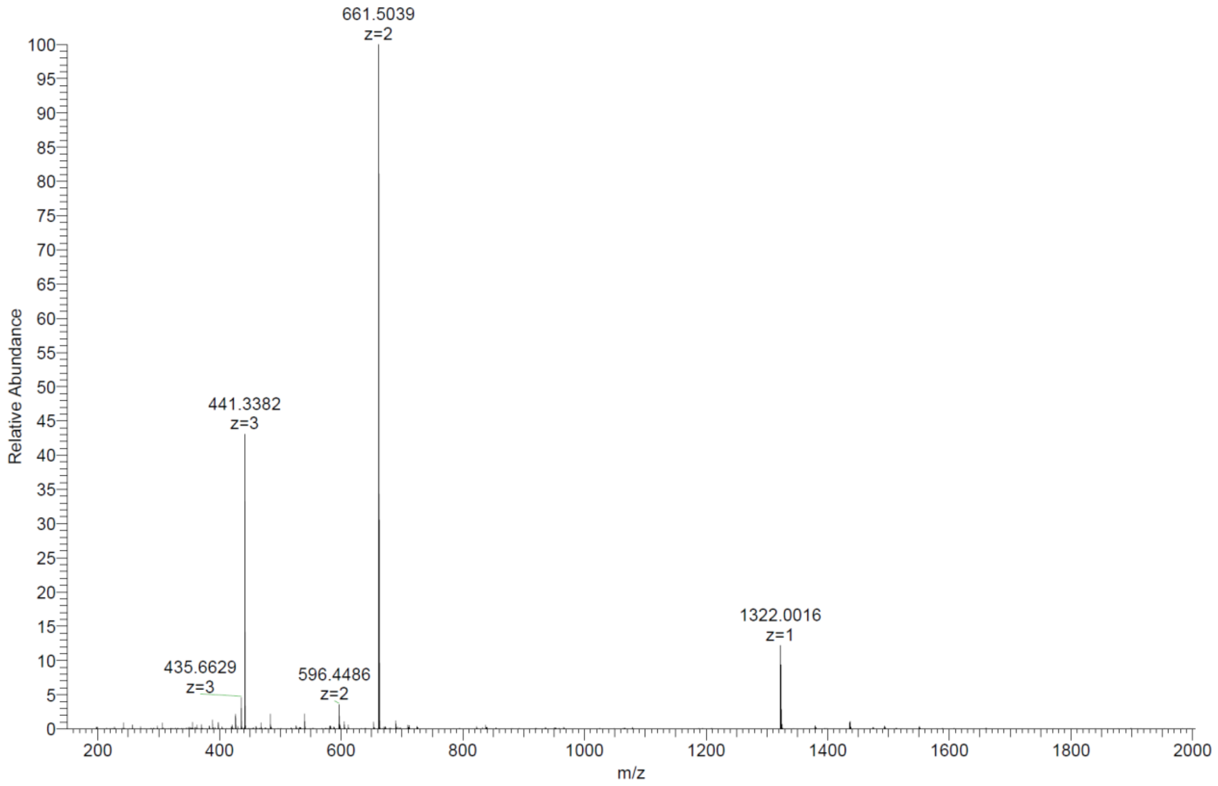

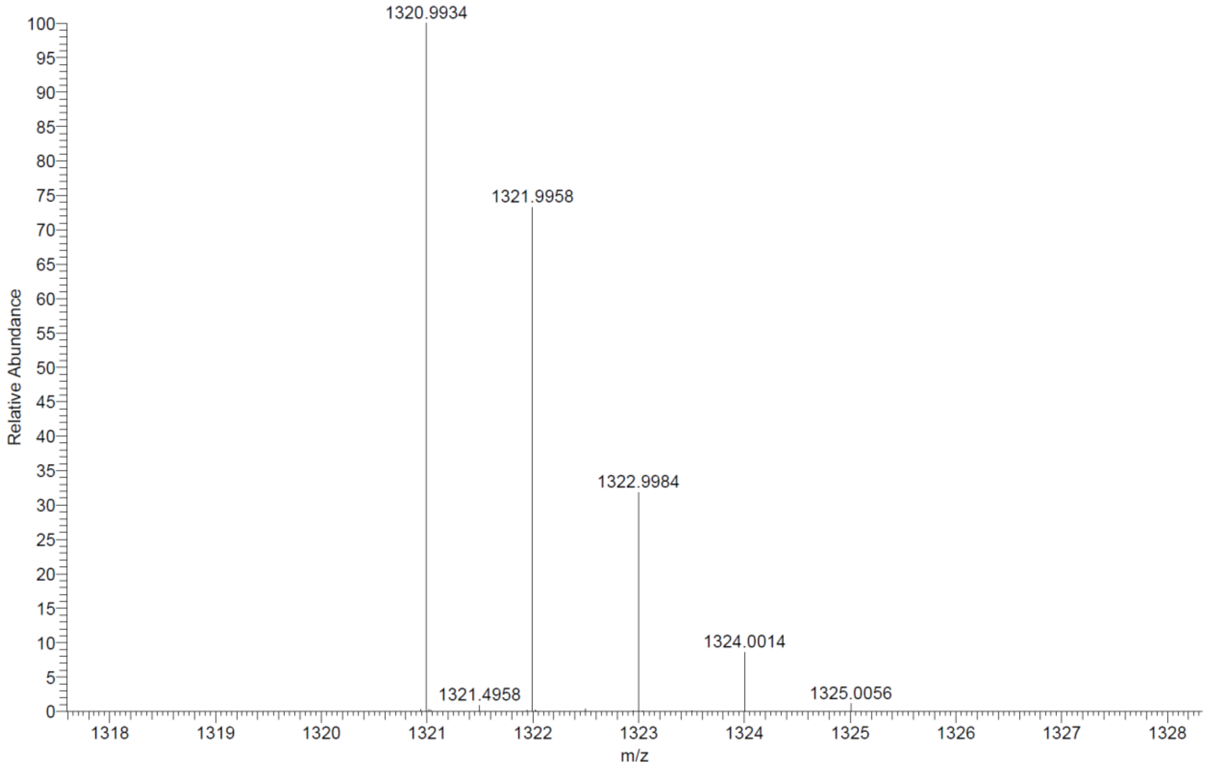

KKLlkllkLLL (HP29) was obtained as white solid after preparative RP-HPLC (59.3 mg, 53.4%). Analytical RP-HPLC: t_R = 1.58 min (A/D 100:0 to 0:100 in 3.5 min, λ = 214 nm). MS (ESI+): $C_{66}H_{128}N_{16}O_{11}$ calc./obs. 1321.99/1322.00 Da $[M+H]^+$.

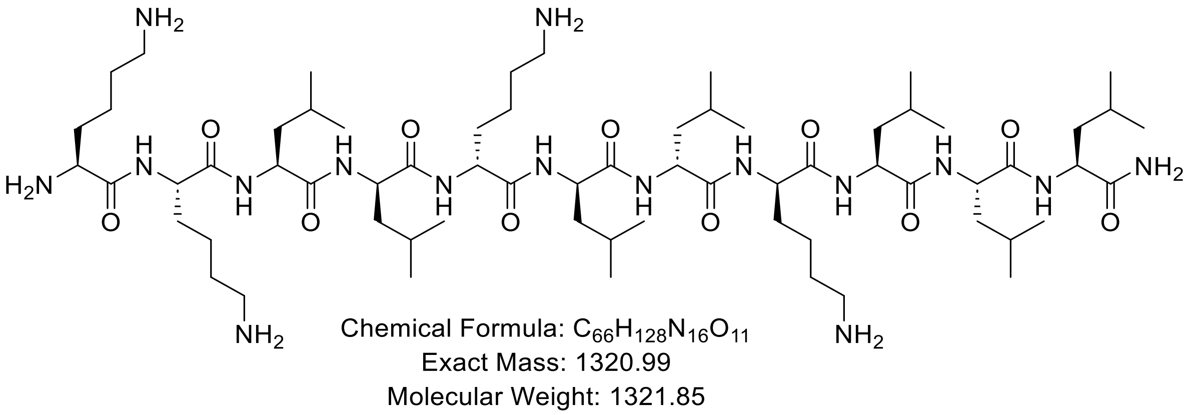

Analytical HPLC-MS data:

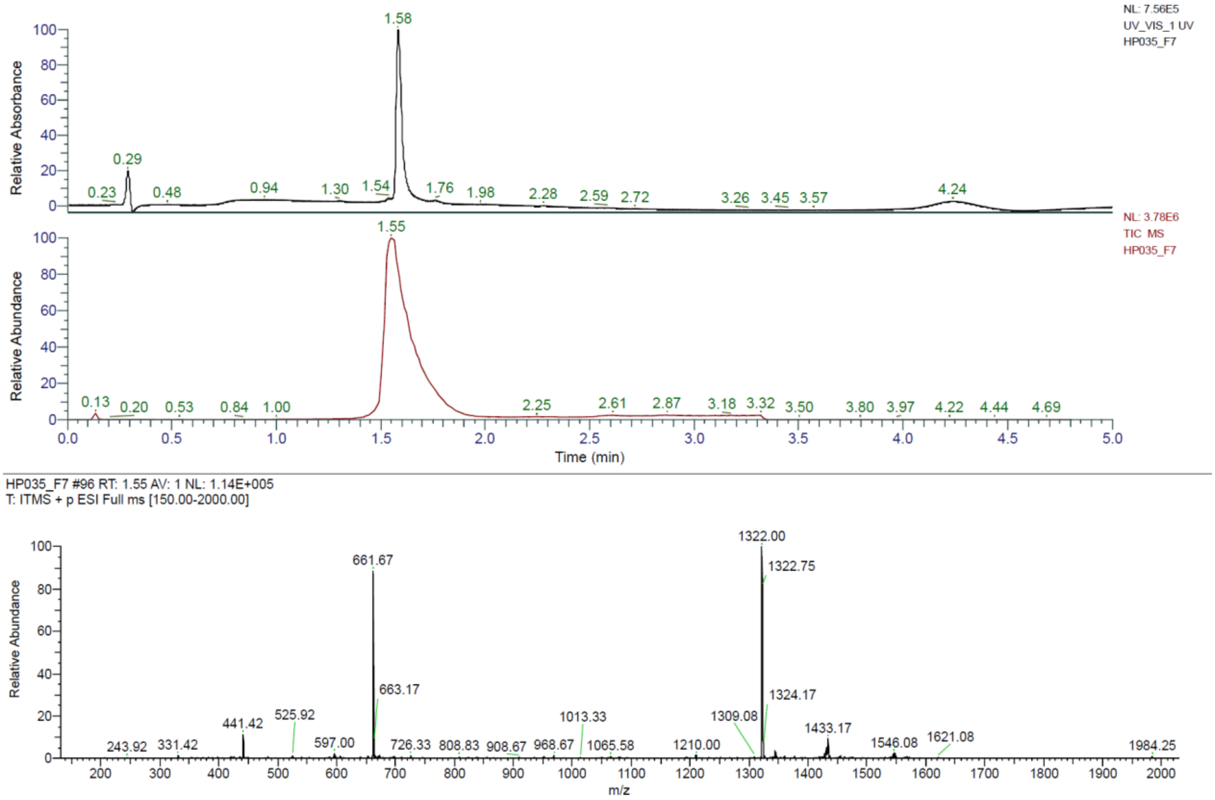

HRMS spectra:

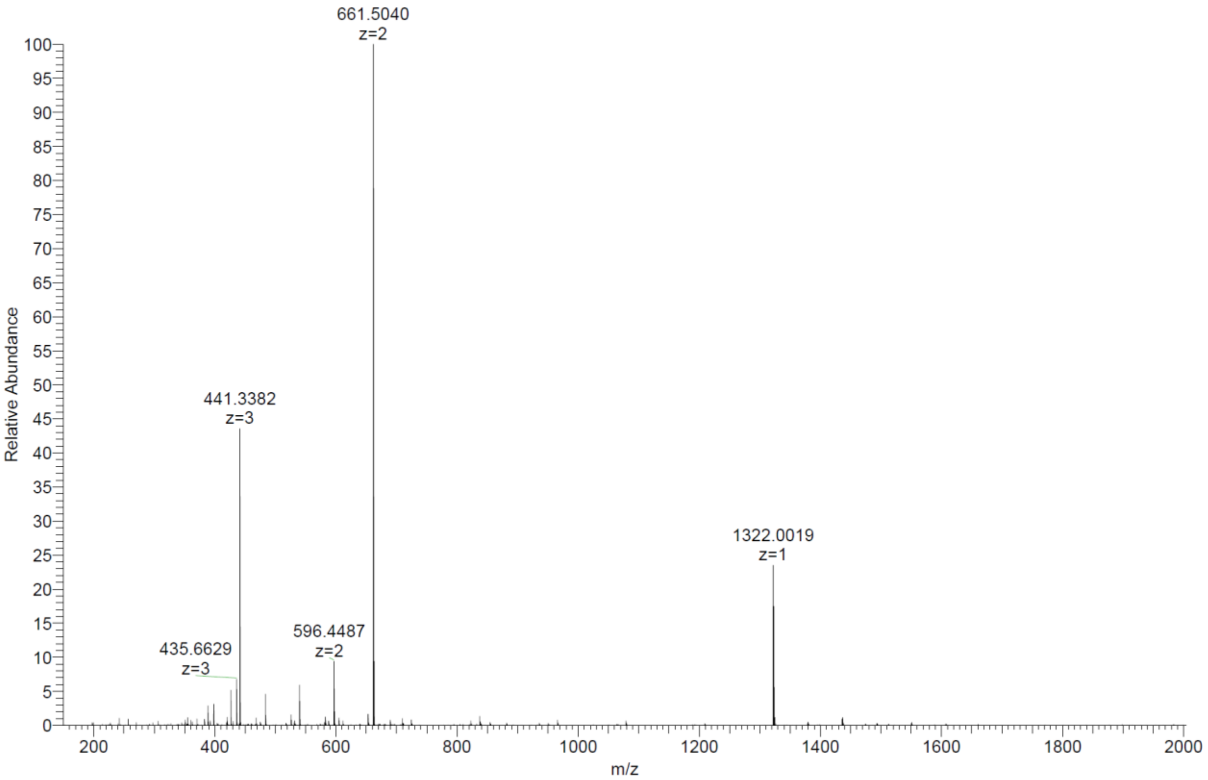

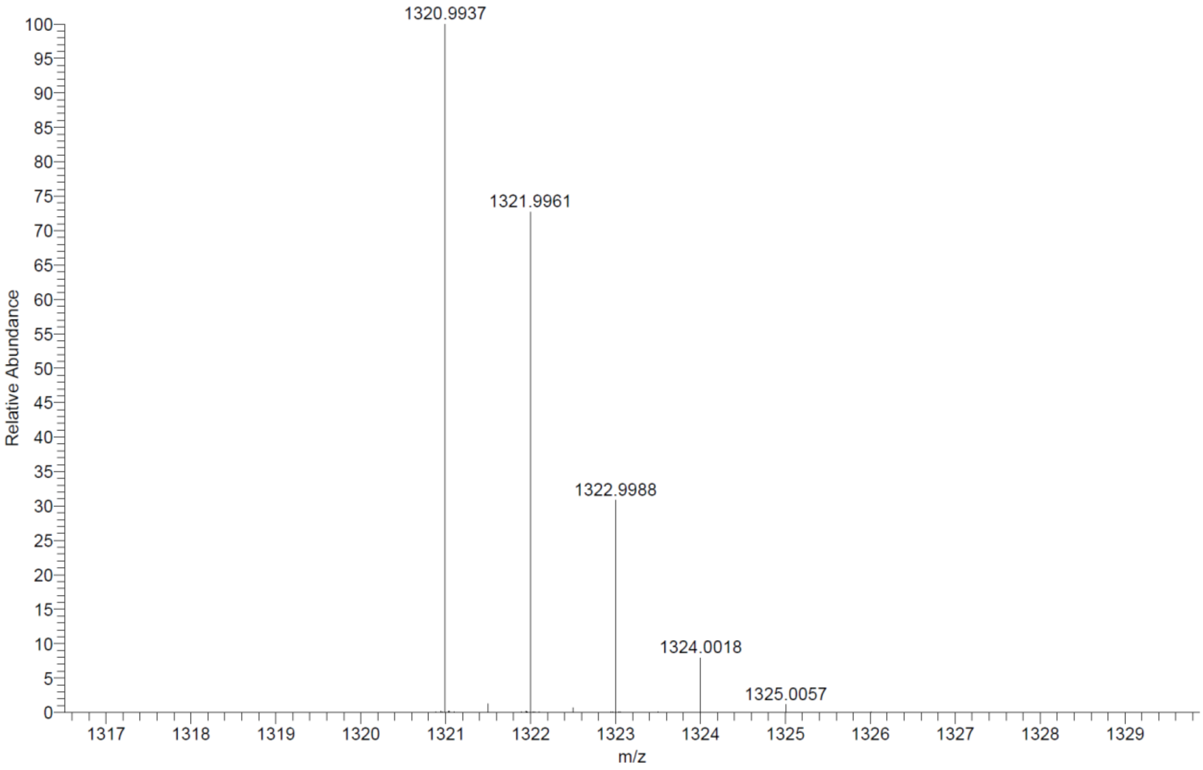

KkLlKlLkLlL (HP30) was obtained as white solid after preparative RP-HPLC (35.7 mg, 32.1%). Analytical RP-HPLC: t_R = 1.57 min (A/D 100:0 to 0:100 in 3.5 min, λ = 214 nm). MS (ESI+): $C_{66}H_{128}N_{16}O_{11}$ calc./obs. 1321.99/1322.00 Da $[M+H]^+$.

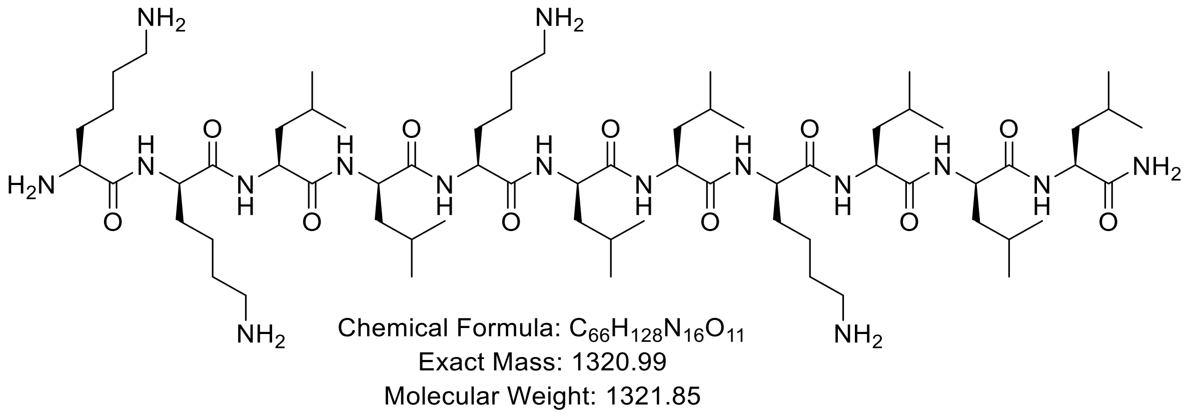

Analytical HPLC-MS data:

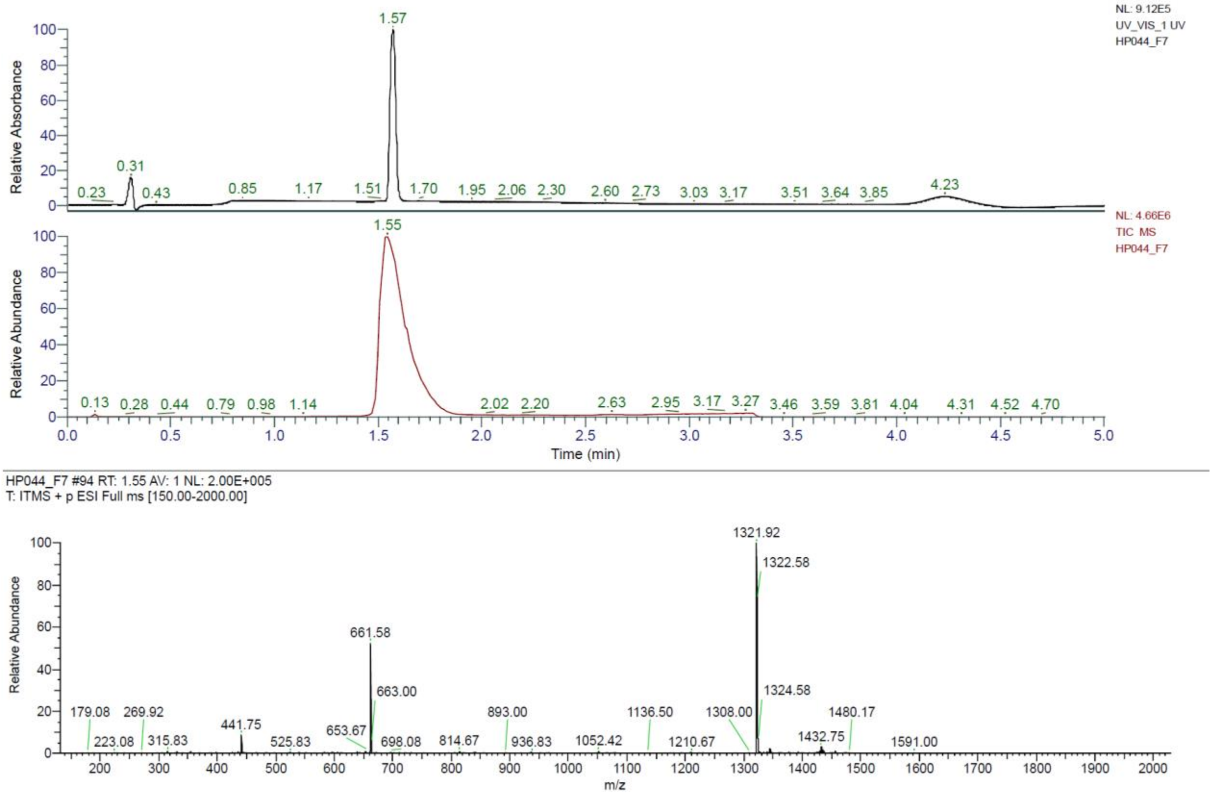

HRMS spectra:

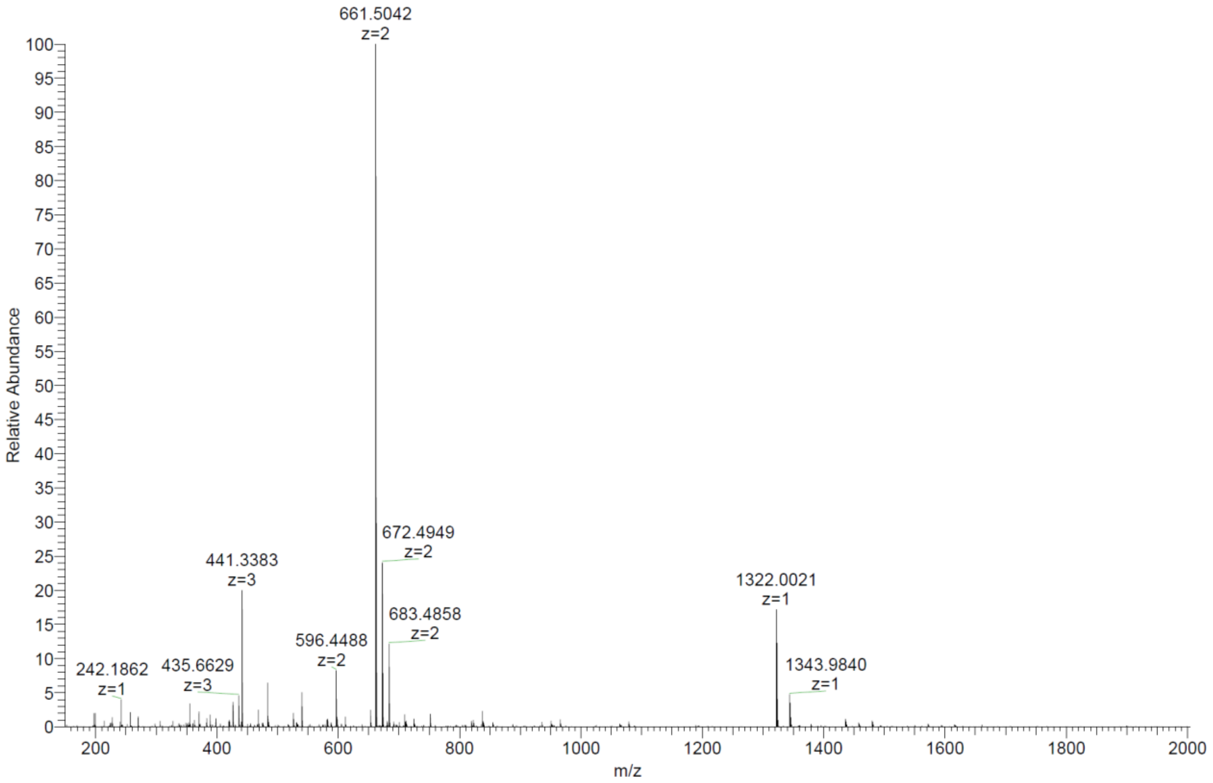

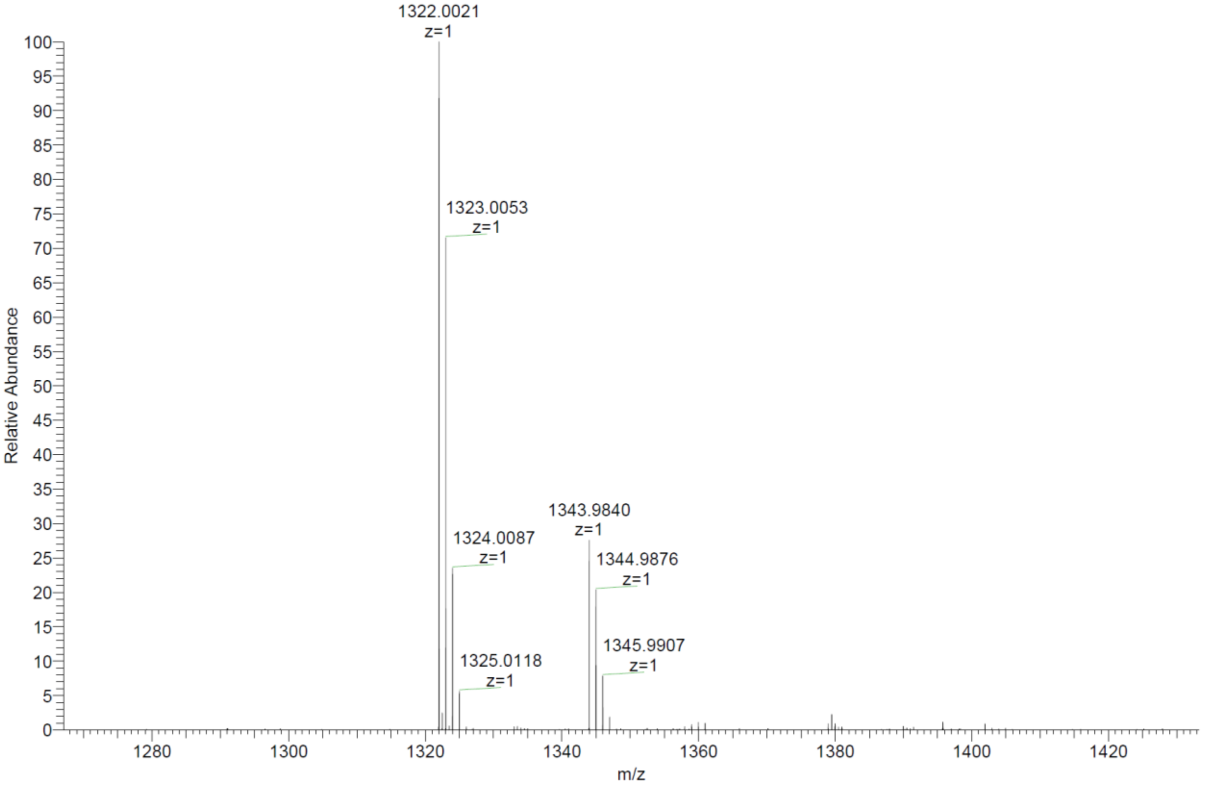

kKlLkLlKlLl (HP31) was obtained as white solid after preparative RP-HPLC (45.3 mg, 40.8%). Analytical RP-HPLC: t_R = 1.57 min (A/D 100:0 to 0:100 in 3.5 min, λ = 214 nm). MS (ESI+): $C_{66}H_{128}N_{16}O_{11}$ calc./obs. 1321.99/1322.00 Da $[M+H]^+$.

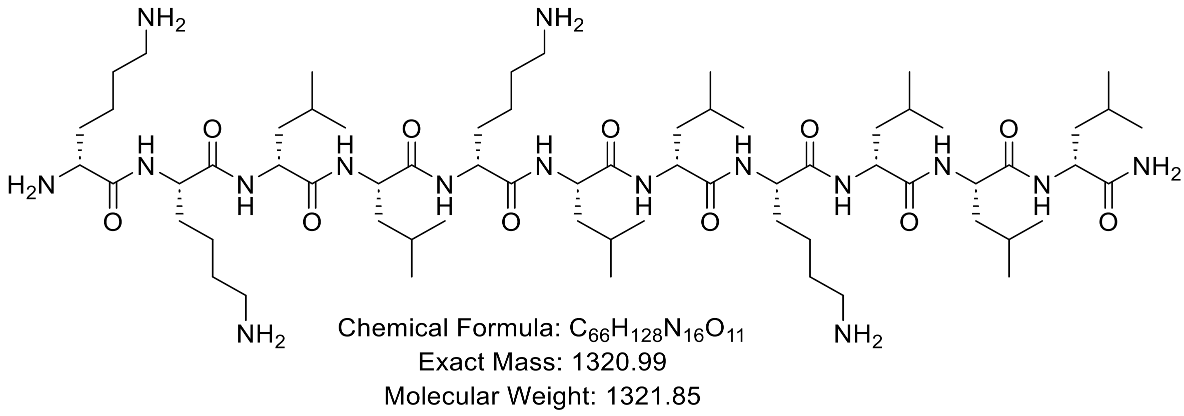

Analytical HPLC-MS data:

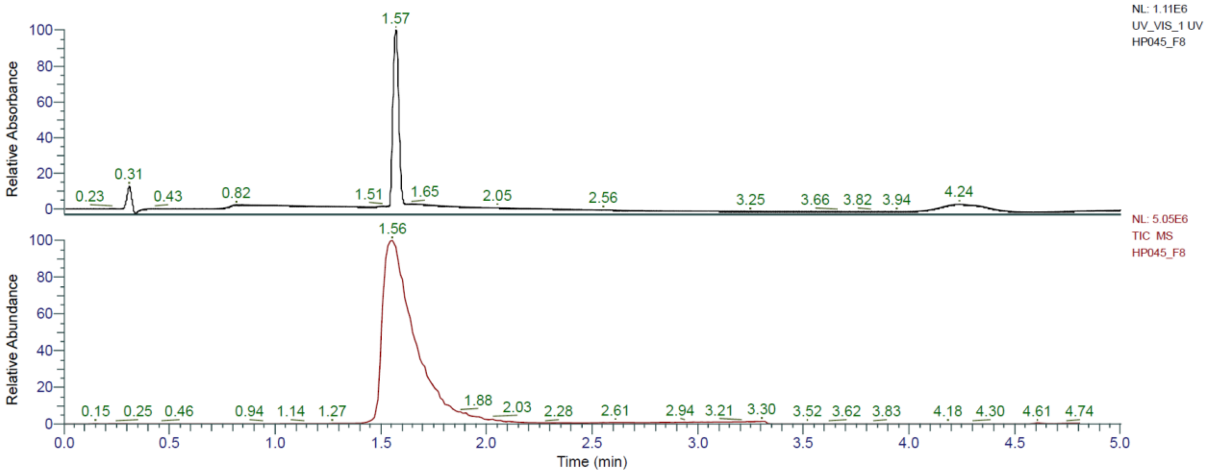

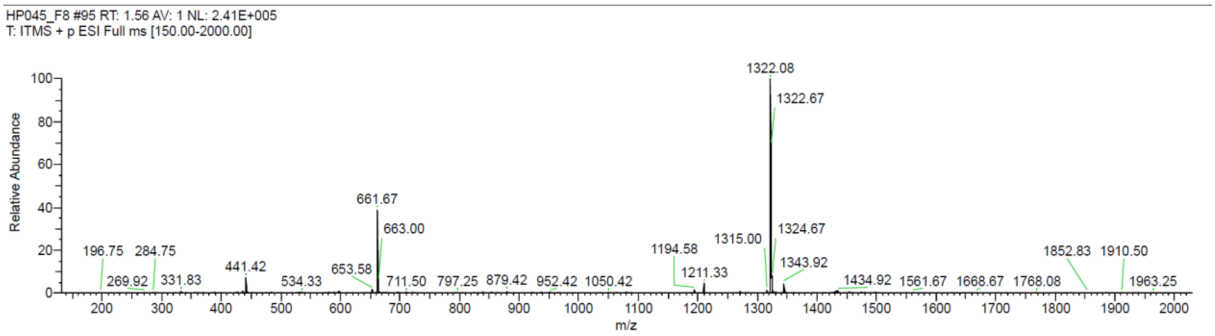

HRMS spectra:

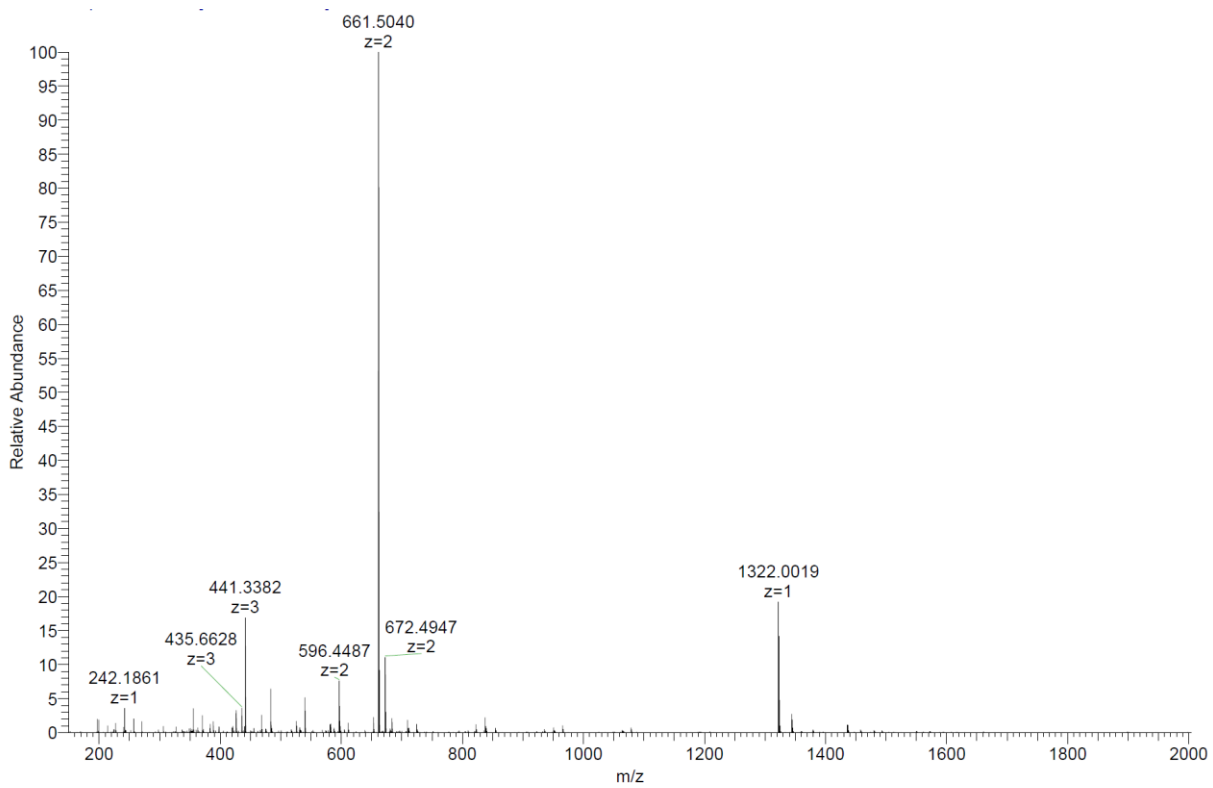

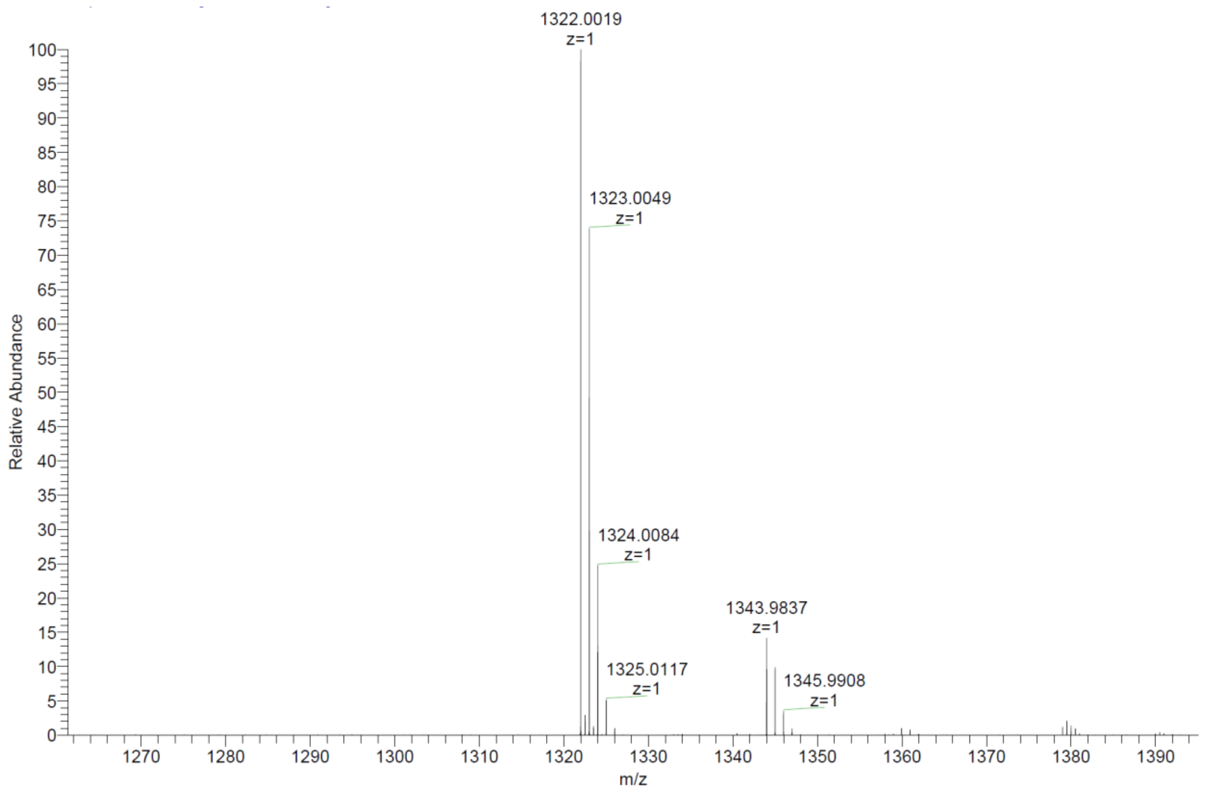

RRLLRLLRLLL (HP32) was obtained as white solid after preparative RP-HPLC (32.5 mg, 27.5%). Analytical RP-HPLC: t_R = 1.64 min (A/D 100:0 to 0:100 in 3.5 min, λ = 214 nm). MS (ESI+): $C_{66}H_{128}N_{24}O_{11}$ calc./obs. 1434.02/1434.03 Da $[M+H]^+$.

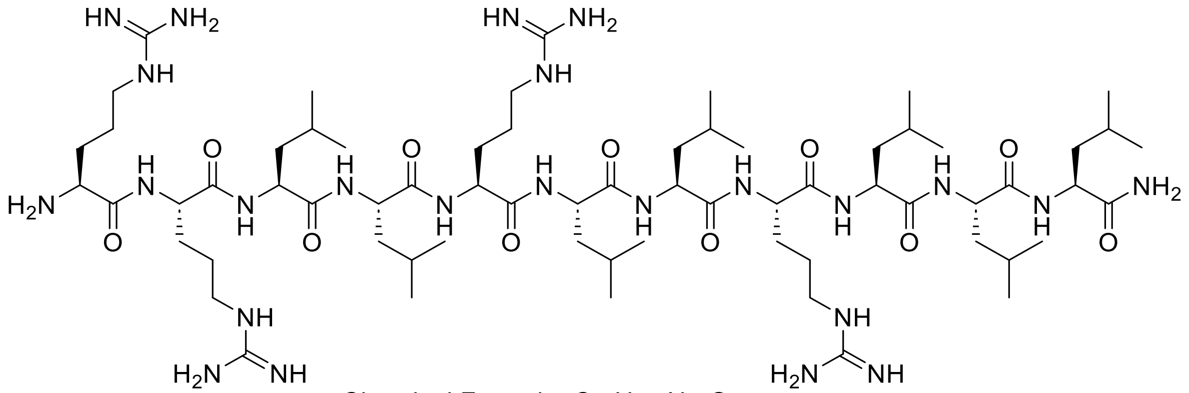

Chemical Formula: $C_{66}H_{128}N_{24}O_{11}$
Exact Mass: 1433.02
Molecular Weight: 1433.91

Analytical HPLC-MS data:

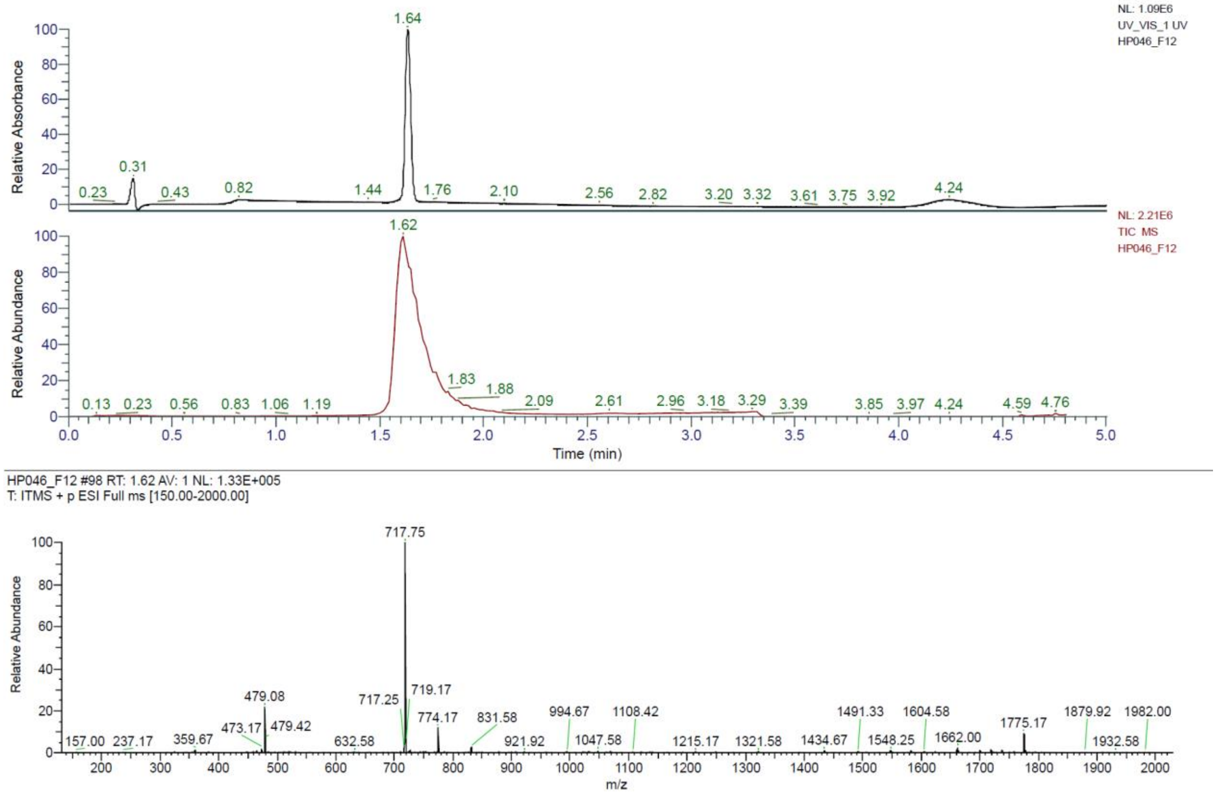

HRMS spectra:

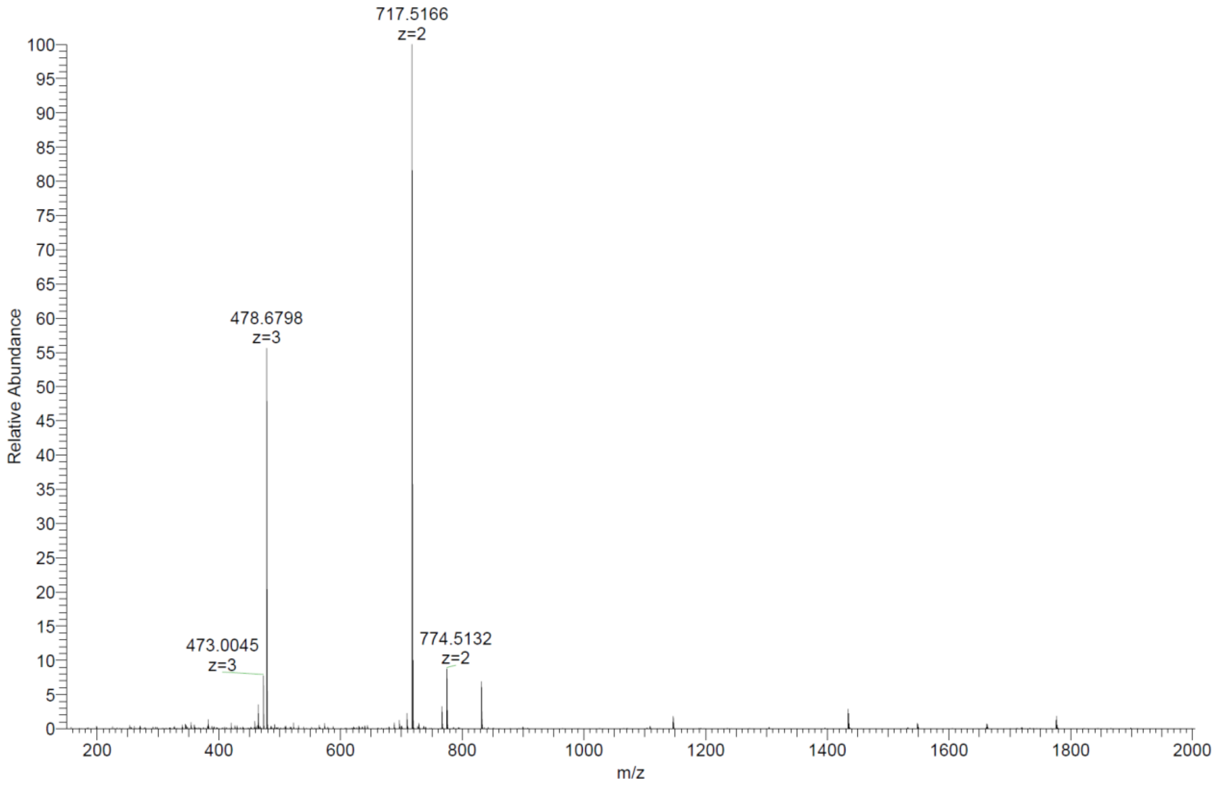

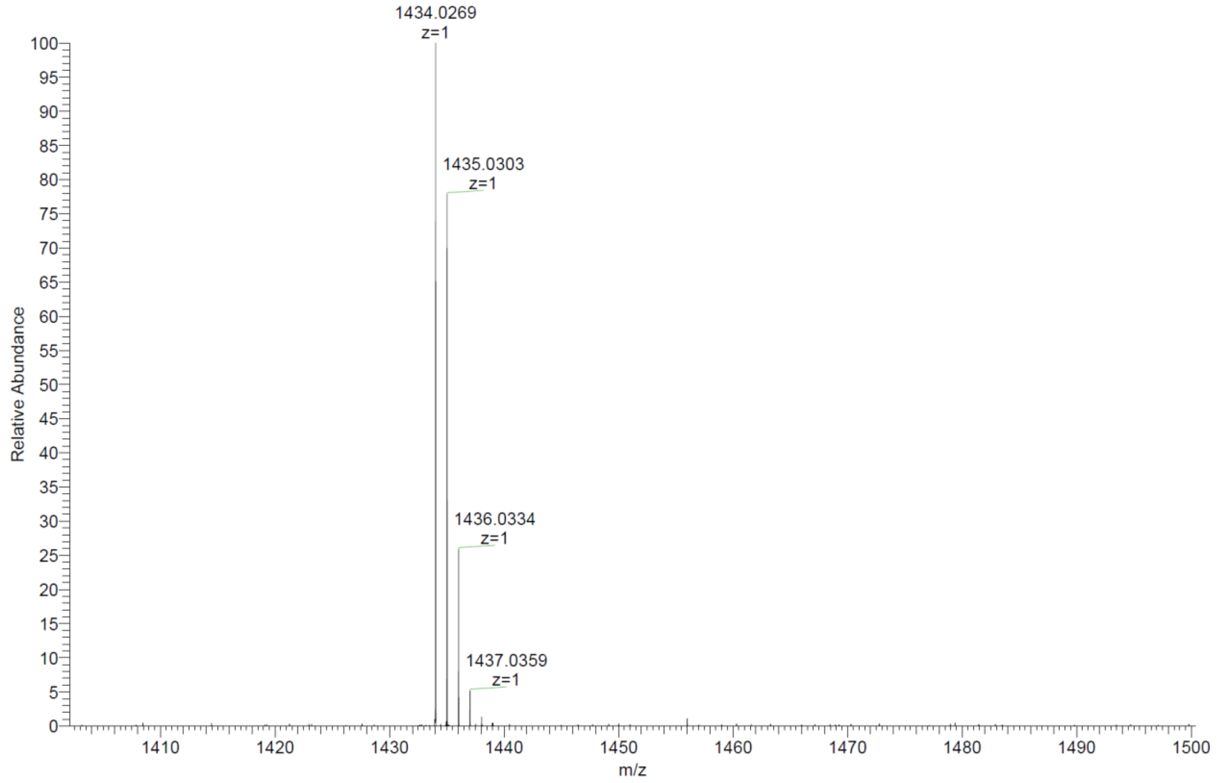

rrLLrLLrLLL (HP33) was obtained as white solid after preparative RP-HPLC (44.3 mg, 37.5%). Analytical RP-HPLC: t_R = 1.52 min (A/D 100:0 to 0:100 in 3.5 min, λ = 214 nm). MS (ESI+): $C_{66}H_{128}N_{24}O_{11}$ calc./obs. 1434.02/1434.03 Da $[M+H]^+$.

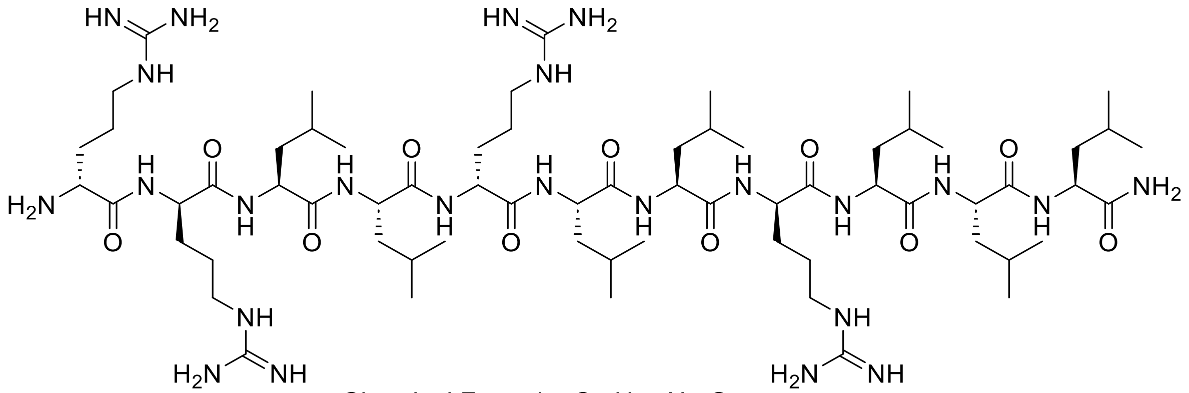

Chemical Formula: $C_{66}H_{128}N_{24}O_{11}$
Exact Mass: 1433.02
Molecular Weight: 1433.91

Analytical HPLC-MS data:

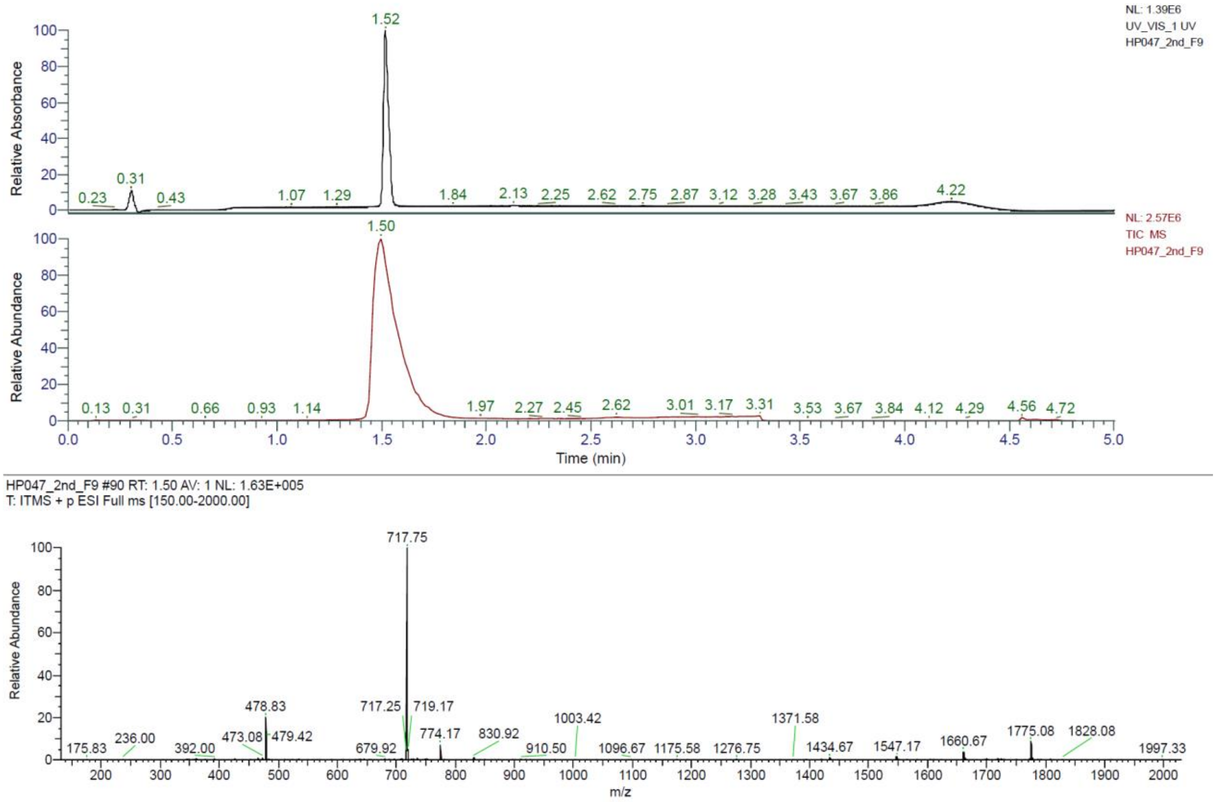

HRMS spectra:

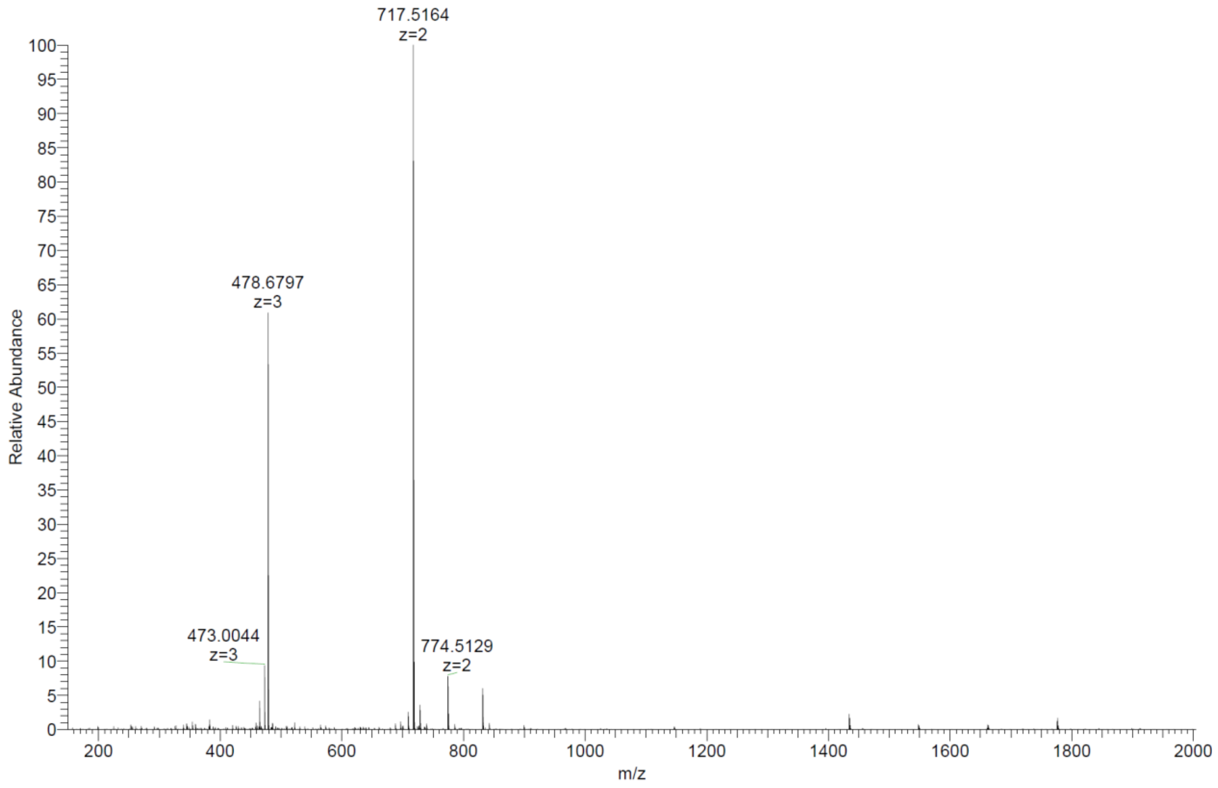

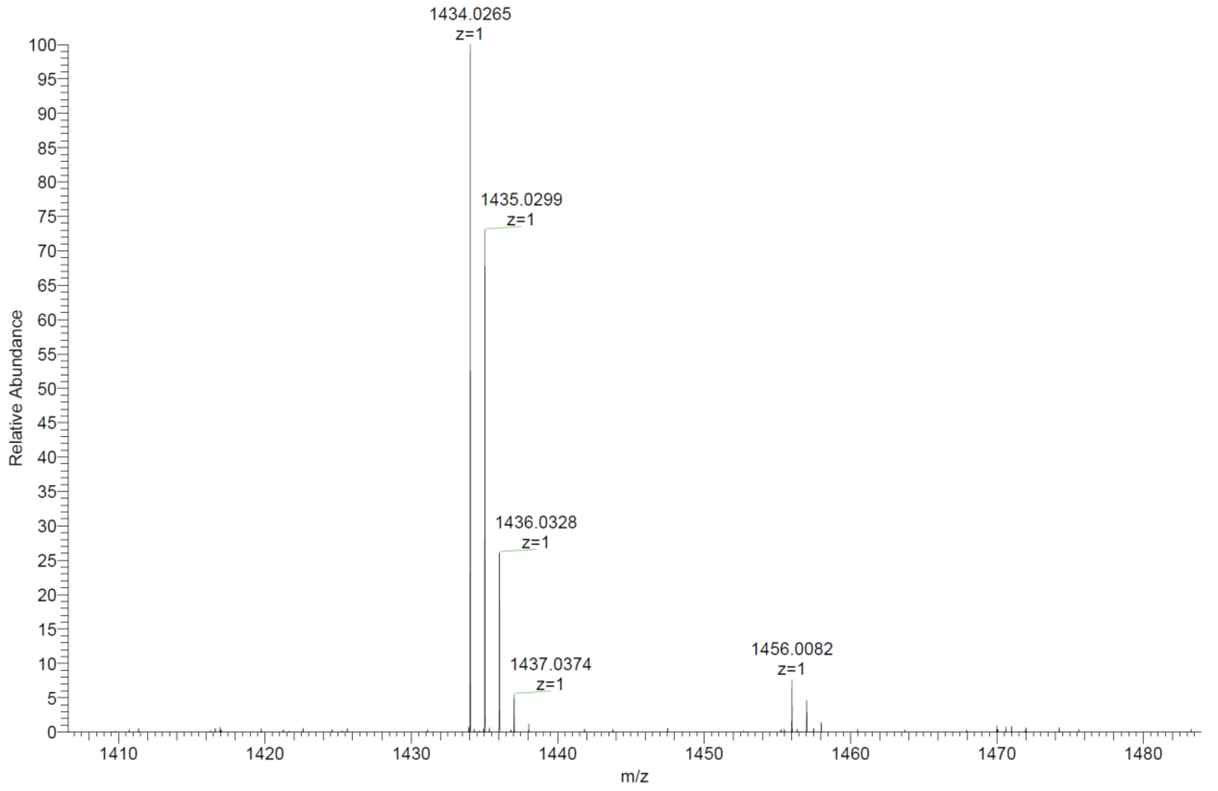

KKIIKIIKIII (HP34) was obtained as white solid after preparative RP-HPLC (35.1 mg, 31.6%). Analytical RP-HPLC: t_R = 1.44 min (A/D 100:0 to 0:100 in 3.5 min, λ = 214 nm). MS (ESI+): $C_{66}H_{128}N_{16}O_{11}$ calc./obs. 1321.99/1322.00 Da $[M+H]^+$.

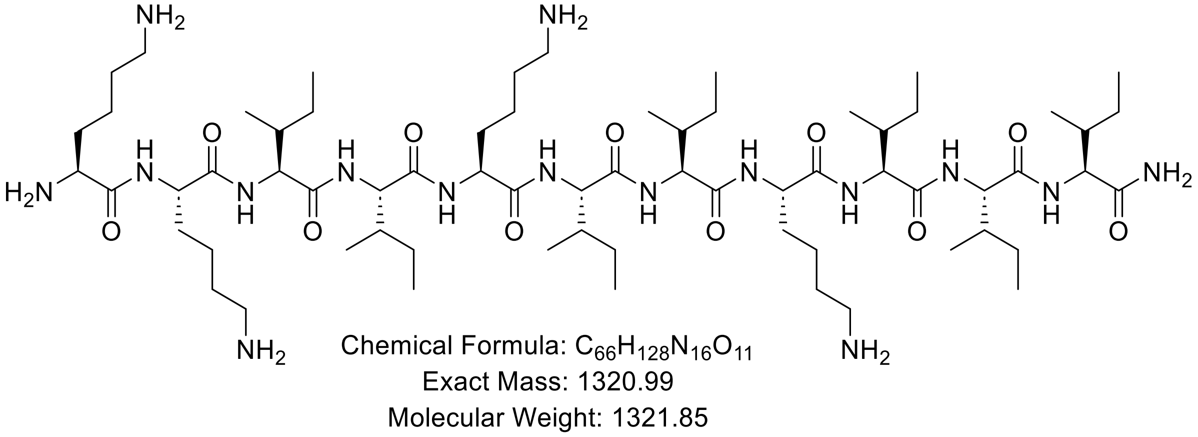

Analytical HPLC-MS data:

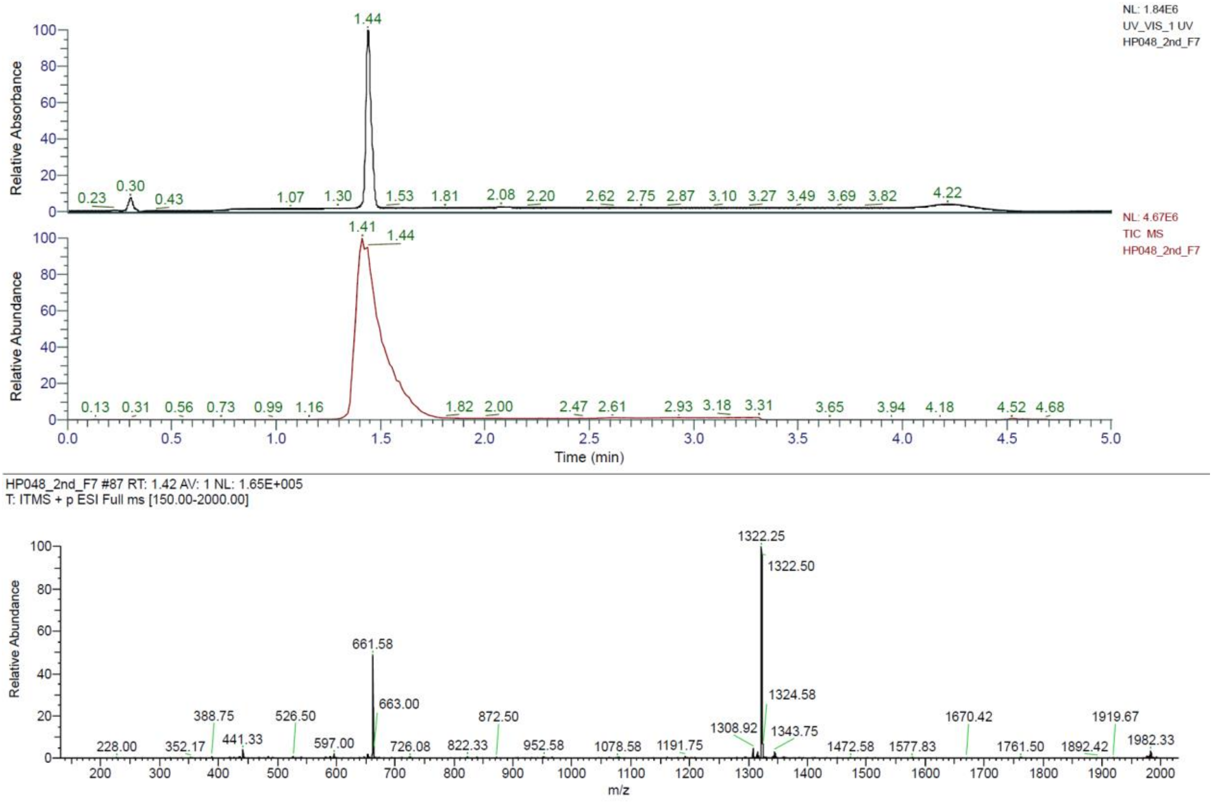

HRMS spectra:

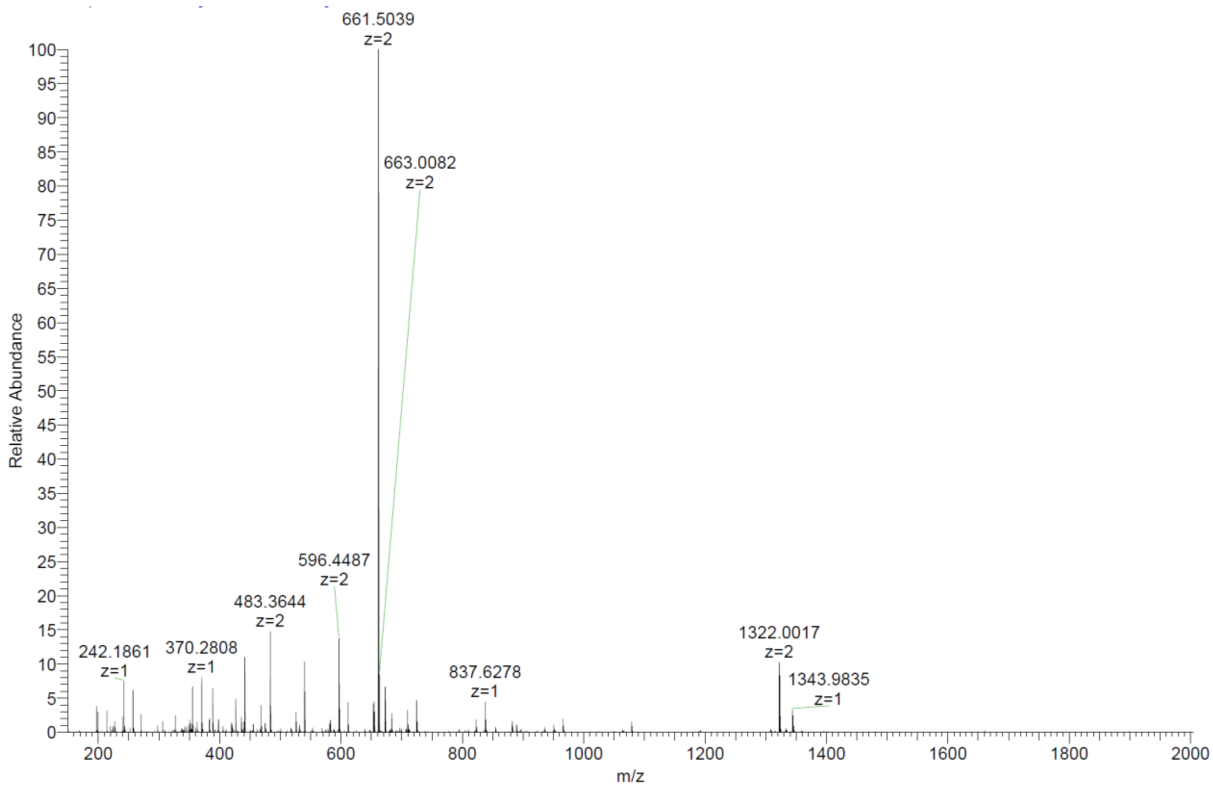

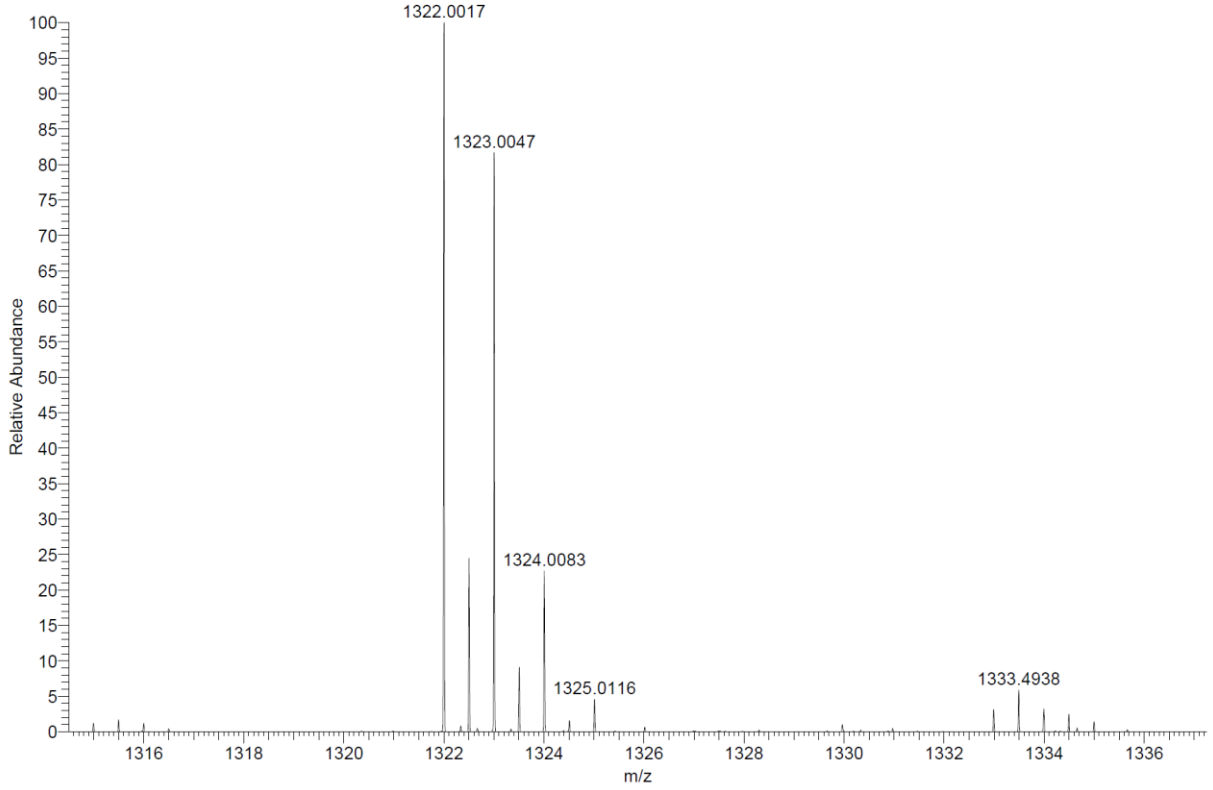

kkIIkIIkIII (HP35) was obtained as white solid after preparative RP-HPLC (29.3 mg, 26.4%). Analytical RP-HPLC: t_R = 1.36 min (A/D 100:0 to 0:100 in 3.5 min, λ = 214 nm). MS (ESI+): $C_{66}H_{128}N_{16}O_{11}$ calc./obs. 1321.99/1322.00 Da $[M+H]^+$.

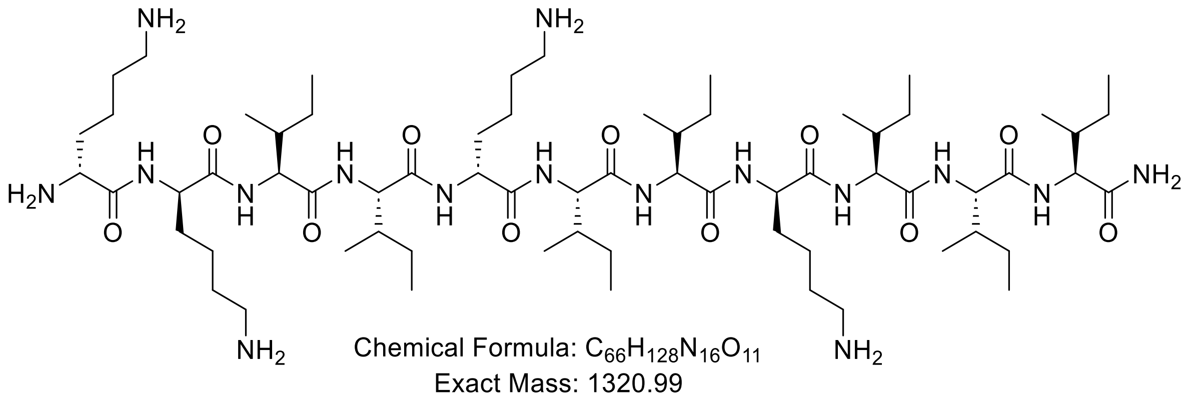

Analytical HPLC-MS data:

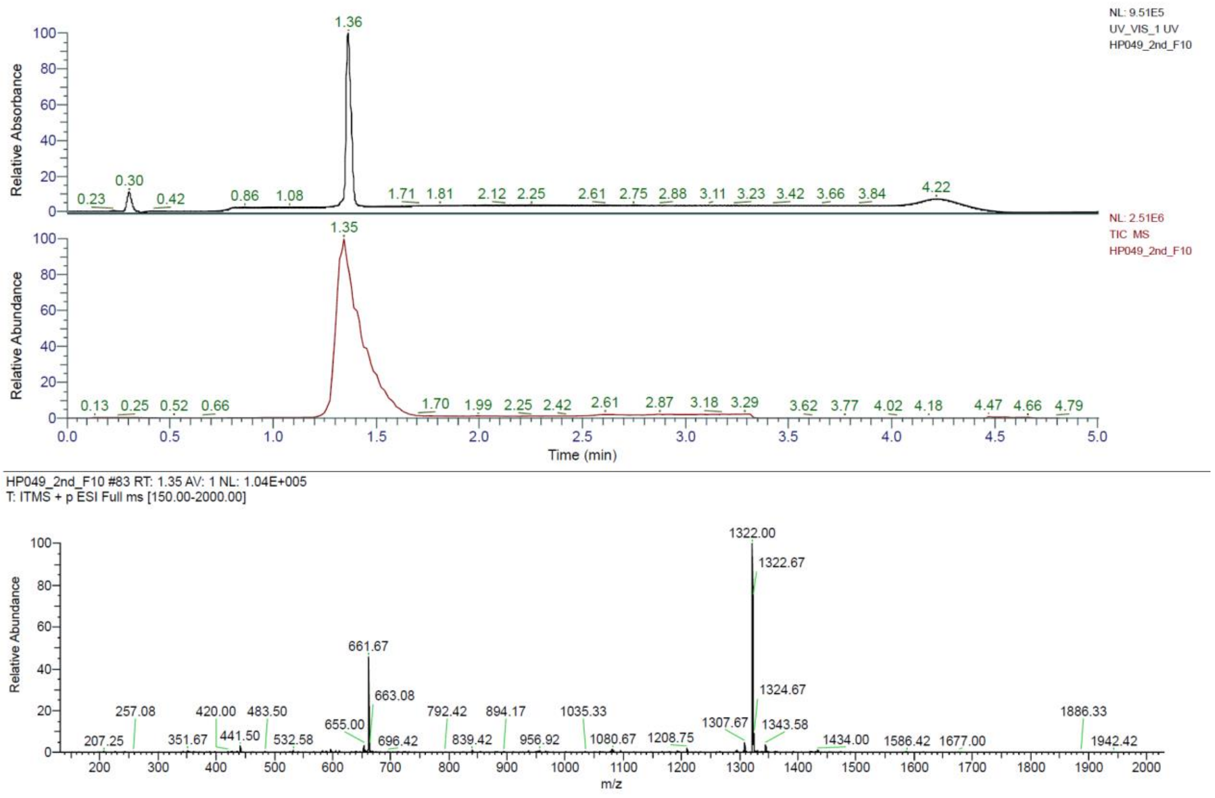

HRMS spectra:

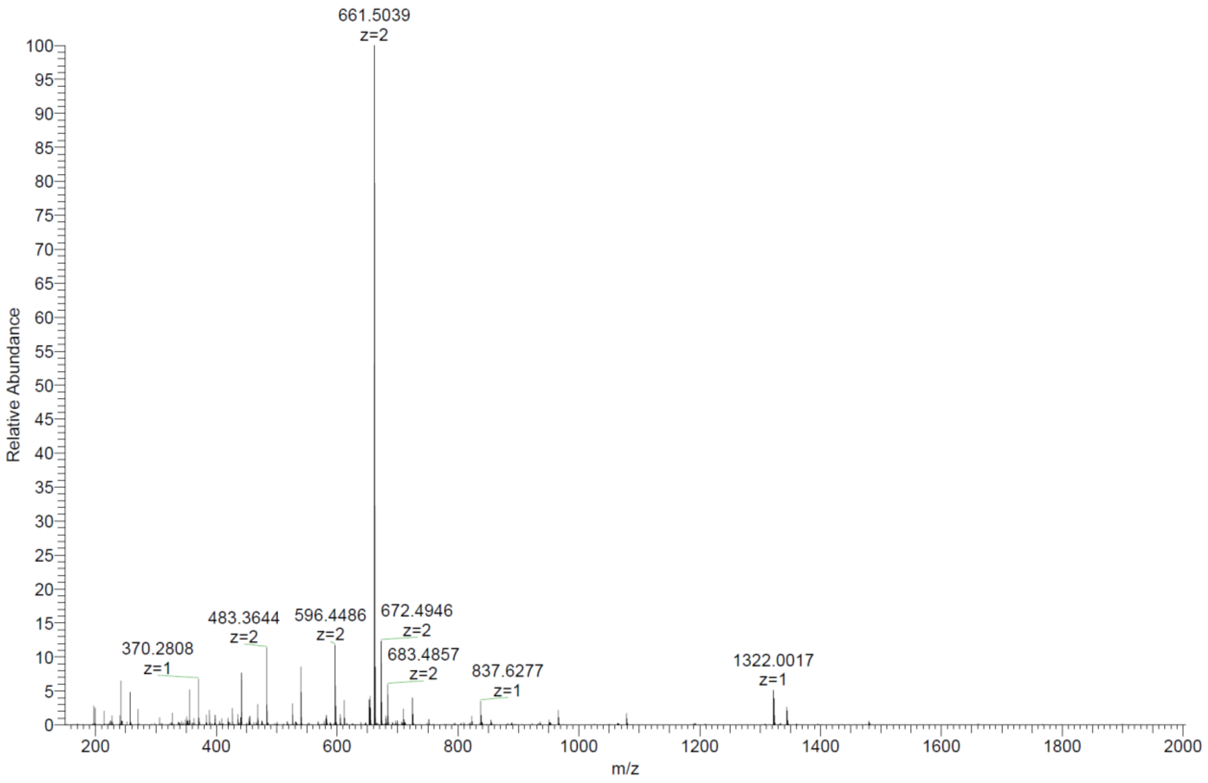

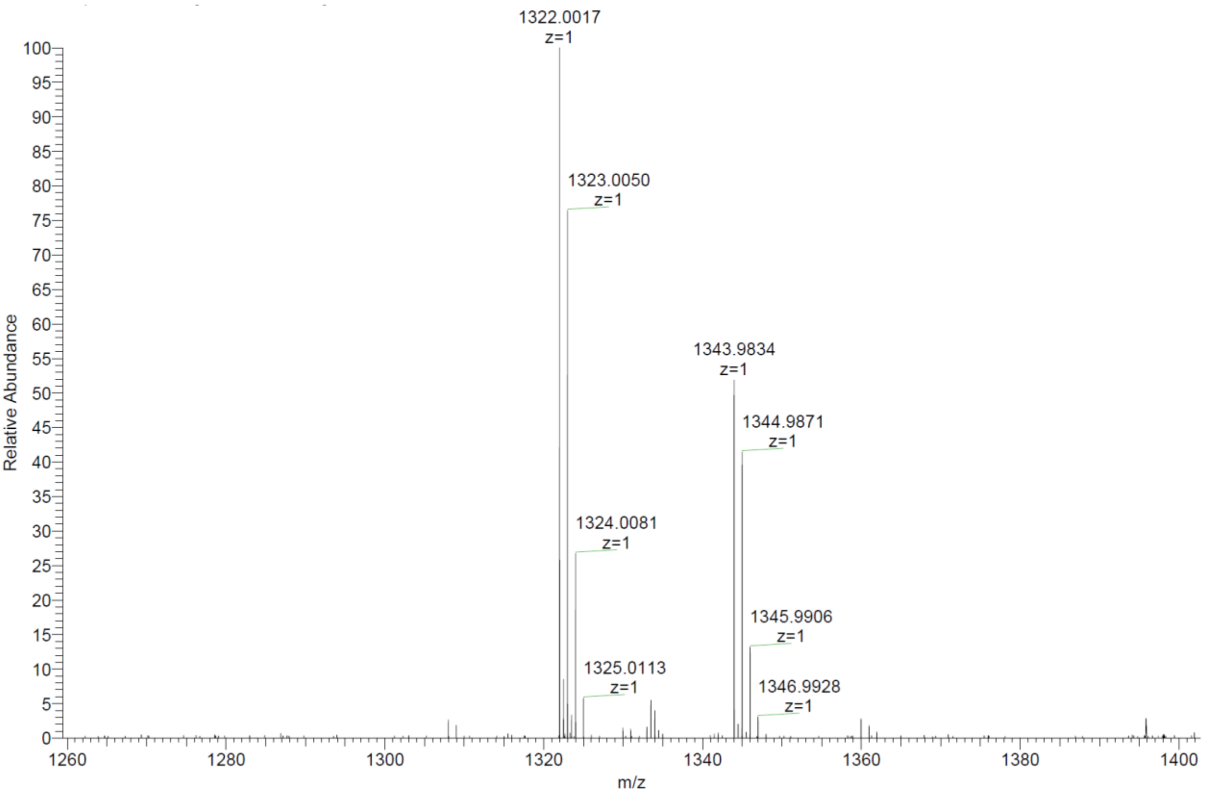

RRIIRIIRIII (HP36) was obtained as white solid after preparative RP-HPLC (7.7 mg, 6.5%). Analytical RP-HPLC: t_R = 1.47 min (A/D 100:0 to 0:100 in 3.5 min, λ = 214 nm). MS (ESI+): $C_{66}H_{128}N_{24}O_{11}$ calc./obs. 1434.02/1434.03 Da $[M+H]^+$.

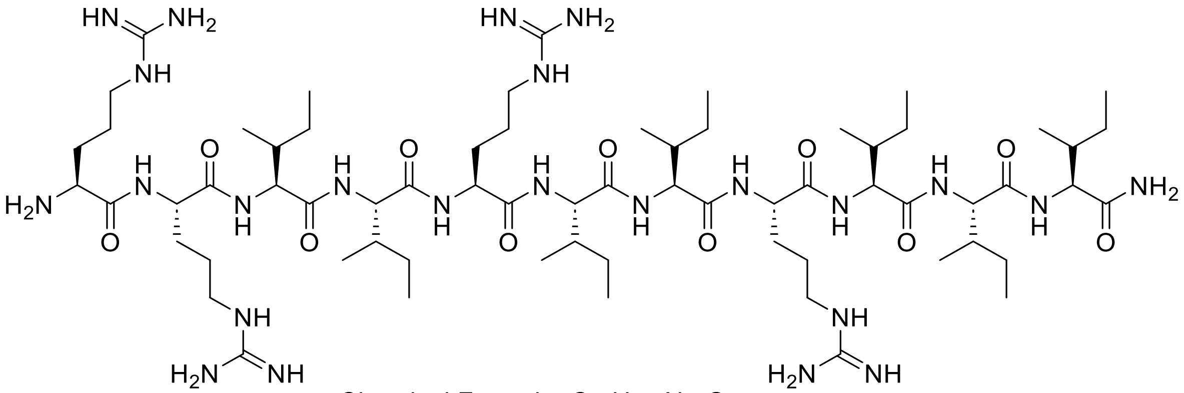

Chemical Formula: $C_{66}H_{128}N_{24}O_{11}$
Exact Mass: 1433.02
Molecular Weight: 1433.91

Analytical HPLC-MS data:

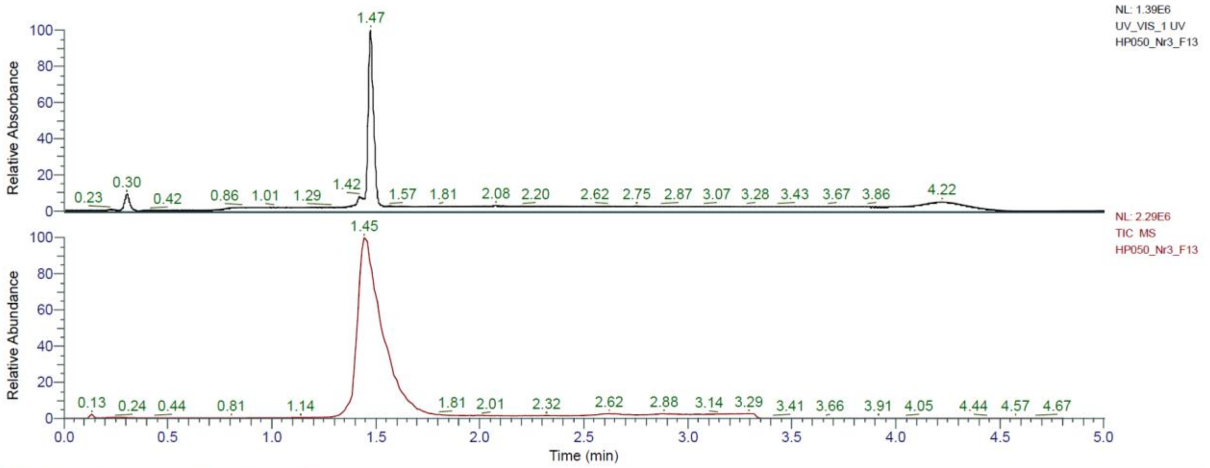

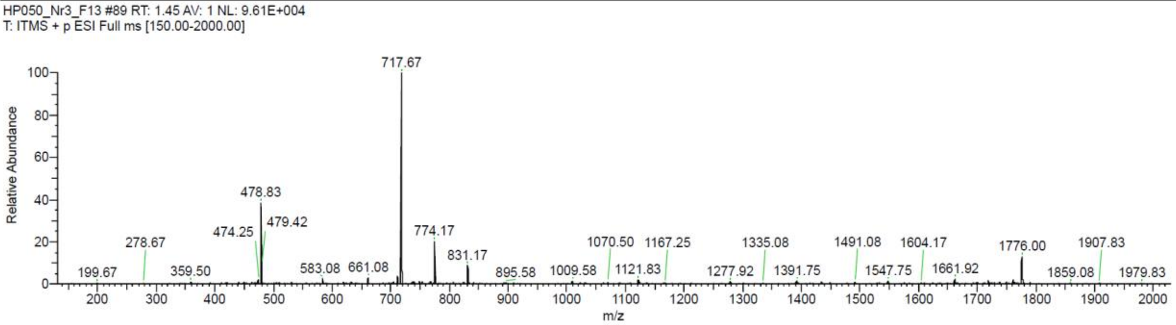

HRMS spectra:

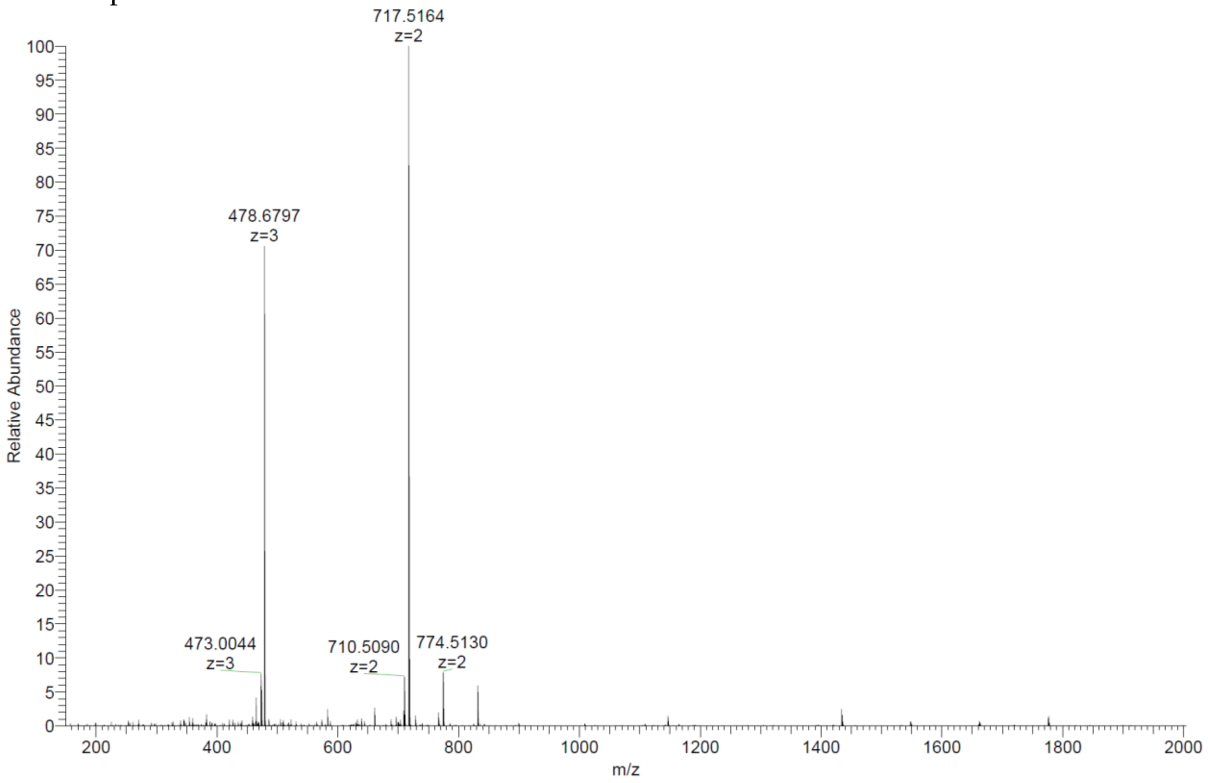

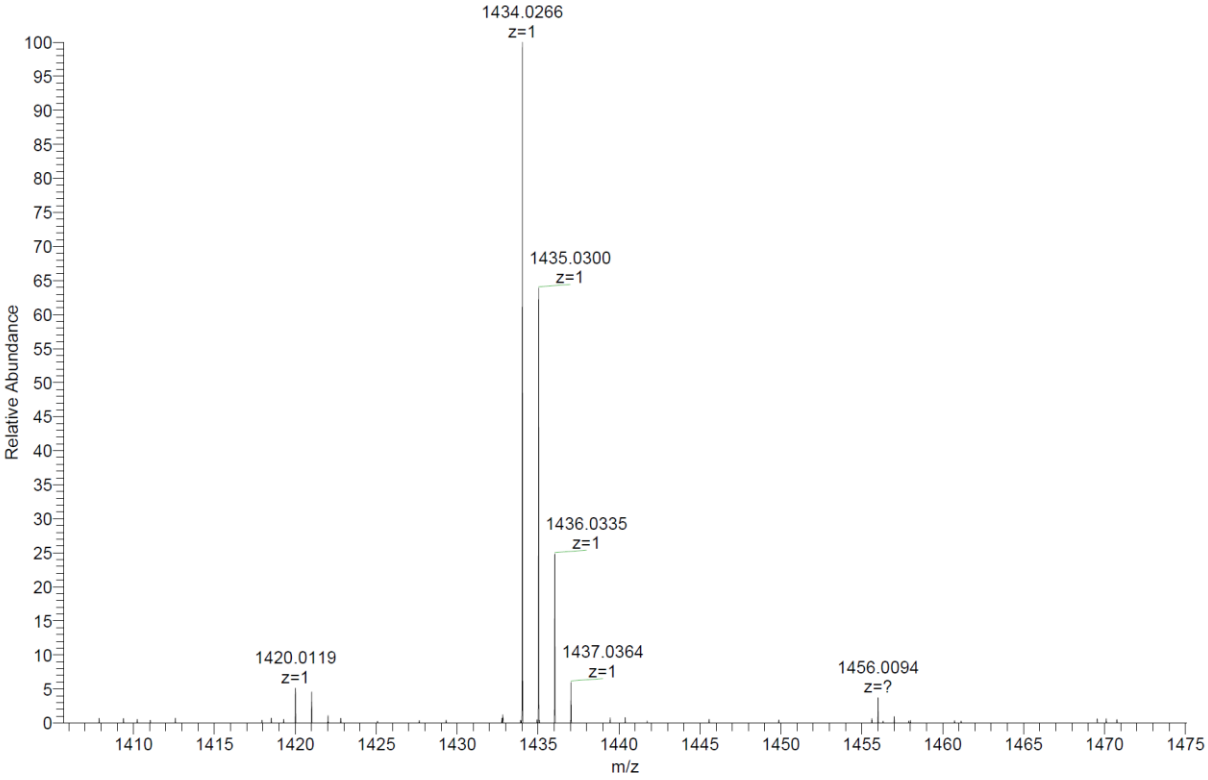

rrIIrIIrIII (HP37) was obtained as white solid after preparative RP-HPLC (38.7 mg, 32.8%). Analytical RP-HPLC: t_R = 1.68 min (A/D 100:0 to 0:100 in 3.5 min, λ = 214 nm). MS (ESI+): $C_{66}H_{128}N_{24}O_{11}$ calc./obs. 1434.02/1434.03 Da $[M+H]^+$.

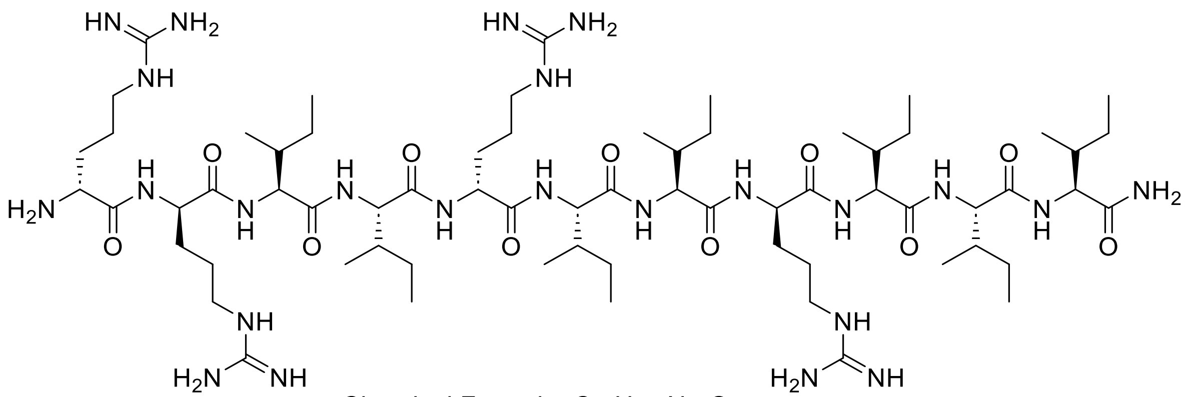

Chemical Formula: $C_{66}H_{128}N_{24}O_{11}$
Exact Mass: 1433.02
Molecular Weight: 1433.91

Analytical HPLC-MS data:

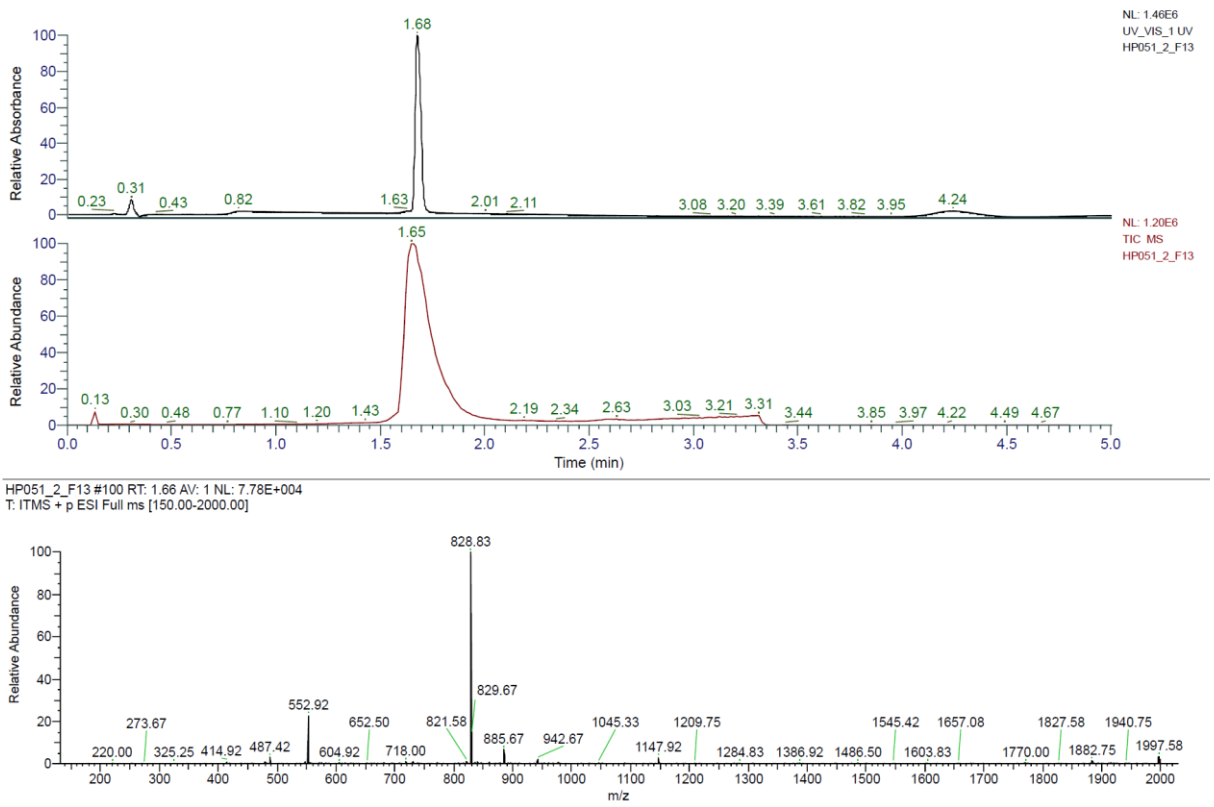

HRMS spectra:

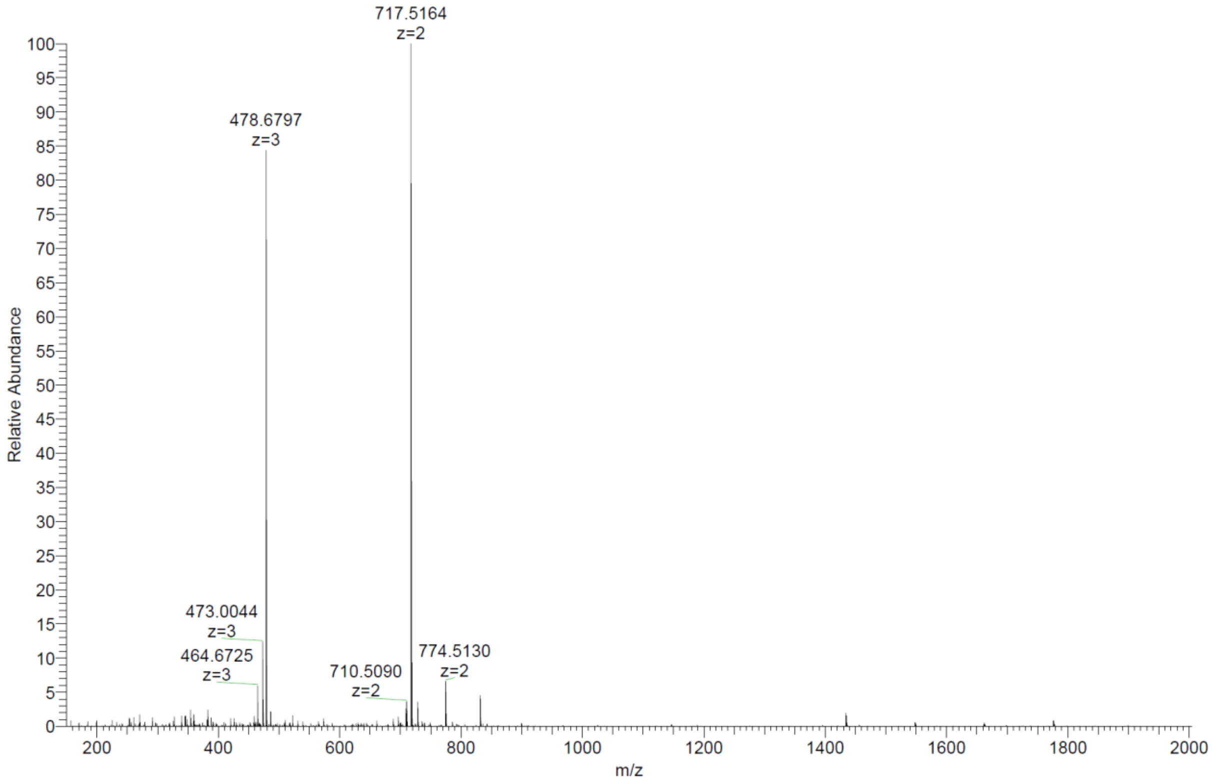

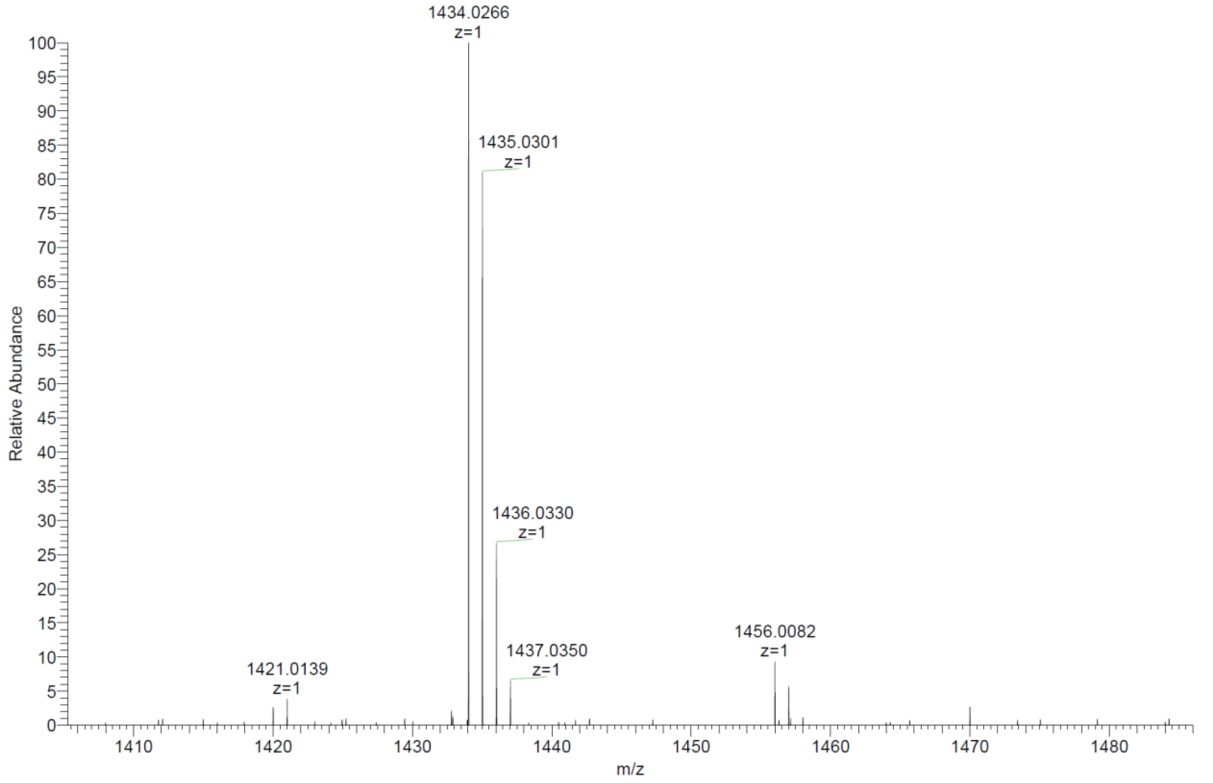

KKLLKLLKLLLKKLLKLLKLLL (2ln65) was obtained as white solid after preparative RP-HPLC (119.3 mg, 38.3%). Analytical RP-HPLC: t_R = 2.38 min (A/D 100:0 to 0:100 in 3.5 min, λ = 214 nm). MS (ESI+): $C_{132}H_{253}N_{31}O_{22}$ calc./obs. 2625.96/2625.97 Da $[M+H]^+$.

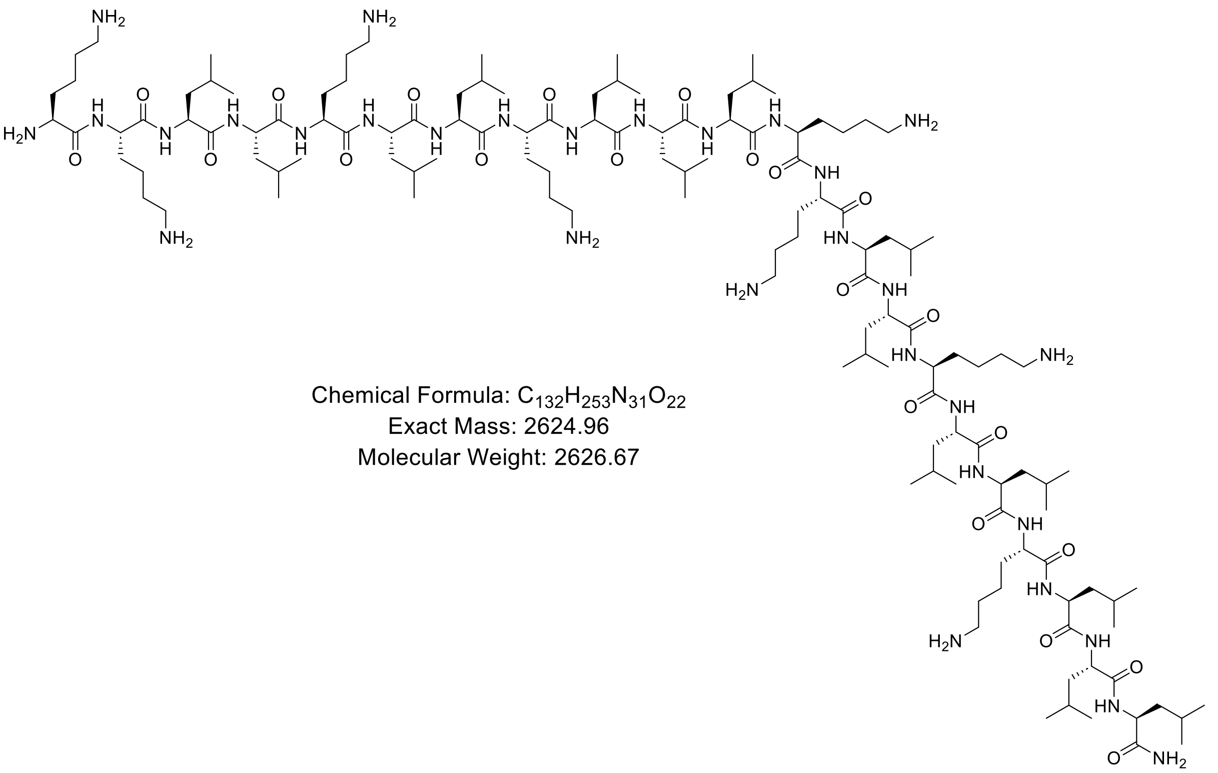

Analytical HPLC-MS data:

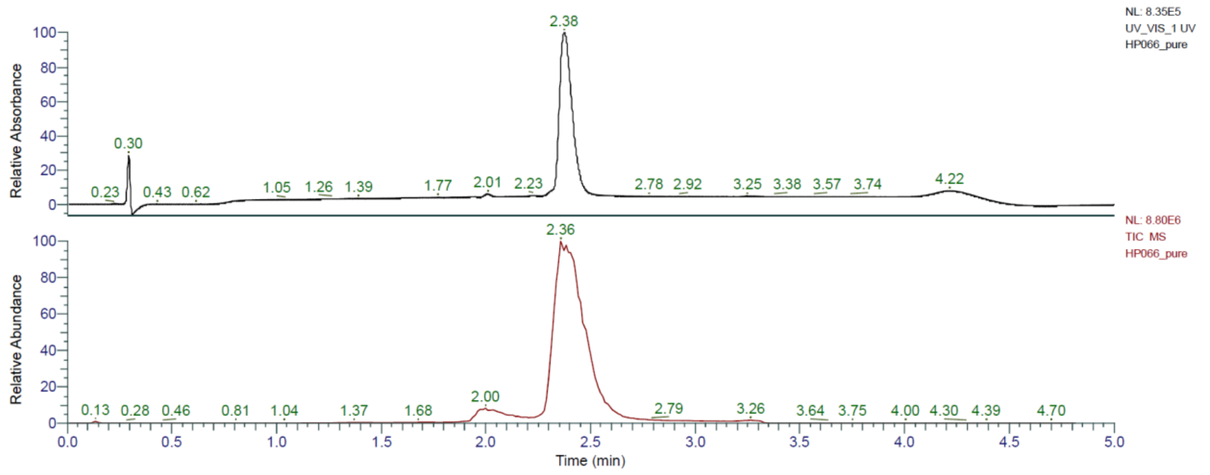

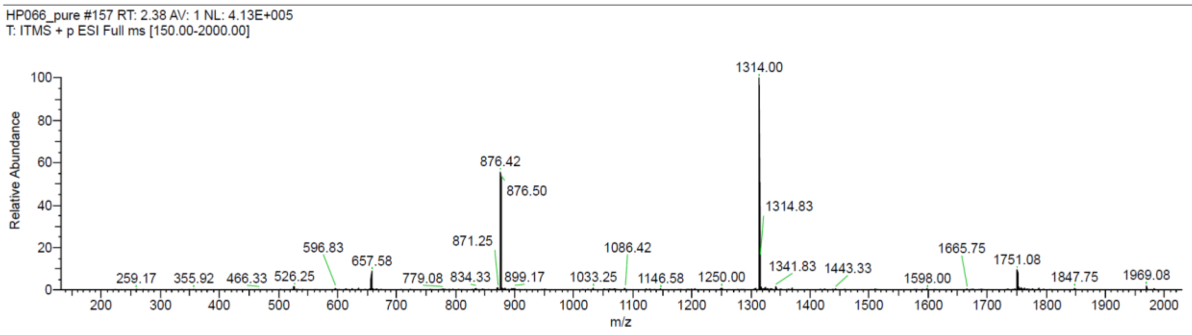

HRMS spectra:

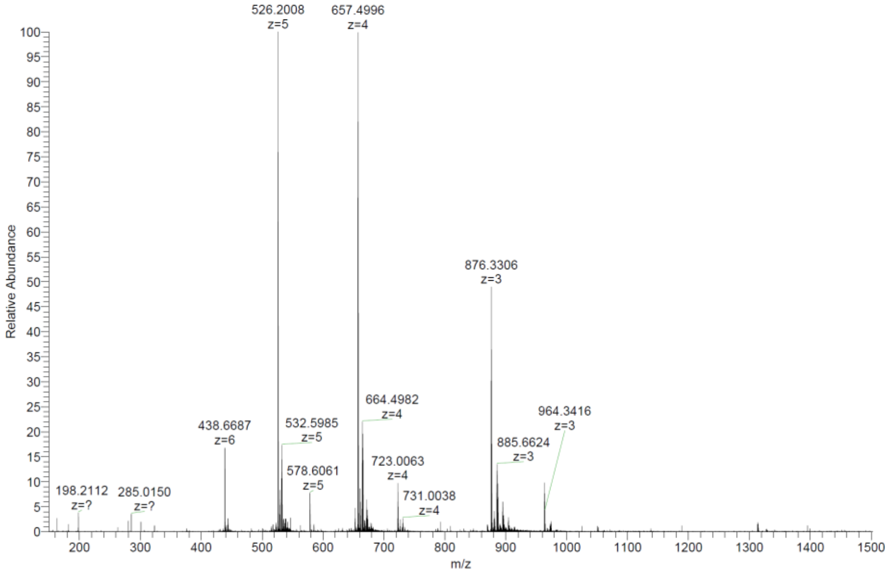

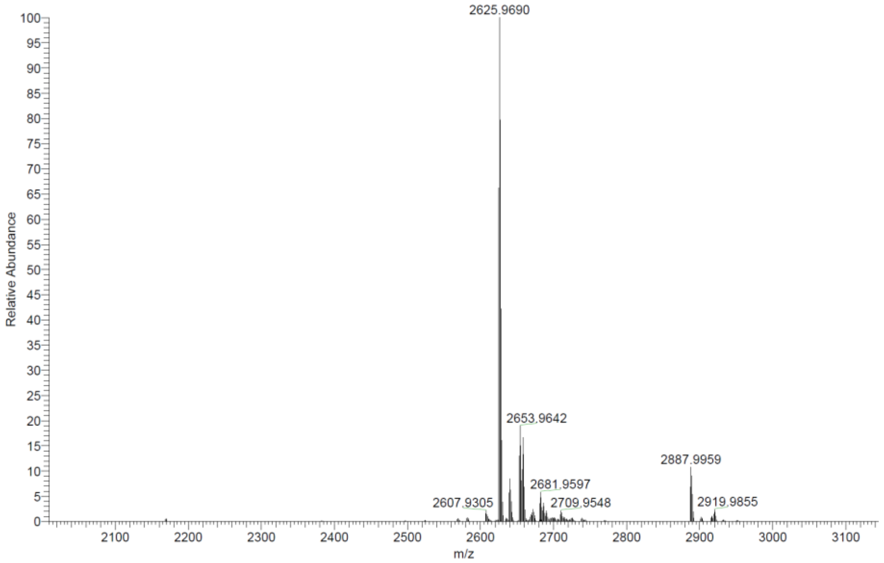

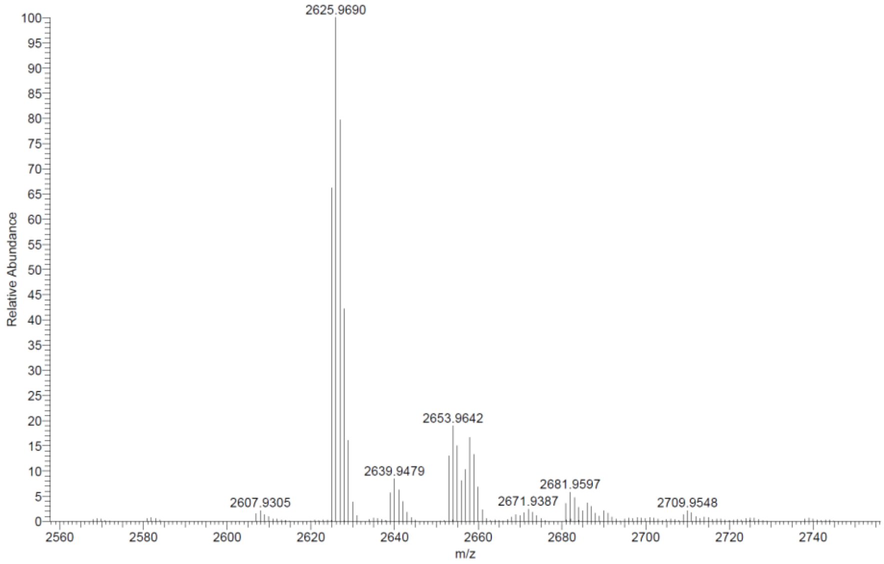

kkLLkLLkLLLkkLLkLLkLLL (2ln69) was obtained as white solid after preparative RP-HPLC (85.8 mg, 27.6%). Analytical RP-HPLC: t_R = 1.85 min (A/D 100:0 to 0:100 in 3.5 min, λ = 214 nm). MS (ESI+): $C_{132}H_{253}N_{31}O_{22}$ calc./obs. 2625.96/2625.97 Da $[M+H]^+$.

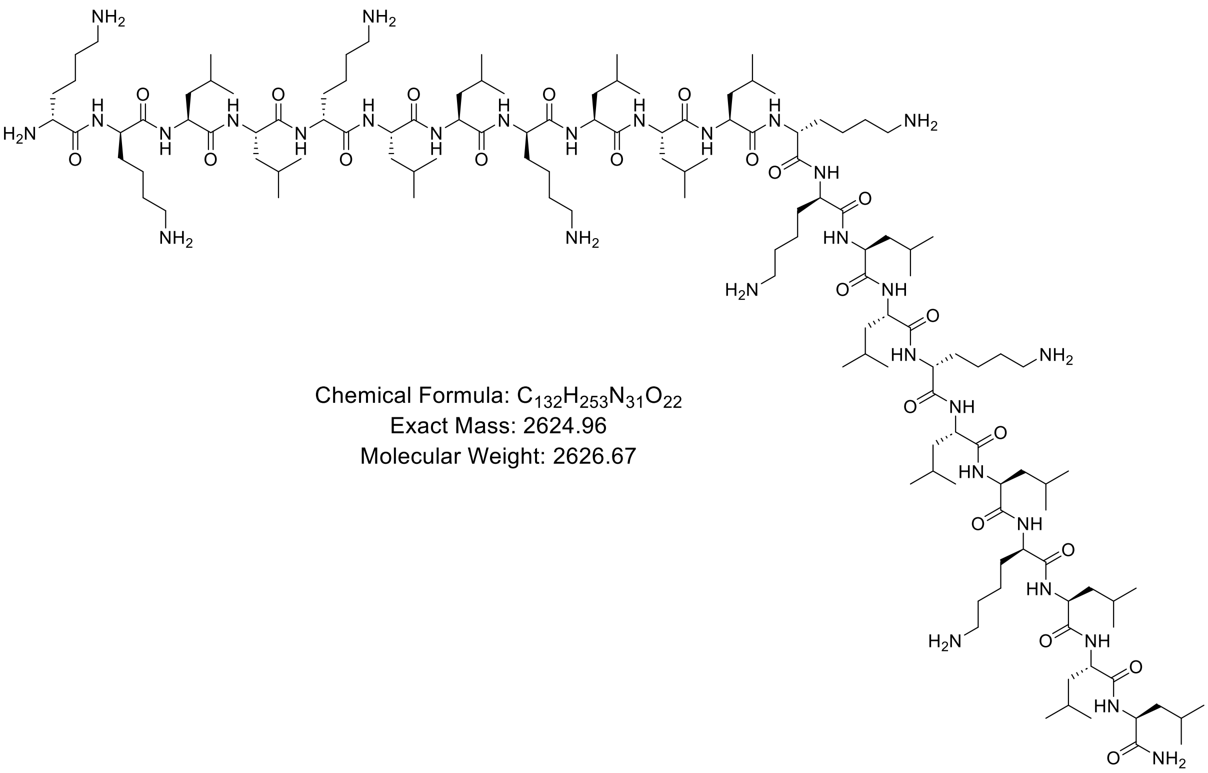

Analytical HPLC-MS data:

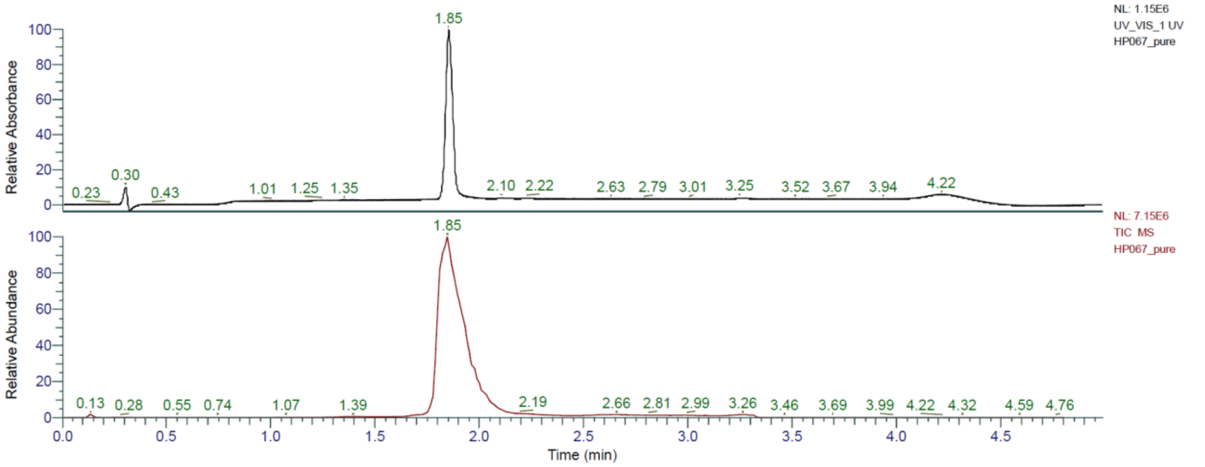

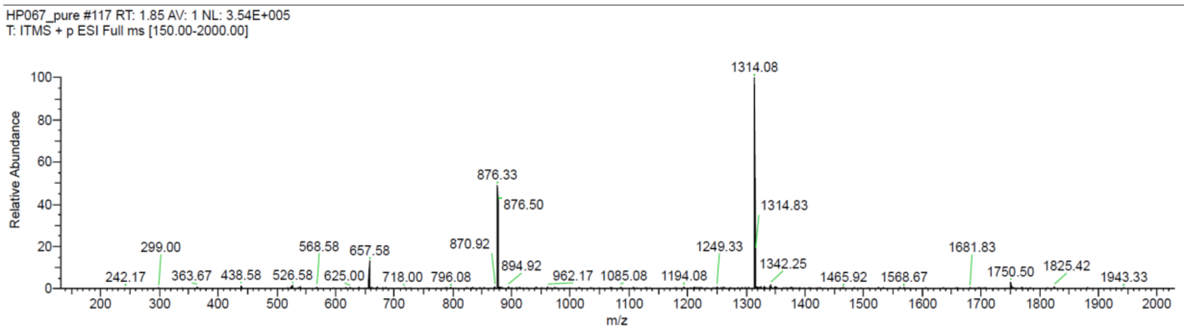

HRMS spectra:

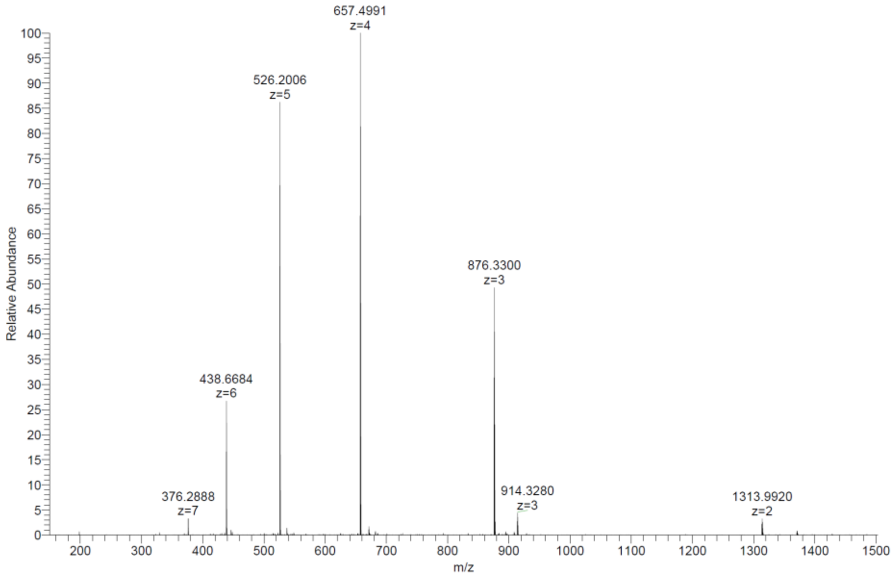

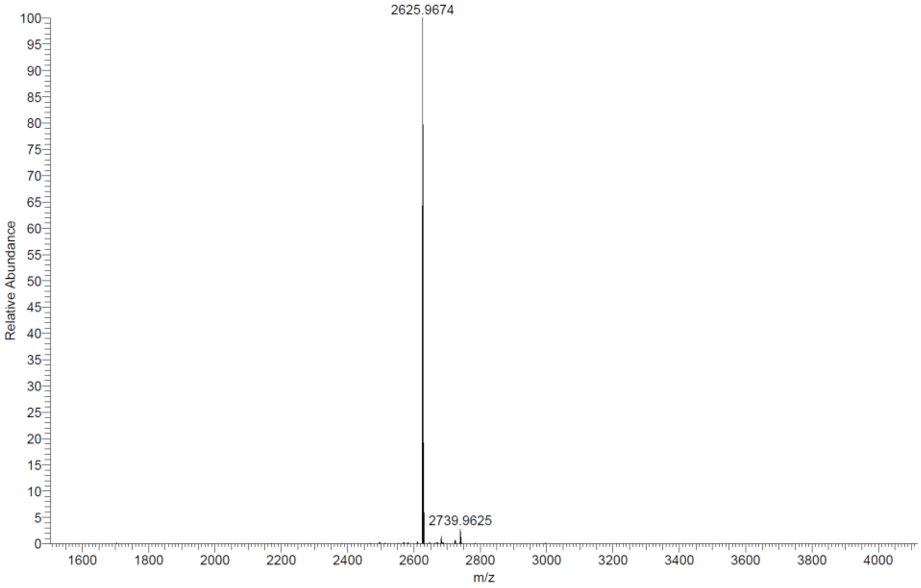

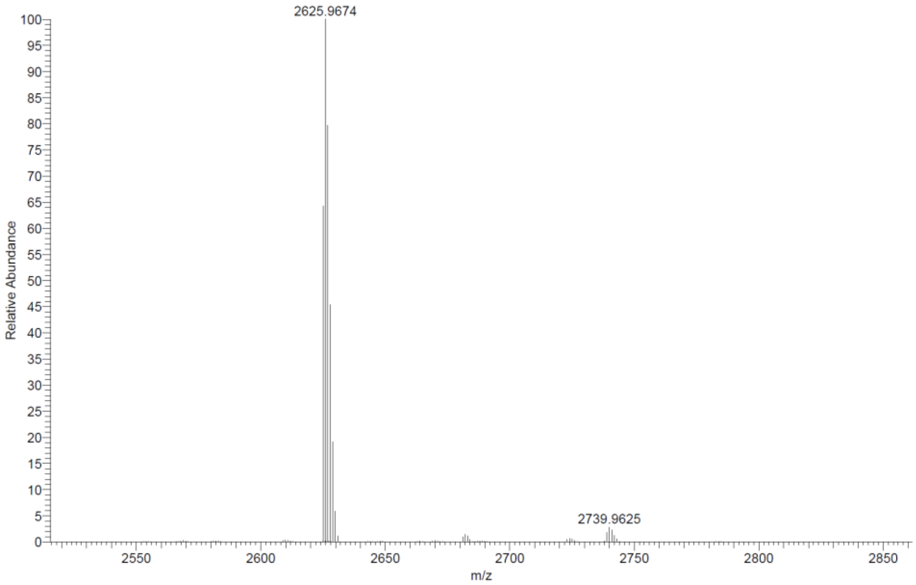

(*)KkLLKLLKLLL (FHP1) was obtained as white solid after preparative RP-HPLC (2.9 mg, 3.9%). Analytical RP-HPLC: t_R = 1.76 min (A/D 100:0 to 0:100 in 3.5 min, λ = 214 nm). MS (ESI+): $C_{66}H_{128}N_{16}O_{11}$ calc./obs. 1511.06/1511.07 Da $[M+2H]^{2+}$.

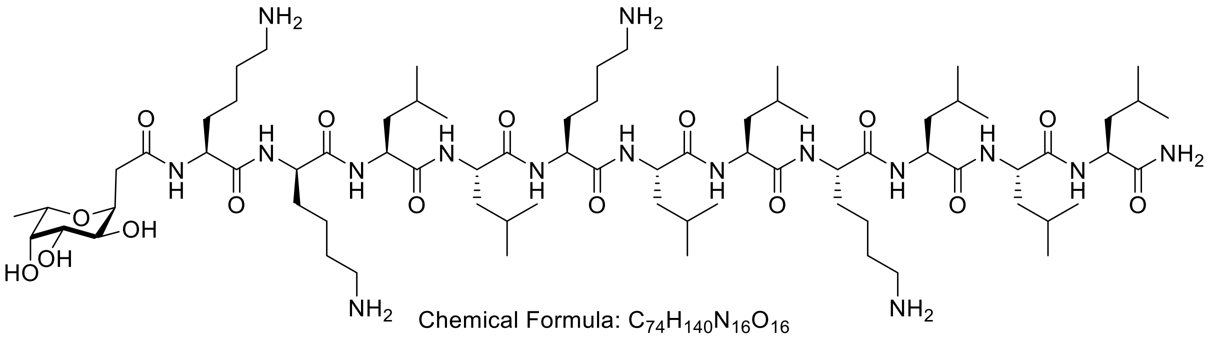

Chemical Formula: $C_{74}H_{140}N_{16}O_{16}$
Exact Mass: 1509.06
Molecular Weight: 1510.03

Analytical HPLC-MS data:

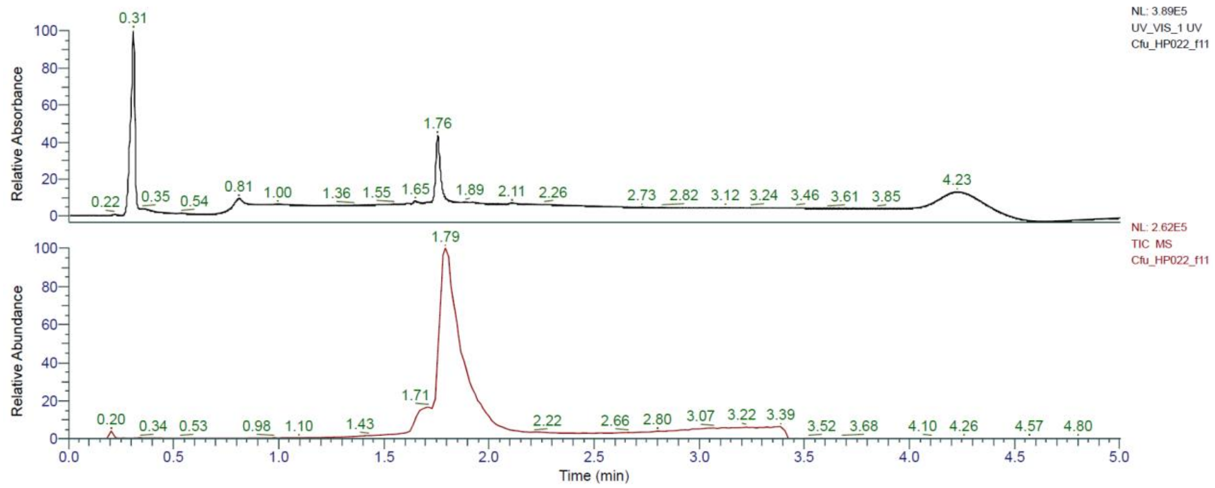

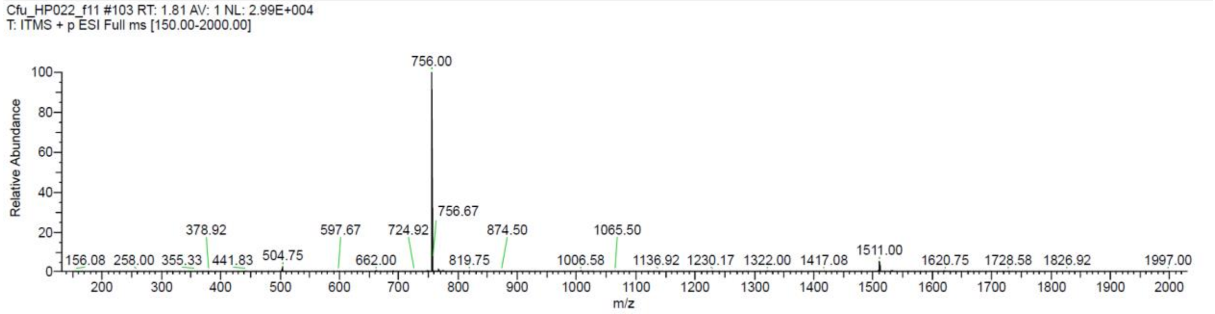

HRMS spectra:

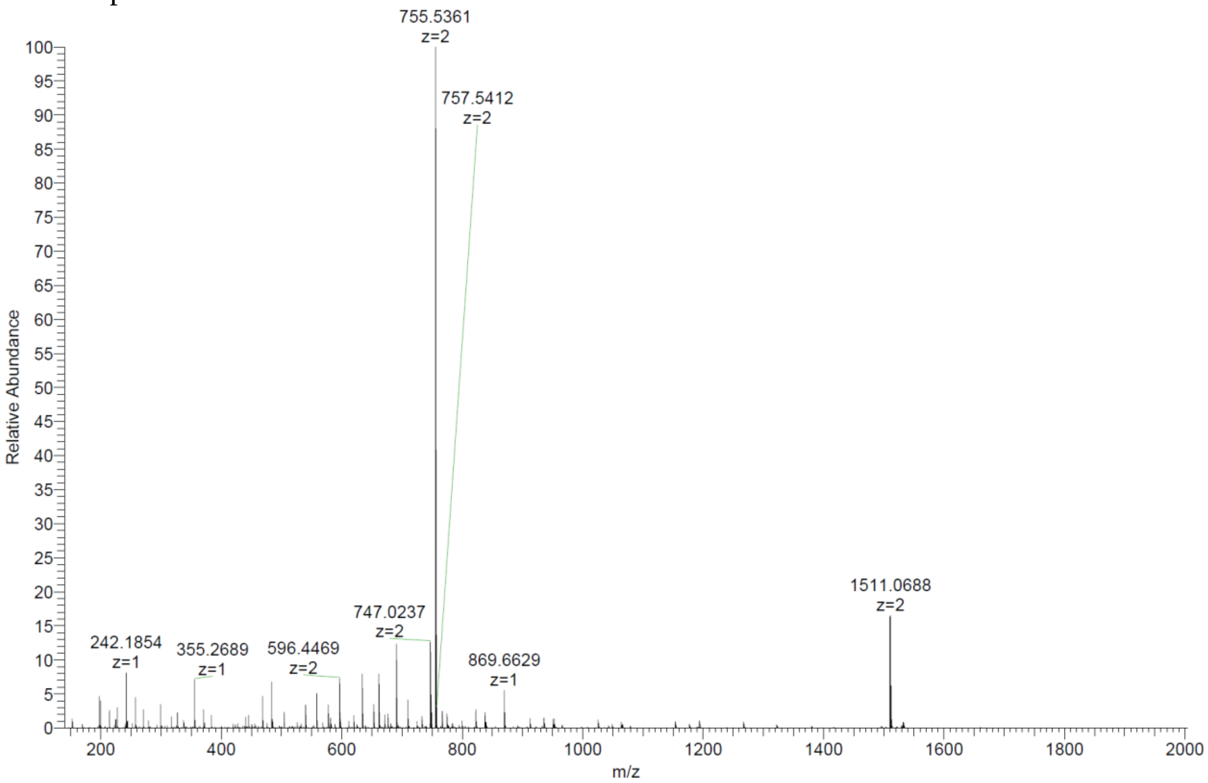

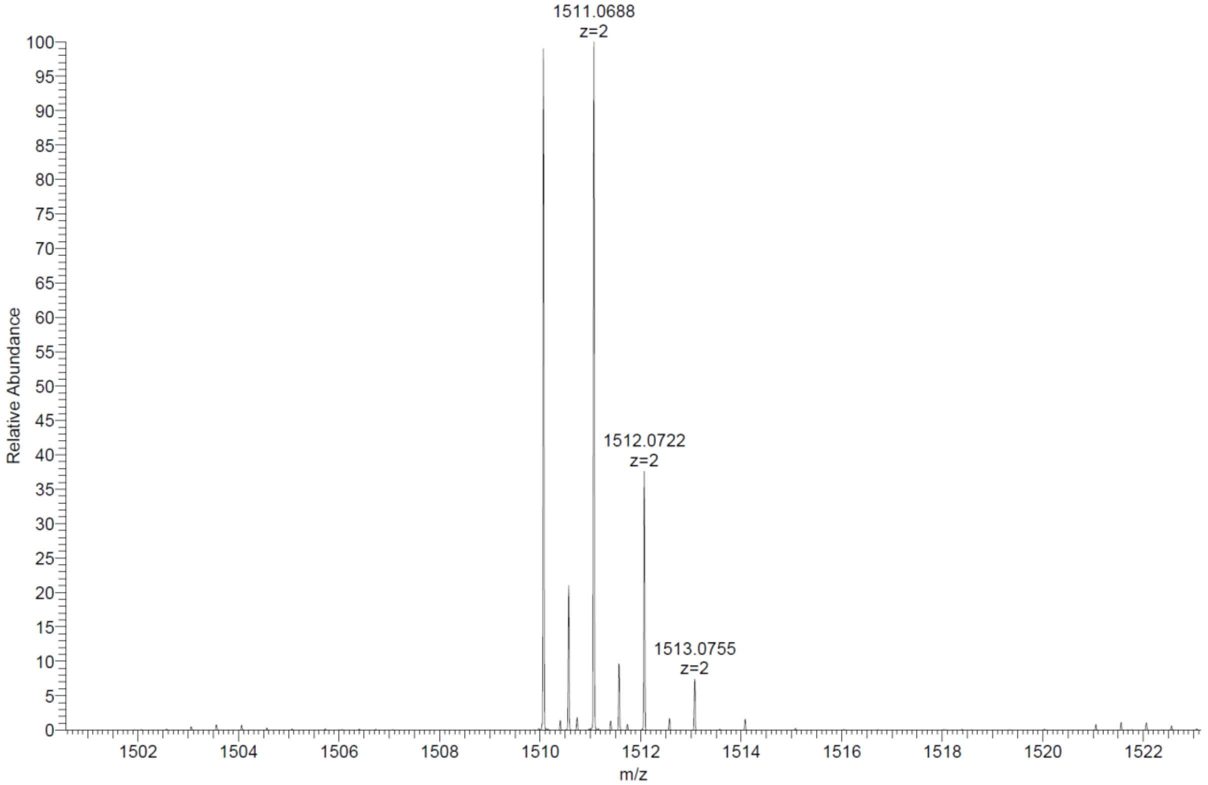

(*)kkLLKLLKLLL (FHP2) was obtained as white solid after preparative RP-HPLC (0.6 mg, 0.8%). Analytical RP-HPLC: t_R = 1.73 min (A/D 100:0 to 0:100 in 3.5 min, λ = 214 nm). MS (ESI+): $C_{66}H_{128}N_{16}O_{11}$ calc./obs. 1510.06/1510.07 Da $[M+H]^+$.

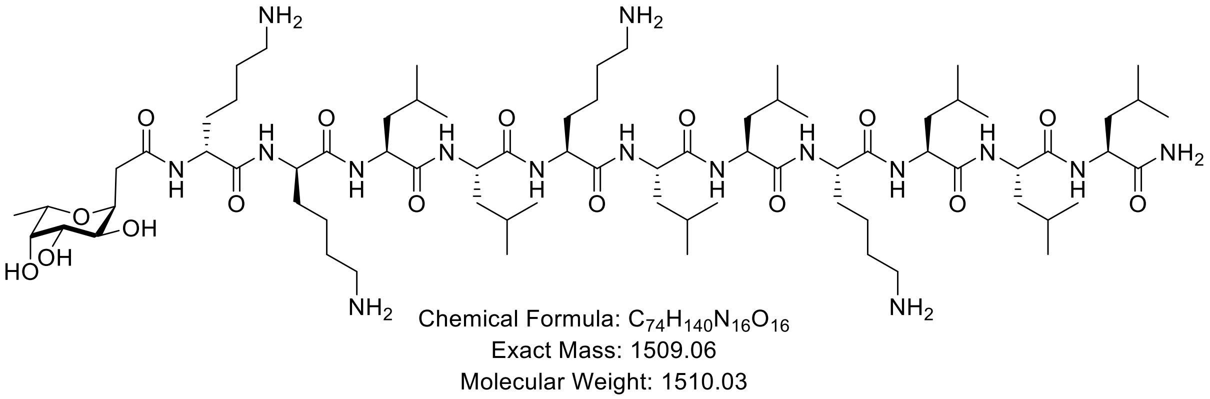

Analytical LC-MS data:

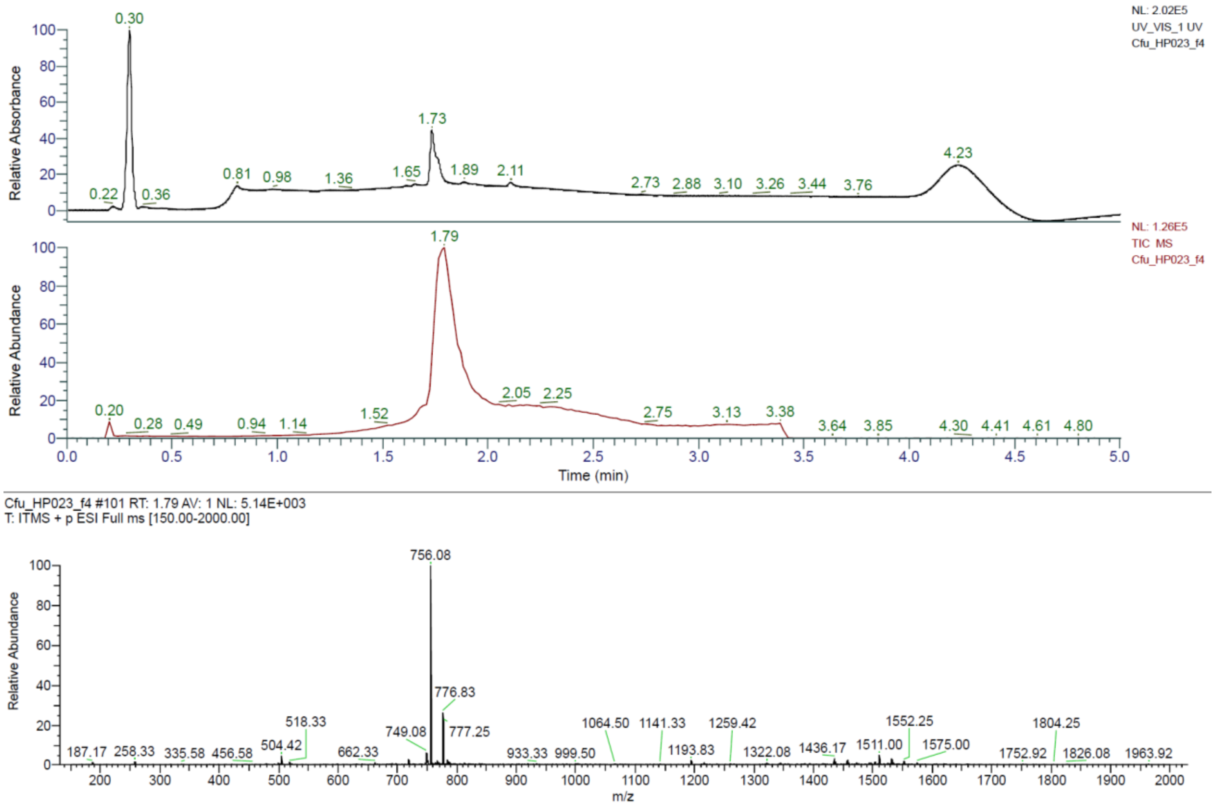

HRMS spectra:

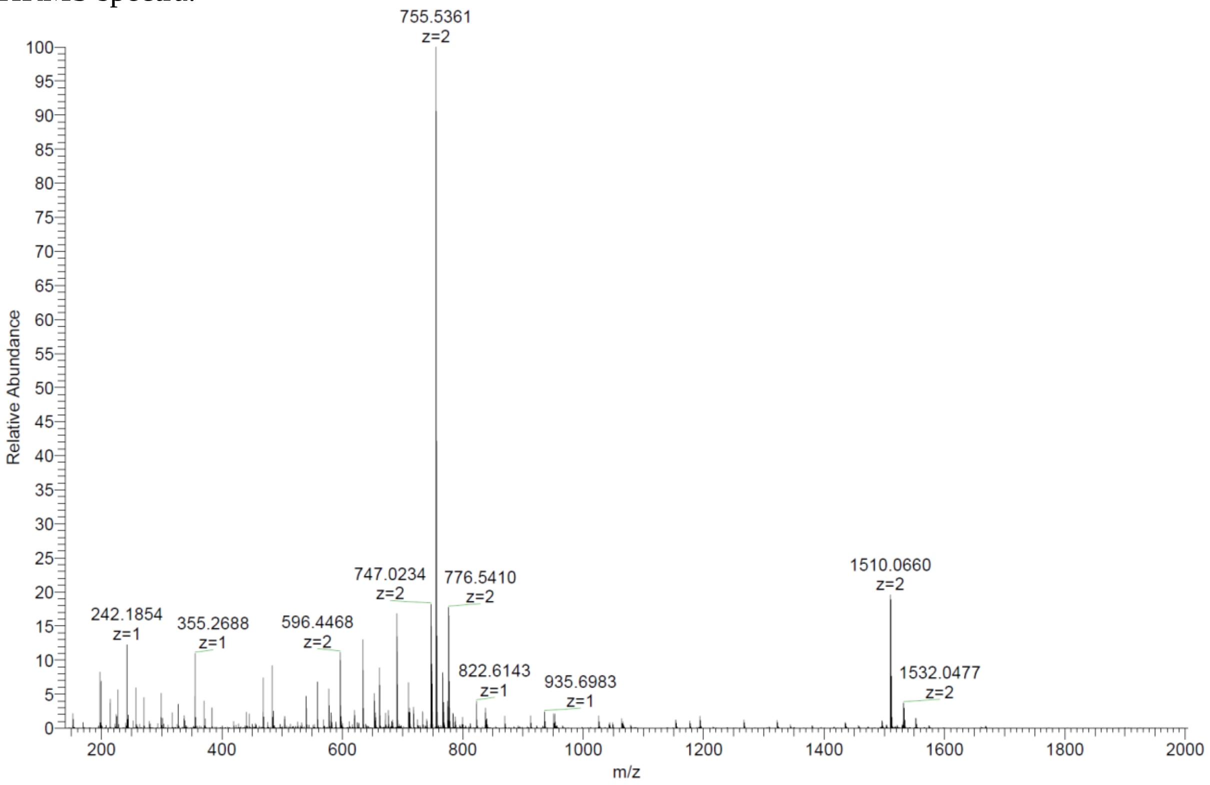

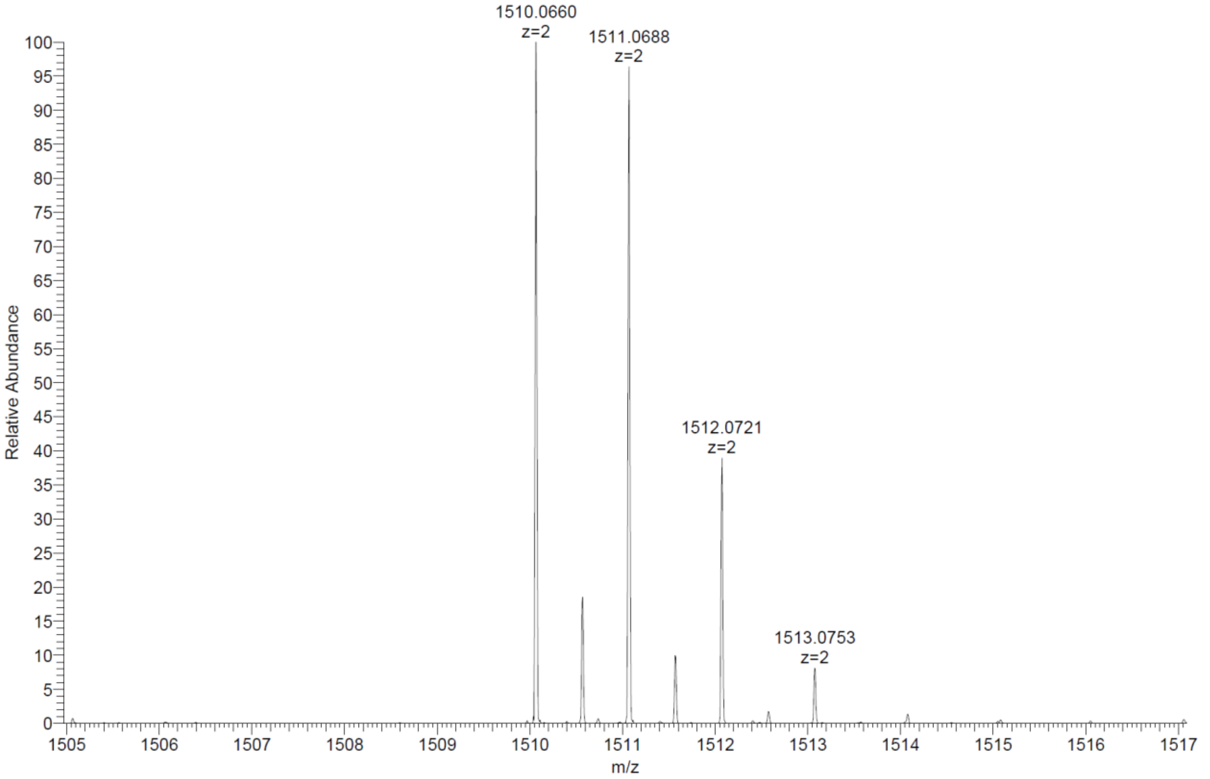

(*)KkLLkLLKLLL (FHP3) was obtained as white solid after preparative RP-HPLC (5.0 mg, 6.8%). Analytical RP-HPLC: t_R = 1.67 min (A/D 100:0 to 0:100 in 3.5 min, λ = 214 nm). MS (ESI+): $C_{66}H_{128}N_{16}O_{11}$ calc./obs. 1510.06/1510.07 Da $[M+H]^+$.

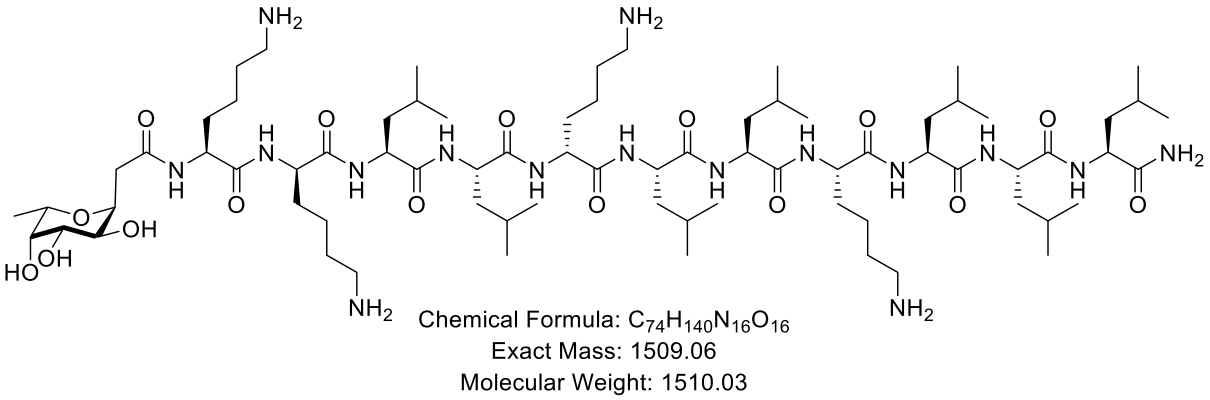

Analytical LC-MS data:

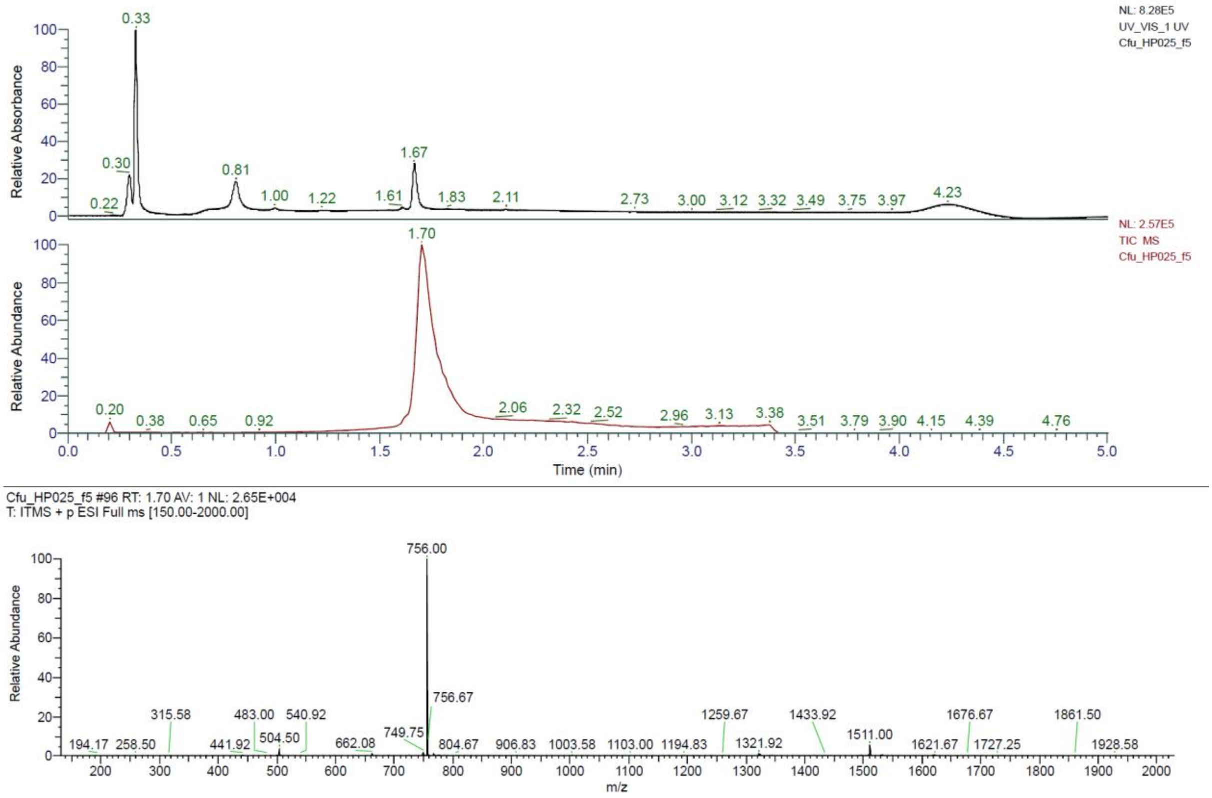

HRMS spectra:

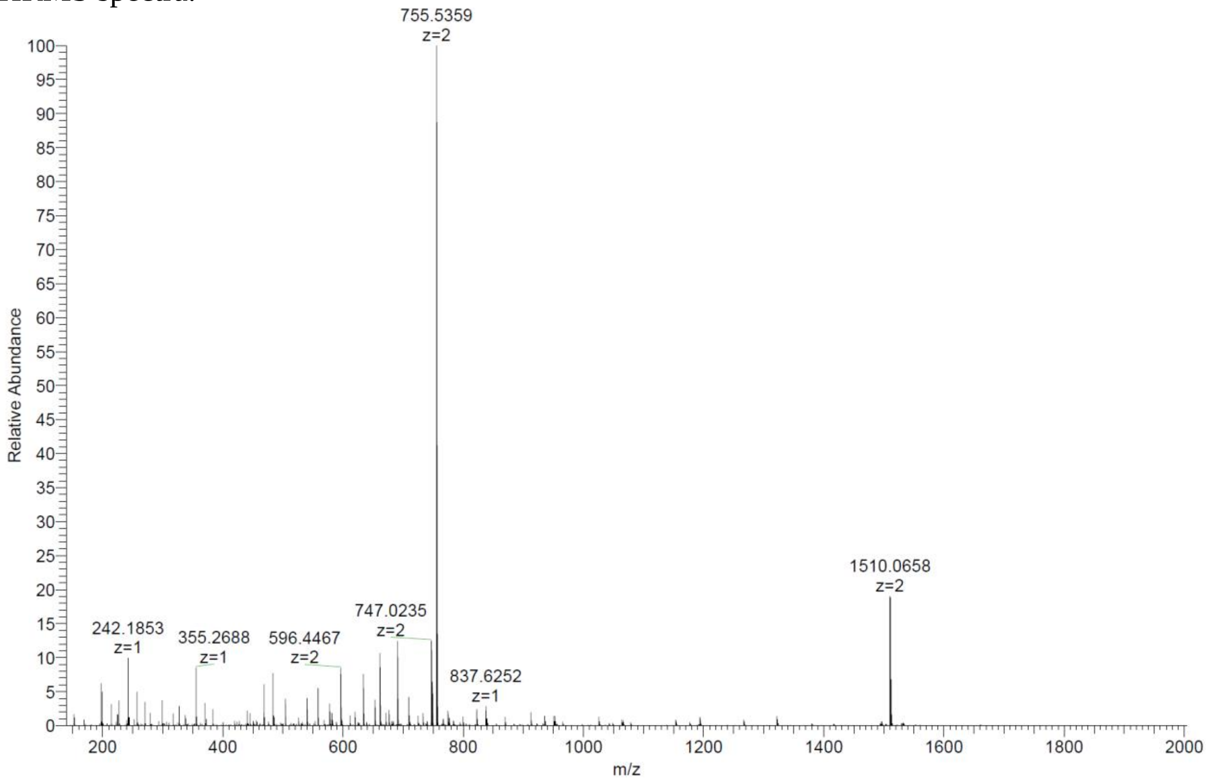

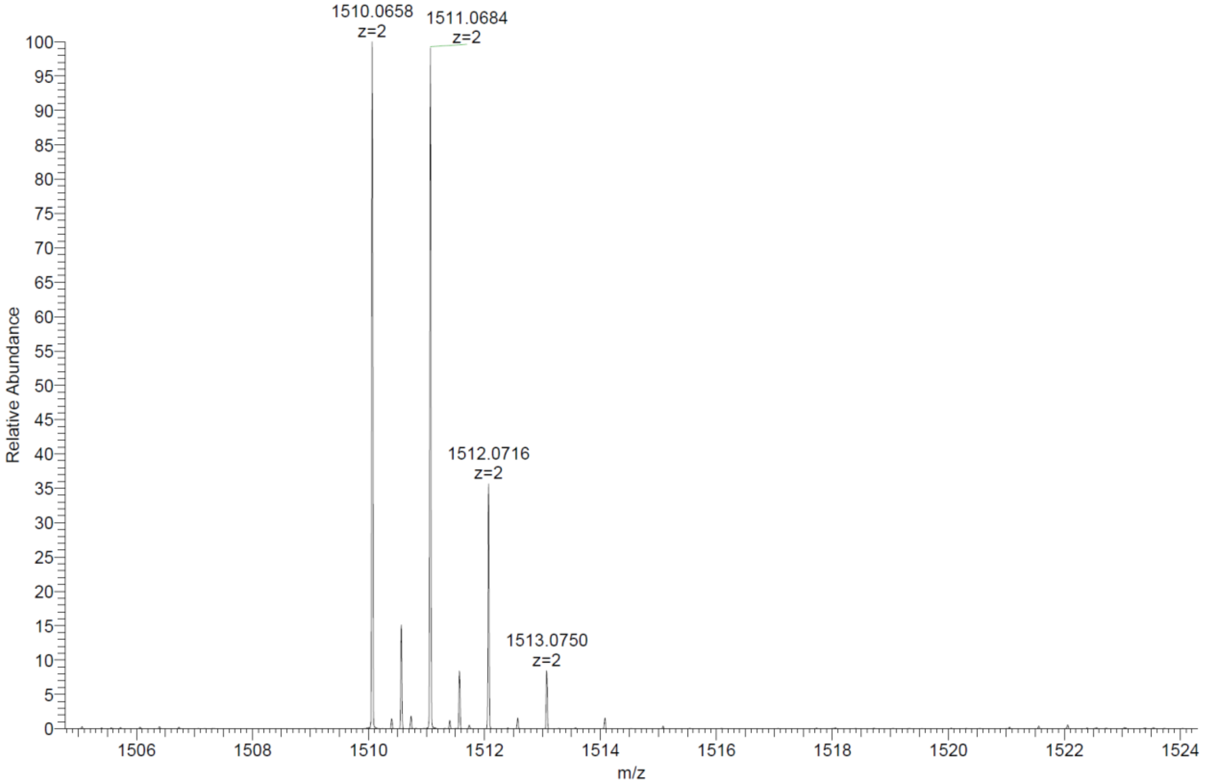

(*)KkLlKLLKLLL (FHP4) was obtained as white solid after preparative RP-HPLC (4.5 mg, 6.1%). Analytical RP-HPLC: t_R = 1.74 min (A/D 100:0 to 0:100 in 3.5 min, λ = 214 nm). MS (ESI+): $C_{66}H_{128}N_{16}O_{11}$ calc./obs. 1510.06/1510.07 Da $[M+H]^+$.

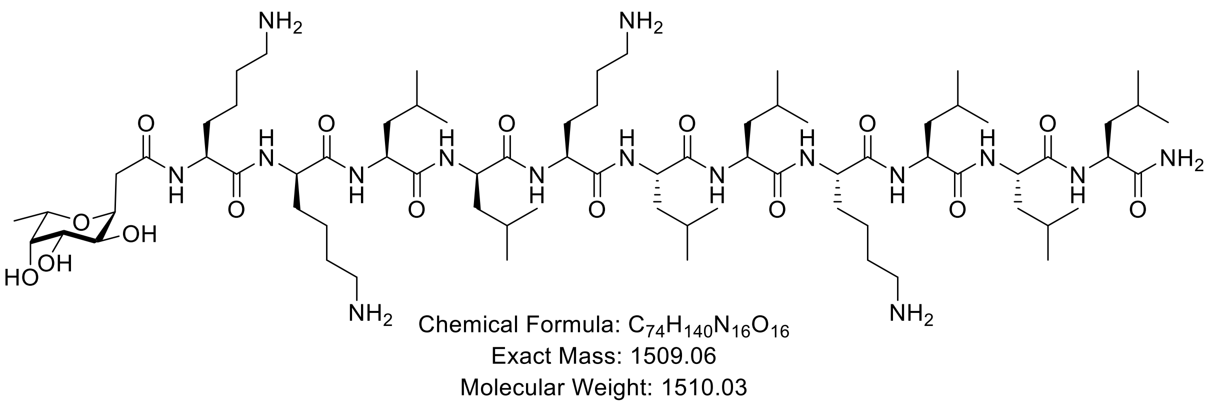

Analytical LC-MS data:

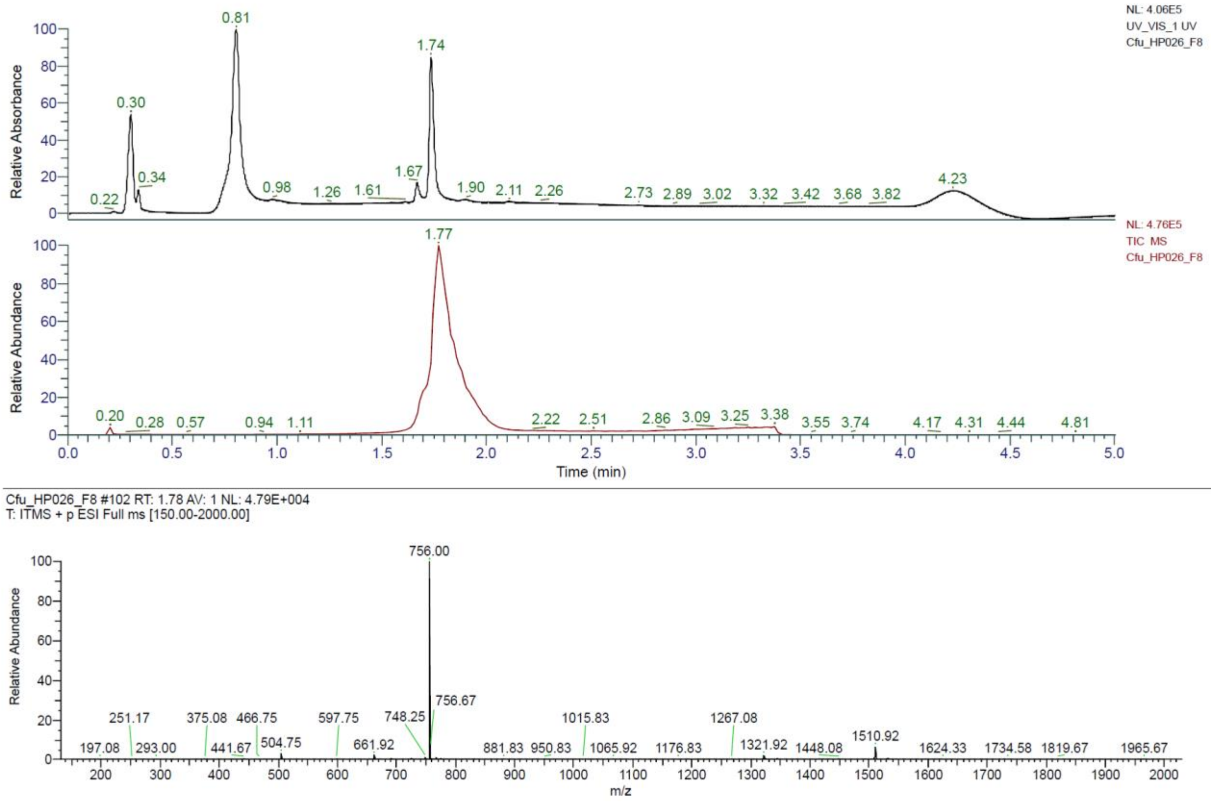

HRMS spectra:

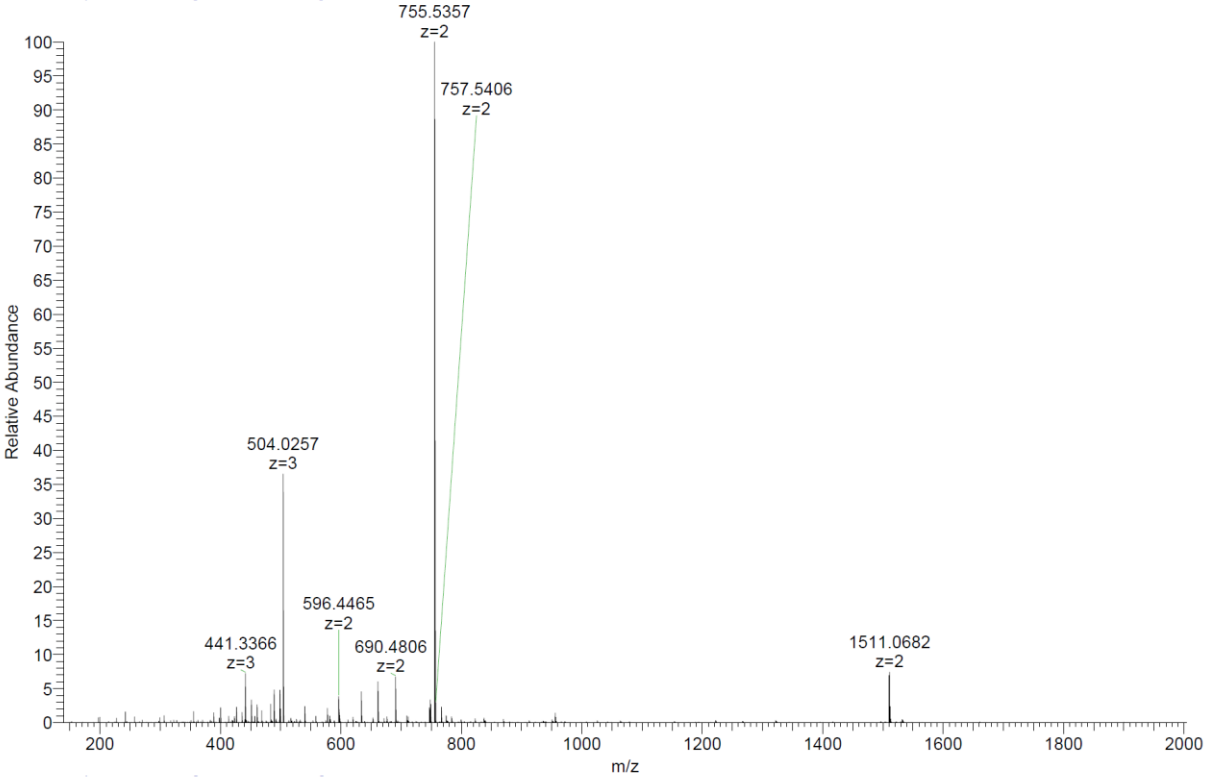

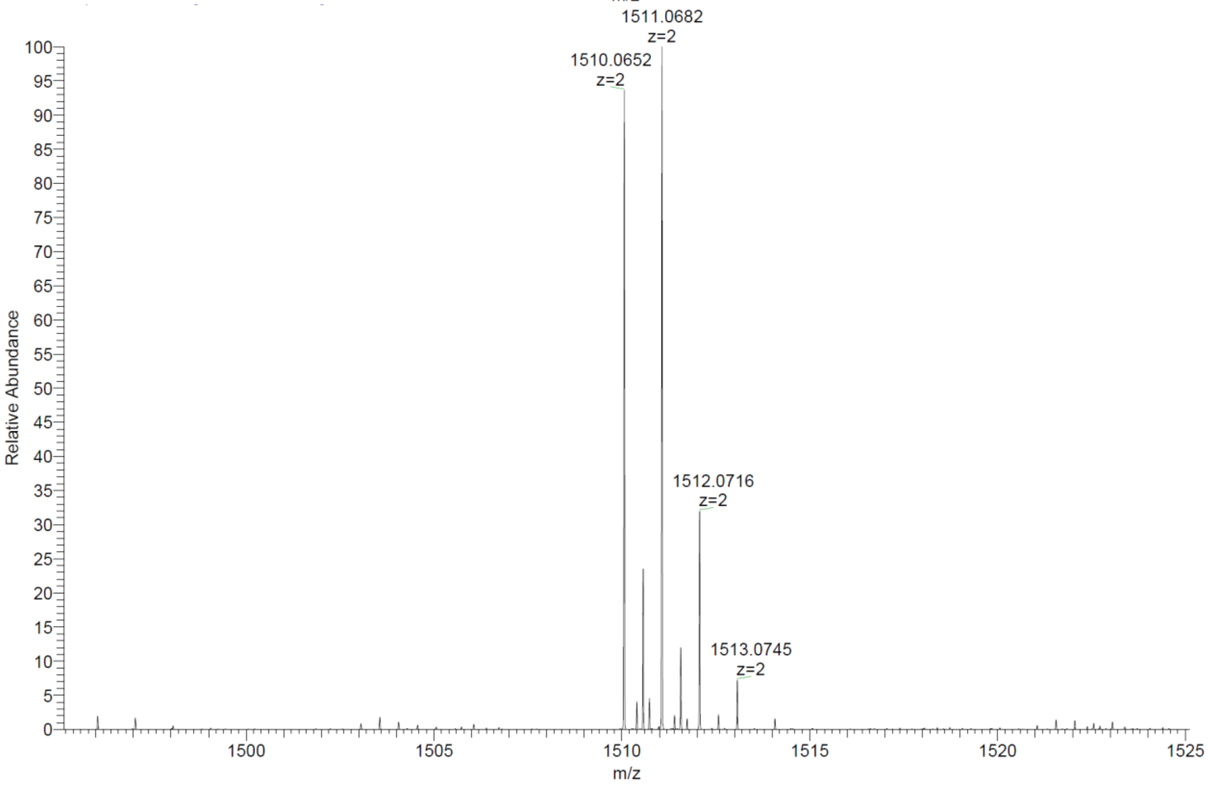

(*)kKLLKLLKLLl (FHP5) was obtained as white solid after preparative RP-HPLC (4.3 mg, 5.8%). Analytical RP-HPLC: t_R = 1.66 min (A/D 100:0 to 0:100 in 3.5 min, λ = 214 nm). MS (ESI+): $C_{66}H_{128}N_{16}O_{11}$ calc./obs. 1510.06/1510.07 Da $[M+H]^+$.

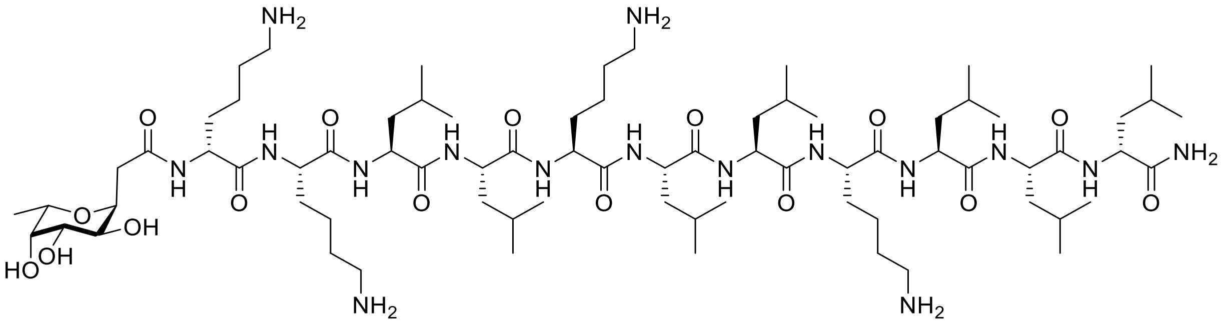

Chemical Formula: $C_{74}H_{140}N_{16}O_{16}$
Exact Mass: 1509.06
Molecular Weight: 1510.03

Analytical HPLC-MS data:

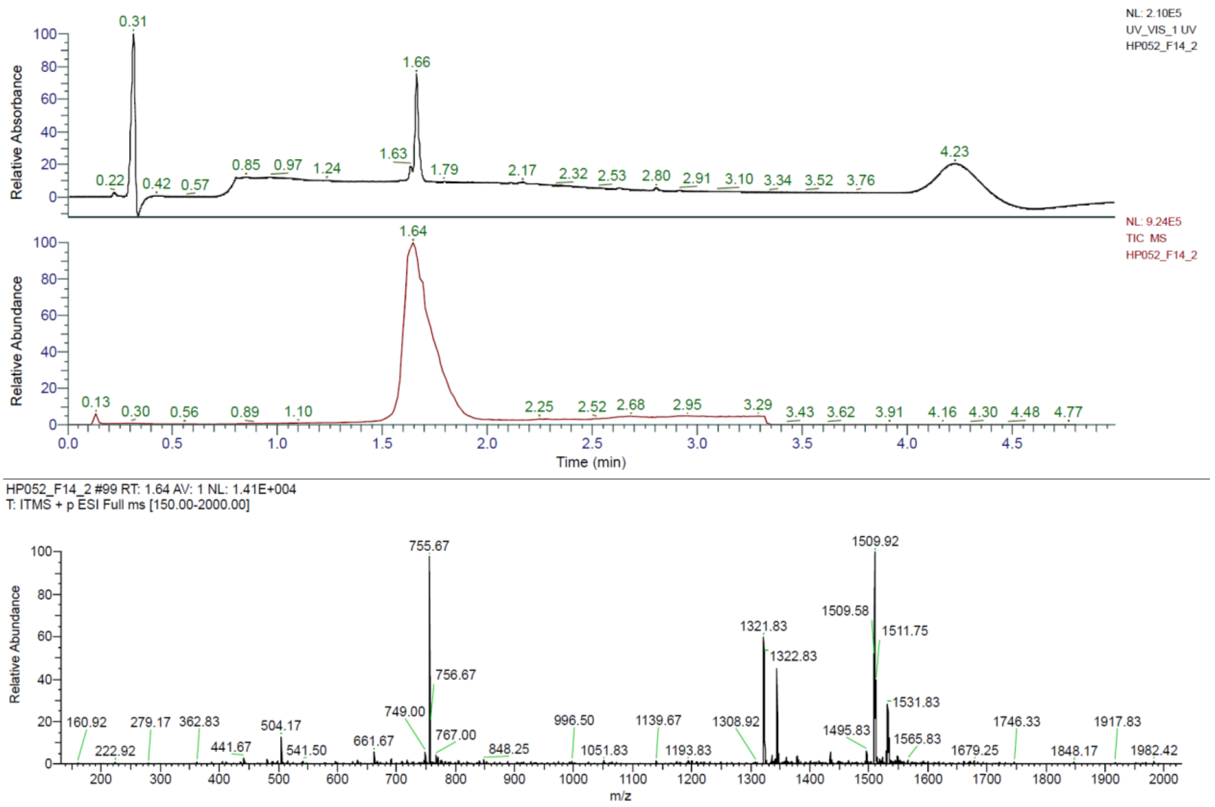

HRMS spectra:

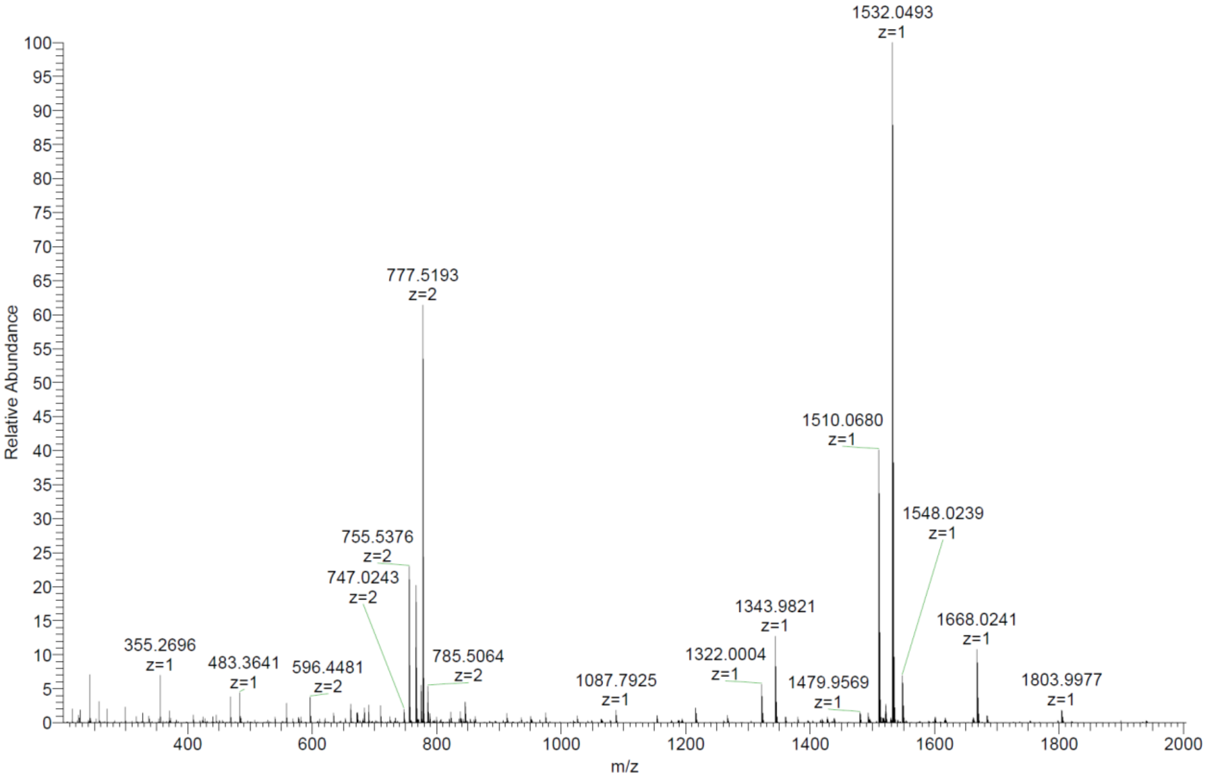

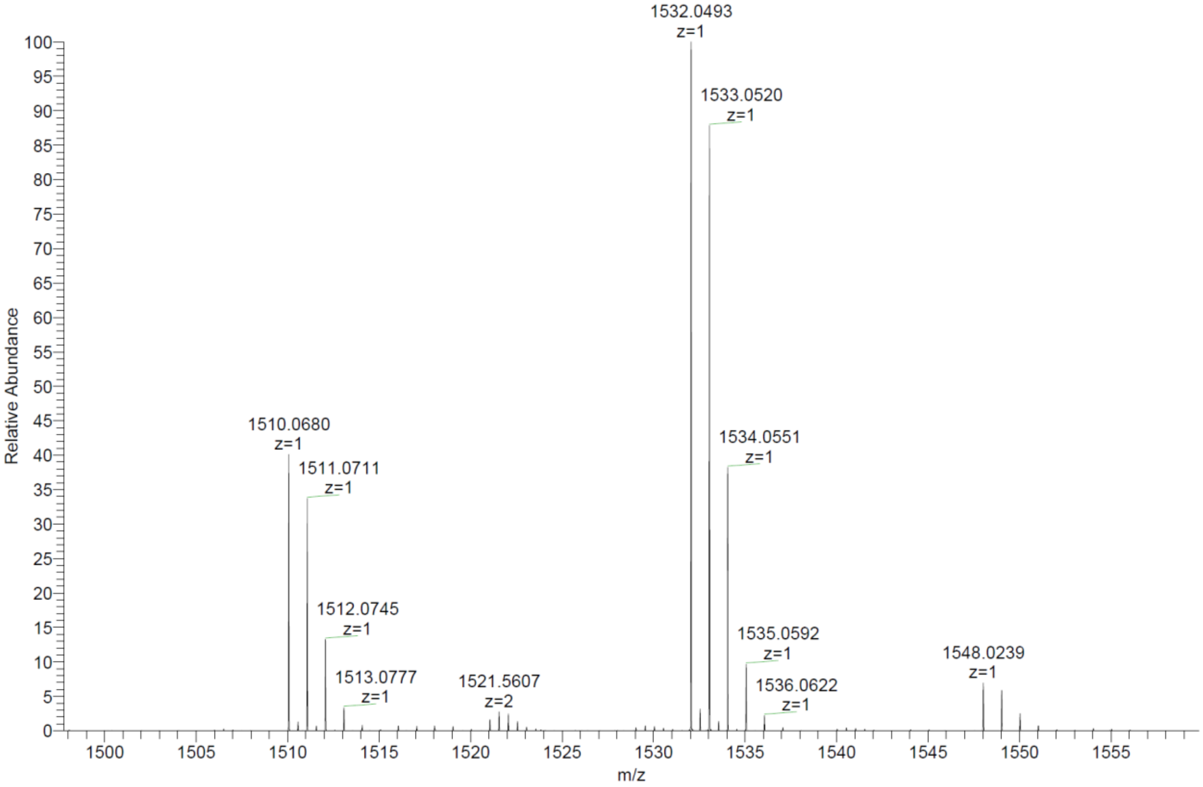

(*)kkLLKLLKLLl (FHP7) was obtained as white solid after preparative RP-HPLC (3.4 mg, 4.6%). Analytical RP-HPLC: t_R = 1.63 min (A/D 100:0 to 0:100 in 3.5 min, λ = 214 nm). MS (ESI+): $C_{74}H_{140}N_{16}O_{16}$ calc./obs. 1510.06/1510.07 Da $[M+H]^+$.

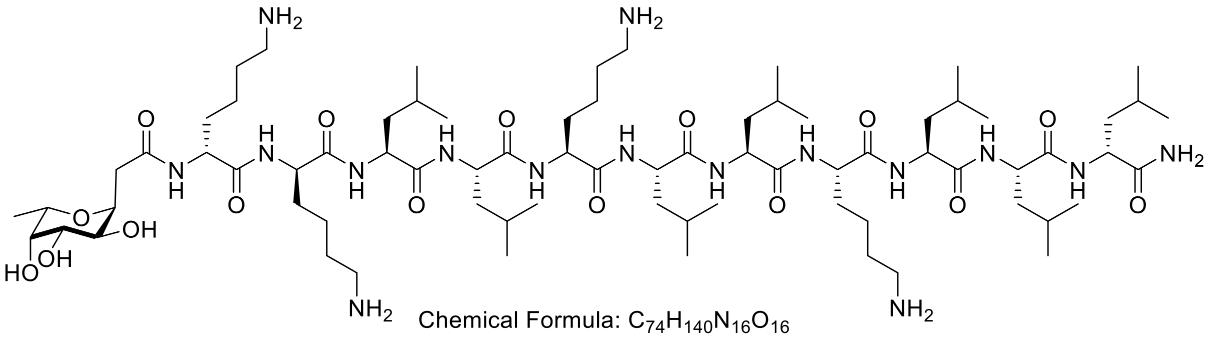

Chemical Formula: $C_{74}H_{140}N_{16}O_{16}$
Exact Mass: 1509.06
Molecular Weight: 1510.03

Analytical LC-MS data:

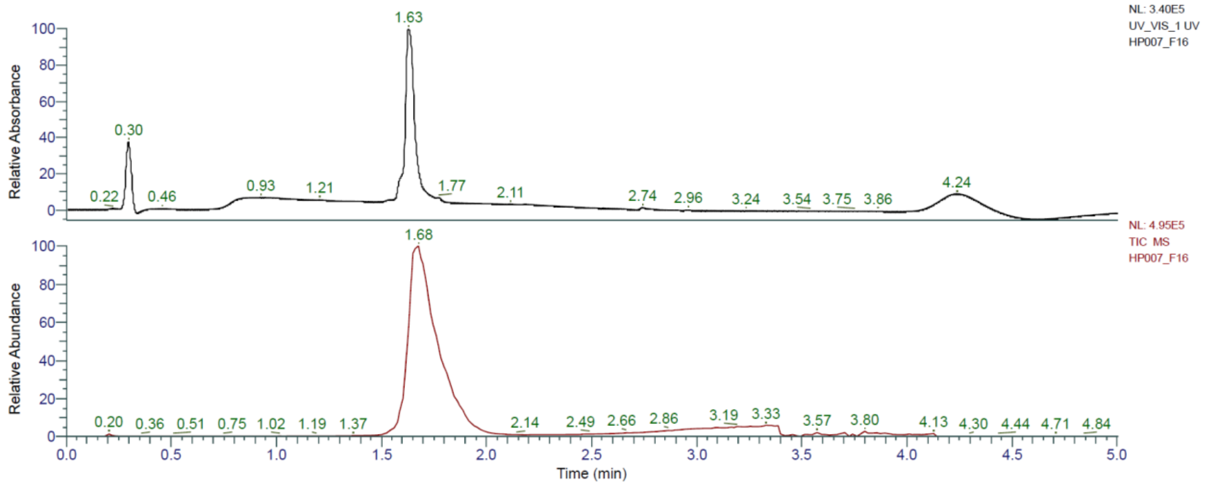

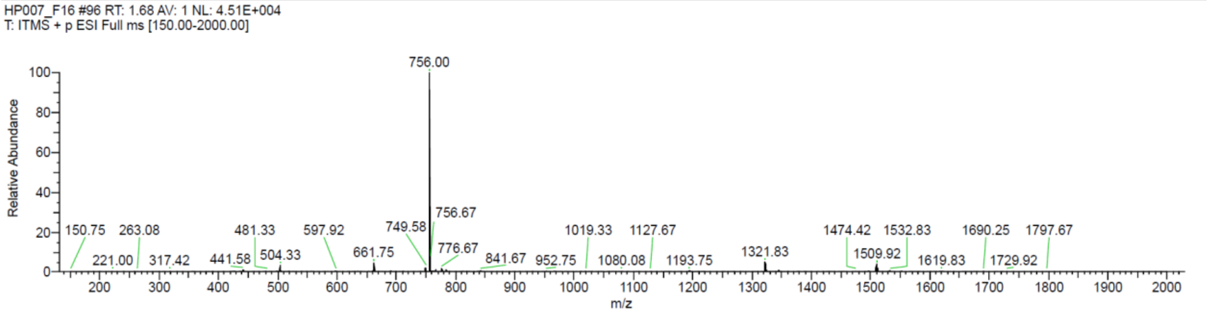

HRMS spectra:

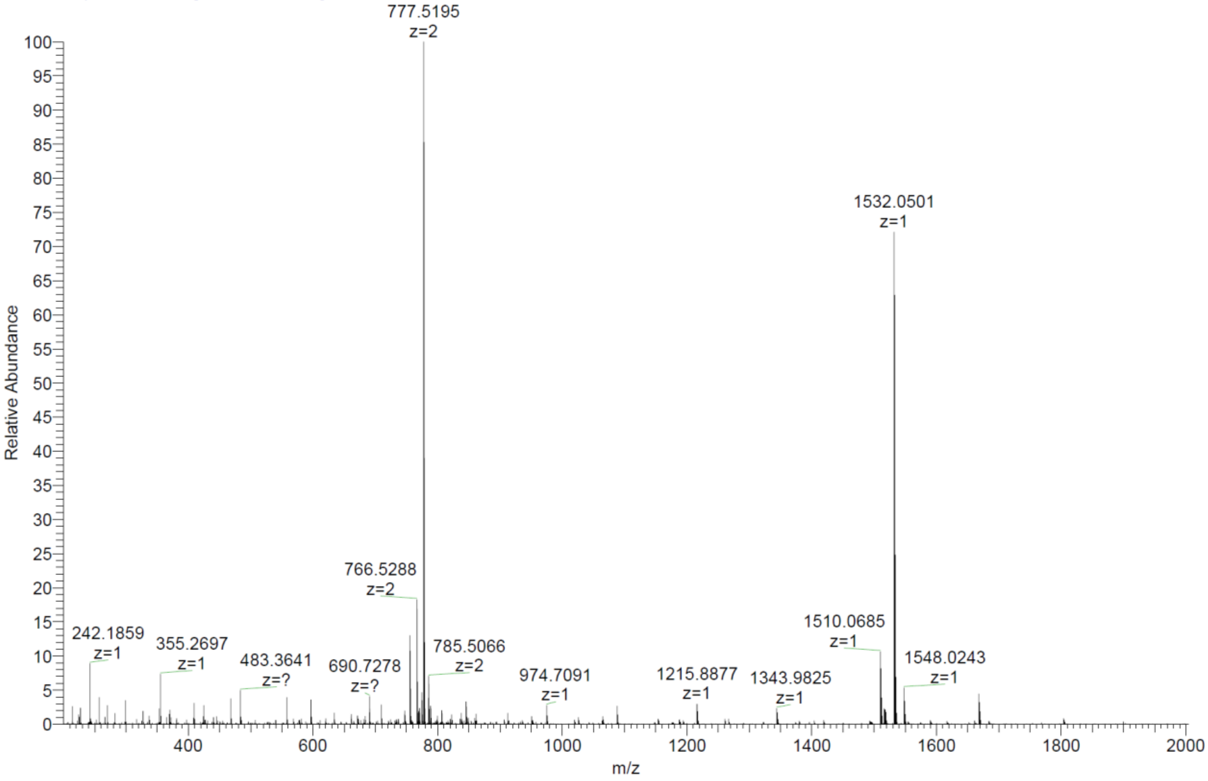

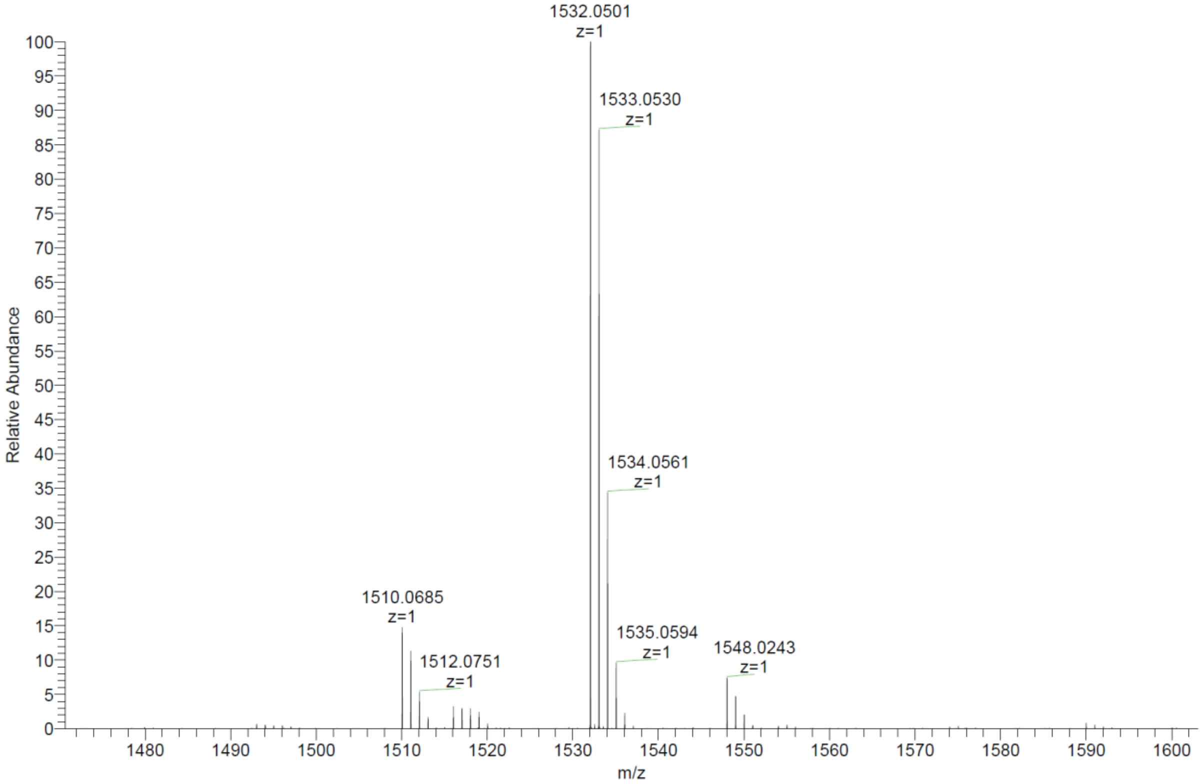

(*)KkllKLLKLLL (FHP8) was obtained as white solid after preparative RP-HPLC (5.7 mg, 7.7%). Analytical RP-HPLC: t_R = 1.63 min (A/D 100:0 to 0:100 in 3.5 min, λ = 214 nm). MS (ESI+): $C_{74}H_{140}N_{16}O_{16}$ calc./obs. 1510.06/1510.06 Da $[M+H]^+$.

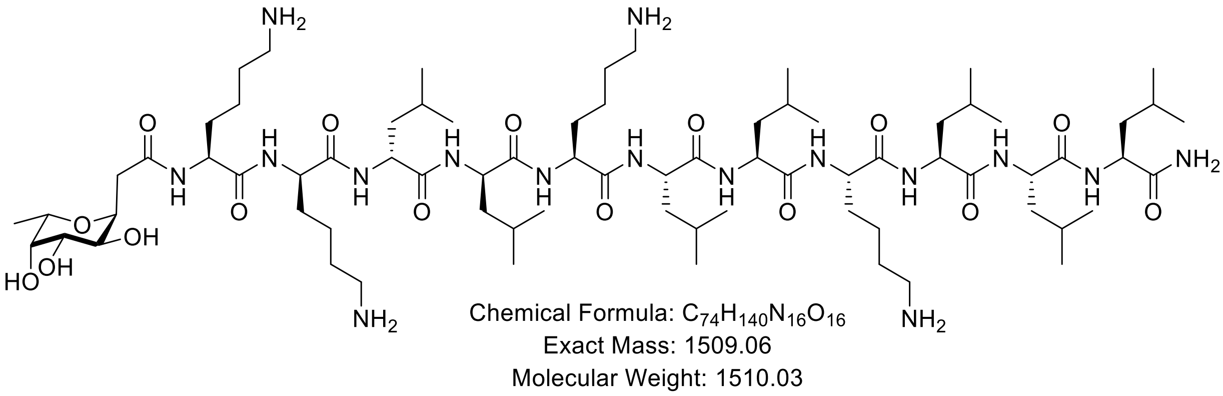

Analytical HPLC-MS data:

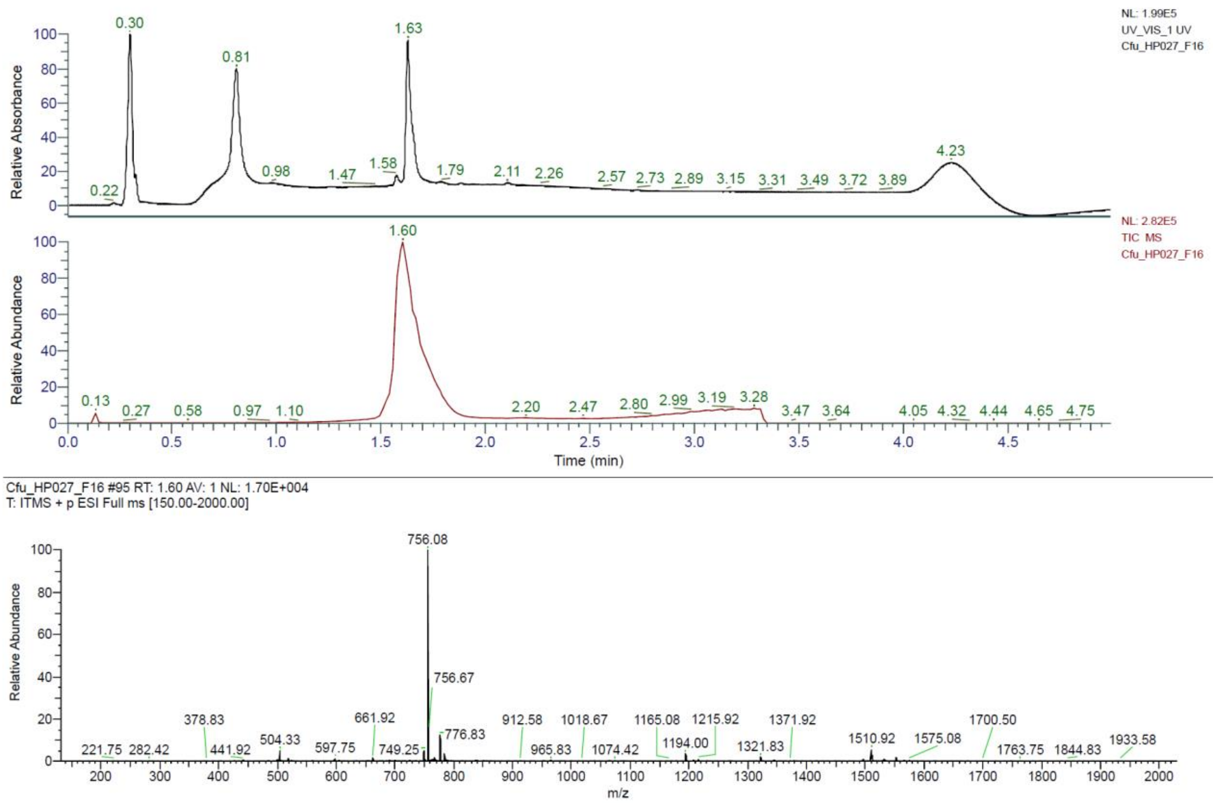

HRMS spectra:

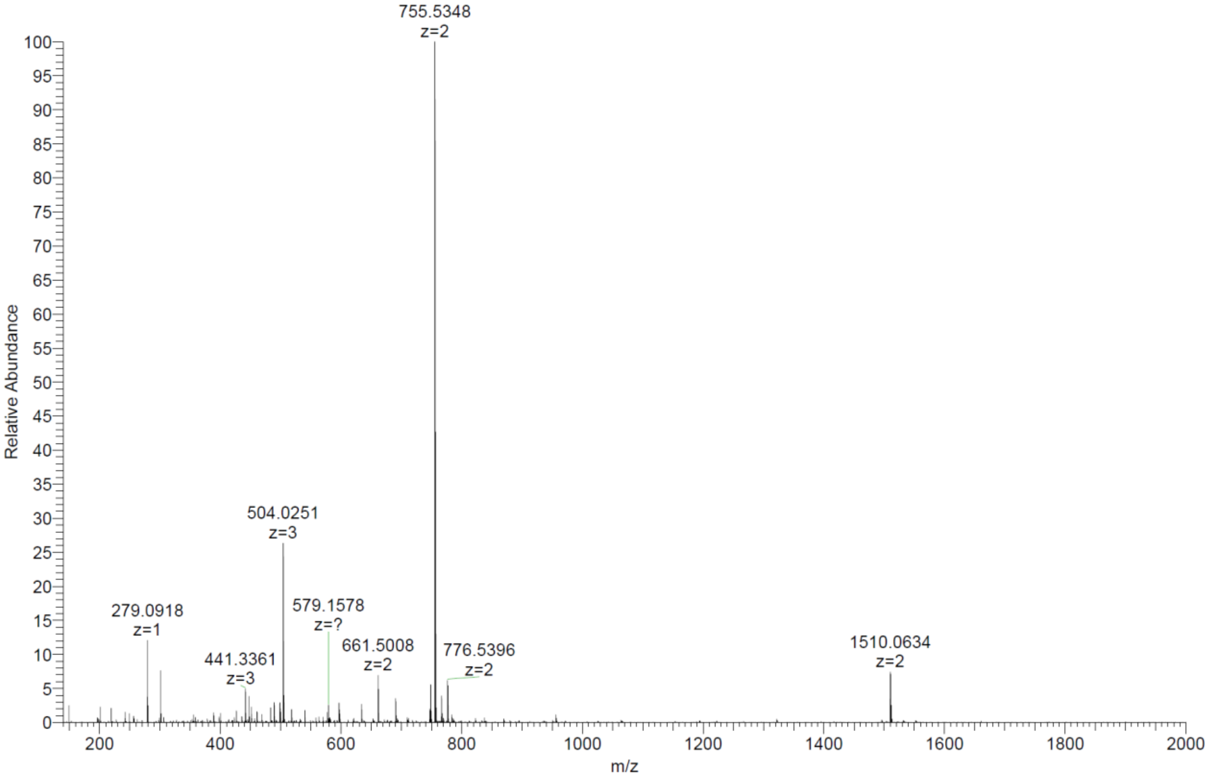

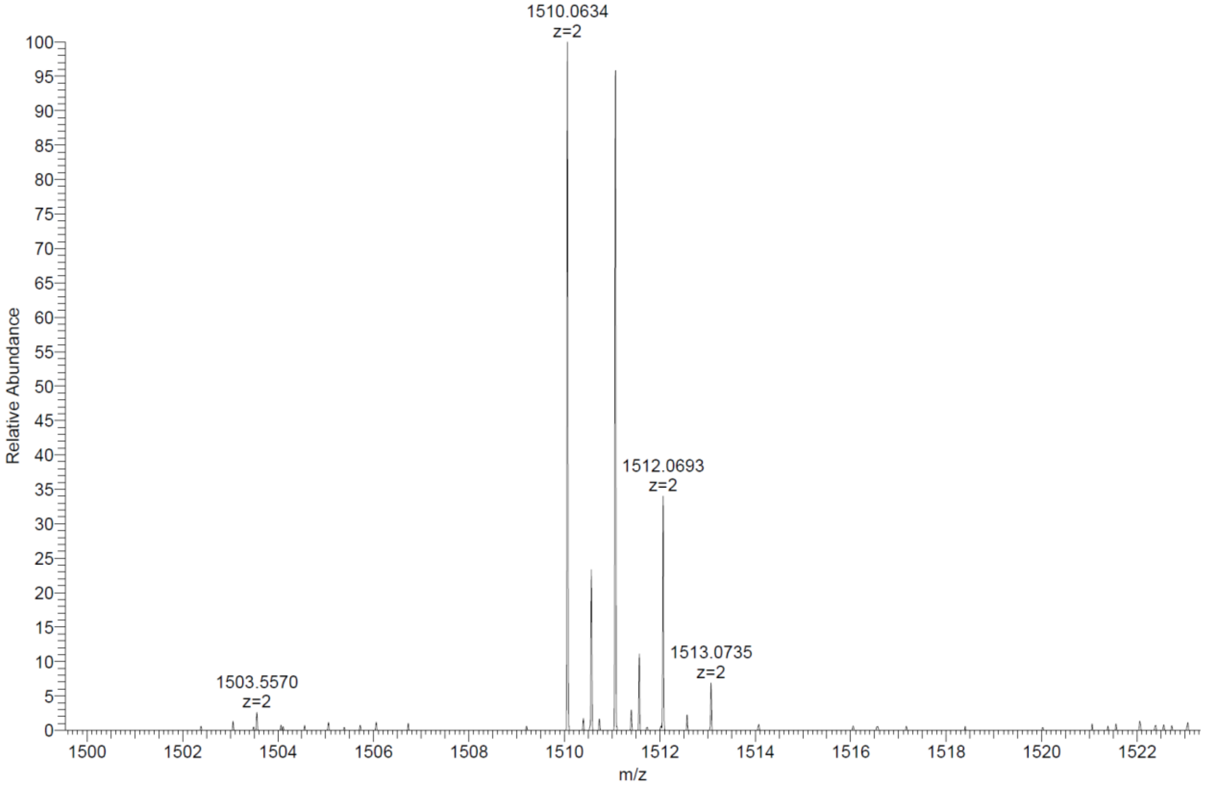

(*)kkLLkLLKLLL (FHP10) was obtained as white solid after preparative RP-HPLC (4.0 mg, 5.4%). Analytical RP-HPLC: t_R = 1.67 min (A/D 100:0 to 0:100 in 3.5 min, λ = 214 nm). MS (ESI+): $C_{74}H_{140}N_{16}O_{16}$ calc./obs. 1510.06/1510.07 Da $[M+H]^+$.

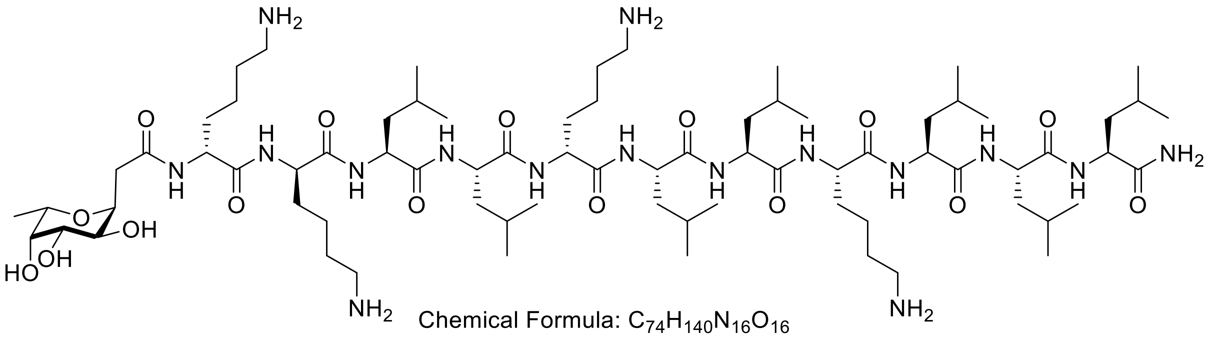

Analytical LC-MS data:

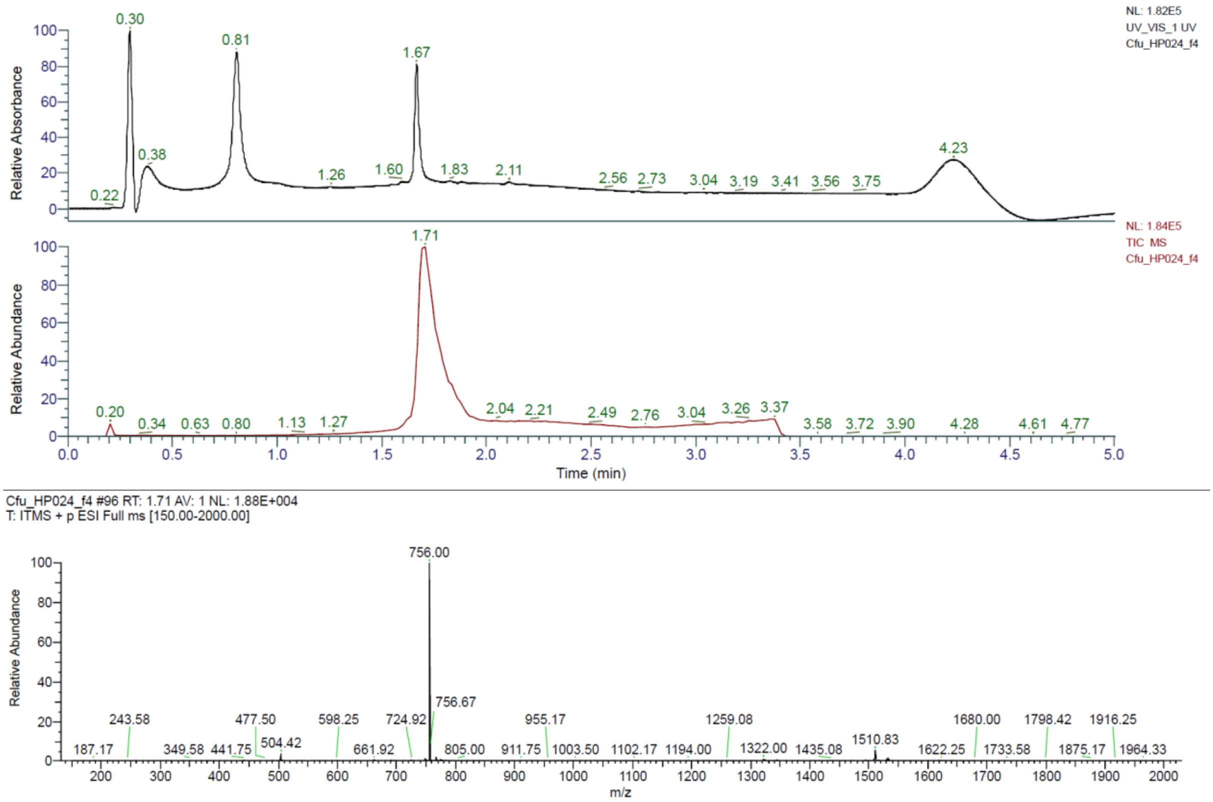

HRMS spectra:

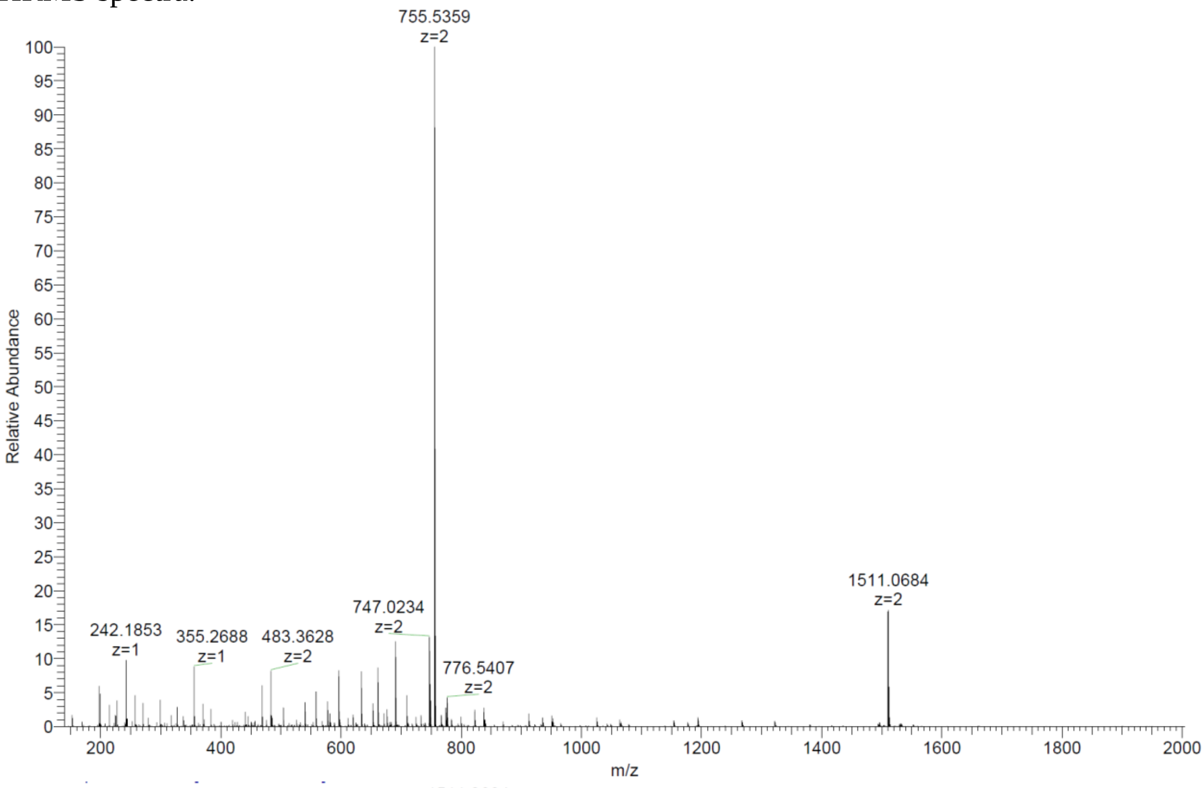

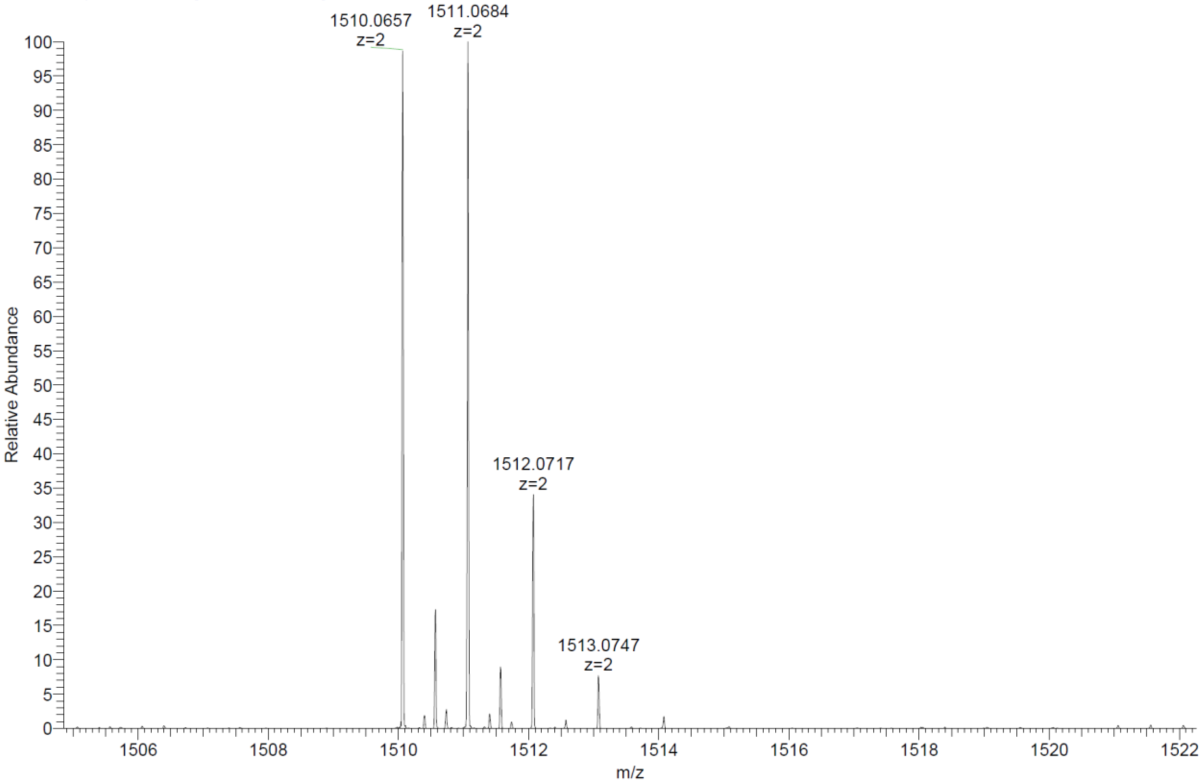

(*)KkllKLlKLLL (FHP11) was obtained as white solid after preparative RP-HPLC (16.9 mg, 17.2%). Analytical RP-HPLC: t_R = 1.63 min (A/D 100:0 to 0:100 in 3.5 min, λ = 214 nm). MS (ESI+): $C_{74}H_{140}N_{16}O_{16}$ calc./obs. 1510.06/1510.07 Da $[M+H]^+$.

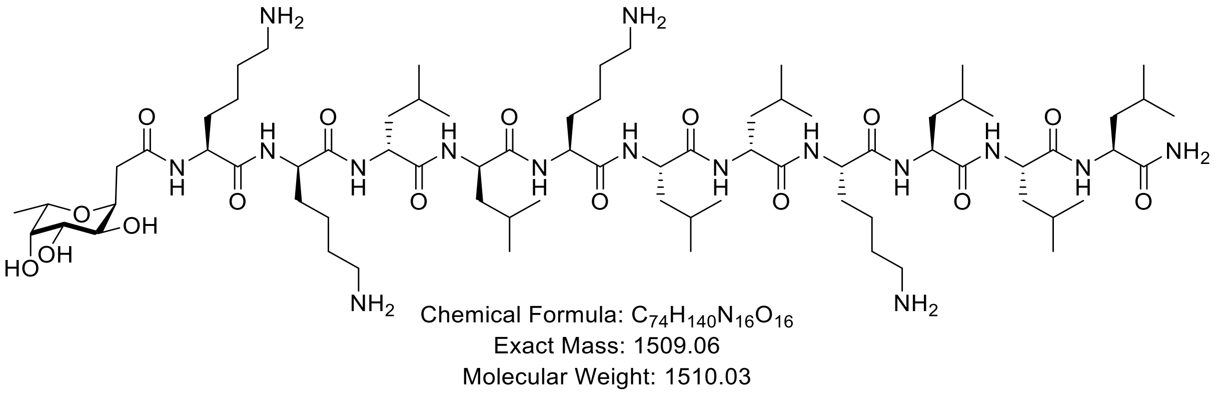

Analytical LC-data:

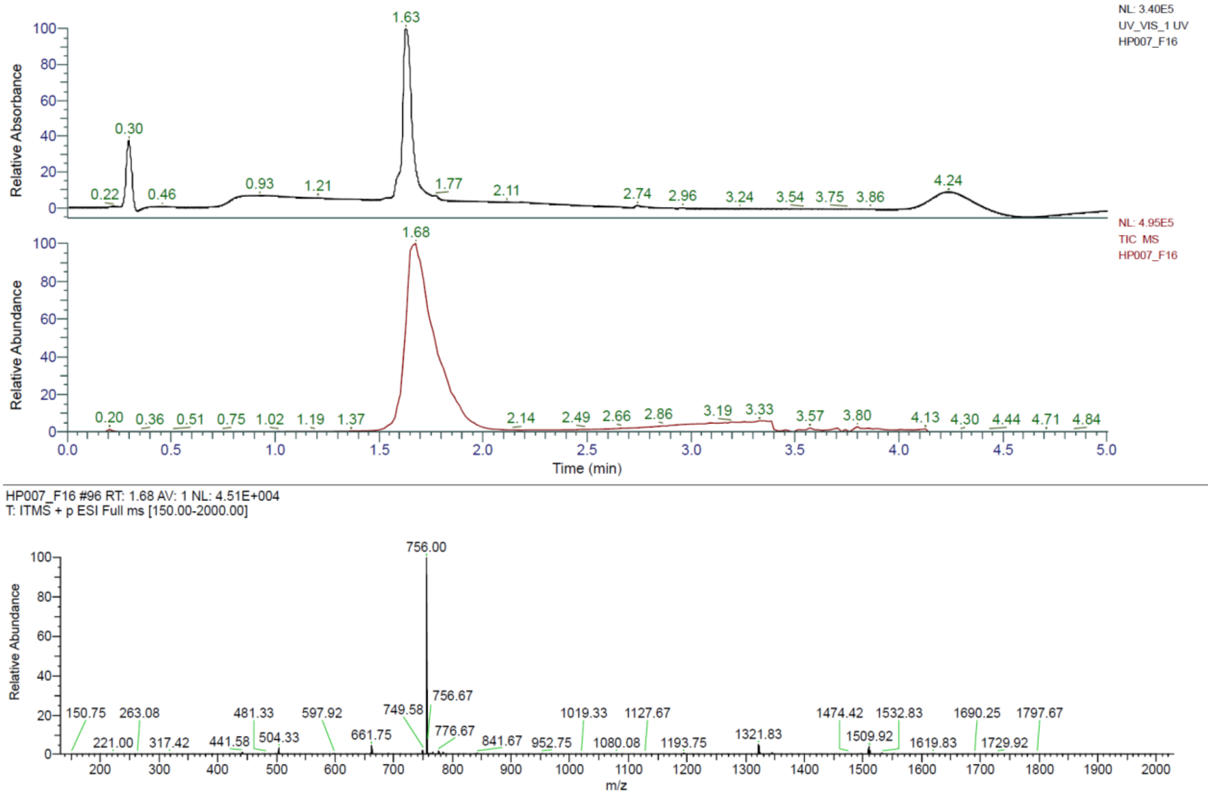

HRMS spectra:

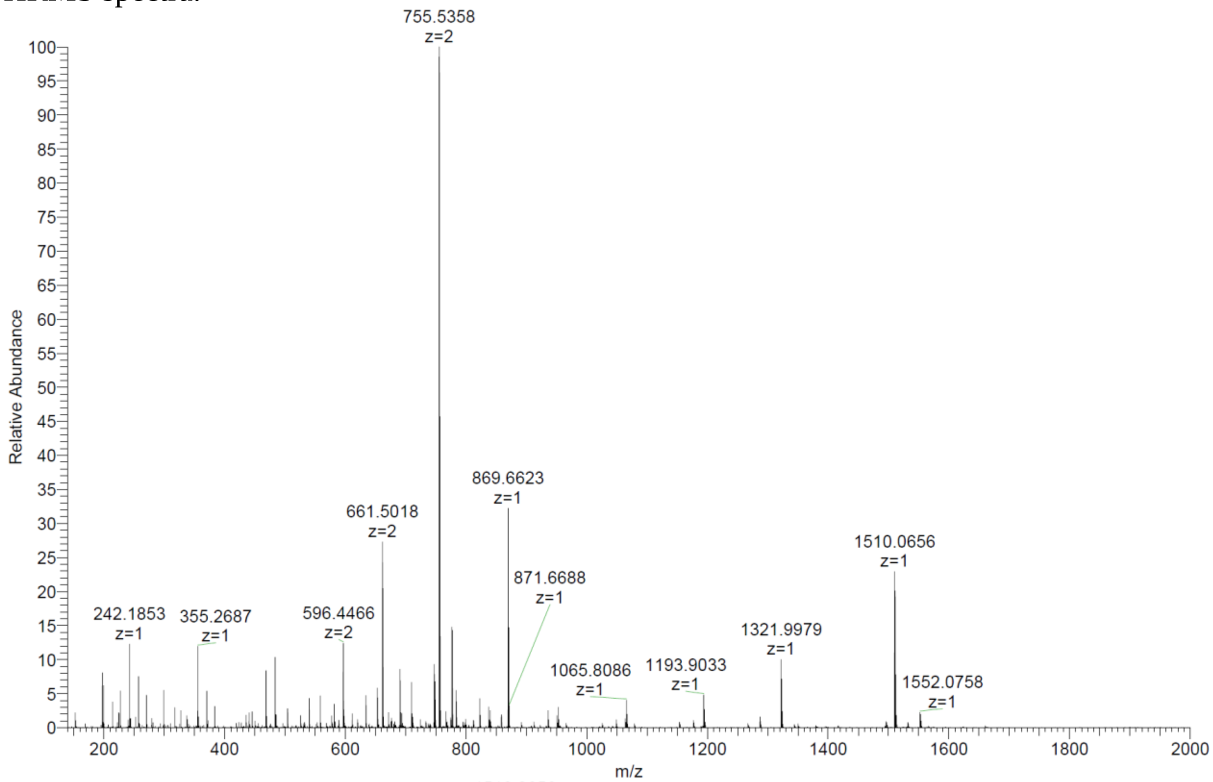

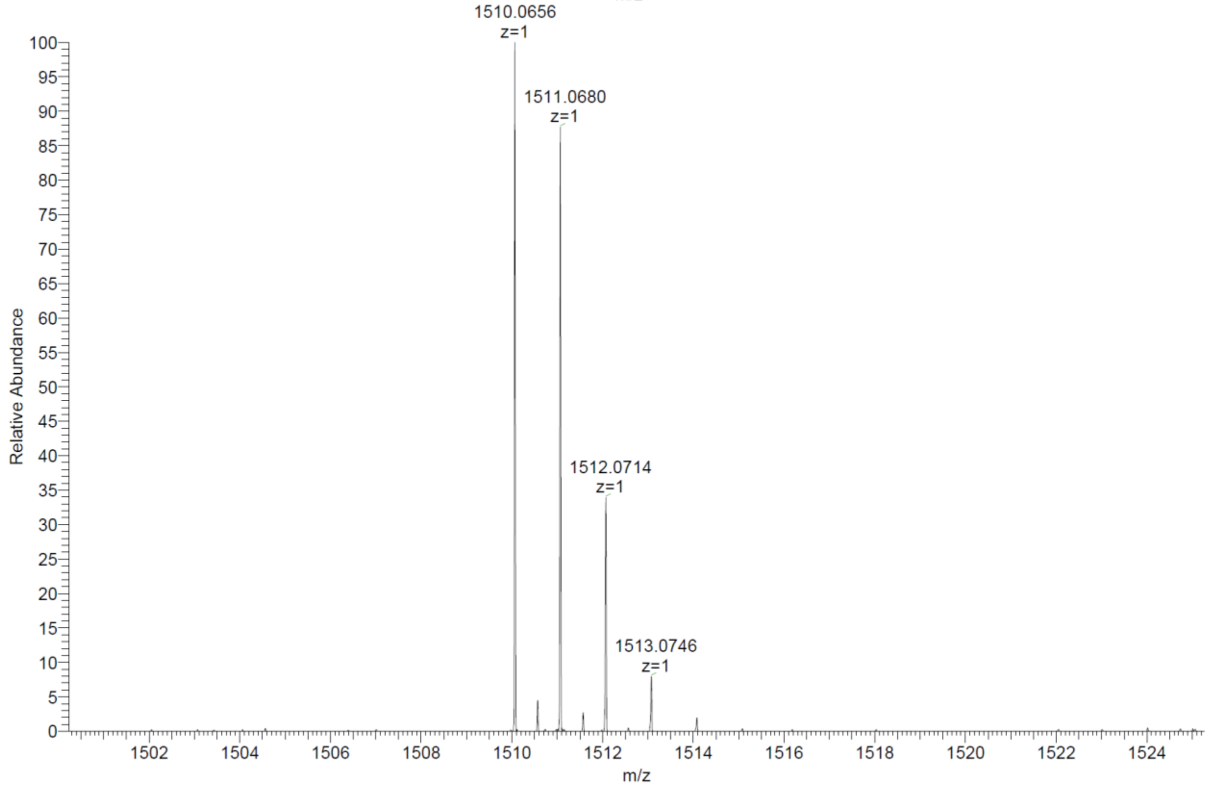

(*)KkLlKlLkLlL (FHP30) was obtained as white solid after preparative RP-HPLC (9.0 mg, 12.2%). Analytical RP-HPLC: t_R = 1.62 min (A/D 100:0 to 0:100 in 3.5 min, λ = 214 nm). MS (ESI+): $C_{66}H_{128}N_{16}O_{11}$ calc./obs. 1510.06/1510.07 Da $[M+H]^+$.

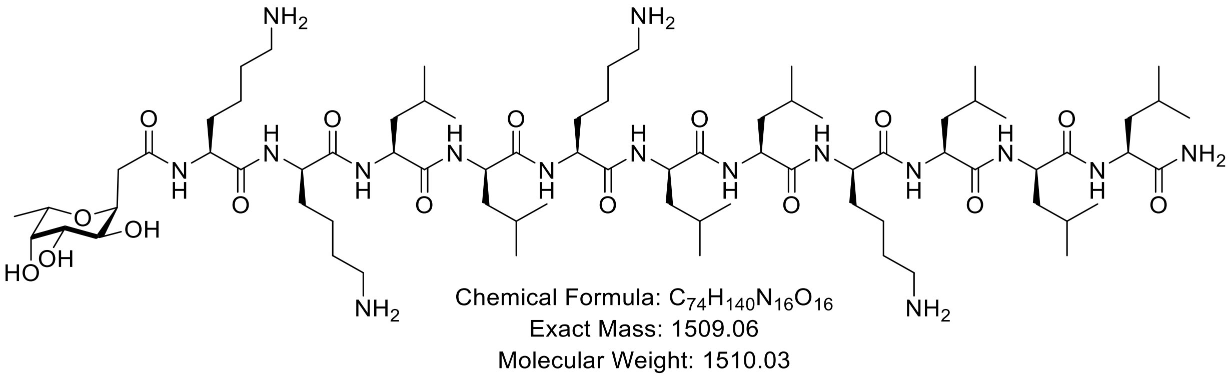

Analytical HPLC-MS data:

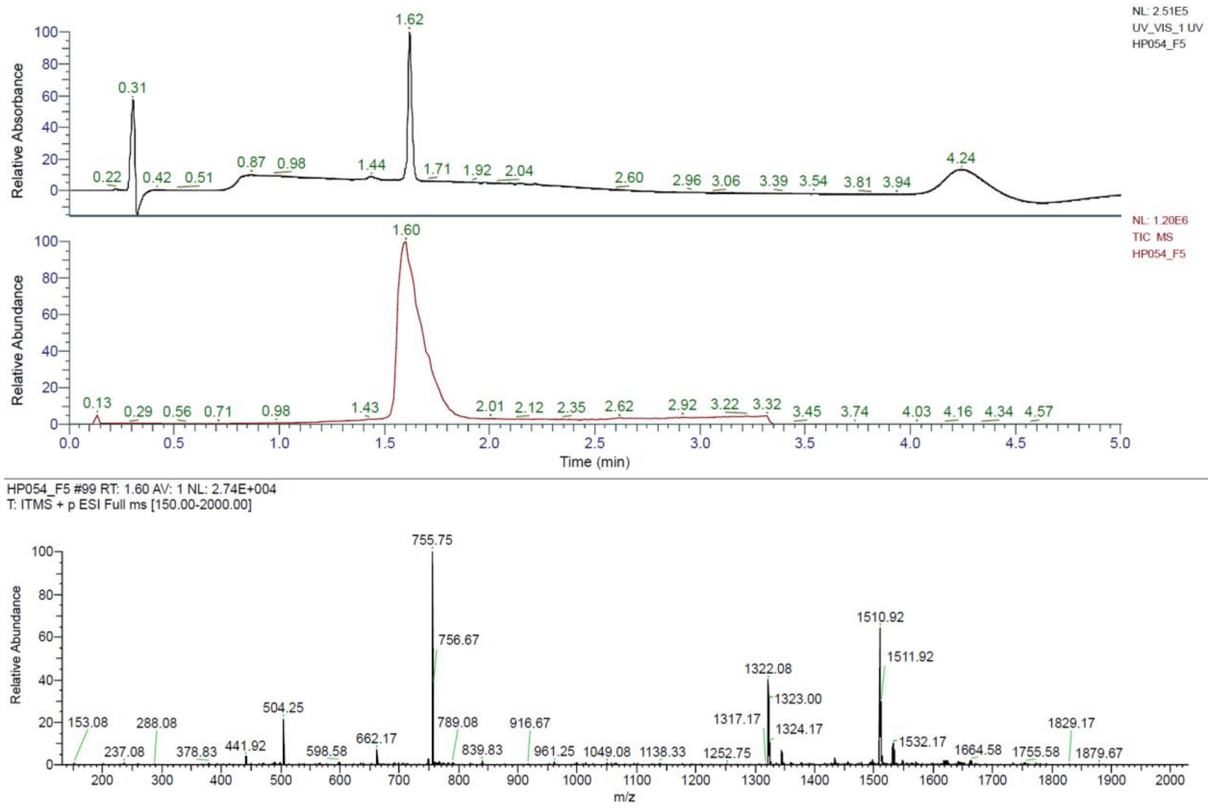

HRMS spectra:

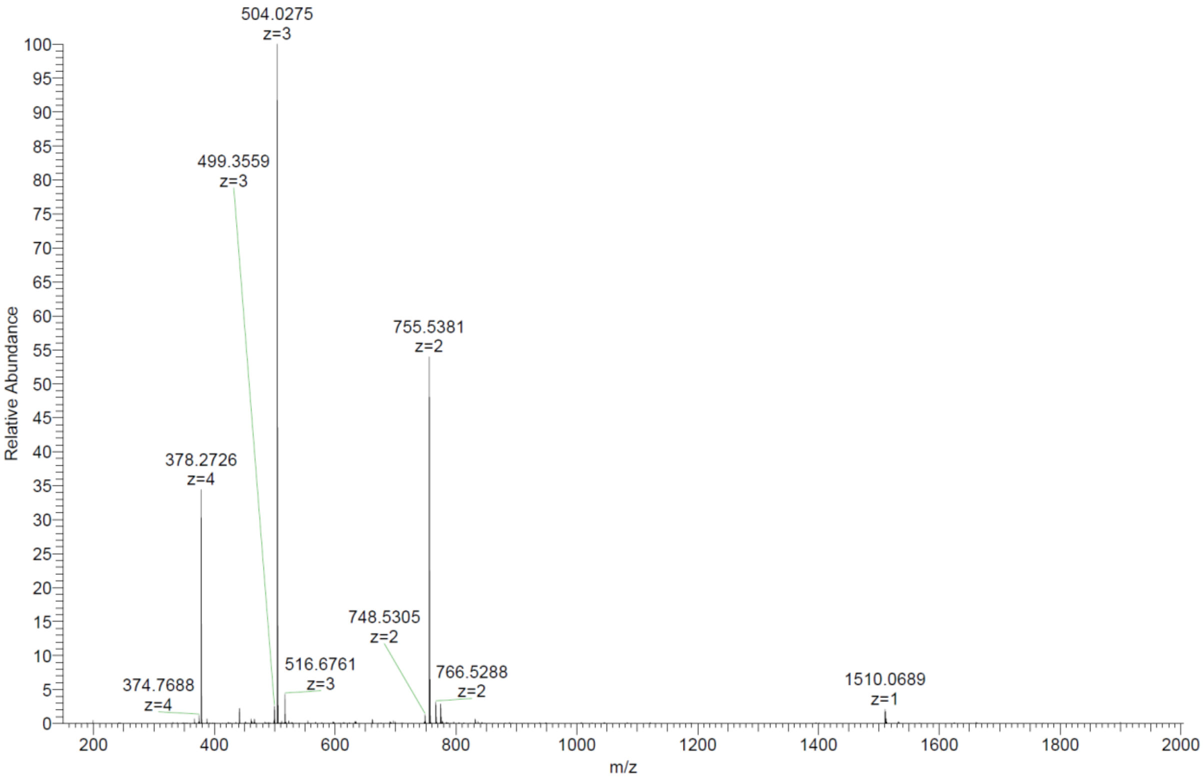

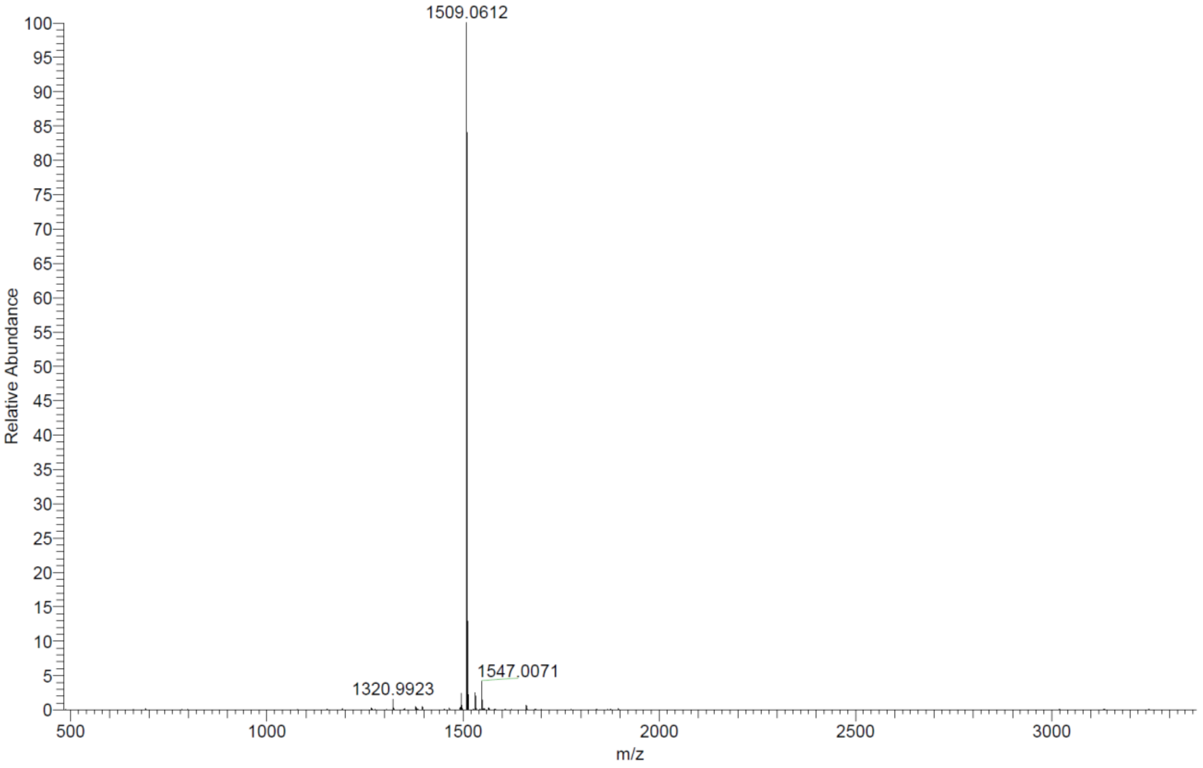

(*)kKlLkLlKlLl (FHP31) was obtained as white solid after preparative RP-HPLC (5.7 mg, 7.7%). Analytical RP-HPLC: t_R = 1.62 min (A/D 100:0 to 0:100 in 3.5 min, λ = 214 nm). MS (ESI+): $C_{66}H_{128}N_{16}O_{11}$ calc./obs. 1510.06/1510.07 Da $[M+H]^+$.

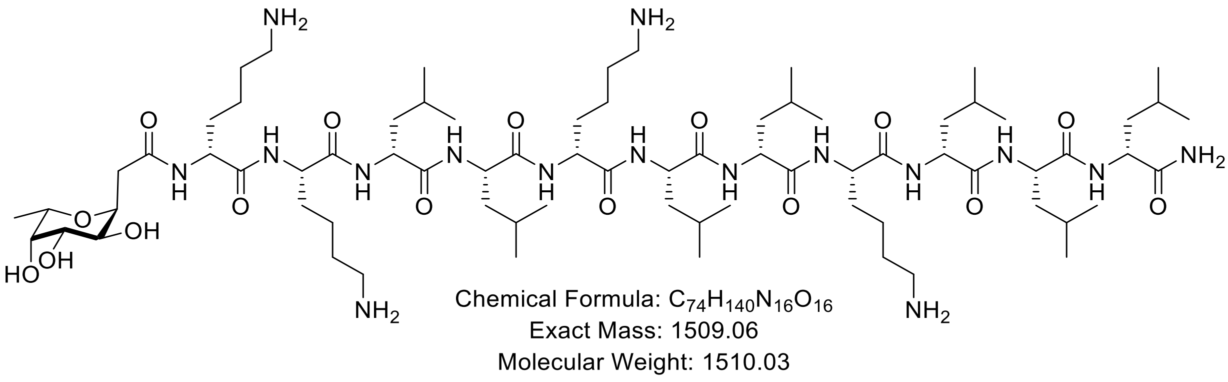

Analytical HPLC-MS data:

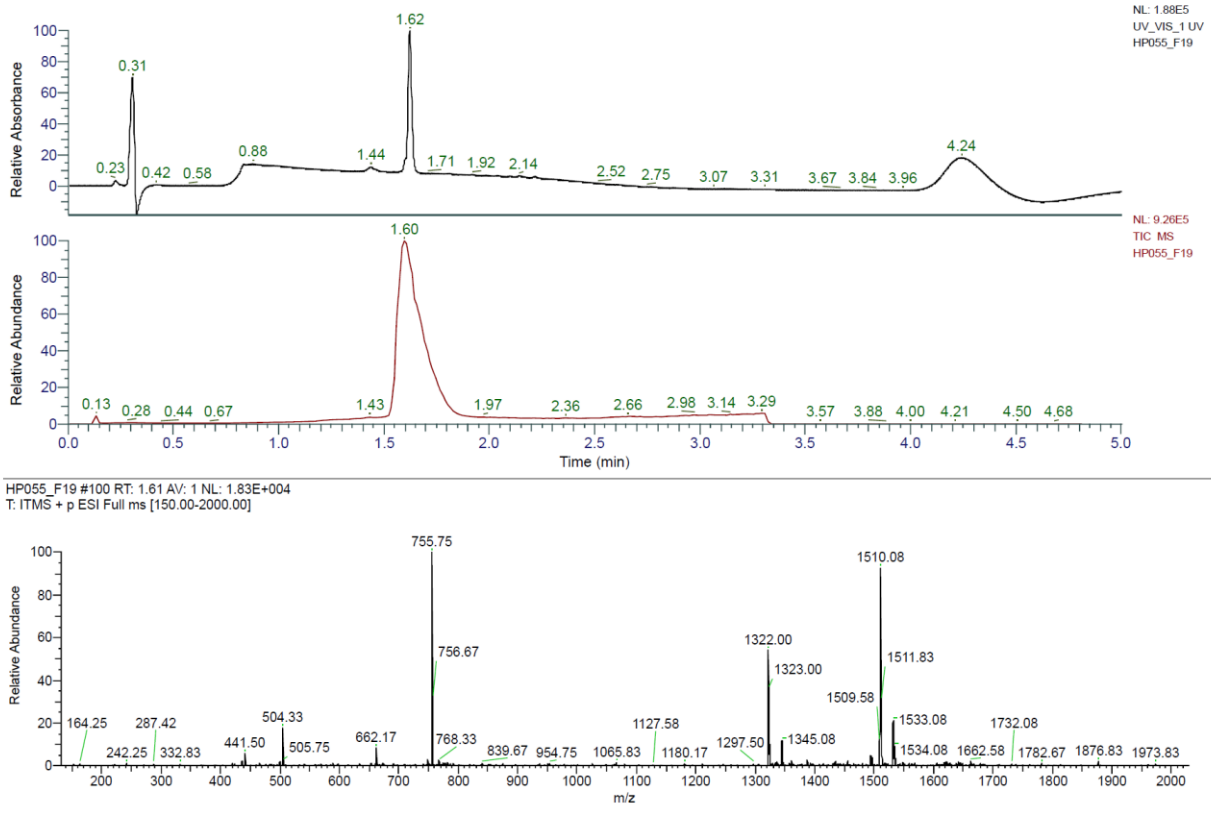

HRMS spectra:

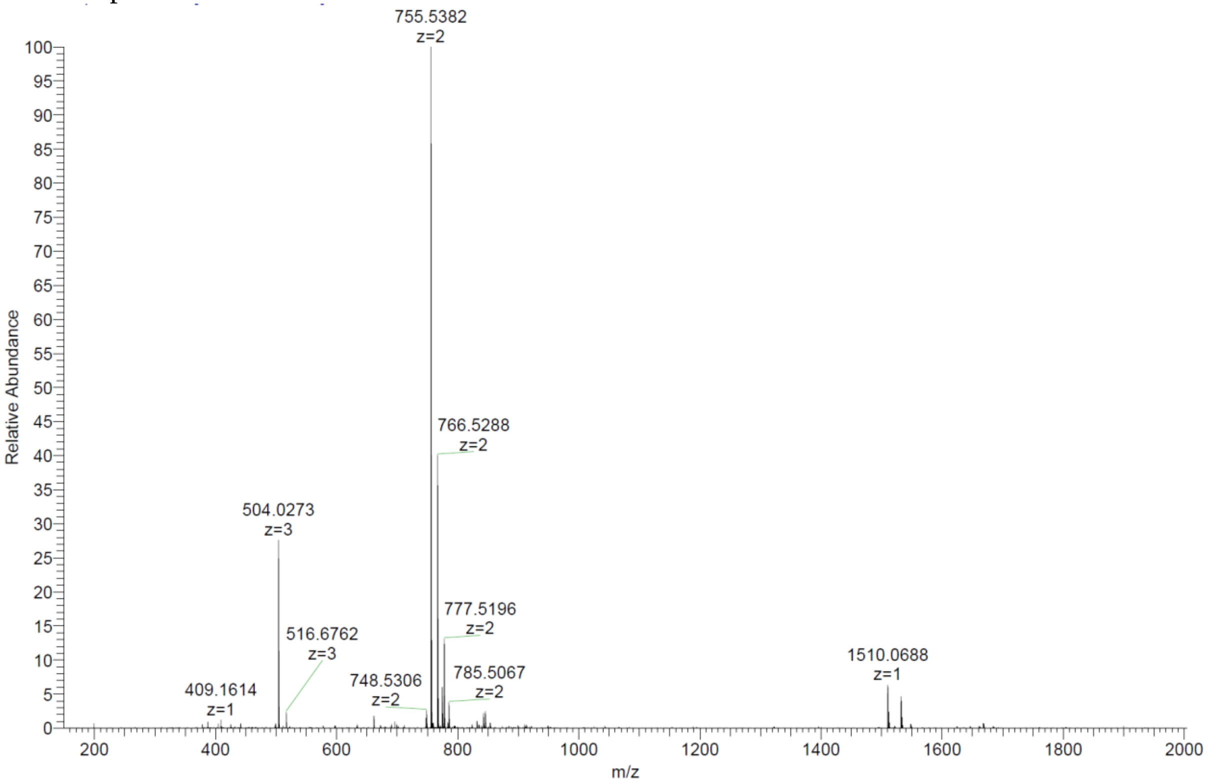

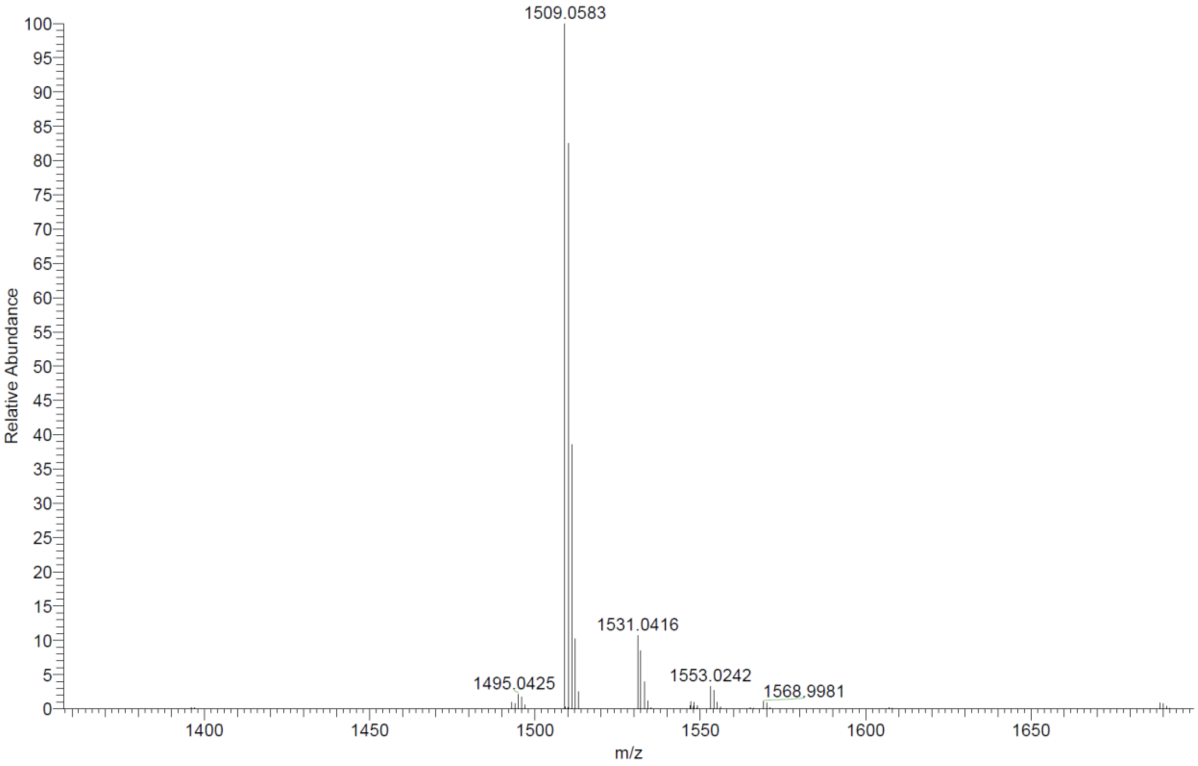

(*)RRLLRLLRLLL (FHP32) was obtained as white solid after preparative RP-HPLC (5.7 mg, 7.3%). Analytical RP-HPLC: t_R = 1.79 min (A/D 100:0 to 0:100 in 3.5 min, λ = 214 nm). MS (ESI+): $C_{74}H_{140}N_{24}O_{16}$ calc./obs. 1621.09/1621.09/ Da [M].

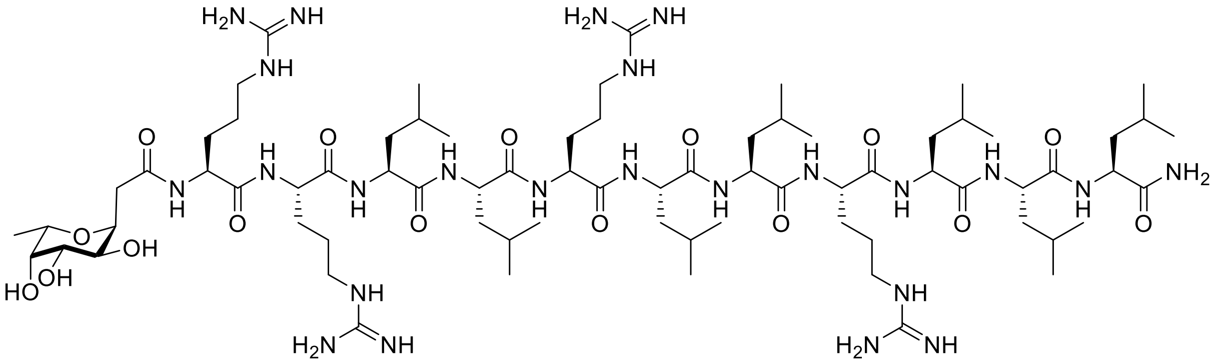

Chemical Formula: $C_{74}H_{140}N_{24}O_{16}$
Exact Mass: 1621.09
Molecular Weight: 1622.09

Analytical LC-MS data:

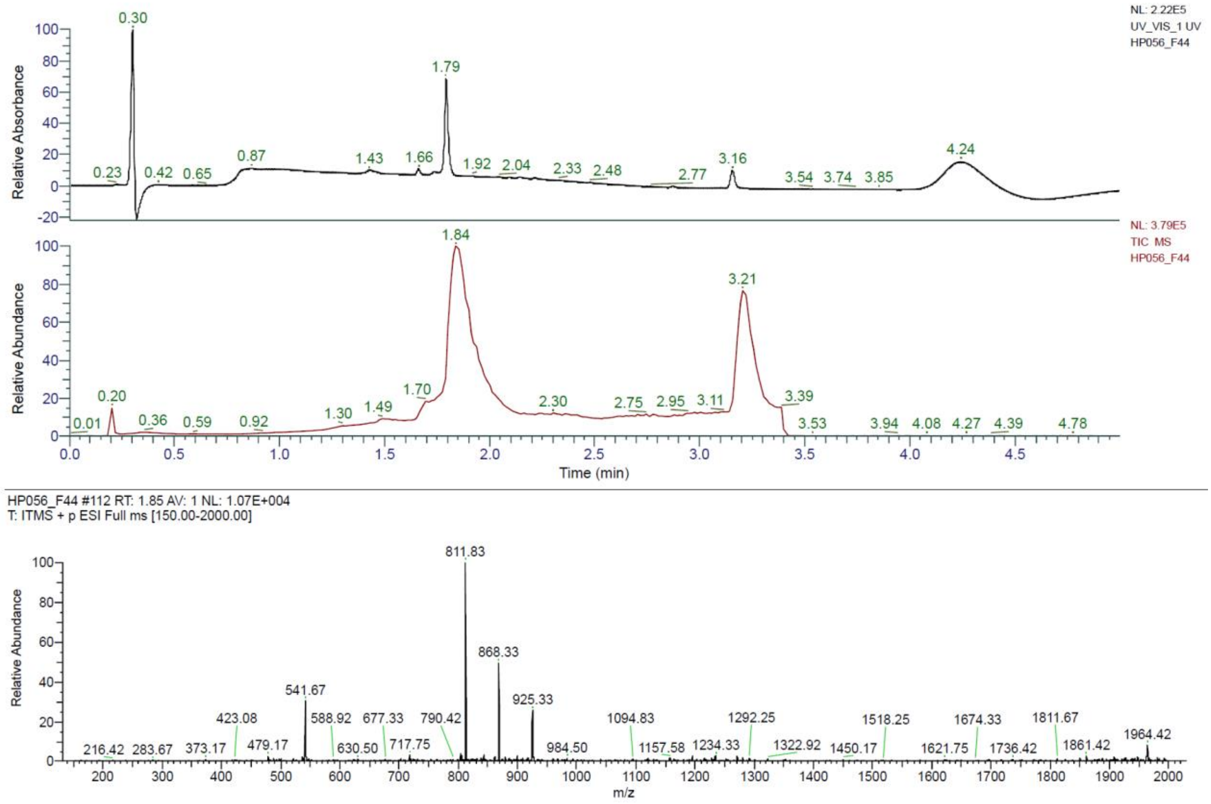

HRMS spectra:

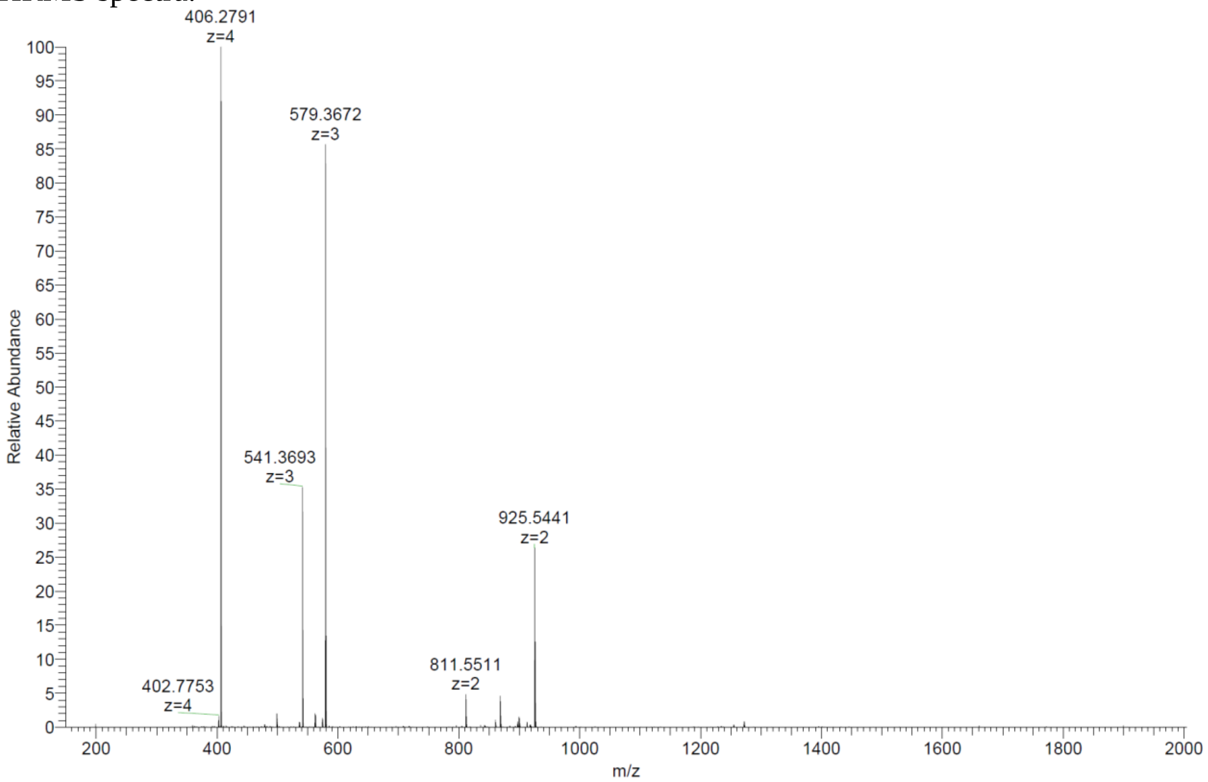

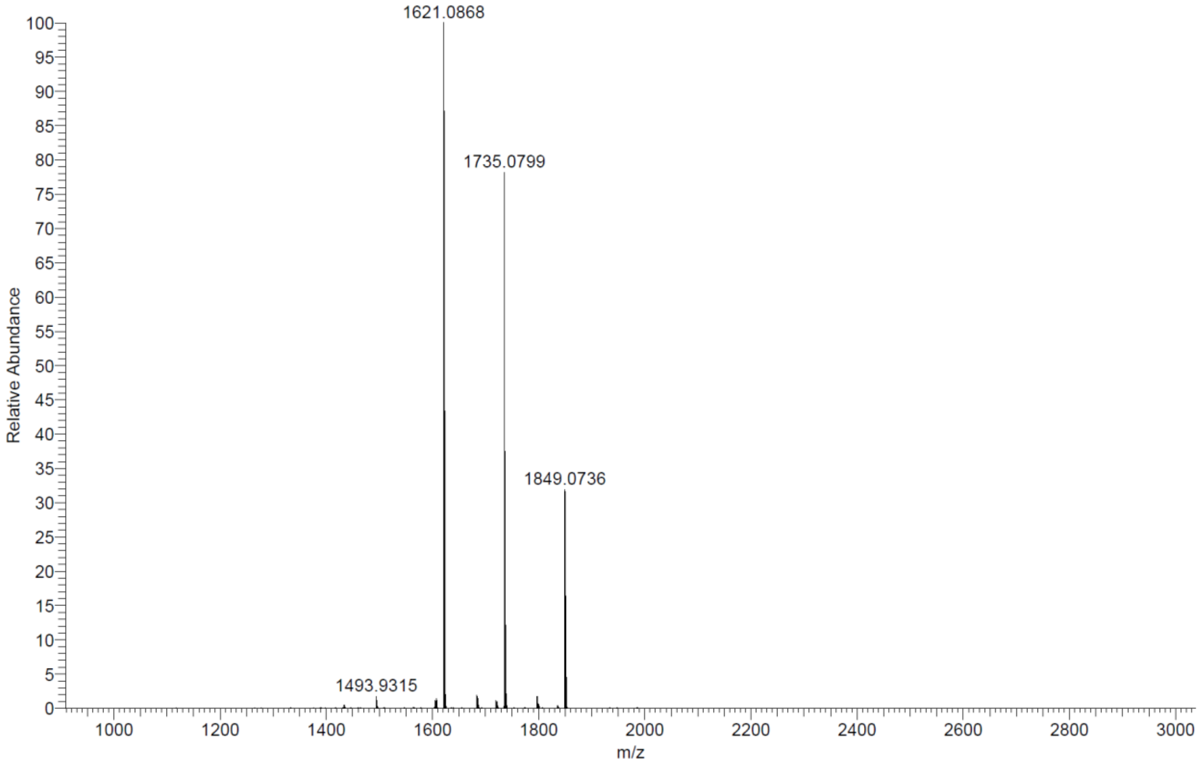

(*)RRLLRLLRLLL (FHP33) was obtained as white solid after preparative RP-HPLC (2.5 mg, 3.2%). Analytical RP-HPLC: t_R = 1.62 min (A/D 100:0 to 0:100 in 3.5 min, λ = 214 nm). MS (ESI+): $C_{74}H_{140}N_{24}O_{16}$ calc./obs. 1621.09/1621.09 Da [M].

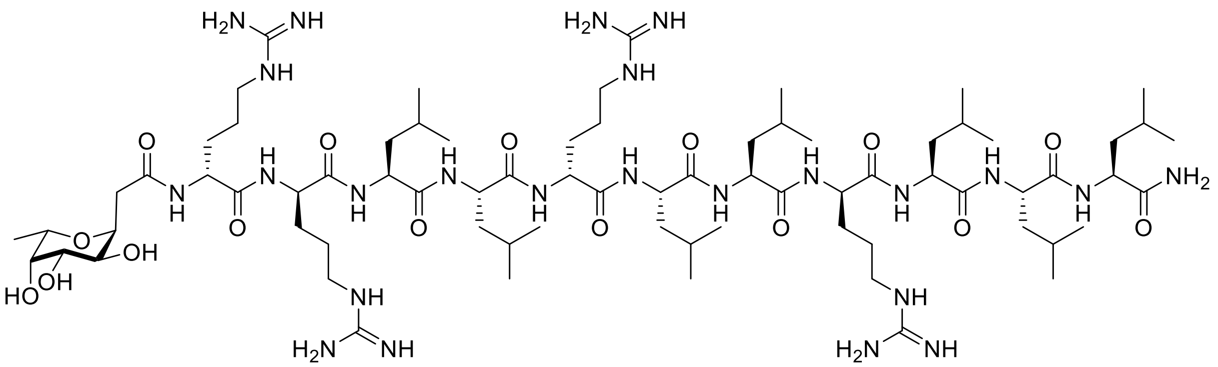

Chemical Formula: $C_{74}H_{140}N_{24}O_{16}$
Exact Mass: 1621.09
Molecular Weight: 1622.09

Analytical LC-MS:

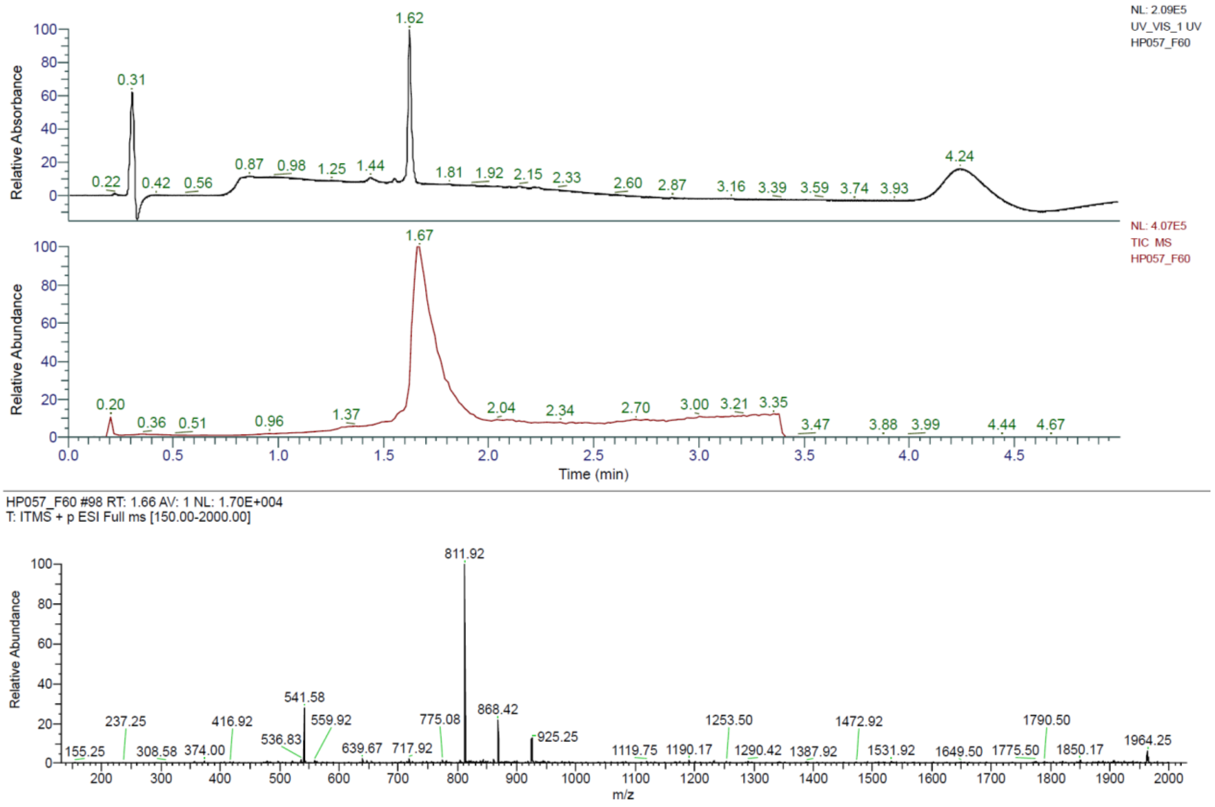

HRMS spectra:

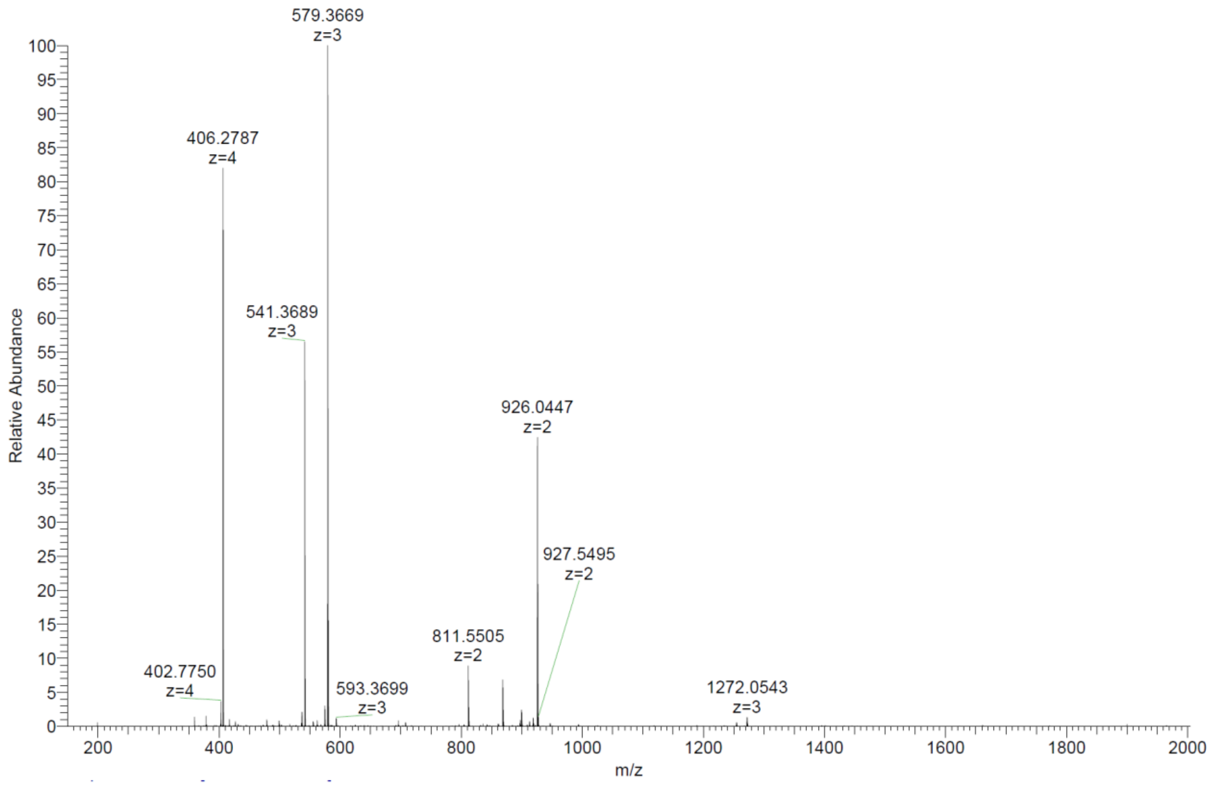

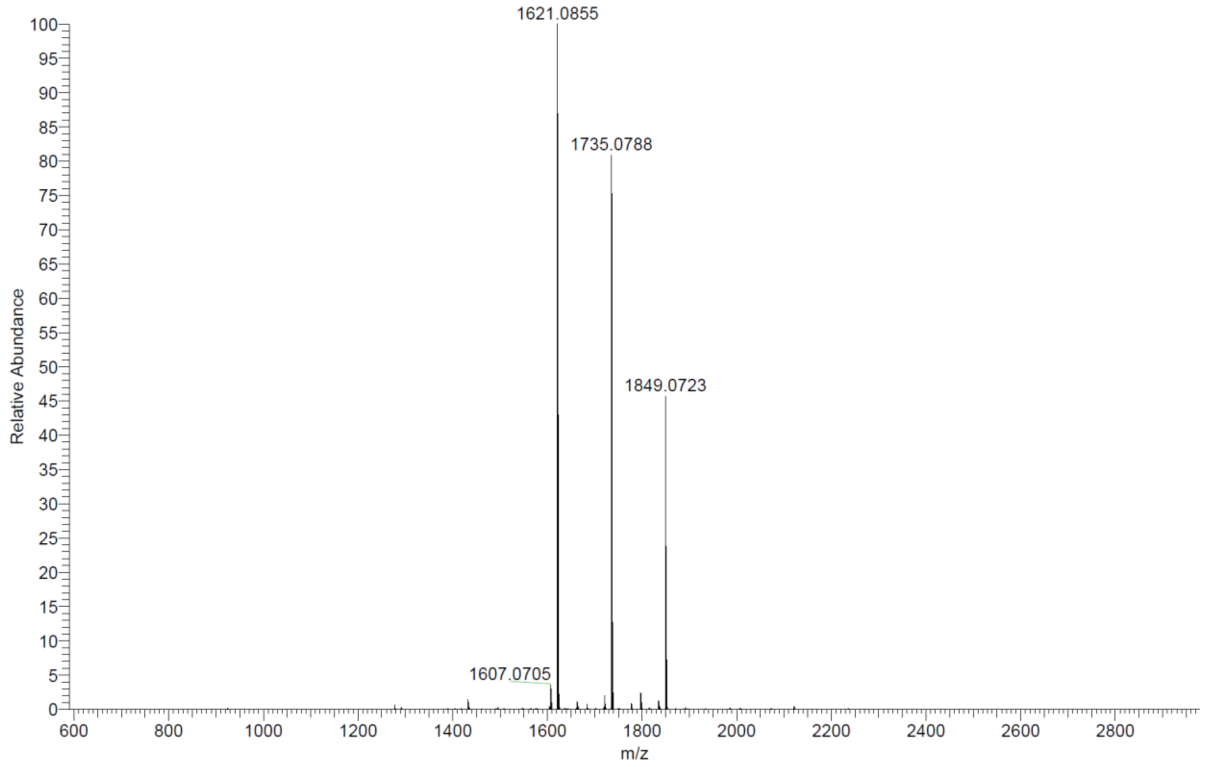

11. References

(1) Provencher, S. W.; Gloeckner, J. Estimation of Globular Protein Secondary Structure from Circular Dichroism. *Biochemistry* **1981**, *20* (1), 33–37. https://doi.org/10.1021/bi00504a006.

(2) Chiu, S. W.; Clark, M.; Balaji, V.; Subramaniam, S.; Scott, H. L.; Jakobsson, E. Incorporation of Surface Tension into Molecular Dynamics Simulation of an Interface: A Fluid Phase Lipid Bilayer Membrane. *Biophys. J.* **1995**, *69* (4), 1230–1245. https://doi.org/10.1016/S0006-3495(95)80005-6.

(3) Probst, D.; Reymond, J.-L. FUn: A Framework for Interactive Visualizations of Large, High-Dimensional Datasets on the Web. *Bioinforma. Oxf. Engl.* **2018**, *34* (8), 1433–1435. https://doi.org/10.1093/bioinformatics/btx760.
